# Enantioselective Giese Additions of Prochiral α-Amino Radicals

**DOI:** 10.1021/jacs.2c11367

**Published:** 2022-12-01

**Authors:** Antti S. K. Lahdenperä, P. David Bacoş, Robert J. Phipps

**Affiliations:** Yusuf Hamied Department of Chemistry, University of Cambridge, Lensfield Road, Cambridge, CB2 1EW, United Kingdom

## Abstract

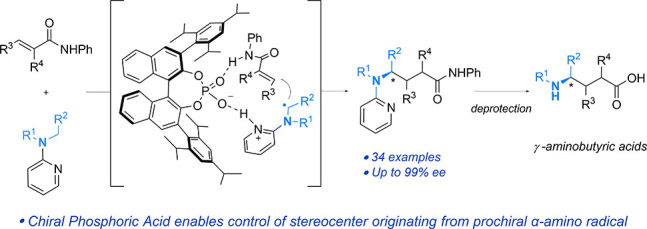

Amines featuring an adjacent stereocenter are important building blocks, and recent years have seen remarkable growth in methods forming these via prochiral α-amino radical intermediates. However, very few can exert control over the newly formed stereocenter. We disclose a strategy to overcome this in the context of one of the most widely used radical carbon–carbon bond forming reactions, the Giese reaction. Incorporation of a removable basic heteroarene into the substrate enables a network of attractive noncovalent interactions between a phosphoric acid catalyst, the subsequently formed α-amino radical, and the Giese acceptor, allowing the catalyst to exert control during the C–C bond forming step. Deprotection of the products leads to analogues of γ-aminobutyric acid. We anticipate that this strategy will be applicable to other asymmetric radical transformations in which catalyst control is presently challenging.

Amines are a ubiquitous feature of compounds for a range of applications, and many feature a stereocenter on a carbon attached to the nitrogen. Enantioenriched chiral amines are typically obtained through hydrogenation of enamides using chiral metal complexes, resolution by formation of diastereomeric salts, or reduction using biocatalysis.^1^ There has been dramatic recent growth in synthetic methods based on radical chemistry, and stabilized α-amino radicals can now be accessed by numerous methods, undergoing countless useful reactions.^2^ Many of these form a new α-amino stereocenter,^3^ but associated strategies to render these enantioenriched have lagged behind conspicuously.^4^ Asymmetric Brønsted acid catalysis has been utilized for several approaches (Figure 1a, light blue arrows). This includes a cationic Brønsted acid catalyst that mediates a radical–radical coupling to form 1,2-diamines^5^ and the use of chiral phosphoric acids to promote enantioselective Minisci reactions^6^ as well as addition to vinylpyridines.^7^ Using transition metal catalysis, prochiral α-amino radicals have been trapped with chiral nickel complexes enabling arylation,^8^ acylation,^9^ and alkylation,^10^ and copper catalysis has been used analogously for cyanation (Figure 1a, dark blue arrows).^11^ These relatively few successful examples, when compared to the very large number of reports that form chiral amines from prochiral α-amino radicals in a racemic manner, emphasize the dearth of strategies for control of these stereocenters. One of the most widely used C–C bond forming radical reactions is the Giese addition of nucleophilic radicals to electron deficient alkenes.^12^ When α-amino radicals undergo addition to acrylate derivatives, the products are particularly important as they are analogues of γ-aminobutyric acid (GABA), the main inhibitory neurotransmitter in the central nervous system and the basis for many pharmaceuticals.^13^ There are numerous examples of racemic Giese-type additions of α-amino radicals^14^ and also a number of enantioselective examples wherein the new stereocenter originates from the β-position of a substituted acceptor.^15^ However, Giese reactions able to control the stereocenter originating from a prochiral α-amino radical are extremely rare (Figure 1a, black arrow). Most enantioselective Giese additions hinge on activation of the electrophile using strategies such as chiral Lewis acids or chiral iminium ions (Figure 1b). These typically preclude control of the forming stereocenter on a prochiral radical as it is too distant from the chiral information—selectivity can only result as a consequence of diastereocontrol if the Giese acceptor features a β-substituent.^14a,15a,15c,16^ A notable exception is the elegant addition of prochiral α-amino radicals to vinylpyridines by Jiang and co-workers; while this can be formally classed as a Giese-type addition, it is limited to vinylpyridines as acceptors and is not transferrable to unsaturated carbonyl compounds.^7,17^

**Figure 1:**
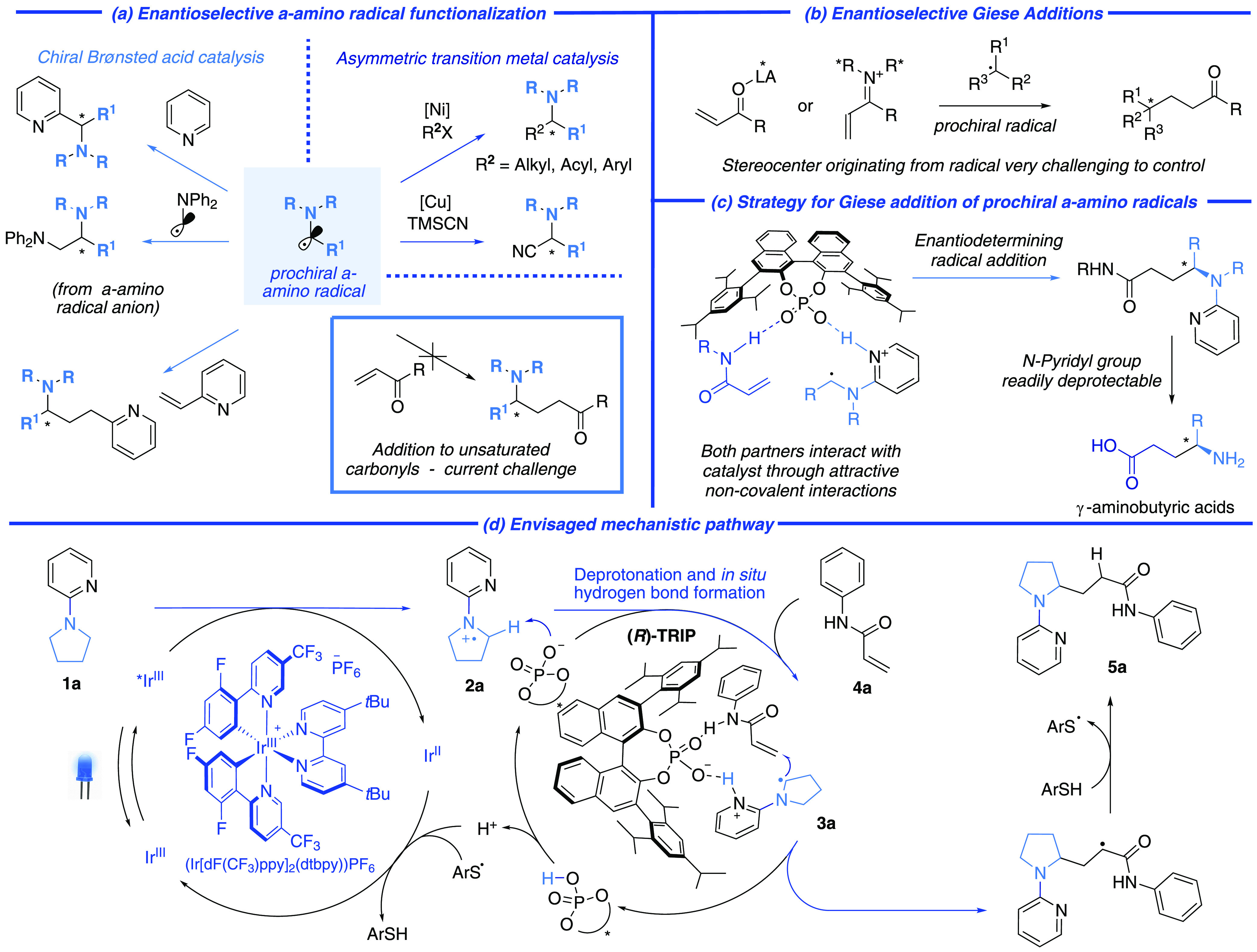
Background, envisaged strategy, and proposed mechanism.

In considering this challenge, we drew upon our prior experience in control of enantioselectivity in the Minisci reaction.^6a,6c,6e,6f^ There, having a basic heteroarene as the electrophile permitted association with a Chiral Phosphoric Acid (CPA) catalyst through protonation, providing orientation with the incoming α-amino radical via a combination of hydrogen bonding and electrostatic interactions. We imagined adapting this to allow the orchestration of asymmetric Giese additions by instead incorporating the basic heteroarene into the structure of the nucleophilic α-amino radical, providing association with the chiral catalyst through protonation (Figure 1c). For the Giese acceptor, derivatization as a secondary amide would provide a hydrogen bond donor to function as the second interaction point to assemble a highly organized transition state for presumed enantiodetermining radical addition.^12a,18^ Deprotection of the pyridyl group and amide hydrolysis would lead to the γ-aminobutyric acid. The 2-pyridylamine motif has been used with transition metals as a removable directing group, but to the best of our knowledge has not been explored in radical transformations or in combination with organocatalysts such as CPAs.^19^ Mechanistically, we envisaged that a cationic iridium photocatalyst would oxidize the starting amine **1a** (Figure 1d, left cycle). The presence of chiral phosphate anion in solution should allow anion exchange such that this becomes the conjugate anion of amine radical cation **2a**, deprotonation of which by the associated phosphate gives α-amino radical **3a**. We anticipate that the formed phosphoric acid would be deprotonated by the pyridine and these would interact through hydrogen bonding and electrostatic interactions, locating the α-amino radical inside the chiral pocket of the catalyst. With the Giese acceptor **4a** interacting with the phosphoryl oxygen of the catalyst through hydrogen bonding, a highly organized transition state may be achieved (Figure 1d, right cycle). Inclusion of a thiol cocatalyst (ArSH) would allow hydrogen atom transfer (HAT), forming **5a**, and the thiyl radical produced would enable turnover of the photoredox cycle.

We commenced with amine **1a**, *N*-phenylacrylamide (**4a**), (Ir[dF(CF_3_)ppy]_2_(dtbpy))PF_6_ as photocatalyst and (*R*)-TRIP as the CPA. Twenty-five mol% of 2,4,6-triisopropylbenzenethiol was used as the thiol cocatalyst. While the yield of product **5a** was low, potentially due to competitive polymerization of **4a**, it was obtained with a highly encouraging 84% ee (Scheme 1a). Both yield and enantioselectivity were improved by the inclusion of a substituent at the acceptor α-position, resulting in **5b**. No diastereocontrol was observed during the quenching of the α-carbonyl radical by the thiol, but remarkably the diastereomers exhibited distinct retention times on silica, allowing **5ba** and **5bb** to be isolated separately in good yield, with 90% and 94% ee, respectively. We believe that this is due to differing extents of intramolecular hydrogen bonding in the two diastereomers (see SI). These diastereomers could be individually deprotected through a telescoped sequence of methylation, reduction, and hydrolysis to obtain the γ-aminobutyric acid HCl salts (see SI).

**Scheme 1:**
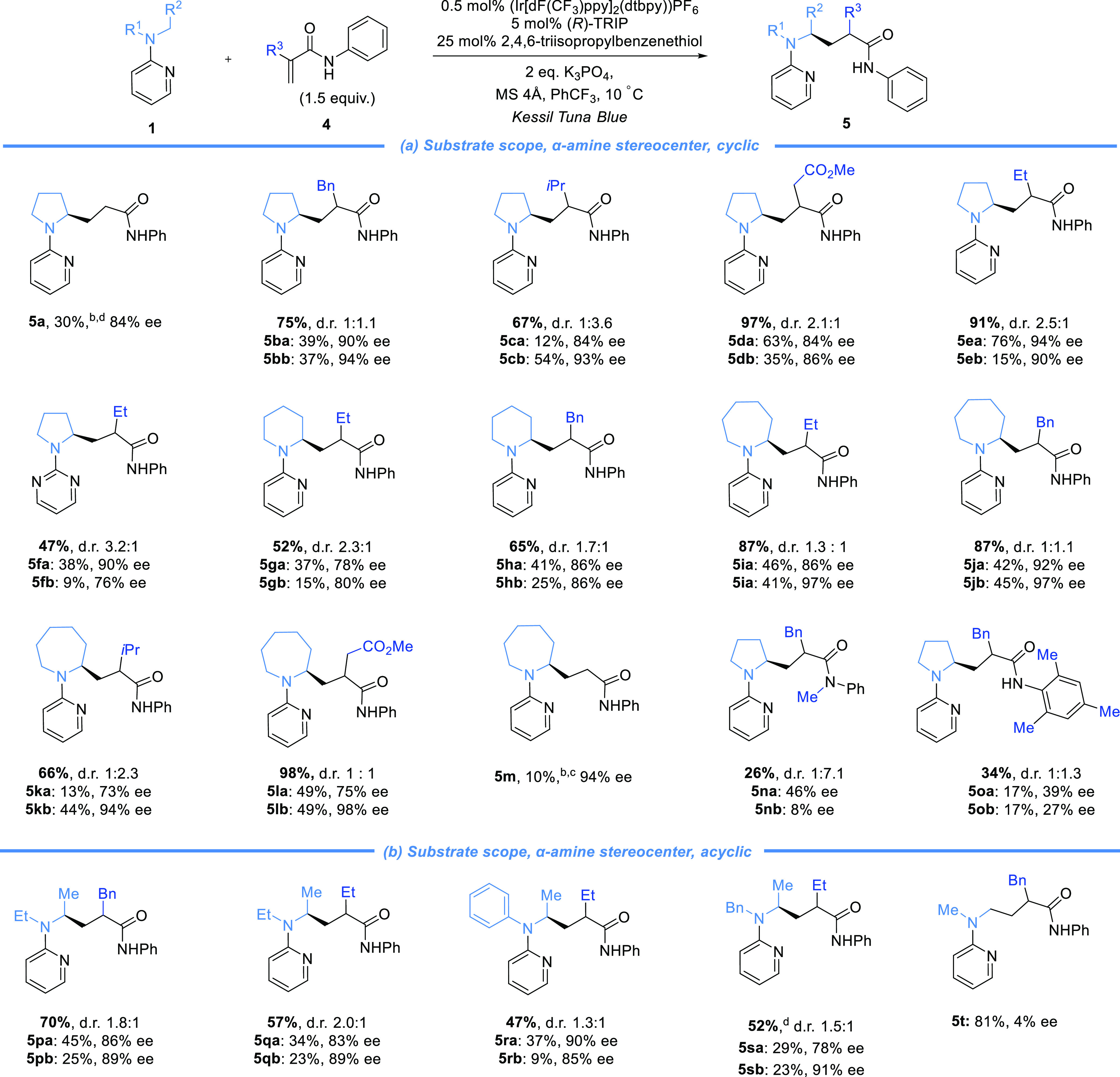
Scope Demonstrating Control of Stereochemistry Derived from the Prochiral α-Amino Radical Total yield (in bold) refers to the combined yield of the two separately isolated diastereomers. The diastereomeric ratio (d.r.) was determined by crude ^1^H NMR analysis. Subsequent yields and enantioselectivities are reported for each isolated diastereomer. Reaction performed with 1.25 equiv of **1**, 1 equiv of *N*-phenylacrylamide and at −40 °C. One mol % (Ir[dF(CF_3_)ppy]_2_(dtpby))PF_6_. Three mol % (Ir[dF(CF_3_)ppy]_2_(dtpby))PF_6_.

We first evaluated the scope of cyclic amines (Scheme 1a). Various α-substituted acceptors worked well in reaction with pyrrolidine-derived amine **1a** (**5b**–**5e**). There was little control of the second stereocenter, but the two diastereomers could be separately isolated, giving single diastereoisomers in very high ee. A pyrimidine directing group could be used (**5f**), and piperidine-based amines (**5g** and **5h**) and azepane-derived amines (**5i** – **5l**) were compatible. Although the yield was low, excellent ee could be obtained for an azepane substrate with the unsubstituted acceptor **4a**, underlining the high level of stereocontrol at the α-amino stereocenter (**5m**). We tested several acceptors in which the ability to form a hydrogen bond with the catalyst is either removed, with use of *N*-methylated acrylamide **5n**, or likely impaired by use of a bulky *N*-mesityl amide **5o**. Both outcomes were greatly inferior. We then examined acyclic amines (Scheme 1b) and were pleased to find that an *N*,*N*- diethyl substrate gave excellent outcomes (**5p** and **5q**), as did a substrate with an *N*-phenyl group on the amine (**5r**). The substrates evaluated so far led to secondary amine products upon pyridyl deprotection, and we sought a way to access primary amines. This was achieved using an *N*-benzyl, *N*-ethyl substrate in which good yields were obtained for Giese addition at the α-position of the ethyl group (**5s**). In this case, the benzyl position remained unfunctionalized. Product **5t** was close to racemic, clearly demonstrating that catalyst control is occurring exclusively at the α-amino position.

Having demonstrated control at the stereocenter originating from the α-amino radical, we next sought to evaluate control at the β-position of the acceptor (Scheme 2a). We combined simple *N,N*-dimethyl pyridylamine with a β-methyl-substituted acceptor and observed excellent enantioselectivity (**6a**). An *N*-phenyl variant performed similarly well (**6b**, **6c**) and an electron-withdrawing CF_3_ group could be tolerated on either the aromatic ring of the amine radical precursor or the acrylamide (**6d**, **6e**). The deprotectable *N*-benzyl group could be used on the amine (**6f** and **6g**), and heteroatom functionality could be incorporated into the amine (**6h**). A phenyl ring at the acceptor β-position gave reduced enantioselectivity, however (see SI). To probe substrates that generate diastereomers, we tested an α,β-dimethyl acceptor (**6i**). As before, control in the terminating HAT was low, but the diastereomers could again be isolated separately and this could also be applied to a cyclic acceptor (**6j**). For prochiral amines with β-substituted acceptors, diastereocontrol was improved (Scheme 2b). *N*-Pyridyl azetidine combined with a β-*iso*propyl acceptor gave 5:1 d.r. (each diastereomer isolated in 99% ee, **6k**), while *N*-pyridyl azepane gave 13:1 d.r. (98% ee, **6l**). In comparison, acyclic amines were found to be insufficiently reactive (see SI). We finally explored the possibility of controlling all three stereocenters (**6m**–**6o**). Remarkably, excellent diastereocontrol was achieved in two cases and in all the major diastereomer was obtained as a single compound in excellent enantioselectivity. We next targeted pregabalin, a blockbuster anticonvulsant prescribed as a single enantiomer (Scheme 2c).^20^ Our protocol delivered **6p** in 99% ee, and a deprotection sequence consisting of three telescoped steps (90% yield overall) gave *ent*-pregabalin **6pa**. We also completed the formal synthesis of the pyrrolizidine alkaloid (−)-pseudoheliotridane (**6qc**) (Scheme 2d).^21^ Following Giese addition, the desired diastereomer **6q** was obtained with acceptable diastereoselectivity (90% ee for major diastereomer). Removal of the pyridyl group preceded lactam formation to give **6qb**.^22^

**Scheme 2:**
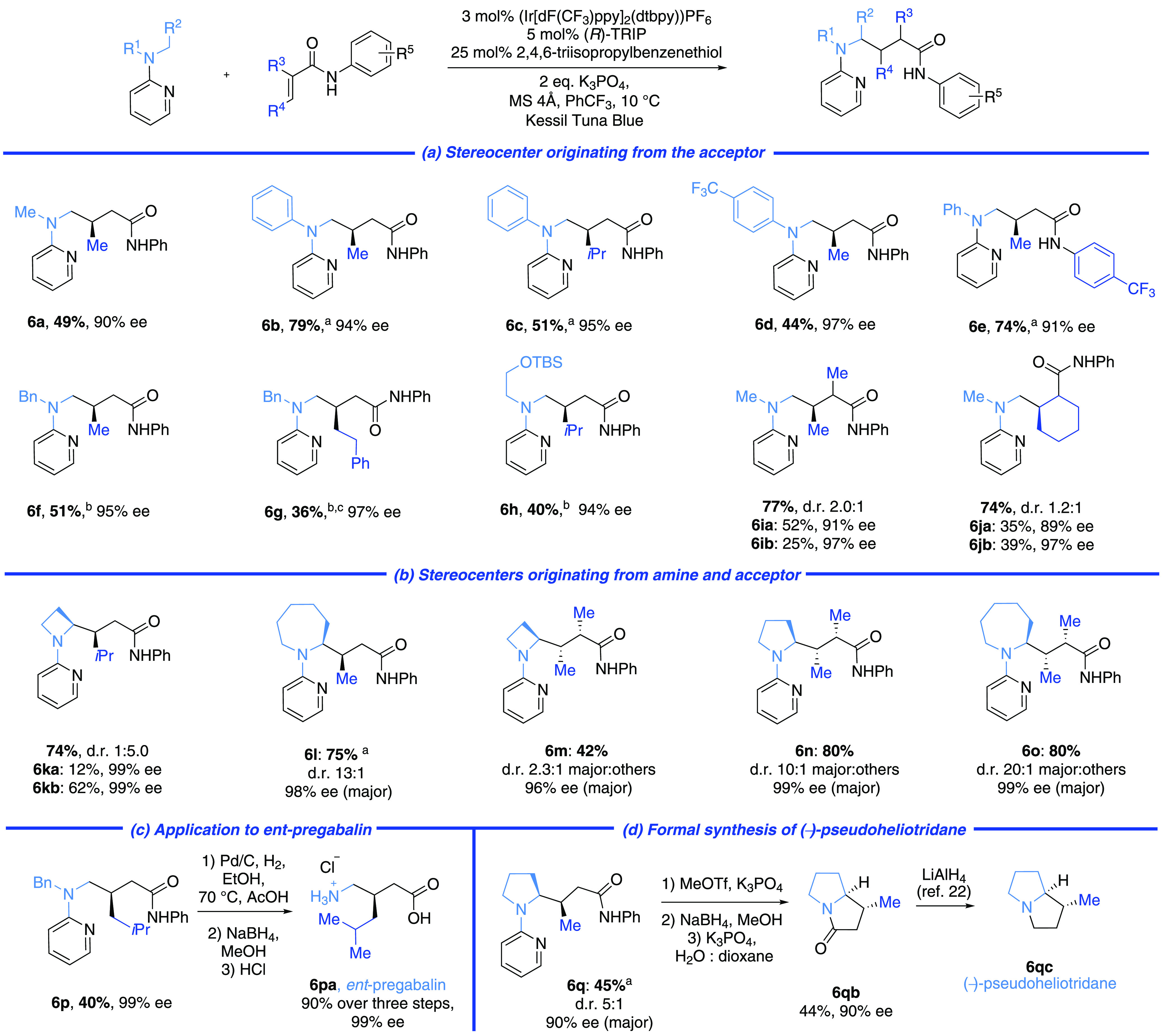
Scope Demonstrating Control of Stereochemistry Derived from the Acceptor β-Position and Applications One mol % (Ir[dF(CF_3_)ppy]_2_(dtpby))PF_6_. Five mol % (Ir[dF(CF_3_)ppy]_2_(dtpby))PF_6_. Reaction performed in CH_2_Cl_2_.

To probe the mechanism of the reaction, we first established that use of the 2-isomer of the pyridyl group is crucial to both reactivity and selectivity (Scheme 3a). The 3-isomer resulted in a low yield of diastereomeric products (**7aa**, **7ab**) derived from Minisci-type addition of the intermediate α-carbonyl radical to the pyridine, with very low enantioselectivity. The 4-pyridyl isomer gave no Giese product suggesting that, despite electronic similarity with the 2-isomer, the proximity of the α-amino radical to the heteroatom is crucial. We next probed each reaction component systematically (Scheme 3b). Without (*R*)-TRIP, no product was obtained, demonstrating negligible background reaction (entry 2). With neither (*R*)-TRIP nor thiol, reactivity was restored, suggesting that the thiol plays an essential role in preventing a pathway leading to noncatalyzed, racemic background reaction (entry 3). This scenario was supported by performing the reaction with (*R*)-TRIP but without thiol, leading to excellent yield, but significantly reduced enantioselectivity (entry 4 vs entry 1). The tetra-*n*-butylammonium salt of (*R*)-TRIP was evaluated in the absence of K_3_PO_4_, and this gave only slightly reduced enantioselectivity, demonstrating that the stoichiometric inorganic base is not playing a significant role in stereoinduction (entry 5). Interestingly, the use of simple BINOL-derived phosphoric acid as its tetra-*n*-butylammonium salt (to promote solubility) gave both poor yield and ee, showing that the lipophilic “arms” of TRIP are crucial to both reactivity and enantioinduction (entry 6 vs entry 5). Circumstantial support for the facile association of the reaction components was gained by X-ray crystallographic analysis of a ternary complex of **4d**, (*R*)-TRIP and **1a**, crystals of which were formed by simply mixing the three in CH_2_Cl_2_/*n*-hexane (Scheme 3c). Although this does not include the radical intermediate and cannot provide information relating to the transition state, it neverthless helps to visualize how the reaction components assemble through hydrogen bonding and electrostatic interactions, under the guidance of the chiral phosphate.^23^ We believe that the thiol is suppressing racemic background reaction by quenching, via HAT, α-amino radicals that are not complexed with the catalyst. The bulky phosphate creates a sheltered chiral pocket, inside which this quenching is prevented and in which productive Giese addition to the acrylamide, also hydrogen bonded to the catalyst, may occur. Reducing the size of the catalyst, as well as moving the point of hydrogen bonding on the substrate further away from the radical site, exposes the α-amino radical to increased rate of HAT quenching by the thiol. Finally, we sought to establish proof-of-concept that this strategy for the asymmetric functionalization of prochiral α-amino radicals could be applied beyond Giese additions. We discovered that enamide **8** could be used in place of an acrylamide to give **9**, a chiral 1,3-diamine (Scheme 3d). While the yield was low, the enantioselectivity was extremely high. Radical addition to the enamide would consitute a polarity mismatched process, and an alternative mechanism involving enamide oxidation cannot be ruled out at this stage.^24^

**Scheme 3:**
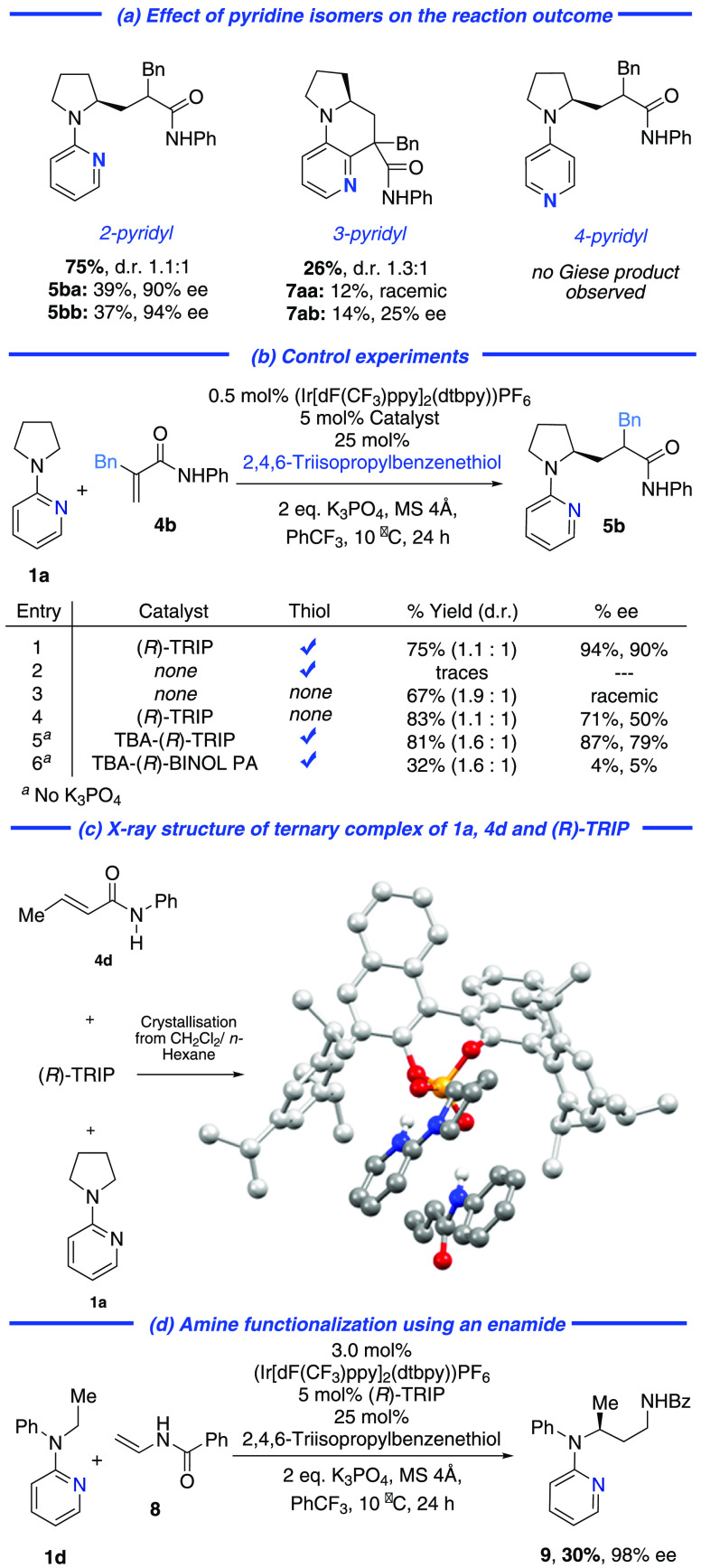
Control Experiments, Investigations to Probe Mechanism and an Enamide Acceptor

We have demonstrated a strategy for the functionalization of prochiral α-amino radicals through Giese addition by using a heteroarene as a protecting group on the amine to enable complexation with a CPA catalyst, which can also interact with the Giese acceptor. We believe that this will be useful for the synthesis of bespoke, stereochemially complex GABA analogues and that the strategy could be applicable to other α-amino radical functionalization processes.

## Supporting Information Available

Additional optimization, full experimental details, and characterization data for compounds (PDF)
