# Supplementary material for: Enantioselective Giese Additions of Prochiral α-Amino Radicals
Source: J Am Chem Soc. 2022 Dec 1;144(49):22451–7. doi: 10.1021/jacs.2c11367 (PMC9756345; doi:10.1021/jacs.2c11367)
Supplement: Supplementary file 1 — ja2c11367_si_001.pdf [file ja2c11367_si_001.pdf]

Supporting Information

Enantioselective Giese Additions of Prochiral α-Amino Radicals

Antti S. K. Lahdenperä, P. David Bacoş and Robert J. Phipps *

Table of Contents

General Information S3
Reaction optimisation S5
Less effective substrates: S11
General Procedures: S12
Copper-catalysed C-N cross-coupling S12
Buchwald-Hartwig cross-coupling: S12
General procedure for acrylamide synthesis S13
General procedure for the asymmetric Giese reaction S13
General acid workup procedure: S14
Determination of stereochemistry in products, relative and absolute S15
Stereochemistry of the asymmetric Giese products with α-substituted *N*-phenylacrylamides S15
Absolute stereochemistry of the asymmetric Giese products with β-substituted *N*-phenylacrylamides and no α-amino stereocenter S19
Relative and absolute stereochemistry of the asymmetric Giese products with β-substituted N-phenylacrylamides, also containing an α-amine stereocenter S20
Stereochemistry of the asymmetric Giese products with α,β-disubstituted *N*-phenylacrylamides S21
Synthesis of products through asymmetric Giese reaction S22
Deprotection and cyclisation of 5ba and 5bb S56
Formal synthesis of (–)-pseudoheliotridane S61
Synthesis of *ent*-pregabalin S64
Use of an enamide as the acceptor S66
Less effective substrate S67
Synthesis of Starting Materials S67
X-ray structure of complex between 2-(pyrrolidin-1-yl)pyridine **1a**, (*E*)-*N*-phenylbut-2-enamide **4d** and (*R*)-TRIP S87
Stern-Volmer quenching studies S88
Chiral SFC, HPLC and GC traces S93
NMR Spectra S139
References S350

General Information

Reagents: All reagents, unless otherwise stated, were used as supplied from commercial sources without further purification. Trifluorotoluene ($PhCF_3$) was purified by distillation on site under inert atmosphere from calcium hydride and stored over 4 Å molecular sieves under nitrogen atmosphere.

Reaction setup: Reactions were carried out in dried 4 mL, 15x45mm crimp-top vials. When cooling was required the reactions were performed in a cooled room (temperature 7-10 °C). The syntheses of (*R*)-TRIP[1], $[Ir(dF(CF_3)ppy)_2(dtbpy)]PF_6$[2] and 2,4,6-triisopropylbenzenethiol (TRIP thiol)[3] were prepared as described in the literature.

NMR spectra: ^1H NMR spectra were recorded on a 600 MHz Bruker Avance DRX-600 spectrometer, 500 MHz Bruker DCH Cryoprobe, 400 MHz Bruker QNP Cryoprobe or 400 MHz Bruker Avance NEO Prodigy N2 Cryoprobe. Chemical shifts are reported in parts per million (ppm) and the spectra are calibrated to the resonance resulting from incomplete deuteration of the solvent ($CDCl_3$: 7.26 ppm; DMSO-d_6: 2.50 ppm, qn; MeOD-d_4: 3.31 ppm, qn; D_2O: 4.79 ppm, CD_3CN: 1.96 ppm). ^{13}C NMR spectra were recorded on the same spectrometers with complete proton decoupling. Chemical shifts are reported in ppm with the solvent resonance as the internal standard ($^{13}CDCl_3$: 77.16 ppm, t; DMSO-d_6: 39.52 ppm, sept; MeOD-d_4: 49.00 ppm, sept; CD_3CN: 118.26 ppm, s; 29.92 ppm, sept). Data are reported as follows: chemical shift δ/ppm, integration (^1H only), multiplicity (s = singlet, d = doublet, t = triplet, q = quartet, qn = quintet, sext = sextet, sept = septet, br = broad, m = multiplet or combinations thereof; ^{13}C and ^{19}F signals are singlets unless otherwise stated), coupling constants *J* in Hz. ^1H-COSY, HSQC, HMBC and NOESY were used where appropriate to facilitate structural determination of regioisomers. ^{19}F spectra were recorded on a 400 MHz Bruker Avance III HD and 400 MHz Bruker Avance NEO Prodigy N2 Cryoprobe Spectrometer with complete proton decoupling.

High Resolution Mass Spectrometry (HRMS): Some were recorded on a Waters Micromass LCT Premier spectrometer using an electrospray ionization (ESI) or on a Waters Xevo G2-S bench top QTOF using an electrospray ionization (ESI) or atmospheric solids analysis probe (ASAP). Measured values are reported to 4 decimal places are within ±5 ppm of the calculated value. The calculated values are based on the most abundant isotope.

Chromatography: Analytical thin layer chromatography was performed using precoated Merck glass backed silica gel plates (Silicagel 60 F254). Visualisation was by ultraviolet fluorescence (λ = 254 or 365 nm) and/or staining with cerium ammonium molybdate (CAM), potassium permanganate ($KMnO_4$) or vanillin. Flash column chromatography was performed using silica gel 60 (0.040-0.063 µm) from Material Harvest Ltd.

Optical rotations: Measured in spectrophotometric grade $CHCl_3$ or H_2O on a Perkin Elmer 43 Polarimeter using a sodium lamp (λ = 589 nm, D-line). α_D values are reported at a given temperature (°C) with concentration in g/100mL.

Chiral HPLC analysis: Performed on a Shimadzu XR-LC system with DAICEL CHIRALPAK AD-H, IC, AS or IA columns (4.6x250 mm, 5 µm) or CHIRALCEL OD (4.6x250 mm, 5 µm) in a mixed solvent system of *n*-hexane and *i*PrOH.

Chiral SFC analysis: Performed on a Waters ACQUITY UPC2 system with YMC CHIRAL ART SB, SC or SJ, or DAICEL CHIRALPAK IE or IG columns (4.6x250 mm, 3 µm) in a mixed solvent system of supercritical CO_2 and MeOH. A system backpressure of 138 bar was used in all cases.

X-ray crystallography: Performed on a Nonius Kappa CCD diffractometer or a Bruker D8- QUEST PHOTON-100 diffractometer using CuKa radiation (λ = 1.5418 Å) at the Cambridge University Chemistry X-Ray Laboratory. Data collection and analysis was performed by Dr. Andrew Bond (University of Cambridge).

Racemic reactions: Reactions to obtain racemic SFC samples were either run using racemic TRIP catalyst or else no phosphoric acid catalyst with no thiol co-catalyst.

Preparative High Performance Liquid Chromatography (HPLC): Samples were purified by preparative high performance liquid chromatography (Waters CSH C18 OBD, 30 x 100 mm, 5 µm), using decreasingly polar mixtures of H_2O (containing 0.3% ammonium hydroxide or 0.1% formic acid) and MeCN as eluents. After chromatography, fractions containing the desired compound were dried via Biotage V10 (using "HPLC fractions" method (MeCN washes)).

Reaction optimisation

Supplementary Table 1: Solvent optimisation for the asymmetric Giese reaction between compounds **1a** and **4a**.

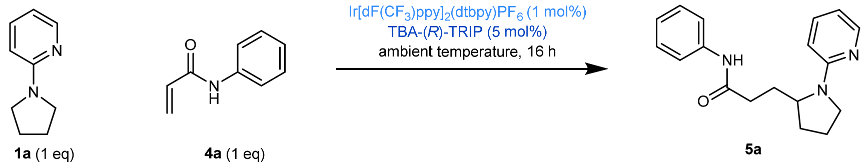

Entry	Solvent	Yield*	ee†
1	$PhCF_3$	23%	19%
2	THF	19%	8%
3	MeCN	33%	−2%
4	Et_2O	13%	2%
5	DCM	6%	30%
6	1,4-Dioxane	9%	8%
7	Benzene	17%	24%
8	MeOH	15%	2%
9	*n*-Hexane	traces	-
10	CPME	17%	14%

* Yields determined by ^{1}H NMR from the crude mixture of the reaction. † ee determined by chiral SFC assay.

Supplementary Table 2: Reaction optimisation for the asymmetric Giese reaction between compounds **1a** and **4a**.

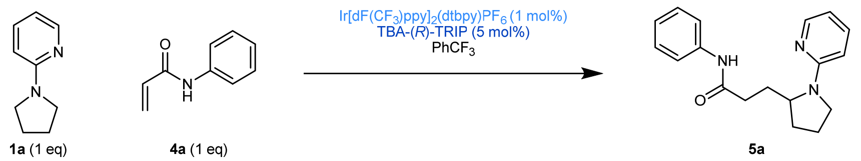

Entry	Time / h	Temp.	1a (eq)	4a (eq)	Additive	CPA Catalyst	Yield*	ee†
1	16	RT	**1**	**1**	-	TBA-(*R*)-TRIP	23%	19%
2	16	RT	**1**	**1**	**2,4,6-triisopropylbenzenethiol (0.25 eq)**	TBA-(*R*)-TRIP	24%	58%
3	16	RT	1	1	2,4,6-triisopropylbenzenethiol **(1 eq)**	TBA-(*R*)-TRIP	33%	62%
4	16	RT	**1**	**1**	**Thiophenol (0.25 eq)**	TBA-(*R*)-TRIP	20%	64%
5	**7**	RT	1	1	Thiophenol (0.25 eq)	TBA-(*R*)-TRIP	20%	56%
6	7	RT	1	1	Thiophenol (0.25 eq), **MS 4Å**	TBA-(*R*)-TRIP	19%	73%
7	7	RT	1	1	Thiophenol (0.25 eq), MS 4Å, **K_3PO_4 (2 eq)**	**(*R*)-TRIP**	16%	77%
8	7	RT	1	1	Thiophenol (0.25 eq), **MS 4Å**	**Ir-(*R*)-TRIP‡**	16%	63%
9	**20**	**−40 °C**	**2**	**1**	2,4,6-triisopropylbenzenethiol (0.25 eq), MS 4Å, K_3PO_4 (2 eq)	**(*R*)-TRIP**	30%	84%

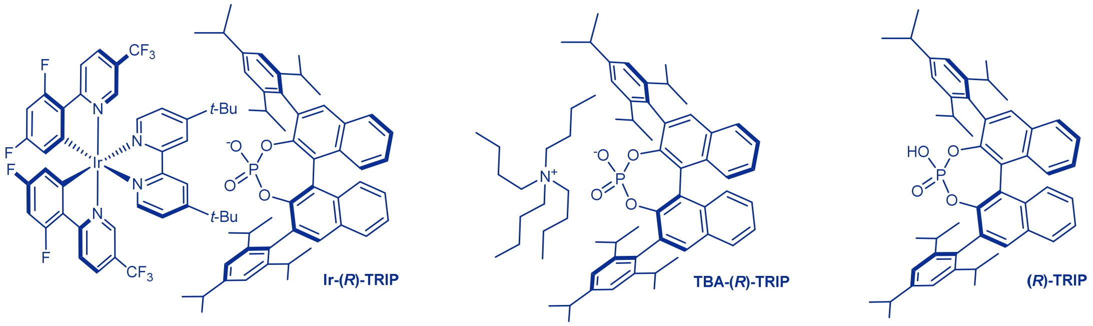

* Yields determined by ^{1}H NMR from the crude mixture of the reaction. † ee determined by chiral SFC assay. ‡ 1 mol% CPA catalyst loading.

Supplementary Table 3: CPA catalyst optimisation for the asymmetric Giese reaction of **1a** and **4b**.

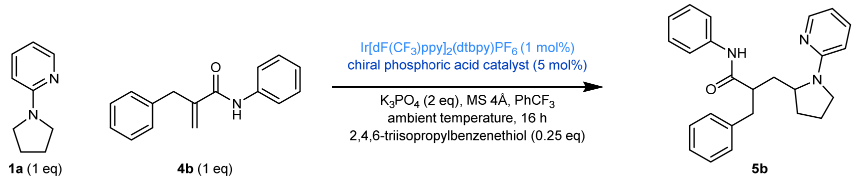

Entry	Catalyst	Yield*	d.r.*	Diastereomer 1, 5ba (ee)†	Diastereomer 2, 5bb (ee)†
1	**1**	78%	1:1.4	87%	91%
2	**2**	82%	1:1.4	74%	83%
3	**3**	70%	1:1.7	13%	10%
4	**4**	77%	1:1.8	−10%	−16%
5	**5**	78%	1:1.8	20%	13%
6	**6**	71%	1:2.3	−10%	−16%
7	**7**	77%	1:1.7	83%	89%
8	**8**	70%	1:1.3	26%	22%
9	**9**	73%	1:2.2	12%	10%

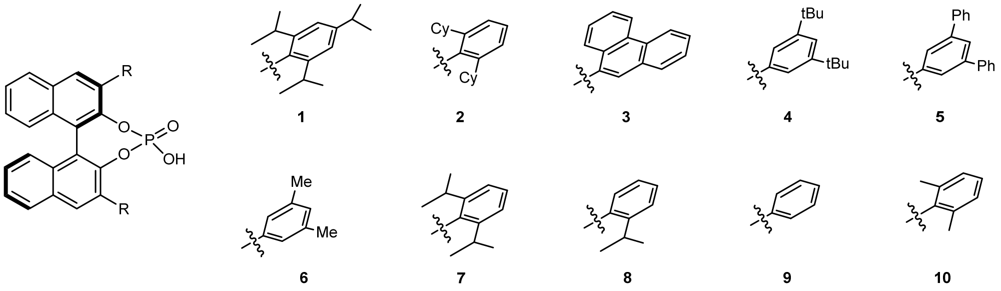

* Yields and diastereoselectivities determined by ^{1}H NMR from the crude mixture of the reaction. † ee determined by chiral SFC assay.

Supplementary Table 4: Base optimisation for the asymmetric Giese reaction of **1a** and **4b**.

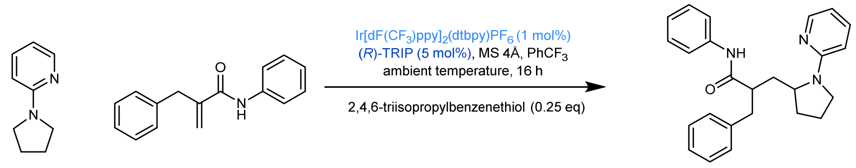

Entry	Base (2 eq)	Yield*	d.r.*	Diastereomer 1, 5ba (ee)†	Diastereomer 2, 5bb (ee)†
1	K_3PO_4	78%	1:1.4	87%	91%
2	KF	79%	1:1.7	87%	90%
3	Na_2CO_3	81%	1:1.7	81%	83%
4	Na_3PO_4	82%	1.4:1	87%	92%
5	K_2HPO_4	71%	1:1.9	82%	83%
6	Cs_2CO_3	80%	1:1.5	85%	92%
7	TEA	58%	1:2.0	35%	40%
8	Rb_2CO_3	66%	1:1.4	84%	87%
9	KOAc	79%	1:2.0	85%	89%
10	K_2CO_3	76%	1:2.0	85%	89%
11	No base	64%	1:1.8	54%	57%

* Combined yields of the diastereomers and diastereoselectivities were determined by ^{1}H NMR from the crude mixture of the reaction. † ee determined by chiral SFC assay.

Supplementary Table 5: Thiol optimisation for the asymmetric Giese reaction of **1a** and **4b**.

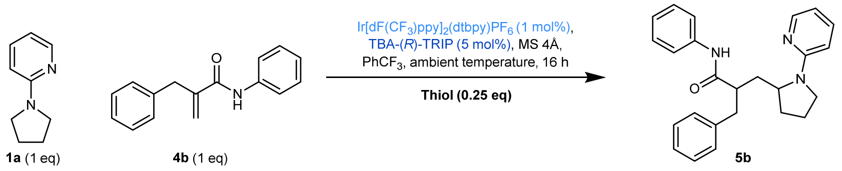

Entry	Thiol (25 mol%)	Yield*	d.r.*	Diast. 1 5ba, (ee)†	Diast. 2 5bb, (ee)†
1	Thiophenol	62%	1:1.3	70%	78%
2	2,4,6-triisopropylbenzenethiol	64%	1:2.0	73%	86%
3	2-mercaptopyridine	51%	1:1.5	34%	61%
4	Ph_3MeSH	66%	1:1.3	42%	72%
5	Me_3MeSH	72%	1:1.1	54%	72%
6	EtSH	73%	1:1.3	62%	78%
7	Ethyl thioglycolate	67%	1.6:1	2%	59%
8	Cyclohexanethiol	61%	1:1	50%	71%
9	Thiobenzoic acid	56%	1:1	10%	21%
10‡	L-cysteine methyl ester hydrochloride	51%	1:1.2	5%	12%
11	*N*-(*tert*-Butoxycarbonyl)-L-cysteine methyl ester	66%	1:1.3	78%	88%

* Combined yields of the diastereomers and diastereoselectivities were determined by ^{1}H NMR from the crude mixture of the reaction. † ee determined by chiral SFC assay. ‡ 3 eq of K_3PO_4.

Supplementary Table 6: Reaction optimisation for the asymmetric Giese reaction between **1a** and **4b**.

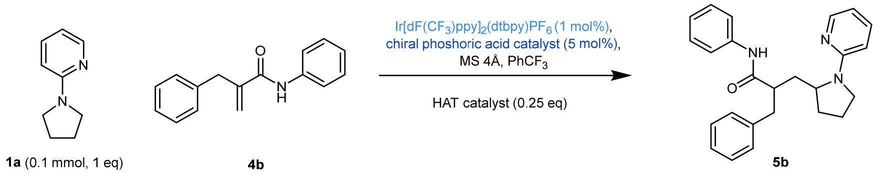

Entry	Time / h	Photocatalyst Loading	Base*	T / ˚C	CPA Catalyst	HAT catalyst	Eq (4b)	Yield **	d.r. **	Diast. 1 5ba (ee)†	Diast. 2 5bb (ee)†
1	16	1 mol%	-	25-30	TBA-(*R*)-TRIP	Thiophenol	1	62%	1:1.3	70%	78%
2	16	1 mol%	-	25-30	TBA-(*R*)-TRIP	**TRIP thiol**	1	64%	1:2.0	73%	86%
3	16	1 mol%	K_3PO_4	25-30	**(*R*)-TRIP**	TRIP thiol	1	78%	1:1.4	87%	91%
4	16	1 mol%	-	25-30	**(*R*)-TRIP**	TRIP thiol	1	64%	1:1.8	54%	57%
5	16	1 mol%	K_3PO_4	**10**	(*R*)-TRIP	TRIP thiol	1	74%	1:1.3	88%	93%
6	7	1 mol%	K_3PO_4	10	(*R*)-TRIP	TRIP thiol	**1.5**	83%	1:1.2	90%	93%
7‡	8	**0.5 mol%**	K_3PO_4	10	(*R*)-TRIP	TRIP thiol	1.5	75%	1:1.1	90%	94%
8‡	8	0.5 mol%	K_3PO_4	10	**(*S*)-TRIP**	TRIP thiol	1.5	74%	1.1:1	−90%	−92%
9‡	8	0.5 mol%	K_3PO_4	10	**(*rac*)-TRIP**	TRIP thiol	1.5	75%	1.2:1	rac	rac
10≠	14	0.5 mol%	K_3PO_4	10	(*R*)-TRIP	TRIP thiol	1.5	72%	1:1.6	88%	96%

* Examples including 2 eq of base.** Combined yields of the diastereomers and diastereoselectivities were determined by ^{1}H NMR from the crude mixture of the reaction. † ee determined by chiral SFC assay. ‡ 0.2 mmol scale, isolated yields. ≠ 4 mmol scale, isolated yield.

Supplementary Table 7: Reaction optimisation for the asymmetric Giese reaction between compounds **1c** and **4c**.

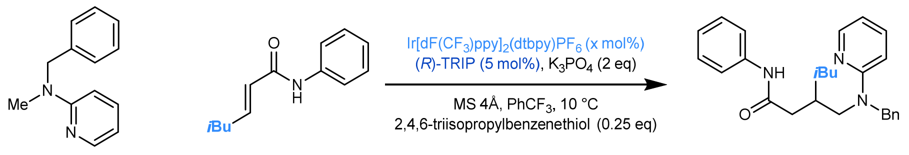

1c (1 eq) **4c** (1.5 eq) **6p**

Entry	Photocatalyst Loading	Time / h	Yield*	ee†
1	3 mol%	48	16%	No data
2	5 mol%	24	29%	99%
3#	5 mol%	24	30%	99%
4	10 mol%	24	40%‡	99%

* Yields determined by ^{1}H NMR from the crude mixture of the reaction. † ee determined by chiral SFC assay.
# Reaction performed in DCM ‡ Isolated yield.

Less effective substrates:

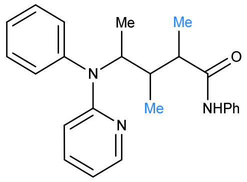

<11% *

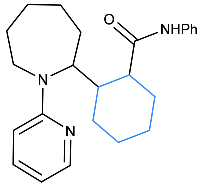

<20% *

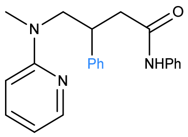

52% isolated yield
(37% ee)

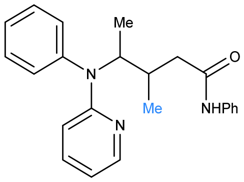

<10% *

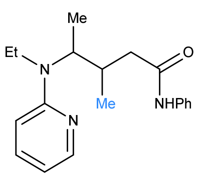

<5% *

* Yields determined by ^{1}H NMR from the crude mixture of the reaction.

General Procedures:

Copper-catalysed C-N cross-coupling

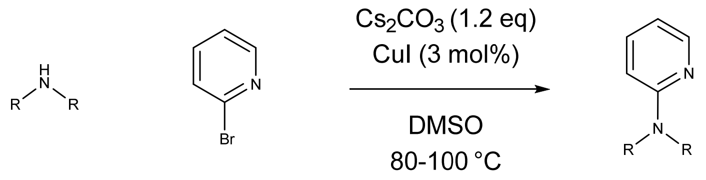

Scheme 1. Procedure for copper-catalysed C-N cross-coupling.

Secondary amine (2 eq), 2-bromopyridine (1 eq), Cs_2CO_3 (1.2 eq), CuI (3 mol%) and DMSO (5 mL) were added to a microwave vial. The vial was evacuated and refilled with nitrogen three times, sealed and heated between 80 and 100 °C overnight. The reaction mixture was cooled to ambient temperature and the crude was purified by silica gel column chromatography to afford the desired product.

Buchwald-Hartwig cross-coupling:

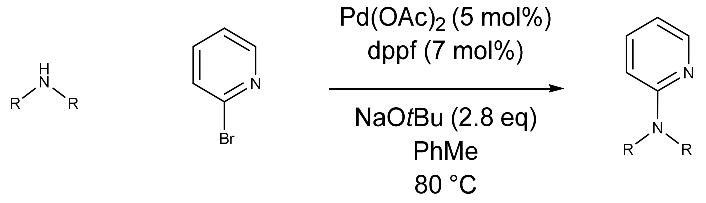

Scheme 2. Procedure for Buchwald-Hartwig cross-coupling.

Under a dry, inert atmosphere, sodium *tert*-butoxide (2.8 eq), DPPF (7 mol%), palladium(II) acetate (5 mol%), amine (1.1-2.0 eq), and 2-bromopyridine (1.0 eq) were added to a reaction vial. The vial was evacuated and refilled with nitrogen three times. Anhydrous toluene (5-10 mL) was added and the reaction was stirred overnight at 80 °C. The reaction mixture was cooled to room temperature and filtered through celite. The mixture was then concentrated *in vacuo* and purified by silica gel column chromatography to afford the desired products.

General procedure for acrylamide synthesis

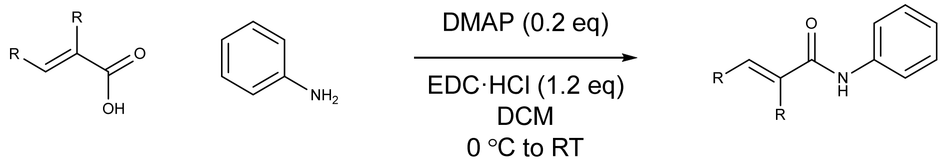

Scheme 3. General procedure for acrylamide synthesis.

Under a dry, inert atmosphere, to a cooled mixture (0 °C) of carboxylic acid (1.1 eq), aniline (1.0 eq), and DMAP (0.20 eq) in anhydrous dichloromethane (100 mL) was added EDC·HCl (1.2 eq) in portions. The mixture was stirred overnight at room temperature, after which it was concentrated under reduced pressure and purified by silica gel column chromatography. If required, the purified product was triturated with diethyl ether to remove minor impurities.

General procedure for the asymmetric Giese reaction

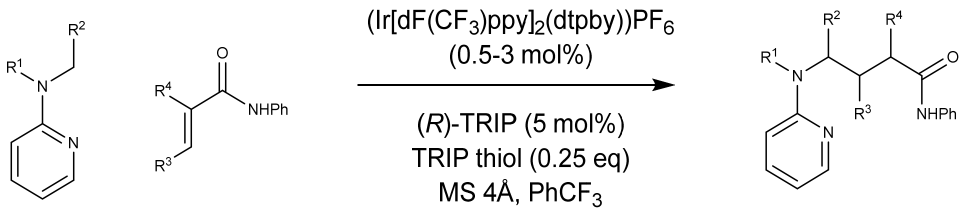

Scheme 4. General procedure for the asymmetric Giese reaction.

The acrylamide (0.30 mmol, 1.5 eq), chiral phosphoric acid (0.010 mmol, 5 mol%), powdered dried 4Å molecular sieves (30 mg), (Ir[dF(CF_3)ppy]$_2$(dtpby))PF_6 (0.5 to 3 mol%) and K_3PO_4 (84.9 mg, 0.40 mmol, 2 eq) were measured into a 4.0 mL, oven-dried crimp-top vial. The vial was sealed, evacuated and backfilled with nitrogen three times. Freshly prepared solutions of the amine (1.0 mL, 0.20 mmol, 0.2 M in anhydrous $PhCF_3$, 1 eq) and the 2,4,6-triisopropylbenzenethiol (1.0 mL, 0.050 mmol, 0.25 M in anhydrous $PhCF_3$, 0.25 eq) were added *via* syringe to the sealed vial. The reaction vessel was evacuated and backfilled with nitrogen, placed in a cooled room (temperature 5-7 °C, reaction temperature 10 °C) and irradiated with Kessil Tuna blue lamp (100% intensity, 33% ocean blue colour). The crude reaction mixture was filtered through small plug of silica and the solvents were removed *in vacuo.* The diastereoselectivity was measured from the crude mixture by ^{1}H NMR, which was purified without further workup by column chromatography.

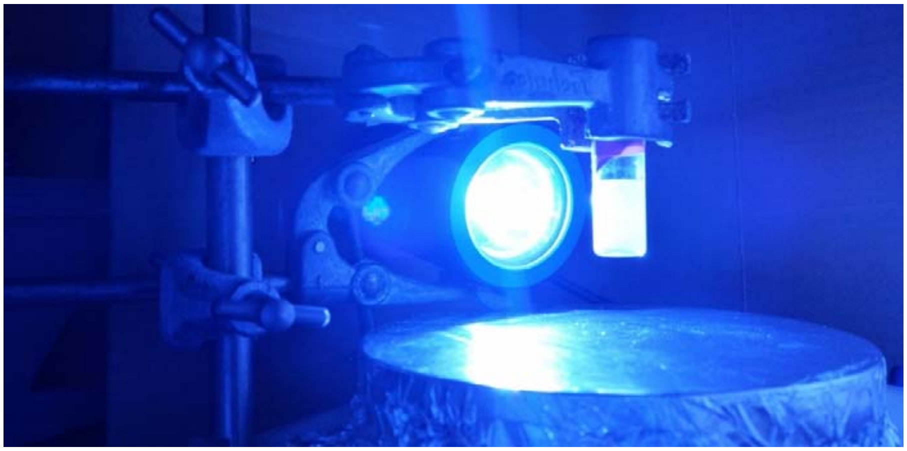

Figure 1: Photograph of the typical reaction setup.

General acid workup procedure:

When the acrylamide showed challenging separation form the products the crude mixture was acidified by 1 M HCl (20 mL). The additional acrylamide was removed by extracting the acidified aqueous mixture with EtOAc (20-40 mL). The protonated product in the aqueous mixture was neutralised by addition of $NaHCO_3$ or Na_2CO_3 and the aqueous mixture was extracted with DCM (40-200 mL). The combined organic phases were dried over $MgSO_4$, filtered, concentrated *in vacuo* followed by silica gel chromatography purification.

Alternatively, when the purification was performed without the workup and the desired purified product still contained acrylamide, the material could be further subjected to the general acid workup procedure.

Determination of stereochemistry in products, relative and absolute

Stereochemistry of the asymmetric Giese products with α-substituted *N*-phenylacrylamides

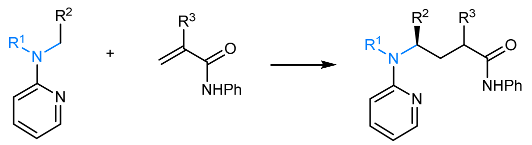

Scheme 5. Asymmetric Giese reaction with α-substituted N-phenylacrylamides.

In most instances using α-substituted acrylamides no control over the stereochemistry formed at the position α to the amide was observed and mixtures of diastereomers were obtained. These two diastereomers exhibited distinctive and consistent behavior on silica gel and had consistent and distinctive differences in the ^{1}H-NMR spectra.

Diastereomer 1 refers to the faster running diastereomer on silica, diastereomer 2 to the slower running.

In diastereomer 1 the chemical shift of the NH proton in the ^{1}H-NMR spectrum exhibited a markedly downfield shift (higher ppm) compared with that in diastereomer 2. This suggested a greater extent of intramolecular hydrogen bonding in diastereomer 1.

Absolute and relative stereochemistry: Determined by single crystal X-ray analysis of the hydrochloride salt of Diastereomer 1 for the below compound: (*S*)-*N*-phenyl-2-(((*S*)-1-(pyridin-2-yl)pyrrolidin-2-yl)methyl)butanamide (**5ea**). Deposited in the CCDC with deposition number 2183882. This revealed diastereomer 1 in this case to be the 'anti' compound.

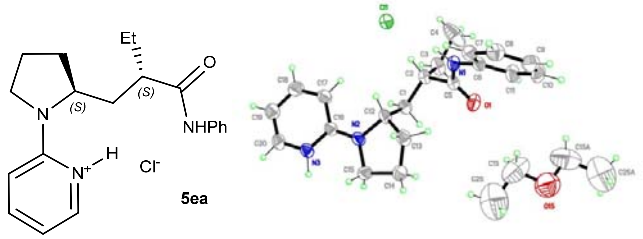

Figure 2. X-ray crystal structure of protonated 5ea.

The assignment of the absolute stereochemistry at the α-amine stereocenter was extended to the other substrates in analogy with the above example. This includes substrates where the amine is acyclic (Manuscript, Scheme 1b) and also includes the tentative assignment of product **9**, originating from the enantioselective reaction of **1d** with enamide **8** (Manuscript, Scheme 3d).

Relative stereochemistry confirmation (5ba): Determined by single crystal X-ray analysis of crystals from a sample of the hydrochloride salt of enantioenriched diastereomer 1 of the below compound: (*S*)-2-benzyl-*N*-phenyl-3-((*S*)-1-(pyridin-2-yl)pyrrolidin-2-yl)propanamide **5ba**. However, the crystals obtained were of a racemic mixture of *R* and *S* so did not allow absolute stereochemical determination but allowed confirmation of relative stereochemistry. Deposited in the CCDC with deposition number 2183885. This revealed diastereomer 1 in this case also to be the 'anti' compound.

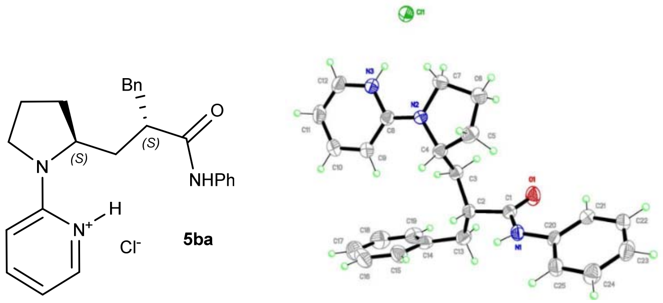

Figure 3. X-ray crystal structure of protonated 5ba.

The different characteristics of the two diastereomers can be rationalized by considering the propensity of each to form intramolecular hydrogen bonds in solution:

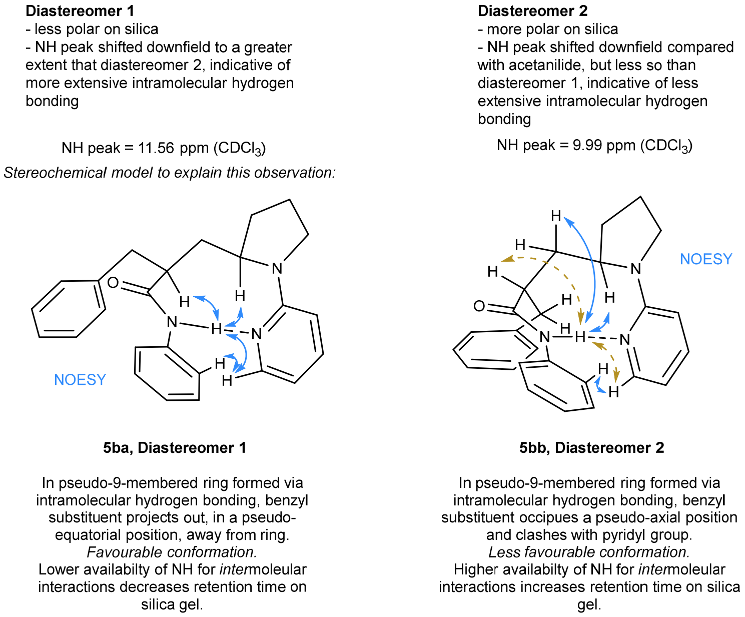

Figure 4. Comparison of the intramolecular hydrogen bonging of diastereomers 5ba and 5bb.

Using the above rationalization, relative stereochemistry assignment was extended to the other products by tentative analogy through comparison of the ^{1}H NMR spectra and retention times on silica between the two diastereomers.

Additionally, we sought to convert each diastereomer of enantioenriched 5ba (diasteromer 1) and 5bb (diasteromer 2) through to the corresponding lactams through a deprotection/cyclization procedure both to demonstrate deprotection and also to compare the resulting lactams **5be** and **5bf** with literature data relating to relative stereochemistry.

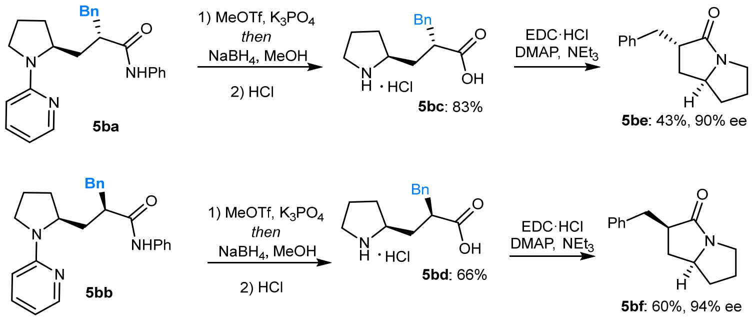

Scheme 6. Conversion of 5ba and 5bb to the corresponding lactams 5be and 5bf for the determination of the relative stereochemistry.

Compound **5be** has been reported to have been synthesised by Jin and co-workers from L-proline through a multistep sequence involving an enolate benzylation to introduce the second stereocenter.[4] This was highly diastereoselective and after conversion to the lactam the diastereomer was assigned as being the *anti* diastereomer **5be** using NOESY experiments. However, we found that the ^{1}H and ^{13}C NMR data in that paper matched our *syn* diastereomer **5bf** rather than the expected **5be.** A subsequent report from Frost and co-workers obtained both diasteromers **5be** and **5bf** and assigned the relative stereochemistry based on referral back to the earlier report from Jin and co-workers.[5] We have carried out NOESY experiments on both **5be** and **5bf**. The analysis of **5bf** is challenging due to the overlapping of critical peaks, which could understandably have led to possible misassignment. NOESY analysis of **5bf** does show a critical NOE between *syn* 1,3-protons on the lactam ring which are strongly suggestive of the *syn* stereochemistry shown above (H4 and H6 in NOESY spectrum below). This together with our confirmation of the relative stereochemistry of precursor **5ba** by X-ray crystallography leads us to believe that it is likely that the relative stereochemistry of these diastereomers has previously been misassigned in the preceding two reports.

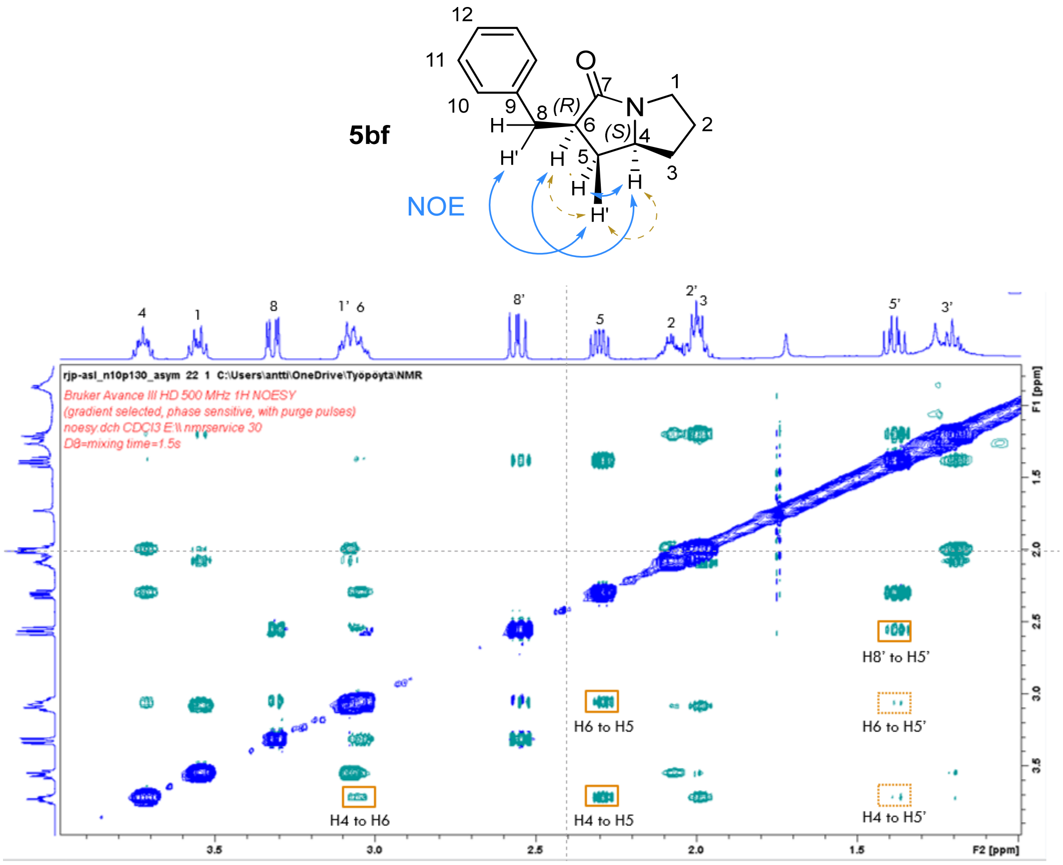

Figure 5. NOESY correlations and spectrum of 5bf.

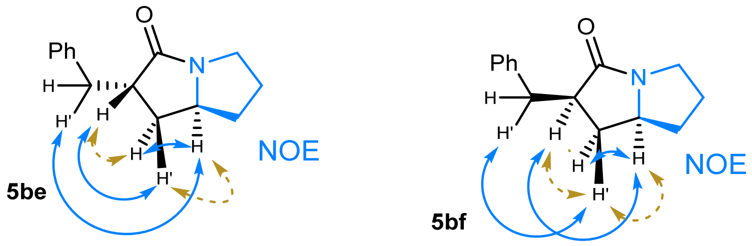

Figure 6. Summary of NOE interactions (full spectra provided later in SI):

Absolute stereochemistry of the asymmetric Giese products with β-substituted *N*-phenylacrylamides and no α-amino stereocenter

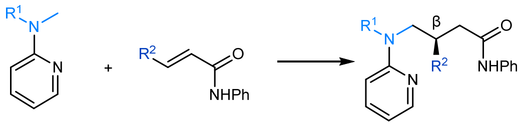

Scheme 7. Asymmetric Giese reaction with β-substituted N-phenylacrylamides.

The absolute stereochemistry of β-substituted products of the asymmetric Giese reaction was assigned by analogy to the (*R*)-3-((benzyl(pyridin-2-yl)amino)methyl)-5-methyl-*N*-phenylhexanamide **6p**, which was after conversion to hydrochloride salt of (*R*)-pregabalin compared to literature optical rotation values. The hydrochloride salt of (*R*)-pregabalin was further in analytical scale converted to a (*R*)-4-isobutylpyrrolidin-2-one **6pa** and compared by SFC analysis to (*S*)-4-isobutylpyrrolidin-2-one **6pb** prepared from commercially obtained (*S*)-pregabalin and racemic 4-isobutylpyrrolidin-2-one **6pb** prepared according to a literature procedure.[6] or comparison of opical rotation values of (*R*)-4-isobutylpyrrolidin-2-one, material was obtained by deprotection of (*R*)-3-((benzyl(pyridin-2-yl)amino)methyl)-5-methyl-*N*-phenylhexanamide **6a** to its' free amine **6pc** which was observed to cyclise over time to (*R*)-4-isobutylpyrrolidin-2-one in deuterated chloroform.

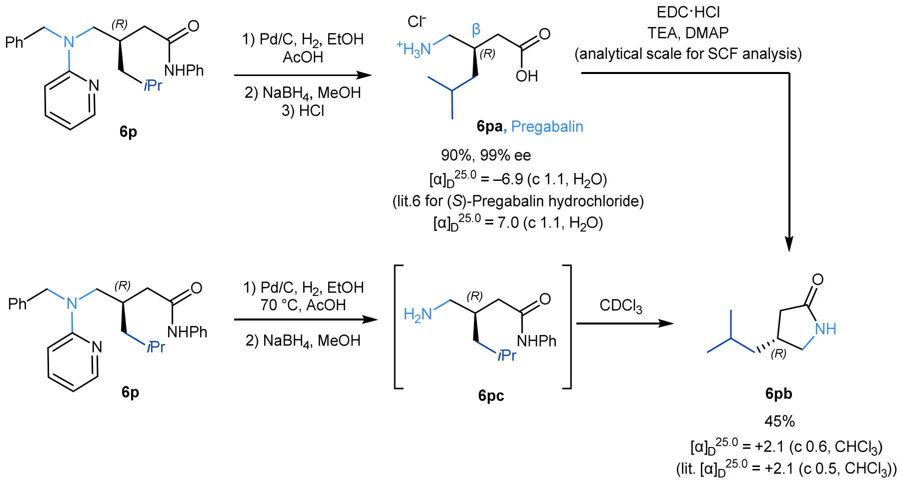

Scheme 8. Conversion of 6p to (R)-pregabalin and to lactam 6pb for the determination of the absolut stereochemistry.

Relative and absolute stereochemistry of the asymmetric Giese products with β-substituted N-phenylacrylamides, also containing an α-amine stereocenter

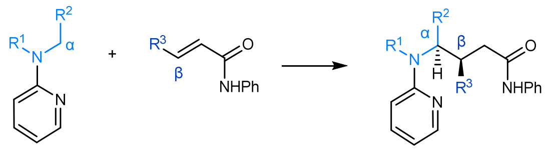

Scheme 9. Asymmetric Giese reaction forming α-amine and β-carbonyl stereocenter.

The absolute and relative stereochemistry of β-substituted products of asymmetric Giese reactions that also produce an α-amine stereocenter was assigned by analogy to (*R*)-*N*-phenyl-3-((*S*)-1-(pyridin-2-yl)pyrrolidin-2-yl)butanamide **6q** which was converted to (1*R*,7a*S*)-1-methylhexahydro-3*H*-pyrrolizin-3-one **6qb**, a known precursor to (–)-pseudoheliotridane. The NMR and optical rotation data for this compound was compared to literature data to confirm the stereochemistry shown. Additional NOESY studies were conducted on **6qc**. These studies, summarized here, are detailed later in the relevant section of the supporting information. The absolute stereochemistry of the α-amino stereocenter was found to be (*S*) when (*R*)-TRIP was used as catalyst, consistent with the outcome using α-substituted Giese acceptors (see above).

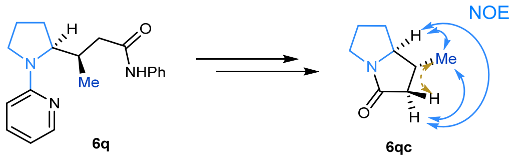

Scheme 10. NOESY correlations of 6qc for the determination of the relative stereochemistry.

Stereochemistry of the asymmetric Giese products with α,β-disubstituted *N*-phenylacrylamides

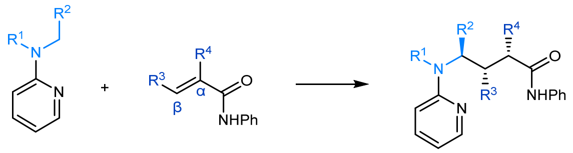

Scheme 11. Asymmetric Giese reacion forming α-amine, β- and α-carbonyl stereocenters.

The relative stereochemistry of the products from the asymmetric Giese reactions with α,β-disubstituted *N*-phenylacrylamides was determined by single crystal X-ray analysis of the hydrochloride salt of (*2S,3S*)-2-methyl-*N*-phenyl-3-((*S*)-1-(pyridin-2-yl)pyrrolidin-2-yl)butanamide **6n**. In this case the absolute stereochemistry could not be definitively determined by crystallogprahic analysis but the relative stereochemistry could. Deposited in the CCDC with deposition number 2183884.

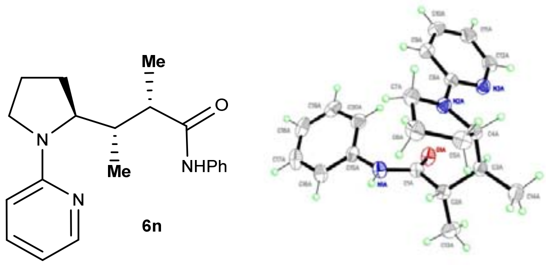

Figure 7. X-ray crystal structure of 6n.

By analogy, the relative stereochemistry of related products was tentatively assigned. It is interesting to note that in the major diastereomer the relative stereochemistry of the α-amino stereocenter and the stereocenter arising from the β-position of the acceptor is opposite to that obtained when the acceptor possesses only β-substitution (see earlier section). This could feasibly arise due to a flipping of the acceptor in the transition state when an α-substituent is present on the acceptor.

For assignment of absolute stereochemistry, we have tentatively assigned this as being (*S*) at the α-amino stereocenter when (*R*)-TRIP is used as catalyst. This is an analogy with the outcome on pyrrolidine-based substrates using α-substituted Giese acceptors as well as β-substituted acceptors (see above sections).

Synthesis of products through asymmetric Giese reaction

(*S*)-*N*-Phenyl-3-(1-(pyridin-2-yl)pyrrolidin-2-yl)propanamide (5a)

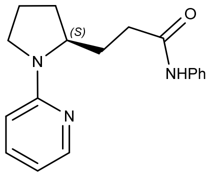

5a

Following the general procedure for the asymmetric Giese reaction using 2-(pyrrolidin-1-yl)pyridine **1a** (1.0 mL, 0.25 mmol, 0.25 M in anhydrous $PhCF_3$, 1.25 eq), *N*-phenylacrylamide (29 mg, 0.20 mmol, 1 eq) and (Ir[dF(CF_3)ppy]$_2$(dtpby))PF_6 (6.6 mg, 6.0 µmol, 3 mol%) for 20 h at –40 °C. (cryocooler). The crude mixture was purified by column chromatography (eluting with 30% to 35 EtOAc in in petroleum ether to yield the title compound **5a** (18 mg, 0.060 mmol, 30% yield, 84% ee) as a colourless film.

^{1}H NMR (400 MHz, $CDCl_3$) δ 10.83 (br. s, 1H), 8.07 (ddd, *J* = 5.2, 1.7, 0.5 Hz, 1H), 7.65 (dd, *J* = 8.5, 0.9 Hz, 2H), 7.47 (ddd, *J* = 8.5, 7.2, 1.4 Hz, 1H), 7.32 (t, *J* = 7.8 Hz, 2H), 7.10 (td, *J* = 7.3, 0.9 Hz, 1H), 6.56 (dd, *J* = 6.6, 5.6 Hz, 1H), 6.43 (d, *J* = 8.7 Hz, 1H), 4.45 (q, *J* = 6.5 Hz, 1H), 3.49 (ddd, *J* = 9.5, 8.4, 1.3 Hz, 1H), 3.24 (td, *J* = 9.6, 7.3 Hz, 1H), 2.60 (ddd, *J* = 15.2, 8.9, 3.5 Hz, 1H), 2.37 (ddd, *J* = 15.2, 9.6, 3.4 Hz, 1H), 2.18 (dddd, *J* = 14.2, 9.8, 6.4, 3.6 Hz, 1H), 2.14 – 2.07 (m, 1H), 2.07 – 2.01 (m, 1H), 2.01 – 1.93 (m, 1H), 1.79 (dd, *J* = 11.7, 6.8 Hz, 1H), 1.65 (dddd, *J* = 14.5, 8.7, 6.6, 3.3 Hz, 1H). **^{13}C NMR** (126 MHz, $CDCl_3$) δ 172.0, 157.4, 147.1, 139.1, 137.8, 128.9, 124.0, 121.0, 111.8, 107.3, 56.6, 47.8, 34.6, 31.0, 30.5, 23.6. **HRMS** m/z: $[M + H]^+$ calc'd for $[C_{18}H_{22}N_3O]^+$ expect 296.1757; found 296.1756. **$[\alpha]_D^{25.0}$** = +157.8 (*c* 1.0, $CHCl_3$).

(*S*)-2-Benzyl-*N*-phenyl-3-((*S*)-1-(pyridin-2-yl)pyrrolidin-2-yl)propanamide (5ba) and (*R*)-2-benzyl-*N*-phenyl-3-((*S*)-1-(pyridin-2-yl)pyrrolidin-2-yl)propanamide (5bb)

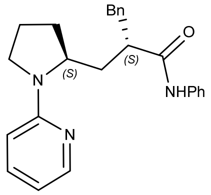

5ba

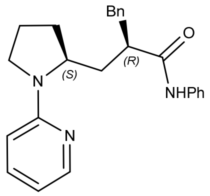

5bb

Following the general procedure for the asymmetric Giese reaction using 2-(pyrrolidin-1-yl)pyridine **1a** (1.0 mL, 0.20 mmol, 0.2 M in anhydrous $PhCF_3$, 1 eq), 2-benzyl-*N*-phenylacrylamide **4b** (71 mg, 0.30 mmol, 1.5 eq) and (Ir[dF(CF_3)ppy]$_2$(dtpby))PF_6 (1.1 mg, 1.0 µmol, 0.5 mol%) for 8 h. The diastereoselectivity was determined from the crude mixture (1:1.1 d.r. (**5ba**:**5bb**) ^{1}H NMR) and the crude was purified without further workup by column chromatography (eluting with 15% EtOAc in 0.5% TEA in petroleum ether) to yield 5ba as the first isolated diastereomer (30 mg, 0.078 mmol, 39% yield, 90% ee) and the **5bb** as the second diastereomer (29 mg, 0.074 mmol, 37% yield, 94% ee) both as white solids. Combined yield: 58 mg, 0.15 mmol, 75%.

Diastereomer 1 (**5ba**):

^{1}**H NMR** (400 MHz, $CDCl_3$) δ 11.56 (br. s, 1H), 8.16 (d, *J* = 4.9 Hz, 1H), 7.70 (d, *J* = 8.1 Hz, 1H), 7.53 (t, *J* = 7.8 Hz, 1H), 7.33 (t, *J* = 7.7 Hz, 2H), 7.20 (t, *J* = 7.3 Hz, 2H), 7.14 (d, *J* = 6.8 Hz, 1H), 7.12-7.04 (m, 3H), 4.72 (app q, *J* = 6.9 Hz, 1H), 3.24-3.07 (m, 3H), 2.99-2.85 (m, 1H), 2.64 (dd, *J* = 14.1, 7.9 Hz, 1H), 2.05-1.81 (m, 3H), 1.75 (dd, *J* = 7.8, 5.4 Hz, 2H), 1.69-1.61 (m, 1H). ^{13}**C NMR** (101 MHz, $CDCl_3$) δ 174.6, 158.3, 146.6, 140.5, 139.8, 138.5, 129.3, 129.2, 128.5, 126.2, 123.7, 120.0, 112.4, 107.4, 56.1, 48.2, 44.4, 40.3, 38.1, 32.5, 23.7. **HRMS** m/z: $[M + H]^+$ calc'd for $[C_{25}H_{28}N_3O]^+$ expect 386.2227; found 386.2233. $[\alpha]_D^{25.0}$ = +203.7 (*c* 1.0, $CHCl_3$).

X-Ray Crystallography Sample preparation

The purified sample obtained from above was suspended in HCl in 1,4-dioxane (0.2 mL, 4 M) and the solvent was removed *in vacuo*. The resultant salt was then crystallized via vapour diffusion (Et_2O / DCM) to yield colourless crystals which were analysed by x-ray diffraction. The enantioenriched material crystallised as mixture of *R*,*R* and *S*,*S* enantiomers in equal proportions. The structure was deposited in the Cambridge Crystallographic Data Centre (deposition no.: CCDC 2183885). The X-ray crystallogaphig data contains A and B level alerts. Explanations for the alerts are shown below:

Alert level A:

Author Response: instrument problems arose part-way through the data collection. The missing 8% of data are distributed quite evenly through reciprocal space (see intensity statistics in _reflns_special_details) and the refinement is very good. There is no doubt about the structure.

Alert level B:

Author Response: this is a standard warning for a KappaCCD instrument, related to obscuring of the lowest resolution reflections by the beam stop.

Diastereomer 2 (**5bb**):

^{1}**H NMR** (400 MHz, $CDCl_3$) δ 9.99 (br. s, 1H), 8.00 (d, *J* = 4.9 Hz, 1H), 7.54 (d, *J* = 8.2 Hz, 2H), 7.46 (t, *J* = 7.7 Hz, 1H), 7.35 (t, *J* = 7.6 Hz, 2H), 7.30 (t, *J* = 6.8 Hz, 2H), 7.25-7.19 (m, 3H), 7.16 (t, *J* = 7.3 Hz, 1H), 6.52 (t, *J* = 6.1 Hz, 1H), 6.41 (d, *J* = 8.5 Hz, 1H), 4.30 (app t, *J* = 8.7 Hz, 1H), 3.42 (t, *J* = 8.6 Hz, 1H), 3.32 (dd, *J* = 13.3, 4.8 Hz, 1H), 3.23 (q, *J* = 8.3 Hz, 1H), 2.87 (dd, *J* = 13.1, 9.1 Hz, 1H), 2.77-2.67 (m, 1H), 2.36 (t, *J* = 13.2 Hz, 1H), 2.07-1.81 (m, 3H), 1.68-1.57 (m, 1H), 1.44-1.26 (m, 1H). ^{13}**C NMR** (101 MHz, $CDCl_3$) δ 173.5, 157.1, 148.0, 139.9, 138.9, 137.8, 129.5, 129.0, 128.7, 126.6, 124.9, 122.9, 111.7, 107.7, 56.6, 47.6,47.3, 40.9, 32.9, 30.4, 23.7. **HRMS** m/z: $[M + H]^+$ calc'd for $[C_{25}H_{28}N_3O]^+$ expect 386.2227; found 386.2232. $[\alpha]_D^{25.0}$ = +17.9 (*c* 1.0, $CHCl_3$).

Scale-up:

$(Ir[dF(CF_3)ppy](dtbpy))PF_6$ (22.5 mg, 0.02 mmol, 0.5 mol%), 4 Å molecular sieves (600 mg), K_3PO_4 (1.7 g, 8 mmol, 2 eq), 2-benzyl-*N*-phenylacrylamide **4b** (949 mg, 4 mmol, 1 eq) and (*R*)-TRIP (151 mg, 0.20 mmol, 5 mol%) were measured in a 50 mL conical flask. The flask was sealed with rubber suba-seal and carefully evacuated and backfilled with nitrogen six times avoiding full vacuum due the container shape. A freshly prepared stock solution of 2-(Pyrrolidin-1-yl)pyridine **1a** (593 mg, 4 mmol, 5 mL, 0.8 M in anhydrous $PhCF_3$, 1 eq), 2,4,6-triisopropylbenzenethiol (236 mg, 1 mmol, 5 mL, 0.2 M in anhydrous $PhCF_3$, 0.25 eq) and further anhydrous $PhCF_3$ (30 mL) was added to the sealed flask. As

before the flask was evacuated and backfilled with nitrogen and placed in a cooled room (temperature 5-7 °C). The mixture was irradiated with Kessil Tuna blue lamp (100% intensity, 33% ocean blue colour) for 14 h. The diastereoselectivity was determined from the crude mixture (1:1.6 d.r. (**5ba:5bb**) ^{1}H NMR) and the crude was purified without further workup by column chromatography (eluting with 15% EtOAc to 25% EtOAc in 0.5 % of TEA in petroleum ether) to yield the **5ab** as the first isolated diastereomer (515 mg, 1.34 mmol, 33% yield, 88% ee) and the second diastereomer **5bb** (590 mg, 1.53 mmol, 38% yield, 96% ee) both as white solids. Combined yield: 1.11 g, 2.87 mmol, 72%.

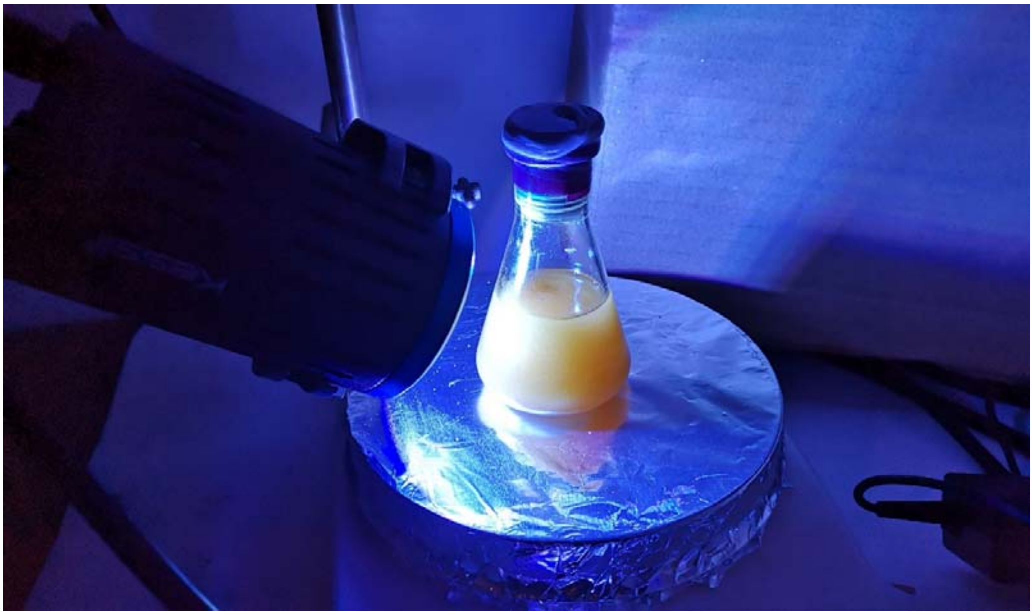

Figure 8: Reaction setup for scale-up procedure.

(*R*)-3-Methyl-*N*-phenyl-2-(((*S*)-1-(pyridin-2-yl)pyrrolidin-2-yl)methyl)butanamide (5ca) and (*S*)-3-methyl-*N*-phenyl-2-(((*S*)-1-(pyridin-2-yl)pyrrolidin-2-yl)methyl)butanamide (5cb)

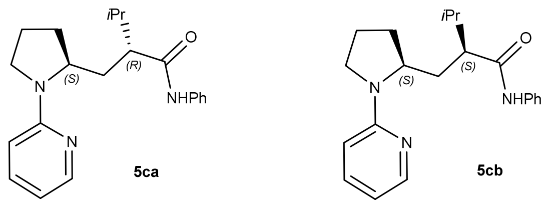

Following the general procedure for the asymmetric Giese reaction using 2-(pyrrolidin-1-yl)pyridine **1a** (1.0 mL, 0.20 mmol, 0.2 M in anhydrous $PhCF_3$, 1 eq), 3-methyl-2-methylene-*N*-phenylbutanamide **4g** (57 mg, 0.30 mmol, 1.5 eq) and $(Ir[dF(CF_3)ppy](dtbpy))PF_6$ (1.1 mg, 1.0 µmol, 0.5 mol%) for 8 h. The diastereomeric ratio was determined from the crude mixture (1:3.6 d.r. (**5ca**:**5cb**) (^{1}H NMR)) and was purified after general acid workup procedure by column chromatography (eluting with 5% to 30% EtOAc in 0.5% TEA in petroleum ether) to yield **5ca** as the first eluting diastereomer (8.0 mg, 0.024 mmol, 12% yield, 84% ee) and **5cb** the second eluting diastereomer (37 mg, 0.11 mmol, 54% yield, 93% ee) both as colourless films. Combined yield: 44 mg, 0.13 mmol, 67%.

Diastereomer 1 (**5ca**):

^{1}H NMR (400 MHz, $CDCl_3$) δ 11.05 (br. s, 1H), 8.19 (d, *J* = 4.8 Hz, 1H), 7.71 (d, *J* = 8.0 Hz, 2H), 7.53 (t, *J* = 7.8 Hz, 1H), 7.32 (t, *J* = 7.7 Hz, 2H), 7.07 (t, *J* = 7.4 Hz, 1H), 6.64 (t, *J* = 6.1 Hz, 1H), 6.45 (d, *J* = 8.6 Hz, 1H), 4.66 (t, *J* = 7.6 Hz, 1H), 3.51 (t, *J* = 9.0 Hz, 1H), 3.23 (q, *J* = 9.1 Hz, 1H), 2.38 – 2.25 (m, 1H), 2.23 – 2.12 (m, 1H), 2.10 – 1.99 (m, 1H), 1.98 – 1.81 (m, 3H), 1.75 (dd, *J* = 12.1, 6.5 Hz, 1H), 1.67 (ddd, *J* = 13.9, 11.5, 2.5 Hz, 1H). **^{13}C NMR** (101 MHz, $CDCl_3$) δ 175.3, 157.7, 145.9, 139.5, 138.5, 129.0, 123.6, 120.0, 112.1, 107.7, 56.5, 51.3, 48.3, 37.9, 32.4, 29.8, 23.7, 21.3, 20.1. **HRMS** m/z: $[M + H]^+$ calc'd for $[C_{21}H_{28}N_3O]^+$ expect 338.2227; found 338.2234. **$[\alpha]_D^{25.0}$** = +222.0 (*c* 0.7, $CHCl_3$).

Diastereomer 2 (**5cb**):

^{1}H NMR (400 MHz, $CDCl_3$) δ 10.17 (br. s, 1H), 8.02 (dd, *J* = 5.0, 1.5 Hz, 1H), 7.57 (d, *J* = 7.8 Hz, 2H), 7.46 (ddd, *J* = 8.6, 7.0, 1.8 Hz, 1 H), 7.35 (t, *J* = 7.9 Hz, 2H), 7.13 (t, *J* = 7.4 Hz, 1H), 6.52 (t, *J* = 6.1 Hz, 1H), 6.43 (d, *J* = 8.6 Hz, 1H), 4.30-4.20 (m, 1H), 3.50 (t, *J* = 9.5 Hz, 1H), 3.29 (q, *J* = 8.2 Hz, 1H), 2.44-2.25 (m, 3H), 2.14-2.00 (m, 3H), 1.87-1.77 (m, 1H), 1.34 (t, *J* = 11.7 Hz, 1H), 1.02 (d, *J* = 6.8 Hz, 3H), 0.91 (d, *J* = 6.8 Hz, 3H). **^{13}C NMR** (101 MHz, $CDCl_3$) δ 173.8, 156.9, 147.9, 139.0, 137.8, 128.9, 124.7, 122.9, 111.6, 107.6, 56.8, 51.1, 47.6, 31.6, 30.6, 28.6, 23.8, 21.1, 18.6. **HRMS** m/z: $[M + H]^+$ calc'd for $[C_{21}H_{28}N_3O]^+$ expect 338.2227; found 338.2233. **$[\alpha]_D^{25.0}$** = +93.0 (*c* 1.0, $CHCl_3$).

Methyl (*R*)-4-oxo-4-(phenylamino)-3-(((*S*)-1-(pyridin-2-yl)pyrrolidin-2-yl)methyl)butanoate (5da) and methyl (*S*)-4-oxo-4-(phenylamino)-3-(((*S*)-1-(pyridin-2-yl)pyrrolidin-2-yl)methyl)butanoate (5db)

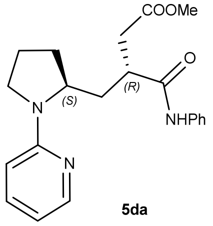

5da

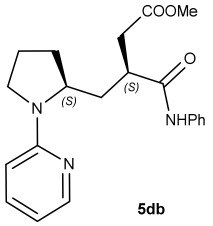

5db

Following the general procedure for the asymmetric Giese reaction using 2-(pyrrolidin-1-yl)pyridine **1a** (1.0 mL, 0.20 mmol, 0.2 M in anhydrous $PhCF_3$, 1 eq), methyl 3-(phenylcarbamoyl)but-3-enoate **4h** (66 mg, 0.30 mmol, 1.5 eq) and (Ir[dF(CF_3)ppy](dtbpy))PF_6 (1.1 mg, 1.0 µmol, 0.5 mol%) for 8 h. The diastereomeric ratio was determined from the crude mixture (2.1:1 d.r. (**5da**:**5db**) (^{1}H NMR)) and was purified without workup by column chromatography (eluting with 10% to 50% EtOAc in petroleum ether) to yield **5da** as the first eluting diastereomer (46 mg, 0.13 mmol, 63% yield, 84% ee) as a colourless oil and **5db** as the second eluting diastereomer (26 mg, 0.070 mmol, 35% yield, 86% ee) as off-white solid. Combined yield: 72 mg, 0.19 mmol, 97% yield.

Diastereomer 1 (**5da**):

^{1}H NMR (700 MHz, $CDCl_3$) δ 12.06 (br. s, 1H), 8.19 (d, *J* = 5.4 Hz, 1H), 7.74 (d, *J* = 8.3 Hz, 2H), 7.52 (t, *J* = 7.8 Hz, 1H), 7.33 (t, *J* = 7.7 Hz, 2H), 7.07 (t, *J* = 7.4 Hz, 1H), 6.65 (t, *J* = 6.0 Hz, 1H), 6.46 (d, *J* = 8.6 Hz, 1H), 4.74 (app t, *J* = 8.7 Hz, 1H), 3.61 (s, 3H), 3.52 (t, *J* = 8.9 Hz, 1H), 3.23 (app q, *J* = 9.0 Hz, 1H), 3.11-3.05 (m, 1H), 2.92 (dd, *J* = 16.7, 9.4 Hz, 1H), 2.28 (dd, *J* = 16.8, 5.4 Hz, 1H), 2.20-2.09 (m, 1H), 2.06 (dt, 13.7, 6.8 Hz, 1H), 1.93 (tt, *J* = 12.8, 7.2 Hz, 1H), 1.76-1.93 (m, 3H). **^{13}C NMR** (176 MHz, $CDCl_3$) δ 173.3, 172.8, 158.0, 146.3, 139.6, 138.2, 128.9, 123.4, 119.7, 112.3, 107.3, 55.6, 51.6, 48.3, 40.9, 39.4, 36.9, 32.4, 23.6. **HRMS** m/z: $[M + H]^+$ calc'd for $[C_{21}H_{25}N_3O_3]^+$ expect 368.1969; found 368.1971. **$[\alpha]_D^{25.0}$** = +212.4 (*c* 1.0, $CHCl_3$).

Diastereomer 2 (**5db**):

^{1}H NMR (700 MHz, $CDCl_3$) δ 10.4 (br. s, 1H), 7.97 (d, *J* = 4.3 Hz, 1H), 7.56 (d, *J* = 7.9 Hz, 2H), 7.45 (t, *J* = 7.4 Hz, 1H), 7.33 (t, *J* = 7.8 Hz, 2H), 7.14 (t, *J* = 7.4 Hz, 1H), 6.50 (t, *J* = 6.2 Hz, 1H), 6.42 (br. d, *J* = 8.3 Hz, 1H), 4.32 (br. app t, *J* = 7.3 Hz, 1H), 3.69 (s, 3H), 3.48 (t, *J* = 7.6 Hz, 1H), 3.28 (app q, *J* = 8.1 Hz, 1H), 2.97 (dd, *J* = 16.8, 5.5 Hz, 1H), 2.97-2.85 (m, 1H), 2.64 (dd, *J* = 16.5, 8.1 Hz, 1H), 2.46 (t, *J* = 12.3 Hz, 1H), 2.14-2.03 (m, 3H), 2.02-1.95 (m, 1H), 1.40 (ddd, *J* = 13.8, 11.5, 2.6 Hz, 1H). **^{13}C NMR** (176 MHz, $CDCl_3$) δ 173.6, 173.1, 158.4, 146.6, 139.9, 138.6, 129.2, 123.7, 120.0, 112.6, 107.6, 55.9, 51.9, 48.6, 41.2, 39.7, 37.2, 32.7, 23.9. **HRMS** m/z: $[M + H]^+$ calc'd for $[C_{21}H_{25}N_3O_3]^+$ expect 368.1969; found 368.1970. **$[\alpha]_D^{25.0}$** = +41.4 (*c* 1.0, $CHCl_3$).

(*S*)-*N*-Phenyl-2-(((*S*)-1-(pyridin-2-yl)pyrrolidin-2-yl)methyl)butanamide (5ea) and (*R*)-*N*-phenyl-2-(((*S*)-1-(pyridin-2-yl)pyrrolidin-2-yl)methyl)butanamide (5eb)

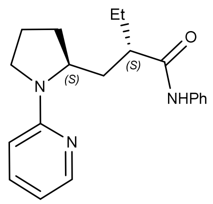

5ea

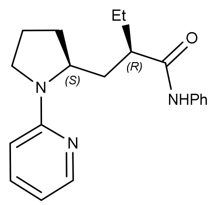

5eb

Following the general procedure for the asymmetric Giese reaction using 2-(pyrrolidin-1-yl)pyridine **1a** (1.0 mL, 0.20 mmol, 0.2 M in anhydrous $PhCF_3$, 1 eq), 2-methylene-*N*-phenylbutanamide **4i** (53 mg, 0.30 mmol, 1.5 eq) and $(Ir[dF(CF_3)ppy](dtbpy))PF_6$ (1.1 mg, 1.0 µmol, 0.5 mol%) for 8 h. The diastereomeric ratio was determined from the crude mixture (3.9:1 d.r. (**5ea**:**5eb**) (^{1}H NMR) and was purified after general acid workup procedure by column chromatography (eluting with 5% to 50% EtOAc in petroleum ether) to yield **5ea** as the first eluting diastereomer (49 mg, 0.15 mmol, 76% yield, 94% ee) and **5eb** the second eluting diastereomer (10 mg, 0.03 mmol, 15% yield, 90% ee) both as white solids. Combined yield: 59 mg, 0.18 mmol, 91%.

Diastereomer 1 (**5ea**):

^{1}H NMR (400 MHz, $CDCl_3$) δ 11.63 (br. s, 1H), 8.19 (dd, *J* = 5.1, 1.8 Hz, 1H), 7.76 (d, *J* = 8.1 Hz, 2H), 7.52 (ddd, *J* = 8.6, 7.0, 1.7 Hz, 1H), 7.33 (app t, *J* = 7.9 Hz, 2H), 7.07 (app t, *J* = 7.4 Hz, 1H), 6.64 (dd, *J* = 6.9, 5.2 Hz, 1H), 6.45 (d, *J* = 8.6 Hz, 1H), 4.66 (app q, *J* = 7.0 Hz, 1H), 3.52 (t, *J* = 9.0 Hz, 1H), 3.22 (td, *J* = 9.9, 7.7 Hz, 1H), 2.46 (tt, *J* = 9.1, 4.8 Hz, 1H), 2.23-2.10 (m, 1H), 2.10-2.00 (m, 1H), 1.96-1.79 (m, 2H), 1.70 (dd, *J* = 7.8, 5.6 Hz, 2H), 1.33-1.21 (m, 1H), 0.87 (t, *J* = 7.3 Hz, 3H). **^{13}C NMR** (101 MHz, $CDCl_3$) δ 175.2, 158.3, 146.5, 139.9, 138.4, 129.2, 123.6, 120.0, 112.3, 107.6, 56.3, 48.6, 45.7, 41.9, 32.8, 26.2, 23.9, 12.5. **HRMS** m/z: $[M + H]^+$ calc'd for $[C_{20}H_{26}N_3O]^+$ expect 324.2070; found 324.2075. **$[\alpha]_D^{25.0}$** = +296.5 (*c* 1.0, $CHCl_3$).

X-Ray Crystallography Sample preparation

The purified sample obtained from above was suspended in HCl in 1,4-dioxane (0.2 mL, 4 M) and the solvent was removed *in vacuo*. The resultant salt was then crystallized via vapour diffusion (Et_2O / DCM) to yield colourless crystals which were analysed by x-ray diffraction. The absolute configuration was determined to be *S,S*. The structure was deposited in the Cambridge Crystallographic Data Centre (deposition no.: CCDC 2183882).

Diastereomer 2 (**5eb**):

^{1}H NMR (400 MHz, $CDCl_3$) δ 9.98 (br. s, 1H), 8.04 (dd, *J* = 5.1, 1.7 Hz, 1H), 7.47 (ddd, *J* = 8.6, 7.1, 1.6 Hz, 2H), 7.33 (app t, *J* = 7.9 Hz, 2H), 7.12 (app t, *J* = 7.4 Hz, 1H), 6.53 (app t, *J* = 6.1 Hz, 1H), 6.43 (app t, *J* = 8.4 Hz, 1H), 4.28 (br. td, *J* = 5.3, 5.5 Hz, 1H), 3.50 (t, *J* = 8.2 Hz, 1H), 3.28 (app q, *J* = 8.2 Hz, 1H), 2.42 (app d, *J* = 8.3 Hz, 1H), 2.16-1.98 (m, 3H), 1.89-1.74 (m, 3H), 1.37 (app t, *J* = 11.1 Hz, 1H), 0.98 (t, *J* = 7.4 Hz, 3H); **^{13}C NMR** (101 MHz, $CDCl_3$) δ 173.9, 156.4, 147.4, 138.7, 137.8, 128.7, 125.5, 122.3, 111.4, 107.6, 56.5, 47.4, 46.2, 33.1, 30.2, 27.8, 23.5, 11.1; **HRMS** m/z: $[M + H]^+$ calc'd for $[C_{20}H_{26}N_3O]^+$ expect 324.2070; found 324.2074. **$[\alpha]_D^{25.0}$** = +64.0 (*c* 0.2, $CHCl_3$).

(*S*)-*N*-Phenyl-2-(((*S*)-1-(pyrimidin-2-yl)pyrrolidin-2-yl)methyl)butanamide (5fa) and (*R*)-*N*-phenyl-2-(((*S*)-1-(pyrimidin-2-yl)pyrrolidin-2-yl)methyl)butanamide (5fb)

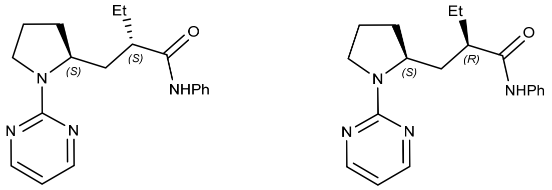

5fa **5fb**

Following the general procedure for the asymmetric Giese reaction using 2-(pyrrolidin-1-yl)pyrimidine **1e** (1.0 mL, 0.20 mmol, 0.2 M in anhydrous $PhCF_3$, 1 eq), 2-methylene-*N*-phenylbutanamide **4i** (53 mg, 0.30 mmol, 1.5 eq) and $(Ir[dF(CF_3)ppy]_2(dtpby))PF_6$ (1.1 mg, 1.0 µmol, 0.5 mol%) for 24 h. The diastereomeric ratio was determined from the crude mixture (3.2:1 d.r. (**5fa**:**5fb**) (^{1}H NMR)), which was purified without further workup by silica gel column chromatography (eluting with 0% to 50% Et_2O in *n*-hexane), followed by additional purification by silica gel column chromatography (5% MeCN in DCM) to afford **5fa** as the first eluting diastereomer (25 mg, 0.076 mmol, 38% yield, 90% ee) and **5fb** as the second eluting diastereomer (6.0 mg, 0.018 mmol, 9% yield, 76% ee) both as white solids. Combined yield: 31 mg, 0.094 mmol, 47% yield.

Diastereomer 1 (**5fa**):

^{1}H NMR (400 MHz, CD_3CN) δ = 9.59 (br. s, 1H), 8.31 (d, *J* = 4.7 Hz, 2H), 7.62 (d, *J* = 7.8 Hz, 2H), 7.32 (t, *J* = 7.8 Hz, 2H), 7.07 (t, *J* = 7.8 Hz, 1H), 6.55 (t, *J* = 4.7 Hz, 1H), 4.36 (q, *J* = 6.4 Hz, 1H), 3.61–3.47 (m, 2H), 2.34 (tt, *J* = 9.2, 4.6 Hz, 1H), 2.07 – 1.99 (m, 1H), 1.96 – 1.94 (m, 1H), 1.93 – 1.88 (m, 1H), 1.83 (ddd, *J* = 13.9, 7.7, 3.9 Hz, 1H), 1.76 – 1.64 (m, 3H), 1.46 – 1.40 (m, 1H),), 0.87 (t, *J* = 7.3 Hz, 3H). **^{13}C NMR** (101 MHz, CD_3CN) δ = 175.3, 161.8, 158.6, 140.3, 129.7, 124.3, 120.5, 110.3, 57.0, 47.9, 47.1, 39.0, 32.0, 26.9, 23.8, 12.2. **HRMS** m/z: $[M + H]^+$ calc'd for $[C_{19}H_{25}N_4O]^+$ expect 325.2028; found 325.2022. **$[\alpha]_D^{25.0}$** = +251.2 (*c* 1.0, $CHCl_3$).

Diastereomer 2 (**5fb**):

^{1}H NMR (700 MHz, CD_3CN) δ = 8.65 (br. s, 1H), 8.24 (d, *J* = 4.8 Hz, 2H), 7.54 (d, *J* = 7.8 Hz, 2H), 7.31 (t, *J* = 7.8 Hz, 2H), 7.08 (t, *J* = 7.8 Hz, 1H), 6.48 (t, *J* = 4.8 Hz, 1H), 4.28–4.24 (m, 1H), 3.57–3.50 (m, 2H), 2.38–2.33 (m, 1H), 2.23 (ddd, *J* = 13.7, 10.6, 3.9 Hz, 1H), 2.06 – 1.98 (m, 2H), 1.92 – 1.89 (m, 1H), 1.89 – 1.83 (m, 1H), 1.71–1.62 (m, 2H), 1.48 (ddd, *J* = 13.7, 9.6, 3.9 Hz, 1H), 0.92 (t, *J* = 7.4 Hz, 3H). **^{13}C NMR** (101 MHz, CD_3CN) δ = 174.7, 161.5, 158.6, 140.0, 129.6, 124.6, 121.4, 110.1, 56.7, 47.7, 47.5, 36.0, 31.0, 27.8, 23.8, 12.0. **HRMS** m/z: $[M + H]^+$ calc'd for $[C_{19}H_{25}N_4O]^+$ expect 325.2028; found 325.2023. **$[\alpha]_D^{25.0}$** = +29.6 (*c* 0.5, $CHCl_3$).

(*S*)-*N*-Phenyl-2-(((*S*)-1-(pyridin-2-yl)piperidin-2-yl)methyl)butanamide (5ga) and (*R*)-*N*-phenyl-2-(((*S*)-1-(pyridin-2-yl)piperidin-2-yl)methyl)butanamide (5gb)

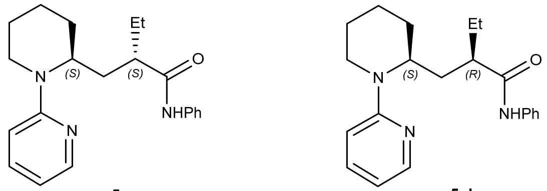

Following the general procedure for the asymmetric Giese reaction using 2-(piperidin-1-yl)pyridine **1f** (1.0 mL, 0.20 mmol, 0.2 M in anhydrous $PhCF_3$, 1 eq), 2-methylene-*N*-phenylbutanamide **4i** (53 mg, 0.30 mmol, 1.5 eq) and $(Ir[dF(CF_3)ppy]_2(dtpby))PF_6$ (1.1 mg, 1.0 µmol, 0.5 mol%) for 8 h. The diastereomeric ratio was determined from the crude mixture (2.3:1 d.r. (**5ga**:**5gb**) (^{1}H NMR)), which was purified was purified without further workup by silica gel column chromatography (eluting with 10% EtOAc to 20% EtOAc in 0.5% TEA in petroleum ether) to yield **5ga** as the first eluting diastereomer (37% yield, 25 mg, 0.074 mmol, 78% ee) and **5gb** as the second eluting diastereomer (15% yield, 10 mg, 0.030 mmol, 80% ee) both as colourless films. Combined yield: 35 mg, 0.10 mmol, 52% yield.

Diastereomer 1 (**5ga**):

^{1}H NMR (400 MHz, $CDCl_3$) δ 9.99 (br. s, 1H), 8.18 (d, *J* = 5.0 Hz, 1H), 7.63 (d, *J* = 7.9 Hz, 2H), 7.44 (t, *J* = 7.8 Hz, 1H), 7.32 (t, *J* = 7.8 Hz, 2H), 7.08 (t, *J* = 7.4 Hz, 1H), 6.66 (d, *J* = 8.7 Hz, 1H), 6.56 (t, *J* = 6.1 Hz, 1H), 4.90 (d, *J* = 11.9 Hz, 1H), 3.86 (d, *J* = 14.3 Hz, 1H), 3.19 (t, *J* = 13.4 Hz, 1H), 2.25 – 2.16 (m, 2H), 1.89 (dp, *J* = 14.5, 7.7 Hz, 1H), 1.77 – 1.60 (m, 5H), 1.58 – 1.47 (m, 2H), 1.39 (dt, *J* = 14.5, 6.9 Hz, 1H), 0.91 (t, *J* = 7.2 Hz, 3H). **^{13}C NMR** (101 MHz, $CDCl_3$) δ 174.9, 159.4, 147.2, 139.1, 138.4, 129.0, 123.8, 120.3, 112.0, 107.3, 50.6, 45.6, 40.1, 34.3, 29.9, 26.2, 25.0, 19.8, 12.4. **HRMS** m/z: $[M + H]^+$ calc'd for $[C_{21}H_{28}N_4O]^+$ expect 338.2232; found 338.2232. **$[\alpha]_D^{25.0}$** = +116.2 (*c* 1.0, $CHCl_3$).

Diastereomer 2 (**5gb**):

^{1}H NMR (700 MHz, $CDCl_3$) δ 8.12 (dd, *J* = 5.3, 2.0 Hz, 1H), 7.68 (s, 1H), 7.31 (ddd, *J* = 8.9, 6.9, 2.0 Hz, 1H), 7.27 – 7.26 (m, 1H), 7.26 – 7.24 (m, 1H), 7.20 (t, *J* = 7.5 Hz, 2H), 7.01 (t, *J* = 7.5 Hz, 1H), 6.54 – 6.44 (m, 2H), 4.91 (br. s, 1H), 3.68 (d, *J* = 14.2 Hz, 1H), 3.13 – 3.08 (m, 1H), 2.42 (dt, *J* = 14.4, 9.7 Hz, 1H), 2.32 (br. s, 1H), 1.80 – 1.55 (m, 8H), 1.50 – 1.39 (m, 1H), 0.94 (t, *J* = 7.4 Hz, 3H). **^{13}C NMR** (176 MHz, $CDCl_3$) δ 174.2, 158.9, 147.4, 138.3, 138.0, 128.7, 123.9, 120.1, 111.9, 107.5, 51.0, 48.2, 40.4, 32.0, 28.8, 27.6, 25.1, 19.7, 12.0. **HRMS** m/z: $[M + H]^+$ calc'd for $[C_{21}H_{28}N_4O]^+$ expect 338.2232; found 338.2231. **$[\alpha]_D^{25.0}$** = –161.3 (*c* 0.6, $CHCl_3$).

(*S*)-2-Benzyl-*N*-phenyl-3-((*S*)-1-(pyridin-2-yl)piperidin-2-yl)propanamide (5ha) and (*R*)-2-benzyl-*N*-phenyl-3-((*S*)-1-(pyridin-2-yl)piperidin-2-yl)propanamide (5hb)

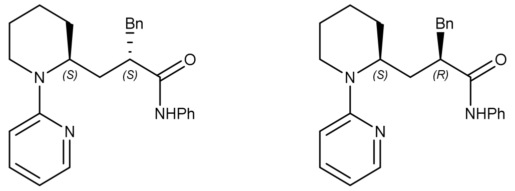

Following the general procedure for the asymmetric Giese reaction using 2-(piperidin-1-yl)pyridine **1f** (1.0 mL, 0.20 mmol, 0.2 M in anhydrous $PhCF_3$, 1 eq), 2-benzyl-*N*-phenylacrylamide **4b** (71 mg, 0.30 mmol, 1.5 eq) and(Ir[dF(CF_3)ppy]$_2$(dtpby))PF_6 (1.1 mg, 1.0 µmol, 0.5 mol%) for 8 h. The diastereomeric ratio was determined from the crude mixture (1.7:1 d.r. (**5ha**:**5hb**) (^{1}H NMR)), which was purified after general workup procedure by column chromatography (eluting with 5 to 15% EtOAc in 0.5% TEA in petroleum ether) to yield **5ha** as the first eluting diastereomer (32 mg, 0.082 mmol, 41% yield, 86% ee) as an off-white solid and **5hb** as the second eluting diastereomer (20 mg, 0.050 mmol, 25% yield, 86% ee) as a colourless film. Combined yield: 65% yield, 52 mg, 0.13 mmol.

Diastereomer 1 (**5ha**):

^{1}H NMR (500 MHz, $CDCl_3$) δ 10.27 (br. s, 1H), 8.17 (ddd, *J* = 4.9, 1.94, 0.9 Hz, 1H), 7.64 – 7.60 (m, 2H), 7.44 (ddd, *J* = 8.8, 7.0, 1.9 Hz, 1H), 7.35 – 7.30 (m, 2H), 7.26 – 7.21 (m, 2H), 7.16 (tt, *J* = 7.3, 1.1 Hz, 1H), 7.14 – 7.11 (m, 2H), 7.09 (tt, *J* = 7.4, 1.0 Hz, 1H), 6.57 – 6.54 (m, 2H), 5.00 – 4.92 (m, 1H), 3.47 (br. d, *J* = 14.8 Hz, 1H), 3.14 (dd, *J* = 13.8, 6.1 Hz, 1H), 2.72 (dd, *J* = 13.8, 9.1 Hz, 1H), 2.60 – 2.51 (m, 1H), 2.39 (ddd, *J* = 14.3, 12.7, 2.3 Hz, 1H), 2.26 (ddd, *J* = 14.0, 13.2, 0.9 Hz, 1H), 1.68 – 1.58 (m, 2H), 1.58 – 1.51 (m, 2H), 1.51 – 1.44 (m, 2H), 1.43 – 1.36 (m, 1H). **^{13}C NMR** (101 MHz, $CDCl_3$) δ 174.5, 159.5, 147.3, 130.5, 139.3, 138.6, 129.6, 129.2, 128.6, 126.4, 124.0, 120.5, 112.1, 107.4, 50.4, 45.0, 39.8, 38.3, 33.0, 25.1, 19.8. **HRMS** m/z: $[M + H]^+$ calc'd for $[C_{26}H_{30}N_3O]^+$ expect 400.2383; found 400.2387. **$[\alpha]_D^{25.0}$** = +62.5 (*c* 1.0, $CHCl_3$).

Diastereomer 2 (**5hb**):

^{1}H NMR (500 MHz, $CDCl_3$) δ 8.13 – 8.09 (m, 1H), 7.44 (br. m, 1H), 7.32 (ddd, *J* = 8.7, 7.0, 1.8 Hz, 1H), 7.25 – 7.21 (m, 2H), 7.19 – 7.14 (m, 5H), 7.11 (d, *J* = 7.6 Hz, 2H), 7.00 (t, *J* = 7.4 Hz, 1H), 6.51 (d, *J* = 8.6 Hz, 1H), 6.49 (dd, *J* = 6.9, 5.0 Hz, 1H), 4.95 – 4.88 (m, 1H), 3.69 (br. d, *J* = 14.2 Hz, 1H), 3.11 – 3.02 (ddd, *J* = 8.7, 7.0, 1.8 Hz, 2H), 2.85 (dd, *J* = 13.6, 6.7 Hz, 1H), 2.66 – 2.53 (br. m, 1H), 2.47 (dt, *J* = 14.3, 9.5 Hz, 1H), 1.73 – 1.52 (m, 7H), 1.49 – 1.38 (m, 1H). **^{13}C NMR** (101 MHz, $CDCl_3$) δ 174.5, 159.5, 147.3, 140.5, 139.3, 138.6, 129.6, 129.2, 128.6, 126.4, 124.0, 120.5, 112.2, 107.4, 50.4, 45.0, 39.8, 38.3, 33.0, 30.3, 25.1, 19.8. **HRMS** m/z: $[M + H]^+$ calc'd for $[C_{26}H_{30}N_3O]^+$ expect 400.2383; found 400.2385. **$[\alpha]_D^{25.0}$** = –64.6 (*c* 0.8, $CHCl_3$).

(*S*)-*N*-Phenyl-2-pyridine-(pyridin-2-yl)azepan-2-yl)methyl)butanamide (5ia) and (*R*)-*N*-phenyl-2-pyridine-(pyridin-2-yl)azepan-2-yl)methyl)butanamide (5ib)

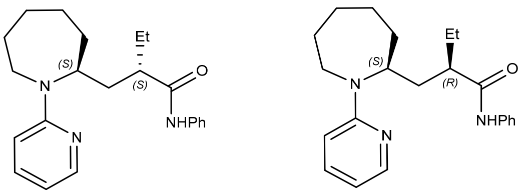

Following the general procedure for the asymmetric Giese reaction using 1-(pyridin-2-yl)azepane **1g** (1.0 mL, 0.20 mmol, 0.2 M in anhydrous $PhCF_3$, 1 eq), 2-methylene-*N*-phenylbutanamide **4i** (53 mg, 0.30 mmol, 1.5 eq) and (Ir[dF(CF_3)ppy]$_2$(dtpby))PF_6 (1.1 mg, 1.0 µmol, 0.5 mol%) for 8 h. The diastereomeric ratio was determined from the crude mixture (1.6:1 d.r. (**5ia**:**5ib**) (^{1}H NMR)) and was purified after general workup procedure by column chromatography (eluting with 0 to 10% Et_2O in DCM) to yield **5ia** as the first eluting diastereomer (32 mg, 0.92 mmol, 46% yield, 86% ee) and **5ib** as the second eluting diastereomer (29 mg, 0.082 mmol, 41% yield, 97% ee) both as off-white solids. Combined yield: 61 mg, 0.17 mmol, 87% yield.

Diastereomer 1 (**5ia**):

^{1}H NMR (400 MHz, $CDCl_3$) δ 11.06 (br. s, 1H), 8.19 (dd, *J* = 5.2, 1.7 Hz, 1H), 7.74 (d, *J* = 7.74 Hz, 2H), 7.50 (ddd, *J* = 8.8, 7.0, 1.9 Hz, 1H), 7.35 (t, *J* = 7.9 Hz, 2H), 7.08 (tt, *J* = 7.5, 1.0 Hz, 1H), 6.62-6.56 (m, 2H), 4.90 (app q, *J* = 9.4 Hz, 1H), 3.51 (d, *J* = 16.8 Hz, 1H), 3.29-3.17 (m, 1H), 2.26-2.14 (m, 1H), 2.09 (qn, *J* = 7.4 Hz, 1H), 1.88-1.76 (m, 2H), 1.75-1.64 (m, 4H), 1.56 (ddd, *J* = 14.3, 10.9, 1.40 Hz, 1H), 1.35-1.20 (m, 3H), 1.11 (q, *J* = 12.6 Hz, 1H), 0.83 (t, *J* = 7.3 Hz, 3H). **^{13}C NMR** (101 MHz, $CDCl_3$) δ 174.9, 158.4, 146.6, 139.5, 137.9, 128.9, 123.4, 119.9, 111.3, 106.3, 52.5, 44.9 ,41.5, 39.4, 36.5, 30.0, 25.7, 25.5, 24.8, 12.1. **HRMS** m/z: $[M + H]^+$ calc'd for $[C_{22}H_{30}N_3O]^+$ expect 352.2383; found 352.2380. **$[\alpha]_D^{25.0}$** = +245.9 (*c* 1.0, $CHCl_3$).

Diastereomer 2 (**5ib**):

^{1}H NMR (400 MHz, $CDCl_3$) δ 8.09 (dd, *J* = 5.3, 2.1 Hz, 1H), 7.84 (br. s, 1H), 7.33 (d, *J* = 8.0 Hz, 2H), 7.28 (ddd, *J* = 8.8, 6.9, 1.5 Hz, 1H), 7.22 (t, *J* = 7.9 Hz, 2H), 7.03 (t, *J* = 7.4 Hz, 1H), 6.46 (dd, *J* = 6.6, 5.4 Hz, 1H), 6.39 (d, *J* = 8.8 Hz, 1H), 4.84 (m, 1H), 3.39 (d, *J* = 15.6 Hz, 1H), 3.15 (dd, *J* = 14.8 , 12.1 Hz, 1H), 2.46-2.33 (m, 1H), 2.24 (qn, *J* = 7.3 Hz, 1H), 2.13 (ddd, *J* = 14.3, 10.8, 8.1 Hz, 1H), 1.86-1.76 (m, 1H), 1.76-1.48 (m, 6H), 1.38-1.21 (m, 2H), 1.14 (qn, *J* = 12.4 Hz, 1H), 0.93 (t, *J* = 7.5 Hz, 3H). **^{13}C NMR** (101 MHz, $CDCl_3$) δ 174.1, 158.0, 146.8, 138.3, 137.7, 128.5, 123.8, 120.3, 110.9, 106.5, 52.9, 47.1, 42.1, 36.2, 35.0, 30.1, 27.9, 26.1, 24.8, 11.7. **HRMS** m/z: $[M + H]^+$ calc'd for $[C_{22}H_{30}N_3O]^+$ expect 352.2383; found 352.2384. **$[\alpha]_D^{25.0}$** = –96.4 (*c* 1.0, $CHCl_3$).

(*S*)-2-Benzyl-*N*-phenyl-3-((*S*)-1-(pyridin-2-yl)azepan-2-yl)propanamide (5ja) and (*R*)-2-benzyl-*N*-phenyl-3-((*S*)-1-(pyridin-2-yl)azepan-2-yl)propanamide (5jb)

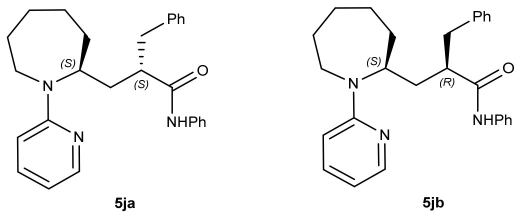

Following the general procedure for the asymmetric Giese reaction using 1-(pyridin-2-yl)azepane **1g** (1.0 mL, 0.20 mmol, 0.2 M in anhydrous $PhCF_3$, 1 eq), 2-benzyl-*N*-phenylacrylamide **4b** (71 mg, 0.30 mmol, 1.5 eq) and$(Ir[dF(CF_3)ppy]_2(dtpby))PF_6$ (1.1 mg, 1.0 µmol, 0.5 mol%) for 8 h. The diastereomeric ratio was determined from the crude mixture (1:1.1 d.r. (**5ja**:**5jb**) (^{1}H NMR)), which was purified without further workup by column chromatography (eluting with 5 to 20% EtOAc in 0.5% TEA in petroleum ether) to yield **5ja** as the first isolated minor diastereomer (35 mg, 0.084 mmol, 42% yield, 92% ee) and **5jb** as the second diastereomer which was repurified (eluting 0 to 1% TEA in DCM) (37 mg, 0.090 mmol, 45% yield, 97% ee) both as off-white solids. Combined yield: 72 mg, 0.17 mmol, 87% yield.

Diastereomer 1 (**5ja**):

^{1}H NMR (400 MHz, $CDCl_3$) δ 11.15 (br. s, 1H) 8.19 (dd, *J* = 5.1, 1.6 Hz, 1H), 7.74 (app d, *J* = 7.5 Hz, 2H), 7.49 (ddd, *J* = 8.8, 6.9, 1.9 Hz, 1H), 7.35 (t, *J* = 8.0 Hz, 2H), 7.21 (app t, *J* = 7.2 Hz, 2H), 7.15 (app t, *J* = 7.3 Hz, 1H), 7.10 (tt, *J* = 7.4, 0.9 Hz, 1H), 7.02 (d, *J* = 7.0 Hz, 2H), 6.59 (ddd, *J* = 7.10, 5.2, 0.7 Hz, 1H), 6.50 (d, *J* = 8.8 Hz, 1H), 4.93 (app q, *J* = 9.4 Hz, 1H), 3.12-3.04 (m, 1H), 3.00 (br. d, 1H), 2.71-2.50 (m, 3H), 2.05 (dt, *J* = 14.5, 7.3 Hz, 1H), 1.80-1.48 (m, 7H), 1.21-1.04 (m, 3H). **^{13}C NMR** (101 MHz, $CDCl_3$) δ 174.5, 158.4, 146.7, 140.1, 139.4, 138.0, 129.1, 128.9, 128.2, 125.9, 123.5, 119.8, 111.3, 105.9, 52.2, 43.4, 40.9, 37.2, 37.2, 36.2, 29.3, 25.4, 24.6. **HRMS** m/z: $[M + H]^+$ calc'd for $[C_{27}H_{32}N_3O]^+$ expect 414.2540; found 414.2542. **$[\alpha]_D^{25.0}$** = +144.8 (*c* 1.0, $CHCl_3$).

Diastereomer 2 (**5jb**):

^{1}H NMR (400 MHz, $CDCl_3$) δ 8.08 (ddd, *J* = 5.0, 2.0, 0.7 Hz, 1H), 7.56 (br. s, 1H), 7.27 (ddd, *J* = 8.8, 7.0, 1.9 Hz, 1H), 7.24-7.18 (m, 5H), 7.18-7.14 (m, 3H), 7.03 (sept *J* = 4.3 Hz, 1H), 6.46 (ddd, *J* = 7.0, 5.0, 0.6 Hz, 1H), 6.38 (d, *J* = 8.8 Hz, 1H), 4.94-4.79 (m, 1H), 3.38 (d, *J* = 15.0 Hz, 1H), 3.09 (ddd, *J* = 15.2, 11.5, 1.3 Hz, 1H), 3.03 (dd, *J* = 13.4, 7.7 Hz, 1H), 2.86 (dd, *J* = 13.4, 7.2 Hz, 1H), 2.76 (app q, *J* = 7.1 Hz, 1H), 2.65 (dddd, *J* = 10.5, 7.1, 7.1, 3.4 Hz, 1H), 2.24-2.09 (m, 2H), 1.82-1.73 (m, 1H), 1.73-1.64 (m, 1H), 1.64-1.47 (m, 3H), 1.32-1.08 (m, 3H). **^{13}C NMR** (101 MHz, $CDCl_3$) δ 173.3, 158.5, 147.6, 139.5, 138.0, 137.3, 129.0, 128.5, 128.4, 127.3, 124.0, 120.7, 111.0, 106.1, 52.3, 47.7, 46.0, 41.9, 40.7, 35.8, 35.0, 30.1, 26.2, 24.8. **HRMS** m/z: $[M + H]^+$ calc'd for $[C_{27}H_{32}N_3O]^+$ expect 414.2540; found 414.2545. **$[\alpha]_D^{25.0}$** = –56.0 (*c* 1.0, $CHCl_3$).

(*R*)-3-Methyl-*N*-phenyl-2-33yridine-(pyridin-2-yl)azepan-2-yl)methyl)butanamide (5ka) and (*S*)-3-methyl-*N*-phenyl-2-33yridine-(pyridin-2-yl)azepan-2-yl)methyl)butanamide (5kb)

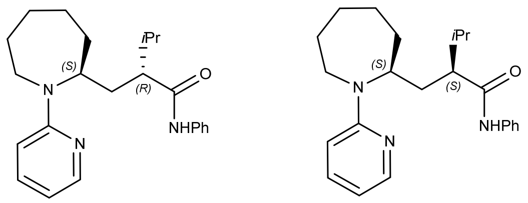

Following the general procedure for the asymmetric Giese reaction using 1-(pyridin-2-yl)azepane **1g** (1.0 mL, 0.20 mmol, 0.2 M in anhydrous $PhCF_3$, 1 eq), 3-methyl-2-methylene-*N*-phenylbutanamide **4g** (57 mg, 0.30 mmol, 1.5 eq) and $(Ir[dF(CF_3)ppy]_2(dtpby))PF_6$ (1.1 mg, 1.0 µmol, 0.5 mol%) for 8 h. The diastereomeric ratio was determined from the crude mixture (1:2.3 d.r. (**5ka**:**5kb**) ^{1}H NMR) and was purified after the general workup procedure by column chromatography (eluting with 5 to 20% EtOAc in 0.5% TEA in petroleum ether) to yield **5ka** as the first eluting diastereomer (9.2 mg, 0.025 mmol, 13% yield, 73% ee) and **5kb** as the second eluting diastereomer (32 mg, 0.087 mmol, 44% yield, 94% ee) both as white solids. Combined yield: 41 mg, 0.11 mmol, 56% yield.

Diastereomer 1 (**5ka**):

^{1}H NMR (400 MHz, $CDCl_3$) δ 11.01 (br. s, 1H), 8.19 (d, *J* = 5.3 Hz, 1H), 7.74 (d, *J* = 8.4 Hz, 1H), 7.50 (t, *J* = 7.8 Hz, 1H), 7.34 (t, *J* = 7.8 Hz, 2H), 7.08 (t, *J* = 7.4 Hz, 1H), 6.62-6.55 (m, 2H), 4.89 (q, *J* = 9.0 Hz, 1H), 3.52 (d, *J* = 15.2 Hz, 1H), 3.23 (m, 1H), 2.28-2.15 (m, 1H), 2.09 (qn, *J* = 7.40 Hz, 1H), 1.87-1.78 (m, 2H), 1.74-1.64 (m, 4H), 1.58 (d, *J* = 13.1, 11.7 Hz, 1H), 1.35-1.26 (m, 4H), 1.12 (qn, *J* = 11.8 Hz, 2H), 8.83 (t, *J* = 7.3 Hz, 3H). **^{13}C NMR** (101 MHz, $CDCl_3$) δ 175.1, 158.6, 146.8, 139.8, 138.4, 129.2, 123.7, 120.2, 111.6, 106.7, 52.9, 45.2, 41.9, 39.7, 36.8, 30.3, 30.0, 26.0, 25.9, 25.1, 12.4. **HRMS** m/z: $[M + H]^+$ calc'd for $[C_{23}H_{32}N_3O]^+$ expect 366.2540; found 366.2539. **$[\alpha]_D^{25.0}$** = +208.0 (*c* 1.0, $CHCl_3$).

Diastereomer 2 (**5kb**):

^{1}H NMR (400 MHz, $CDCl_3$) δ 8.12 (d, *J* = 4.6 Hz, 1H), 7.98 (br. s, 1H), 7.37 (d, *J* = 8.4 Hz, 2H), 7.33-7.23 (m, 3H), 7.07 (t, *J* = 7.3 Hz, 1H), 6.49 (t, *J* = 6.0 Hz, 1H), 6.40 (d, *J* = 8.7 Hz, 1H), 4.86-4.75 (m, 1H), 3.41 (d, *J* = 15.4 Hz, 1H), 3.18 (dd, *J* = 14.18, 13.11 Hz, 1H), 2.34-2.21 (m, 2H), 2.19-2.09 (m, 1H), 2.04 (sext, *J* = 6.7 Hz, 1H), 1.86-1.80 (m, 1H), 1.79-1.71 (m, 1H), 1.69-1.53 (m, 3H), 1.40-1.33 (m, 2H), 1.19 (qn, *J* = 11.4 Hz, 1H), 1.00 (d, *J* = 3.24 Hz, 3H), 0.98 (d, *J* = 3.2 Hz, 3H). **^{13}C NMR** (101 MHz, $CDCl_3$) δ 173.4, 158.3, 147.4, 138.3, 137.4, 128.5, 123.9, 120.7, 110.9, 106.3. 53.1, 51.7, 42.1, 34.8, 32.5, 31.9, 30.2, 26.4, 24.9, 20.5, 19.8. **HRMS** m/z: $[M + H]^+$ calc'd for $[C_{23}H_{32}N_3O]^+$ expect 366.2540; found 366.2546. **$[\alpha]_D^{25.0}$** = –74.9 (*c* 1.0, $CHCl_3$).

Methyl (*R*)-4-oxo-4-(phenylamino)-3-(((*S*)-1-(pyridin-2-yl)azepan-2-yl)methyl)butanoate (5la) and Methyl (*S*)-4-oxo-4-(phenylamino)-3-(((*S*)-1-(pyridin-2-yl)azepan-2-yl)methyl)butanoate (5lb)

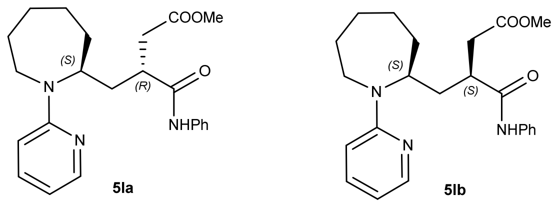

Following the general procedure for the asymmetric Giese reaction using 1-(pyridin-2-yl)azepane **1g** (1.0 mL, 0.20 mmol, 0.2 M in anhydrous $PhCF_3$, 1 eq), methyl 3-(phenylcarbamoyl)but-3-enoate **4h** (66 mg, 0.30 mmol, 1.5 eq) and $(Ir[dF(CF_3)ppy]_2(dtpby))PF_6$ (1.1 mg, 1.0 μmol, 0.5 mol%) for 8 h. The diastereomeric ratio was determined from the crude mixture (1:1 d.r. (**5la**:**5lb**) ^{1}H NMR) and was purified after the general workup procedure by column chromatography (eluting with 0% to 5% Et_2O in DCM and) to yield **5la** as the first eluting diastereomer (40 mg, 0.10 mmol, 51% yield, 75% ee) and **5lb** as the second eluting diastereomer (37 mg, 0.094 mmol, 47% yield, 93% ee) both as white solids. Combined yield: 77 mg, 0.20 mmol, 98% yield.

Diastereomer 1 (**5la**):

^{1}H NMR (400 MHz, $CDCl_3$) δ 11.58 (br. s, 1H), 8.12 (d, *J* = 4.4 Hz, 1H), 7.91 (d, *J* = 7.9 Hz, 2H), 7.53 (t, *J* = 7.5 Hz, 2H), 7.34 (t, *J* = 7.3 Hz, 2H), 7.09 (t, *J* = 7.2 Hz, 1H), 6.65 – 6.54 (m, 2H), 4.96 (q, *J* = 9.2 Hz, 1H), 3.60 (s, 3H), 3.51 (d, *J* = 15.1 Hz, 1H), 3.26 – 3.12 (m, 1H), 2.91 – 2.75 (m, 2H), 2.39 – 2.27 (m, 1H), 2.09 (dt, *J* = 14.0, 7.0 Hz, 1H), 1.83 (d, *J* = 12.8 Hz, 1H), 1.78 – 1.63 (m, 4H), 1.58 (t, *J* = 12.6 Hz, 1H), 1.36 – 1.20 (m, 2H), 1.14 (t, *J* = 12.4 Hz, 1H). **^{13}C NMR** (176 MHz, $CDCl_3$) δ 173.3, 173.0, 158.5, 146.7, 139.5, 138.2, 129.0, 123.6, 120.0, 111.7, 106.4, 52.3, 51.7, 41.7, 38.9, 38.9, 36.8, 36.5, 30.1, 25.7, 24.9. **HRMS** m/z: $[M + H]^+$ calc'd for $[C_{23}H_{30}N_3O_3]^+$ expect 396.2282; found 396.2282. $[\alpha]_D^{25.0}$ = +27.8 (*c* 1.0, $CHCl_3$).

Diastereomer 2 (**5lb**):

^{1}H NMR (500 MHz, $CDCl_3$) δ 8.31 (br. s, 1H), 8.04 (dd, *J* = 4.8, 1.7 Hz, 1H), 7.36 – 7.34 (m, 2H), 7.30 (ddd, *J* = 8.7, 7.0, 1.9 Hz, 1H), 7.27 – 7.22 (m, 2H), 7.08 – 7.03 (m, 1H), 6.46 (dd, *J* = 6.45, 5.1 Hz, 1H), 6.42 (d, *J* = 8.7 Hz, 1H), 4.97 – 4.81 (m, 1H), 3.66 (s, 3H), 3.43 (d, *J* = 16.2 Hz, 1H), 3.14 (dd, *J* = 15.7, 14.2 Hz, 1H), 2.92 – 2.82 (m, 1H), 2.78 (dd, *J* = 16.9, 7.9 Hz, 1H), 2.67 (dd, *J* = 16.7, 5.9 Hz, 1H), 2.25 (qn, *J* = 7.4 Hz, 1H), 2.15 (dt, *J* = 14.5, 8.7 Hz, 1H), 1.85 – 1.7 (m, 2H), 1.69 – 1.51 (m, 3H), 1.41 – 1.31 (m, 1H), 1.31 – 1.22 (m, 1H), 1.21 – 1.11 (m, 1H). **^{13}C NMR** (126 MHz, $CDCl_3$) δ 173.2, 172.9, 158.4 (br.), 147.7 (br.), 138.4, 137.6 (br.), 128.8, 124.2, 120.7, 111.3, 106.2 (br.), 52.5 (br.), 51.9, 42.2, 41.1, 38.3, 36.3, 35.1, 30.2, 26.5, 25.0. **HRMS** m/z: $[M + H]^+$ calc'd for $[C_{23}H_{30}N_3O_3]^+$ expect 396.2282; found 396.2282. $[\alpha]_D^{25.0}$ = –30.2 (*c* 1.0, $CHCl_3$).

(*S*)-*N*-Phenyl-3-(1-(pyridin-2-yl)azepan-2-yl)propenamide (5m)

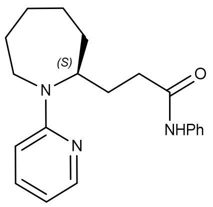

5m

Following the general procedure for the asymmetric Giese reaction using 1-(pyridin-2-yl)azepane **1g** (1.0 mL, 0.25 mmol, 0.25 M in anhydrous $PhCF_3$, 1.25 eq), *N*-phenylacrylamide (29 mg, 0.2 mmol, 1 eq) and $(Ir[dF(CF_3)ppy]_2(dtpby))PF_6$ (0.01 mmol, 1 mol %) in anhydrous DCM (2 mL) for 18 h at –40 °C (cryocooler). The crude mixture was purified by column chromatography (eluting with 30% to 40 %EtOAc in petroleum ether) to yield the title compound **5m** (7 mg, 0.02 mmol, 10% yield, 92% ee) as a colourless film.

^{1}H NMR (700 MHz, $CDCl_3$) δ 10.34 (br. s, 1H), 8.14 (dd, *J* = 5.1, 1.2 Hz, 1H), 7.64 (d, *J* = 7.8 Hz, 1H), 7.46 (ddd, *J* = 8.4, 7.3, 1.3 Hz, 2H), 7.33 (t, *J* = 7.9 Hz, 2H), 7.08 (t, *J* = 7.4 Hz, 1H), 6.57 (d, *J* = 8.8 Hz, 1H), 6.55 (d, *J* = 12.0 Hz, 1H), 4.84 (m, 1H), 3.51 (d, *J* = 15.3 Hz, 1H), 3.21 (dd, *J* = 14.7, 12.3 Hz, 1H), 2.39 (ddd, *J* = 13.7, 10.4, 3.4 Hz, 1H), 2.33 – 2.24 (m, 1H), 2.17 (m, *J* = 7.4 Hz, 1H), 1.92 – 1.86 (m, 1H), 1.86 – 1.78 (m, 2H), 1.78 – 1.62 (m, 3H), 1.36 (td, *J* = 14.5, 11.2 Hz, 1H), 1.34 – 1.25 (m, 1H), 1.17 (q, *J* = 12.3 Hz, 1H). **^{13}C NMR** (176 MHz, $CDCl_3$) δ 172.4, 158.3, 146.6, 139.3, 138.2, 129.0, 123.8, 120.2, 111.4, 106.4, 53.2, 41.9, 35.9, 34.6, 31.3, 30.1, 26.1, 25.0. **HRMS** m/z: $[M + H]^+$ calc'd for $[C_{20}H_{26}N_3O]^+$ expect 324.2070; found 324.2071. **$[\alpha]_D^{25.0}$** = +207.8 (*c* 1.0, $CHCl_3$).

(*S*)-2-Benzyl-*N*-methyl-*N*-phenyl-3-((*S*)-1-(pyridin-2-yl)pyrrolidin-2-yl)propenamide (5na) and (*R*)-2-benzyl-*N*-methyl-*N*-phenyl-3-((*S*)-1-(pyridin-2-yl)pyrrolidin-2-yl)propenamide (5nb)

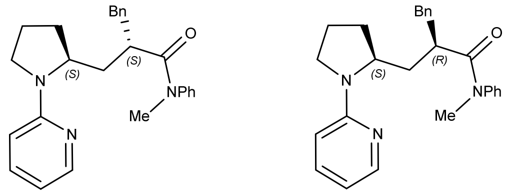

5na **5nb**

Following the general procedure for the asymmetric Giese reaction using 2-(pyrrolidin-1-yl)pyridine **1a** (1.0 mL, 0.20 mmol, 0.2 M in anhydrous $PhCF_3$, 1 eq), 2-benzyl-*N*-methyl-*N*-phenylacrylamide **4e** (75 mg, 0.30 mmol, 1.5 eq) and $(Ir[dF(CF_3)ppy]_2(dtpby))PF_6$ (1.1 mg, 1.0 µmol, 0.5 mol%) for 8 h. The diastereomeric ratio was determined from the crude mixture (1.7:1 d.r. (**5na**:**5nb**) (^{1}H NMR)), which was purified by column chromatography (eluting with 30% EtOAc in 0.5% TEA in petroleum ether) to yield mixture of the title diastereomers **5na** and **5nb** (19 mg, 0.052 mmol, 26% yield, 46% ee (**5na**, major), 8% ee (**5nb**, minor)).

^{1}H NMR (500 MHz, $CDCl_3$) δ 8.11 (ddd, *J* = 5.0, 1.9, 0.8 Hz, <1H (minor)), 8.05 (ddd, *J* = 4.9, 1.8, 0.6 Hz, 1H (major)), 7.50 (ddd, *J* = 8.7, 7.0, 1.8 Hz, <1H (minor)), 7.32 – 7.21 (m, region overlapping with the $CDCl_3$), 7.03 – 6.99 (m, 1H (major)), 6.98 – 6.96 (m, <1H (minor)), 6.63 (d, *J* = 8.6 Hz, <1H (minor)), 6.52 (ddd, *J* = 6.9, 4.9, 0.5 Hz, <1H (minor)), 6.45 (ddd, *J* = 7.0, 5.0, 0.7 Hz, 1H (major)), 6.07 (d, *J* = 8.5 Hz, 1H (major)), 3.87 – 3.80 (m, 1H (major)), 3.76 – 3.70 (m, <1H (minor)), 3.57 – 3.52 (m, <1H (minor)), 3.47 (ddd, *J* = 10.2, 7.8, 2.6 Hz, 1H (major)), 3.39 – 3.32 (m, <1H (minor)), 3.28 (ddd, *J* = 9.6, 9.6, 7.4 Hz, 1H (major)), 3.22 (s, <2H (minor)), 3.16 (s, 3H (major)), 3.03 (dd, *J* = 13.0, 9.4 Hz, 1H (major)), 2.97 (dd, *J* = 14.6, 10.3 Hz, <1H (minor)), 2.74 (dd, *J* = 13.0, 5.1 Hz, 1H), 2.69 – 2.64 (m, 1H (major), 2.63 – 2.57 (m, <2H (minor)), 2.20 (ddd, *J* = 13.2, 9.9, 3.4 Hz, <1H (minor)), 2.00 – 1.88 (m, <4H, (major, minor)), 1.86 – 1.75 (m, <3H (major, minor)), 1.65 (ddd, *J* = 13.9, 10.4, 6.1 Hz, 1H (major)), 1.54 – 1.47 (m, 1H (major)), 1.39 – 1.34 (m, <1H (minor)), 1.29 – 1.25 (m, 1H (major)). **^{13}C NMR** (126 MHz, $CDCl_3$) δ 175.0 (major), 174.7 (minor), 157.2 (minor), 156.8 (major), 148.4 (major), 148.1 (minor), 143.8 (major), 143.6 (minor), 140.1 (major), 139.8 (minor), 137.3 (minor), 136.9 (major), 130.1 (minor), 129.7 (major), 129.5 (minor), 129.4 (major), 129.4 (minor), 128.4 (minor), 128.4 (major), 127.9 (major), 127.7 (major), 127.5 (minor), 126.5 (minor), 126.4 (major), 111.5 (minor), 111.3 (major) 107.3 (minor), 106.6 (major), 55.7 (major), 55.4 (minor), 47.1 (minor), 46.9 (major), 43.2 (major), 42.3 (minor), 40.1 (minor), 39.0 (major), 37.6 (minor), 37.5 (major), 36.7 (major), 30.9 (minor) 30.1 (major), 23.4 (major), 23.1 (minor).

(*S*)-2-Benzyl-*N*-mesityl-3-((S)-1-(pyridin-2-yl)pyrrolidin-2-yl)propenamide (5oa) and (*R*)-2-benzyl-*N*-mesityl-3-((S)-1-(pyridin-2-yl)pyrrolidin-2-yl)propenamide (5ob)

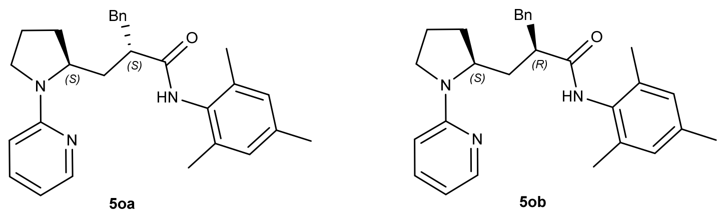

5oa **5ob**

Following the general procedure for the asymmetric Giese reaction using 2-(pyrrolidin-1-yl)pyridine **1a** (1.0 mL, 0.20 mmol, 0.2 M in anhydrous $PhCF_3$, 1 eq), 2-benzyl-*N*-mesitylacrylamide **4f** (84 mg, 0.30 mmol, 1.5 eq) and $(Ir[dF(CF_3)ppy]_2(dtpby))PF_6$ (1.1 mg, 1.0 µmol, 0.5 mol%) for 8 h. The diastereomeric ratio was determined from the crude mixture (1:1.3 d.r. (^{1}H NMR)), which was purified by column chromatography (eluting with 10% EtOAc in 0.5% TEA in petroleum ether) to yield **5oa** as the first eluting diastereomer (15 mg, 0.034 mmol, 17% yield, 39% ee) and **5ob** as the second eluting diastereomer (15 mg, 0.034 mmol, 17% yield, 27% ee) both as a colourless film. Combined yield: 30 mg, 0.068 mmol, 34%.

Diastereomer 1 (**5oa**):

^{1}H NMR (500 MHz, $CDCl_3$) δ 10.23 (br. s, 1H), 7.88 (dd, *J* = 5.0, 1.5 Hz, 1H), 7.49 (ddd, *J* = 8.7, 7.0, 1.8 Hz, 1H), 7.21 – 7.17 (m, 2H), 7.14 – 7.10 (m, 2H), 6.86 (s, 2H), 6.54 (dd, *J* = 6.7, 5.2 Hz, 1H), 6.40 (d, *J* = 8.6 Hz, 1H), 4.89 (ddd, *J* = 10.7, 7.5, 3.0 Hz, 1H), 3.31 (ddd, *J* = 9.6, 6.8, 3.0 Hz, 1H), 3.15 (q, *J* = 5.0, 1.5 Hz, 1H), 3.12 – 3.00 (m, 2H), 2.67 (dd, *J* = 13.0, 6.1 Hz, 1H), 2.25 (s, 3H), 2.07 (br. s, 6H), 2.04 – 1.99 (m, 2H), 1.91 – 1.69 (m, 4H). **^{13}C NMR** (126 MHz, $CDCl_3$) δ 174.0, 158.4, 146.7, 140.5, 137.9, 136.3, 135.3, 132.7, 129.4, 128.9, 128.2, 125.9, 112.1, 107.0, 56.2, 48.0, 44.3, 39.9, 38.6, 32.0, 23.8, 21.0, 18.8. **HRMS** m/z: $[M + H]^+$ calc'd for $[C_{28}H_{34}N_3O]^+$ expect 428.2696; found 428.2683. **$[\alpha]_D^{25.0}$** = +4.11 (*c* 1.0, $CHCl_3$).

Diastereomer 2 (**5ob**):

^{1}H NMR (500 MHz, $CDCl_3$) δ 10.56 (br. s, 1H), 7.48 (ddd, *J* = 4.9, 1.7, 0.7 Hz, 1H), 7.39 (ddd, *J* = 8.6, 6.9, 1.9 Hz, 1H), 7.30 – 7.26 (m, 2H), 7.26 – 7.23 (m, 2H), 7.21 – 7.17 (m, 1H), 6.90 (s, 2H), 6.37 (ddd, *J* = 7.1, 5.1, 0.8 Hz, 1H), 6.33 (d, *J* = 8.6 Hz, 1H), 4.30 – 4.24 (m, 1H), 3.41 (dd, *J* = 13.2, 4.0 Hz, 1H), 3.38 (ddd, *J* = 9.0, 7.5, 1.1 Hz, 1H), 3.16 (td, *J* = 9.5, 6.5 Hz, 1H), 2.79 (dd, *J* = 13.2, 9.8 Hz, 1H), 2.70 (dddd, *J* = 11.9, 9.8, 4.0, 2.1 Hz, 1H), 2.35 – 2.29 (m, 1H), 2.28 (s, 3H), 2.17 (s, 6H), 1.99 – 1.89 (m, 2H), 1.86 – 1.77 (m, 1H), 1.58 – 1.51 (m, 1H), 1.41 – 1.24 (m, 1H). **^{13}C NMR** (126 MHz, $CDCl_3$) δ 173.3, 156.5, 147.4, 140.1, 137.7, 136.5, 135.5, 133.1, 129.0, 128.5, 126.3, 111.6, 107.1, 57.1, 47.3, 46.4, 40.5, 33.0, 30.1, 23.6, 21.1, 18.7. **HRMS** m/z: $[M + H]^+$ calc'd for $[C_{28}H_{34}N_3O]^+$ expect 428.2696; found 428.2694. **$[\alpha]_D^{25.0}$** = +16.9 (*c* 1.0, $CHCl_3$).

The relative stereochemistry was assigned by analogy to **5ba** and **5bb** comparing matching shifts of the NMR spectra and the absolute stereochemistry was assigned tentatively by comparison to single crystal X-ray structure of (*S*)-*N*-phenyl-2-(((*S*)-1-(pyridin-2-yl)pyrrolidin-2-yl)methyl)butanamide (**5ea**).

(2*S*,4*S*)-2-Benzyl-4-(ethyl(pyridin-2-yl)amino)-*N*-phenylpentanamide (5pa) and (2*R*,4*S*)-2-benzyl-4-(ethyl(pyridin-2-yl)amino)-*N*-phenylpentanamide (5pb)

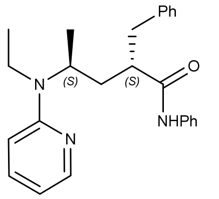

5pa

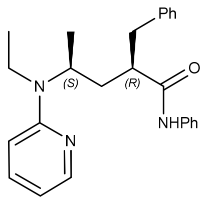

5pb

Following the general procedure for the asymmetric Giese reaction using *N*,*N*-diethyl-2-aminopyridine **1h** (1.0 mL, 0.20 mmol, 0.2 M in anhydrous $PhCF_3$, 1 eq), 2-benzyl-*N*-phenylacrylamide **4b** (71 mg, 0.30 mmol, 1.5 eq) and $(Ir[dF(CF_3)ppy]_2(dtpby))PF_6$ (1.1 mg, 1.0 µmol, 0.5 mol%) for 24h. The diastereomeric ratio was determined from the crude mixture (1.8:1 d.r. (**5pa**:**5pb**) (^{1}H NMR)) and was purified after general workup procedure by column chromatography (eluting with 15% EtOAc in 0.5% TEA in hexane) to yield **5pa** as the first eluting diastereomer (35 mg, 0.088 mmol, 45% yield, 86% ee) and **5pb** as the second eluting diastereomer (20 mg, 0.05 mmol, 25% yield, 89% ee) both as colourless films. Combined yield: 55 mg, 0.14 mmol, 70% yield.

Diastereomer 1 (**5pa**):

^{1}H NMR (400 MHz, CD_3CN) δ 9.07 (br. s, 1H), 8.14 (s, 1H), 8.06 (d, *J* = 4.9 Hz, 1H), 7.56-7.48 (m, 2H), 7.36 (t, *J* = 8.0 Hz, 2H), 7.31 (d, *J* = 7.0 Hz, 2H), 7.24 (app t, *J* = 6.7 Hz, 3H), 7.14 (t, *J* = 7.3 Hz, 1H), 6.63 (d, *J* = 8.8 Hz, 1H), 6.58 (t, *J* = 6.0 Hz, 1H), 5.03 (sext, *J* = 6.9 Hz, 1H), 3.34-3.18 (m, 2H), 3.07 (dd, *J* = 13.5, 8.1 Hz, 1H), 2.73 (dd, *J* = 13.4, 7.0 Hz, 1H), 2.62 (qn, *J* = 7.0 Hz, 1H), 1.89 (app t, *J* = 6.8 Hz, 2H), 1.20 (d, *J* = 7.0 Hz, 3H), 1.12 (t, *J* = 7.0 Hz, 3H). **^{13}C NMR** (101 MHz, CD_3CN) δ 174.3, 158.9, 147.8, 140.8, 139.7, 138.2, 129.9, 129.4, 128.9, 126.8, 124.3, 120.6, 112.1, 107.6, 49.0, 46.7, 39.5, 38.0, 36.7, 19.7, 14.0. **HRMS** m/z: $[M + H]^+$ calc'd for $[C_{25}H_{30}N_3O]^+$ expect 388.2383; found 388.2379. **$[\alpha]_D^{25.0}$** = +21.0 (*c* 0.1, $CHCl_3$).

Diastereomer 2 (**5pb**):

^{1}H NMR (400 MHz, CD_3CN) δ 8.16 (br. s, 1H), 8.08 (d, *J* = 4.52 Hz, 1H), 7.92 (br. s, 1H), 7.14 (t, *J* = 7.8 Hz, 1H), 7.33 (d, *J* = 8.1 Hz, 2H), 7.24 (t, *J* = 8.3 Hz, 2H), 7.22 (t, *J* = 7.9 Hz, 2H), 7.15 (t, *J* = 7.0 Hz, 1H), 7.09 (d, *J* = 7.4 Hz, 2H), 7.05 (t, *J* = 7.3 Hz, 1H), 6.56-6.48 (m, 2H), 5.09 (sext, *J* = 6.8 Hz, 1H), 3.34 (sept, *J* = 7.2 Hz, 2H), 2.95 (dd, *J* = 13.2, 5.4 Hz, 1H), 2.86 (dd, *J* = 12.8, 9.8 Hz, 1H), 2.54 (qn, *J* = 7.0 Hz, 1H), 2.08 (dt, *J* = 15.1, 7.5 Hz, 1H), 1.70 (dt, *J* = 13.8, 6.0 Hz, 1H), 1.22 (d, *J* = 6.7 Hz, 3H), 1.15 (t, *J* = 7.0 Hz, 3H). **^{13}C NMR** (101 MHz, CD_3CN) δ 174.1, 158.3, 147.6, 140.7, 139.4, 138.2, 129.7, 129.3, 128.8, 126.8, 124.3, 120.5, 111.9, 107.5, 48.6, 48.0, 39.7, 37.9, 37.0, 19.3, 14.2. **HRMS** m/z: $[M + H]^+$ calc'd for $[C_{25}H_{30}N_3O]^+$ expect 388.2383; found 388.2387. **$[\alpha]_D^{25.0}$** = +96.2 (*c* 0.75, $CHCl_3$).

(2*S*,4*S*)-2-Ethyl-4-(ethyl(pyridin-2-yl)amino)-*N*-phenylpentanamide (5qa) and (2*R*,4*S*)-2-ethyl-4-(ethyl(pyridin-2-yl)amino)-*N*-phenylpentanamide (5qb)

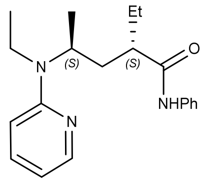

5qa

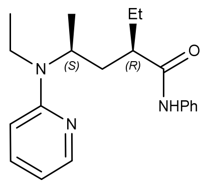

5qb

Following the general procedure for the asymmetric Giese reaction using *N*,*N*-diethyl-2-aminopyridine **1h** (1.0 mL, 0.20 mmol, 0.2 M in anhydrous $PhCF_3$, 1 eq), 2-methylene-*N*-phenylbutanamide **4i** (53 mg, 0.30 mmol, 1.5 eq) and $(Ir[dF(CF_3)ppy]_2(dtpby))PF_6$ (1.1 mg, 1.0 µmol, 0.5 mol%) for 24 h. The diastereomeric ratio was determined from crude mixture (2.0:1 d.r. (**5qa**:**5qb**) ^{1}H NMR), which was purified without further workup by silica gel column chromatography (eluting with 10% EtOAc to 20% EtOAc in 0.5% TEA in petroleum ether) to afford **5qa** as the first eluting diastereomer (22 mg, 0.068 mmol, 34% yield, 83% ee) and **5qb** as the second eluting diastereomer (15 mg, 0.046 mmol, 23% yield, 89% ee) both as colourless films. Combined yield: 37 mg, 0.11 mmol, 57% yield.

Diastereomer 1 (**5qa**):
^{1}H NMR (400 MHz, CD_3CN) δ = 8.91 (br. s, 1H), 7.98 (ddd, *J* = 5.0, 1.9, 0.8 Hz, 1H), 7.52–7.48 (m, 2H), 7.43 (ddd, *J* = 8.7, 7.0, 1.9 Hz, 1H), 7.30 (tt, *J* = 8.0, 2.0 Hz, 2H), 7.09–7.04 (m, 1H), 6.57 (dt, *J* = 8.7, 0.8 Hz, 1H), 6.48 (ddd, *J* = 7.0, 5.0, 0.8 Hz, 1H), 4.98–4.87 (m, 1H), 3.44–3.29 (m, 2H), 2.16–2.12 (m, 1H), 1.79–1.73 (m, 2H), 1.65 (m, 1H), 1.46–1.34 (m, 1H), 1.16 (t, *J* = 7.0 Hz, 3H), 1.13 (d, *J* = 6.7 Hz, 3H), 0.86 (t, *J* = 7.7 Hz, 3H). **^{13}C NMR** (101 MHz, CD_3CN) δ 175.1, 159.3, 148.3, 140.1, 138.2, 129.6, 124.4, 120.7, 112.2, 107.6, 49.2, 46.7, 38.7, 37.0, 27.2, 19.9, 14.4, 12.3. **HRMS** m/z: $[M + H]^+$ calc'd for $[C_{20}H_{28}N_3O]^+$ expect 326.2232; found 326.2221. **$[\alpha]_D^{25.0}$** = +176.8 (*c* 1.0, $CHCl_3$).

Diastereomer 2 (**5qb**):

^{1}H NMR (400 MHz, CD_3CN) δ = 8.07 (br. s, 1H), 8.03 (ddd, *J* = 4.8, 2.0, 0.8 Hz, 1H), 7.48–7.43 (m, 2H), 7.33 (ddd, *J* = 8.6, 7.1, 2.0 Hz, 1H), 7.26 (tt, *J* = 8.0, 1.7 Hz, 2H), 7.08–7.02 (m, 1H), 6.47–6.45 (m, 1H), 6.45–6.43 (m, 1H), 4.92 (m, 1H), 3.28 (q, *J* = 7.0 Hz, 2H), 2.23-2.15 (m, 1H), 2.04-1.97 (m, 1H), 1.63–1.54 (m, 3H), 1.19 (d, *J* = 6.9 Hz, 3H), 1.11 (t, *J* = 7.0 Hz, 3H), 0.86 (t, *J* = 7.4 Hz, 3H). **^{13}C NMR** (101 MHz, CD_3CN) δ = 175.1, 158.9, 148.6, 139.9, 137.9, 129.5, 124.4, 120.6, 111.9, 107.2, 48.9, 47.7, 38.1, 37.3, 27.4, 19.4, 14.5, 12.1. **HRMS** m/z: $[M + H]^+$ calc'd for $[C_{20}H_{28}N_3O]^+$ expect 326.2232; found 326.2226. **$[\alpha]_D^{25.0}$** = –131.3 (*c* 1.0, $CHCl_3$).

(2*S*,4*S*)-2-Ethyl-*N*-phenyl-4-(phenyl(pyridin-2-yl)amino)pentanamide (5ra) and (2*R*,4*S*)-2-ethyl-*N*-phenyl-4-(phenyl(pyridin-2-yl)amino)pentanamide (5rb)

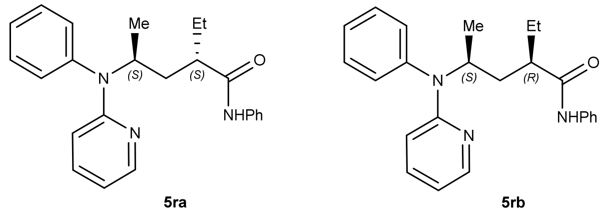

Following the general procedure for the asymmetric Giese reaction using *N*-ethyl-*N*-phenylpyridin-2-amine **1j** (1.0 mL, 0.20 mmol, 0.2 M in anhydrous $PhCF_3$, 1 eq), 2-methylene-*N*-phenylbutanamide **4i** (53 mg, 0.30 mmol, 1.5 eq) and (Ir[dF(CF_3)ppy]$_2$(dtpby))PF_6 (1.1 mg, 1.0 µmol, 0.5 mol%) for 36 h. The diastereomeric ratio was determined from the crude mixture (1.3:1 d.r. (**5ra**:**5rb**) (^{1}H NMR)), which was purified by column chromatography (eluting with DCM to 2% Et_2O and 0.5% TEA in DCM) to yield **5ra** as the first eluting diastereomer (28 mg, 0.075 mmol, 37% yield, 90% ee) and **5rb** as the second eluting diastereomer (7.0 mg, 0.019 mmol, 9% yield, 85% ee) both as yellow films. Combined yield: 35 mg, 0.094 mmol, 47% yield.

Diastereomer 1 (**5ra**):

^{1}H NMR (500 MHz, CD_3CN) δ 9.08 (br. s, 1H), 8.00 – 7.96 (m, 1H), 7.58 – 7.53 (m, 1H), 7.51 – 7.45 (m, 2H), 7.42 – 7.36 (m, 1H), 7.33 – 7.27 (m, 2H), 7.26 – 7.21 (m, 1H), 7.21 – 7.16 (m, 2H), 7.08 – 7.02 (m, 1H), 6.54 – 6.49 (m, 1H), 5.94 (td, *J* = 8.8, 0.8 Hz, 1H), 5.36 – 5.23 (m, 1H), 2.51 – 2.41 (m, 1H), 2.24 – 2.15 (m, 1H), 1.94 (s, 3H), 1.71 – 1.59 (m, 2H), 1.53 (ddd, *J* = 14.0, 10.8, 3.1 Hz, 1H), 1.38 (dddd, *J* = 14.9, 13.3, 7.3, 5.7 Hz, 1H), 1.06 (d, *J* = 6.8 Hz, 3H), 0.86 (t, *J* = 7.3 Hz, 3H). **^{13}C NMR** (126 MHz, CD_3CN) δ 174.8, 160.1, 146.5, 139.6, 139.5, 137.9, 123.1, 130.4, 129.3, 128.3, 124.0, 120.5, 113.1, 110.2, 49.4, 45.9, 40.2, 26.4, 22.2, 12.9. **HRMS** m/z: $[M + H]^+$ calc'd for $[C_{24}H_{28}N_3O]^+$ expect 374.2227; found 374.2232. **$[\alpha]_D^{25.0}$** = +29.3 (*c* 1.0, $CHCl_3$).

Diastereomer 2 (**5rb**):

^{1}H NMR (500 MHz, CD_3CN) δ 8.38 (br. s, 1H), 8.01 (ddd, *J* = 5.0, 1.9, 0.7 Hz, 1H), 7.52 (br. d, *J* = 7.9 Hz, 2H), 7.44 (t, *J* = 7.6 Hz, 2H), 7.37 – 7.33 (m, 1H), 7.28 (br. t, *J* = 7.9 Hz, 2H), 7.22 – 7.17 (m, 3H), 7.06 (br. t, *J* = 7.4 Hz, 1H), 6.49 (dd, *J* = 6.9, 5.0 Hz, 1H), 5.91 (d, *J* = 8.7 Hz, 1H), 5.16 (sext, *J* = 6.9 Hz, 1H), 2.33 (qn, *J* = 7.0 Hz, 1H), 2.01 – 1.95 (m, 1H), 1.91 (qn, *J* = 2.4 Hz, 1H), 1.61 (qn, *J* = 7.3 Hz, 2H), 1.43 (ddd, *J* = 13.7, 7.7, 5.9 Hz, 1H), 1.12 (d, *J* = 6.8 Hz, 2H), 0.85 (t, *J* = 7.4 Hz, 3H). **^{13}C NMR** (126 MHz, CD_3CN) δ 175.1, 160.1, 148.1, 142.4, 139.9, 137.6, 132.2, 130.7, 129.6, 128.2, 124.6, 120.9, 113.3, 109.8, 49.4, 47.5, 39.1, 27.4, 19.7, 12.1. **HRMS** m/z: $[M + H]^+$ calc'd for $[C_{24}H_{28}N_3O]^+$ expect 374.2227; found 374.2231. **$[\alpha]_D^{25.0}$** = +78.3 (*c* 0.1, $CHCl_3$).

(2*S*,4*S*)-4-(Benzyl(pyridin-2-yl)amino)-2-ethyl-*N*-phenylpentanamide (5sa) and (2*R*,4*S*)-4-(benzyl(pyridin-2-yl)amino)-2-ethyl-*N*-phenylpentanamide (5sb)

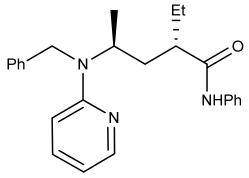

5sa

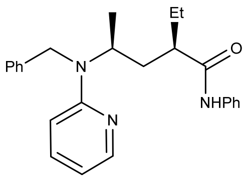

5sb

Following the general procedure for the asymmetric Giese reaction using *N*-benzyl-*N*-ethyl-2-aminopyridine **1i** (1.0 mL, 0.20 mmol, 0.2 M in anhydrous $PhCF_3$, 1 eq), 2-methylene-*N*-phenylbutanamide **4i** (53 mg, 0.30 mmol, 1.5 eq) and(Ir[dF(CF_3)ppy]$_2$(dtpby))PF_6 (6.6 mg, 6.0 μmol, 3 mol%) for 48 h. The diastereomeric ratio was determined from the crude mixture (1.5:1 d.r. (**5sa**:**5sb**) ^{1}H NMR), which was purified without further workup by silica gel column chromatography (eluting with 10% EtOAc in 0.5% TEA in petroleum ether) to afford **5sa** as the first eluting diastereomer (22 mg, 0.057 mmol, 29% yield, 78% ee) and **5sb** as the second eluting diastereomer (18 mg, 0.046 mmol, 23% yield, 91% ee) both as white solids. Combined yield: 40 mg, 0.10 mmol, 52%.

Diastereomer 1 (**5sa**):

^{1}H NMR (400 MHz, CD_3CN) δ = 8.86 (br. s, 1H), 8.04 (ddd, *J* = 5.0, 2.0, 0.8 Hz, 1H), 7.57–7.53 (m, 2H), 7.34 (t, *J* = 1.7 Hz, 1H), 7.33–7.25 (m, 6H), 7.25–7.19 (m, 1H), 7.08 (tt, *J* = 7.4, 1.2 Hz, 1H), 6.52 (ddd, *J* = 7.1, 5.0, 0.8 Hz, 1H), 6.40 (dt, *J* = 8.6, 0.8 Hz, 1H), 5.15–5.05 (m, 1H), 4.60 (ABq, *J* = 17.4 Hz, 2H), 2.29–2.20 (m, 1H), 1.84–1.69 (m, 2H), 1.64–1.55 (m, 1H), 1.30–1.21 (m, 1H), 1.15 (d, *J* = 6.8 Hz, 3H), 0.81 (t, *J* = 7.5 Hz, 3H). **^{13}C NMR** (101 MHz, CD_3CN) δ 175.1, 160.0, 148.3, 140.9, 140.1, 138.2, 129.7, 129.3, 127.5, 127.4, 124.4, 120.7, 113.1, 108.5, 49.9, 46.9, 46.7, 38.6, 27.1, 19.7, 12.2. **HRMS** m/z: $[M + H]^+$ calc'd for $[C_{25}H_{30}N_3O]^+$ expect 388.2329; found 388.2387. **$[\alpha]_D^{25.0}$** = +130.5 (*c* 1.0, $CHCl_3$).

Diastereomer 2 (**5sb**):

^{1}H NMR (400 MHz, CD_3CN) δ = 8.07 (br. s, 1H), 8.04 (ddd, *J* = 5.0, 1.9, 0.8 Hz, 1H), 7.52–7.48 (m, 2H), 7.31–7.16 (m, 8H), 7.07 (t, *J* = 7.3 Hz, 1H), 6.48 (ddd, *J* = 6.9, 5.0, 0.8 Hz, 1H), 6.30 (d, *J* = 8.7 Hz, 1H), 5.05 (tq, *J* = 7.0, 6.9 Hz, 1H), 4.55 (ABq, *J* = 17.7 Hz, 2H), 2.30–2.20 (m, 1H), 2.04 (ddd, *J* = 13.8, 8.7, 7.6 Hz, 1H), 1.65–1.55 (m, 3H), 1.19 (d, *J* = 6.7 Hz, 3H), 0.86 (t, *J* = 7.4 Hz, 3H). **^{13}C NMR** (101 MHz, CD_3CN) δ 174.9, 159.5, 148.4, 141.2, 139.9, 137.9, 129.6, 129.2, 127.4, 124.5, 120.7, 112.8, 108.0, 49.4, 47.5, 46.9, 38.1, 27.5, 19.0, 12.1. **HRMS** m/z: $[M + H]^+$ calc'd for $[C_{25}H_{30}N_3O]^+$ expect 388.2329; found 388.2383. **$[\alpha]_D^{25.0}$** = –24.6 (*c* 0.7, $CHCl_3$).

2-Benzyl-4-(methyl(pyridin-2-yl)amino)-*N*-phenylbutanamide (5t)

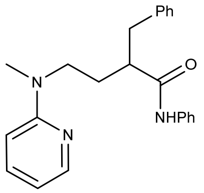

5t

Following the general procedure for the asymmetric Giese reaction using *N,N*-dimethylpyridin-2-amine (1.0 mL, 0.20 mmol, 0.2 M in anhydrous $PhCF_3$, 1 eq), 2-benzyl-*N*-phenylacrylamide **4b** (71 mg, 0.30 mmol, 1.5 eq) and $(Ir[dF(CF_3)ppy]_2(dtpby))PF_6$ (1.1 mg, 1.0 µmol, 0.5 mol%) for 8 h. The crude mixture was purified by column chromatography (eluting with 20 to 25% EtOAc in 0.5% TEA in petroleum ether) to yield the title compound **5t** (86 mg, 0.16 mmol, 81% yield, 4% ee) as a yellow film.

^{1}H NMR (400 MHz, $CHCl_3$) δ 10.30 (br. s, 1H), 8.17 (ddd, *J* = 5.0, 1.9, 0.7 Hz, 1H), 7.63 – 7.59 (m, 2H), 7.50 (ddd, *J* = 8.7, 7.0, 1.9 Hz, 1H), 7.35 – 7.29 (m, 2H), 7.25 – 7.20 (m, 2H), 7.19 – 7.05 (m, 4H), 6.61 (ddd, *J* = 7.0, 5.1, 0.7 Hz, 1H), 6.46 (d, *J* = 6.7 Hz, 1H), 4.35 (ddd, *J* = 14.6, 9.2, 5.7 Hz, 1H), 3.17 – 3.07 (m, 2H), 2.73 (dd, *J* = 13.7, 8.6 Hz, 1H), 2.67 (s, 3H), 2.66 – 2.59 (m, 1H), 1.92 – 1.79 (m, 2H). **^{13}C NMR** (101 MHz, $CDCl_3$) δ 174.0, 159.2, 147.1, 140.8, 139.1, 138.2, 129.3, 129.0, 128.4, 126.2, 123.9, 120.3, 112.0, 106.0, 47.6, 45.0, 38.1, 35.5, 30.8. **HRMS** m/z: $[M + H]^+$ calc'd for $[C_{23}H_{26}N_3O]^+$ expect 360.2070; found 360.2070.

(*R*)-3-Methyl-4-(methyl(pyridin-2-yl)amino)-*N*-phenylbutanamide (6a)

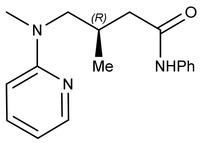

6a

Following the general procedure for the asymmetric Giese reaction using *N*,*N*-dimethylpyridin-2-amine (1.0 mL, 0.20 mmol, 0.2 M in anhydrous $PhCF_3$, 1 eq), (*E*)-*N*-phenylbut-2-enamide **4d** (32 mg, 0.30 mmol, 1.5 eq) and $(Ir[dF(CF_3)ppy]_2(dtpby))PF_6$ (6.6 mg, 6.0 µmol, 3 mol%) for 24 h. The crude was purified without further workup by silica gel column chromatography (eluting with 30% EtOAc in 0.5% TEA in petroleum ether) to yield the title compound **6a** as a white solid (28 mg, 0.099 mmol, 49% yield, 90% ee).

^{1}H NMR (500 MHz, CD_3CN) *δ* = 8.81 (br. s, 1H), 8.03 (br. s, 1H), 7.54 (d, *J* = 7.6 Hz, 2H), 7.49 (ddd, *J* = 8.6, 6.8, 1.8 Hz, 1H), 7.29 (tt, *J* = 7.9, 1.8 Hz, 2H), 7.06 (tt, *J* = 7.3, 1.2 Hz, 1H), 6.62 (d, *J* = 8.6 Hz, 1H), 6.54 (dd, *J* = 6.8, 5.6 Hz, 1H), 3.70 (dd, *J* = 14.2, 7.5 Hz, 1H), 3.33 (dd, *J* = 14.2, 7.2 Hz, 1H), 3.03 (s, 3H), 2.46 (m, 1H), 2.34 (dd, *J* = 14.1, 6.3 Hz, 1H), 2.17 (dd, *J* = 14.1, 7.2 Hz, 1H), 0.97 (d, *J* = 6.9 Hz, 3H). **^{13}C NMR** (126 MHz, CD_3CN) *δ* = 171.7, 159.3, 147.4, 140.1, 138.7, 129.7, 124.4, 120.6, 112.3, 107.2, 56.2, 42.4, 37.8, 30.8, 17.9. **HRMS** m/z: $[M + H]^+$ calc'd for $[C_{17}H_{22}N_3O]^+$ expect 284.1763; found 284.1756. **$[\alpha]_D^{25.0}$** = +122.2 (*c* 1.0, $CHCl_3$).

(*R*)-3-Methyl-*N*-phenyl-4-(phenyl(pyridin-2-yl)amino)butanamide (6b)

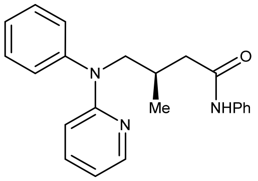

6b

Following the general procedure for the asymmetric Giese reaction using *N*-methyl-*N*-phenyl-2-aminopyridine **1j** (1.0 mL, 0.20 mmol, 0.2 M in anhydrous $PhCF_3$, 1 eq), (*E*)-*N*-phenylbut-2-enamide **4d** (32 mg, 0.30 mmol, 1.5 eq) and $(Ir[dF(CF_3)ppy]_2(dtpby))PF_6$ (2.2 mg, 2.0 µmol, 1 mol%) for 24 h. The crude was purified without further workup by silica gel column chromatography (eluting with 30% EtOAc in 0.5% TEA in petroleum ether) followed by general workup procedure for the collected fractions to yield the title compound **6b** as a white solid (55 mg, 0.16 mmol, 79% yield, 94% ee).

^{1}H NMR (400 MHz, CD_3CN) δ 8.87 (br. s, 1H), 8.13 (dd, *J* = 5.3, 2.0 Hz, 1H), 7.58 – 7.51 (m, 2H), 7.50 – 7.41 (m, 2H), 7.37 – 7.26 (m, 6H), 7.11 – 7.02 (m, 1H), 6.63 (dd, *J* = 6.7, 5.3 Hz, 1H), 6.35 (d, *J* = 8.6 Hz, 1H), 4.13 (dd, *J* = 14.1, 7.7 Hz, 1H), 3.84 (dd, *J* = 14.1, 6.7 Hz, 1H), 2.43 (dd, *J* = 13.6, 5.5 Hz, 1H), 2.39 – 2.30 (m, 1H), 2.25 (dd, *J* = 13.6, 7.0 Hz, 1H), 0.96 (d, *J* = 6.6 Hz, 3H). **^{13}C NMR** (101 MHz, CD_3CN) δ 171.6, 160.2, 148.0, 146.3, 140.1, 138.1, 130.9, 129.7, 128.6, 127.0, 124.4, 120.6, 114.2, 109.9, 55.8, 42.3, 30.9, 17.9. **HRMS** m/z: $[M + H]^+$ calc'd for $[C_{22}H_{24}N_3O]^+$ expect 346.1919; found 346.1914. **$[\alpha]_D^{25.0}$** = +106.8 (*c* 1.0, $CHCl_3$).

(*S*)-4-Methyl-*N*-phenyl-3-((phenyl(pyridin-2-yl)amino)methyl)pentanamide (6c)

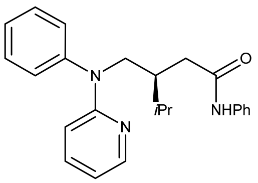

6c

Following the general procedure for the asymmetric Giese reaction using *N*-methyl-*N*-phenyl-2-aminopyridine **1j** (1.0 mL, 0.20 mmol, 0.2 M in anhydrous $PhCF_3$, 1 eq), (*E*)-4-methyl-*N*-phenylpent-2-enamide **4j** (57 mg, 0.30 mmol, 1.5 eq) and $(Ir[dF(CF_3)ppy]_2(dtpby))PF_6$ (2.2 mg, 2.0 µmol, 1 mol%) for 24 h. The crude mixture was purified by column chromatography (eluting with 20% EtOAc in 0.5% TEA in petroleum ether), followed by general workup procedure for the isolated material to yield the title compound **6c** (38 mg, 0.10 mmol, 51% yield, 95% ee) as a white solid.

^{1}H NMR (700 MHz, $CDCl_3$) δ 10.36 (br. s), 8.15 (d, *J* = 4.3 Hz, 1H), 7.59 (d, *J* = 7.8 Hz, 2H), 7.45 (t, *J* = 7.8 Hz, 2H), 7.32 (t, *J* = 7.8 Hz, 2H), 7.30 – 7.26 (m, 2H), 7.25 (d, *J* = 7.8 Hz, 2H), 7.09 (t, *J* = 7.4 Hz, 1H), 6.61 (t, *J* = 6.0 Hz, 1H), 6.34 (d, *J* = 8.7 Hz, 1H), 4.67 (dd, *J* = 14.4, 9.0 Hz, 1H), 3.66 (dd, *J* = 14.6, 5.3 Hz, 1H), 2.61 (d, *J* = 14.6, 2.5 Hz, 1H), 2.44 (dd, *J* = 13.0, 8.7 Hz, 1H), 2.13 – 2.07 (m, 1H), 1.87 (sext, *J* = 6.5 Hz, 1H), 0.91 (d, *J* = 6.8 Hz, 3H), 0.89 (d, *J* = 6.8 Hz, 3H). **^{13}C NMR** (176 MHz, $CDCl_3$) δ 171.4, 159.3 (br.), 146.6 (br.), 144.6, 139.0, 137.7, 130.3, 129.0, 127.8, 126.7, 124.0, 120.7, 113.4, 110.2, 52.1, 50.6, 36.8, 28.9, 20.5, 19.2. **HRMS** m/z: $[M + H]^+$ calc'd for $[C_{24}H_{28}N_3O]^+$ expect 374.2227; found 374.2233. **$[\alpha]_D^{25.0}$** = +65.1 (*c* 1.0, $CHCl_3$).

(*R*)-3-Methyl-*N*-phenyl-4-(pyridin-2-yl(4-(trifluoromethyl)phenyl)amino)butanamide (6d)

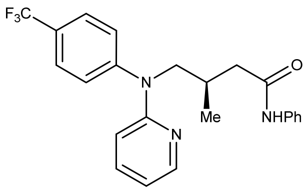

6d

Following the general procedure for the asymmetric Giese reaction using *N*-Methyl-*N*-(4-(trifluoromethyl)phenyl)-2-aminopyridine **1k** (1.0 mL, 0.20 mmol, 0.2 M in anhydrous $PhCF_3$, 1 eq), (*E*)-*N*-phenylbut-2-enamide **4d** (32 mg, 0.30 mmol, 1.5 eq) and (Ir[dF(CF_3)ppy]$_2$(dtpby))PF_6 (6.6 mg, 6.0 µmol, 3 mol%) for 24 h. The crude was purified without further workup by silica gel column chromatography (eluting with 20% EtOAc in 0.5% TEA in petroleum ether) to yield the title compound **6d** as a white solid (36 mg, 0.087 mmol, 44% yield, 97% ee).

^{1}H NMR (500 MHz, $CDCl_3$) δ = 10.00 (br. s, 1H), 8.25 (ddd, *J* = 5.2, 1.9, 0.8 Hz, 1H), 7.67 (d, *J* = 8.4 Hz, 2H), 7.60 (d, *J* = 7.7 Hz, 2H), 7.42–7.32 (m, 5H), 7.10 (t, *J* = 7.4 Hz, 1H), 6.75 (ddd, *J* = 7.0, 5.2, 0.8 Hz, 1H), 6.55 (d, *J* = 8.6 Hz, 1H), 4.56 (dd, *J* = 14.5, 8.9 Hz, 1H), 3.67 (dd, *J* = 14.5, 5.1 Hz, 1H), 2.58 (dd, *J* = 12.9, 3.2 Hz, 1H), 2.46–2.32 (m, 2H), 1.03 (d, *J* = 6.8 Hz, 3H). **^{13}C NMR** (126 MHz, $CDCl_3$) δ = 170.4, 158.6, 148.1 (br. s), 146.9, 138.9, 138.1, 129.1, 127.5 (q, $^2J_{C\text{-}F}$ = 30.2 Hz), 127.4 (q, $^3J_{C\text{-}F}$ = 3.3 Hz), 126.5, 124.1, 124.0 (q, $^1J_{C\text{-}F}$ = 271.5 Hz), 120.4, 115.1, 111.2, 55.1, 41.0, 29.8, 17.6. **^{19}F NMR** (471 MHz, $CDCl_3$) δ = –63.3. **HRMS** m/z: $[M + H]^+$ calc'd for $[C_{23}H_{23}F_3N_3O]^+$ expect 414.1793; found 414.1788. **$[\alpha]_D^{25.0}$** = +42.6 (*c* 1.0, $CHCl_3$).

(*R*)-3-Methyl-4-(phenyl(pyridin-2-yl)amino)-*N*-(4-(trifluoromethyl)phenyl)butanamide (6e)

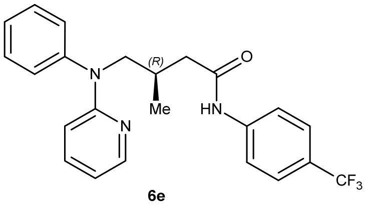

6e

Following the general procedure for the asymmetric Giese reaction using *N*-methyl-*N*-phenyl-2-aminopyridine **1j** (1.0 mL, 0.20 mmol, 0.2 M in anhydrous $PhCF_3$, 1 eq), (*E*)-*N*-(4-(trifluoromethyl)phenyl)but-2-enamide **4k** (69 mg, 0.3 mmol, 1.5 eq) and (Ir[dF(CF_3)ppy]$_2$(dtpby))PF_6 (2.2 mg, 2.0 µmol, 1 mol%) for 24 h. The crude mixture was purified by column chromatography (eluting with 5% *i*PrOH in 0.5% TEA in petroleum ether) to yield the title compound **6e** (61 mg, 0.15 mmol, 74% yield, 91% ee) as a colourless film.

^{1}H NMR (400 MHz, $CDCl_3$) δ 11.18 (br. s, 1H), 8.17 (br. s, 1H), 7.84 (d, *J* = 8.1 Hz, 2H), 7.61 (d, *J* = 7.8 Hz, 2H), 7.50 (t, *J* = 7.4 Hz, 2H), 7.35 (d, *J* = 6.8 Hz, 2H), 7.28 (d, *J* = 7.6 Hz, 2H), 6.69 (t, *J* = 5.7 Hz, 1H), 6.36 (d, *J* = 8.7 Hz, 1H), 4.64 (dd, *J* = 14.5, 9.7 Hz, 1H), 3.54 (dd, *J* = 14.7, 4.1 Hz, 1H), 2.69 (d, *J* = 12.9 Hz, 1H), 2.46 (dd, *J* = 12.9, 7.4 Hz, 1H), 2.36 (br. s, 1H), 1.02 (d, *J* = 6.7 Hz, 3H). **^{13}C NMR** (126 MHz, $CDCl_3$) δ 171.3, 159.7, 146.5, 144.7, 142.6, 138.1, 130.7, 128.3, 127.2, 126.5 (q, $^3J_{C\text{-}F}$=3.8 Hz,), 125.7 (q, $^2J_{C\text{-}F}$=32.7 Hz), 124.6 (q, $^1J_{C\text{-}F}$=271.4 Hz), 120.1, 113.8, 110.2, 55.3, 40.8, 30.2, 17.4. **^{19}F NMR** (376 MHz,

$CHCl_3$) δ –62.9. **HRMS** m/z: $[M + H]^+$ calc'd for $[C_{23}H_{22}F_3N_3O]^+$ expect 414.1788; found 414.1786. $[\alpha]_D^{25.0}$ = +116.8 (*c* 1.0, $CHCl_3$).

(*R*)-4-(Benzyl(pyridin-2-yl)amino)-3-methyl-*N*-phenylbutanamide (6f)

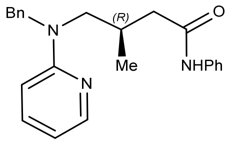

6f

Following the general procedure for the asymmetric Giese reaction using *N*-benzyl-*N*-methylpyridin-2-amine **1c** (1.0 mL, 0.20 mmol, 0.2 M in anhydrous $PhCF_3$, 1 eq), (*E*)-*N*-phenylbut-2-enamide **4d** (32 mg, 0.30 mmol, 1.5 eq) and $(Ir[dF(CF_3)ppy]_2(dtpby))PF_6$ (11 mg, 0.010 mmol, 5 mol%) for 24 h. The crude mixture was purified by column chromatography (eluting with 20 to 25% EtOAc in 0.5% TEA in petroleum ether) to yield the title compound **6f** (37 mg, 0.10 mmol, 51% yield, 95% ee) as a colourless film.

^{1}H NMR (500 MHz, $CDCl_3$) δ 10.2 (br. s, 1H), 8.17 (ddd, *J* = 5.0, 1.7, 0.6 Hz, 1H), 7.65 – 7.60 (m, 2H), 7.37 (ddd, *J* = 8.9, 7.2, 2.0 Hz, 1H), 7.34 – 7.29 (m, 4H), 7.28 – 7.22 (m, 1H), 7.17 (dd, *J* = 7.4, 1.3 Hz, 2H), 7.10 (tt, *J* = 7.4, 1.0 Hz, 1H), 6.59 (ddd, *J* = 7.2, 5.1, 0.6 Hz, 1H), 6.44 (dt, *J* = 8.6, 0.6 Hz, 1H), 4.69 (dd, *J* = 24.6, 17.4 Hz, 2H), 4.34 (dd, *J* = 14.5, 8.9 Hz, 1H), 3.18 (dd, *J* = 14.7, 5.1 Hz, 1H), 2.56 – 2.43 (m, 2H), 2.39 (dd, *J* = 13.2, 7.2 Hz, 1H), 1.08 (d, *J* = 6.7 Hz, 3H). **^{13}C NMR** (101 MHz, $CDCl_3$) δ 170.7, 158.6, 147.0, 139.0, 138.1, 137.3, 129.0, 128.9, 127.3, 126.4, 123.9, 120.4, 112.5, 107.2, 54.1, 52.6, 41.5, 30.3, 17.8. **HRMS** m/z: $[M + H]^+$ calc'd for $[C_{23}H_{26}N_3O]^+$ expect 360.2070; found 360.2075. $[\alpha]_D^{25.0}$ = +67.6 (*c* 1.0, $CHCl_3$).

(*R*)-3-((Benzyl(pyridin-2-yl)amino)methyl)-*N*,5-diphenylpentanamide (6g)

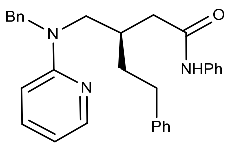

6g

Following the general procedure for the asymmetric Giese reaction using *N*-benzyl-*N*-methyl-2-aminopyridine **1c** (1.0 mL, 0.20 mmol, 0.2 M in anhydrous $PhCF_3$, 1 eq), (*E*)-*N*,5-diphenylpent-2-enamide **4l** (75 mg, 0.30 mmol, 1.5 eq) and (Ir[dF(CF_3)ppy]$_2$(dtpby))PF_6 (11 mg, 0.010 mmol, 5 mol%) for 24 h in anhydrous DCM. The crude was purified without further workup by silica gel column chromatography (eluting with 20% EtOAc in 0.5% TEA in petroleum ether) to yield the title compound **6g** as a white solid (32 mg, 0.071 mmol, 36% yield, 97% ee).

^{1}H NMR (500 MHz, $CDCl_3$) δ = 10.53 (br. s, 1H), 8.17 (ddd, *J* = 5.1, 1.9, 0.6 Hz, 1H), 7.64 (dd, *J* = 8.5, 0.9 Hz, 2H), 7.39–7.31 (m, 5H), 7.29–7.26 (m, 1H), 7.26–7.21 (m, 2H), 7.18–7.14 (m, 5H), 7.10 (tt, *J* = 7.5, 1.1 Hz, 1H), 6.60 (ddd, *J* = 7.0, 5.1, 0.6 Hz, 1H), 6.42 (d, *J* = 8.8 Hz, 1H), 4.59 (ABq, *J* = 17.6, 2H), 4.48 (dd, *J* = 14.7, 9.7 Hz, 1H), 3.21 (dd, *J* = 14.7, 4.3 Hz, 1H), 2.85 (ddd, *J* = 13.8, 9.5, 6.1 Hz, 1H), 2.71–2.63 (m, 2H), 2.45 (dd, *J* = 13.8, 7.7 Hz, 1H), 2.29 (br. s, 1H), 1.88–1.78 (m, 1H), 1.69–1.61 (m, 1H). **^{13}C NMR** (126 MHz, $CDCl_3$) δ = 170.5, 158.7, 146.8, 142.0, 139.2, 138.3, 137.0, 129.0, 128.9, 128.6, 128.5, 127.3, 126.4, 126.0, 123.9, 120.3, 112.6, 107.3, 52.2, 52.1, 39.1, 34.8, 33.5, 33.4. **HRMS** m/z: $[M + H]^+$ calc'd for $[C_{30}H_{32}N_3O]^+$ expect 450.2545; found 450.2537. **$[\alpha]_D^{25.0}$** = +93.5 (*c* 1.0, $CHCl_3$).

(*S*)-3-(((2-((tert-butyldimethylsilyl)oxy)ethyl)(pyridin-2-yl)amino)methyl)-4-methyl-*N*-phenylpentanamide (6h)

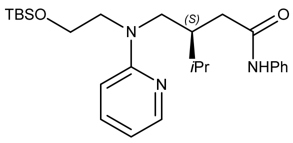

6h

Following the general procedure for the asymmetric Giese reaction using *N*-(2-((*tert*-butyldimethylsilyl)oxy)ethyl)-*N*-methylpyridin-2-amine **1l** (1.0 mL, 0.20 mmol, 0.2 M in anhydrous $PhCF_3$, 1 eq), (*E*)-4-methyl-*N*-phenylpent-2-enamide **4j** (57 mg, 0.30 mmol, 1.5 eq) and $(Ir[dF(CF_3)ppy]_2(dtpby))PF_6$ (11 mg, 0.010 mmol, 5 mol%) for 24 h. The crude mixture was purified by column chromatography (eluting with 15 to 20% EtOAc in 0.5% TEA in petroleum ether) to yield the title compound **6h** (36 mg, 0.080 mmol, 40% yield, 94% ee) as a colorless film.

^{1}H NMR (500 MHz, $CDCl_3$) δ 9.77 (br. s, 1H), 8.06 (ddd, *J* = 5.1, 2.0, 0.8 Hz, 1H), 7.50 (d, *J* = 7.7 Hz, 2H), 7.40 (ddd, *J* = 8.8, 7.0, 1.9 Hz, 1H), 7.31 – 7.27 (m, 2H), 7.08 (tt, *J* = 7.4, 0.9 Hz, 1H), 6.59 (dt, *J* = 8.8, 0.7 Hz, 1H), 6.52 (ddd, *J* = 7.0, 5.1, 0.6 Hz, 1H), 4.22 (dd, *J* = 14.2, 7.3 Hz, 1H), 3.81 – 3.74 (m, 1H), 3.60 (dt, *J* = 14.9, 5.9 Hz, 1H), 3.43 (dt, *J* = 15.0, 6.5 Hz, 1H), 3.23 (dd, *J* = 14.6, 6.2 Hz, 1H), 2.49 – 2.39 (m, 1H), 2.30 – 2.22 (m, 1H), 1.90 – 1.82 (m, 1H), 0.99 (dd, *J* = 14.3, 9.9 Hz, 6H), 0.86 (s, 9H), 0.01 (d, *J* = 1.3 Hz, 9H). **^{13}C NMR** (126 MHz, $CDCl_3$) δ 171.4, 158.4, 147.4, 138.9, 137.7, 128.9, 124.1, 120.9, 111.8, 106.8, 60.2, 51.5, 50.8, 40.0, 36.6, 28.6, 26.0, 20.7, 18.8, 18.4, –5.3, –5.31. **HRMS** m/z: $[M + H]^+$ calc'd for $[C_{26}H_{42}N_3O_2Si]^+$ expect 456.3041; found 456.3046. **$[\alpha]_D^{25.0}$** = +13.3 (*c* 1.0, $CHCl_3$).

(3*R*)-2,3-Dimethyl-4-(methyl(pyridin-2-yl)amino)-*N*-phenylbutanamide (6i)

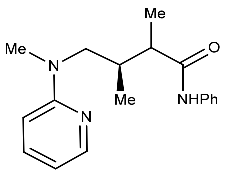

6i

Following the general procedure for the asymmetric Giese reaction using *N*,*N*-dimethylpyridin-2-amine (1.0 mL, 0.20 mmol, 0.2 M in anhydrous $PhCF_3$, 1 eq), (*E*)-2-methyl-*N*-phenylbut-2-enamide **4m** (53 mg, 0.30 mmol, 1.5 eq) and $(Ir[dF(CF_3)ppy]_2(dtpby))PF_6$ (6.6 mg, 6.0 µmol, 3 mol%) for 24 h. The diastereomeric ratio was determined from the crude mixture (2.0:1 d.r. (**6ia**:**6ib**) ^{1}H NMR), which was purified without further workup by silica gel column chromatography (eluting with 20% to 30% EtOAc in 0.5% TEA in petroleum ether) to afford **6ia** as the first eluting diastereomer (31 mg, 0.10 mmol, 52% yield, 91% ee) and **6ib** as the second eluting diastereomer (15 mg, 0.050 mmol, 25% yield, 97% ee) both as white solids. Combined yield: 46 mg, 0.15 mmol, 77%.

Diastereomer 1 (**6ia**):

^{1}H NMR (400 MHz, $CDCl_3$) δ = 11.25 (br. s, 1H), 8.16 (d, *J* = 4.9 Hz, 1H), 7.74 (d, *J* = 8.1 Hz, 2H), 7.55 (t, *J* = 7.7 Hz, 1H), 7.33 (t, *J* = 7.7 Hz, 2H), 7.08 (t, *J* = 7.4 Hz, 1H), 6.63 (t, *J* = 6.2 Hz, 1H), 6.58 (d, *J* = 8.8 Hz, 1H), 4.52 (dd, *J* = 15.0, 11.6 Hz, 1H), 3.01 (s, 3H), 2.86 (d, *J* = 15.0 Hz, 1H), 2.67 (q, *J* = 6.9 Hz, 1H), 2.01–1.89 (m, 1H), 1.16 (d, *J* = 6.9 Hz, 3H), 0.94 (d, *J* = 7.0 Hz, 3H). **^{13}C NMR** (101 MHz, $CDCl_3$) δ = 172.7, 158.8, 146.3, 139.6, 138.7, 129.0, 123.5, 120.0, 112.2, 106.2, 54.8, 40.2, 37.7, 36.5, 16.6, 13.6. **HRMS** m/z: $[M + H]^+$ calc'd for $[C_{18}H_{24}N_3O]^+$ expect 298.1919; found 298.1917. **$[\alpha]_D^{25.0}$** = –332.2 (*c* 1.0, $CHCl_3$).

Diastereomer 2 (**6ib**):

^{1}H NMR (400 MHz, $CDCl_3$) δ = 8.54 (br. s, 1H), 8.14 (d, *J* = 5.1 Hz, 1H), 7.49–7.39 (m, 3H), 7.30 – 7.22 (m, 2H), 7.05 (t, *J* = 7.2 Hz, 1H), 6.56 (dd, *J* = 7.1, 5.1 Hz, 1H), 6.50 (d, *J* = 8.8 Hz, 1H), 3.89 (dd, *J* = 14.2, 5.4 Hz, 1H), 3.35 (dd, *J* = 14.2, 6.5 Hz, 1H), 3.01 (s, 3H), 2.44–2.35 (m, 2H), 1.23 (d, *J* = 5.7 Hz, 3H), 0.99 (d, *J* = 5.9 Hz, 3H). **^{13}C NMR** (101 MHz, $CDCl_3$) δ = 174.9, 158.4, 146.7, 138.6, 138.2, 128.9, 123.9, 120.2, 111.8, 106.6, 55.5, 45.3, 38.3, 35.3, 15.2, 15.1. **HRMS** m/z: $[M + H]^+$ calc'd for $[C_{18}H_{24}N_3O]^+$ expect 298.1919; found 298.1913. **$[\alpha]_D^{25.0}$** = +126.6 (*c* 1.0, $CHCl_3$).

(2*R*)-2-((Methyl(pyridin-2-yl)amino)methyl)-*N*-phenylcyclohexane-1-carboxamide (6j)

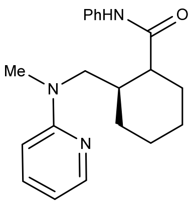

6j

Following the general procedure for the asymmetric Giese reaction using *N*,*N*-dimethylpyridin-2-amine (1.0 mL, 0.20 mmol, 0.2 M in anhydrous $PhCF_3$, 1 eq), *N*-phenylcyclohex-1-ene-1-carboxamide **4n** (60 mg, 0.30 mmol, 1.5 eq) and $(Ir[dF(CF_3)ppy]_2(dtpby))PF_6$ (6.6 mg, 6.0 µmol, 3 mol%) for 24 h. The diastereomeric ratio was determined from the crude mixture (1.2:1 d.r. (**6ja**:**6jb**) ^{1}H NMR)), which was purified without further workup by silica gel column chromatography (eluting with 20% EtOAc in 0.5% TEA in petroleum ether) to afford **6ja** as the first eluting diastereomer (23 mg, 0.071 mmol, 35% yield, 89% ee) and **6jb** as the second eluting diastereomer (25 mg, 0.077 mmol, 39% yield, 97% ee) both as white solids. Combined yield: 48 mg, 0.15 mmol, 74%.

Diastereomer 1 (**6ja**):

^{1}H NMR (500 MHz, $CDCl_3$) δ = 9.20 (br. s, 1H), 8.06 (ddd, *J* = 5.1, 1.9, 0.8 Hz, 1H), 7.53–7.49 (m, 2H), 7.41 (ddd, *J* = 8.6, 7.0, 1.9 Hz, 1H), 7.29 (tt, *J* = 8.0, 1.7 Hz, 2H), 7.08 (tt, *J* = 7.4, 1.1 Hz, 1H), 6.52 (ddd, *J* = 7.0, 5.1, 0.8 Hz, 1H), 6.48 (d, *J* = 8.6 Hz, 1H), 4.14 (dd, *J* = 14.6, 5.9 Hz, 1H), 3.28 (dd, *J* = 14.6, 8.3 Hz, 1H), 3.07 (s, 3H), 2.65 (td, *J* = 8.2, 4.1 Hz, 1H), 2.47 (br. s, 1H), 2.02–1.93 (m, 1H), 1.92–1.81 (m, 2H), 1.81–1.69 (m, 2H), 1.51–1.39 (m, 3H). **^{13}C NMR** (101 MHz, $CDCl_3$) δ = 173.2, 158.2, 146.9, 138.7, 138.0, 128.9, 124.1, 121.1, 111.6, 106.6, 51.6, 45.3, 38.6, 36.1, 27.7, 26.2, 24.4, 23.1. **HRMS** m/z: $[M + H]^+$ calc'd for $[C_{20}H_{26}N_3O]^+$ expect 324.2076; found 324.2073. $[\alpha]_D^{25.0}$ = –108.5 (*c* 1.0, $CHCl_3$).

Diastereomer 2 (**6jb**):

^{1}H NMR (500 MHz, $CDCl_3$) δ = 8.58 (br. s, 1H), 8.09 (ddd, *J* = 5.1, 1.9, 0.7 Hz, 1H), 7.42–7.38 (m, 2H), 7.35 (ddd, *J* = 8.7, 7.1, 1.9 Hz, 1H), 7.26–7.21 (m, 2H), 7.05 (tt, *J* = 7.4, 1.2 Hz, 1H), 6.49 (ddd, *J* = 7.1, 5.1, 0.7 Hz, 1H), 6.42 (d, *J* = 8.7 Hz, 1H), 4.02 (dd, *J* = 14.2, 5.6 Hz, 1H), 3.12 (dd, *J* = 14.2, 8.0 Hz, 1H), 2.98 (s, 3H), 2.30–2.20 (m, 1H), 2.13 (dd, *J* = 11.7, 3.7 Hz, 1H), 2.09–2.02 (m, 1H), 1.90–1.84 (m, 1H), 1.84–1.78 (m, 1H), 1.78–1.72 (m, 1H), 1.59–1.50 (m, 1H), 1.30–1.24 (m, 2H), 1.14–1.04 (m, 1H). **^{13}C NMR** (101 MHz, $CDCl_3$) δ = 174.4, 158.6, 147.0, 138.5, 137.8, 128.8, 124.1, 120.7, 111.6, 106.5, 55.4, 50.5, 38.4, 37.7, 31.6, 30.3, 25.9, 25.6. **HRMS** m/z: $[M + H]^+$ calc'd for $[C_{20}H_{26}N_3O]^+$ expect 324.2076; found 324.2073. $[\alpha]_D^{25.0}$ = +65.1 (*c* 1.0, $CHCl_3$).

(*R*)-4-Methyl-*N*-phenyl-3-((*S*)-1-(pyridin-2-yl)azetidin-2-yl)pentanamide (6ka) and (*S*)-4-methyl-*N*-phenyl-3-((*S*)-1-(pyridin-2-yl)azetidin-2-yl)pentanamide (6kb)

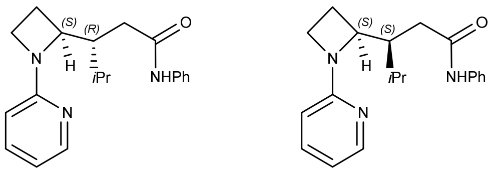

6ma **6mb**

Following the general procedure for the asymmetric Giese reaction using 2-(azetidin-1-yl)pyridine **1m** (1.0 mL, 0.20 mmol, 0.2 M in anhydrous $PhCF_3$, 1 eq), (*E*)-4-methyl-*N*-phenylpent-2-enamide **4j** (57 mg, 0.30 mmol, 1.5 eq) and $(Ir[dF(CF_3)ppy]_2(dtpby))PF_6$ (6.6 mg, 6.0 µmol, 3 mol%) for 24 h. The diastereomeric ratio was determined from the crude mixture (1:5.0 d.r. (**6ka**:**6kb**) ^1H NMR)), which was purified without further workup by silica gel column chromatography (eluting with 20% to 40% EtOAc in 0.5% TEA in petroleum ether) to yield **6ka** as the first eluting diastereomer (8.0 mg, 0.025 mmol, 12% yield, 99% ee) and **6kb** as the second eluting diastereomer (40 mg, 0.12 mmol, 62% yield, 99% ee) both as white solids. Combined yield: 48 mg, 0.15 mmol, 74%.

Diastereomer 1 (**6ka**):

^{1}H NMR (500 MHz, $CDCl_3$) δ 10.35 (br. s, 1H), 8.15 (ddd, *J* = 5.1, 1.9, 0.9 Hz, 1H), 7.51 (ddd, *J* = 8.7, 7.1, 1.9 Hz, 1H), 7.46 – 7.41 (m, 2H), 7.26 – 7.21 (m, 2H), 7.02 (tt, *J* = 7.4, 1.2 Hz, 1H), 6.70 (ddd, *J* = 7.1, 5.1, 0.9 Hz, 1H), 6.37 (dt, *J* = 8.7, 0.9 Hz, 1H), 4.65 (ddd, *J* = 10.4, 8.4, 5.8 Hz, 1H), 4.14 (ddd, *J* = 9.0, 7.8, 5.3 Hz, 1H), 3.72 (ddd, *J* = 9.9, 7.8, 6.6 Hz, 1H), 2.69 (dd, *J* = 13.9, 7.4 Hz, 1H), 2.63 – 2.51 (m, 2H), 2.22 (m, 2H), 1.99-1.92 (m, 1H), 1.11 (d, *J* = 6.9 Hz, 3H), 0.94 (d, *J* = 6.9 Hz, 3H). **^{13}C NMR** (126 MHz, $CDCl_3$) δ 172.3, 162.5, 146.7, 139.4, 137.9, 128.9, 123.4, 119.5, 113.8, 108.1, 66.2, 50.3, 36.5, 28.3, 24.4, 21.2, 18.5. **HRMS** m/z: $[M + H]^+$ calc'd for $[C_{20}H_{26}N_3O]^+$ expect 324.2076; found 324.2064. **$[\alpha]_D^{25.0}$** = +132.7 (*c* 0.7, $CHCl_3$).

Diastereomer 2 (**6kb**):

^{1}H NMR (400 MHz, $CDCl_3$) δ 9.46 (br. s, 1H), 8.18 (ddd, *J* = 5.1, 1.9, 1.0 Hz, 1H), 7.48 (ddd, *J* = 8.6, 7.2, 1.9 Hz, 1H), 7.42 – 7.34 (m, 2H), 7.25 – 7.20 (m, 2H), 7.01 (tt, *J* = 7.2, 1.2 Hz, 1H), 6.67 (ddd, *J* = 7.2, 5.1, 1.0 Hz, 1H), 6.33 (dt, *J* = 8.6, 1.0 Hz, 1H), 4.71 (td, *J* = 7.8, 3.0 Hz, 1H), 3.97 (td, *J* = 8.5, 5.2 Hz, 1H), 3.78 (dt, *J* = 8.5, 7.6 Hz, 1H), 2.98 (dd, *J* = 13.4, 8.1 Hz, 1H), 2.35 – 2.18 (m, 4H), 2.14-2.03 (m, 1H), 1.03 (d, *J* = 6.8 Hz, 3H), 0.98 (d, *J* = 6.7 Hz, 3H). **^{13}C NMR** (101 MHz, $CDCl_3$) δ 173.0, 161.6, 147.7, 138.9, 137.7, 128.9, 123.6, 119.6, 113.7, 107.2, 65.4, 49.1, 45.9, 36.4, 28.9, 21.9, 19.9, 19.8. **HRMS** m/z: $[M + H]^+$ calc'd for $[C_{20}H_{26}N_3O]^+$ expect 324.2076; found 324.2064. **$[\alpha]_D^{25.0}$** = –107.0 (*c* 1.0, $CHCl_3$).

(*R*)-*N*-Phenyl-3-((*S*)-1-(pyridin-2-yl)azepan-2-yl)butanamide (6l)

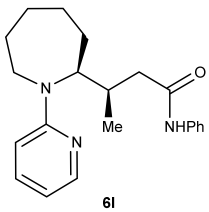

6l

Following the general procedure for the asymmetric Giese reaction using 1-(pyridin-2-yl)azepane **1g** (1.0 mL, 0.20 mmol, 0.2 M in anhydrous $PhCF_3$, 1 eq), (*E*)-*N*-phenylbut-2-enamide **4d** (32 mg, 0.30 mmol, 1.5 eq) and $(Ir[dF(CF_3)ppy]_2(dtpby))PF_6$ (2.2 mg, 2.0 µmol, 1 mol%) for 24 h. The diastereomeric ratio was determined from the crude mixture (13:1 d.r. ((^{1}H NMR)), which was purified by column chromatography (eluting with 20% EtOAc in 0.5% TEA in petroleum ether), followed by a second purification (eluting with 8% *i*PrOH in 0.5% TEA in petroleum ether), and a third purification (eluting with 20% EtOAc in 0.5% TEA in petroleum ether) to yield the title compound **6l** (51 mg, 0.15 mmol, 75% yield, 98% ee) as a colourless film.

^{1}H NMR (400 MHz, $CDCl_3$) δ 9.26 (br. s. 1H), 8.12 (d, *J* = 4.4 Hz, 1H), 7.51 (d, *J* = 7.7 Hz, 2H), 7.39 (t, *J* = 7.8 Hz, 1H), 7.29 (t, *J* = 7.5 Hz, 2H), 7.07 (t, *J* = 7.3 Hz, 1H), 6.51 (t, *J* = 6.1 Hz, 1H), 6.49 (d, *J* = 9.0 Hz, 1H), 4.76 (q, *J* = 7.21 Hz, 1H), 3.49 (d, *J* = 15.6 Hz, 1H), 3.22 (d, *J* = 11.9 Hz, 1H), 2.37-2.17 (m, 4H), 1.85-1.72 (m, 2H), 1.72-1.54 (m, 3H), 1.42-1.21 (m, 2H), 1.07 (d, *J* = 5.7 Hz, 3H). **^{13}C NMR** (126 MHz, $CDCl_3$) δ 171.1, 158.7, 147.5, 139.1 138.0, 129.1, 124.0, 120.4, 111.5, 106.5, 57.3, 42.9, 42.3, 35.2, 33.1, 30.6, 25.8, 25.1, 16.6. **HRMS** m/z: $[M + H]^+$ calc'd for $[C_{21}H_{28}N_3O]^+$ expect 338.2227; found 337.2223. **$[\alpha]_D^{25.0}$** = +113.0 (*c* 1.0, $CHCl_3$).

(2*S*,3*S*)-2-Methyl-*N*-phenyl-3-((*S*)-1-(pyridin-2-yl)azetidin-2-yl)butanamide (6m)

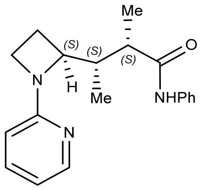

6m

Following the general procedure for the asymmetric Giese reaction using 2-(azetidin-1-yl)pyridine **1m** (1.0 mL, 0.20 mmol, 0.2 M in anhydrous $PhCF_3$, 1 eq), (*E*)-2-methyl-*N*-phenylbut-2-enamide **4m** (53 mg, 0.30 mmol, 1.5 eq) and $(Ir[dF(CF_3)ppy]_2(dtpby))PF_6$ (6.6 mg, 6.0 µmol, 3 mol%) for 24 h. The diastereomeric ratio was determined from the crude mixture (2.3:1 (**6m**:**6mb**) ^{1}H NMR), which was purified without further workup by silica gel column chromatography (eluting with 20% EtOAc in 0.5% TEA in petroleum ether) to yield the title compound **6m** as one diastereomer (26 mg, 0.084 mmol, 42% yield, 96% ee), isolated as a white solid.

^{1}H NMR (500 MHz, $CDCl_3$) δ 11.17 (br. s, 1H), 8.17 (ddd, *J* = 5.2, 2.0, 0.9 Hz, 1H), 7.63 – 7.57 (m, 2H), 7.49 (ddd, *J* = 8.6, 7.2, 2.0 Hz, 1H), 7.30 – 7.26 (m, 2H), 7.04 (tt, *J* = 7.4, 1.2 Hz, 1H), 6.65 (ddd, *J* = 7.2, 5.2, 0.9 Hz, 1H), 6.27 (dt, *J* = 8.6, 0.9 Hz, 1H), 4.82 (ddd, *J* = 9.0, 5.7, 1.3 Hz, 1H), 4.00 (ddd, *J* = 9.0, 7.7, 5.8 Hz, 1H), 3.72 (ddd, *J* = 9.7, 7.7, 5.8 Hz, 1H), 2.87 (dq, *J* = 11.3, 6.8 Hz, 1H), 2.39 (dtd, *J* = 11.6, 9.7, 5.7 Hz, 1H), 2.11 (ddt, *J* = 11.6, 9.0, 5.8 Hz, 1H), 1.82 (dqd, *J* = 11.3, 6.8, 1.3 Hz, 1H), 1.21 (d, *J* = 6.8 Hz, 3H), 1.16 (d, *J* = 6.8 Hz, 3H). **^{13}C NMR** (101 MHz, $CDCl_3$) δ 176.2, 160.6, 146.6, 139.2, 137.8, 129.0, 123.5, 119.7, 112.7, 107.0, 64.9, 49.3, 45.2, 42.3, 20.9, 15.7, 10.9. **HRMS** m/z: $[M + H]^+$ calc'd for $[C_{19}H_{24}N_3O]^+$ expect 310.1919; found 310.1916. **$[\alpha]_D^{25.0}$** = +390.1 (*c* 1.0, $CHCl_3$).

(*2S,3S*)-2-Methyl-*N*-phenyl-3-((*S*)-1-(pyridin-2-yl)pyrrolidin-2-yl)butanamide (6n)

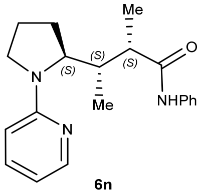

6n

Following the general procedure for the asymmetric Giese reaction using 2-(pyrrolidin-1-yl)pyridine **1a** (2 mL, 0.40 mmol, 0.2 M in anhydrous $PhCF_3$, 1 eq), (*E*)-2-methyl-*N*-phenylbut-2-enamide **4m** (105 mg, 0.60 mmol, 1.5 eq) and $(Ir[dF(CF_3)ppy]_2(dtpby))PF_6$ (6.6 mg, 6.0 µmol, 3 mol%) for 24 h. The diastereomeric ratio was determined from the crude mixture (10:1 d.r. (**6n**:others) quantitative ^{13}C NMR), which was purified by column chromatography (eluting with 20% EtOAc in 0.5% TEA in petroleum ether) to yield the title compound **6n** (104 mg, 0.32 mmol, 80% yield, 99% ee) as a white solid.

^{1}H NMR (700 MHz, $CDCl_3$) δ 10.7 (br. s, 1 H), 8.17 (dd, *J* = 4.8, 1.4 Hz, 1 H), 7.58 (d, *J* = 7.21 Hz, 2 H), 7.47 (br. t, *J* = 6.93 Hz, 1 H), 7.28 (t, *J* = 8.0 Hz, 2 H), 7.04 (t, *J* = 7.4 Hz, 1 H), 6.63 (t, *J* = 6.2 Hz, 1 H), 6.43 (d, *J* = 8.3 Hz, 1 H), 4.64 (br. s, 1 H), 3.46 (q, *J* = 7.8 Hz, 1 H), 3.37 (q, *J* = 8.0 Hz, 1 H), 2.79 (br. s, 1 H), 2.15-2.05 (m, 2 H), 2.04-1.98 (m, 1 H), 1.92-1.84 (m, 1 H), 1.82-1.74 (m, 1 H), 1.12 (d, *J* = 6.8 Hz, 3 H), 0.90 (d, *J* = 6.9 Hz, 1 H). **^{13}C NMR** (176 MHz, $CDCl_3$) δ 176.0, 158.3, 146.2, 139.2, 137.9, 128.9, 123.4, 119.6, 112.2, 108.7, 59.3, 50.6, 44.7, 44.2, 32.0, 24.6, 15.8, 12.7. **HRMS** m/z: $[M + H]^+$ calc'd for $[C_{20}H_{26}N_3O]^+$ expect 324.2070; found 324.2066. **$[\alpha]_D^{25.0}$** = +289.1 (*c* 1.0, $CHCl_3$).

X-Ray Crystallography Sample preparation

The purified sample obtained from above was crystallized via vapour diffusion (Et_2O / DCM) to yield colourless crystals which were analysed by X-ray diffraction. The relative configuration was determined to be *S,S,S* or *R,R,R*. By analogy with the hydrochloride salt of (*S*)-*N*-Phenyl-2-(((*S*)-1-(pyridin-2-yl)pyrrolidin-2-yl)methyl)butanamide (**6n**) the absolute stereochemistry was assigned as *S,S,S*. The structure was deposited in the Cambridge Crystallographic Data Centre (deposition no.: CCDC 2183884).

(*2S,3S*)-2-Methyl-*N*-phenyl-3-((*S*)-1-(pyridin-2-yl)azepan-2-yl)butanamide (6o)

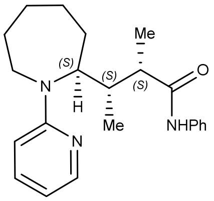

6o

Following the general procedure for the asymmetric Giese reaction using 1-(pyridin-2-yl)azepane **1g** (2 mL, 0.40 mmol, 0.2 M in anhydrous $PhCF_3$, 1 eq), (*E*)-2-methyl-*N*-phenylbut-2-enamide **4m** (105 mg, 0.60 mmol, 1.5 eq) and $(Ir[dF(CF_3)ppy]_2(dtpby))PF_6$ (6.6 mg, 6.0 µmol, 3 mol%) for 24 h. The diastereomeric ratio was determined from the crude mixture (20:1 (others) d.r. (^{13}C NMR and ^{1}H NMR)), which was purified by column chromatography (eluting with 20% EtOAc in 0.5% TEA in petroleum ether) followed by the general workup procedure for the impure isolated fractions to yield the title compound **6o** (112 mg, 0.32 mmol, 80% yield, 99% ee) as a white solid.

^{1}H NMR (700 MHz, $CDCl_3$) δ 10.8 (br. s, 1H), 8.19 (dd, *J* = 4.8, 1.3 Hz, 1H), 7.68 (d, *J* = 8.0 Hz, 1H), 7.48 (ddd, *J* = 8.7, 7.0, 1.8 Hz, 1H), 7.33 (t, *J* = 7.9 Hz, 2H), 7.08 (t, *J* = 7.4 Hz, 1H), 6.61 (d, *J* = 8.8 Hz, 1H), 6.59 (dd, *J* = 7.4, 4.6 Hz, 1H), 4.90 (d, *J* = 11.2, 5.6 Hz, 1H), 3.62 (d, *J* = 15.8 Hz, 1H), 3.30 (dd, *J* = 15.4, 11.7 Hz, 1H), 2.64 (dq, *J* = 9.4, 7.0 Hz, 1H), 2.01 – 1.95 (m, 1H), 1.95 – 1.90 (m, 1H), 1.79 – 1.72 (m, 2H), 1.72 – 1.65 (m, 1H), 1.61 – 1.54 (m, 2H), 1.32 – 1.24 (m, 1H), 1.09 (d, *J* = 6.9 Hz, 3H), 1.07 – 1.03 (m, 1H), 0.94 (d, *J* = 7.1 Hz, 3H). **^{13}C NMR** (176 MHz, $CDCl_3$) δ 176.2, 158.9, 146.6, 139.3, 137.3, 129.0, 123.6, 120.1, 120.0, 111.4, 107.0, 56.8, 44.5, 43.3, 42.7, 34.6, 30.0, 26.2, 25.9, 14.7, 12.1. **HRMS** m/z: $[M + H]^+$ calc'd for $[C_{22}H_{30}N_3O]^+$ expect 352.2383; found 352.2386. **$[\alpha]_D^{25.0}$** = +206.3 (*c* 1.0, $CHCl_3$).

(5*S*,6a*S*)-5-Benzyl-*N*-phenyl-5,6,6a,7,8,9-hexahydropyrrolo[1,2-a][1,5]naphthyridine-5-carboxamide (7aa) and (5*R*,6a*S*)-5-benzyl-*N*-phenyl-5,6,6a,7,8,9-hexahydropyrrolo[1,2-a][1,5]naphthyridine-5-carboxamide (7ab)

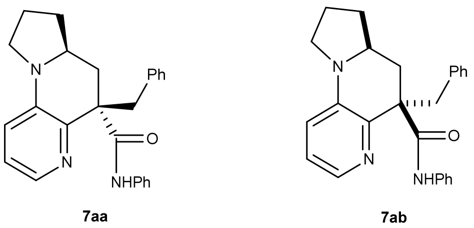

Following the general procedure for the asymmetric Giese reaction using 3-(pyrrolidin-1-yl)pyridine **1n** (1.0 mL, 0.20 mmol, 0.2 M in anhydrous $PhCF_3$, 1 eq), 2-benzyl-*N*-phenylacrylamide **4b** (71 mg, 0.30 mmol, 1.5 eq) and $(Ir[dF(CF_3)ppy]_2(dtpby))PF_6$ (2.2 mg, 2.0 µmol, 1 mol%) for 8 h. The diastereomeric ratio was determined from the crude mixture (1:1.3 d.r. (^1H NMR)) and was purified by column chromatography (eluting with 5% EtOAc in 0.5% TEA in petroleum ether) and further preparatory TLC (eluting with 5% EtOAc in 0.5% TEA in petroleum ether) to yield **7aa** as the first eluting diastereomer (9.0mg, 0.023 mmol, 12% yield, racemic) and **7ab** as the second eluting diastereomer (11 mg, 0.029 mmol, 14% yield, 25% ee). Combined yield: 20 mg, 0.052 mmol, 26%.

Diastereomer 1 (**7aa**):

^{1}H NMR (700 MHz, $CDCl_3$) δ 9.87 (br. s, 1H), 8.04 (ddd, *J* = 4.7, 1.0 Hz, 1H), 7.53 (d, *J* = 7.6 Hz, 2H), 7.30 (t, *J* = 8.8 Hz, 2H), 7.20 – 7.15 (m, 3H), 7.11 – 7.08 (m, 1H), 7.07 – 7.00 (m, 3H), 6.67 (d, *J* = 7.8 Hz, 1H), 3.62 (d, *J* = 13.4 Hz, 1H), 3.46 (d, *J* = 13.5 Hz, 1H), 3.37 – 3.29 (m, 1H), 3.21 (q, *J* = 8.7 Hz, 1H), 3.18 – 3.14 (m, 1H), 2.81 (d, *J* = 12.2 Hz, 1H), 2.18 – 2.12 (m, 1H), 2.07 – 2.01 (m, 1H), 1.92 – 1.83 (m, 1H), 1.47 – 1.40 (m, 1H), 1.15 (dd, *J* = 12.6, 11.7 Hz, 1H). **^{13}C NMR** (126 MHz, $CDCl_3$) δ 172.1, 142.1, 131.4, 138.7, 135.0, 130.4, 129.0, 128.2, 126.7, 123.8, 123.6, 119.5, 117.3, 54.5, 51.8, 46.7, 46.1, 45.9, 33.0, 23.8. **HRMS** m/z: $[M + H]^+$ calc'd for $[C_{25}H_{26}N_3O]^+$ expect 384.2070; found 384.2077. **$[\alpha]_D^{25.0}$** = –18.1 (*c* 1.0, $CHCl_3$).

Diastereomer 2 (**7ab**):

^{1}H NMR (500 MHz, $CDCl_3$) δ 12.26 (br. s, 1H), 7.81 (dd, *J* = 4.6, 1.4 Hz, 1H), 7.53 – 7.50 (m, 2H), 7.32 – 7.28 (m, 2H), 7.19 – 7.13 (m, 3H), 7.07 (dd, *J* = 8.2, 4.6 Hz, 1H), 7.07 – 7.04 (m, 1H), 6.93 – 6.90 (m, 2H), 6.73 (dd, *J* = 8.2, 1.4 Hz, 1H), 3.49 (dddd, *J* = 12.1, 10.3, 4.7, 3.0 Hz, 1H), 3.31 (dd, *J* = 15.4, 13.3 Hz, 2H), 3.29 – 3.22 (m, 2H), 2.70 (dd, *J* = 14.0, 3.0 Hz, 1H), 2.21 – 2.10 (m, 2H), 2.03 – 1.92 (m, 1H), 1.89 (dd, *J* = 14.0, 12.0 Hz, 1H), 1.58 -1.49 (m, 2H, partly under water peak), 0.91 – 0.77 (m, 1H). **^{13}C NMR** (126 MHz, $CDCl_3$) δ 172.2, 143.0, 141.7, 139.1, 137.0, 130.7, 129.1, 128.2, 127.1, 123.8, 120.5, 117.7, 53.8, 50.1, 48.5, 47.1, 34.3, 33.7, 24.1. **HRMS** m/z: $[M + H]^+$ calc'd for $[C_{25}H_{26}N_3O]^+$ expect 384.2070; found 384.2082. **$[\alpha]_D^{25.0}$** = –6.3 (*c* 0.4, $CHCl_3$).

Deprotection and cyclisation of 5ba and 5bb

(*S*)-2-Benzyl-*N*-phenyl-3-((*S*)-pyrrolidin-2-yl)propenamide (5bg)

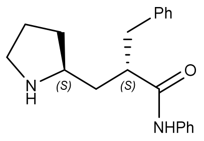

5bg

(*S*)-2-Benzyl-*N*-phenyl-3-((S)-1-(pyridin-2-yl)pyrrolidin-2-yl)propanamide (**5ba**) (40 mg, 0.10 mmol, 1 eq) and K_3PO_4 (6.6 mg, 0.031 mmol, 0.3 eq) in anhydrous DCM (1 mL) were measured in an oven-dried vial. MeOTf (17 µL, 0.12 mmol, 1.2 eq) was added at 0 °C and the reaction was allowed to warm to ambient temperature and was further stirred overnight. The reaction mixture was concentrated to dryness *in vacuo*. MeOH (3 mL) was added to the crude mixture and $NaBH_4$ (39 mg, 1.0 mmol, 10 eq) was added portionwise to the mixture at 0 °C. The reaction was further stirred for 1 h at 0 °C and 3 hours at ambient temperature. The reaction mixture was quenched by dropwise addition of water and acidified by addition of 3 M HCl at 0 °C, stirred for 15 minutes and basified at 0 °C by addition of K_3PO_4. The mixture was diluted with water and extracted with DCM. The combined organic layers were dried over $MgSO_4$, filtered and concentrated *in vacuo*. The crude product was purified by silica gel chromatography (eluting with 0 to 5% MeOH in DCM) to afford the title compound **5bg** (27 mg, 0.087 mmol, 84% yield).

^{1}H NMR (400 MHz, $CDCl_3$) δ 9.14 (br. s, 1H), 7.47 (m, 2H), 7.30 – 7.15 (m, 7H), 7.05 (t, *J* = 7.4 Hz, 1H), 3.15 (dd, *J* = 13.4, 7.5 Hz, 1H), 3.06 – 2.97 (m, 1H), 2.95 (ddd, *J* = 11.8, 7.4, 5.6 Hz, 1H), 2.87 – 2.76 (m, 1H), 2.72 (dd, *J* = 13.3, 7.0 Hz, 1H), 2.53 (br. s, 1H), 2.03 (ddd, *J* = 14.0, 11.3, 3.7 Hz, 1H), 1.90 – 1.80 (m, 1H), 1.78 – 1.61 (m, 2H), 1.48 (ddd, *J* = 14.3, 10.1, 3.6 Hz, 1H), 1.22 – 1.10 (m, 1H). **^{13}C NMR** (101 MHz, $CDCl_3$) δ 173.4, 140.0, 138.6, 129.2, 129.0, 128.6, 126.4, 123.9, 119.7, 57.8, 47.6, 46.6, 38.9, 37.8, 32.5, 25.8. **HRMS** m/z: $[M + H]^+$ calc'd for $[C_{20}H_{25}N_2O]^+$ expect 309.1961; found 309.1966. **$[\alpha]_D^{25.0}$** = –21.1 (*c* 1.0, $CHCl_3$).

(*S*)-2-((*S*)-2-Carboxy-3-phenylpropyl)pyrrolidin-1-ium chloride (5bc)

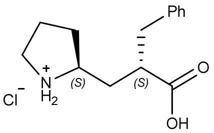

5bc

(*S*)-2-Benzyl-*N*-phenyl-3-((*S*)-pyrrolidin-2-yl)propenamide **5bg** (15 mg, 0.049 mmol, 1 eq) and concentrated HCl (1 mL) were added to a microwave vial. The vial was sealed and heated at 100 °C overnight (NOTE: pressure formation). The reaction mixture was cooled to 0 °C and the mixture was basified with aqueous 10% w/w NaOH solution. The aqueous mixture was extracted with Et_2O and acidified (pH 1) with concentrated HCl. The acidified mixture was extracted with mixture of *i*PrOH in chloroform (1:3). The combined organic layers were dried over $MgSO_4$, filtered and concentrated *in vacuo* to afford the tittle compound **5bc** (13 mg, 0.049 mmol, quantitative yield)

^{1}H NMR (500 MHz, D_2O) δ 7.40 – 7.34 (m, 2H), 7.33 – 7.30 (m, 1H), 7.29 – 7.25 (m, 2H), 3.57 (dtd, *J* = 8.4, 7.6, 7.2 Hz, 1H), 3.57 (ddd, *J* = 8.6, 6.2, 2.6 Hz, 2H), 2.94 (d, *J* = 7.3 Hz, 2H), 2.90 – 2.80 (m, 1H), 2.20 (dddd, *J* = 13.0, 7.2, 7.2, 3.7 Hz, 1H), 2.11 – 2.02 (m, 2H), 2.01 – 1.93 (m, 2H), 1.63 (ddt, *J* = 13.1, 9.2, 9.0 Hz, 1H). **^{13}C NMR** (126 MHz, D_2O) δ 178.6, 138.2, 129.0, 128.7, 126.9, 58.8, 45.1, 44.8, 37.8, 33.3, 29.9, 23.0. **HRMS** m/z: $[M + H]^+$ calc'd for $[C_{20}H_{25}N_2O]^+$ expect 309.1961; found 309.1966. **$[\alpha]_D^{25.0}$** = –113.3 (*c* 1.0, H_2O).

(2*S*,7a*S*)-2-Benzylhexahydro-3*H*-pyrrolizin-3-one (5be)

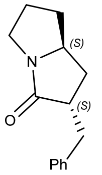

5be

(*S*)-2-((*S*)-2-Carboxy-3-phenylpropyl)pyrrolidin-1-ium chloride **5bc** (13 mg, 0.049 mmol, 1 eq), Et_3N (9 µL, 0.063 mmol, 1.3 eq), DMAP (0.6 mg, 0.006 mmol, 0.1 eq) and anhydrous DCM were measured in an oven-dried vial. EDC·HCl (11 mg, 0.058 mmol, 1.2 eq) was added to the mixture at 0 °C and the mixture was allowed to warm up to ambient temperature and further stirred for 4 h. Water was added to the reaction and the mixture was extracted with DCM, dried over $MgSO_4$, filtered and concentrated *in vacuo*. The crude mixture was purified by silica gel chromatography (eluting with 50% EtOAc in petroleum ether) to afford the title product **5be** (4.5 mg, 0.021 mmol, 43% yield, 90% ee).

^{1}H NMR (500 MHz, $CDCl_3$) δ 7.31 – 7.26 (m, 2H), 7.23 – 7.19 (m, 3H), 3.62 – 3.53 (m, 2H), 3.11 (dd, *J* = 13.5, 4.1 Hz, 1H), 3.03 (ddd, *J* = 11.8, 9.1, 3.5 Hz, 1H), 2.90 (dddd, *J* = 9.4, 9.4, 4.1, 2.7 Hz, 1H), 2.81 (dd, *J* = 13.5, 9.7 Hz, 1H), 2.13 – 2.04 (m, 1H), 2.05 (ddd, *J* = 13.2, 7.0, 2.6 Hz, 1H), 1.98 – 1.89 (m, 2H), 1.77 (ddd, *J* = 13.2, 9.1, 6.9 Hz, 1H), 1.27 – 1.17 (m, 1H). **^{13}C NMR** (126 MHz, $CDCl_3$) δ 176.9, 139.6, 129.4, 128.7, 126.7, 60.6, 49.0, 41.4, 37.8, 32.4, 31.1, 27.0. **HRMS** m/z: $[M + H]^+$ calc'd for $[C_{14}H_{17}NO]^+$ expect 216.1383; found 216.1381. **$[\alpha]_D^{25.0}$** = +50.6 (*c* 0.09, $CHCl_3$).

The data for the relative stereochemistry are not in accordance with the reported literature data – see earlier discussion.[4,5]

(*R*)-2-Benzyl-*N*-phenyl-3-((*S*)-pyrrolidin-2-yl)propenamide (5bh)

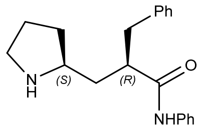

5bh

(*R*)-2-Benzyl-*N*-phenyl-3-((*S*)-1-(pyridin-2-yl)pyrrolidin-2-yl)propanamide (**5bb**) (94 mg, 0.24 mmol, 1 eq) and K_3PO_4 (16 mg, 0.073 mmol, 0.3 eq) in dry DCM (2 mL) were measured in an oven-dried vial. MeOTf (40 µL, 0.37 mmol, 1.5 eq) was added at 0 °C and the reaction was allowed to warm to ambient temperature and was further stirred overnight. The reaction mixture was concentrated to dryness *in vacuo*. MeOH (4 mL) was added to the crude and $NaBH_4$ (92 mg, 2.4 mmol, 10 eq) was added portionwise to the mixture at 0 °C. The reaction was further stirred for 1 h at 0 °C and 3 hours at ambient temperature. The reaction mixture was quenched by dropwise addition of water and acidified by addition of 3 M HCl at 0 °C, stirred for 15 minutes and basified at 0 °C by addition of K_3PO_4. The mixture was diluted with water and extracted with DCM. The combined organic layers were dried over $MgSO_4$, filtered and concentrated *in vacuo*. The crude product was purified by silica gel chromatography (eluting with 0 to 10% MeOH in DCM) to afford the title compound **5bh** (74 mg, 0.24 mmol, 98% yield).

^{1}H NMR (400 MHz, $CDCl_3$) δ 9.72 (br. s, 1H), 7.53 – 7.39 (m, 2H), 7.29 – 7.21 (m, 6H), 7.20 – 7.14 (m, 1H), 7.04 (tt, *J* = 7.4, 1.0 Hz, 1H), 3.24 – 3.08 (m, 2H), 3.00 – 2.85 (m, 2H), 2.78 – 2.69 (m, 2H), 1.93 – 1.74 (m, 3H), 1.71 – 1.61 (m, 2H), 1.36 – 1.24 (m, 1H). **^{13}C NMR** (126 MHz, $CDCl_3$) δ 173.4, 139.1, 138.4, 129.7, 129.1, 128.9, 127.0, 124.7, 120.6, 59.3, 47.4, 45.6, 38.2, 35.8, 32.0, 24.3. **HRMS** m/z: $[M + H]^+$ calc'd for $[C_{20}H_{25}N_2O]^+$ expect 309.1961; found 309.1963. **$[\alpha]_D^{25.0}$** = –19.3 (*c* 1.0, $CHCl_3$).

(*S*)-2-((*R*)-2-Carboxy-3-phenylpropyl)pyrrolidin-1-ium chloride (5bd)

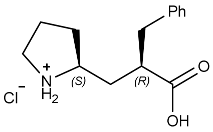

5bd

(*R*)-2-Benzyl-*N*-phenyl-3-((*S*)-pyrrolidin-2-yl)propenamide **5bh** (20 mg, 0.065 mmol, 1 eq) and concentrated HCl (1 mL) was added in 10 mL microwave vial. The vial was sealed and heated at 100 °C for overnight (NOTE: pressure formation). The reaction mixture was cooled to 0 °C and the mixture was basified with aqueous 10% w/w NaOH solution. The aqueous mixture was extracted with Et_2O, organic phase was separated and the aqueous phase was acidified (pH 1) with concentrated HCl. The acidified mixture was extracted with excess of mixture of *i*PrOH in chloroform (1:3). The combined organic layers were dried over $MgSO_4$, filtered and concentrated *in vacuo* to afford the tittle compound **5bd** (14 mg, 0.052 mmol, 80% yield)

^{1}H NMR (500 MHz, D_2O) δ 7.38 (t, *J* = 7.6 Hz, 2H), 7.31 (t, *J* = 6.9 Hz, 1H), 7.28 (d, *J* = 7.6 Hz, 2H), 3.54 (ddt, *J* = 8.9, 8.9, 6.4 Hz, 1H), 3.32 – 3.26 (m, 2H), 2.96 (dd, *J* = 13.7, 8.2 Hz, 1H), 2.92 (dd, *J* = 13.7, 6.5 Hz, 1H), 2.84 – 2.76 (m, 1H), 2.27 – 2.19 (m, 1H), 2.14 (ddd, *J* = 13.8, 10.3, 5.3 Hz, 1H), 2.08 – 1.99 (m, 1H), 2.00 – 1.91 (m, 1H), 1.82 (ddd, *J* = 13.6, 9.3, 4.3 Hz, 1H), 1.61 (dtd, *J* = 13.1, 9.0, 9.0 Hz, 1H). **^{13}C NMR** (126 MHz, D_2O) δ 179.1, 138.5, 129.0, 128.7, 126.8, 58.7, 45.4, 45.0, 38.1, 33.3, 29.4, 22.8. **HRMS** m/z: $[M + H]^+$ calc'd for $[C_{20}H_{25}N_2O]^+$ expect 309.1961; found 309.1966. **$[\alpha]_D^{25.0}$** = –12.5 (*c* 0.35, $CHCl_3$).

(2*R*,7a*S*)-2-Benzylhexahydro-3*H*-pyrrolizin-3-one (5bf)

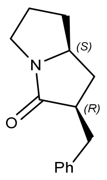

5bf

(*S*)-2-((*S*)-2-Carboxy-3-phenylpropyl)pyrrolidin-1-ium chloride **5bd** (8.8 mg, 0.033 mmol, 1 eq), Et_3N (6 µL, 0.042 mmol, 1.3 eq), DMAP (0.4 mg, 0.003 mmol, 0.1 eq) and anhydrous DCM were measured in an oven-dried vial. EDC·HCl (7.5 mg, 0.039 mmol, 1.2 eq) was added to the mixture at 0 °C and the mixture was allowed to warm up to ambient temperature and further stirred for 4 h. Water was added to the reaction and the mixture was extracted with DCM, dried over $MgSO_4$, filtered and concentrated *in vacuo*. The crude mixture was purified by silica gel chromatography (eluting with 50% EtOAc in petroleum ether) to afford the title product **5bf** (4.3 mg, 0.020 mmol, 60% yield, 94% ee).

^{1}H NMR (500 MHz, $CDCl_3$) δ 7.30 – 7.25 (m, 2H), 7.22 – 7.16 (m, 3H), 3.72 (tt, *J* = 8.8, 6.0 Hz, 1H), 3.55 (dt, *J* = 11.5, 7.7 Hz, 1H), 3.32 (dd, *J* = 14.0, 3.9 Hz, 1H), 3.12 – 3.00 (m, 2H), 2.56 (dd, *J* = 13.9, 10.3 Hz, 1H), 2.30 (ddd, *J* = 12.4, 7.6, 6.3 Hz, 1H), 2.13 – 2.04 (m, 1H), 2.03 – 1.96 (m, 2H), 1.38 (ddd, *J* = 12.0, 12.0, 8.6 Hz, 1H), 1.23 – 1.14 (m, 1H). **^{13}C NMR** (126 MHz, $CDCl_3$) δ 175.2, 140.3, 129.2, 128.7, 126.5, 59.8, 48.7, 41.4, 37.4, 35.4, 32.6, 27.1. **HRMS** m/z: $[M + H]^+$ calc'd for $[C_{14}H_{17}NO]^+$ expect 216.1383; found 216.1380. **$[\alpha]_D^{25.0}$** = –61.2 (*c* 0.29, $CHCl_3$).

The data for the relative stereochemistry are not in accordance with the reported literature – see earlier discussion. [4,5]

Formal synthesis of (–)-pseudoheliotridane

(*R*)-*N*-Phenyl-3-((*S*)-1-(pyridin-2-yl)pyrrolidin-2-yl)butanamide (6q)

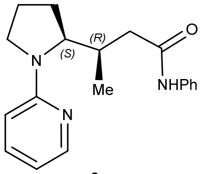

6q

Following the general procedure for the asymmetric Giese reaction using 2-(pyrrolidin-1-yl)pyridine **1a** (1.0 mL, 0.20 mmol, 0.2 M in anhydrous $PhCF_3$, 1 eq), (*E*)-*N*-phenylbut-2-enamide **4d** (32 mg, 0.30 mmol, 1.5 eq) and $(Ir[dF(CF_3)ppy]_2(dtpby))PF_6$ (2.2 mg, 2.0 µmol, 1 mol%) for 24 h. The diastereomeric ratio was determined from the crude mixture (5.0:1 d.r. (^{1}H NMR)) and was purified by column chromatography twice (eluting with 20% EtOAc in 0.5% TEA in petroleum ether) to yield the title compound **6q** (28 mg, 0.90 mmol, 45% yield, 90% ee) as a colourless film.

^{1}H NMR (400 MHz, $CDCl_3$) δ 10.01 (br. s. 1H), 8.08 (dd, *J* = 5.1, 1.3 Hz, 1H), 7.58 (d, *J* = 7.8 Hz, 2H), 7.47 (ddd, *J* = 8.6, 7.1, 1.7 Hz, 1H), 7.31 (t, *J* = 7.9 Hz, 2H), 7.09 (t, *J* = 7.4 Hz, 1H), 6.57 (dd, *J* = 6.5, 5.6 Hz, 1H), 6.45 (d, *J* = 8.6 Hz, 1H), 4.44 (ddd, *J* = 7.8, 5.9, 2.0 Hz, 1H), 3.50 (ddd, *J* = 9.4, 8.3, 2.9 Hz, 1H), 3.28 (q, *J* = 8.7 Hz, 1H), 2.64 (sept, *J* = 6.6 Hz, 1H), 2.43 (d, *J* = 6.6 Hz, 2H), 2.15 – 1.80 (m, 4H), 0.97 (d, *J* = 7.0 Hz, 1 H). **^{13}C NMR** (126 MHz, $CDCl_3$) δ 171.0, 158.0, 147.3, 139.0, 137.8, 128.9, 124.1, 121.1, 112.0, 107.7, 60.7, 48.5, 42.0, 31.9, 26.7, 24.2, 15.7. **HRMS** m/z: $[M + H]^+$ calc'd for $[C_{19}H_{24}N_3O]^+$ expect 310.1914; found 310.1913. **$[\alpha]_D^{25.0}$** = +82.3 (*c* 1.0, $CHCl_3$).

(*R*)-*N*-Phenyl-3-((*S*)-pyrrolidin-2-yl)butanamide (6qa)

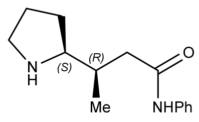

6qa

(*R*)-*N*-Phenyl-3-((*S*)-1-(pyridin-2-yl)pyrrolidin-2-yl)butanamide **6q** (73 mg, 0.24 mmol, 1 eq) and K_3PO_4 (5 mg, 0.024 mmol, 0.1 eq) in anhydrous DCM (3 mL) were measured in an oven-dried vial. MeOTf (28 µL, 0.26 mmol, 1.1 eq) was added at ambient temperature, heated to 40 °C, and was further stirred for overnight. The reaction mixture was concentrated to dryness *in vacuo*. MeOH (4 mL) was added to the crude and $NaBH_4$ (89 mg, 2.4 mmol, 10 eq) was added portionwise to the mixture at 0 °C. The reaction was further stirred for 4 h at 0 °C. The reaction mixture was diluted with water and extracted with DCM. Combined organic layers were dried over $MgSO_4$, filtered and concentrated *in vacuo*. The crude product was purified by silica gel chromatography (eluting with 5 to 10% MeOH in DCM). The collected fractions collected fractions were dissolved in MeOH and the mixture was acidified by addition of HCl 3 M. The mixture was concentrated *in vacuo*. The material was dissolved in water and basified with Na_2CO_3 and the aqueous mixture was extracted with DCM, dried over $MgSO_4$ and concentrated *in vacuo* to afford the title compound **6qa** (37 mg, 0.16 mmol, 68% yield).

^{1}H NMR (500 MHz, $CDCl_3$) δ 7.68 – 7.63 (m, 2H), 7.29 – 7.26 (m, 2H), 7.07 (tt, *J* = 7.3, 1.1 Hz, 1H), 3.37 – 3.29 (m, 1H), 3.23 (ddd, *J* = 11.1, 9.7, 7.6 Hz, 1H), 2.97 (dd, *J* = 14.6, 5.3 Hz, 1H), 2.47 (dd, *J* = 14.4, 6.9 Hz, 1H), 2.42 – 2.33 (m, 1H), 2.19 (dtd, *J* = 13.3, 6.6, 2.8 Hz, 1H), 2.10 – 2.02 (m, 1H), 1.98 – 1.87 (m, 1H), 1.67 (dtd, *J* = 12.9, 10.3, 7.7 Hz, 1H), 1.10 (d, *J* = 6.8 Hz, 3H). **^{13}C NMR** (126 MHz, $CDCl_3$) δ 171.4, 139.1, 129.0, 123.6, 119.5, 65.2, 46.5, 43.8, 36.7, 31.2, 26.4, 19.1. **HRMS** m/z: $[M + H]^+$ calc'd for $[C_{14}H_{21}N_2O]^+$ expect 233.1648; found 233.1648. **$[\alpha]_D^{25.0}$** = –21.2 (*c* 1.0, $CHCl_3$).

(1*R*,7a*S*)-1-Methylhexahydro-3H-pyrrolizin-3-one (6qb)

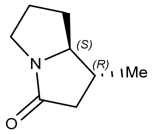

6qb

(*R*)-*N*-Phenyl-3-((*S*)-pyrrolidin-2-yl)butanamide **6qa** (33 mg, 0.14 mmol, 1 eq) and K_3PO_4 (148 mg, 0.70 mmol, 5 eq) were measured in a crimp top vial with water (0.5 mL) and 1,4-dioxane (0.5 mL). The reaction mixture was stirred overnight at 80 °C, cooled down to ambient temperature, diluted with water and extracted with DCM. The combined organic phases were dried over $MgSO_4$, filtered and concentrated *in vacuo*. The crude mixture was purified by silica gel chromatography (eluting with 50 to 80% EtOAc in petroleum ether) to afford the title compound **6qb** (12 mg, 0.090 mmol, 64% yield, 90% ee) as a colourless film.

^{1}H NMR (700 MHz, $CDCl_3$) δ 3.54 (dt, *J* = 11.5, 7.6 Hz, 1H), 3.48 (ddd, *J* = 7.9, 7.9, 6.4 Hz, 1H), 3.03 (dddd, *J* = 11.4, 8.9, 3.7, 1.2 Hz, 1H), 2.53 (dd, *J* = 16.0, 8.1 Hz, 1H), 2.39 (dd, *J* = 15.9, 11.3 Hz, 1H), 2.17 – 2.10 (m, 1H), 2.10 – 1.96 (m, 3H), 1.40 – 1.34 (m, 1H), 1.14 (d, *J* = 6.6 Hz, 3H). **^{13}C NMR** (101 MHz, $CDCl_3$) δ 174.5, 69.1, 44.0, 41.5, 38.1, 30.9, 27.1, 18.1. **HRMS** m/z: $[M + H]^+$ calc'd for $[C_8H_{14}NO]^+$ expect 140.1070; found 140.1068. **$[\alpha]_D^{25.0}$** = –49.6 (*c* 0.37, $CHCl_3$) (lit.[7] –63.5 (*c* 1.0, $CHCl_3$)).

The spectral data are in good agreement with the previous literature.[7]

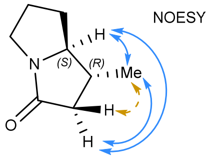

Synthesis of *ent*-pregabalin

(*R*)-3-((Benzyl(pyridin-2-yl)amino)methyl)-5-methyl-*N*-phenylhexanamide (6p)

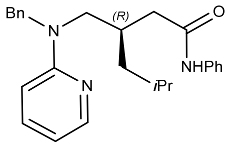

6p

Following the general procedure for the asymmetric Giese reaction using *N*-benzyl-*N*-methylpyridin-2-amine **1c** (1.0 mL, 0.20 mmol, 0.2 M in anhydrous $PhCF_3$, 1 eq), (*E*)-5-methyl-*N*-phenylhex-2-enamide **4c** (61 mg, 0.30 mmol, 1.5 eq) and (Ir[dF(CF_3)ppy]$_2$(dtpby))PF_6 (22 mg, 0.020 mmol, 10 mol%) for 24 h. The crude mixture was purified by column chromatography (eluting with 15% EtOAc in 0.5% TEA in petroleum ether) to yield the title compound **6p** (32 mg, 0.080 mmol, 40% yield, 99% ee) as a colourless viscous oil.

^{1}H NMR (700 MHz, $CDCl_3$) δ 10.5 (br. s, 1H), 8.18 (dd, *J* = 1.3, 5.2 Hz, 1 H), 7.63 (d, *J* = 8.00 Hz, 2 H), 7.36 (ddd, *J* = 1.8, 7.0, 8.7 Hz, 1 H), 7.35-7.31 (m, 4 H), 7.26 (t, *J* = 7.3 Hz, 1 H), 7.19 (d, *J* = 7.5 Hz, 2 H), 7.09 (t, *J* = 7.4 Hz, 1 H), 6.59 (dd, *J* = 5.4, 6.7 Hz, 1 H), 6.43 (d, *J* = 8.7 Hz, 1 H), 4.66 (dd, *J* = 17.4, 28.4 Hz, 2 H), 4.43 (dd, *J* = 9.3, 14.6 Hz, 1 H), 3.17 (dd, *J* = 3.3, 14.6 Hz, 1 H), 2.60 (d, *J* = 11.1 Hz, 1 H), 2.39-2.31 (m, 2 H) 1.80 (sept, *J* = 6.7 Hz, 1 H), 1.39 (td, *J* = 6.9, 13.8 Hz, 1 H), 1.15 (td, *J* = 6.8, 13.7 Hz, 1 H), 0.90 (d, *J* = 6.6 Hz, 1 H), 0.87 (d, *J* = 6.6 Hz, 1 H). **^{13}C NMR** (176 MHz, $CDCl_3$) δ 170.9, 159.0, 147.0, 139.4, 138.4, 137.4, 129.2, 129.1, 127.5, 126.6, 124.0, 120.4, 112.7, 107.4, 52.7, 52.6, 41.2, 39.7, 33.3, 25.5, 23.4, 22.8. **HRMS** m/z: $[M + H]^+$ calc'd for $[C_{26}H_{32}N_3O]^+$ expect 402.2540; found 402.2537. **$[\alpha]_D^{25.0}$** = +124.5.2 (*c* 1.0, $CHCl_3$).

(*R*)-3-(Aminomethyl)-5-methylhexanoic acid hydrochloride ((*R*)-pregabalin HCl salt) (6pa)

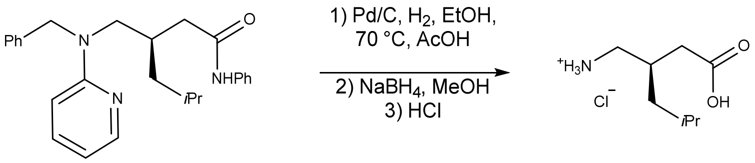

(*R*)-3-((Benzyl(pyridin-2-yl)amino)methyl)-5-methyl-*N*-phenylhexanamide **6p** (25 mg, 0.062 mmol, 1 eq), palladium on carbon 10% w/w (13 mg, 0.013 mmol, 0.2 eq), EtOH (0.5 – 1 mL) and acetic acid (19 µL, 0.31 mmol, 5 eq) were measured into a 10 mL, oven-dried microwave vial. The vial was sealed and evacuated and backfilled with hydrogen 3 times. A balloon of hydrogen was placed on the vial and the reaction mixture was stirred at 70 °C overnight. The reaction mixture was allowed to cool down to ambient temperature and the mixture was filtered through small pad of celite. The mixture was concentrated dry *in vacuo*. The dry mixture was dissolved in methanol (1 – 2 mL) and $NaBH_4$ (24 mg, 0.62 mmol, 10 eq) was added portionwise at 0 °C. The reaction mixture was slowly let to warm to ambient temperature and further stirred for 4-5 hours. The reaction mixture was concentrated to dryness *in vacuo*. The crude material was dissolved in concentrated HCl (1 mL) and heated in sealed 10 mL microwave vial at 100 °C overnight (NOTE: pressure formation). The reaction mixture was cooled to ambient temperature and basified with 10% w/w aqueous NaOH at 0 °C. The basic crude mixture was extracted with Et_2O and acidified with concentrated HCl. The aqueous mixture was

extracted with excess of DCM, dried over $MgSO_4$, filtered, and concentrated *in vacuo*. To remove possible remaining traces of boronic acid, MeOH (10 mL) was added and evaporated to yield the title product **6pa** (10.9 mg, 0.056 mmol, 90% yield). The cyclised (*R*)-4-isobutylpyrrolidin-2-one **6pb** (99% ee) was used for the determination of enantioselectivity by chiral SFC.

^{1}H NMR (500 MHz, MeOD : D_2O) δ 3.02 – 2.92 (m, 2H), 2.43 (ddd, *J* = 17.5, 16.7, 6.2 Hz, 2H), 2.22 (sept, *J* = 6.6 Hz, 1H), 1.69 (sept, *J* = 6.7 Hz, 1H), 1.27 (t, *J* = 7.2 Hz, 2H), 0.95 (d, *J* = 6.7 Hz, 3H), 0.93 (d, *J* = 6.7 Hz, 3H);). **^{13}C NMR** (126 MHz, $CDCl_3$) δ 175.9, 44.5, 42.0, 37.2, 32.6, 26.1, 23.1, 22.5. **$[\alpha]_D^{25.0}$** = –6.9 (*c* 1.1, H_2O) (lit.[8] for (*S*)-Pregabalin hydrochloride. **$[\alpha]_D^{25.0}$** = 7.0 (*c* 1.1, H_2O).

(*R*)-4-Isobutylpyrrolidin-2-one (6pb)

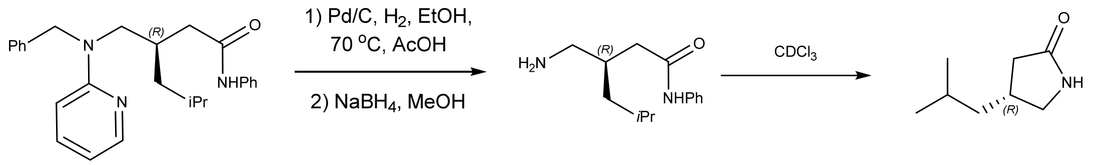

6p **6pc** **6pb**

(*R*)-3-((Benzyl(pyridin-2-yl)amino)methyl)-5-methyl-*N*-phenylhexanamide **6p** (27 mg, 0.067 mmol, 1 eq), palladium on carbon 10% w/w (14 mg, 0.013 mmol, 0.2 eq), EtOH (0.5 – 1 mL) and acetic acid (17 µL, 0.34 mmol, 5 eq) were measured into a 10 mL, oven-dried microwave vial. The vial was sealed and evacuated and backfilled with hydrogen 3 times. A balloon of hydrogen was placed on the vial and the reaction mixture was stirred at 70 °C overnight. The reaction mixture was allowed to cool down to ambient temperature and the mixture was filtered through small pad of celite. The mixture was concentrated *in vacuo*. The dry mixture was dissolved in methanol (1 – 2 mL) and $NaBH_4$ (13 mg, 0.33 mmol, 5 eq) was added portionwise at 0 °C and the mixture was stirred at 0 °C for 3h. The mixture was acidified by dropwise addition of 3 M HCl at 0 °C, stirred for 10 minutes and neutralised by addition of K_3PO_4. The mixture was extracted with DCM. The combined organic layers were dried over MgSO4, filtered, and concentrated *in vacuo*. The crude mixture was purified by silica gel chromatography (eluting with 0 to 5% MeOH in DCM). It was observed that the purified material cyclised in the NMR tube and the material was repurified by silica gel chromatography (eluting with 0 to 30% EtOAc in petroleum ether) to yield the title product **6pb** (7 mg, 0.03 mmol, 45% yield, 99% ee).

^{1}H NMR (700 MHz, $CDCl_3$) δ 6.91 (br. s, 1H), 8.14 (s, 1H), 3.44 (t, *J* = 8.68 Hz, 1 H), 2.95 (dd, *J* = 7.4, 9.2 Hz, 1 H), 2.49 (sept, *J* = 7.81 Hz, 1 H), 2.37 (dd, *J* = 8.6, 16.6 Hz, 1 H), 1.94 (dd, *J* = 8.5, 16.6 Hz, 1 H), 1.54 (sept, *J* = 6.7 Hz, 1 H), 1.31 (dt, *J* = 3.6, 7.2 Hz, 2 H), 0.86 (t, *J* = 7.0 Hz, 6 H). **^{13}C NMR** (176 MHz, $CDCl_3$) δ 179.1, 48.7, 44.1, 37.4, 33.2, 26.4, 23.0, 22.8. **$[\alpha]_D^{25.0}$** = +2.1 (*c* 0.6, $CHCl_3$) (lit. **$[\alpha]_D^{25.0}$** = +2.1 (*c* 0.5, $CHCl_3$).[9]

The spectral data are in good agreement with the previous literature.[10]

Use of an enamide as the acceptor

(S)-N-(3-(Phenyl(pyridin-2-yl)amino)butyl)benzamide (9)

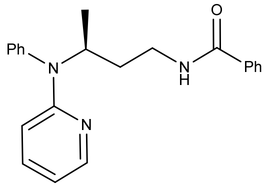

9

Following the general procedure for the asymmetric Giese reaction using *N*-ethyl-*N*-phenyl-2-aminopyridine **1d** (1.0 mL, 0.20 mmol, 0.2 M in anhydrous $PhCF_3$, 1 eq), *N*-vinylbenzamide **8** (44 mg, 0.30 mmol, 1.5 eq) and $(Ir[dF(CF_3)ppy]_2(dtpby))PF_6$ (6.6 mg, 6.0 µmol, 3 mol%) for 24 h. The crude was purified without further workup by silica gel column chromatography (eluting with 20% EtOAc in 0.5% TEA in petroleum ether) to yield the title compound **9** as an off-white solid (21 mg, 0.061 mmol, 30% yield, 98% ee).

^{1}H NMR (400 MHz, CD_3CN) δ 8.08 (ddd, *J* = 5.1, 2.0, 0.9 Hz, 1H), 7.90 – 7.84 (m, 3H), 7.58 – 7.47 (m, 5H), 7.45 – 7.40 (m, 1H), 7.29 (ddd, *J* = 8.8, 7.1, 2.0 Hz, 1H), 7.26 – 7.22 (m, 2H), 6.57 (ddd, *J* = 7.1, 5.1, 0.9 Hz, 1H), 5.97 (d, *J* = 8.8 Hz, 1H), 5.33 (dqd, *J* = 8.9, 6.9, 5.7 Hz, 1H), 3.69 (ddt, *J* = 13.3, 7.2, 5.7 Hz, 1H), 3.30 – 3.19 (m, 1H), 1.80 (ddt, *J* = 14.3, 8.9, 5.7 Hz, 1H), 1.66 (ddt, *J* = 14.3, 8.5, 5.7 Hz, 1H), 1.10 (d, *J* = 6.9 Hz, 3H). **^{13}C NMR** (101 MHz, CD_3CN) δ 167.6, 160.3, 147.7, 141.4, 138.2, 136.3, 132.3, 132.1, 130.9, 129.5, 128.6, 127.9, 113.5, 110.0, 48.7, 37.7, 36.1, 20.4. **HRMS** m/z: $[M + H]^+$ calc'd for $[C_{22}H_{24}N_3O]^+$ expect 346.1919; found 346.1927. **$[\alpha]_D^{25.0}$** = +185.5 (*c* 1.0, $CHCl_3$).

Less effective substrate

(*S*)-4-(Methyl(pyridin-2-yl)amino)-*N*,3-diphenylbutanamide (10)

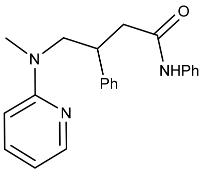

10

Following the general procedure for the asymmetric Giese reaction using *N*,*N*-dimethylpyridin-2-amine (1.0 mL, 0.20 mmol, 0.2 M in anhydrous $PhCF_3$, 1 eq), *N*-phenylcinnamamide **11** (67 mg, 0.30 mmol, 1.5 eq) and $(Ir[dF(CF_3)ppy]_2(dtpby))PF_6$ (1.1 mg, 1.0 µmol, 0.5 mol%) for 8 h. The crude mixture was purified by preparative reverse-phase HPLC (eluting with 20 to 40% MeCN in 0.1% formic acid in water) to yield the title compound (36 mg, 0.10 mmol, 52% yield, 37% ee) as an off-white powder.

^{1}H NMR (400 MHz, $CDCl_3$) δ 8.59 (br. s, 1H), 8.22 (dd, *J* = 5.0, 2.0 Hz, 1H), 7.48 (ddd, *J* = 8.7, 7.3, 2.0 Hz, 1H), 7.38 (d, *J* = 8.0 Hz, 2H), 7.31 – 7.23 (m, 6H), 7.22 – 7.17 (m, 1H), 7.05 (t, *J* = 7.3 Hz, 1H), 6.62 (dd, *J* = 7.3, 5.0 Hz, 1H), 6.45 (d, *J* = 8.7 Hz, 1H), 4.09 (dd, *J* = 14.4, 7.8 Hz, 1H), 3.59 (dd, *J* = 14.4, 5.0 Hz, 1H), 3.28 – 3.11 (m, 2H), 2.90 (s, 3H), 2.88 – 2.80 (m, 1H). **^{13}C NMR** (101 MHz, $CDCl_3$) δ 172.5, 158.3, 147.3, 139.6, 138.3, 138.0, 129.2, 128.9, 128.7, 126.5, 124.1, 120.2, 112.3, 106.5, 53.9, 49.3, 38.0, 37.0. **HRMS** m/z: $[M + H]^+$ calc'd for $[C_{22}H_{24}N_3O]^+$ expect 346.1914; found 346.1913. **$[\alpha]_D^{25.0}$** = +44.6 (*c* 1.0, $CHCl_3$).

Synthesis of Starting Materials

2-Benzyl-*N*-phenylacrylamide (4b)

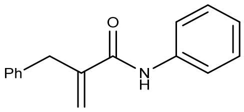

Following the general procedure for the amide coupling, using aniline (0.46 mL, 5.0 mmol, 1 eq) and 2-benzylacrylic acid (1.1 g, 6.5 mmol, 1.3 eq). The crude product was purified by silica gel chromatography (eluting with 5% to 10% EtOAc in petroleum ether), followed by trituration with Et_2O to afford the title compound **4b** as a white solid (0.602 g, 2.55 mmol, 51%).

^{1}H NMR (400 MHz, $CDCl_3$) δ 7.44 (d, *J* = 7.9 Hz, 2H), 7.39 (br. s, 1H), 7.36 – 7.22 (m, 6H), 7.09 (t, *J* = 7.4 Hz, 1H), 5.90 (s, 1H), 5.30 (s, 1H), 3.76 (s, 2H). **^{13}C NMR** (101 MHz, $CDCl_3$) δ 166.2, 145.0, 138.3, 137.8, 129.1, 129.1, 129.0, 127.0, 124.6, 120.7, 120.0, 38.9.

The spectral data are in good agreement with the previous literature.[11]

(*E*)-5-Methyl-*N*-phenylhex-2-enamide (4c)

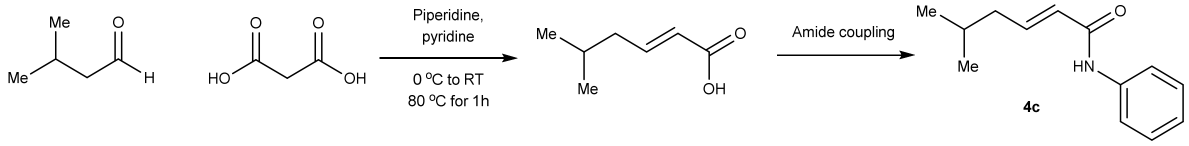

(*E*)-5-Methylhex-2-enoic acid was prepared according to a literature procedure: pyridine (4 mL) was added to isovaleraldehyde (4.89 mL, 46.4 mmol, 1 eq) at 0 °C followed by dropwise addition of malonic acid (4.77 mL, 74.3 mmol, 1.6 eq) and sequential addition of piperidine (0.4 mL). The reaction mixture was allowed to warm up to ambient temperature and the mixture was stirred overnight. Mixture was further heated at 80 °C for 1.5 h, then cooled down to ambient temperature and quenched by addition of 1 M HCl. The reaction mixture was extracted with DCM and the combined organic layers were washed with water and brine, dried over $MgSO_4$, filtered and concentrated *in vacuo* affording (*E*)-5-methylhex-2-enoic acid (4.3 g, 34 mmol, 72%) as a colorless liquid which was used without further purification in the next amide coupling.

Following the general procedure for the amide coupling, using aniline (0.824 mL, 9.02 mmol, 1.3 eq) and (*E*)-5-methylhex-2-enoic acid (1.00 g, 6.94 mmol, 1.0 eq). The crude product was purified by silica gel chromatography (eluting with 10% to 20% Et_2O in petroleum ether), followed by trituration with Et_2O to afford the title compound **4c** as a white solid (1.33 g, 6.07 mmol, 88%).

^{1}H NMR (400 MHz, $CDCl_3$) δ 7.57 (br. d, *J* = 7.5 Hz, 2H), 7.36 – 7.27 (m, 3H), 7.10 (t, *J* = 7.4 Hz, 1H), 6.96 (dt, *J* = 15.0, 7.5 Hz, 1H), 5.92 (dt, *J* = 14.8, 1.2 Hz, 1H), 2.13 – 2.07 (m, 2H), 1.77 (sept, *J* = 6.6 Hz, 1H), 0.93 (d, *J* = 6.6 Hz, 6H). **^{13}C NMR** (101 MHz, $CDCl_3$) δ 164.2, 145.6, 138.2, 129.8, 129.1, 129.1, 125.0, 124.4, 120.1, 120.0, 41.6, 28.0, 22.5; **HRMS** m/z: $[M + H]^+$ calc'd for $[C_{13}H_{18}NO]^+$ expect 204.1383; found 204.1382.

(*E*)-*N*-Phenylbut-2-enamide (4d)

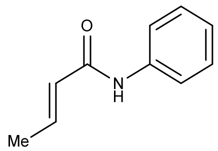

Following the general procedure for the amide coupling, using aniline (1.12 mL, 12.3 mmol, 1.0 eq) and crotonic acid (1.17 g, 13.6 mmol, 1.1 eq). The crude product was purified by silica gel column chromatography (eluting with 50% Et_2O in *n*-hexane), followed by trituration to afford the title compound **4d** as a white solid (335 mg, 2.08 mmol, 17%).

^{1}H NMR (500 MHz, $CDCl_3$) δ 7.60 – 7.52 (m, 3H), 7.34 – 7.25 (m, 2H), 7.13 – 7.05 (m, 1H), 6.98 (dq, *J* = 15.1, 6.9 Hz, 1H), 5.98 (dq, *J* = 15.1, 1.7 Hz, 1H), 1.88 (dd, *J* = 6.9, 1.7 Hz, 3H). **^{13}C NMR** (126 MHz, $CDCl_3$) δ 164.3, 141.6, 138.2, 129.1, 125.6, 124.4, 120.1, 18.0.

The data agree with the literature.[12]

2-Benzyl-*N*-methyl-*N*-phenylacrylamide (4e)

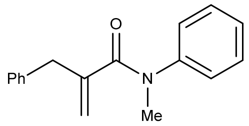

Following the general procedure for the amide coupling, using *N*-methylaniline (1.0 mL, 9.3 mmol, 1 eq) and 2-benzylacrylic acid (1.50 g, 9.25 mmol, 1 eq). The crude product was purified by silica gel chromatography (eluting with 10% to 20% EtOAc in petroleum ether), to afford the title compound **4e** as an off-white solid (1.5 g, 5.9 mmol, 64%).

^{1}H NMR (700 MHz, $CDCl_3$) δ 7.31 – 7.23 (m, 6H), 7.10 (d, *J* = 7.4 Hz, 2H), 6.05 (d, *J* = 7.5 Hz, 2H), 5.00 (s, 1H), 4.97 (s, 1H), 3.49 (s, 2H), 3.31 (s, 3H). **^{13}C NMR** (176 MHz, $CDCl_3$) δ 171.1, 144.5, 137.9, 129.6, 129.2, 128.5, 127.0, 126.9, 126.6, 119.5, 40.5, 38.0. **HRMS** m/z: $[M + H]^+$ calc'd for $[C_{17}H_{18}NO]^+$ expect 252.1383; found 252.1381.

The spectral data are in good agreement with the previous literature.[13]

2-Benzyl-*N*-mesitylacrylamide (4f)

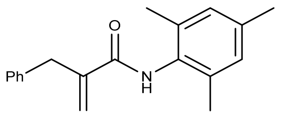

Following the general procedure for the amide coupling, using 2,4,6-trimethylaniline (10.2 mmol, 1.43 mL, 1.1 eq) and 2-benzylacrylic acid (9.25 mmol, 1.50 g, 1 eq). The crude product was purified by silica gel chromatography (eluting with 10% to 20% EtOAc in petroleum ether), followed by trituration with Et_2O to afford the title compound **4f** as a white solid (1.92 g, 6.88 mmol, 74%).

^{1}H NMR (400 MHz, $CDCl_3$) δ 7.34 – 7.27 (m, 4H), 7.26 – 7.21 (m, 1H), 6.84 (br. s, 2H), 6.80 (br. s, 1H), 5.95 (br. s, 1H), 5.46 (q, *J* = 1.07 Hz, 1H), 3.78 (br. s, 2H), 2.24 (br. s, 3H), 1.97 (br. s, 2H). **^{13}C NMR** (126 MHz, $CDCl_3$) δ 166.7, 144.6, 138.3, 137.1, 135.2, 131.0, 129.1, 129.0, 128.9, 127.0, 120.3, 39.5, 21.0, 18.1. **HRMS** m/z: $[M + H]^+$ calc'd for $[C_{19}H_{22}NO]^+$ expect 280.1689; found 280.1696.

3-Methyl-2-methylene-*N*-phenylbutanamide (4g)

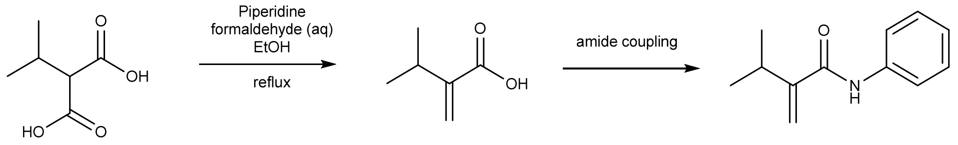

4g

3-Methyl-2-methylenebunatonic acid and 2-methylenebunatonic acid were prepared according to a literature procedure.[14] 2-Ethylmalonic acid (1.32 g, 10 mmol), piperidine (1.19 mL, 12 mmol), 37% aqueous formaldehyde (4 mL) in ethanol (30 mL) were stirred at 80 °C for overnight. The reaction mixture was cooled to ambient temperature and concentrated to dryness. The crude material was treated with Et_2O and aqueous saturated $NaHCO_3$. The organic and aqueous layer were separated and the organic layer was washed with additional aqueous saturated $NaHCO_3$. The combined aqueous layers were washed with Et_2O and acidified with concentrated HCl to pH 1. The acidified aqueous layer was extracted with Et_2O. The combined organic layers were washed with brine, dried over $MgSO_4$ and concentrated *in vacuo* yielding 3-methyl-2-methylenebunatonic acid (0.880 g, 7.71 mmol, 77%) which was used in the next step without further purification.

Following the general procedure for the amide coupling, using aniline (0.69 mL, 5.8 mmol, 1.0 eq) and 3-methyl-2-methylenebutanoic acid (0.86 g, 7.5 mmol, 1.3 eq). The crude product was purified by silica gel chromatography (eluting with 0% to 20% Et_2O in petroleum ether), followed by trituration with Et_2O to afford the title compound **4g** as a white solid (0.81 g, 4.3 mmol, 74%).

^{1}H NMR (400 MHz, $CDCl_3$) δ 7.57 (br. d, *J* = 6.5 Hz, 2H), 7.51 (br. s, 1H), 7.31 (t, *J* = 7.7 Hz, 2H), 7.09 (t, *J* = 7.2 Hz, 1H), 6.96 (dd, *J* = 15.3, 6.6 Hz, 1H), 5.89 (d, *J* = 15.5 Hz, 1H), 2.47 (sext, *J* = 6.7 Hz, 1H), 1.07 (d, *J* = 6.8 Hz, 6H). **^{13}C NMR** (101 MHz, $CDCl_3$) δ 164.6, 152.7, 138.3, 129.1, 124.3, 121.4, 120.1, 31.0, 21.5. **HRMS** m/z: $[M + H]^+$ calc'd for $[C_{12}H_{15}NO]^+$ expect 190.1226; found 190.1226.

Methyl 3-(phenylcarbamoyl)but-3-enoate (4h)

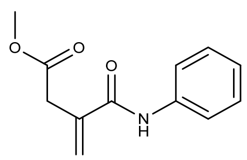

4h

Following the general procedure for the amide coupling, using aniline (5.0 mmol, 0.46 mL, 1 eq) and 4-methyl itaconate (5.5 mmol, 0.79 g, 1.1 eq). The crude product was purified by silica gel chromatography (eluting with 0% to 20% EtOAc in petroleum ether), followed by trituration with Et_2O to afford the title compound **4h** as a white solid (0.92 g, 0.42 mmol, 84%).

^{1}H NMR (400 MHz, $CDCl_3$) δ 8.10 (br. s, 1H), 5.56 (d, *J* = 8.0 Hz, 2H), 7.33 (t, *J* = 7.5 Hz, 2H), 7.11 (t, *J* = 7.4 Hz, 1H), 5.94 (s, 2H), 5.59 (s, 1H), 3.72 (s, 1H), 3.46 (s, 2H). **^{13}C NMR** (176 MHz, $CDCl_3$) δ 171.9, 165.8, 139.0, 137.9, 129.1, 124.6, 122.6, 120.2, 52.4, 38.2. **HRMS** m/z: $[M + H]^+$ calc'd for $[C_{12}H_{14}NO_3]^+$ expect 220.0968; found 220.0971.

2-Methylene-*N*-phenylbutanamide (4i)

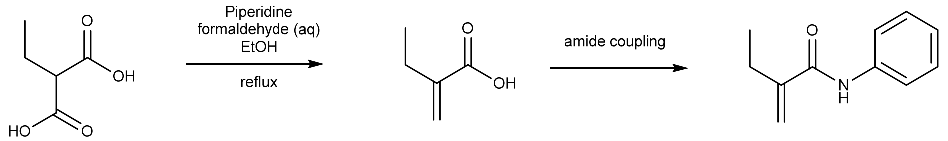

4i

2-Methylenebunatonic acid was prepared according to a literature procedure described above, using 2-ethylmalonic acid (1.32 g, 10.0 mmol) as a starting material to afford 2-methylenebutanoic acid (0.79 g, 7.9 mmol, 79%) which was used in the next step without further purification.

Following the general procedure for the amide coupling, using aniline (5.80 mmol, 0.530 mL, 1 eq) and 2-methylenebutanoic acid (7.54 mmol, 0.792 g, 1.3 eq). The crude product was purified by silica gel chromatography (eluting with 0% to 20% Et_2O in petroleum ether), followed by trituration with Et_2O to afford the title compound **4i** as a white solid (0.67 g, 3.8 mmol, 66%).

^{1}H NMR (400 MHz, $CDCl_3$) δ 7.64 (br. s, 1H), 7.56 (d, *J* = 8.4 Hz, 2H), 7.32 (t, *J* = 8.1 Hz, 2H), 7.11 (tt, *J* = 7.4, 1.1 Hz, 1H), 5.71 (s, 1H), 5.39 (t, *J* = 1.6 Hz, 1H), 2.43 (qt, *J* = 7.4, 1.2 Hz, 2H), 1.12 (t, *J* = 7.4 Hz, 3H). **^{13}C NMR** (101 MHz, $CDCl_3$) δ 167.4, 148.0, 138.2, 129.3, 124.7, 120.3, 117.1, 25.6, 12.7. **HRMS** m/z: $[M + H]^+$ calc'd for $[C_{11}H_{13}NO]^+$ expect 176.1070; found 176.1069.

(*E*)-4-Methyl-*N*-phenylpent-2-enamide (4j)

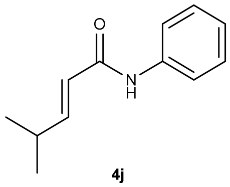

4j

Following the general procedure for the amide coupling, using aniline (1.12 mL, 12.3 mmol, 1.0 eq) and (*E*)-4-methylpent-2-enoic acid (1.62 mL, 13.6 mmol, 1.1 eq). The crude product was purified by silica gel column chromatography (eluting with 0% to 20% EtOAc in petroleum ether), followed by trituration to afford the title compound **4j** as a white solid (1.12 g, 5.90 mmol, 48%).

^{1}H NMR (500 MHz, $CDCl_3$) δ 7.57 (d, *J* = 7.9 Hz, 2H), 7.48 (br. s, 1H), 7.35 – 7.28 (m, 2H), 7.10 (t, *J* = 7.6 Hz, 1H), 6.96 (dd, *J* = 15.3, 6.6 Hz, 1H), 5.89 (dd, *J* = 15.3, 1.5 Hz, 1H), 2.53-2.40 (dsext, *J* = 6.8, 1.5 1H), 1.07 (d, *J* = 6.8 Hz, 6H). **^{13}C NMR** (126 MHz, $CDCl_3$) δ 164.6, 152.8, 138.2, 129.1, 124.3, 121.4, 120.1, 31.0, 21.5. **HRMS** m/z: $[M + H]^+$ calc'd for $[C_{12}H_{16}NO]^+$ expect 190.1232; found 190.1226.

(*E*)-*N*-(4-(Trifluoromethyl)phenyl)but-2-enamide (4k)

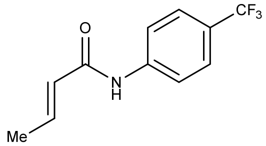

4k

Following the general procedure for the amide coupling, using 4-(trifluoromethyl)aniline (1.75 mL, 9.02 mmol, 13.9 mL, 1.2 eq) and crotonic acid (1.00 g, 11.6 mmol, 1.0 eq). The reaction mixture was stirred at 0 °C for 2 h. The crude was concentrated *in vacuo* and was purified by silica gel chromatography (eluting with 15% EtOAc in petroleum ether), followed by trituration with Et_2O to afford the title compound **4k** as a white solid (0.620 g, 2.71 mmol, 23%).

^{1}H NMR (400 MHz, DMSO) δ 10.31 (br. s, 1H), 3.85 (d, *J* = 8.6 Hz, 2H), 7.67 (d, *J* = 8.9 Hz, 2H), 6.86 (dq, *J* = 14.7, 7.1 Hz, 1H), 6.14 (dq, *J* = 15.0, 1.5 Hz, 1H), 1.88 (dd, *J* = 6.9, 1.6 Hz, 3H). **^{13}C NMR** (101 MHz, DMSO) δ 164.0, 142.9, 141.2, 126.1 (q, $^{3}J_{C\text{-}F}$ = 3.8 Hz), 125.6, 124.5 (q, $^{1}J_{C\text{-}F}$ = 271.2 Hz), 123.2 (q, $^{2}J_{C\text{-}F}$ = 31.9 Hz), 119.2, 17.6. **^{19}F NMR** (471 MHz, $CHCl_3$) δ **–**63.1. **HRMS** m/z: $[M + H]^+$ calc'd for $[C_{11}H_{11}F_3NO]^+$ expect 230.0787; found 230.0789.

(*E*)-*N*,5-Diphenylpent-2-enamide (4l)

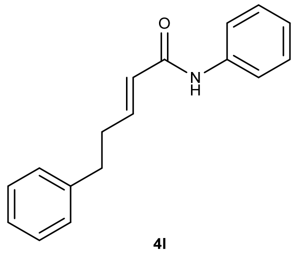

4l

Under a dry, inert atmosphere, *N*-phenylacrylamide (250 mg, 1.70 mmol, 1.0 eq) and 4-phenyl-1-butene (0.26 mL, 1.70 mmol, 1.0 eq) were dissolved in anhydrous DCM (17 mL). The mixture was degassed and then Grubbs first generation catalyst (28 mg, 3.4 µmol, 2 mol%) was added. The reaction mixture was stirred at room temperature for 4 h, after which it was concentrated *in vacuo*. The crude mixture was purified by silica gel column chromatography (eluting with 0% to 20% EtOAc in petroleum ether) to afford the title compound **4l** as a white powder (84 mg, 0.33 mmol, 20%).

^{1}H NMR (500 MHz, $CDCl_3$) δ 7.63 – 7.50 (m, 2H), 7.35 – 7.27 (m, 4H), 7.24 – 7.17 (m, 4H), 7.11 (t, *J* = 7.4 Hz, 1H), 7.03 (dt, *J* = 15.2, 7.0 Hz, 1H), 5.92 (dt, *J* = 15.2, 1.6 Hz, 1H), 2.80 (t, *J* = 8.3 Hz, 2H), 2.56 (dtd, *J* = 8.3, 7.0, 1.6 Hz, 2H). **^{13}C NMR** (126 MHz, $CDCl_3$) δ 164.0, 145.4, 141.0, 138.1, 129.2, 128.6, 128.5, 126.3, 124.6, 124.4, 120.0, 34.7, 34.0. **HRMS** m/z: $[M + H]^+$ calc'd for $[C_{17}H_{18}NO]^+$ expect 252.1388; found 252.1384.

(*E*)-2-Methyl-*N*-phenylbut-2-enamide (4m)

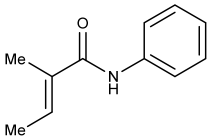

4m

Following the general procedure for the amide coupling, using aniline (1.12 mL, 12.3 mmol, 1.0 eq) and tiglic acid (1.36 g, 13.6 mmol, 1.1 eq). The crude product was purified by silica gel column chromatography (eluting with 0% to 20% EtOAc in petroleum ether), followed by trituration to afford the title compound **4m** as a white solid (1.87 g, 9.88 mmol, 80%).

^{1}H NMR (400 MHz, $CDCl_3$) δ 7.58 – 7.53 (m, 2H), 7.50 (br. s, 1H), 7.36 – 7.27 (m, 2H), 7.13 – 7.03 (m, 1H), 6.51 (qq, *J* = 6.8, 1.2 Hz, 1H), 1.94 (t, *J* = 1.2 Hz, 3H), 1.80 (dq, *J* = 6.8, 1.2 Hz, 3H). **^{13}C NMR** (101 MHz, $CDCl_3$) δ 167.8, 138.3, 133.0, 131.3, 129.1, 124.2, 120.1, 14.20, 12.7.

The data agree with the literature.[15]

N-Phenylcyclohex-1-ene-1-carboxamide (4n)

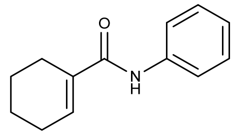

4n

Following the general procedure for the amide coupling, using aniline (1.12 mL, 12.3 mmol, 1.0 eq) and cyclohex-1-ene-1-carboxylic acid (1.56 mL, 13.6 mmol, 1.1 eq). The crude product was purified by silica gel column chromatography (eluting with 0% to 20% EtOAc in petroleum ether), followed by trituration to afford the title compound **4n** as a white solid (1.73 g, 8.60 mmol, 70%).

^{1}H NMR (400 MHz, $CDCl_3$) δ 7.59 – 7.50 (m, 2H), 7.36 (br. s, 1H), 7.35 – 7.29 (m, 3H), 7.13 – 7.06 (m, 1H), 6.74 (tt, *J* = 3.8, 1.7 Hz, 1H), 2.40 – 2.33 (m, 2H), 2.26 – 2.20 (m, 2H), 1.78 – 1.70 (m, 2H), 1.68 – 1.61 (m, 2H). **^{13}C NMR** (126 MHz, $CDCl_3$) δ 166.9, 138.2, 134.4, 134.2, 129.2, 124.3, 120.1, 25.7, 24.5, 22.3, 21.6.

The data agree with the literature.[16]

_N_-Vinylbenzamide (8)

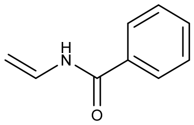

Under a dry, inert atmosphere, a solution of *N*-vinylformamide (0.99 mL, 14.1 mmol, 1.00 eq) and triethylamine (2.3 mL, 16.2 mmol, 1.20 eq) in anhydrous THF (35 mL) was cooled to 0 °C. Benzoyl chloride (1.9 mL, 16.2 mmol, 1.15 eq) was added dropwise and the mixture was stirred and allowed to warm to room temperature overnight. The reaction mixture was then cooled to 0 °C and sodium hydroxide (17.0 mL, 42.2 mmol, 2.5 M in water, 3.00 eq) was added. The resulting mixture was stirred at room temperature for 8 h, after which it was extracted three times with EtOAc. The combined organic layer was washed three times with brine, dried over sodium sulfate, and concentrated *in vacuo*. The crude mixture was purified by silica gel column chromatography (eluting with 0% to 20% EtOAc in petroleum ether) to afford the title compound **(8)** as a white powder (600 mg, 4.08 mmol, 29%).

^{1}H NMR (400 MHz, DMSO-d_6) δ 10.37 (d, *J* = 9.9 Hz, 1H), 7.92 (d, *J* = 7.6 Hz, 2H), 7.63 – 7.54 (m, 1H), 7.53-7.41 (m, 2H), 7.05 (dt, *J* = 16.0, 9.4 Hz, 1H), 4.93 (d, *J* = 16.0 Hz, 1H), 4.45 (d, *J* = 8.8 Hz, 1H). **^{13}C NMR** (101 MHz, DMSO-d_6) δ 164.1, 133.4, 131.9, 129.8, 128.4, 127.6, 96.4.

The data agree with the literature.[17]

_N_-Phenylcinnamamide (11)

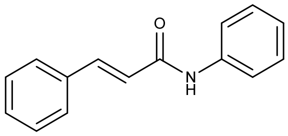

Following the general procedure for the amide coupling, using aniline (0.46 mL, 5.0 mmol, 1.0 eq) and cinnamic acid (0.81 mL, 5.5 mmol, 1.1 eq). The crude product was purified by silica gel column chromatography (eluting with 0% to 50% EtOAc in petroleum ether), followed by trituration to afford the title compound **11** as a white solid (0.46 g, 2.0 mmol, 40%).

^{1}H NMR (400 MHz, $CDCl_3$) δ 7.76 (d, *J* = 15.5 Hz, 1H), 7.67 – 7.59 (m, 2H), 7.57 – 7.49 (m, 2H), 7.46 (s, 1H), 7.41 – 7.31 (m, 5H), 7.14 (t, *J* = 7.4 Hz, 1H), 6.57 (d, *J* = 15.5 Hz, 1H). **^{13}C NMR** (126 MHz, $CDCl_3$) δ 164.1, 142.6, 138.1, 134.7, 130.2, 129.2, 129.0, 128.1, 124.6, 120.9, 120.1.

The data agree with the literature.[18]

2-(Pyrrolidin-1-yl)pyridine (1a)

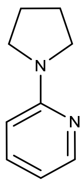

Following the general procedure for the copper-catalysed C-N cross-coupling, using 2-bromopyridine (0.95 mL, 10 mmol, 1 eq) and pyrrolidine (1.6 mL, 20 mmol, 2 eq) at 80 °C. The crude product was purified by silica gel chromatography (eluting with 0% to 15% EtOAc in petroleum ether) to afford the title compound (**1a**) as colorless viscous liquid (0.701 g, 4.73 mmol, 47%).

^{1}H NMR (400 MHz, $CDCl_3$) δ 8.15 (d, *J* = 4.5 Hz, 1H), 7.44 – 7.38 (m, 1H), 6.52 – 6.47 (m, 1H), 6.34 (d, *J* = 8.5 Hz, 1H), 3.48 – 3.41 (m, 4H), 2.03 – 1.98 (m, 4H). **^{13}C NMR** (101 MHz, $CDCl_3$) δ 157.4, 148.3, 137.0, 111.1, 106.6, 46.8, 25.7.

The spectral data are in good agreement with the previous literature.[19]

N-Benzyl-N-methyl-2-aminopyridine (1c)

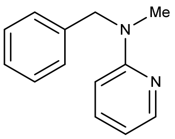

1c

Following the general procedure for the Buchwald-Hartwig coupling, using 2-bromypyridine (1.24 mL, 12.8 mmol, 1.0 eq) and *N*-methyl-1-phenylmethanamine (1.81 mL, 14.0 mmol, 1.1 eq). The crude was purified by silica gel column chromatography (eluting with 0% to 10% Et_2O in petroleum ether) to afford the title compound **1c** as a yellow oil (1.99 g, 10.0 mmol, 78%).

^{1}H NMR (400 MHz, $CDCl_3$) δ 8.25 – 8.18 (m, 1H), 7.46 (t, *J* = 7.7 Hz, 1H), 7.38 – 7.30 (m, 2H), 7.29 – 7.27 (m, 2H), 7.25 – 7.22 (m, 1H), 6.59 (t, *J* = 5.4 Hz, 1H), 6.54 (d, *J* = 8.5 Hz, 1H), 4.83 (s, 2H), 3.11 (s, 3H). **^{13}C NMR** (101 MHz, $CDCl_3$) δ 158.9, 147.9, 138.8, 137.6, 128.7, 127.1, 127.0, 112.0, 105.9, 53.4, 36.4.

The data agree with the literature.[20]

N-Ethyl-N-phenyl-2-aminopyridine (1d)

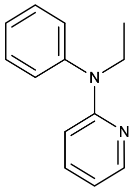

1d

Under a dry, inert atmosphere, sodium *tert*-butoxide (2.30 g, 23.9 mmol, 1.20 eq), RuPhos (230 mg, 0.48 mmol, 2.5 mol%), palladium(II) acetate (110 mg, 0.48 mmol, 2.5 mol%), *N*-ethylaniline (2.44 mL, 19.4 mmol, 1.00 eq), and 2-bromopyridine (1.95 mL, 20.4 mmol, 1.05 eq) were added to a reaction vial. The vial was evacuated and refilled with nitrogen three times. Anhydrous toluene (5-10 mL) was added and the reaction was stirred overnight at 110 °C. The reaction mixture was cooled to room temperature and filtered through celite. The mixture was then concentrated *in vacuo* and purified by silica gel column chromatography (eluting with 0% to 5% Et_2O in *n*-hexane) to afford the desired product **1d** as a yellow oil (2.57 g, 13.0 mmol, 67%).

^{1}H NMR (500 MHz, $CDCl_3$) δ 8.21 (d, *J* = 4.9 Hz, 1H), 7.41 (t, *J* = 7.7 Hz, 2H), 7.29 – 7.27 (m, 1H), 7.26 – 7.23 (m, 3H), 6.57 (dd, *J* = 7.0, 4.9 Hz, 1H), 6.36 (d, *J* = 8.6 Hz, 1H), 4.02 (q, *J* = 7.0 Hz, 3H), 1.23 (t, *J* = 7.0 Hz, 4H). **^{13}C NMR** (126 MHz, $CDCl_3$) δ 158.5, 147.9, 145.4, 136.7, 129.9, 127.9, 126.0, 112.8, 109.2, 44.9, 13.3.

The data agree with the literature.[21]

2-(Pyrrolidin-1-yl)pyrimidine (1e)

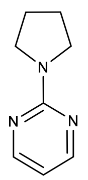

1e

Following the general procedure for the copper-catalysed C-N cross-coupling, using 2-bromopyrimidine (2.4 mL, 23 mmol, 1 eq) and pyrrolidine (3.7 mL, 45 mmol, 2 eq) at ambient temperature. The crude product was purified by silica gel chromatography (eluting with 0% to 15% EtOAc in petroleum ether) to afford the title compound **1e** (2.2 g, 15 mmol, 66%).

^{1}H NMR (400 MHz, $CDCl_3$) δ 8.28 (d, *J* = 4.8 Hz, 2H), 6.41 (t, *J* = 4.9 Hz, 1H), 3.58 – 3.49 (m, 4H), 2.01 – 1.92 (m, 4H). **^{13}C NMR** (101 MHz, $CDCl_3$) δ 160.3, 157.8, 108.9, 46.7, 25.6.

The data agree with the literature.[22]

2-(Piperidin-1-yl)pyridine (1f)

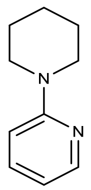

1f

Following the general procedure for the copper-catalysed C-N cross-coupling, using piperidine (1.08 mL, 20.0 mmol, 2 eq) and 2-bromopyridine (0.958 mL, 10.0 mmol, 1 eq) at 80 °C. The crude product was purified by silica gel chromatography (eluting with 0% to 4% MeOH in DCM) to afford the title compound **1f** as colorless viscous oil (0.76 g, 4.7 mmol, 47%)

^{1}H NMR (400 MHz, $CDCl_3$) δ 8.17 (ddd, *J* = 5.0, 1.9, 0.7 Hz, 1H), 7.44 (ddd, *J* = 8.7, 7.0, 1.9 Hz, 1H), 6.65 (d, *J* = 8.6 Hz, 1H), 6.55 (ddd, *J* = 7.0, 5.0, 0.5 Hz, 1H), 3.57 – 3.48 (m, 4H), 1.69 – 1.59 (m, 6H). **^{13}C NMR** (176 MHz, $CDCl_3$) δ 159.8, 147.9, 137.5, 112.5, 107.3, 46.5, 25.7, 24.9.

The spectral data are in good agreement with the previous literature.[23]

1-(Pyridin-2-yl)azepane (1g)

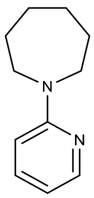

1g

Following the general procedure for the copper-catalysed C-N cross-coupling, using hexamethyleneimine (0.808 mL, 10.0 mmol, 2 eq) and 2-bromopyridine (0.476 mL, 5.00 mmol, 1 eq) at 80 °C. The crude product was purified by silica gel chromatography (eluting with 0% to 20% Et_2O in petroleum ether) to afford the title compound **1g** as colourless viscous oil (0.574 g, 3.85 mmol, 77%)

^{1}H NMR (500 MHz, $CDCl_3$) δ 8.13 (dd, *J* = 5.8, 1.9 Hz, 1H), 7.40 (ddd, *J* = 8.6, 7.1, 2.1 Hz, 1H), 6.49 – 6.45 (m, 2H), 3.63 – 3.60 (m, 4H), 1.81 – 1.74 (m, 4H), 1.59 – 1.51 (m, 4H). **^{13}C NMR** (126 MHz, $CDCl_3$) δ 158.4, 148.1, 137.2, 111.0, 105.5, 47.6, 28.0, 27.4.

The spectral data are in good agreement with the previous literature.[24]

***N*,*N*-Diethyl-2-aminopyridine (1h)**

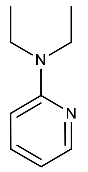

1h

Following the general procedure for the Buchwald-Hartwig coupling, using 2-bromypyridine (0.31 mL, 3.2 mmol, 1.0 eq) and diethylamine (0.66 mL, 6.4 mmol, 2.0 eq). The crude was purified by silica gel column chromatography (eluting with 0% to 10% Et_2O in petroleum ether) to afford the title compound **1h** as a yellow oil (115 mg, 0.77 mmol, 24%).

^{1}H NMR (500 MHz, $CDCl_3$) δ 8.14 (dd, *J* = 5.1, 2.0 Hz, 1H), 7.39 (ddd, *J* = 8.9, 7.0, 2.0 Hz, 1H), 6.55 – 6.39 (m, 2H), 3.51 (q, *J* = 7.1 Hz, 4H), 1.18 (t, *J* = 7.1 Hz, 6H). **^{13}C NMR** (126 MHz, $CDCl_3$) δ 157.7, 148.3, 137.1, 110.9, 105.6, 42.5, 13.1.

The data agree with the literature.[25]

N-Benzyl-N-ethyl-2-aminopyridine (1i)

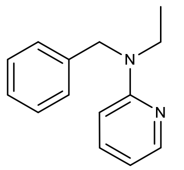

1i

Following the general procedure for the Buchwald-Hartwig coupling, using 2-bromypyridine (0.31 mL, 3.2 mmol, 1.0 eq) and N-benzylethanamine (0.52 mL, 3.5 mmol, 1.1 eq). The crude was purified by silica gel column chromatography (eluting with 0% to 10% Et_2O in petroleum ether) to afford the title compound **1i** as a yellow oil (485 mg, 2.28 mmol, 71%).

^{1}H NMR (500 MHz, $CDCl_3$) δ 8.19 (dd, J = 4.7, 1.9 Hz, 1H), 7.39 (ddd, J = 8.7, 7.0, 1.9 Hz, 1H), 7.31 (t, J = 7.4 Hz, 2H), 7.27 – 7.26 (m, 1H), 7.26 – 7.22 (m, 2H), 6.57 – 6.51 (m, 1H), 6.46 (d, J = 8.7 Hz, 1H), 3.60 (q, J = 7.1 Hz, 2H), 1.19 (t, J = 7.1 Hz, 4H). **^{13}C NMR** (126 MHz, $CDCl_3$) δ 158.2, 148.2, 139.2, 137.3, 128.6, 127.0, 126.9, 111.7, 105.9, 51.0, 42.9, 12.5.

The data agree with the literature.[26]

N-Methyl-N-phenyl-2-aminopyridine (1j)

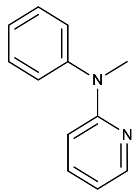

1j

Following the general procedure for the Buchwald-Hartwig coupling, using 2-bromypyridine (0.31 mL, 3.2 mmol, 1.0 eq) and N-methylaniline (0.38 mL, 3.5 mmol, 1.1 eq). The crude was purified by silica gel column chromatography (eluting with 0% to 20% Et_2O in petroleum ether) to afford the title compound **1j** as a yellow oil (388 mg, 2.11 mmol, 66%).

^{1}H NMR (700 MHz, $CDCl_3$) δ 8.23 (ddd, J = 5.2, 2.0, 1.0 Hz, 1H), 7.51 – 7.35 (m, 2H), 7.31 (ddd, J = 8.7, 7.0, 2.0 Hz, 1H), 7.28 – 7.27 (m, 1H), 7.26 – 7.25 (m, 1H), 7.22 (td, J = 7.3, 1.3 Hz, 1H), 6.61 (ddd, J = 7.0, 5.2, 1.0 Hz, 1H), 6.53 (dt, J = 8.7, 1.0 Hz, 1H), 3.49 (s, 3H). **^{13}C NMR** (176 MHz, $CDCl_3$) δ 158.9, 147.7, 146.9, 136.8, 129.9, 126.5, 125.6, 113.2, 109.4, 38.6.

The data agree with the literature.[27]

_N_-Methyl-_N_-(4-(trifluoromethyl)phenyl)-2-aminopyridine (1k)

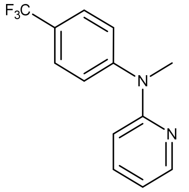

1k

Under a dry, inert atmosphere, to a cooled (0 °C) solution of *N*-(4-(trifluoromethyl)phenyl)pyridin-2-amine (224 mg, 0.94 mmol, 1.0 eq) in anhydrous THF (4 mL) was added KHMDS (1.1 mL, 1.1 mmol, 1.0 M in THF, 1.2 eq) dropwise and mixture was stirred at room temperature for 1 h. The reaction mixture was then cooled to 0 °C and methyl iodide (70 µL, 1.1 mmol, 1.2 eq) was added. The resulting mixture was stirred overnight at room temperature, after which it was concentrated under reduced pressure and diluted with DCM and saturated aqueous solution of ammonium chloride. The layers were then separated and the aqueous layer was extracted twice with DCM. The combined organic layer was dried over sodium sulfate and concentrated under reduced pressure. The crude mixture was purified by silica gel column chromatography (eluting with 0% to 10% EtOAc in petroleum ether) to afford the title compound **1k** as a yellow oil (199 mg, 0.79 mmol, 84%).

^{1}H NMR (500 MHz, $CDCl_3$) δ 8.29 (ddd, *J* = 5.0, 2.0, 0.9 Hz, 1H), 7.64 – 7.57 (m, 2H), 7.43 (ddd, *J* = 8.5, 7.2, 2.0 Hz, 1H), 7.38 – 7.30 (m, 2H), 6.79 (dt, *J* = 8.5, 0.9 Hz, 1H), 6.75 (ddd, *J* = 7.2, 5.0, 0.9 Hz, 1H), 3.52 (s, 3H). **^{13}C NMR** (126 MHz, $CDCl_3$) δ 158.3, 150.1, 148.3, 137.3, 126.7 (q, $^3J_{C\text{-}F}$ = 3.8 Hz), 125.7 (q, $^2J_{C\text{-}F}$ = 32.7 Hz), 124.2 (q, $^1J_{C\text{-}F}$ = 271.5 Hz), 123.9, 115.3, 111.0, 38.3. **^{19}F NMR** (471 MHz, $CDCl_3$) δ –63.1.

The data agree with the literature.[28]

_N_-(4-(Trifluoromethyl)phenyl)pyridin-2-amine (1ka)

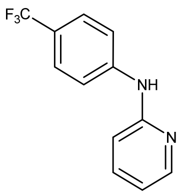

1ka

Under a dry, inert atmosphere, a vial was charged with 2-aminopyridine (282 mg, 3.00 mmol, 1.0 eq), (4-(trifluoromethyl)phenyl)boronic acid (570 mg, 3.00 mmol, 1.0 eq), $Cu(OAc)_2$ (654 mg, 3.60 mmol, 1.2 eq), K_3PO_4 (1.27 g, 6.00 mmol, 2.0 eq) and DMSO (5 mL). The reaction mixture was stirred at 120 °C for 24 h, after which it was cooled to room temperature and extracted three times with EtOAc. The organic layer was washed with brine, dried over sodium sulfate, and concentrated *in vacuo*. The crude mixture was purified by silica gel column chromatography (eluting with 0% to 20% EtOAc in petroleum ether) to afford the title compound **1ka** as a white solid (224 mg, 0.94 mmol, 31%).

^{1}H NMR (400 MHz, $CDCl_3$) δ 8.27 (dd, *J* = 5.0, 1.9 Hz, 1H), 7.60 – 7.52 (m, 3H), 7.49 (d, *J* = 8.6 Hz, 2H), 6.89 (d, *J* = 8.3 Hz, 1H), 6.84 (dd, *J* = 7.2, 5.0 Hz, 1H), 6.75 (br. s, 1H). **^{13}C NMR** (101 MHz, $CDCl_3$) δ 154.8, 148.5, 144.0, 138.0, 126.6 (q, *J* = 3.8 Hz), 124.4 (q, *J* = 271.5 Hz), 123.5 (q, *J* = 31.1 Hz), 118.2, 116.4, 110.0. **^{19}F NMR** (471 MHz, $CDCl_3$) δ –62.8.

The data agree with the literature.[29]

N-(2-((tert-Butyldimethylsilyl)oxy)ethyl)-N-methylpyridin-2-amine (1l)

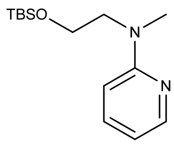

1l

tert-Butyldimethyldilyl trifluoromethanesulfonate (0.906 mL, 3.94 mmol, 1.2 eq) was added dropwise to a mixture of 2-(methyl-2-pyridylamino)ethanol (0.500 g, 3.29 mmol, 1 eq), 2,6-lutidine (0.574 mL, 4.93 mmol, 1.5 eq) in anhydrous DCM (20 mL) at 0 °C. The reaction mixture was stirred at 0 °C for 1 h and was quenched by addition of water. The organic and aqueous layer were separated and the aqueous phase was further extracted with DCM. The combined organic layers were dried over $MgSO_4$, filtered, and concentrated *in vacuo*. The crude mixture was purified by silica gel chromatography (eluting with 5% Et_2O in petroleum ether) to afford the title compound **1l** as a colorless liquid (0.747 g, 2.80 mmol, 85%).

^{1}H NMR (500 MHz, $CDCl_3$) δ 8.14 – 8.11 (m, 1H), 7.42 (ddd, *J* = 8.5, 7.2, 2.0 Hz, 1H), 6.52 – 6.50 (m, 1H), 6.49 (s, 1H), 3.80 (t, *J* = 6.2 Hz, 2H), 3.67 (t, *J* = 5.8 Hz, 2H), 3.10 (s, 3H), 0.87 (s, 9H), 0.01 (s, 6H). **^{13}C NMR** (126 MHz, $CDCl_3$) δ 158.6, 147.9, 137.2, 111.4, 105.9, 61.3, 52.7, 37.8, 26.0, 18.4, –5.3. **HRMS** m/z: $[M + H]^+$ calc'd for $[C_{14}H_{27}N_2OSi]^+$ expect 267.1887; found 267.1900.

2-(Azetidin-1-yl)pyridine (1m)

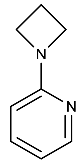

1m

Following the general procedure for the Buchwald-Hartwig coupling, using 2-bromypyridine (0.31 mL, 3.2 mmol, 1.0 eq), azetidine hydrochloride (330 mg, 3.5 mmol, 1.1 eq) and NaO*t*Bu (1.17 g, 12.2 mmol, 3.8 eq). The crude was purified by silica gel column chromatography (eluting with 0% to 100% EtOAc in petroleum ether) to afford the title compound **1m** as a yellow oil (64 mg, 0.48 mmol, 15%).

^{1}H NMR (500 MHz, $CDCl_3$) δ 8.14 (ddd, *J* = 5.1, 1.9, 1.0 Hz, 1H), 7.42 (ddd, *J* = 8.4, 7.2, 1.9 Hz, 1H), 6.57 (ddd, *J* = 7.2, 5.1, 1.0 Hz, 1H), 6.26 (dt, *J* = 8.4, 1.0 Hz, 1H), 4.07 – 4.00 (m, 4H), 2.43 – 2.34 (m, 2H). **^{13}C NMR** (126 MHz, $CDCl_3$) δ 161.1, 148.3, 137.1, 112.6, 105.8, 50.9, 16.8.

The data agree with the literature.[30]

3-(Pyrrolidin-1-yl)pyridine (1n)

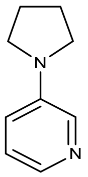

1n

Under a dry, inert atmosphere, sodium *tert*-butoxide (1.1 g, 11 mmol, 3 eq), XPhos (9 mg, 0.019 mmol, 5 mol%), palladium(II) acetate (4 mg, 0.019 mmol, 2.5 mol%), pyrrolidine (0.79 mL, 9.5 mmol, 2.5 eq), and 3-bromopyridine (0.60 g, 3.8 mmol, 1.0 eq) were added to a reaction vial. The vial was evacuated and refilled with nitrogen three times. Anhydrous toluene (5-10 mL) was added and the reaction was stirred overnight at 100 °C. The reaction mixture was cooled to room temperature and filtered through celite. The mixture was then concentrated *in vacuo* and purified by silica gel column chromatography (eluting with 0% to 20% EtOAc in *n*-hexane) to afford the desired product **1n** as a yellow oil (0.29 g, 2.0 mmol, 52%).

^{1}H NMR (400 MHz, $CDCl_3$) δ 7.98 (s, 1H), 7.92 (d, *J* = 4.1 Hz, 1H), 7.10 (dd, *J* = 8.3, 4.6 Hz, 1H), 6.82 – 6.77 (m, 1H), 3.32 – 3.26 (m, 4H), 2.06 – 1.98 (m, 4H). **^{13}C NMR** (101 MHz, $CDCl_3$) δ 143.9, 137.0, 134.4, 123.7, 117.9, 47.4, 25.5.

X-ray structure of complex between 2-(pyrrolidin-1-yl)pyridine **1a**, (*E*)-*N*-phenylbut-2-enamide **4d** and (*R*)-TRIP

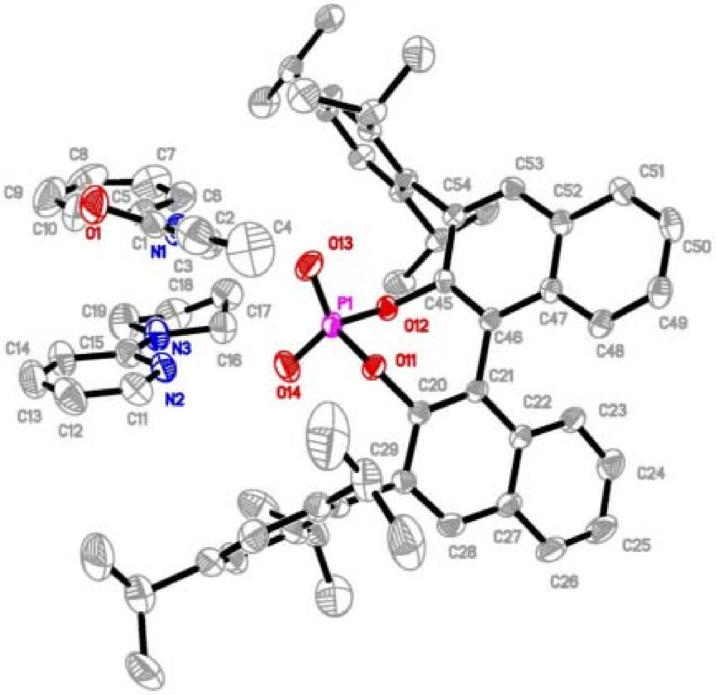

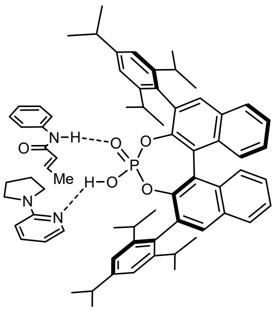

X-Ray Crystallography Sample Preparation

(*E*)-*N*-Phenylbut-2-enamide **4d** (8.7 mg, 0.054 mmol, 1.00 eq), 2-(pyrrolidin-1-yl)pyridine **1a** (20 mg, 0.13, 2.50 eq) and (R)-TRIP (51 mg, 0.067 mmol, 1.25 eq) were dissolved in a mixture of DCM : *n*-hexane and was then crystallized via vapour diffusion to yield colourless crystals which were analysed by x-ray diffraction. The structure was deposited in the Cambridge Crystallographic Data Centre (deposition no.: CCDC 2183883).

Stern-Volmer quenching studies

Stern-Volmer quenching was performed using a Shimadzu RF-6000 spectrofluorometer. Experiments were recorded using a quartz cell equipped with septa-lined screw cap under nitrogen.

Stern-Volmer studies were conducted with starting materials amine **1a** and amide **4b**, diastereomers **5ba** and **5bb** of the reaction product, the 2,4,6-triisopropylbenzenethiol, and the chiral phosphoric acid catalyst (*R*)-TRIP and its corresponding tetrabutylammonium salt TBA-(*R*)-TRIP. Standard solutions of these substrates and (Ir[dF(CF_3)ppy]$_2$(dtbpy))PF_6 were prepared in dry degassed trifluorotoluene. Solutions containing 0.01 mM photocatalyst and varying concentrations of quencher were made up in a quartz cuvette and purged with nitrogen for 5 minutes before irradiating. The samples were irradiated at 465 nm and luminescence was measured at 500 nm. Individual data points represent an average of two readings.

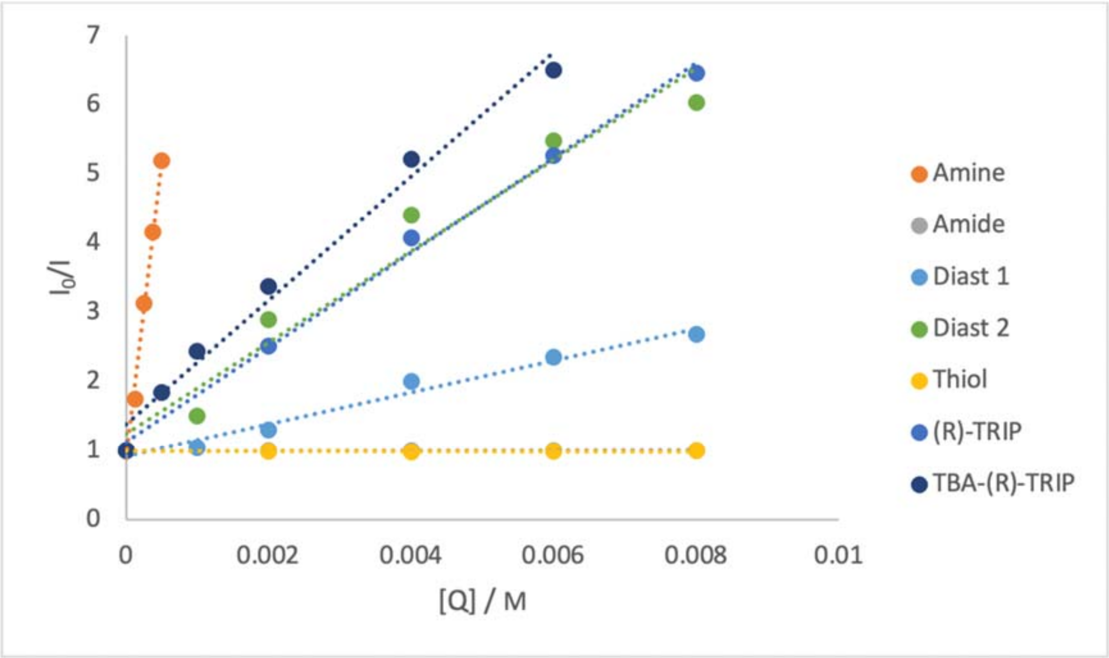

Figure 9. Stern-Volmer plot of (Ir[dF(CF_3)ppy]$_2$(dtbpy))PF_6 (0.01 x 10^{-3} M) in trifluorotoluene at variable concentrations of quenchers.

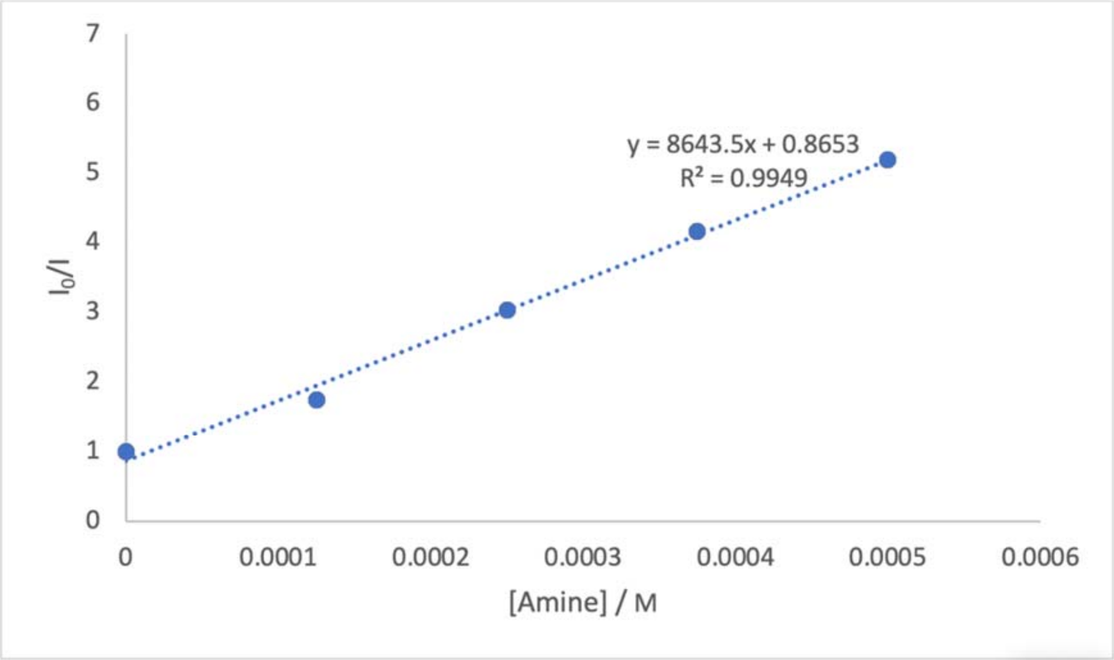

*Figure 10. Stern-Volmer plot of (Ir[dF(CF_3)ppy]$_2$(dtbpy))PF_6 (0.01 x 10^{-3} M) in trifluorotoluene at variable concentrations of amine **1a**.*

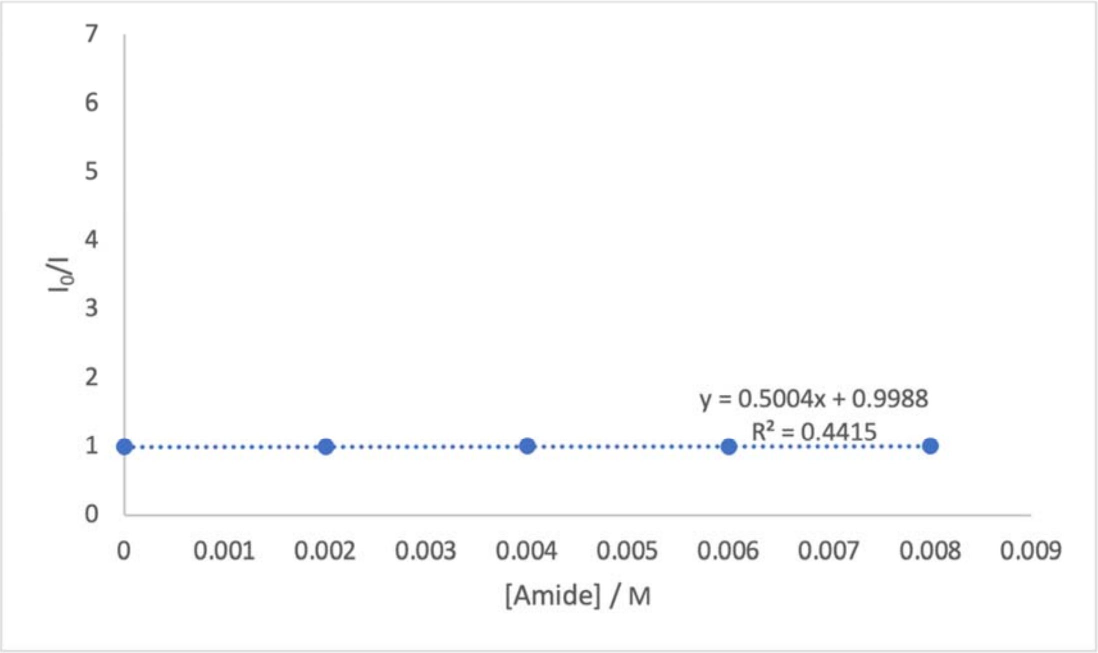

*Figure 11. Stern-Volmer plot of (Ir[dF(CF_3)ppy]$_2$(dtbpy))PF_6 (0.01 x 10^{-3} M) in trifluorotoluene at variable concentrations of amide **4b**.*

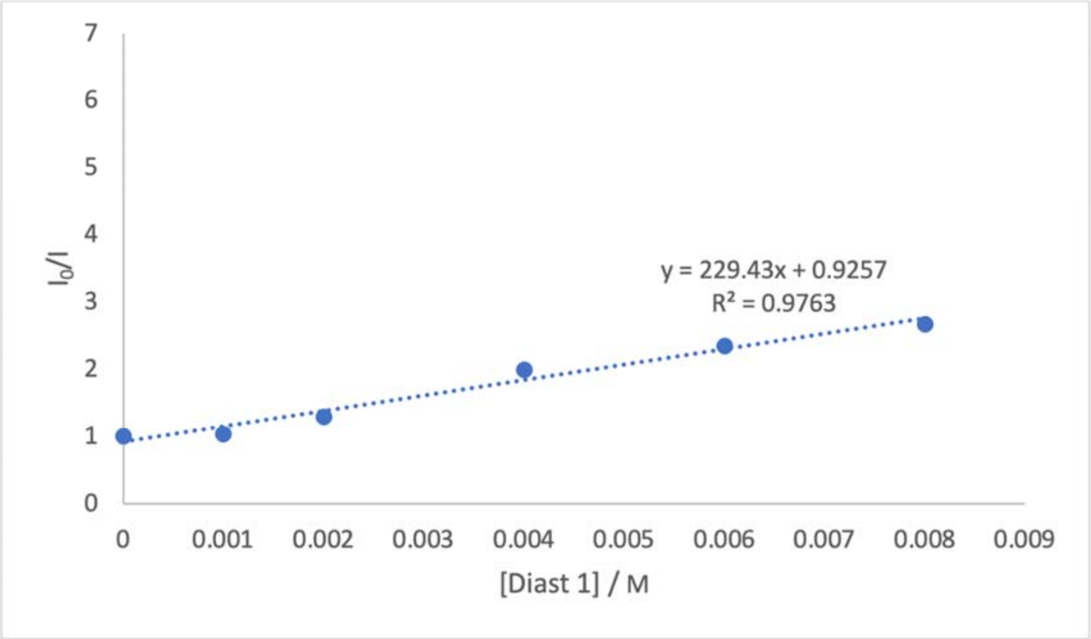

*Figure 12. Stern-Volmer plot of (Ir[dF(CF$_3$)ppy]$_2$(dtbpy))PF$_6$ (0.01 x 10^{-3} M) in trifluorotoluene at variable concentrations of product diastereomer **5ba**.*

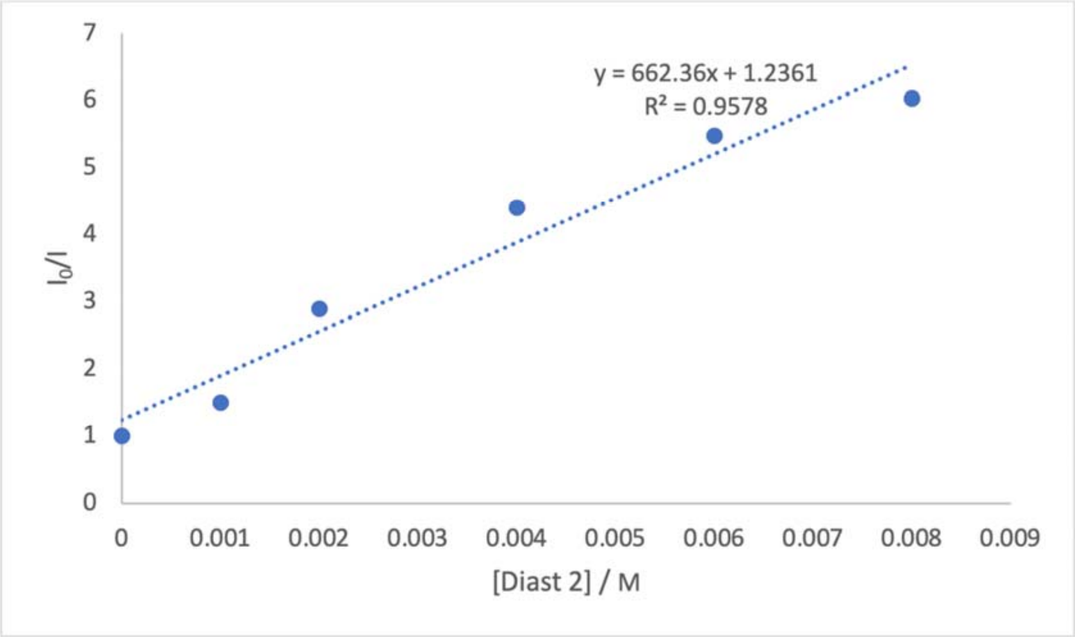

*Figure 13. Stern-Volmer plot of (Ir[dF(CF$_3$)ppy]$_2$(dtbpy))PF$_6$ (0.01 x 10^{-3} M) in trifluorotoluene at variable concentrations of product diastereomer **5bb**.*

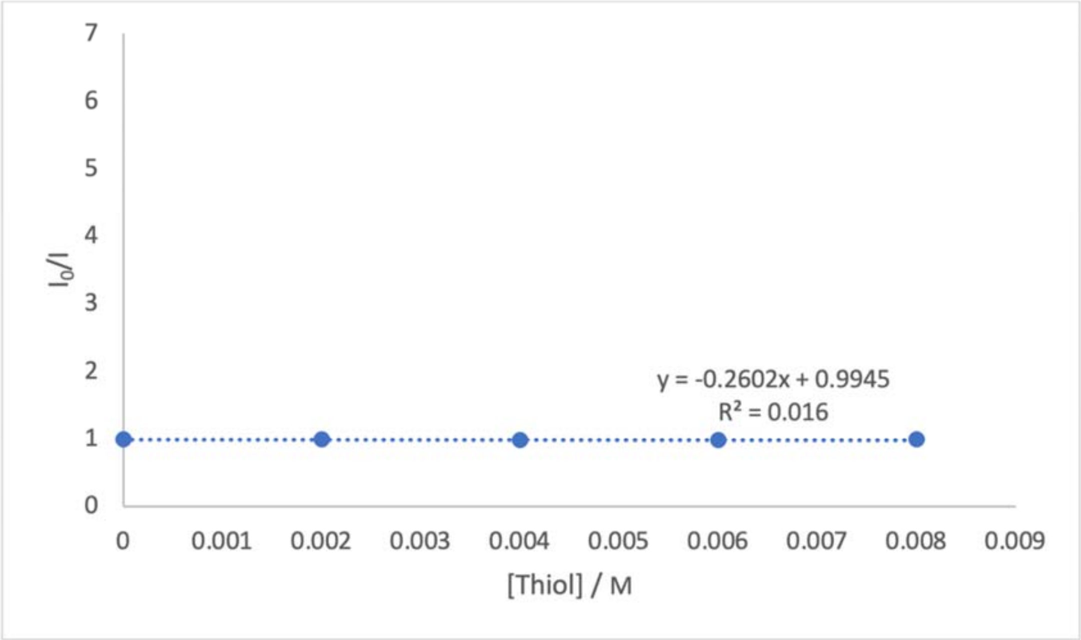

Figure 14. Stern-Volmer plot of (Ir[dF(CF$_3$)ppy]$_2$(dtbpy))PF$_6$ (0.01 x 10^{-3} M) in trifluorotoluene at variable concentrations of 2,4,6-triisopropylbenzenethiol.

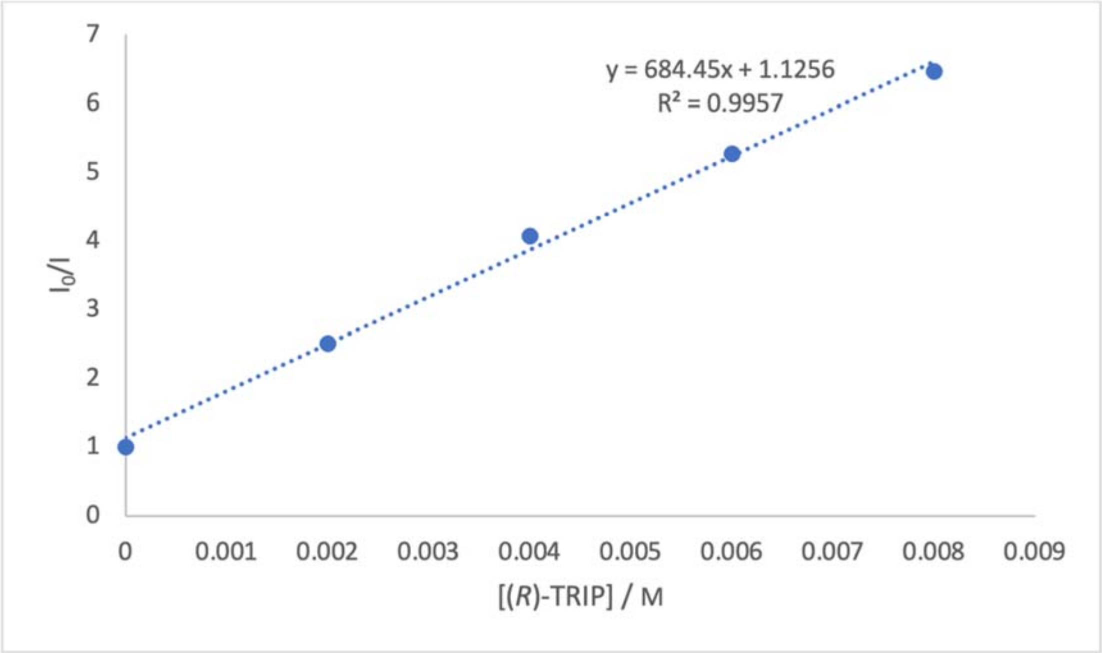

Figure 15. Stern-Volmer plot of (Ir[dF(CF$_3$)ppy]$_2$(dtbpy))PF$_6$ (0.01 x 10^{-3} M) in trifluorotoluene at variable concentrations of (R)-TRIP.

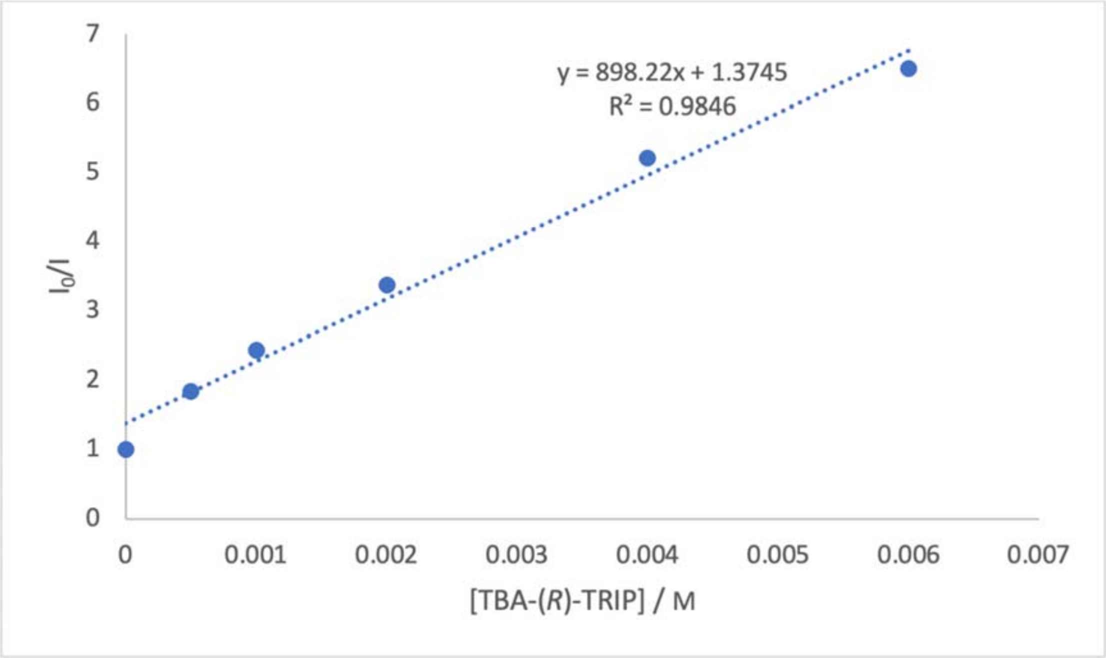

Figure 16. Stern-Volmer plot of (Ir[dF(CF_3)ppy]$_2$(dtbpy))PF_6 (0.01 x 10^{-3} M) in trifluorotoluene at variable concentrations of TBA-(R)-TRIP.

Chiral SFC, HPLC and GC traces

_N_-Phenyl-3-(1-(pyridin-2-yl)pyrrolidin-2-yl)propanamide (5a)

Chiral SFC Analysis CHIRAL ART SC (CO_2: MeOH, 80:20, 2.5 mL min^{-1}, 40 °C, 244 nm) indicated 84% ee, tR = 8.3 (major), 8.7 (minor) minutes.

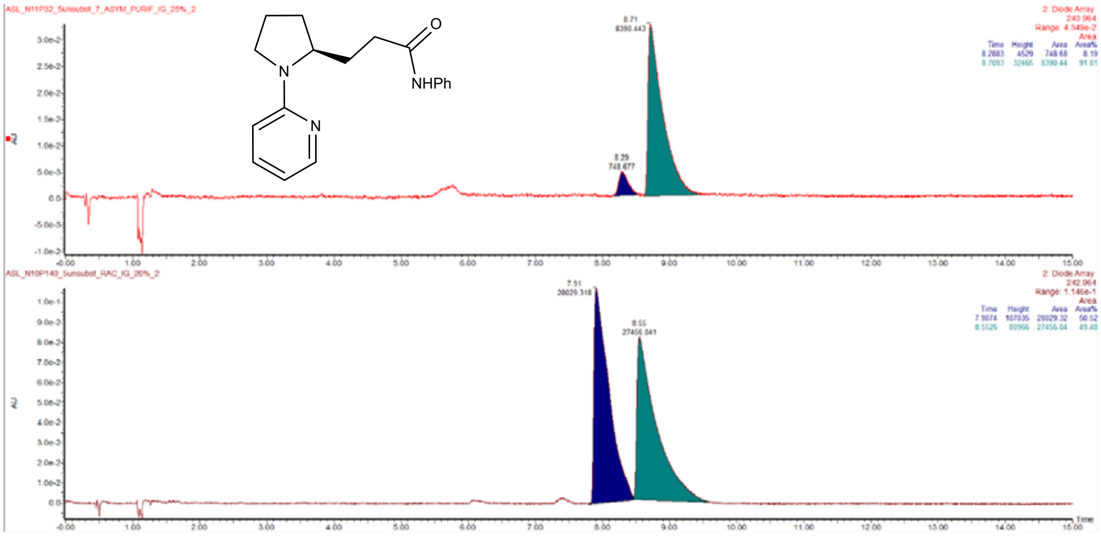

(*S*)-2-Benzyl-*N*-phenyl-3-((*S*)-1-(pyridin-2-yl)pyrrolidin-2-yl)propanamide (5ba)

Chiral SFC Analysis CHIRAL ART SC (CO_2: MeOH, 80:20, 2.5 mL min^{-1}, 40 °C, 244 nm) indicated 90% ee, tR = 5.0 (major), 5.8 (minor) minutes.

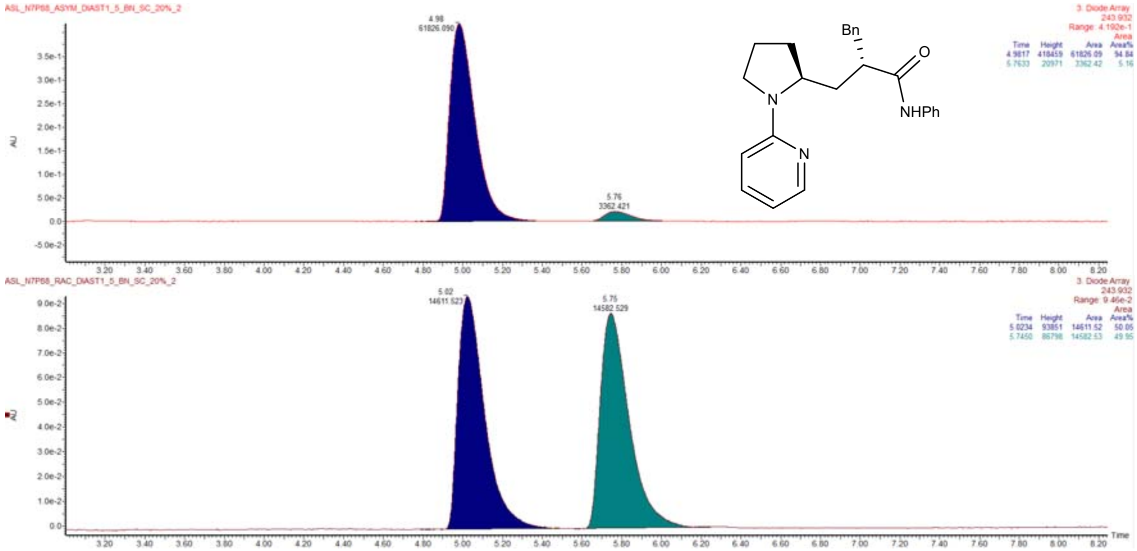

(*R*)-2-Benzyl-*N*-phenyl-3-((*S*)-1-(pyridin-2-yl)pyrrolidin-2-yl)propanamide (5bb)

Chiral SFC Analysis CHIRAL ART SC (CO_2: MeOH, 80:20, 2.5 mL min^{-1}, 40 °C, 244 nm) indicated 94% ee, tR = 6.7 (major), 8.1 (minor) minutes.

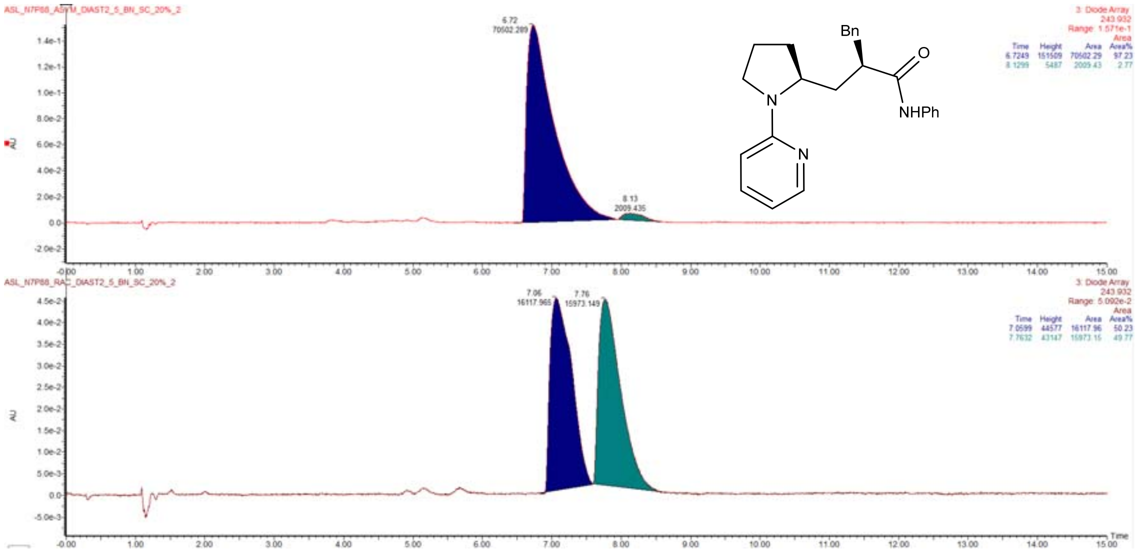

(*S*)-3-Methyl-*N*-phenyl-2-(((*S*)-1-(pyridin-2-yl)pyrrolidin-2-yl)methyl)butanamide (5ca)

Chiral SFC Analysis CHIRALPAK IG (CO_2: MeOH, 85:15, 2.5 mL min^{-1}, 40 °C, 244 nm) indicated 84% ee, tR = 6.6 (minor), 8.5 (major) minutes.

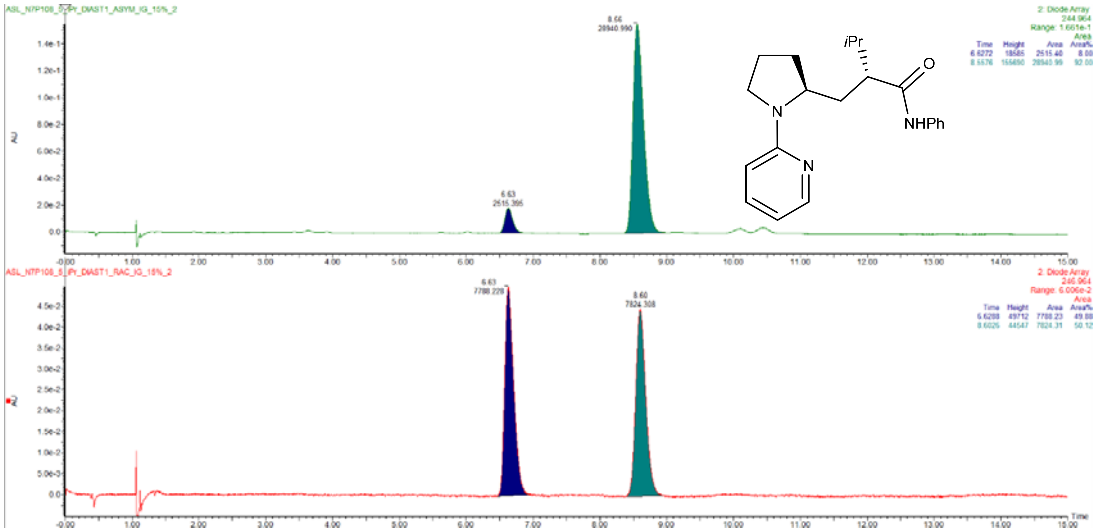

(*S*)-3-Methyl-*N*-phenyl-2-(((*S*)-1-(pyridin-2-yl)pyrrolidin-2-yl)methyl)butanamide (5cb)

SFC Analysis CHIRALPAK IG (CO_2: MeOH, 80:20, 2.5 mL min^{-1}, 40 °C, 244 nm) indicated 93% ee, tR = 4.2 (minor), 5.8 (major) minutes.

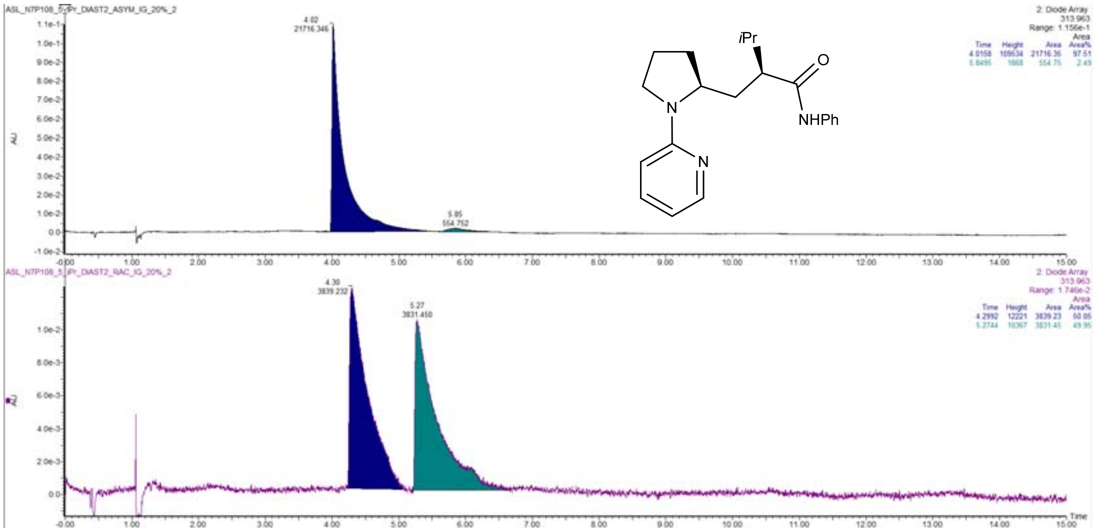

Methyl (*R*)-4-oxo-4-(phenylamino)-3-(((*S*)-1-(pyridin-2-yl)pyrrolidin-2-yl)methyl)butanoate (5da)

Chiral SFC Analysis: CHIRAL ART SC (CO_2: MeOH, 80:20, 2.5 mL min^{-1}, 40 °C, 243 nm) indicated 84% ee, tR = 5.2 (major), 8.5 (minor) minutes.

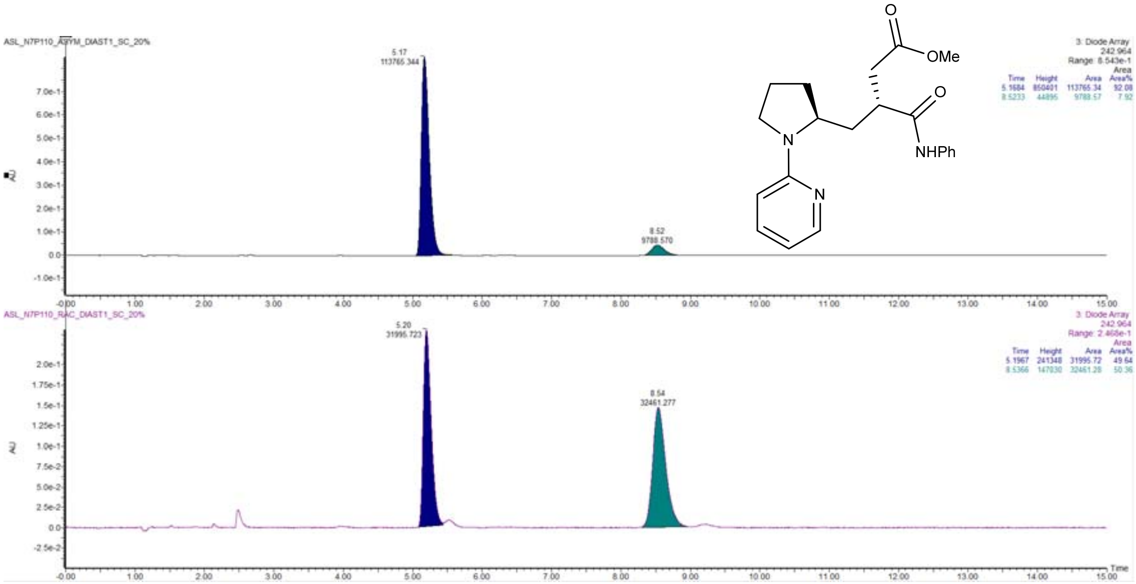

Methyl (*S*)-4-oxo-4-(phenylamino)-3-(((*S*)-1-(pyridin-2-yl)pyrrolidin-2-yl)methyl)butanoate (5db)

Chiral SFC Analysis: CHIRAL ART SC (CO_2: MeOH, 80:20, 2.5 mL min^{-1}, 40 °C, 241 nm) indicated 86% ee, tR = 6.2 (major), 7.7 (minor) minutes.

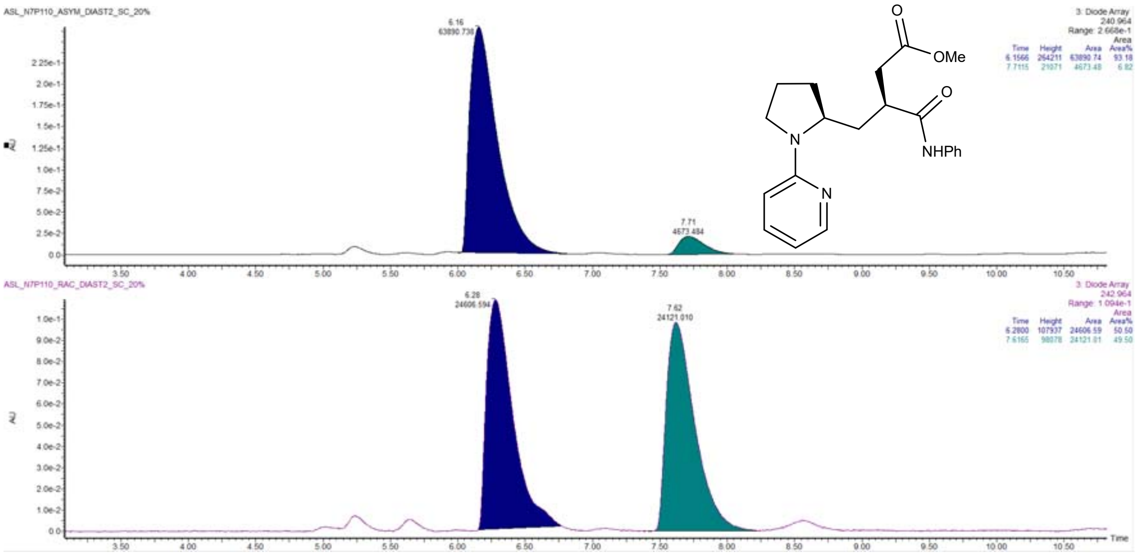

(*S*)-*N*-Phenyl-2-(((*S*)-1-(pyridin-2-yl)pyrrolidin-2-yl)methyl)butanamide (5ea)

Chiral SFC Analysis: CHIRAL ART IG (CO_2: MeOH, 75:25, 2.5 mL min^{-1}, 40 °C, 244 nm) indicated 94% ee, tR = 4.7 (minor), 6.2 (major) minutes.

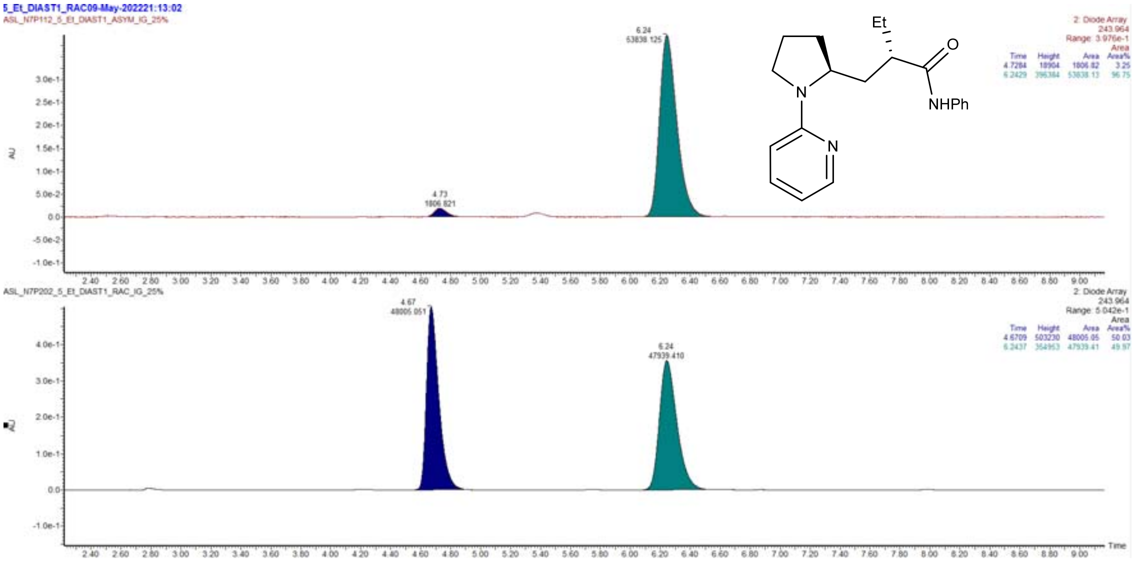

(*R*)-*N*-phenyl-2-(((*S*)-1-(pyridin-2-yl)pyrrolidin-2-yl)methyl)butanamide (5eb)

Chiral SFC Analysis: CHIRAL ART SC (CO_2: MeOH, 85:15, 2.5 mL min^{-1}, 40 °C, 244 nm) indicated 90% ee, tR = 5.7 (major), 6.7 (minor) minutes.

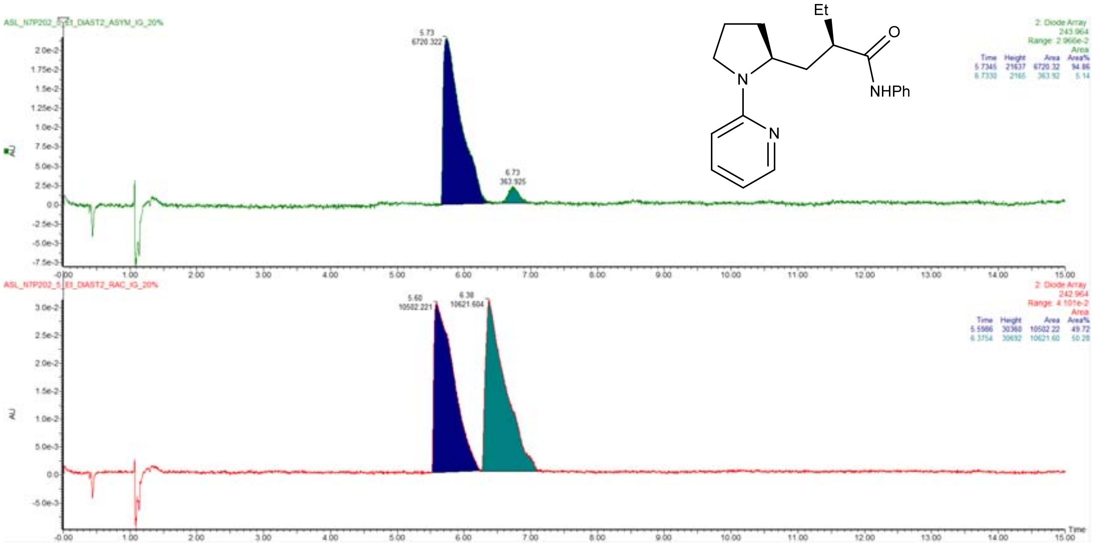

(*S*)-*N*-Phenyl-2-(((*S*)-1-(pyrimidin-2-yl)pyrrolidin-2-yl)methyl)butanamide (5fa)

Chiral SFC Analysis CHIRALPAK IG (CO_2: MeOH, 85:15, 2.5 mL min^{-1}, 40 °C, 243 nm) indicated 90% ee, tR = 8.6 (minor), 9.8 (major) minutes.

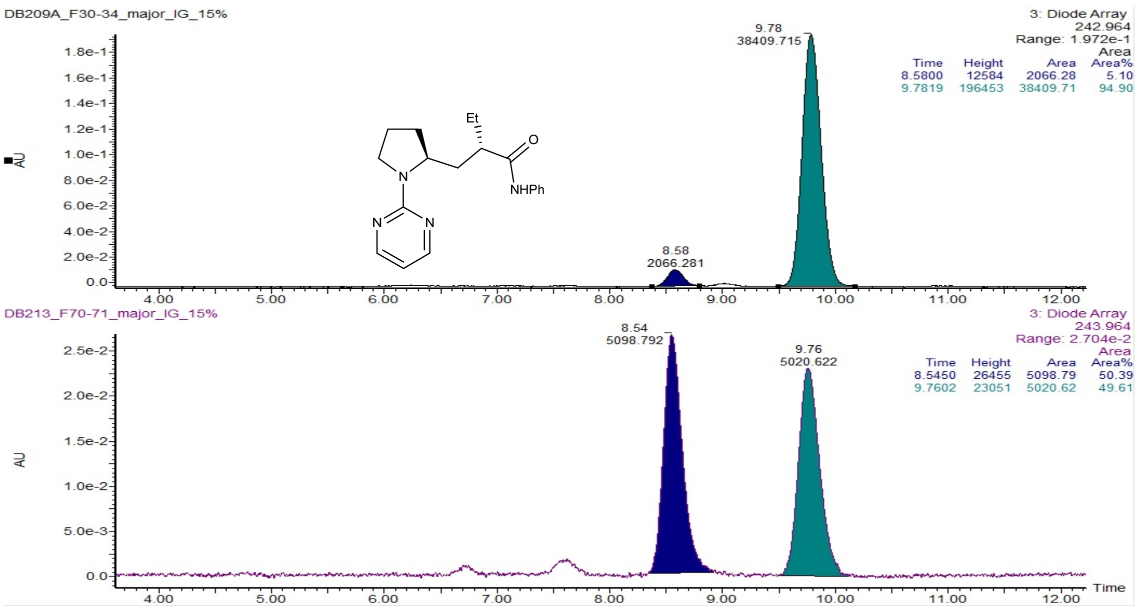

(*R*)-*N*-Phenyl-2-(((*S*)-1-(pyrimidin-2-yl)pyrrolidin-2-yl)methyl)butanamide (5fb)

Chiral SFC Analysis CHIRALPAK IG (CO_2: MeOH, 85:15, 2.5 mL min^{-1}, 40 °C, 243 nm) indicated 76% ee, tR = 8.9 (major), 10.9 (minor) minutes.

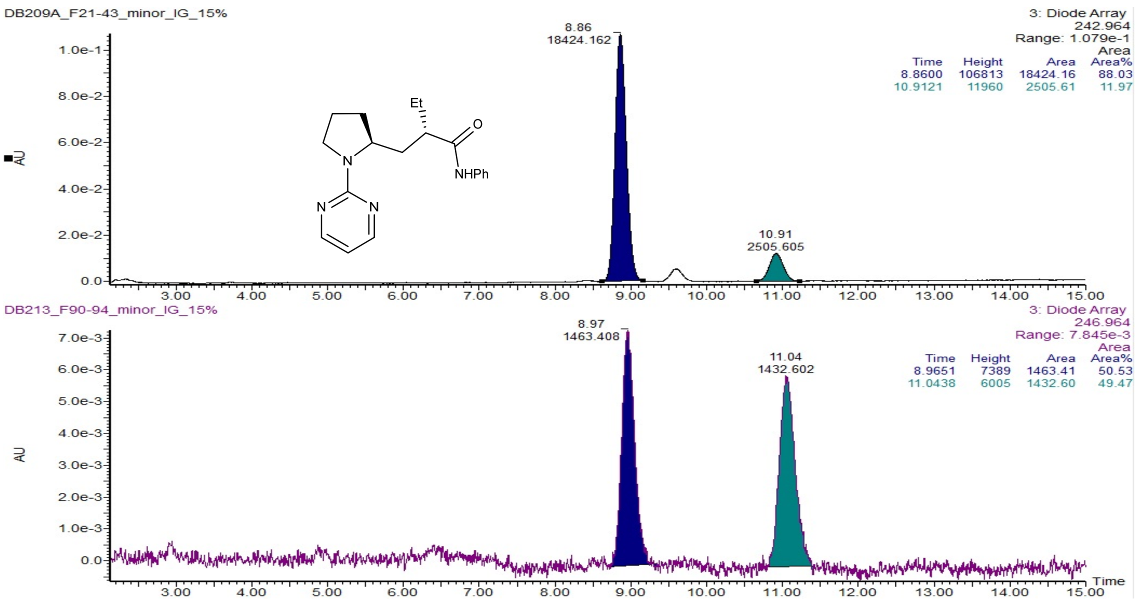

(*S*)-*N*-Phenyl-2-(((*S*)-1-(pyridin-2-yl)piperidin-2-yl)methyl)butanamide (5ga)

Chiral SFC Analysis CHIRALPAK IG (CO_2: MeOH, 80:20, 2.5 mL min^{-1}, 40 °C, 244 nm) indicated 78% ee, tR = 4.8 (minor), 8.0 (major) minutes.

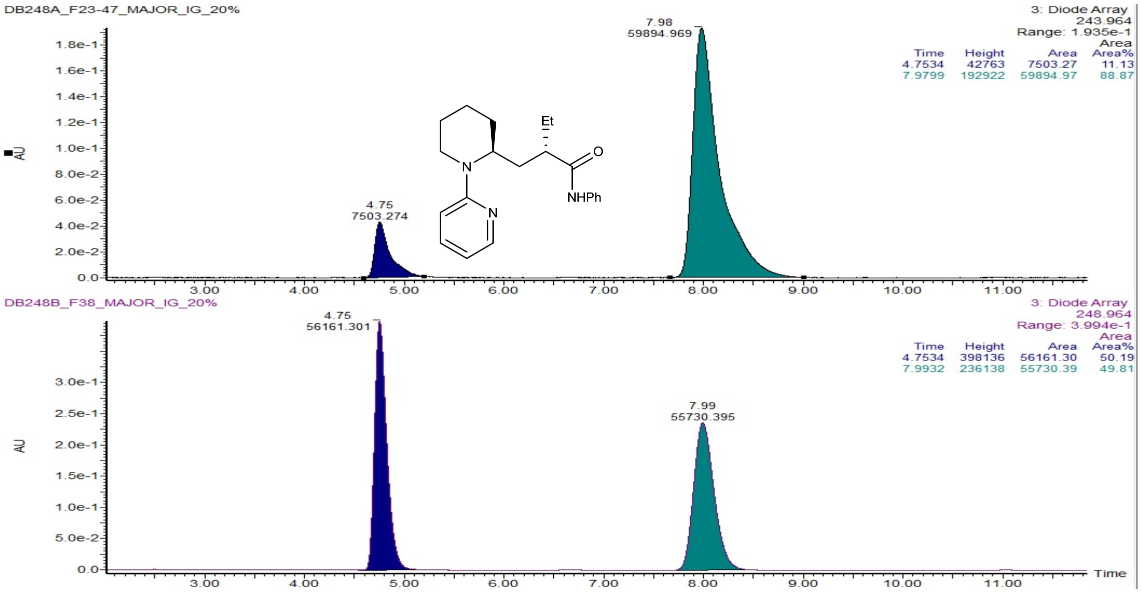

(*R*)-*N*-Phenyl-2-(((*S*)-1-(pyridin-2-yl)piperidin-2-yl)methyl)butanamide (5gb)

Chiral SFC Analysis CHIRALPAK IG (CO_2: MeOH, 80:20, 2.5 mL min^{-1}, 40 °C, 250 nm) indicated 80% ee, tR = 4.6 (major), 5.2 (minor) minutes.

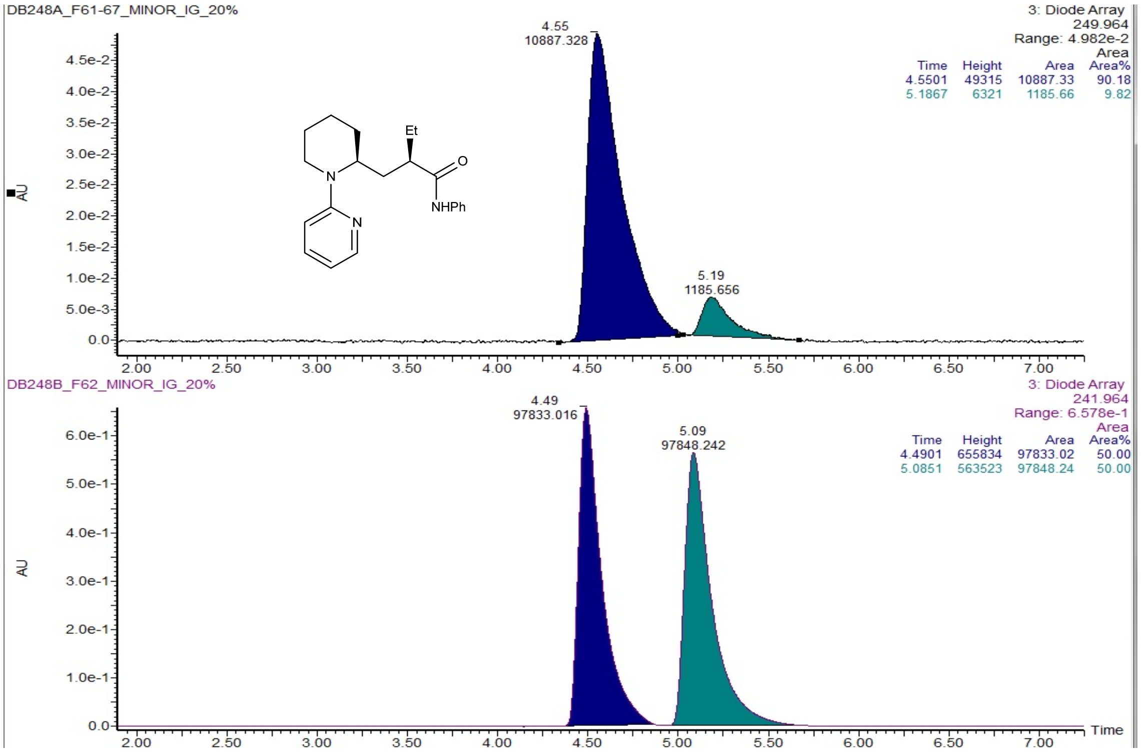

(*S*)-2-Benzyl-*N*-phenyl-3-((*S*)-1-(pyridin-2-yl)piperidin-2-yl)propanamide (5ha)

Chiral SFC Analysis: CHIRALPAK IG (CO_2: MeOH, 75:25, 2.5 mL min^{-1}, 40 °C, 245 nm) indicated 86% ee, tR = 6.5 (minor), 7.6 (major) minutes.

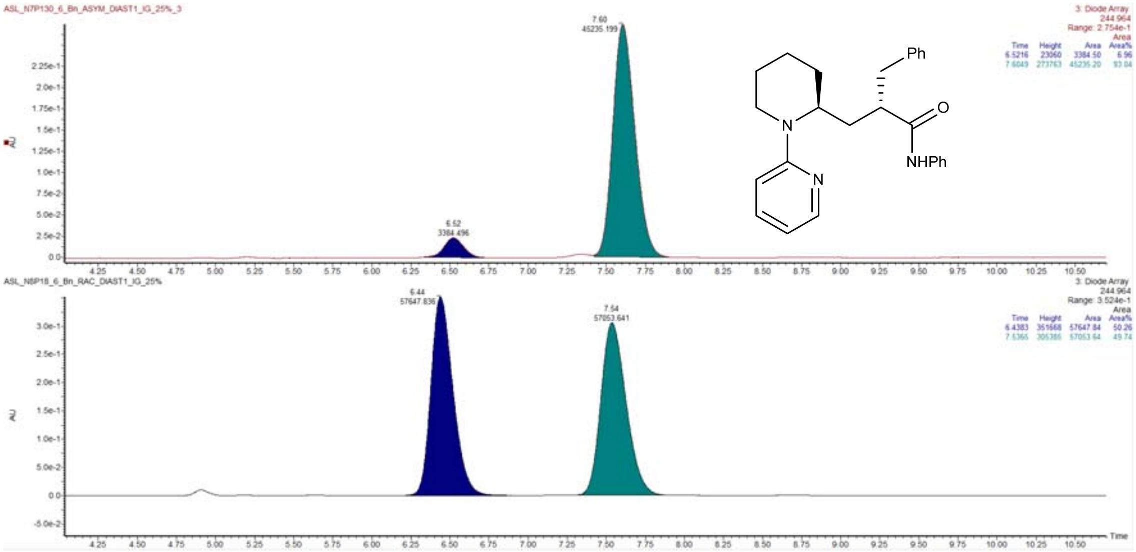

(*R*)-2-Benzyl-*N*-phenyl-3-((*S*)-1-(pyridin-2-yl)piperidin-2-yl)propanamide (5hb)

Chiral SFC Analysis: CHIRALPAK IG (CO_2: MeOH, 75:25, 2.5 mL min^{-1}, 40 °C, 245 nm) indicated 86% ee, tR = 5.6 (minor), 6.4 (major) minutes.

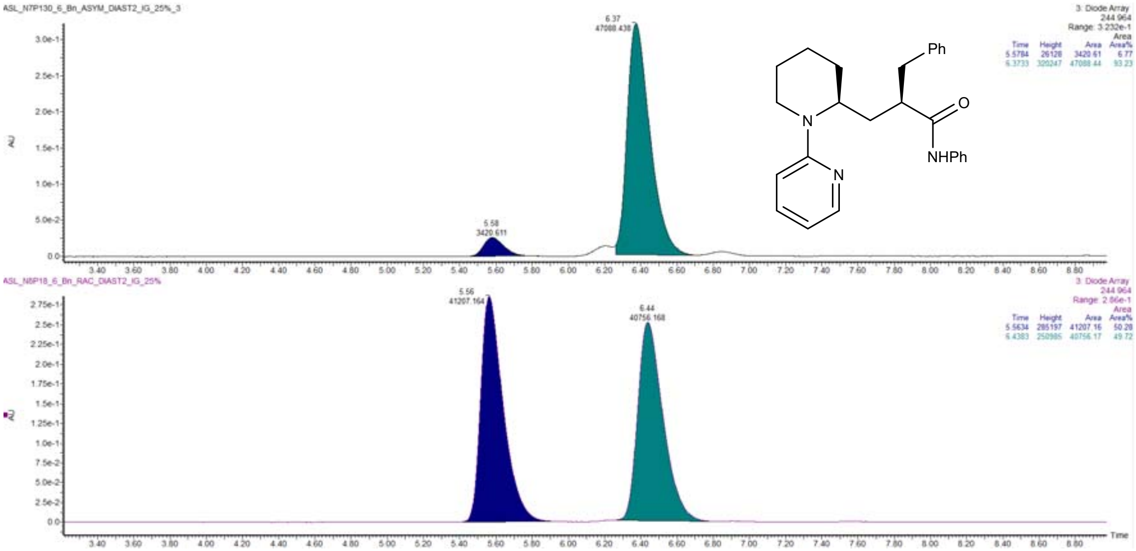

(*S*)-*N*-Phenyl-2-(((*S*)-1-(pyridin-2-yl)azepan-2-yl)methyl)butanamide (5ia)

SFC Analysis CHIRALPAK IG (CO_2: MeOH, 75:25, 2.5 mL min^{-1}, 40 °C, 245 nm) indicated 86% ee, tR = 4.85 (minor), 13.5 (major) minutes.

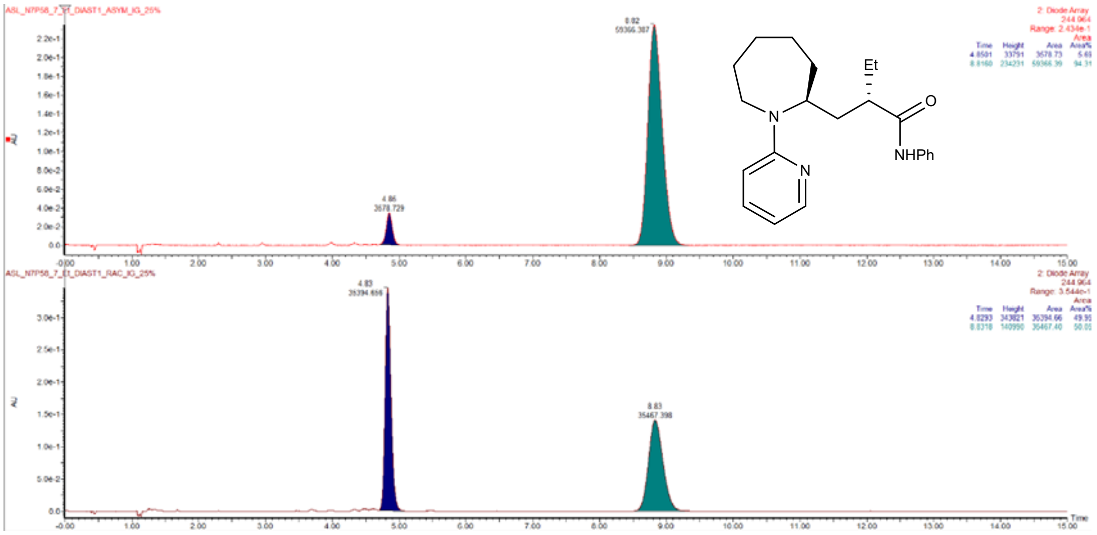

(*R*)-*N*-phenyl-2-(((*S*)-1-(pyridin-2-yl)azepan-2-yl)methyl)butanamide (5ib)

Chiral SFC Analysis: CHIRALPAK IG (CO_2: MeOH, 80:20, 2.5 mL min^{-1}, 40 °C, 245 nm) indicated 97% ee, tR = 4.6 (minor), 5.5 (major) minutes.

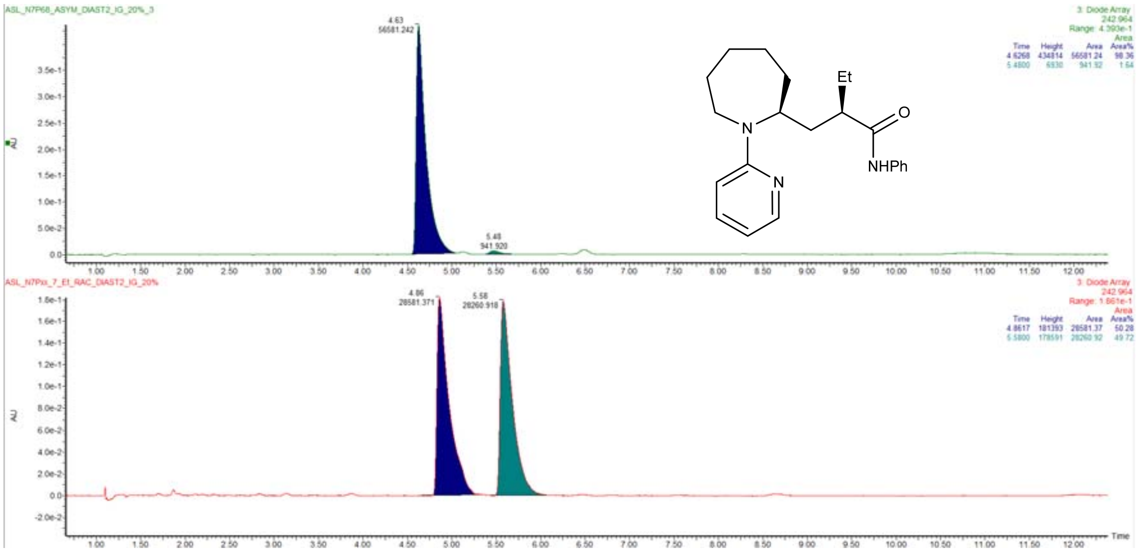

(*S*)-2-Benzyl-*N*-phenyl-3-((*S*)-1-(pyridin-2-yl)azepan-2-yl)propanamide (5ja),

Chiral SFC Analysis: CHIRAL ART SC (CO_2: MeOH, 80:20, 2.5 mL min^{-1}, 40 °C, 244 nm) indicated 92% ee, tR = 5.2 (major), 5.6 (minor) minutes.

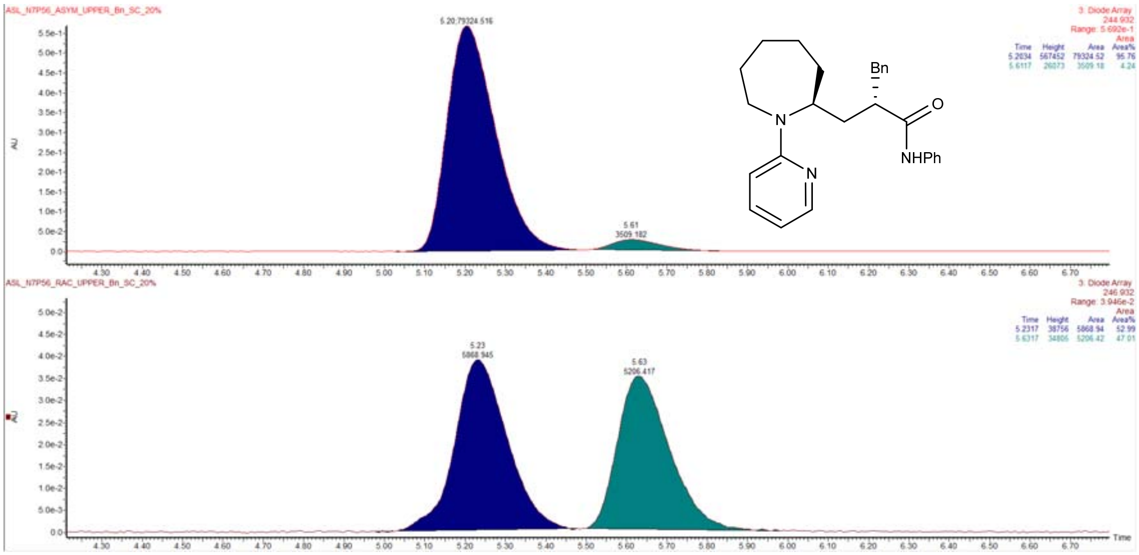

(*R*)-2-Benzyl-*N*-phenyl-3-((*S*)-1-(pyridin-2-yl)azepan-2-yl)propanamide (5jb)

Chiral SFC Analysis: CHIRAL ART SJ (CO_2: MeOH, 80:20, 2.5 mL min^{-1}, 40 °C, 244 nm) indicated 97% ee, tR = 4.5 (minor), 5.8 (minor) minutes.

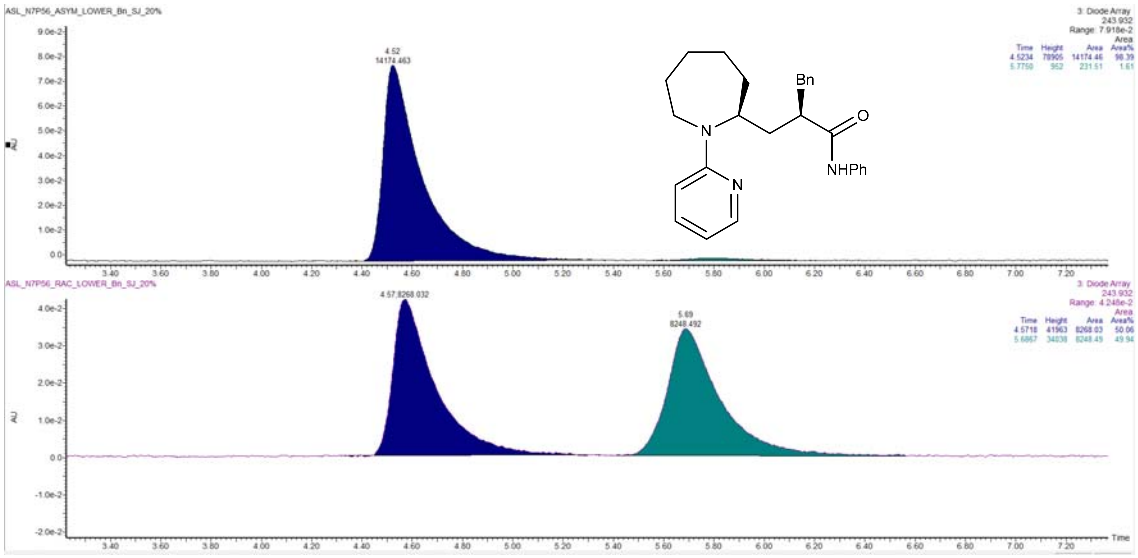

(*R*)-3-Methyl-*N*-phenyl-2-(((*S*)-1-(pyridin-2-yl)azepan-2-yl)methyl)butanamide (5ka)

Chiral SFC Analysis: CHIRALPAK IG (CO_2: MeOH, 85:15, 2.5 mL min^{-1}, 40 °C, 245 nm) indicated 73% ee, tR = 6.9 (minor), 10.6 (major) minutes.

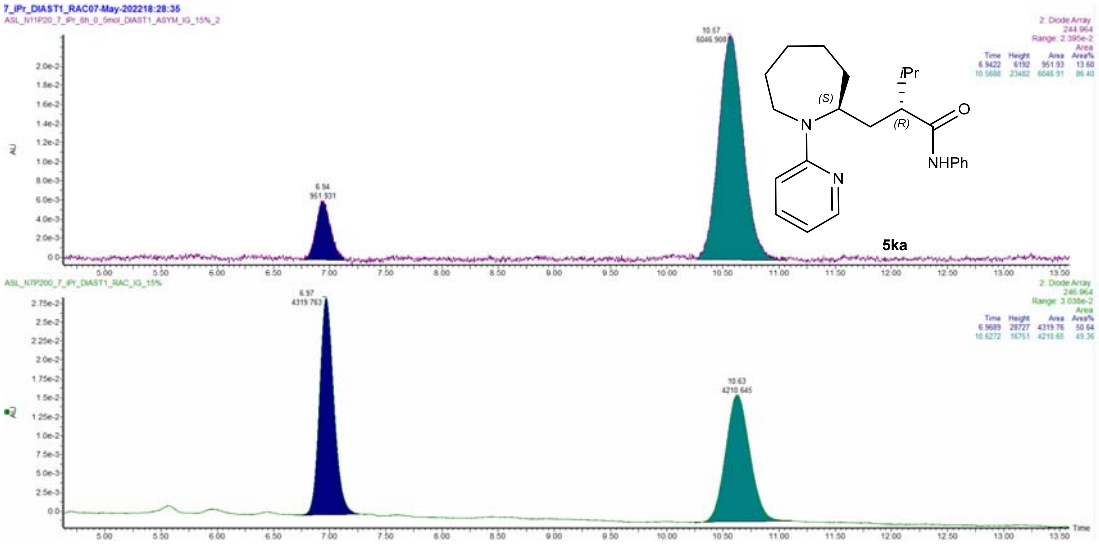

(*S*)-3-Methyl-*N*-phenyl-2-(((*S*)-1-(pyridin-2-yl)azepan-2-yl)methyl)butanamide (5kb)

Chiral SFC Analysis: CHIRALPAK IG (CO_2: MeOH, 80:20, 2.5 mL min^{-1}, 40 °C, 243 nm) indicated 94% ee, tR = 3.9 (major), 4.5 (minor) minutes.

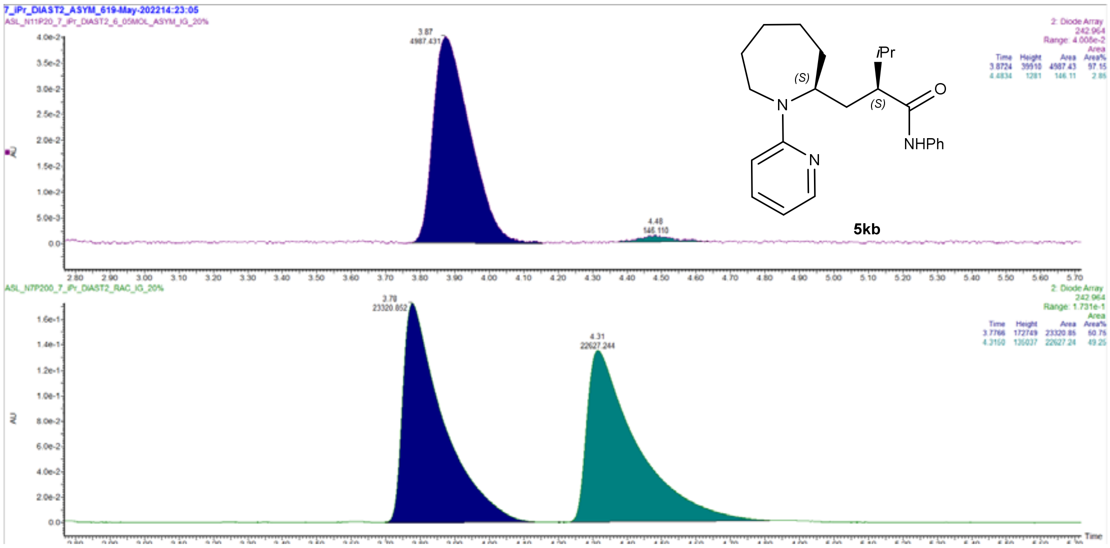

Methyl (R)-4-oxo-4-(phenylamino)-3-(((S)-1-(pyridin-2-yl)azepan-2-yl)methyl)butanoate (5la)

Chiral SFC Analysis: CHIRALPAK IG (CO_2: MeOH, 75:25, 2.5 mL min^{-1}, 40 °C, 245 nm) indicated 75% ee, tR = 7.3 (minor), 9.16 (major) minutes.

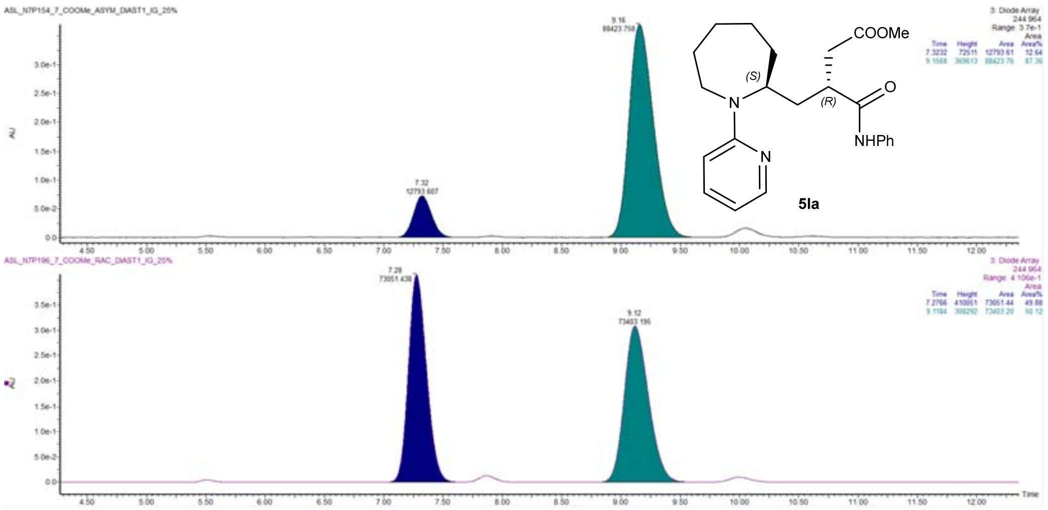

Methyl (S)-4-oxo-4-(phenylamino)-3-(((S)-1-(pyridin-2-yl)azepan-2-yl)methyl)butanoate (5lb)

Chiral SFC Analysis: CHIRALPAK IG (CO_2: MeOH, 70:30, 2.5 mL min^{-1}, 40 °C, 244 nm) indicated 93% ee, tR = 4.3 (minor), 11.7 (major) minutes.

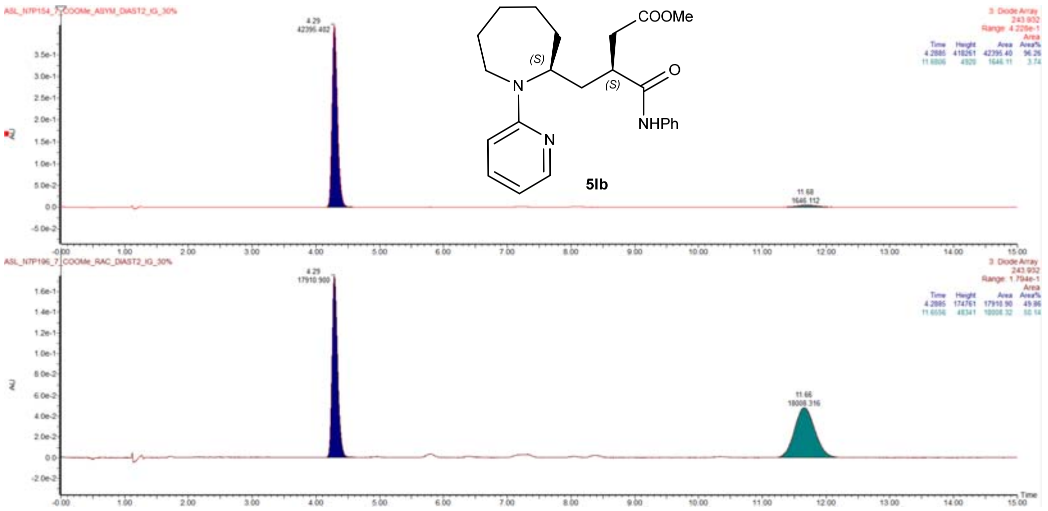

(*S*)-*N*-Phenyl-3-(1-(pyridin-2-yl)azepan-2-yl)propenamide (5m)

Chiral SFC Analysis CHIRAL ART SC (CO_2: MeOH, 80:20, 2.5 mL min^{-1}, 40 °C, 242 nm) indicated 94% ee, tR = 7.31 (major), 8.0 (minor) minutes.

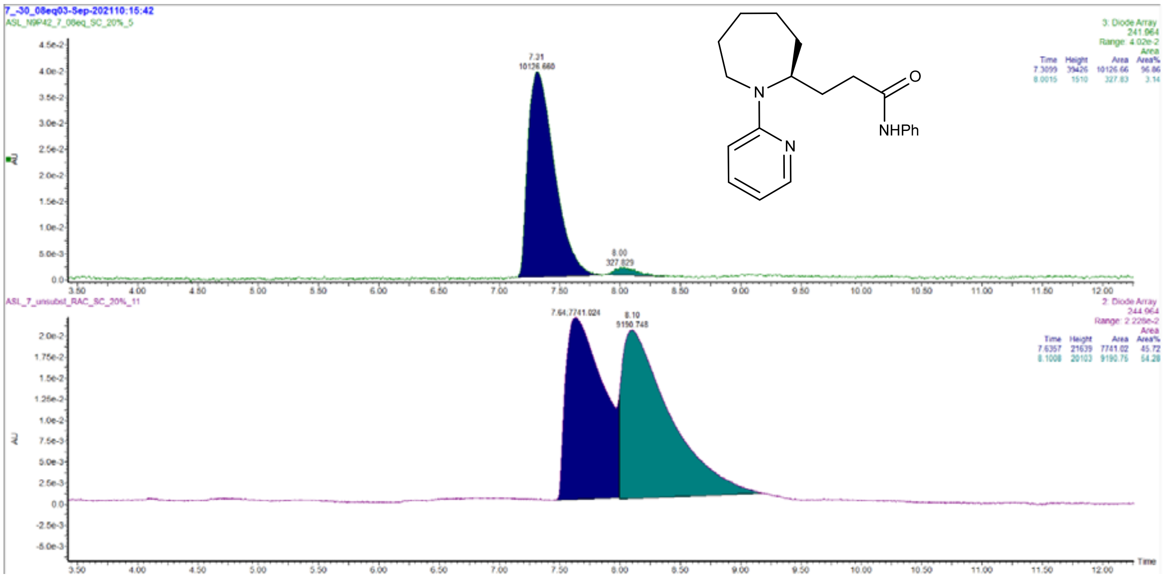

(2*S*,4*S*)-2-Benzyl-4-(ethyl(pyridin-2-yl)amino)-*N*-phenylpentanamide (5pa)

Chiral HPLC Analysis CHIRALPAK IA (*n*-Hexane:iPrOH, 95:5, 1.0 mL min-1, 30 °C, 250 nm) indicated 86% ee, tR = 12.4 (major), 22.5 (minor) minutes

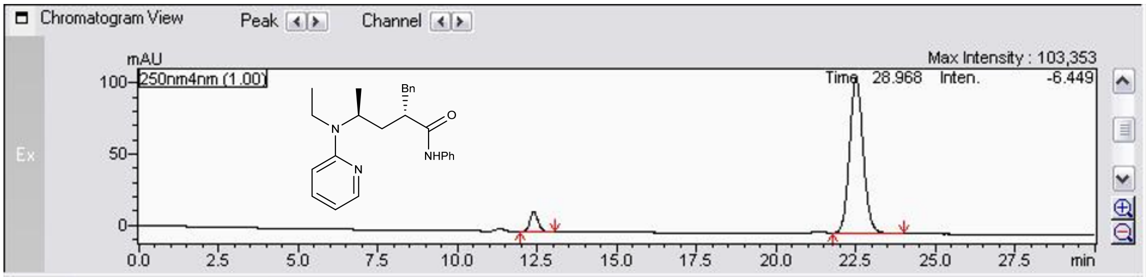

Results View - Peak Table

Peak Table | Compound | Group | Calibration Curve

Peak#	Ret. Time	Area	Height	Area%
1	12.392	240459	14102	6.906
2	22.509	3241396	108950	93.094
Total		3481854	123052	100.000

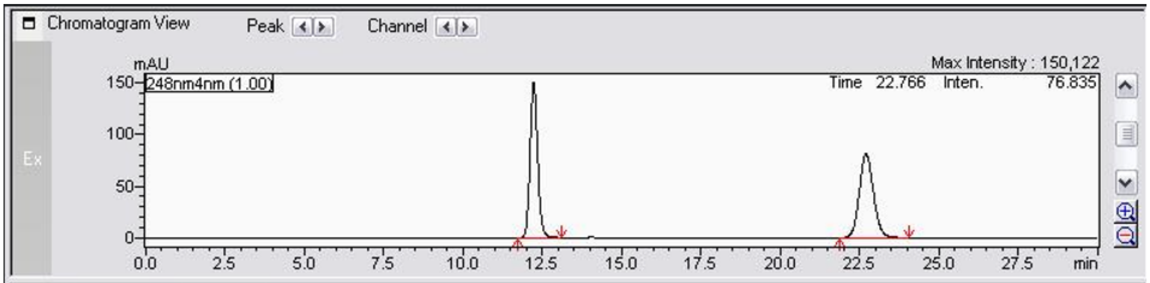

Results View - Peak Table

Peak Table | Compound | Group | Calibration Curve

Peak#	Ret. Time	Area	Height	Area%
1	12.220	2524492	150063	49.957
2	22.696	2528850	81211	50.043
Total		5053342	231274	100.000

(2*R*,4*S*)-2-Benzyl-4-(ethyl(pyridin-2-yl)amino)-*N*-phenylpentanamide (5pb).

Chiral HPLC Analysis: CHIRALPAK AD-H (*n*-Hexane:iPrOH, 95:5, 1.0 mL min-1, 30 °C, 242 nm) indicated 89% ee, tR = 10.6 (minor), 13.3 (major) minutes.

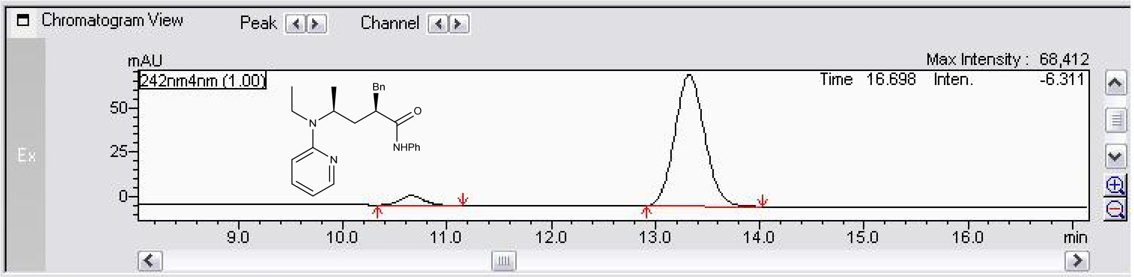

Results View - Peak Table

Peak Table | Compound | Group | Calibration Curve

Peak#	Ret. Time	Area	Height	Area%
1	10.656	84453	5340	5.434
2	13.322	1469617	73812	94.566
Total		1554070	79152	100.000

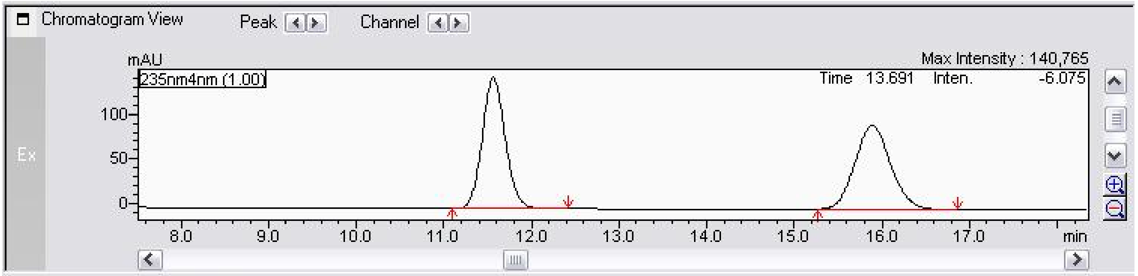

Results View - Peak Table

Peak Table | Compound | Group | Calibration Curve

Peak#	Ret. Time	Area	Height	Area%
1	11.562	2669069	146257	50.154
2	15.888	2652625	93506	49.846
Total		5321694	239763	100.000

2-Ethyl-4-(ethyl(pyridin-2-yl)amino)-*N*-phenylpentanamide (5qa)

Chiral SFC Analysis CHIRALPAK IG (CO_2: MeOH, 90:10, 2.5 mL min^{-1}, 40 °C, 241 nm) indicated 83% ee, tR = 6.5 (minor), 12.3 (major) minutes.

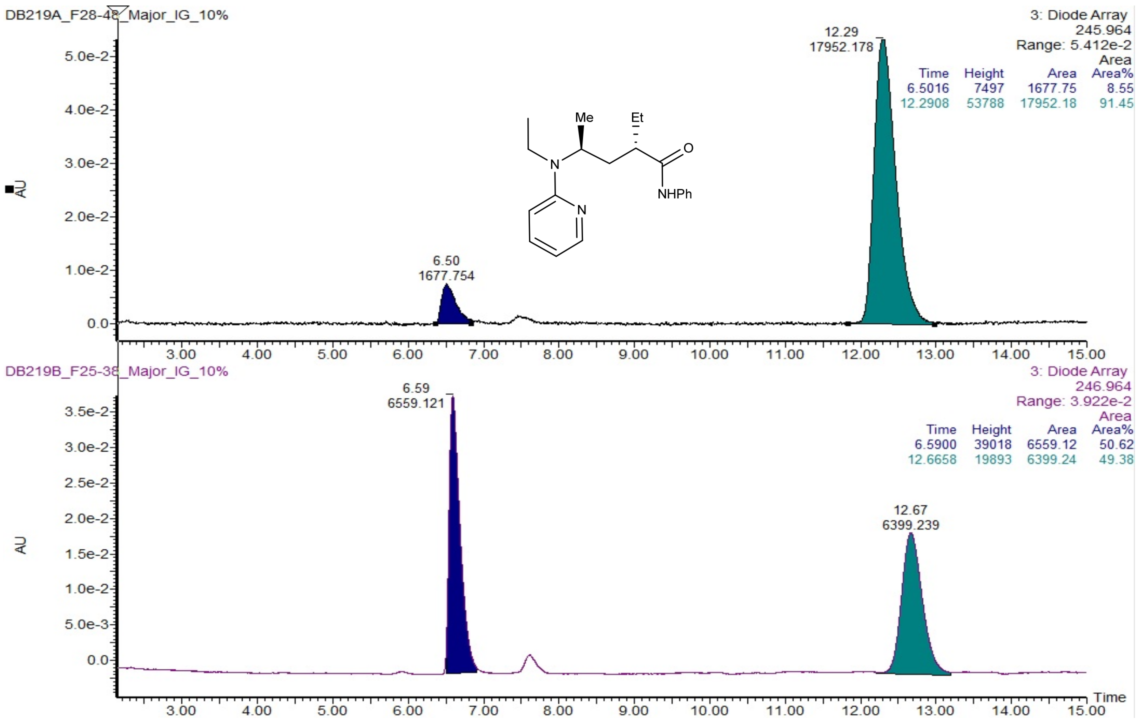

2-Ethyl-4-(ethyl(pyridin-2-yl)amino)-*N*-phenylpentanamide (5qb)

Chiral HPLC Analysis CHIRALCEL OD (*n*-Hexane: iPrOH, 90:10, 1.0 mL min^{-1}, 40 °C, 204 nm) indicated 89% ee, tR = 5.4 (major), 6.0 (minor) minutes.

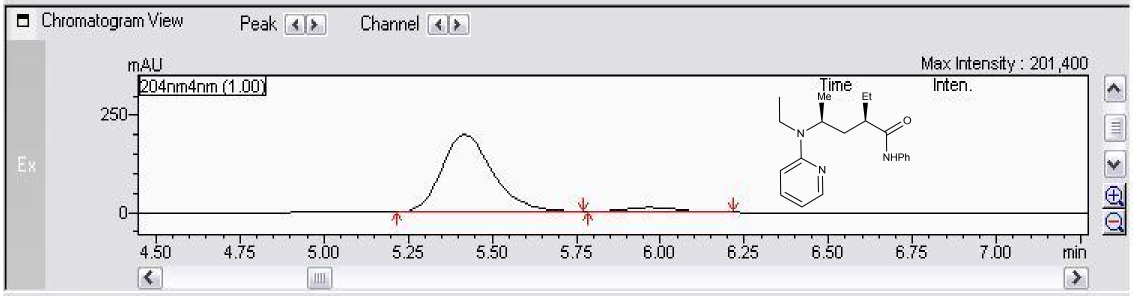

Results View - Peak Table

Peak Table | Compound | Group | Calibration Curve

Peak#	Ret. Time	Area	Height	Area%
1	5.419	2102511	199542	94.559
2	5.969	120983	11501	5.441
Total		2223495	211043	100.000

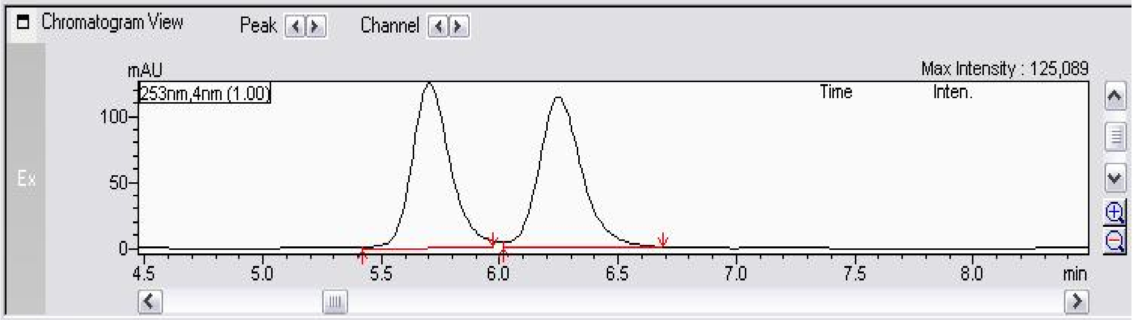

Results View - Peak Table

Peak Table | Compound | Group | Calibration Curve

Peak#	Ret. Time	Area	Height	Area%
1	5.702	1405772	124813	49.635
2	6.248	1426439	113899	50.365
Total		2832212	238712	100.000

(2*S*,4*S*)-2-ethyl-*N*-phenyl-4-(phenyl(pyridin-2-yl)amino)pentanamide (5ra)

Chiral SFC Analysis: CHIRAL ART SJ (CO_2: MeOH, 93:7, 2.5 mL min^{-1}, 40 °C, 240 nm) indicated 90% ee, tR = 5.0 (major), 6.0 (minor) minutes.

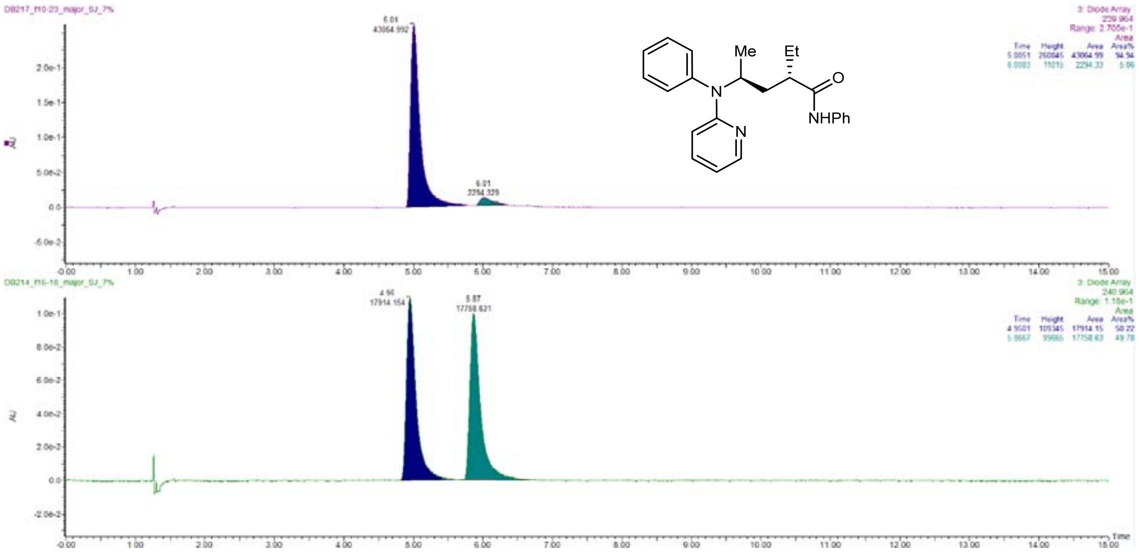

(2R,4S)-2-ethyl-*N*-phenyl-4-(phenyl(pyridin-2-yl)amino)pentanamide (5rb)

Chiral SFC Analysis: CHIRAL ART SJ (CO_2: MeOH, 93:7, 2.5 mL min^{-1}, 40 °C, 239 nm) indicated 85% ee, tR = 7.1 (minor), 8.1 (major) minutes.

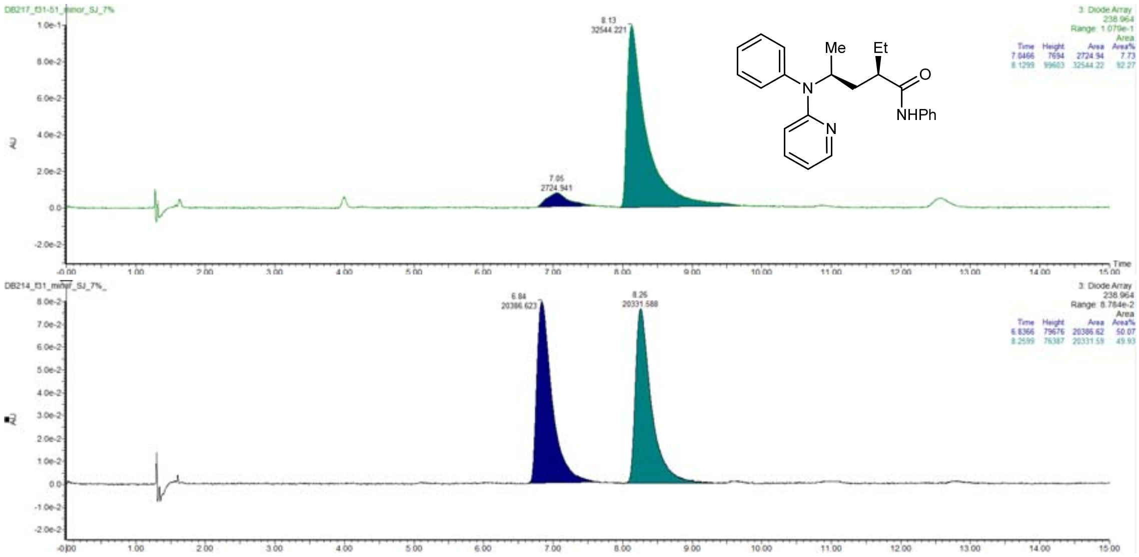

4-(Benzyl(pyridin-2-yl)amino)-2-ethyl-*N*-phenylpentanamide (5sa) diast 1

Chiral SFC Analysis CHIRALPAK IG (CO_2: MeOH, 90:10, 2.5 mL min^{-1}, 40 °C, 242 nm) indicated 78% ee, tR = 10.6 (minor), 18.6 (major) minutes.

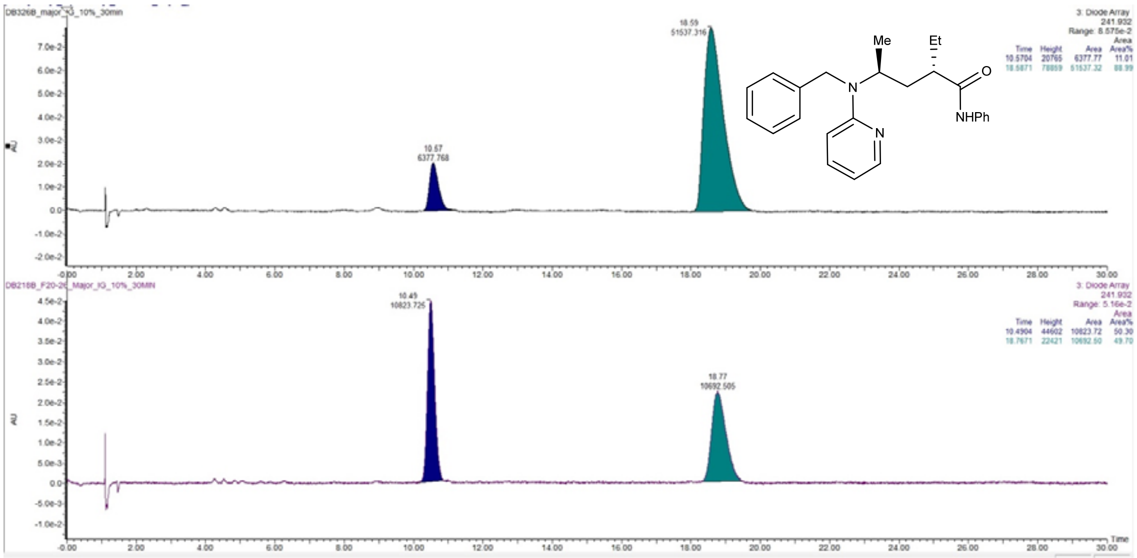

4-(Benzyl(pyridin-2-yl)amino)-2-ethyl-*N*-phenylpentanamide (5sb) diast 2

Chiral SFC Analysis CHIRALPAK IG (CO_2: MeOH, 90:10, 2.5 mL min^{-1}, 40 °C, 241 nm) indicated 91% ee, tR = 9.9 (minor), 10.8 (major) minutes.

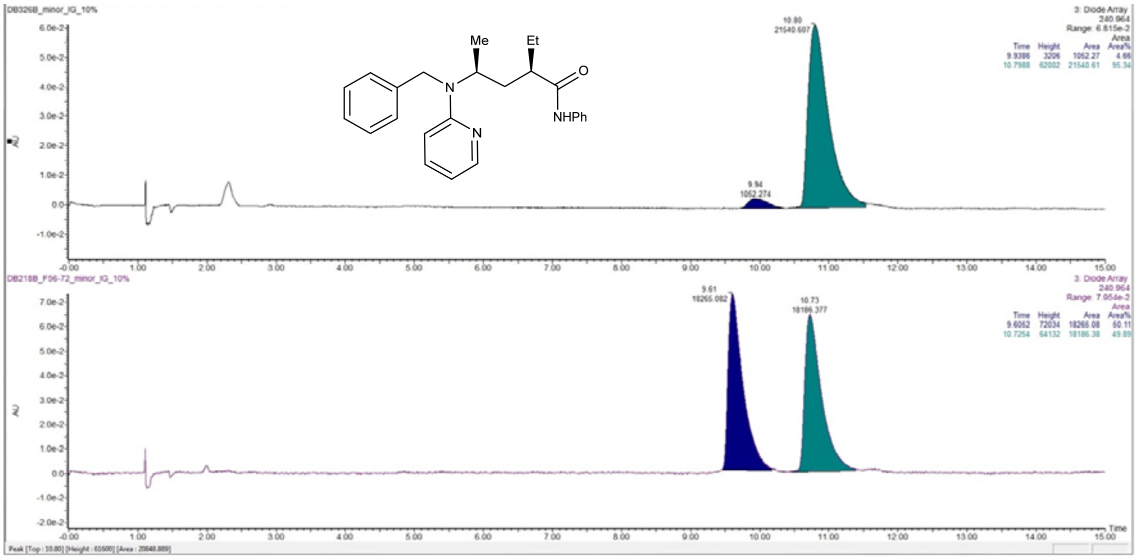

2-Benzyl-4-(methyl(pyridin-2-yl)amino)-*N*-phenylbutanamide (5t)

Chiral SFC Analysis: CHIRAL ART SJ (CO_2: MeOH, 80:20, 2.5 mL min^{-1}, 40 °C, 244 nm) indicated 4% ee, tR = 4.0 (minor), 4.8 (minor) minutes.

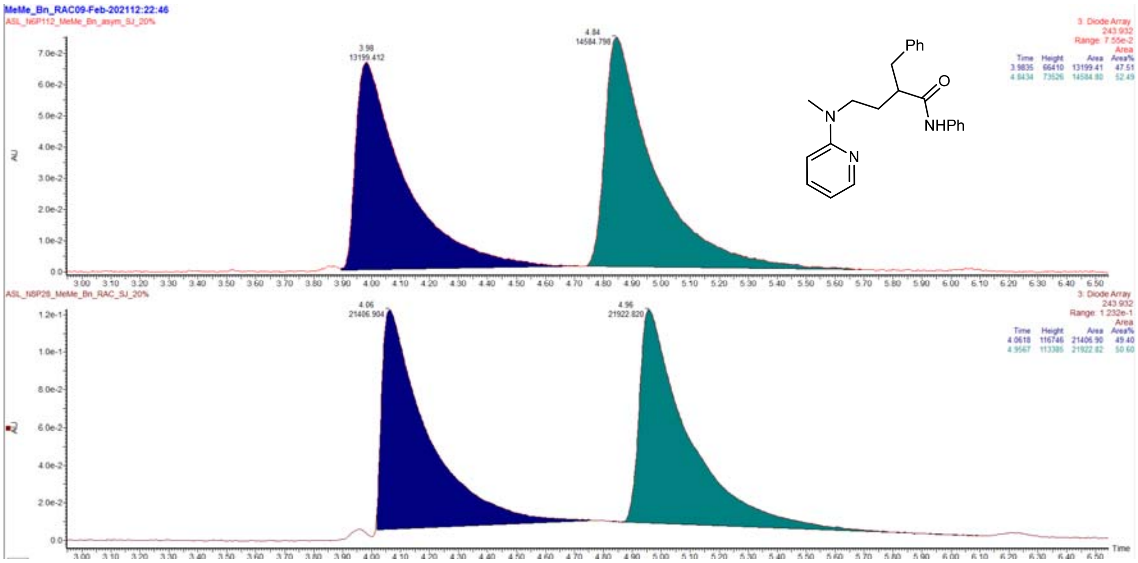

(*R*)-3-Methyl-4-(methyl(pyridin-2-yl)amino)-*N*-phenylbutanamide (6a)

Chiral SFC Analysis CHIRALPAK IG (CO_2: MeOH, 80:20, 2.5 mL min^{-1}, 40 °C, 244 nm) indicated 90% ee, tR = 6.5 (minor), 7.6 (major) minutes.

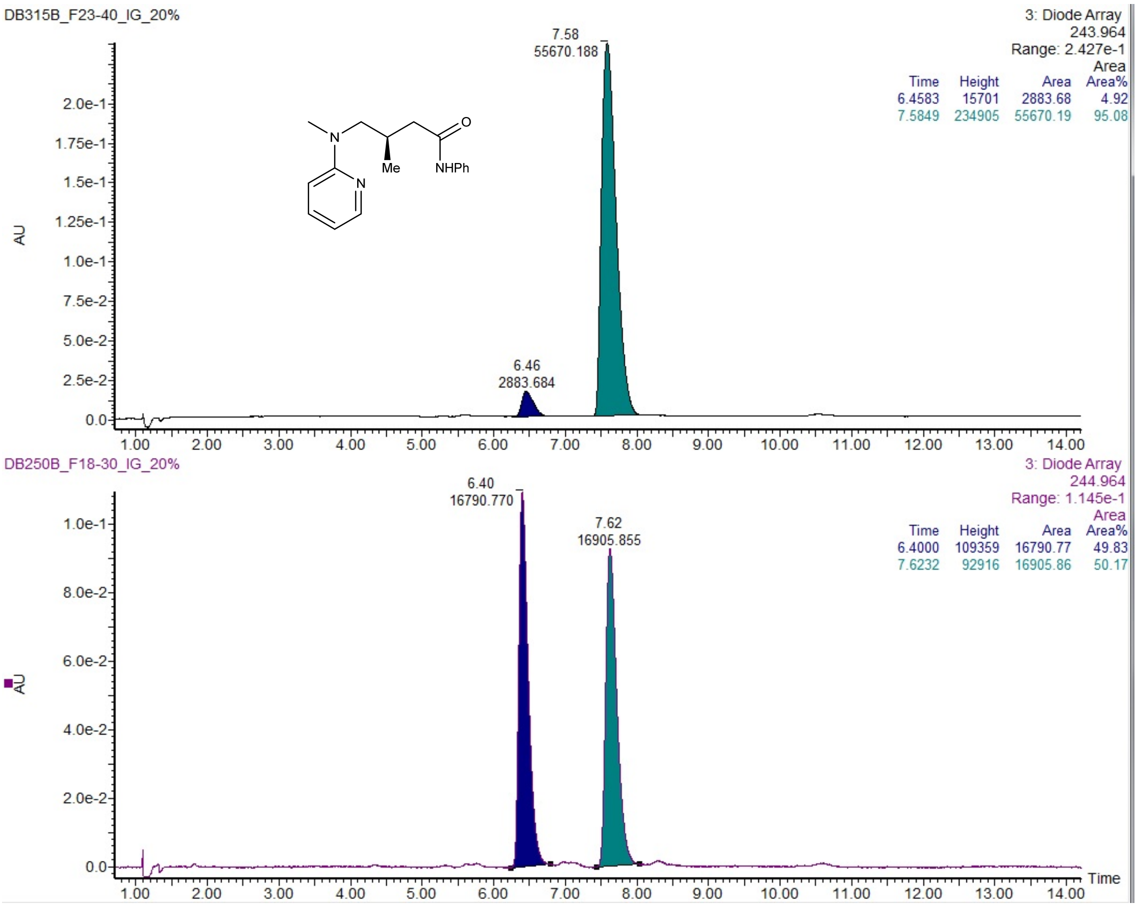

(*S*)-4-methyl-*N*-phenyl-3-((phenyl(pyridin-2-yl)amino)methyl)pentanamide (6c)

Chiral SFC Analysis: CHIRALPAK IG (CO_2: MeOH, 80:20, 2.5 mL min^{-1}, 40 °C, 238 nm) indicated 95% ee, tR = 4.7 (minor), 5.2 (major) minutes.

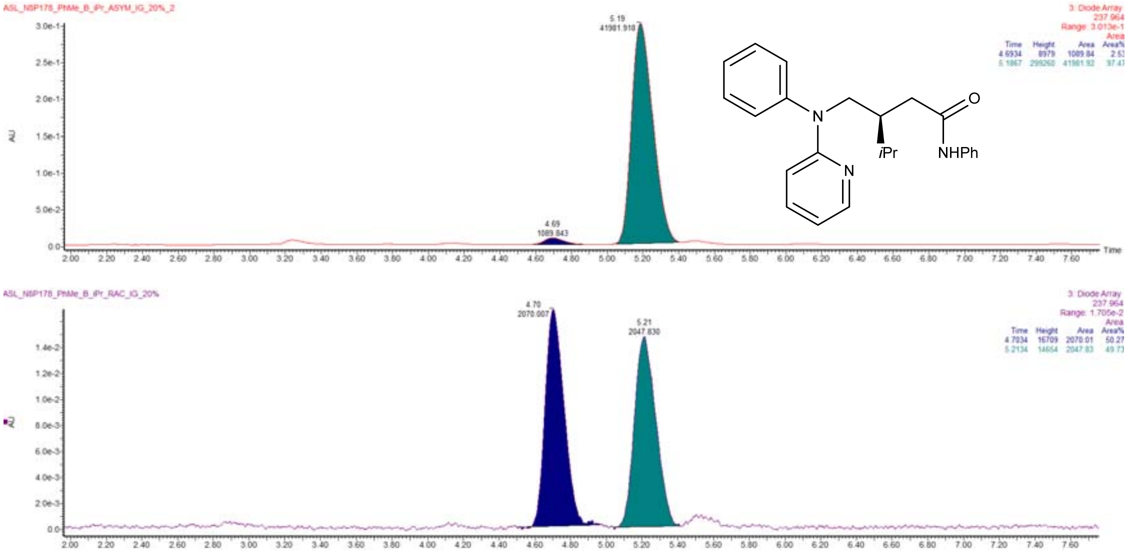

(*R*)-3-Methyl-*N*-phenyl-4-(pyridin-2-yl(4-(trifluoromethyl)phenyl)amino)butanamide (6d)

Chiral SFC Analysis CHIRALPAK IG (CO_2: MeOH, 80:20, 2.5 mL min^{-1}, 40 °C, 244 nm) indicated 97% ee, tR = 3.3 (minor), 4.5 (major) minutes.

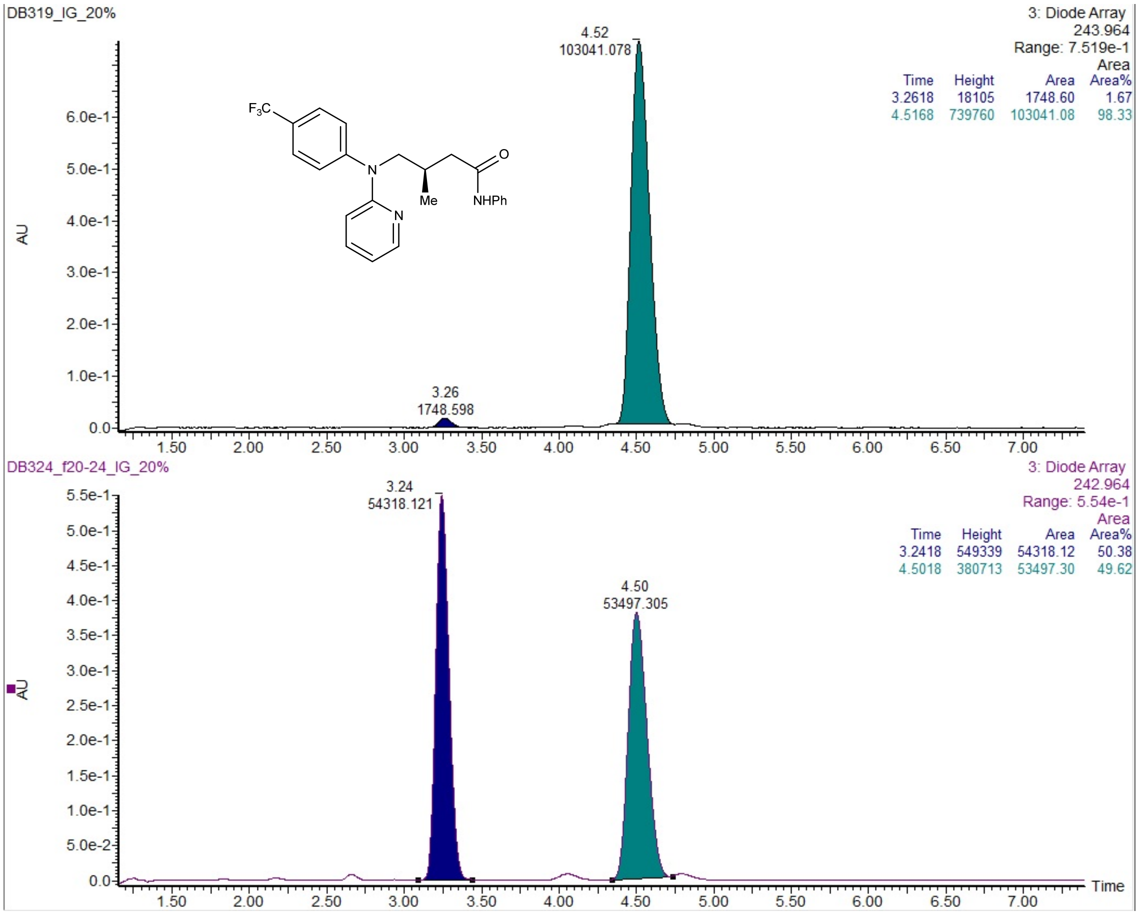

(R)-3-Methyl-4-(phenyl(pyridin-2-yl)amino)-*N*-(4-(trifluoromethyl)phenyl)butanamide (6e)

Chiral SFC Analysis: CHIRALPAK IG (CO_2: MeOH, 80:20, 2.5 mL min^{-1}, 40 °C, 244 nm) indicated 91% ee, tR = 2.8 (minor), 3.0 (major) minutes.

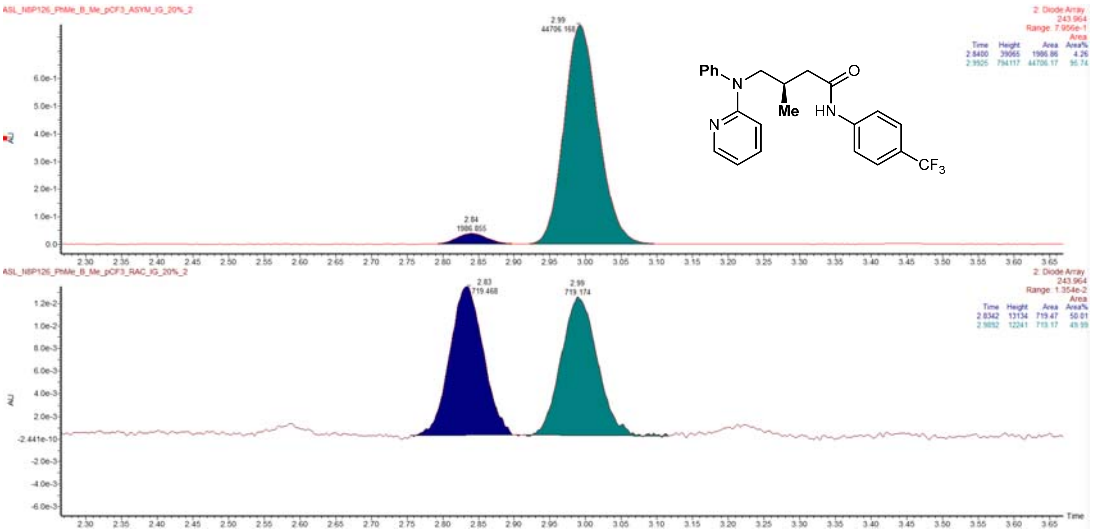

(*R*)-4-(benzyl(pyridin-2-yl)amino)-3-methyl-*N*-phenylbutanamide (6f)

Chiral SFC Analysis: CHIRAL ART SC (CO_2: MeOH, 85:15, 2.5 mL min^{-1}, 40 °C, 243 nm) indicated 95% ee, tR = 7.3 (major), 7.9 (minor) minutes.

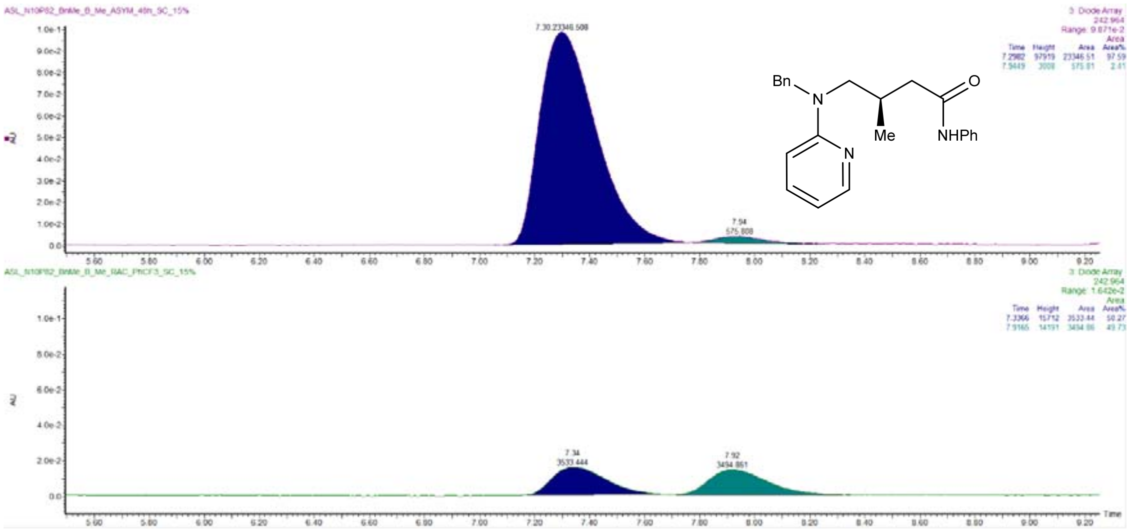

(*R*)-3-((Benzyl(pyridin-2-yl)amino)methyl)-*N*,5-diphenylpentanamide (6g)

Chiral SFC Analysis CHIRAL ART SJ (CO_2: MeOH, 80:10, 2.5 mL min^{-1}, 40 °C, 246 nm) indicated 97% ee, tR = 8.0 (minor), 12.1 (major) minutes.

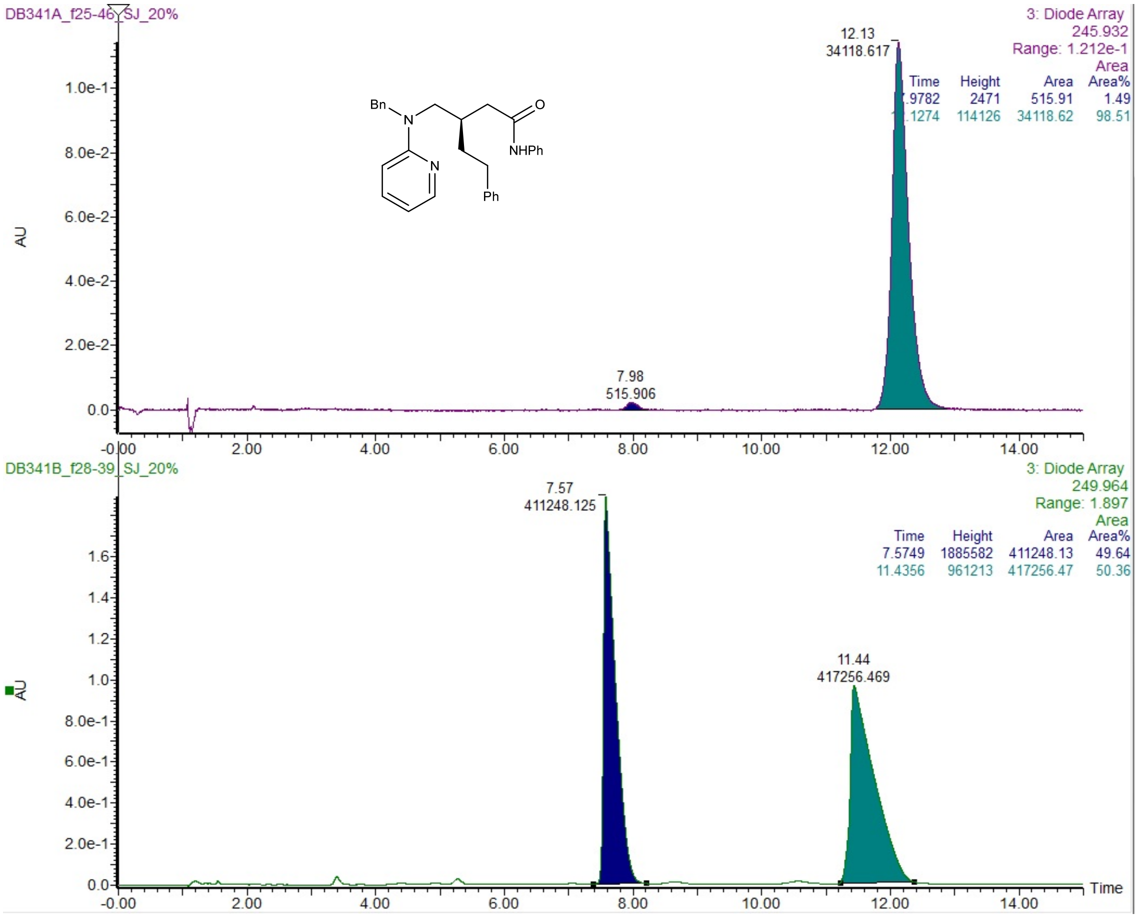

(*S*)-3-(((2-((*tert*-Butyldimethylsilyl)oxy)ethyl)(pyridin-2-yl)amino)methyl)-4-methyl-*N*-phenylpentanamide (6h)

Chiral HPLC Analysis: CHIRALPAK AD-H (*n*-Hexane: iPrOH, 97:3, 1.0 mL min^{-1}, 30 °C, 241 nm) indicated 94% ee, tR = 8.9 (minor), 10.5 (major) minutes.

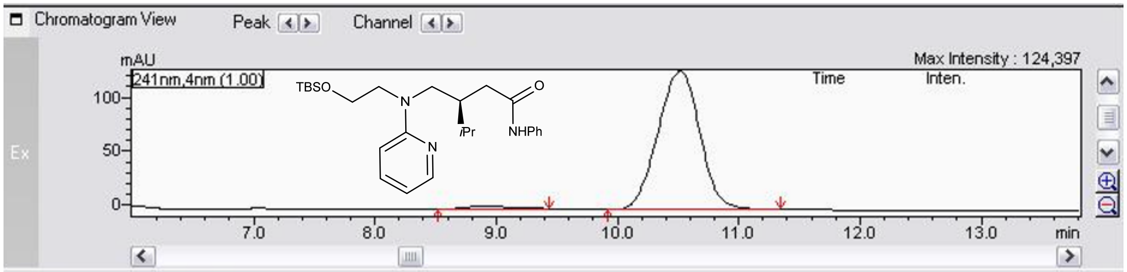

Results View - Peak Table

Peak Table | Compound | Group | Calibration Curve

Peak#	Ret. Time	Area	Height	Area%
1	8.924	87980	3135	2.751
2	10.519	3109590	128736	97.249
Total		3197569	131871	100.000

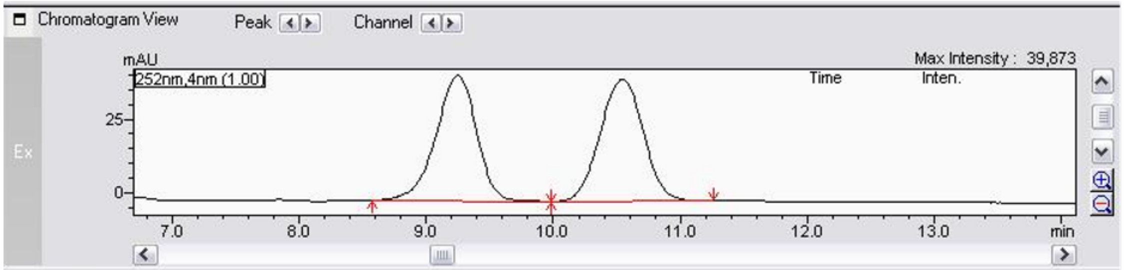

Results View - Peak Table

Peak Table | Compound | Group | Calibration Curve

Peak#	Ret. Time	Area	Height	Area%
1	9.249	940611	42499	49.052
2	10.545	976970	41372	50.948
Total		1917582	83870	100.000

2,3-Dimethyl-4-(methyl(pyridin-2-yl)amino)-*N*-phenylbutanamide (6ia) diast 1

Chiral SFC Analysis CHIRAL ART SC (CO_2: MeOH, 85:15, 2.5 mL min^{-1}, 40 °C, 242 nm) indicated 91% ee, tR = 5.3 (minor), 5.6 (major) minutes.

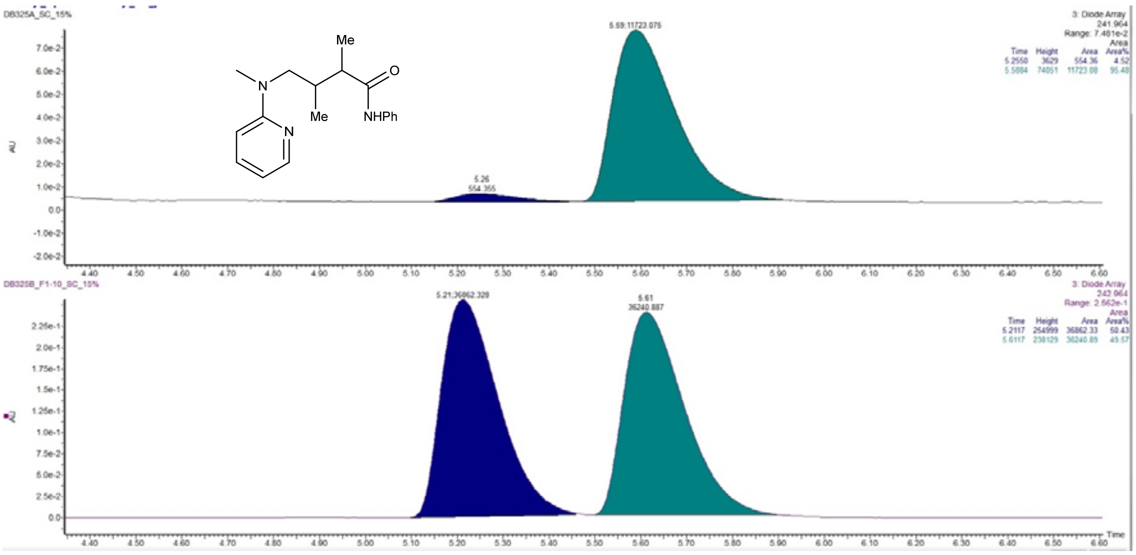

2,3-Dimethyl-4-(methyl(pyridin-2-yl)amino)-*N*-phenylbutanamide (6ib) diast 2

Chiral HPLC Analysis CHIRALPAK IC (*n*-Hexane: iPrOH, 95:5, 1.0 mL min^{-1}, 40 °C, 245 nm) indicated 97% ee, tR = 16.5 (major), 21.2 (minor) minutes.

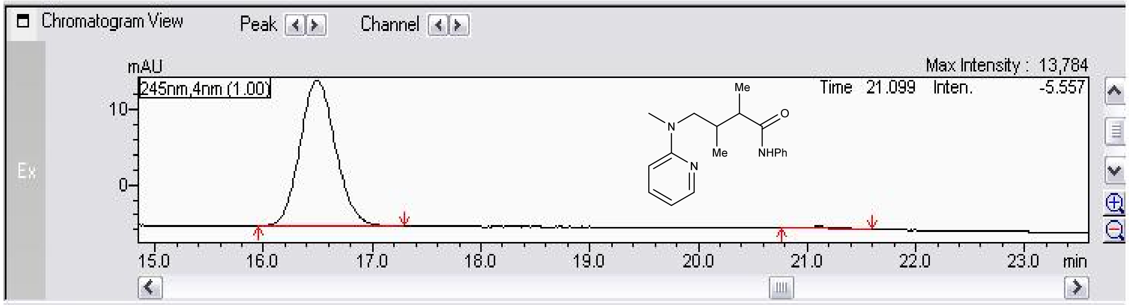

Results View - Peak Table

Peak Table | Compound | Group | Calibration Curve

Peak#	Ret. Time	Area	Height	Area%
1	16.495	438155	19251	98.386
2	21.088	7189	270	1.614
Total		445344	19521	100.000

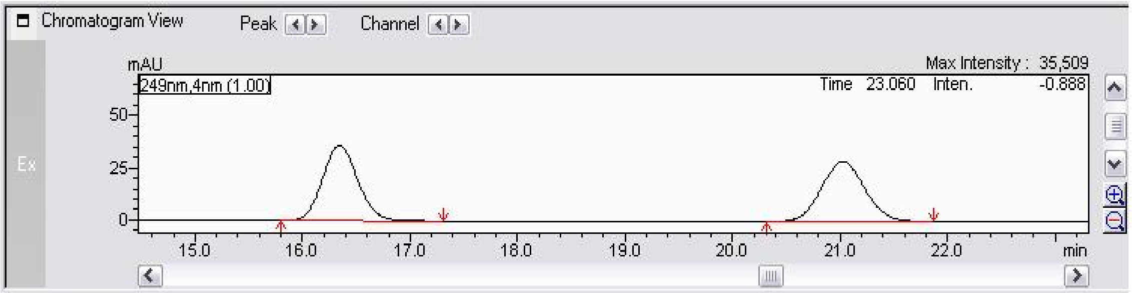

Results View - Peak Table

Peak Table | Compound | Group | Calibration Curve

Peak#	Ret. Time	Area	Height	Area%
1	16.344	813981	35920	50.051
2	21.021	812317	28772	49.949
Total		1626297	64692	100.000

2-((methyl(pyridin-2-yl)amino)methyl)-*N*-phenylcyclohexane-1-carboxamide (6ja) diast 1

Chiral SFC Analysis CHIRAL ART SC (CO_2: MeOH, 85:15, 2.5 mL min^{-1}, 40 °C, 243 nm) indicated 89% ee, tR = 6.4 (minor), 7.1 (major) minutes.

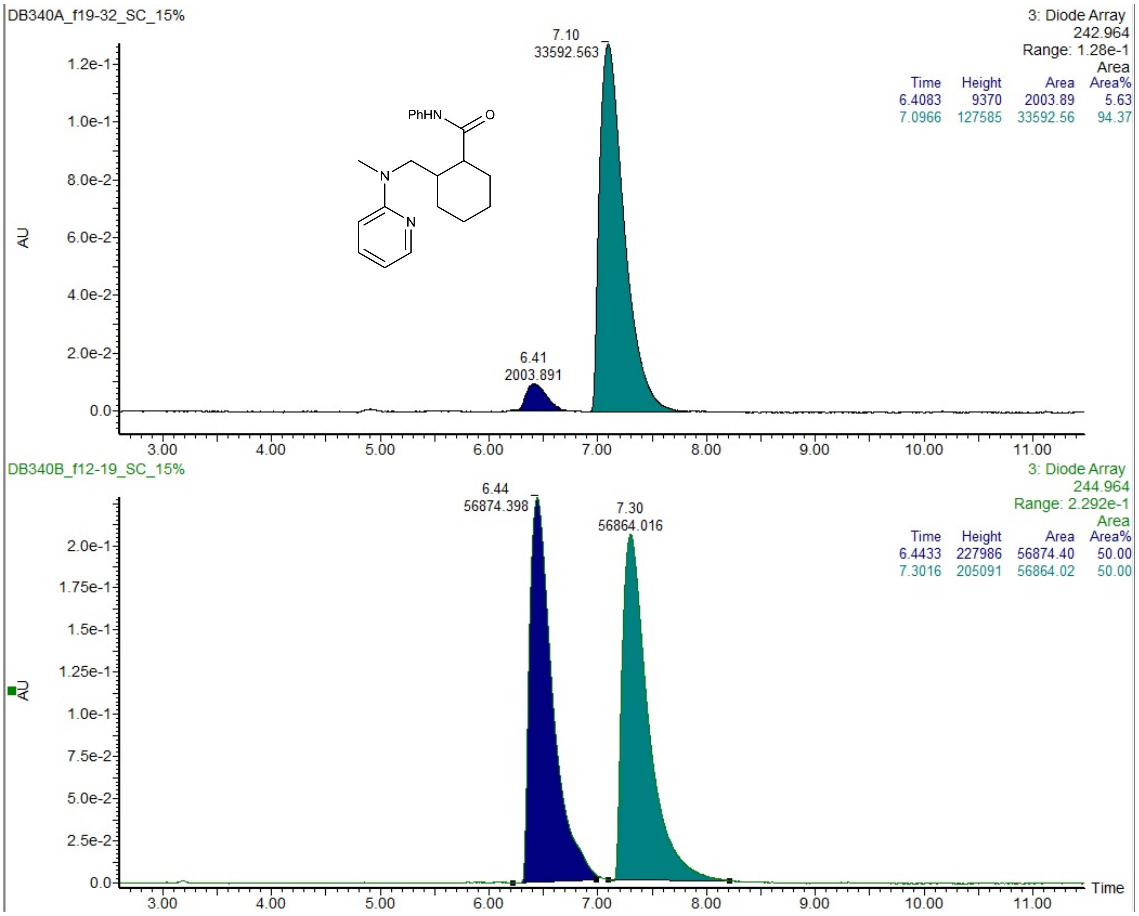

2-((Methyl(pyridin-2-yl)amino)methyl)-*N*-phenylcyclohexane-1-carboxamide (6jb) diast 2

Chiral HPLC Analysis CHIRALCEL OD (*n*-Hexane: iPrOH, 90:10, 1.0 mL min^{-1}, 40 °C, 248 nm) indicated 97% ee, tR = 13.6 (minor), 18.6 (major) minutes.

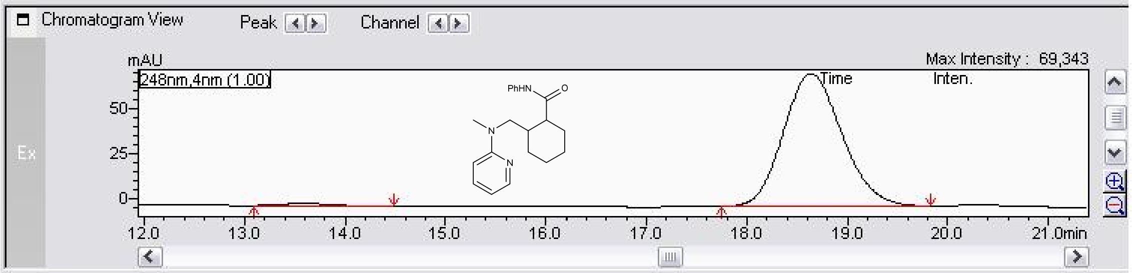

Results View - Peak Table

Peak Table | Compound | Group | Calibration Curve

Peak#	Ret. Time	Area	Height	Area%
1	13.609	42096	1364	1.394
2	18.632	2978156	73748	98.606
Total		3020252	75112	100.000

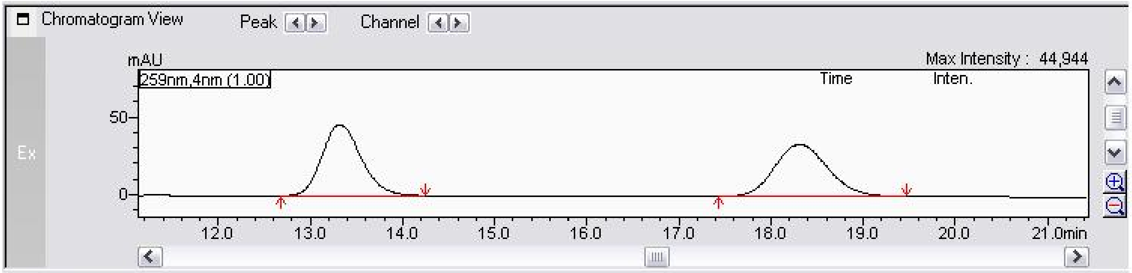

Results View - Peak Table

Peak Table | Compound | Group | Calibration Curve

Peak#	Ret. Time	Area	Height	Area%
1	13.320	1338701	45572	50.339
2	18.312	1320664	33071	49.661
Total		2659365	78643	100.000

4-Methyl-*N*-phenyl-3-(1-(pyridin-2-yl)azetidin-2-yl)pentanamide (6ka) diast 1

Chiral SFC Analysis CHIRALPAK IG (CO_2: MeOH, 85:15, 2.5 mL min^{-1}, 40 °C, 241 nm) indicated 99% ee, tR = 9.3 (minor), 11.2 (major) minutes.

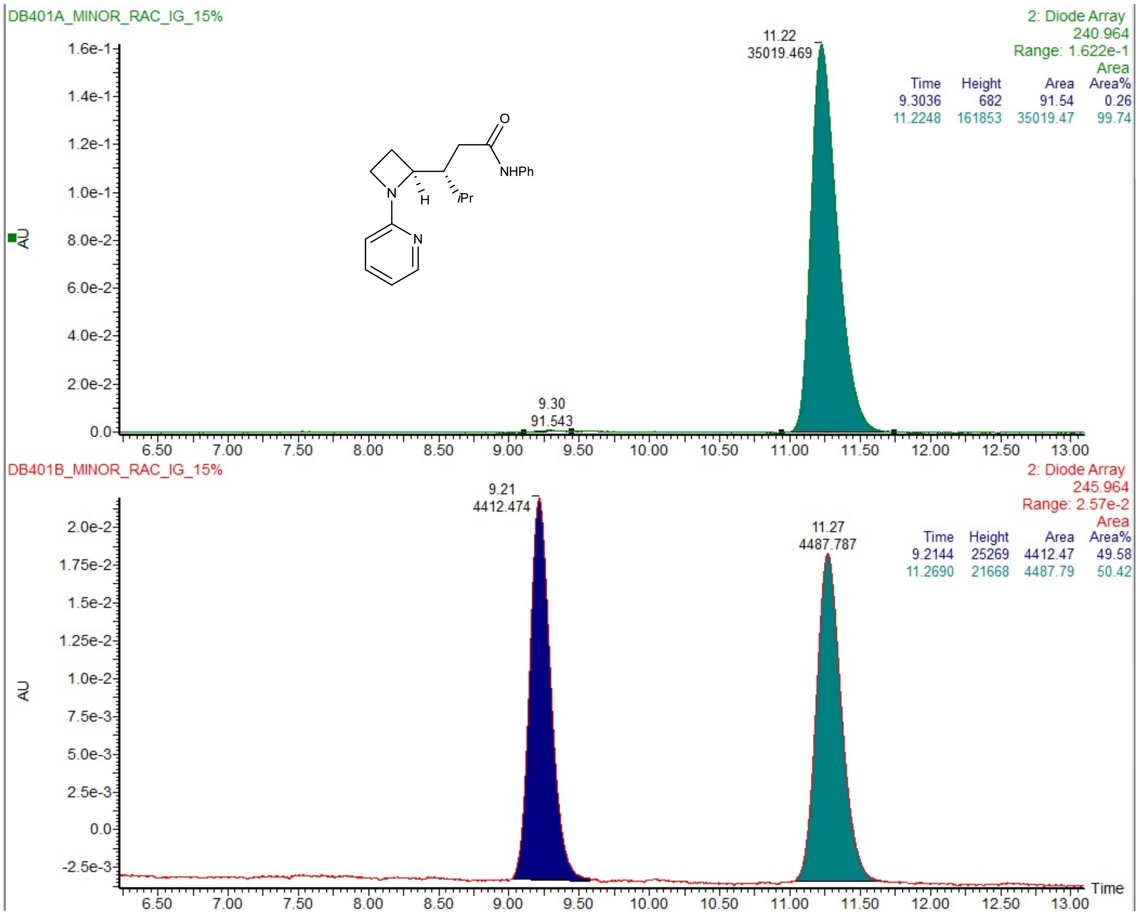

4-Methyl-*N*-phenyl-3-(1-(pyridin-2-yl)azetidin-2-yl)pentanamide (6kb) diast 2

Chiral SFC Analysis CHIRAL ART SB (CO_2: MeOH, 90:10, 2.5 mL min^{-1}, 40 °C, 246 nm) indicated 99% ee, tR = 5.6 (minor), 7.7 (major) minutes.

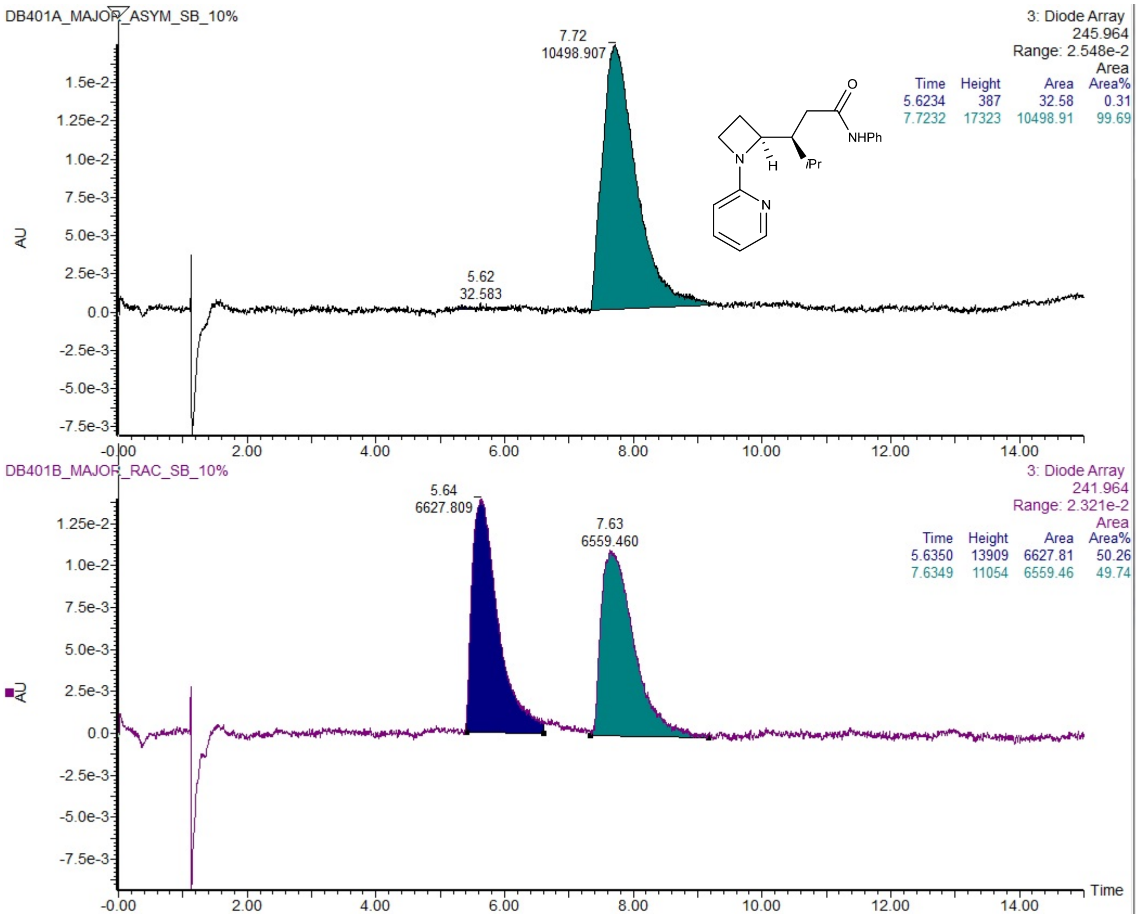

(*R*)-*N*-phenyl-3-((*S*)-1-(pyridin-2-yl)azepan-2-yl)butanamide (6l)

Chiral SFC Analysis: CHIRALPAK IG (CO_2: MeOH, 80:20, 2.5 mL min^{-1}, 40 °C, 244 nm) indicated 98% ee, tR = 7.8 (minor), 9.2 (major) minutes.

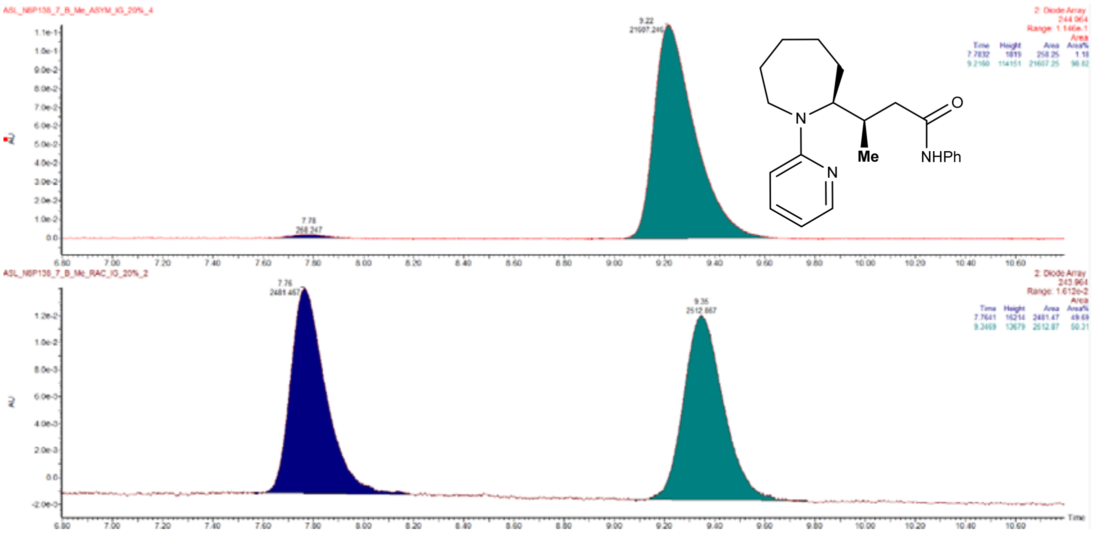

(2*S*,3*S*)-2-methyl-*N*-phenyl-3-((*S*)-1-(pyridin-2-yl)azetidin-2-yl)butanamide (6m)

Chiral SFC Analysis CHIRAL ART SC (CO_2: MeOH, 90:10, 2.5 mL min^{-1}, 40 °C, 242 nm) indicated 96% ee, tR = 7.2 (major), 9.1 (minor) minutes.

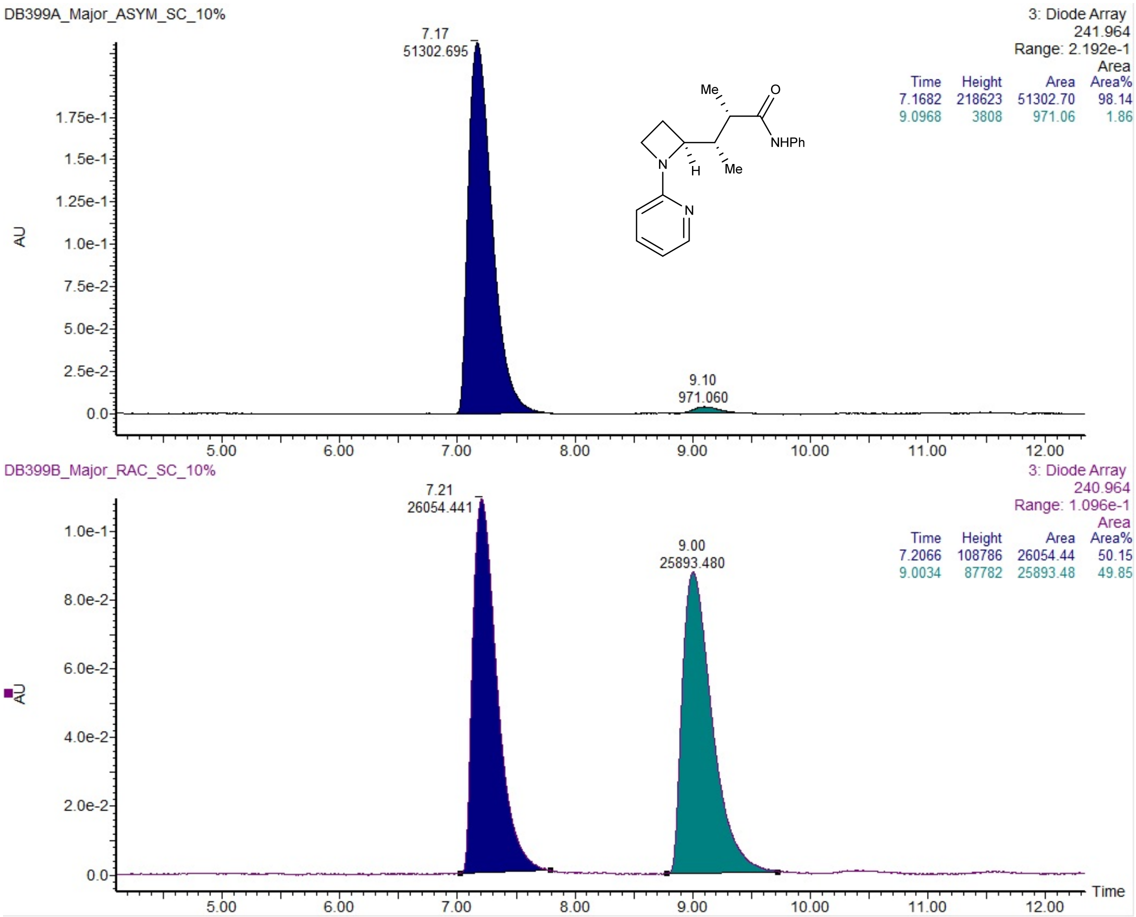

(*2S,3S*)-2-Methyl-*N*-phenyl-3-((*S*)-1-(pyridin-2-yl)pyrrolidin-2-yl)butanamide (6n)

Chiral SFC Analysis: CHIRAL ART SC (CO_2: EtOH, 90:10, 2.5 mL min^{-1}, 40 °C, 247 nm) indicated 99% ee, tR = 10.4 (major), 12.6 (minor) minutes.

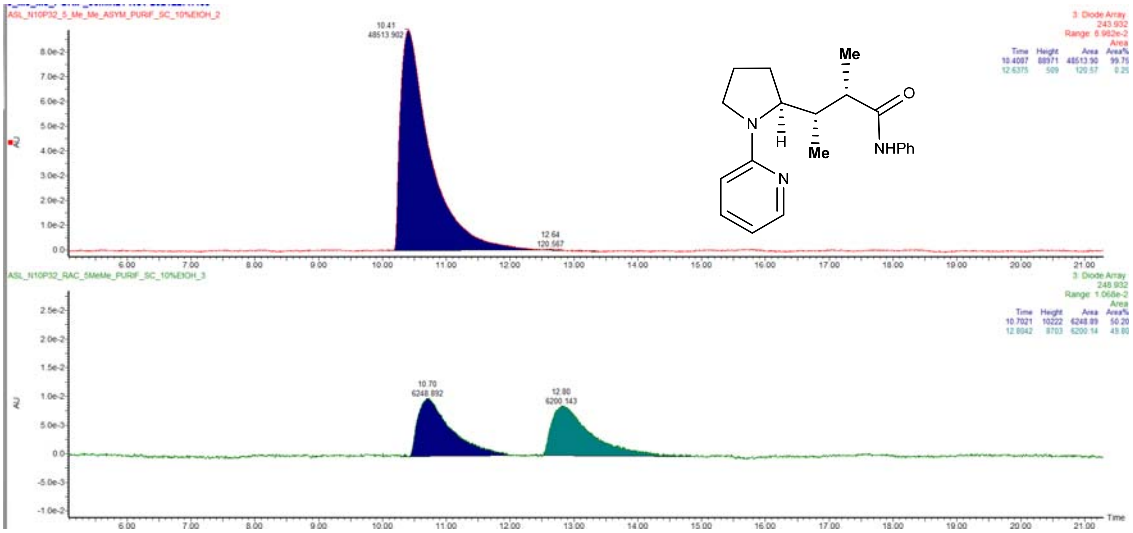

(2*S*,3*S*)-2-Methyl-*N*-phenyl-3-((*S*)-1-(pyridin-2-yl)azepan-2-yl)butanamide (6o)

Chiral SFC Analysis: CHIRAL ART SC (CO_2: EtOH, 90:10, 2.5 mL min^{-1}, 40 °C, 247 nm) indicated 99% ee, tR = 13.8 (major), 15.6 (minor) minutes.

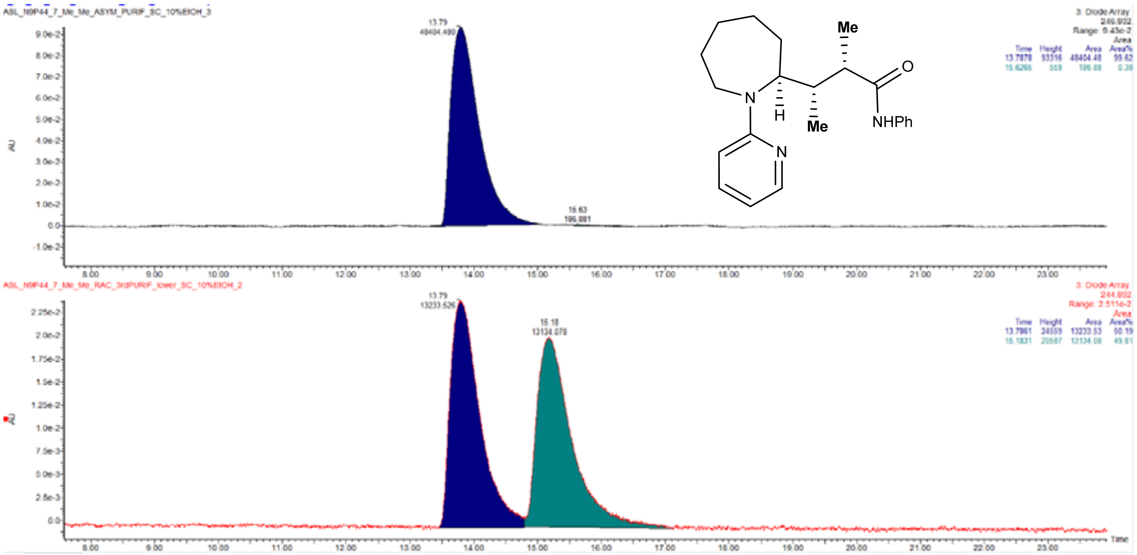

(*R*)-3-((Benzyl(pyridin-2-yl)amino)methyl)-5-methyl-*N*-phenylhexanamide (6p)

Chiral SFC Analysis: CHIRAL ART SC (CO_2: MeOH, 80:20, 2.5 mL min^{-1}, 40 °C, 243 nm) indicated 99% ee, tR = 4.9 (major), 5.3 (minor) minutes.

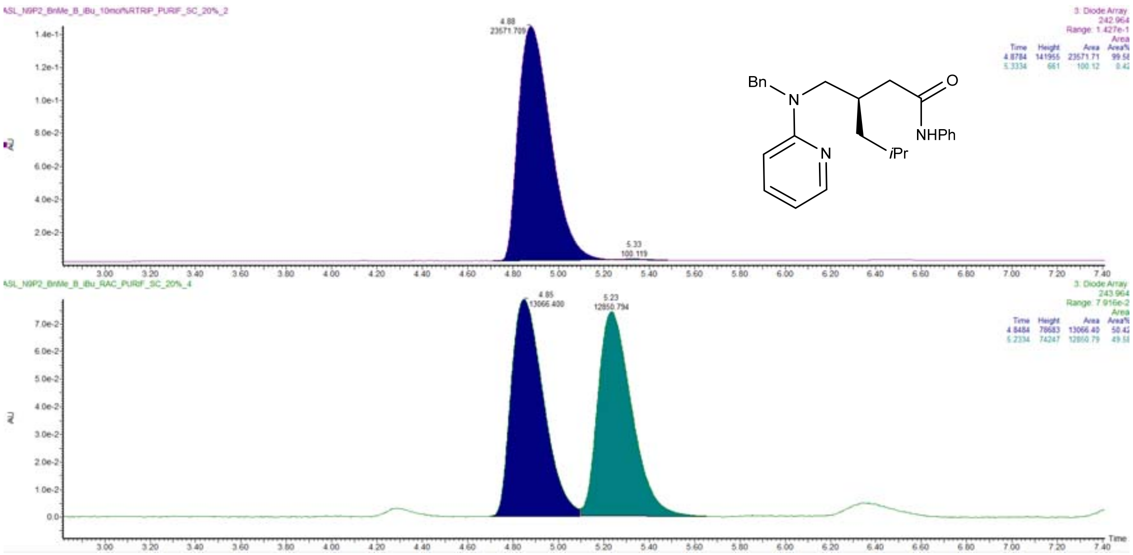

(R)-4-isobutylpyrrolidin-2-one (6pb)

Chiral SFC Analysis: CHIRALPAK IG (CO_2: MeOH, 90:10, 2.5 mL min^{-1}, 40 °C, 202 nm) indicated 99% ee, tR = 4.4 (minor), 4.7 (major) minutes.

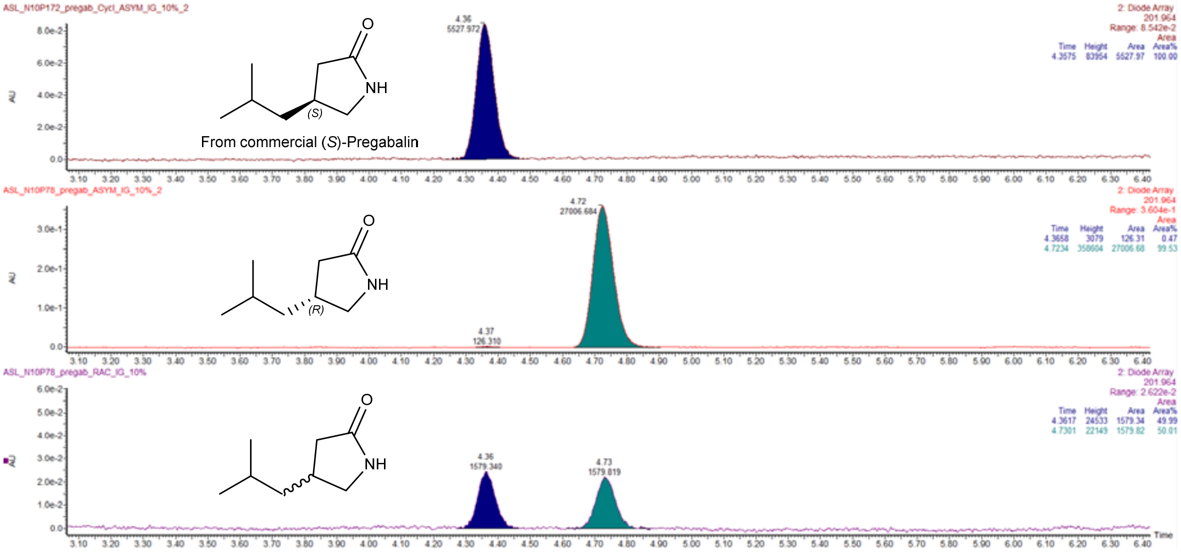

(*R*)-*N*-Phenyl-3-((*S*)-1-(pyridin-2-yl)pyrrolidin-2-yl)butanamide (6q)

Chiral SFC Analysis: CHIRALPAK IG (CO_2: MeOH, 80:20, 2.5 mL min^{-1}, 40 °C, 312 nm) indicated 90% ee, tR = 9.0 (minor), 10.5 (major) minutes.

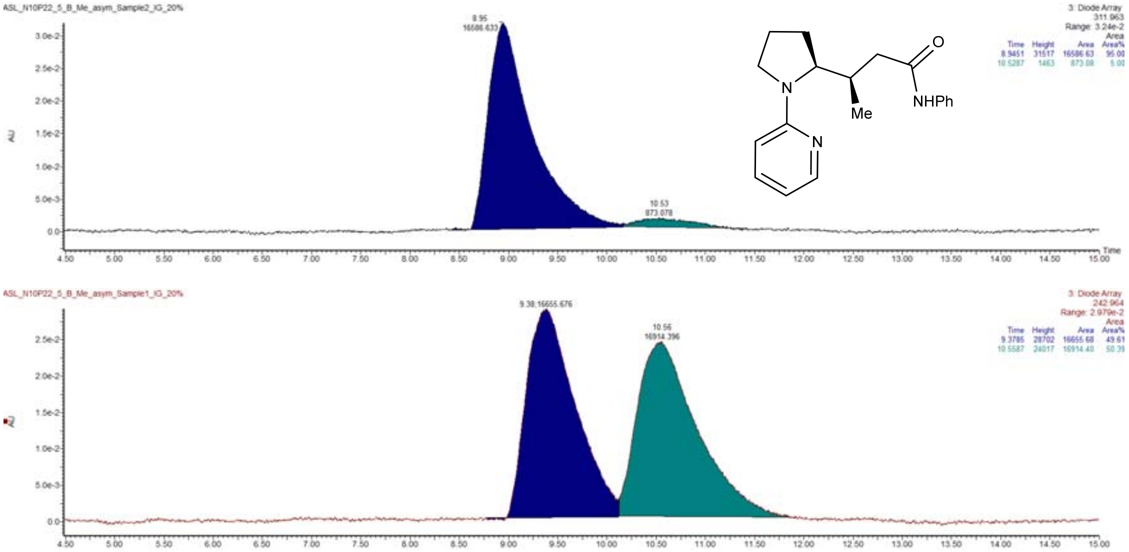

(1*R*,7a*S*)-1-methylhexahydro-3H-pyrrolizin-3-one (6qb)

Chiral GC Analysis: CP-chirasil-Dex CB (70 °C to 100 °C, 5 °C/min, 9.99 psi), indicated 90% ee, tR = 20.0 (minor), 20.3 (major) minutes.

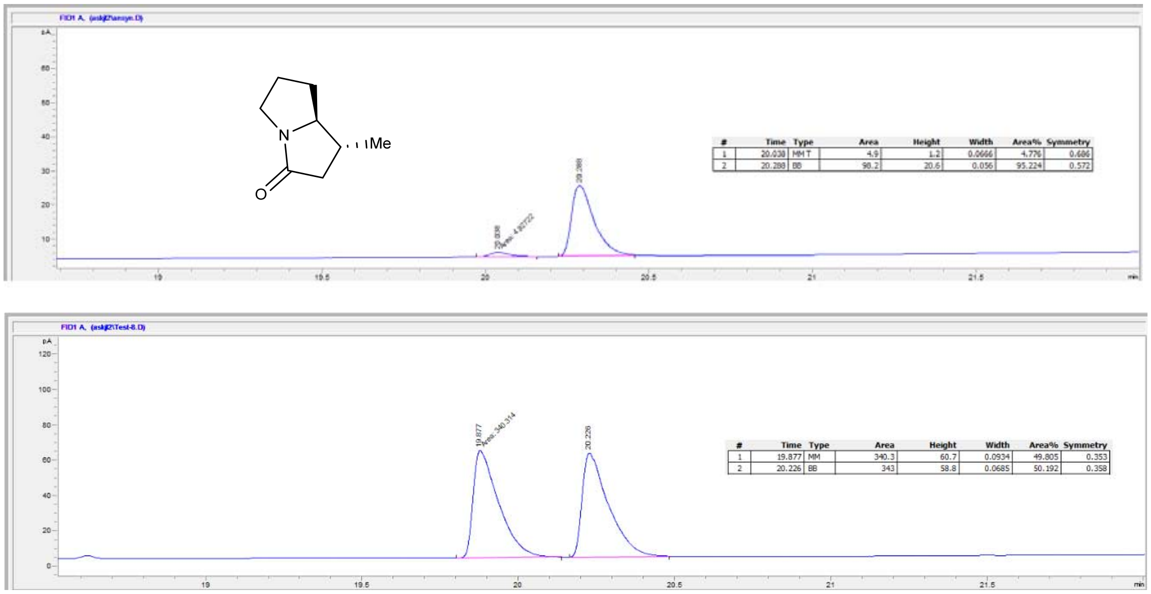

(*5S,6aS*)-5-Benzyl-*N*-phenyl-5,6,6a,7,8,9-hexahydropyrrolo[1,2-a][1,5]naphthyridine-5-carboxamide (7aa)

Chiral SFC Analysis: CHIRALPAK IG (CO_2: MeOH, 75:25, 2.5 mL min^{-1}, 40 °C, 259 nm) indicated racemic, tR = 5.5, 9.3 minutes.

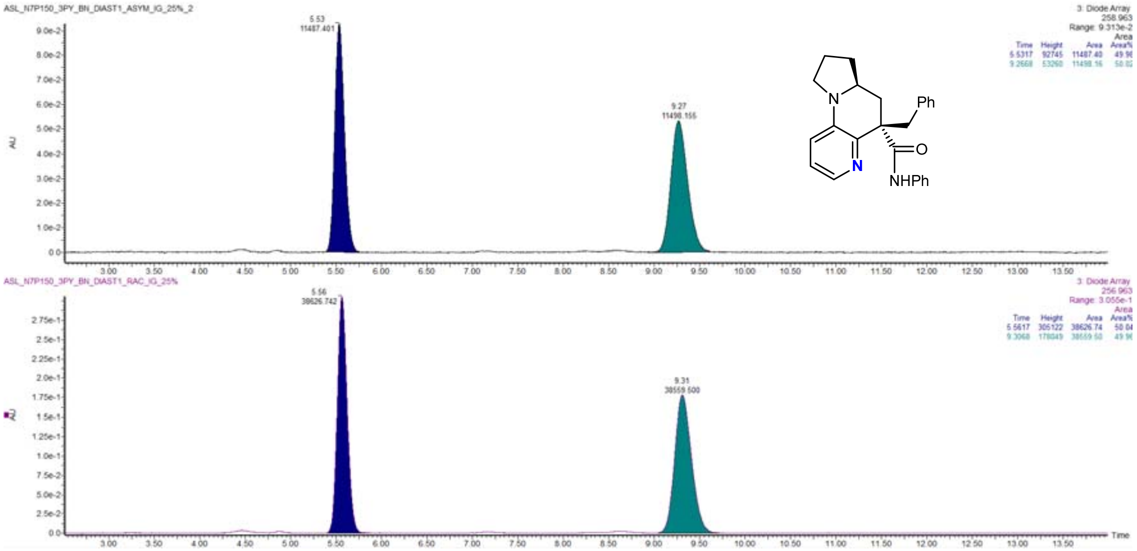

(*5R,6aS*)-5-Benzyl-*N*-phenyl-5,6,6a,7,8,9-hexahydropyrrolo[1,2-a][1,5]naphthyridine-5-carboxamide (7ab)

Chiral SFC Analysis: CHIRALPAK IG (CO_2: MeOH, 60:40, 2.5 mL min^{-1}, 40 °C, 259 nm) indicated 25% ee, tR = 11.6 (minor), 13.2 (major) minutes.

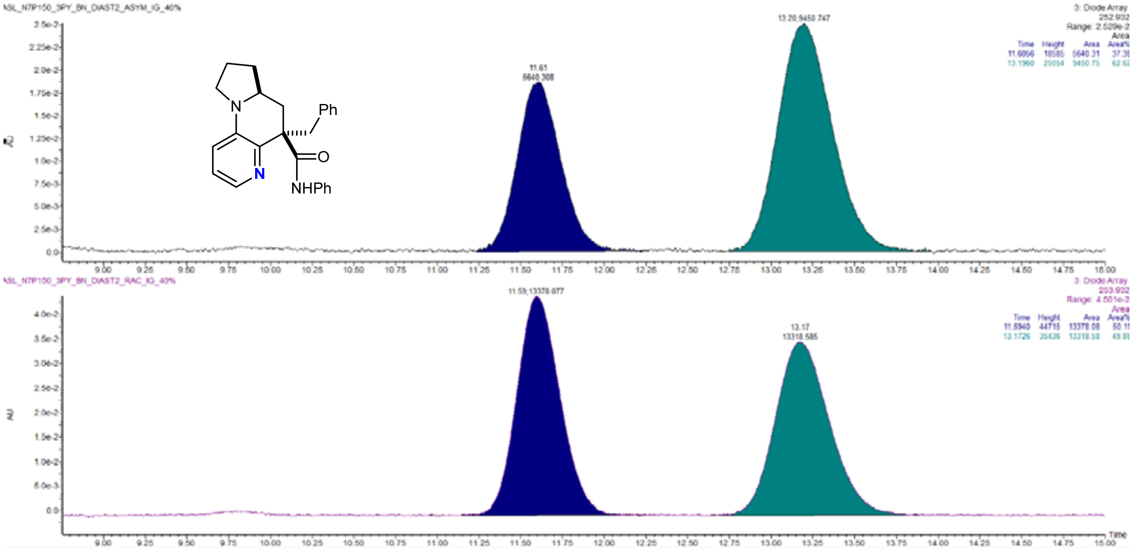

_N_-(3-(Phenyl(pyridin-2-yl)amino)butyl)benzamide (9)

Chiral HPLC Analysis CHIRALPAK AS (*n*-Hexane: iPrOH, 95:5, 1.0 mL min^{-1}, 40 °C, 240 nm) indicated 98% ee, tR = 18.3 (major), 22.5 (minor) minutes.

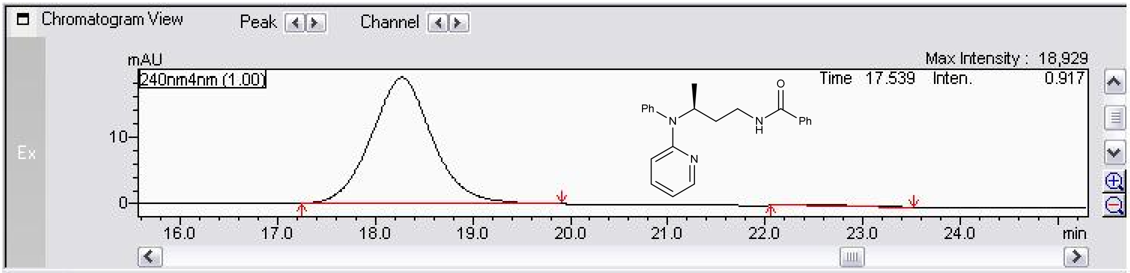

Results View - Peak Table

Peak Table | Compound | Group | Calibration Curve

Peak#	Ret. Time	Area	Height	Area%
1	18.273	823502	18849	98.968
2	22.491	8587	183	1.032
Total		832089	19032	100.000

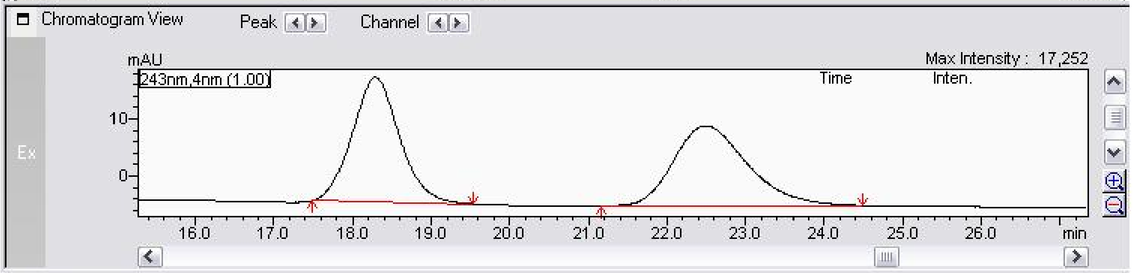

Results View - Peak Table

Peak Table | Compound | Group | Calibration Curve

Peak#	Ret. Time	Area	Height	Area%
1	18.285	909149	21742	49.951
2	22.503	910924	13961	50.049
Total		1820073	35702	100.000

(*S*)-2-Benzyl-*N*-methyl-*N*-phenyl-3-((*S*)-1-(pyridin-2-yl)pyrrolidin-2-yl)propenamide (5na) and (*R*)-2-benzyl-*N*-methyl-*N*-phenyl-3-((*S*)-1-(pyridin-2-yl)pyrrolidin-2-yl)propenamide (5nb)

Chiral SFC Analysis: CHIRAL ART SC (CO_2: MeOH, 75:25, 2.5 mL min^{-1}, 40 °C, 247 nm) indicated 46% ee, tR = 5.9 (minor), 13.4 (major) minutes.

Chiral SFC Analysis: CHIRAL ART SC (CO_2: MeOH, 75:25, 2.5 mL min^{-1}, 40 °C, 247 nm) indicated 8% ee, tR = 7.0 (minor), 9.2 (major) minutes.

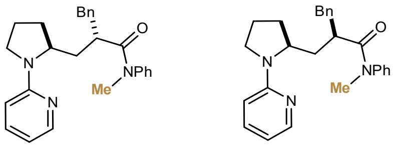

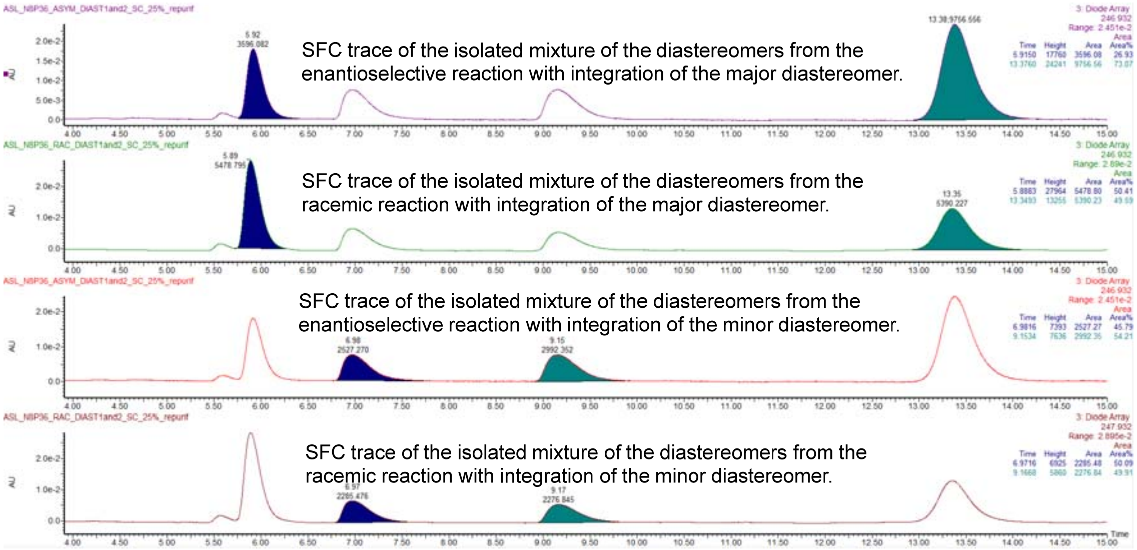

(*R*)-2-Benzyl-*N*-mesityl-3-((*S*)-1-(pyridin-2-yl)pyrrolidin-2-yl)propenamide (5oa)

Chiral SFC Analysis: CHIRALPAK IG (CO_2: MeOH, 65:35, 2.5 mL min^{-1}, 40 °C, 246 nm) indicated 39% ee, tR = 7.3 (minor), 8.2 (major) minutes.

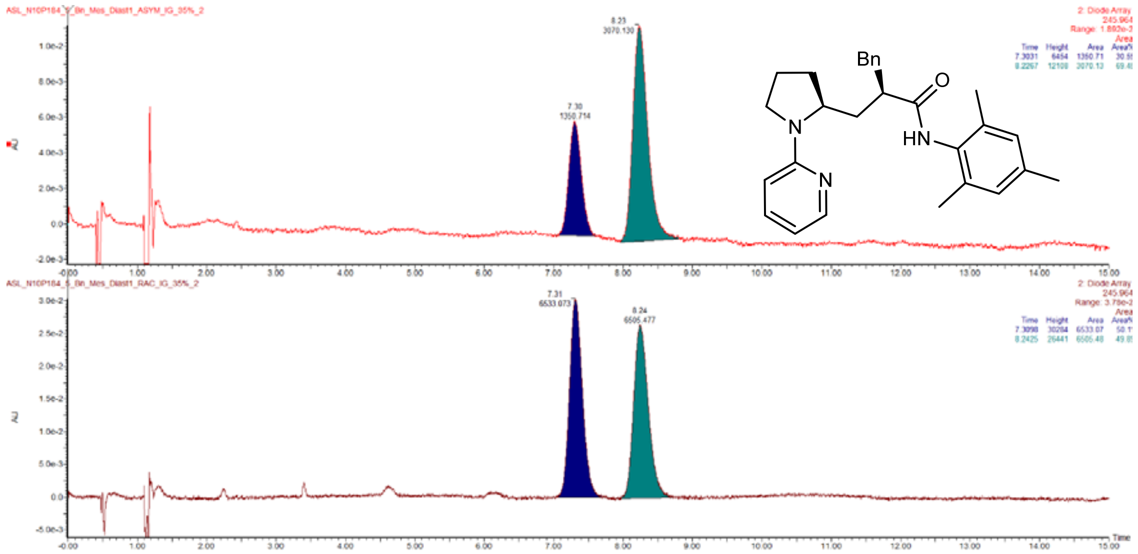

(*S*)-2-Benzyl-*N*-mesityl-3-((*S*)-1-(pyridin-2-yl)pyrrolidin-2-yl)propenamide (5ob)

Chiral SFC Analysis: CHIRALPAK IG (CO_2: MeOH, 75:25, 2.5 mL min^{-1}, 40 °C, 203 nm) indicated 22% ee, tR = 9.5 (minor), 13.3 (major) minutes.

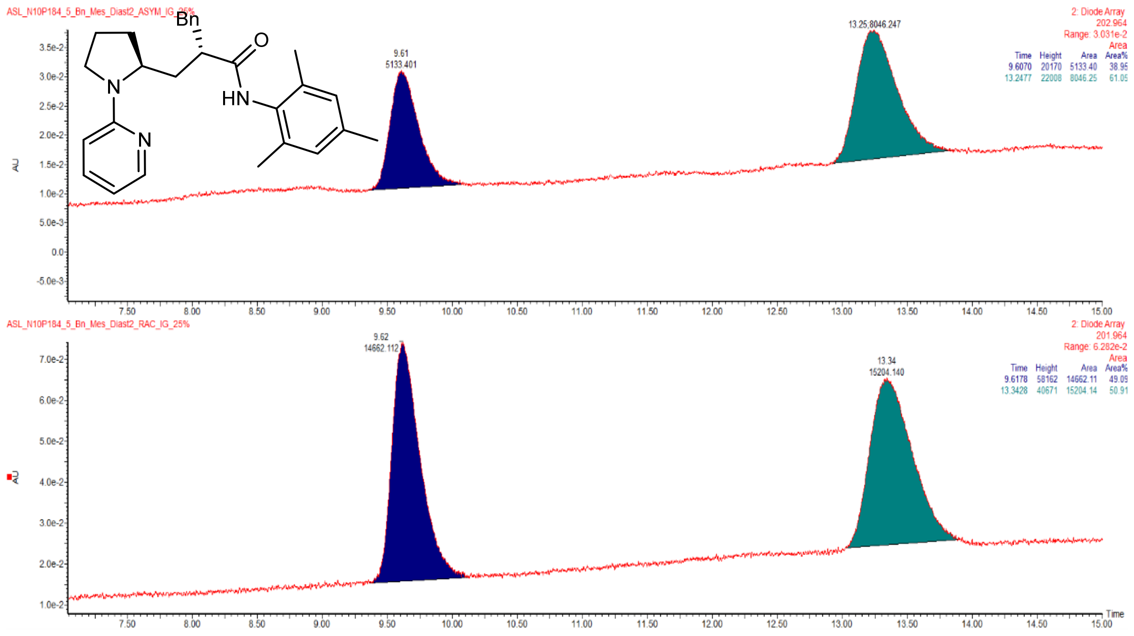

(*S*)-4-(Methyl(pyridin-2-yl)amino)-*N*,3-diphenylbutanamide

Chiral SFC Analysis: CHIRALPAK IG (CO_2: MeOH, 80:20, 2.5 mL min^{-1}, 40 °C, 242 nm) indicated 37% ee, tR = 7.4 (major), 19.3 (minor) minutes.

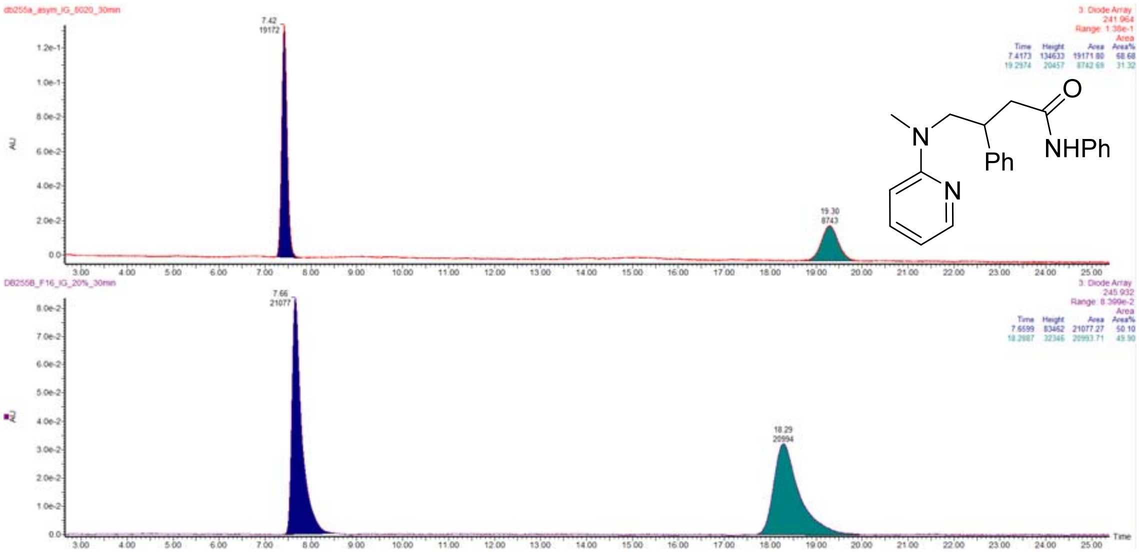

(2*R*,7a*S*)-2-Benzylhexahydro-3H-pyrrolizin-3-one (5be)

Chiral SFC Analysis: CHIRALPAK IG (CO_2: MeOH, 80:20, 2.5 mL min^{-1}, 40 °C, 203 nm) indicated 94% ee, tR = 6.01 (major), 6.43 (minor) minutes.

(2*S*,7a*S*)-2-Benzylhexahydro-3H-pyrrolizin-3-one (5bf)

Chiral SFC Analysis: CHIRALPAK IG (CO_2: MeOH, 80:20, 2.5 mL min^{-1}, 40 °C, 203 nm) indicated 90% ee, tR = 4.31 (major), 7.23 (minor) minutes.

Analytical epimerisation experiment of the chiral products for confirming the α-amine stereocenter.

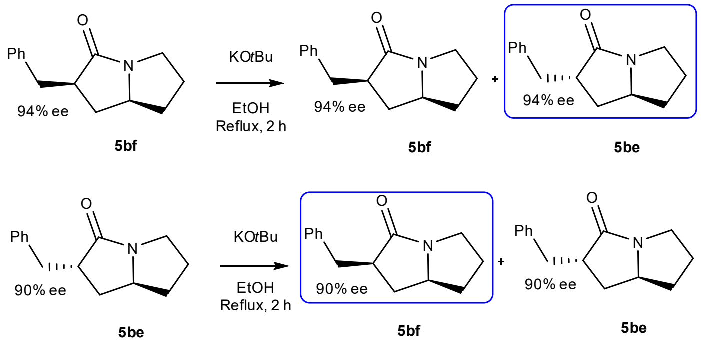

The purified products **5bf** and **5be** were treated with KO*t*Bu (4 eq) in refluxing anhydrous toluene for 2 hours. The crude material was quenched with saturated aqueous NH_4Cl and the mixture was extracted with DCM. The solvent was evaporated *in vacuo* and the crude material was analysed with chiral SFC.

After the treatment with base, both reactions showed epimerization at the α-amide stereocenter.

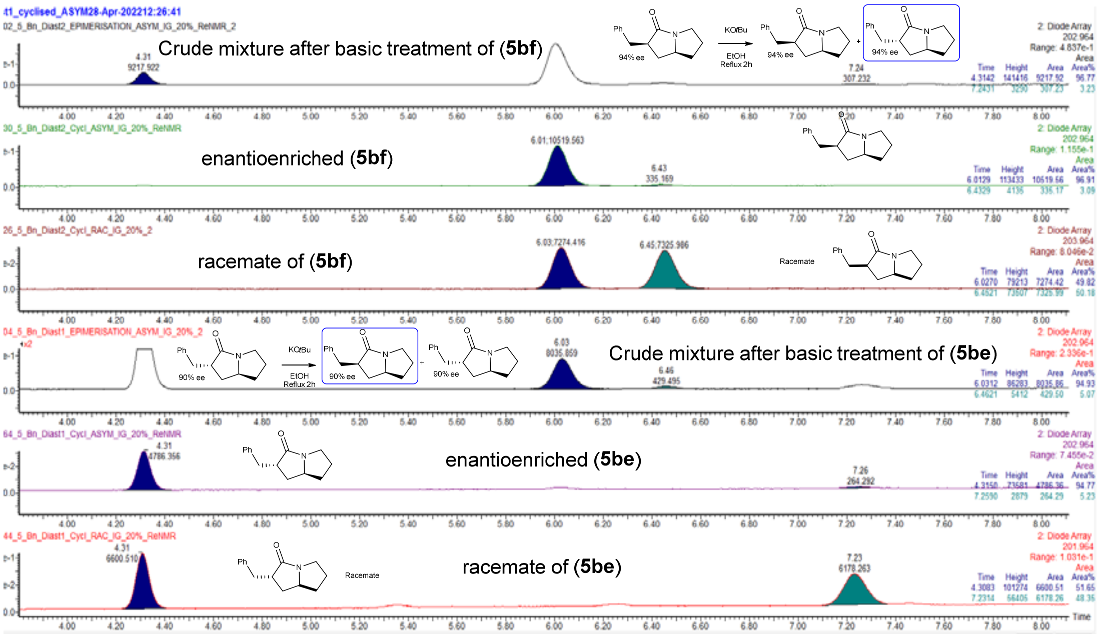

NMR Spectra

^{1}H NMR (400 MHz, $CDCl_3$) for ***N*-Phenyl-3-(1-(pyridin-2-yl)pyrrolidin-2-yl)propanamide (5a)**

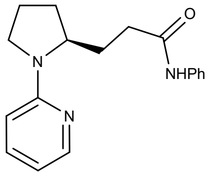

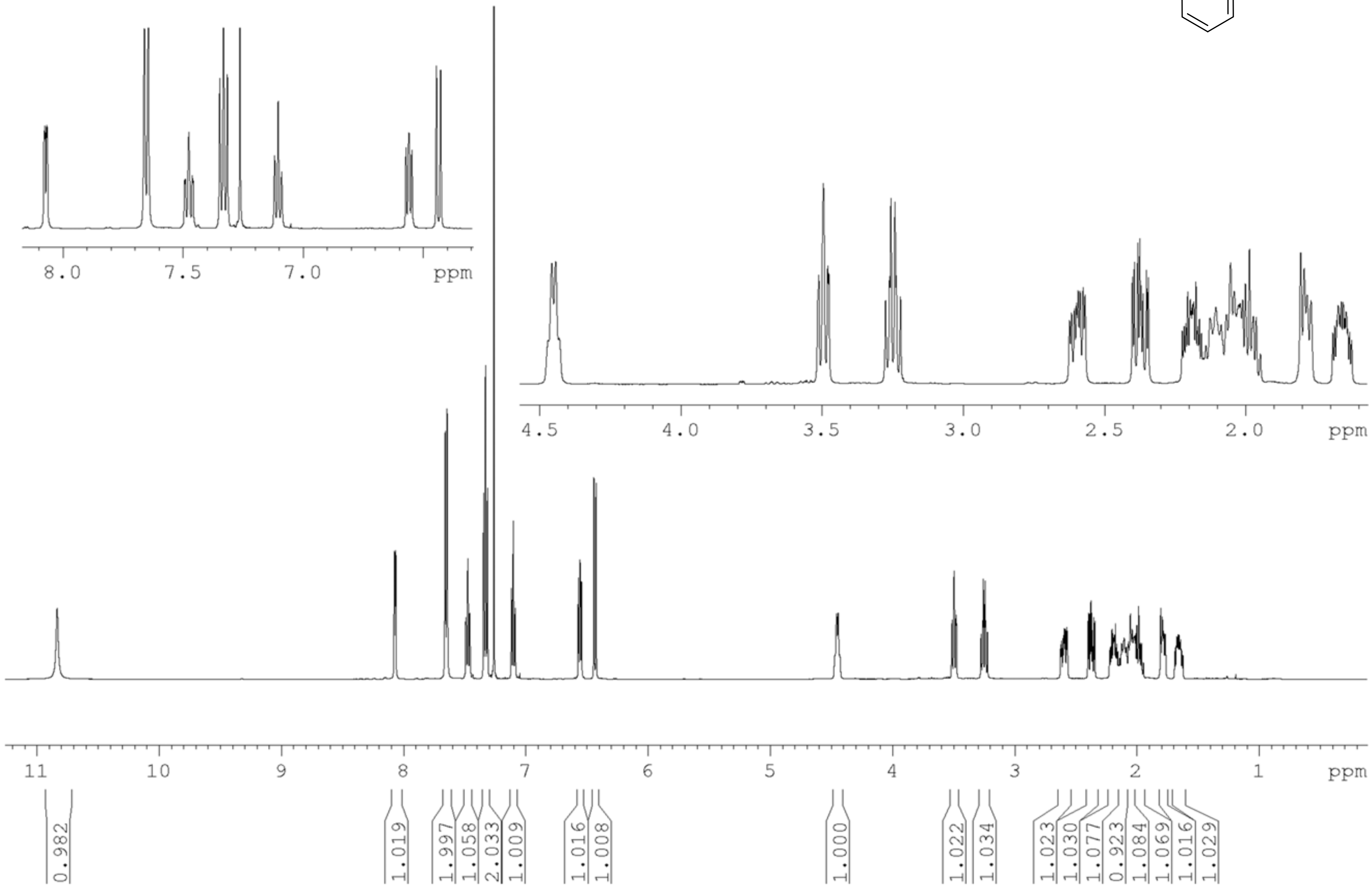

^{13}C NMR (126 MHz, $CDCl_3$) for ***N*-Phenyl-3-(1-(pyridin-2-yl)pyrrolidin-2-yl)propanamide (5a)**

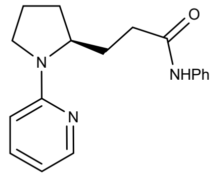

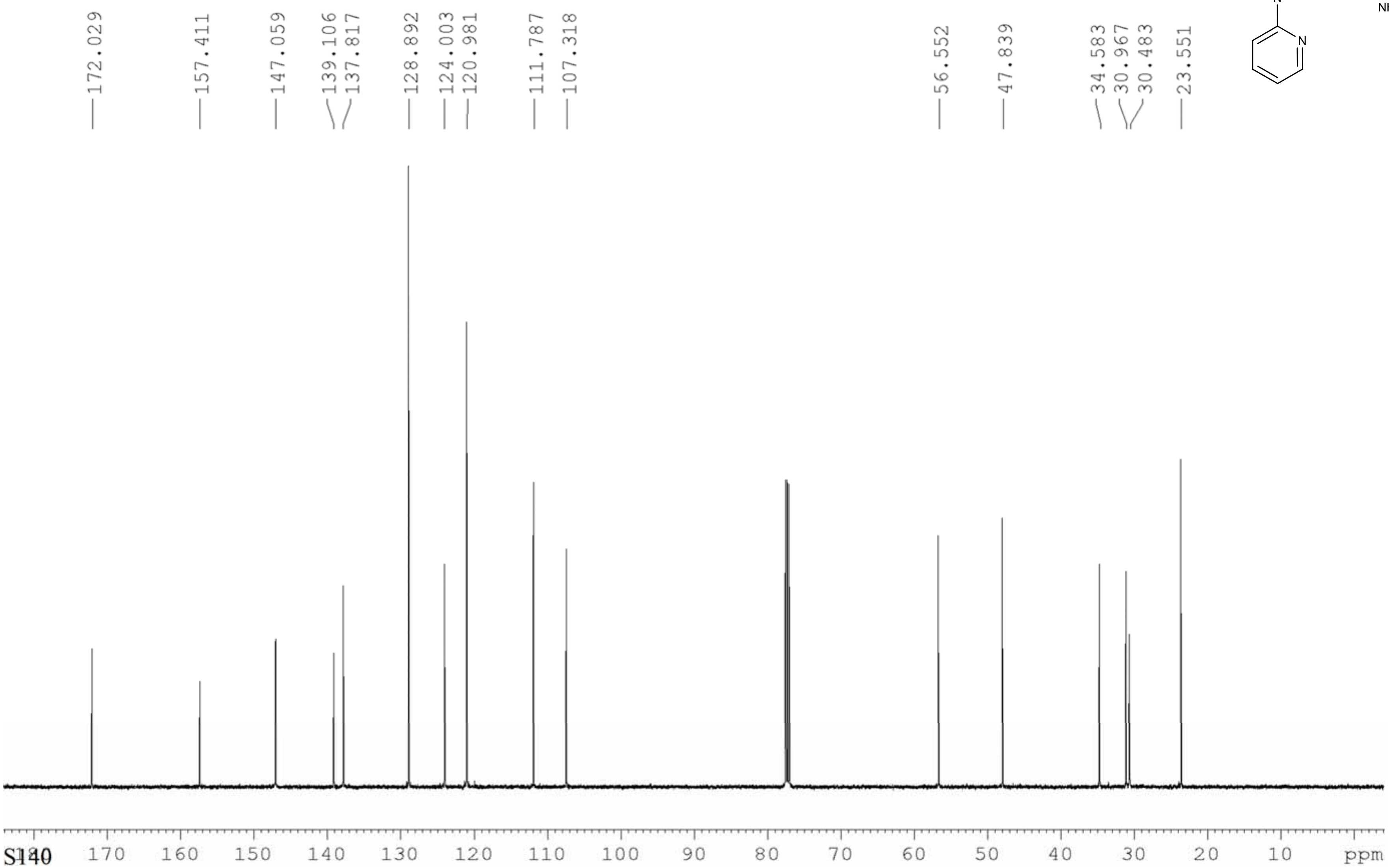

^{13}C NMR (101 MHz, $CDCl_3$) for **(*S*)-2-Benzyl-*N*-phenyl-3-((*S*)-1-(pyridin-2-yl)pyrrolidin-2-yl)propanamide (5ba)**

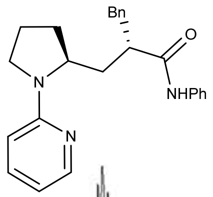

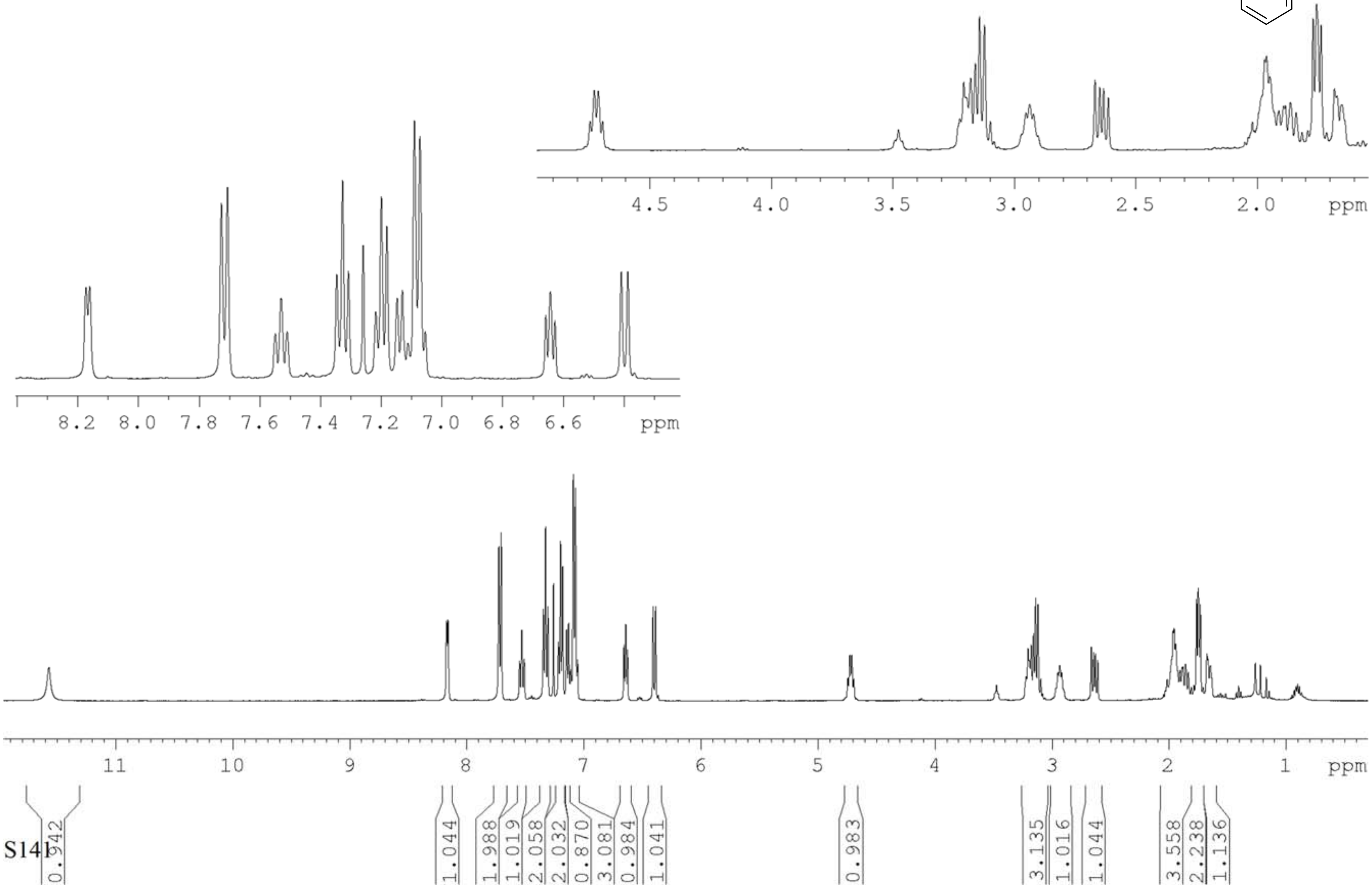

^{13}C NMR (101 MHz, $CDCl_3$) for **(*S*)-2-Benzyl-*N*-phenyl-3-((*S*)-1-(pyridin-2-yl)pyrrolidin-2-yl)propanamide (5ba)**

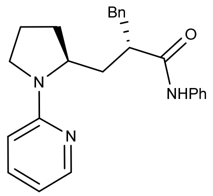

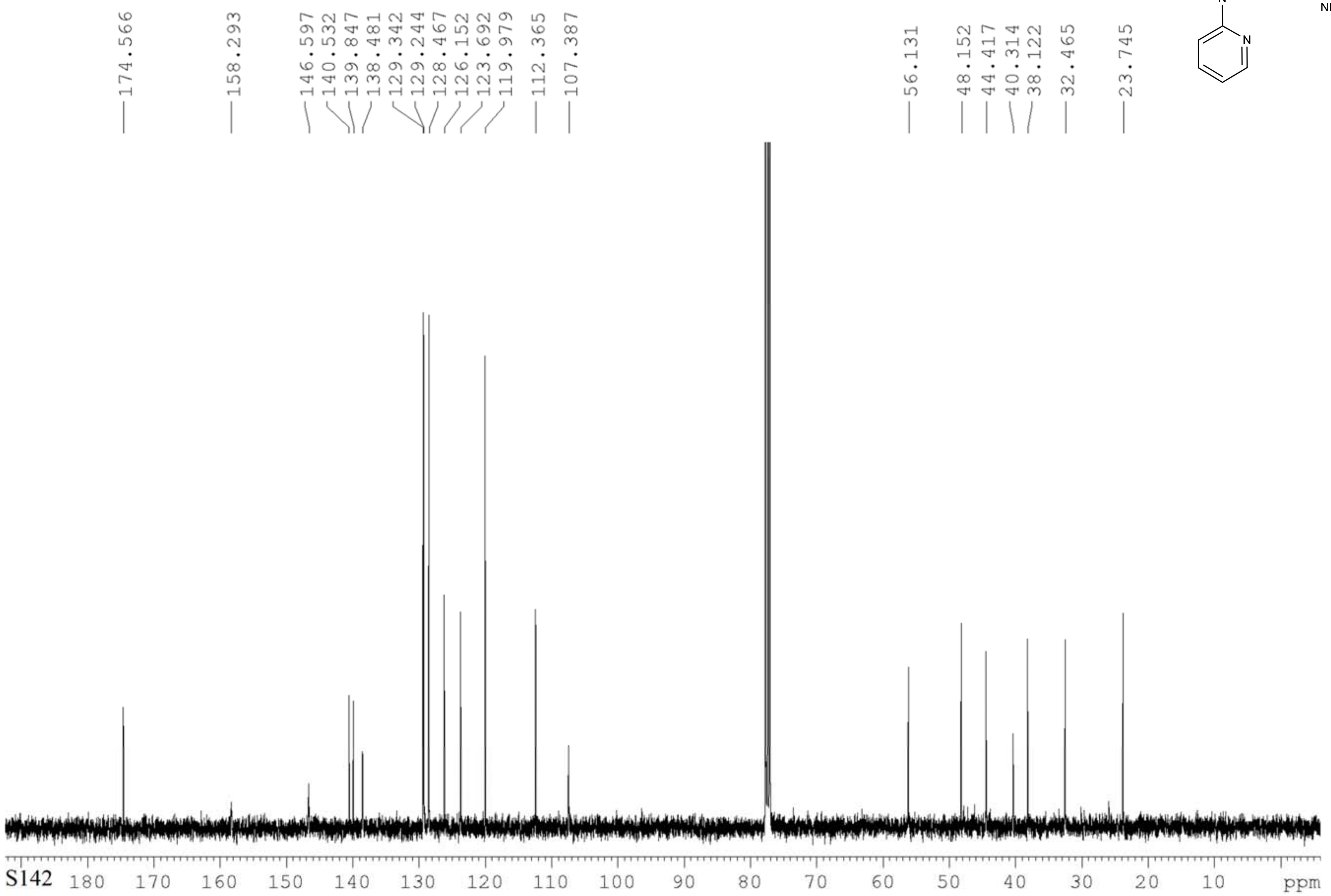

NOESY NMR (500 MHz, $CDCl_3$) for ***(S)*-2-Benzyl-*N*-phenyl-3-((*S*)-1-(pyridin-2-yl)pyrrolidin-2-yl)propanamide (5ba)**

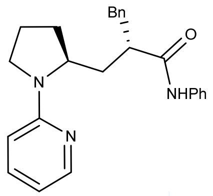

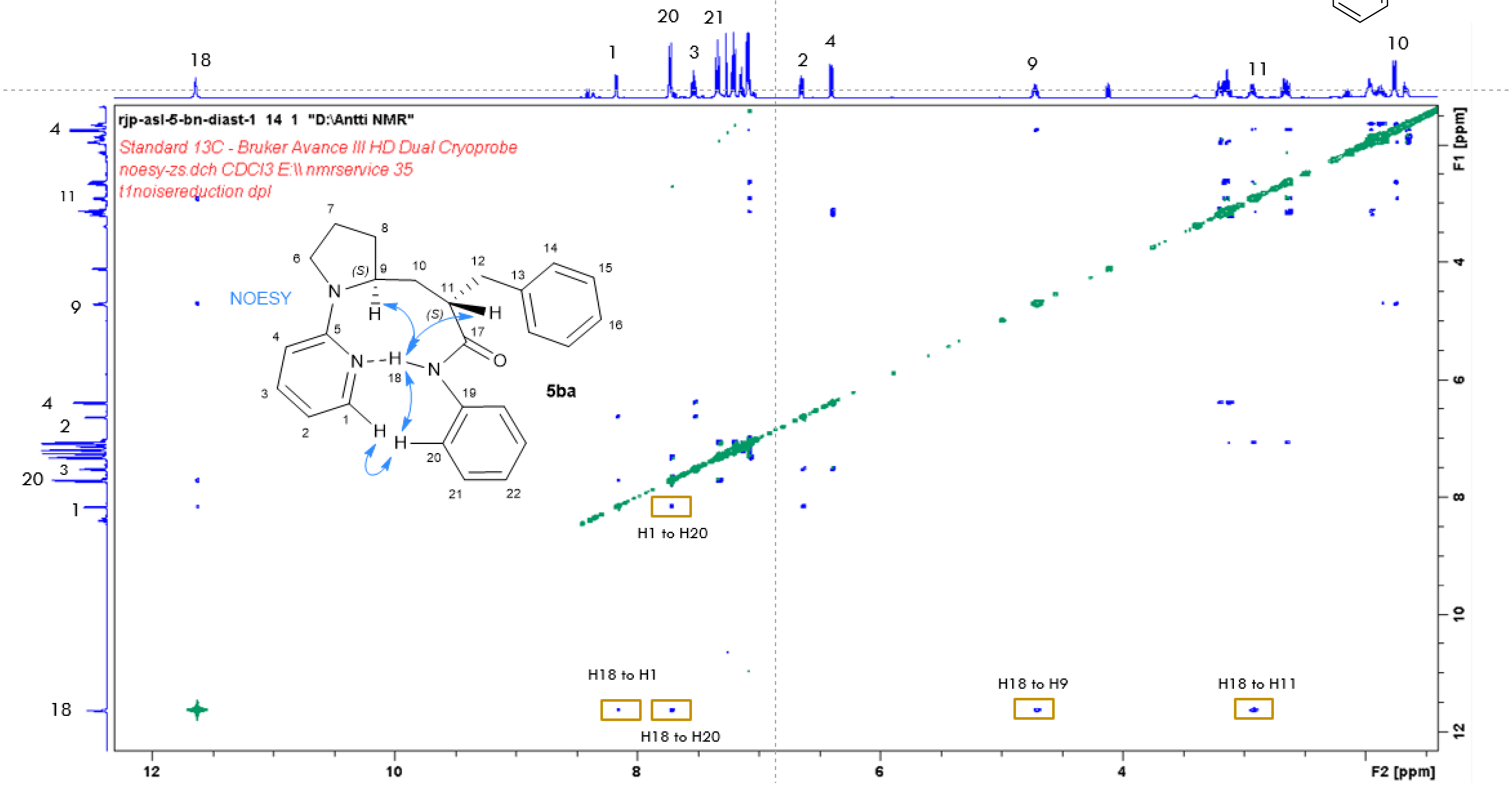

^{1}**H NMR** (400 MHz, $CDCl_3$) for **(*R*)-2-Benzyl-*N*-phenyl-3-((*S*)-1-(pyridin-2-yl)pyrrolidin-2-yl)propanamide (5bb)**

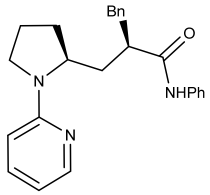

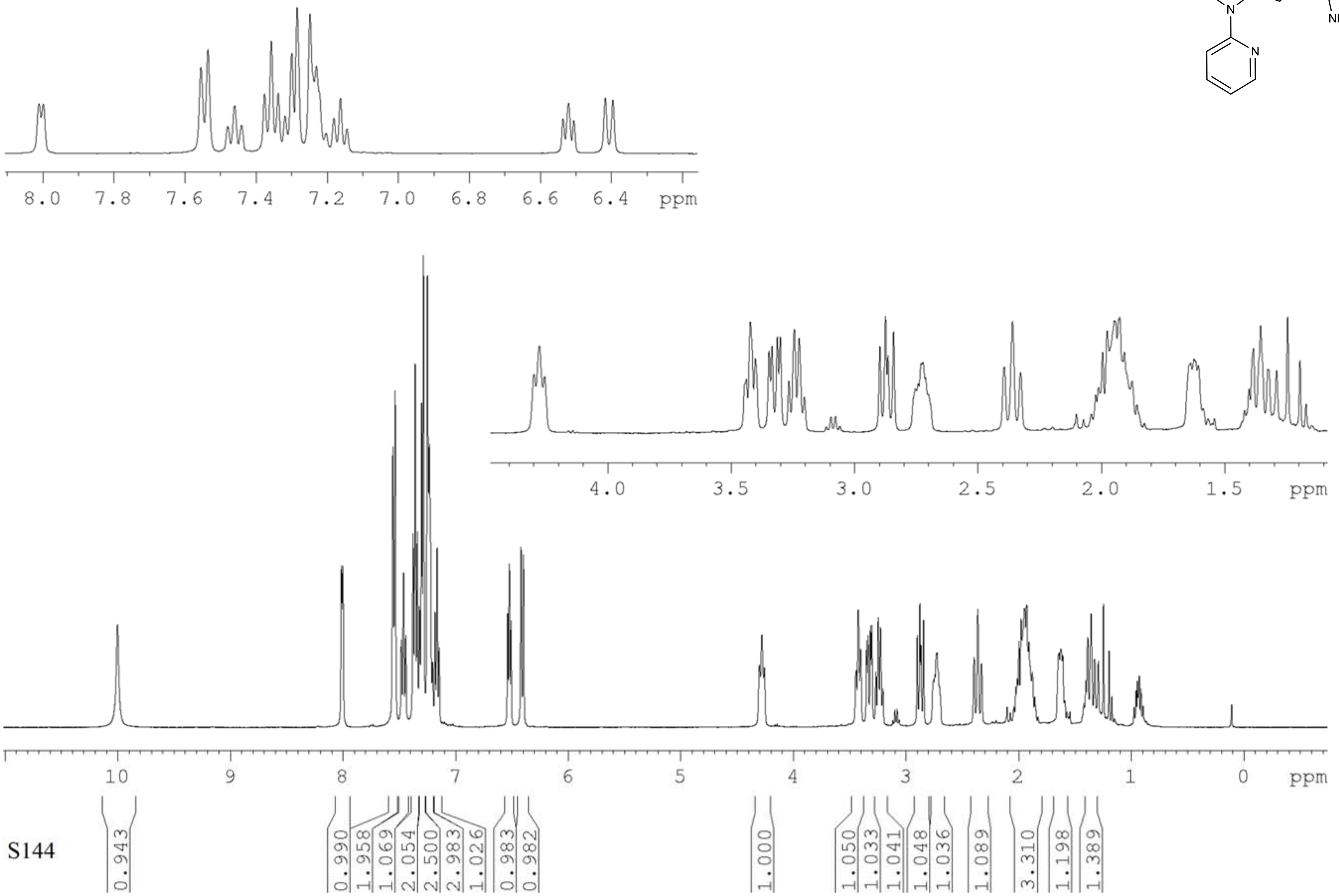

^{13}C NMR (101 MHz, $CDCl_3$) for **(*R*)-2-Benzyl-*N*-phenyl-3-((*S*)-1-(pyridin-2-yl)pyrrolidin-2-yl)propanamide (5bb)**

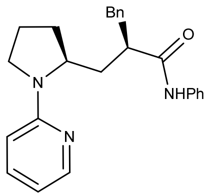

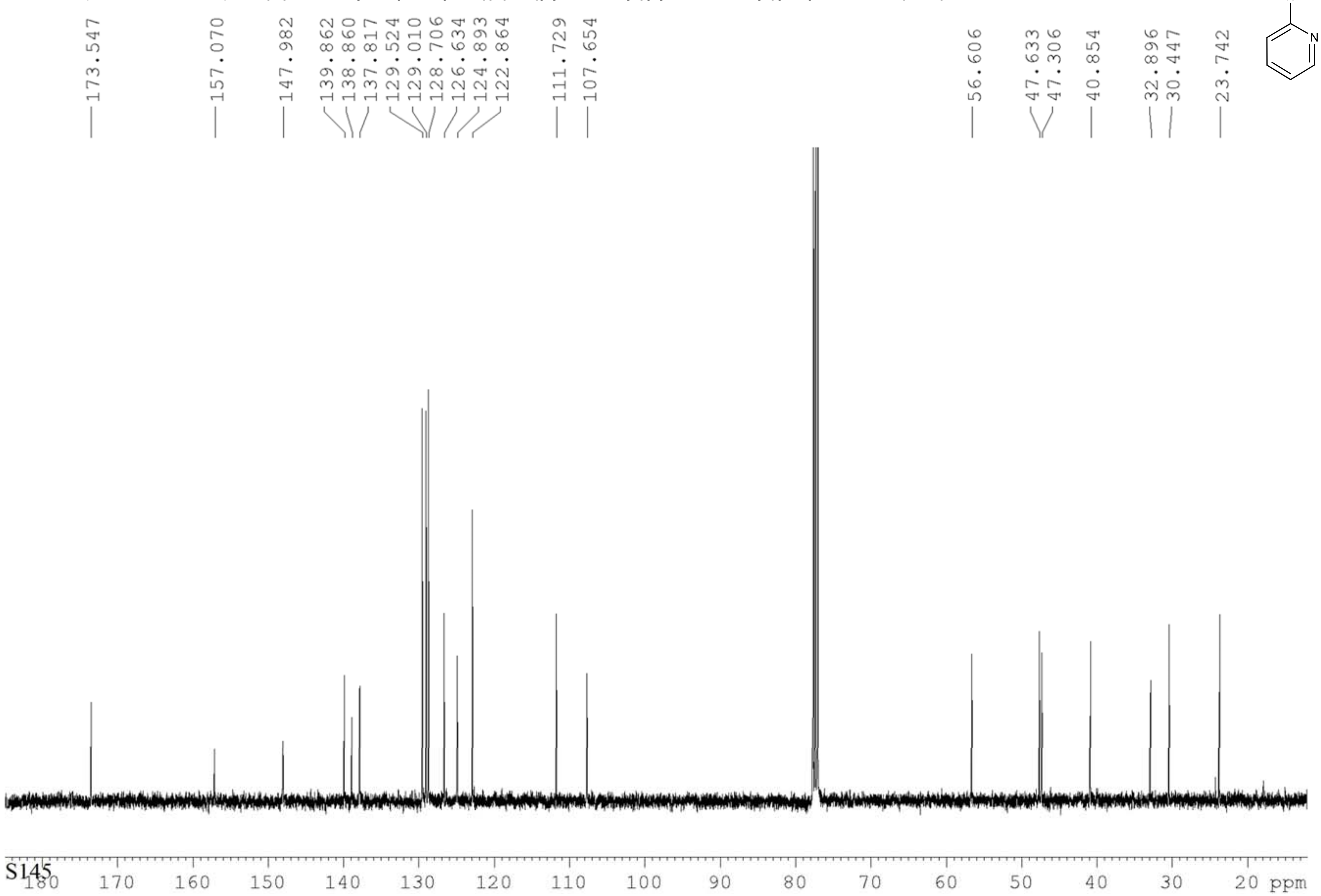

NOESY NMR (500 MHz, $CDCl_3$) for **(*R*)-2-Benzyl-*N*-phenyl-3-((*S*)-1-(pyridin-2-yl)pyrolidin-2-yl)propenamide (5bb)**

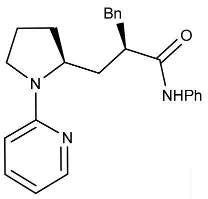

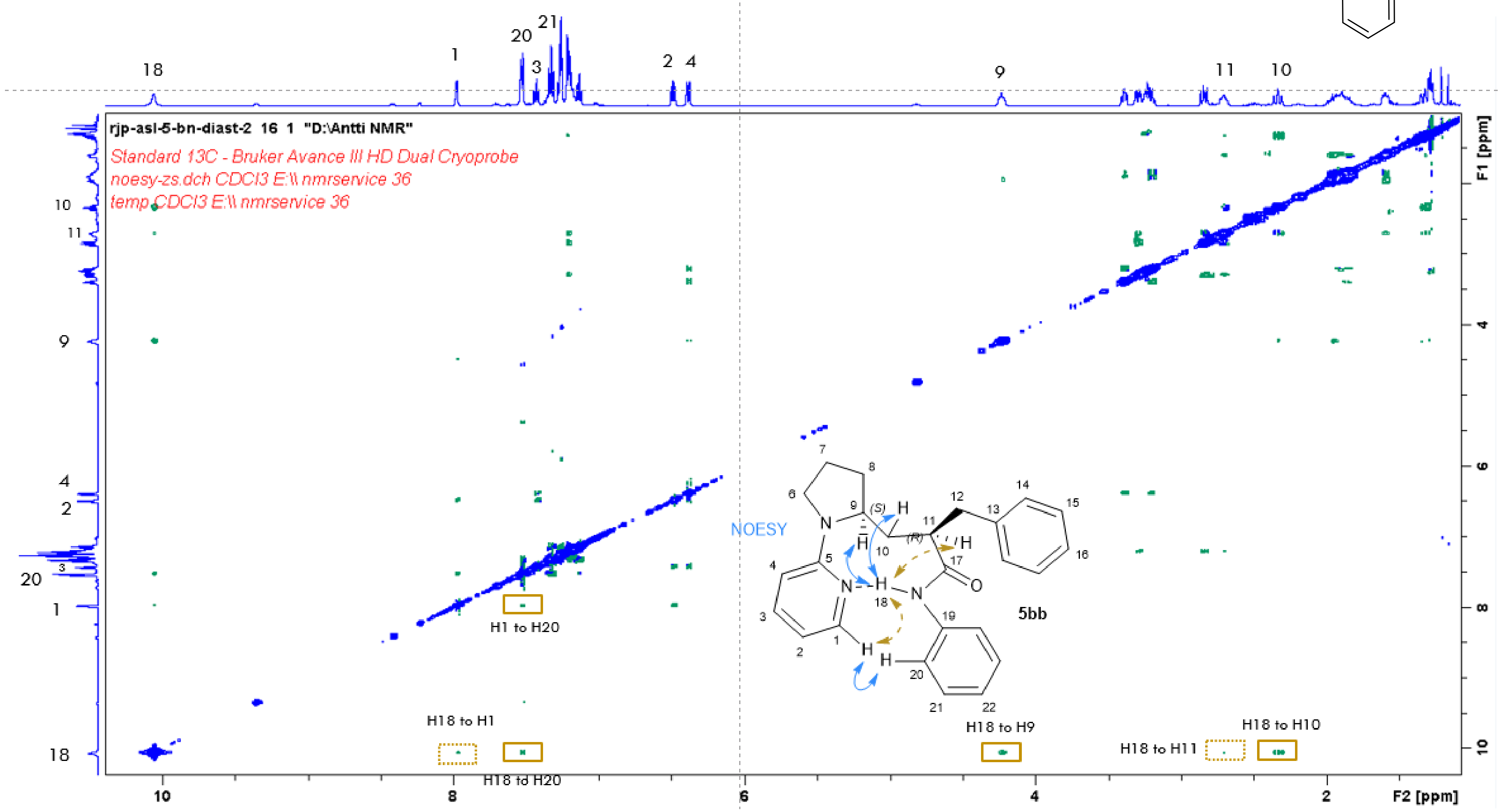

^{1}H NMR (400 MHz, $CDCl_3$) for **(*S*)-3-Methyl-*N*-phenyl-2-(((*S*)-1-(pyridin-2-yl)pyrrolidin-2-yl)methyl)butanamide (5ca)**

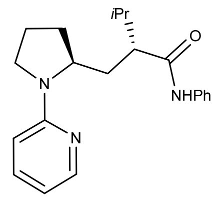

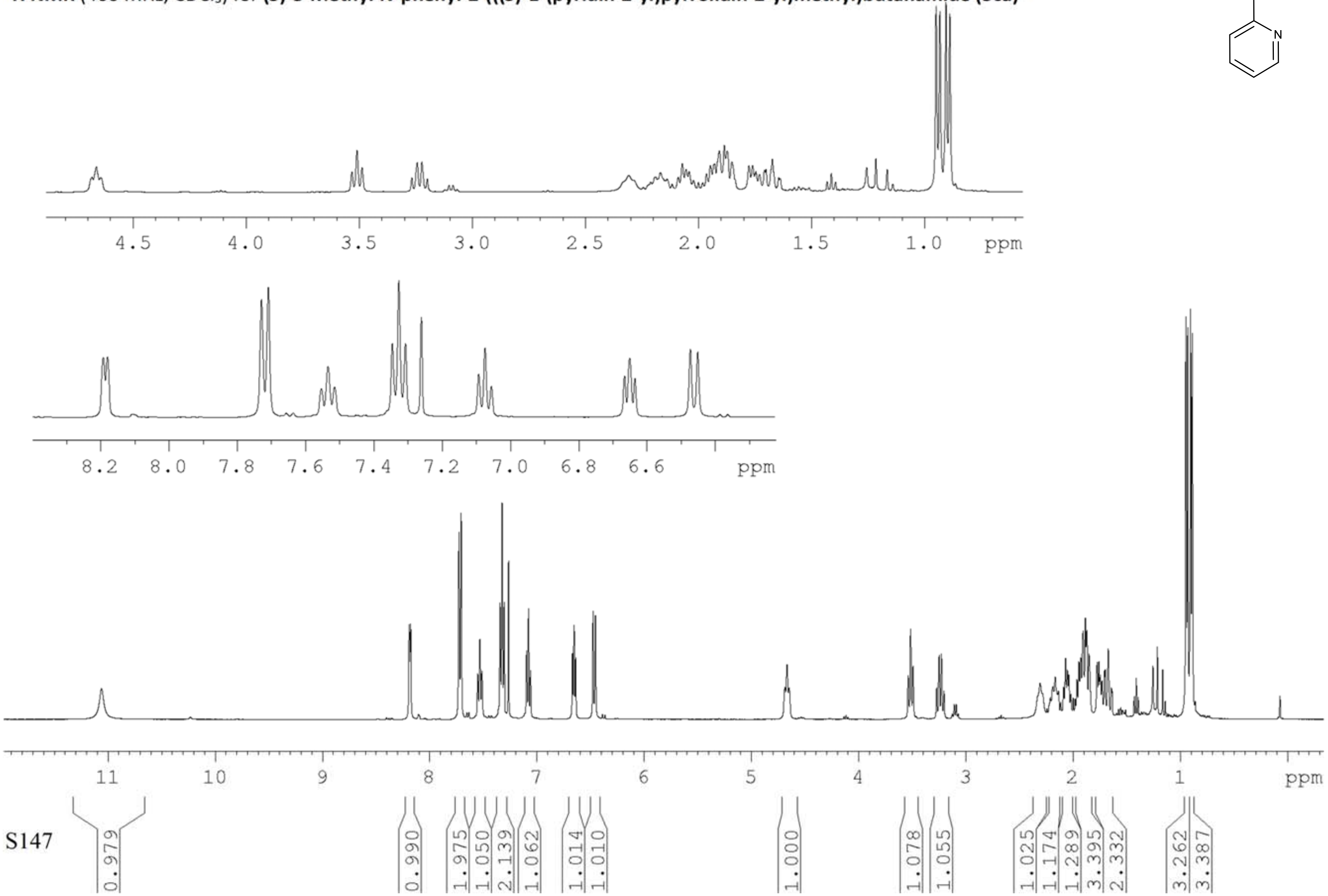

^{13}C NMR (101 MHz, $CDCl_3$) for **(*S*)-3-Methyl-*N*-phenyl-2-(((*S*)-1-(pyridin-2-yl)pyrrolidin-2-yl)methyl)butanamide (5ca)**

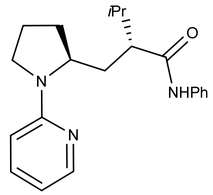

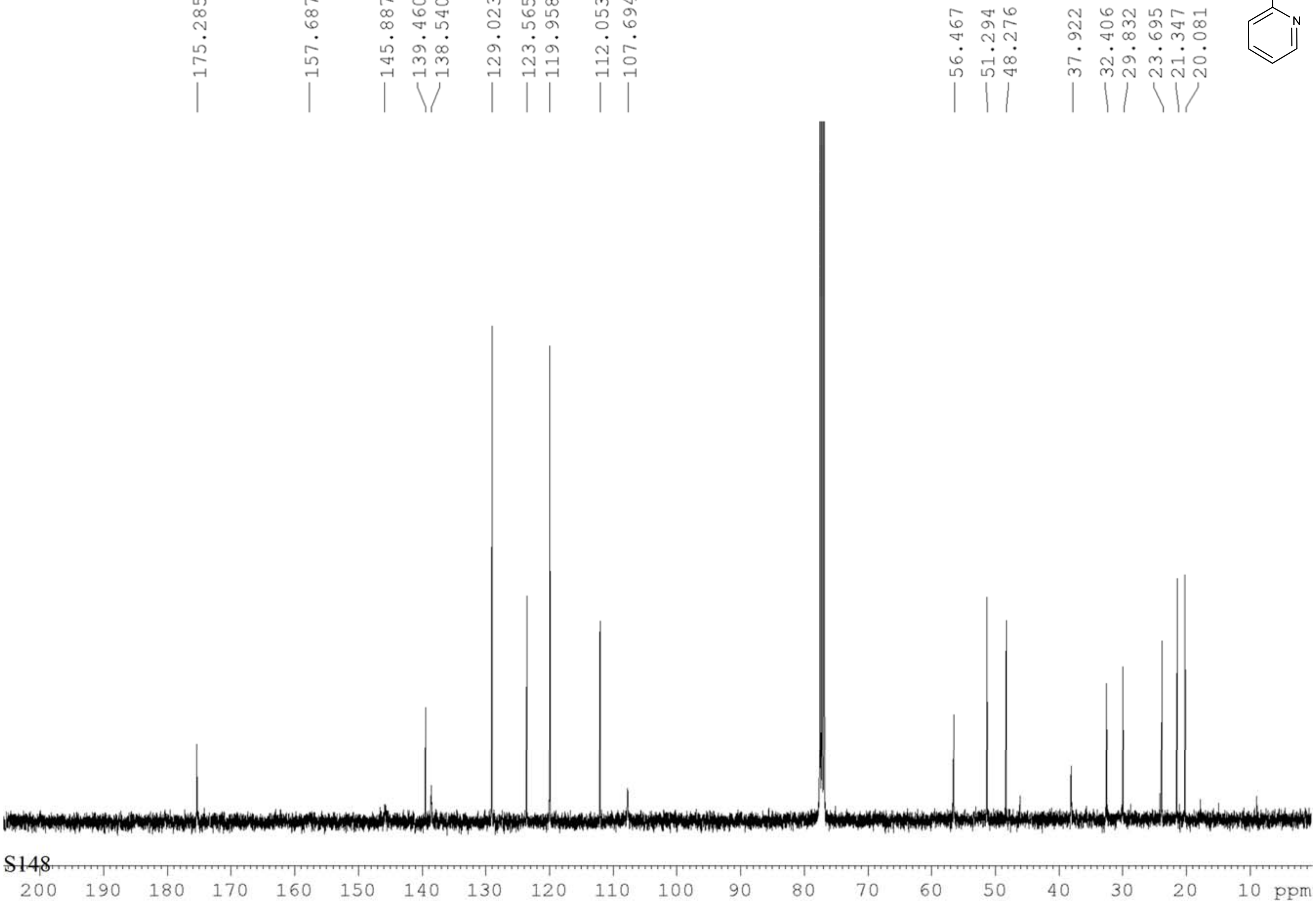

^{1}H NMR (400 MHz, $CDCl_3$) for **(*S*)-3-Methyl-*N*-phenyl-2-(((*S*)-1-(pyridin-2-yl)pyrrolidin-2-yl)methyl)butanamide (5cb)**

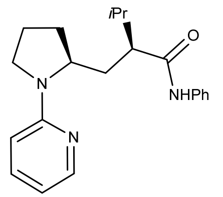

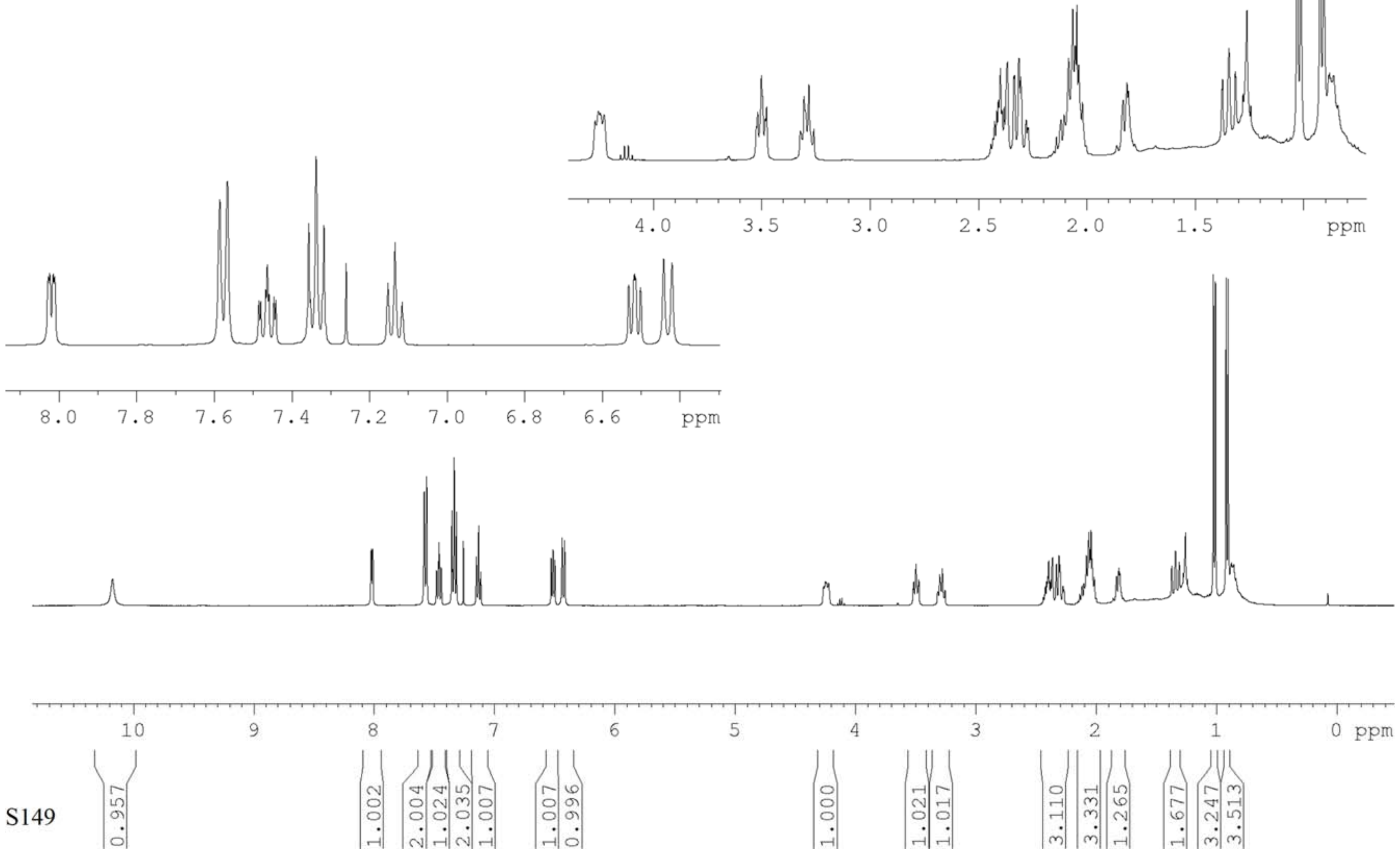

^{13}C NMR (101 MHz, $CDCl_3$) for **(*S*)-3-Methyl-*N*-phenyl-2-(((*S*)-1-(pyridin-2-yl)pyrrolidin-2-yl)methyl)butanamide (5cb)**

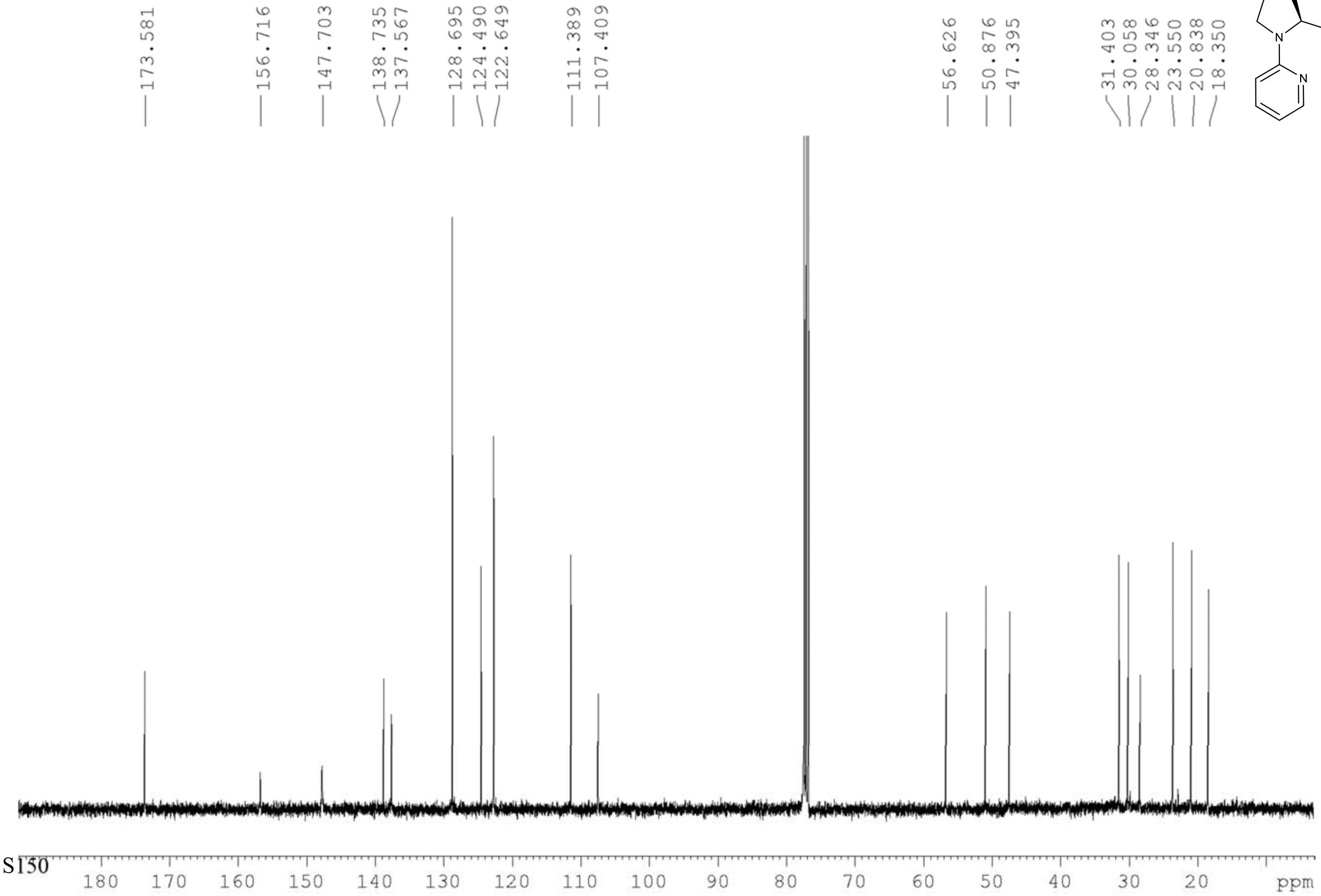

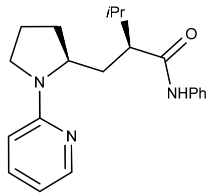

^{1}H NMR (700 MHz, $CDCl_3$) for **Methyl (*R*)-4-oxo-4-(phenylamino)-3-(((*S*)-1-(pyridin-2-yl)pyrrolidin-2-yl)methyl)butanoate (5da)**

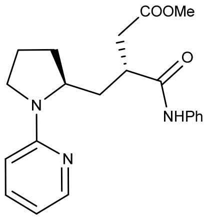

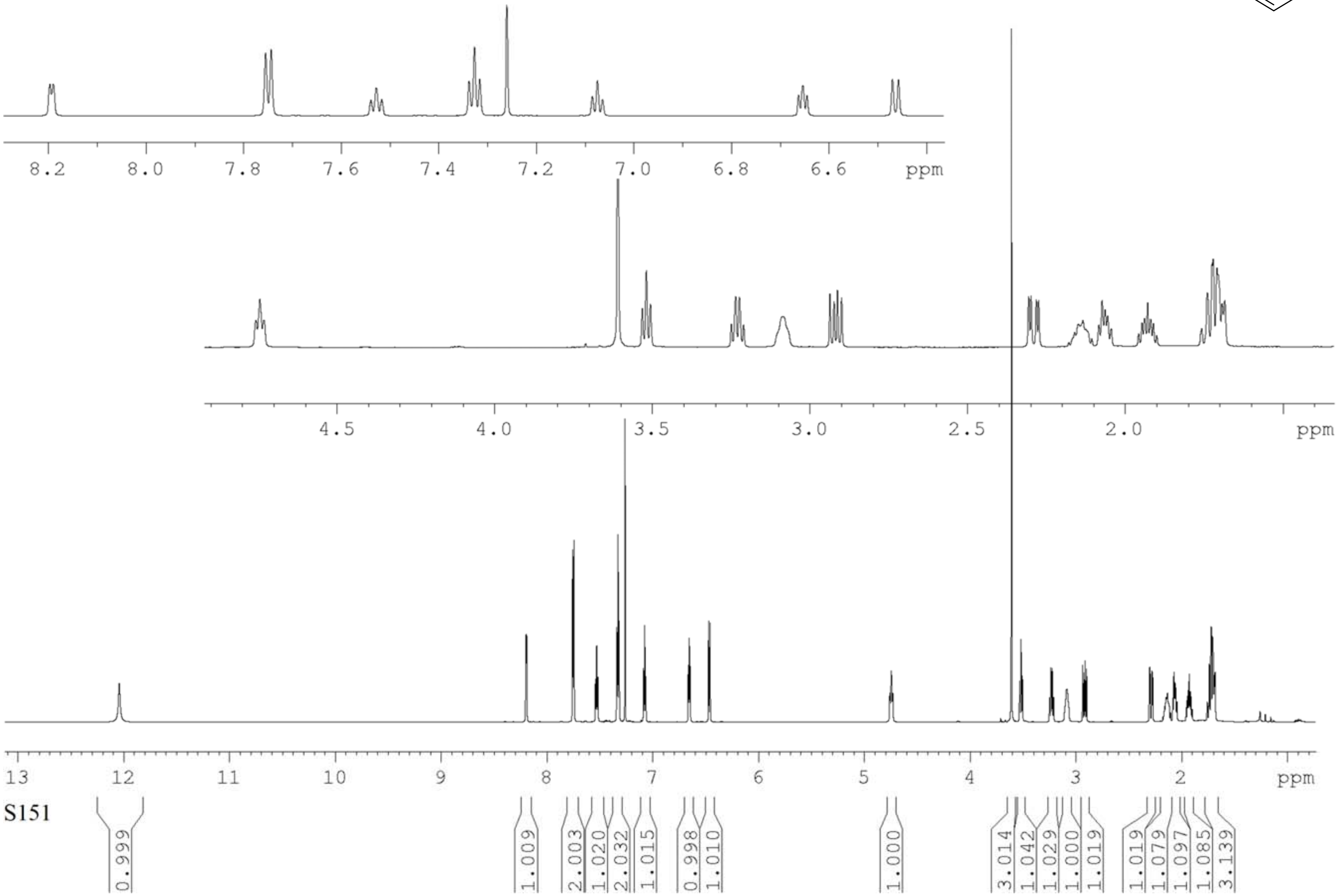

S151

^{13}C NMR (176 MHz, $CDCl_3$) for **Methyl (*R*)-4-oxo-4-(phenylamino)-3-(((*S*)-1-(pyridin-2-yl)pyrrolidin-2-yl)methyl)butanoate (5da)**

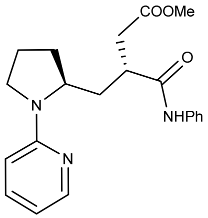

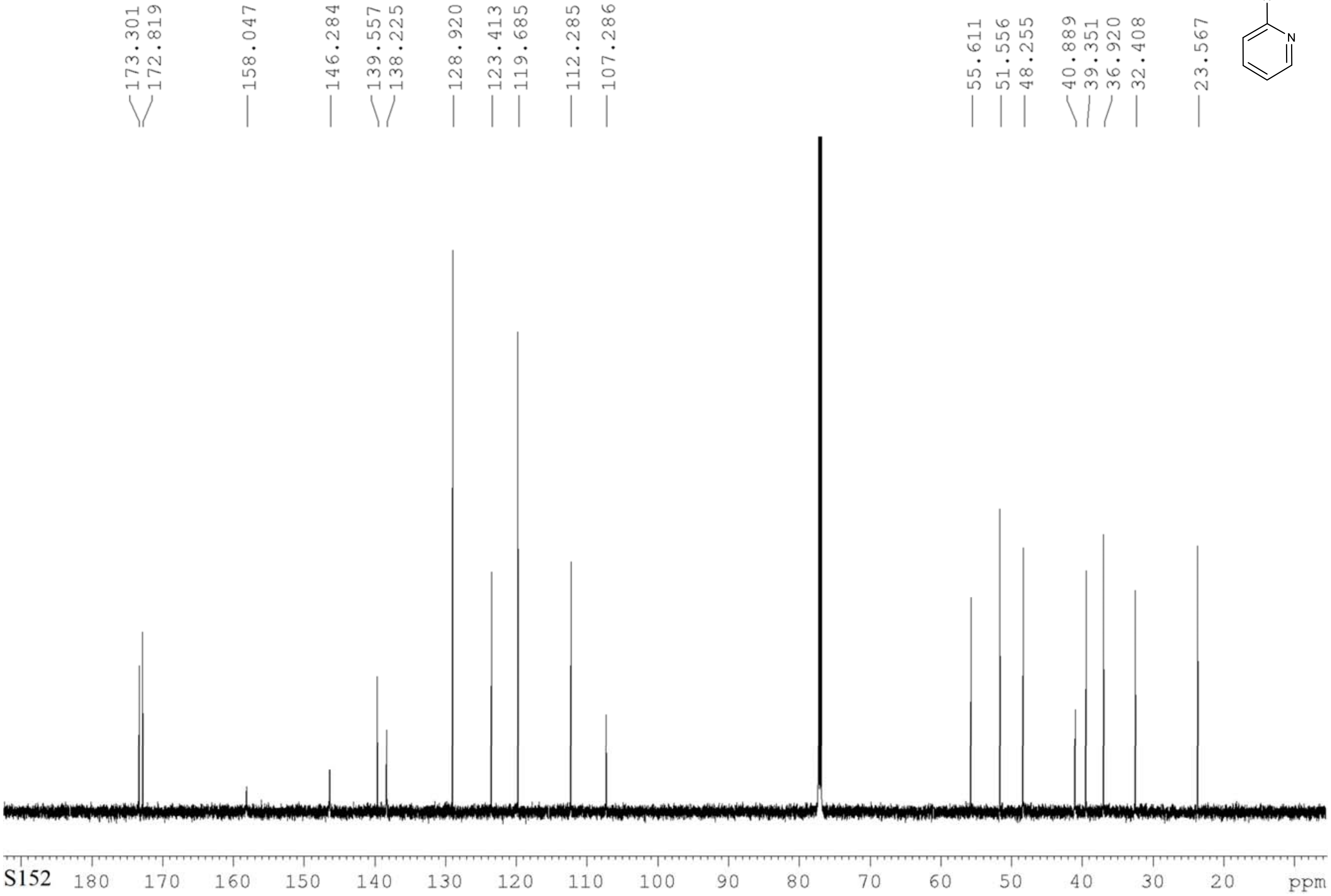

^{1}H NMR (700 MHz, $CDCl_3$) for **Methyl (*S*)-4-oxo-4-(phenylamino)-3-(((*S*)-1-(pyridin-2-yl)pyrrolidin-2-yl)methyl)butanoate (5db)**

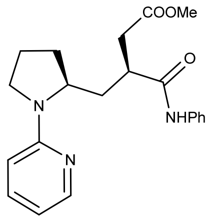

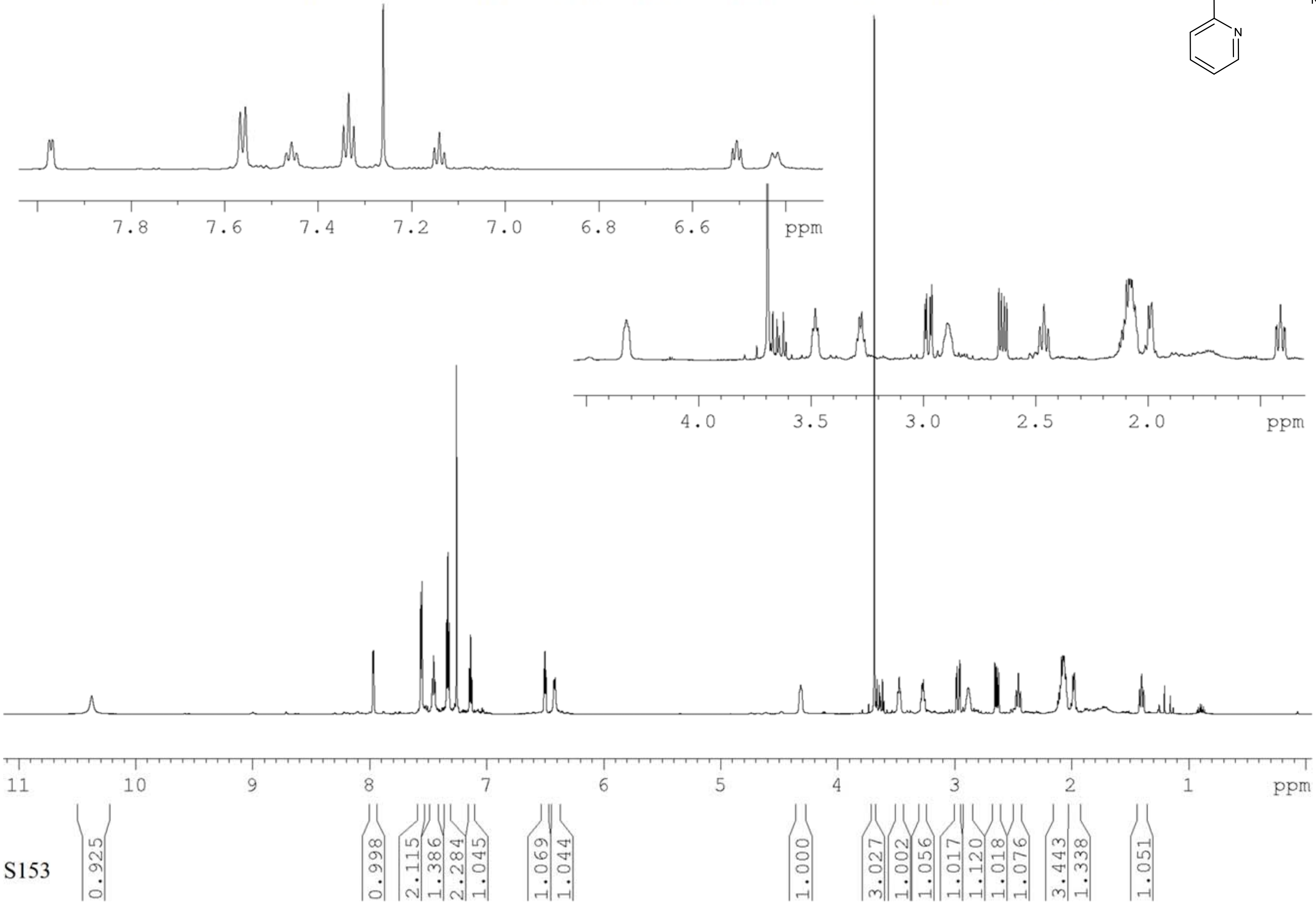

^{13}C NMR (176 MHz, $CDCl_3$) for **Methyl (*S*)-4-oxo-4-(phenylamino)-3-(((*S*)-1-(pyridin-2-yl)pyrrolidin-2-yl)methyl)butanoate (5db)**

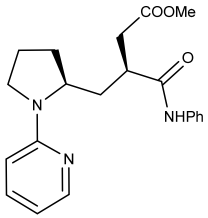

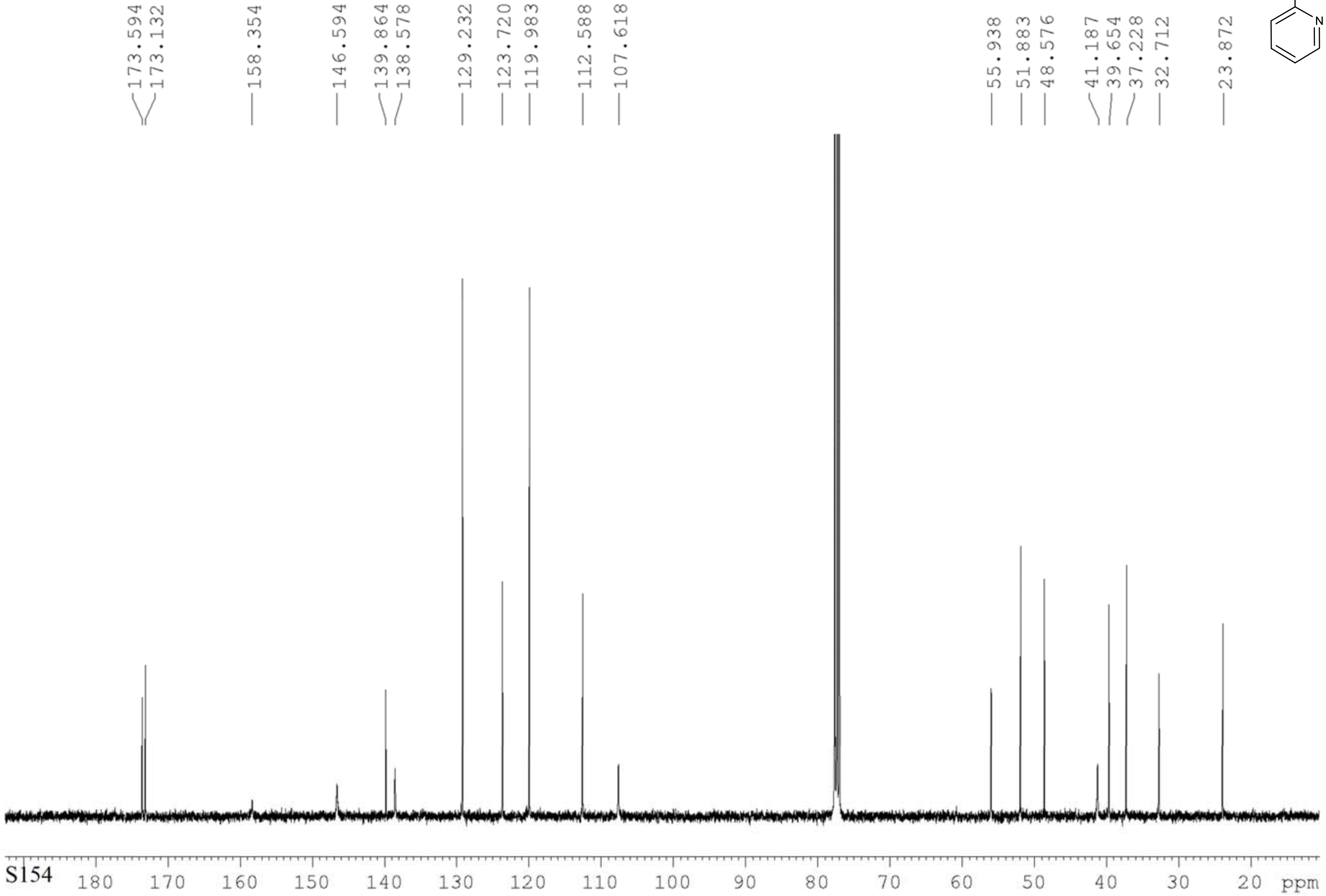

^{1}H NMR (400 MHz, $CDCl_3$) for **(*S*)-*N*-Phenyl-2-(((*S*)-1-(pyridin-2-yl)pyrrolidin-2-yl)methyl)butanamide (5ea)**

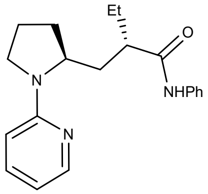

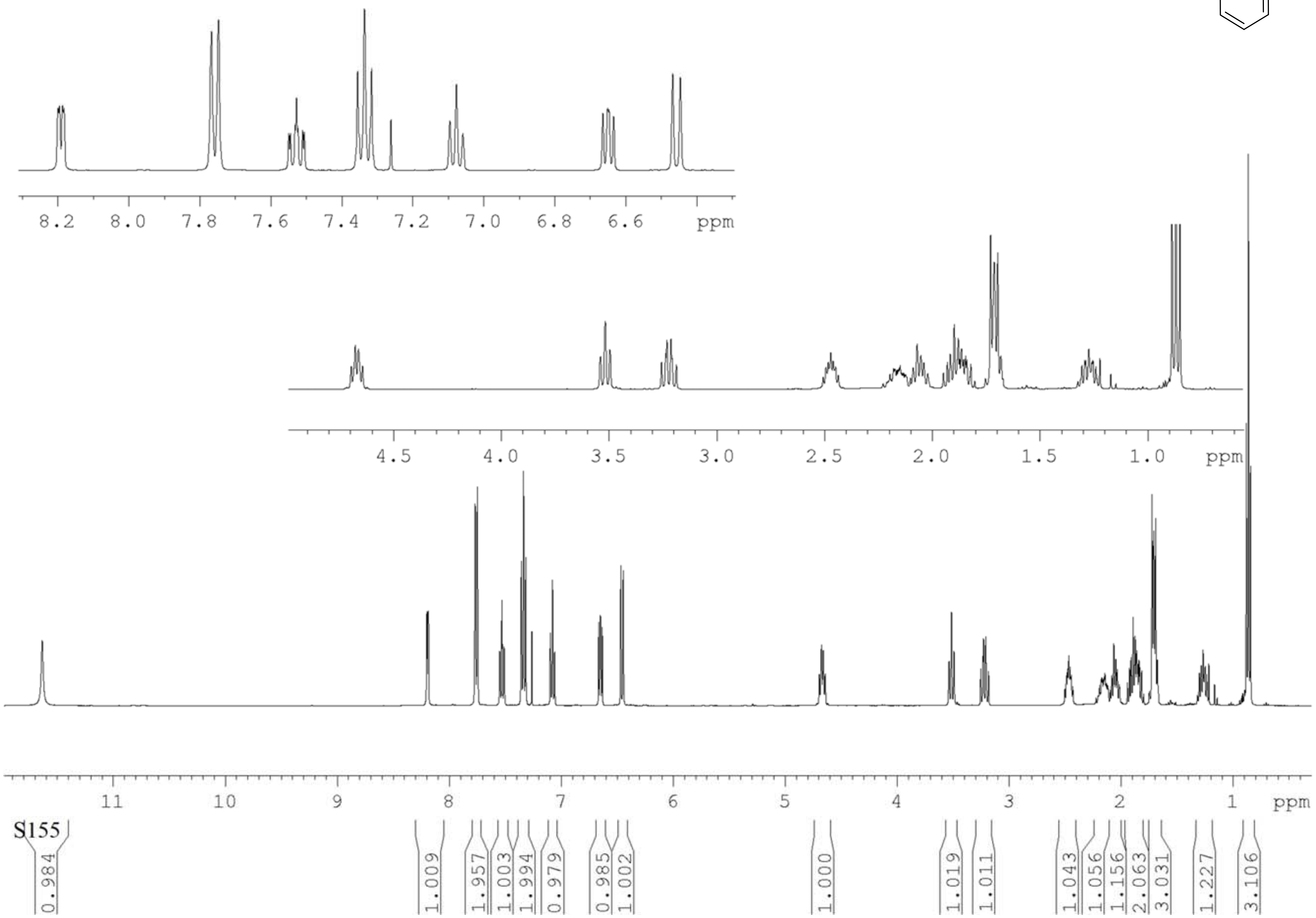

^{13}**C NMR** (101 MHz, $CDCl_3$) for ***(S)*-*N*-Phenyl-2-(((*S*)-1-(pyridin-2-yl)pyrrolidin-2-yl)methyl)butanamide (5ea)**

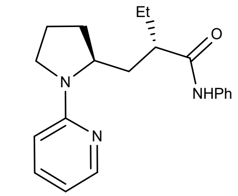

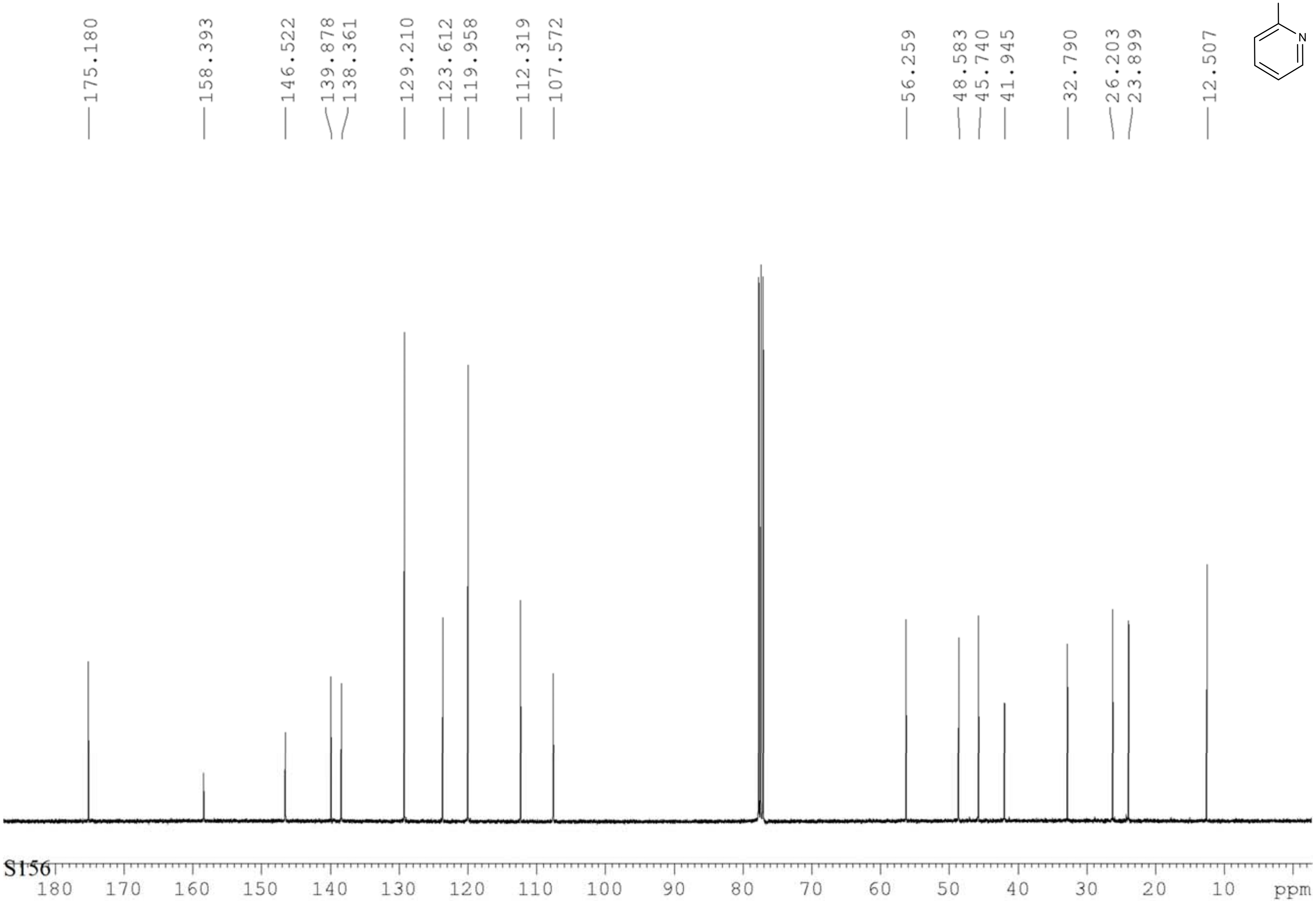

^{1}H NMR (400 MHz, $CDCl_3$) for **(*R*)-*N*-Phenyl-2-(((*S*)-1-(pyridin-2-yl)pyrrolidin-2-yl)methyl)butanamide (5eb)**

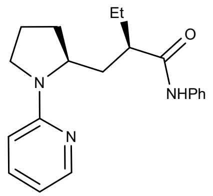

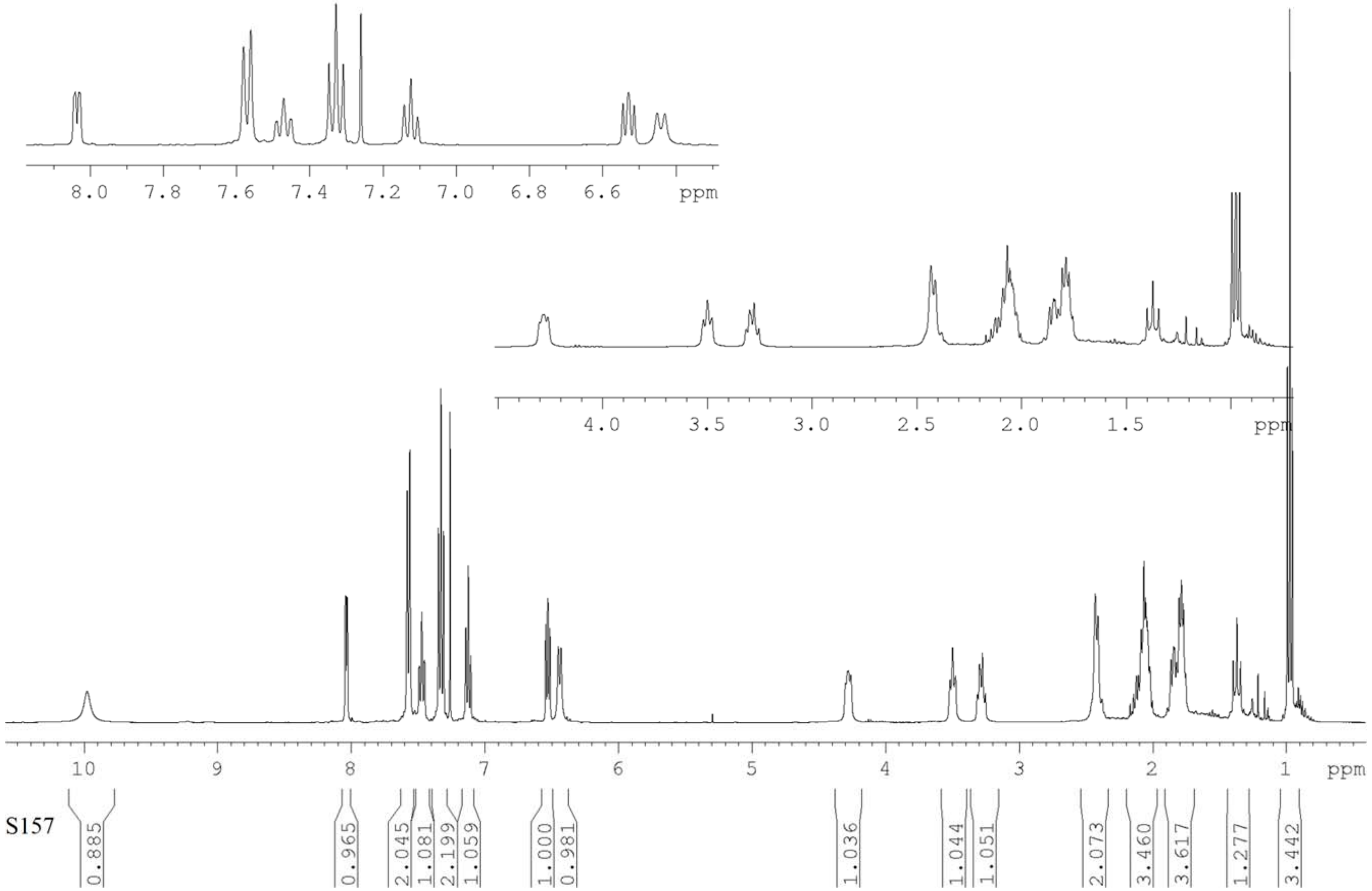

^{13}C NMR (101 MHz, $CDCl_3$) for **(*R*)-*N*-Phenyl-2-(((*S*)-1-(pyridin-2-yl)pyrrolidin-2-yl)methyl)butanamide (5eb)**

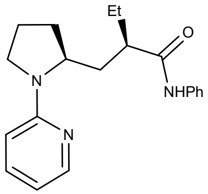

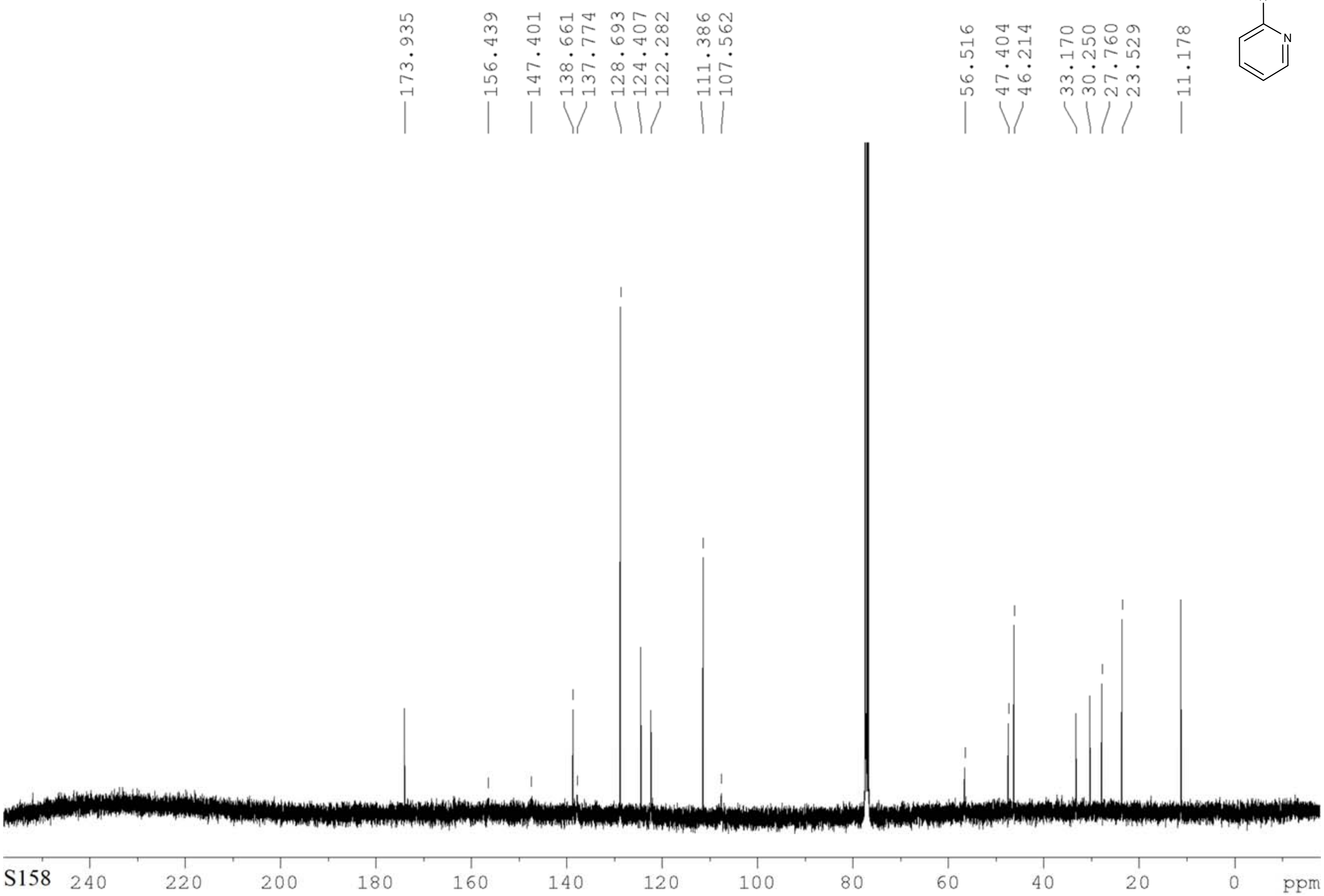

^{1}H NMR (400 MHz, CD_3CN) for ***(S)*-*N*-Phenyl-2-(((*S*)-1-(pyrimidin-2-yl)pyrrolidin-2-yl)methyl)butanamide (5ia)**

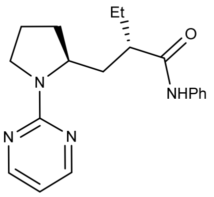

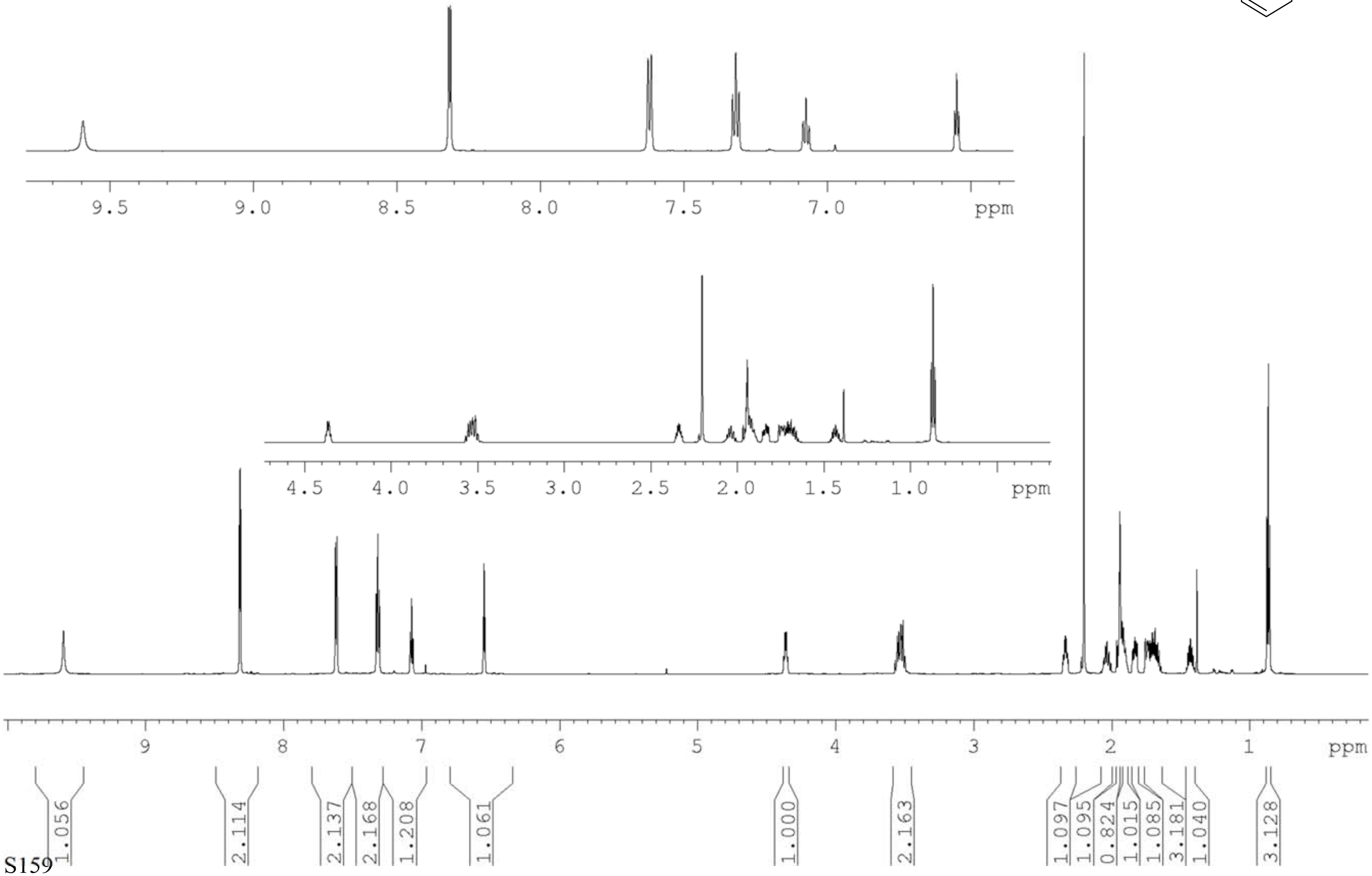

^{13}C NMR (101 MHz, CD_3CN) for ***(S)*-*N*-Phenyl-2-(((*S*)-1-(pyrimidin-2-yl)pyrrolidin-2-yl)methyl)butanamide (5ia)**

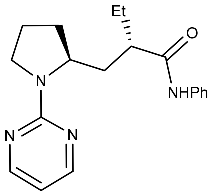

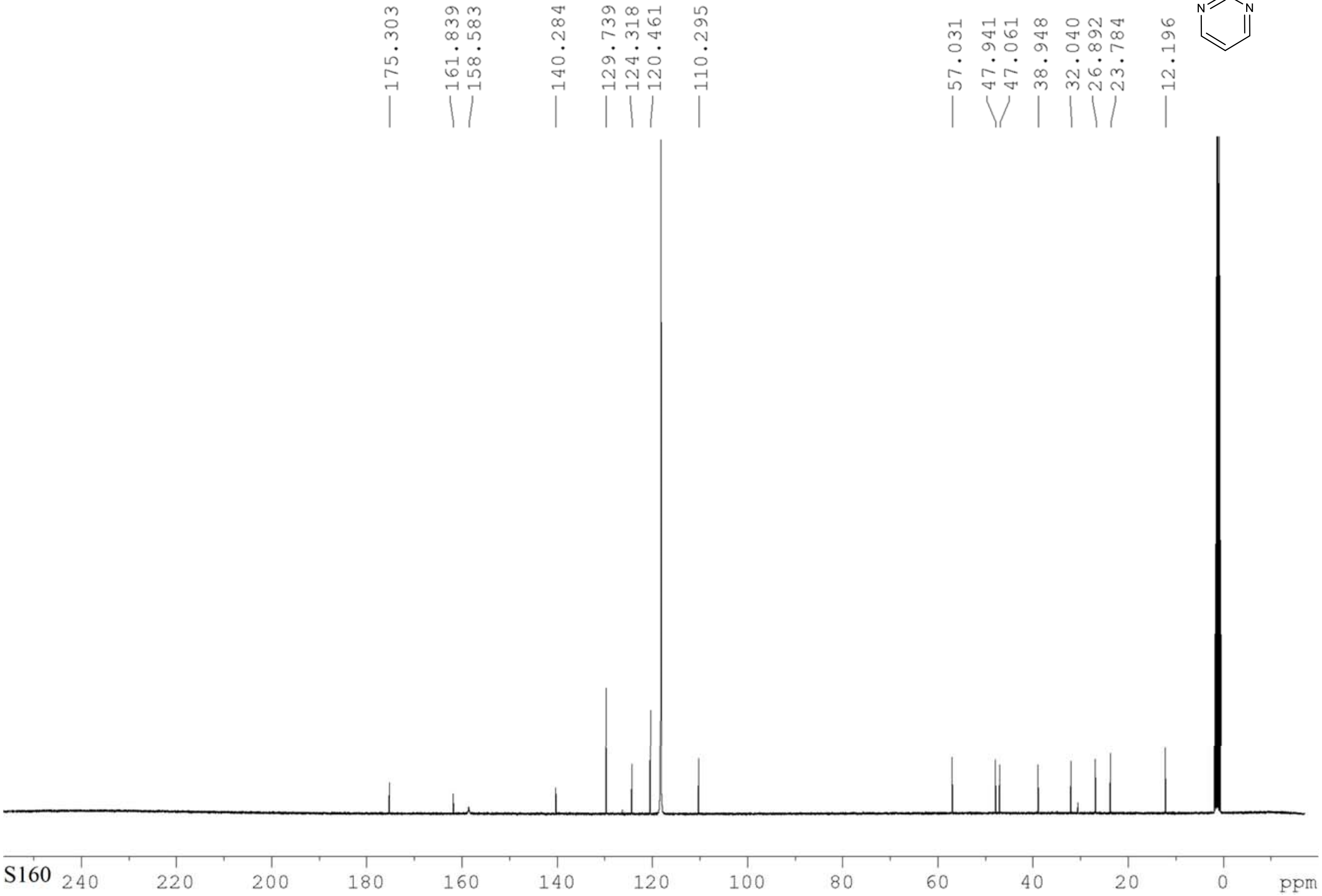

¹H NMR (700 MHz, CD_3CN) for **(*R*)-*N*-Phenyl-2-(((*S*)-1-(pyrimidin-2-yl)pyrrolidin-2-yl)methyl)butanamide (5ib)**

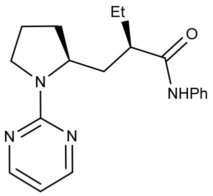

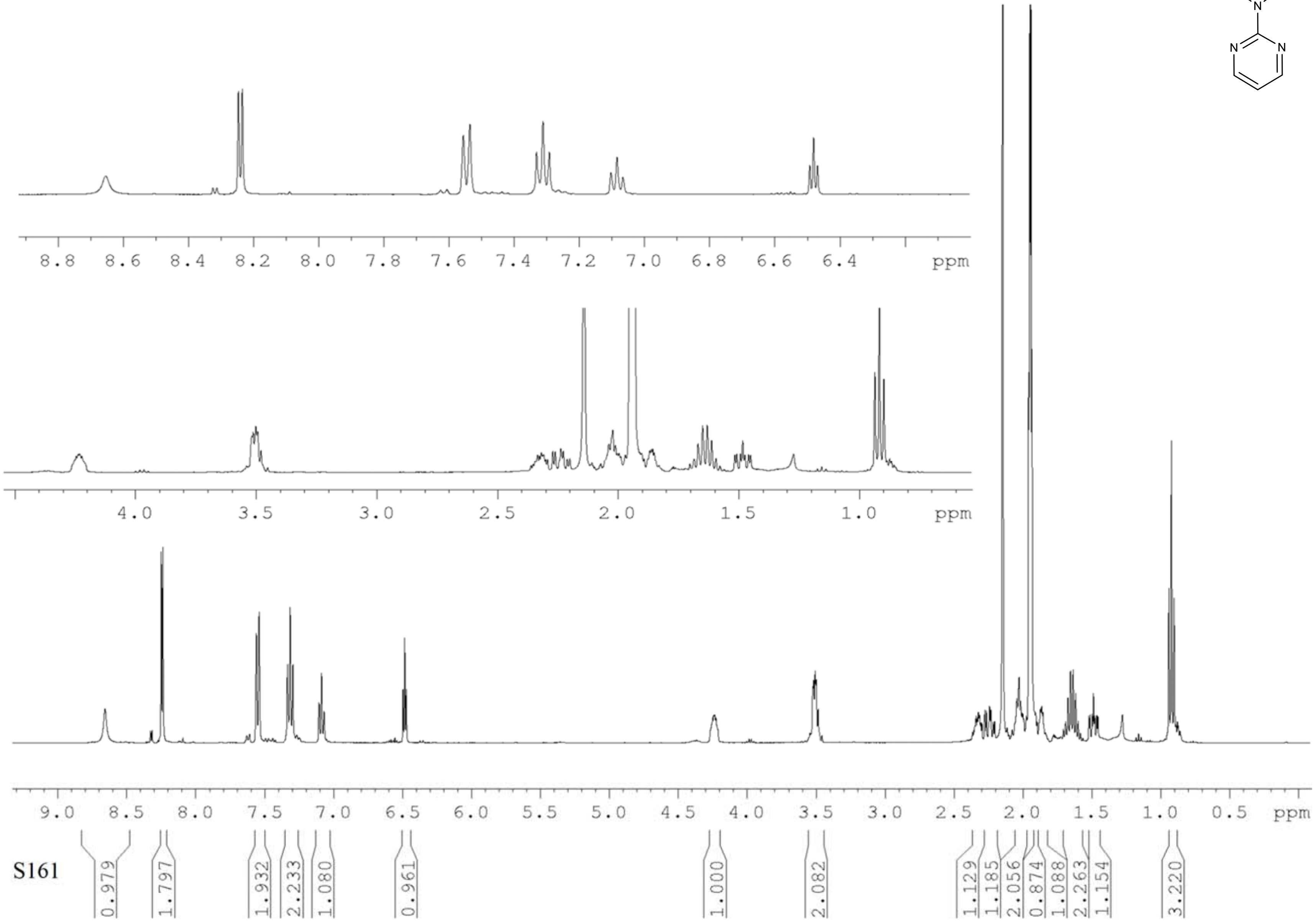

^{13}C NMR (101 MHz, CD_3CN) for **(*R*)-*N*-Phenyl-2-(((*S*)-1-(pyrimidin-2-yl)pyrrolidin-2-yl)methyl)butanamide (5ib)**

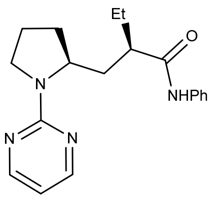

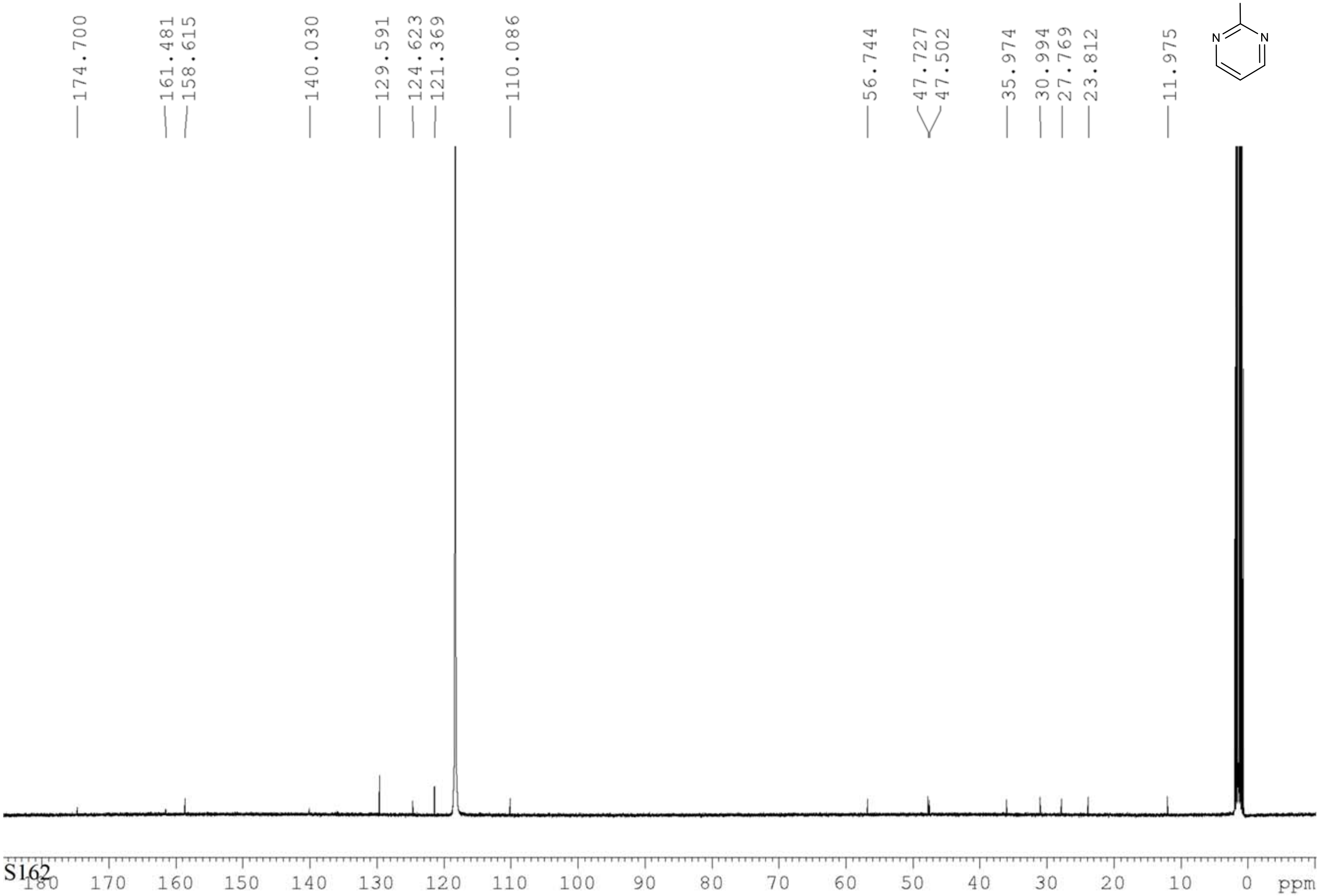

^{1}H NMR (400 MHz, $CDCl_3$) for **(*S*)-*N*-Phenyl-2-(((*S*)-1-(pyridin-2-yl)piperidin-2-yl)methyl)butanamide (5ga)**

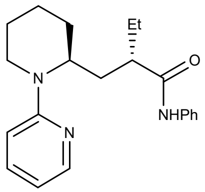

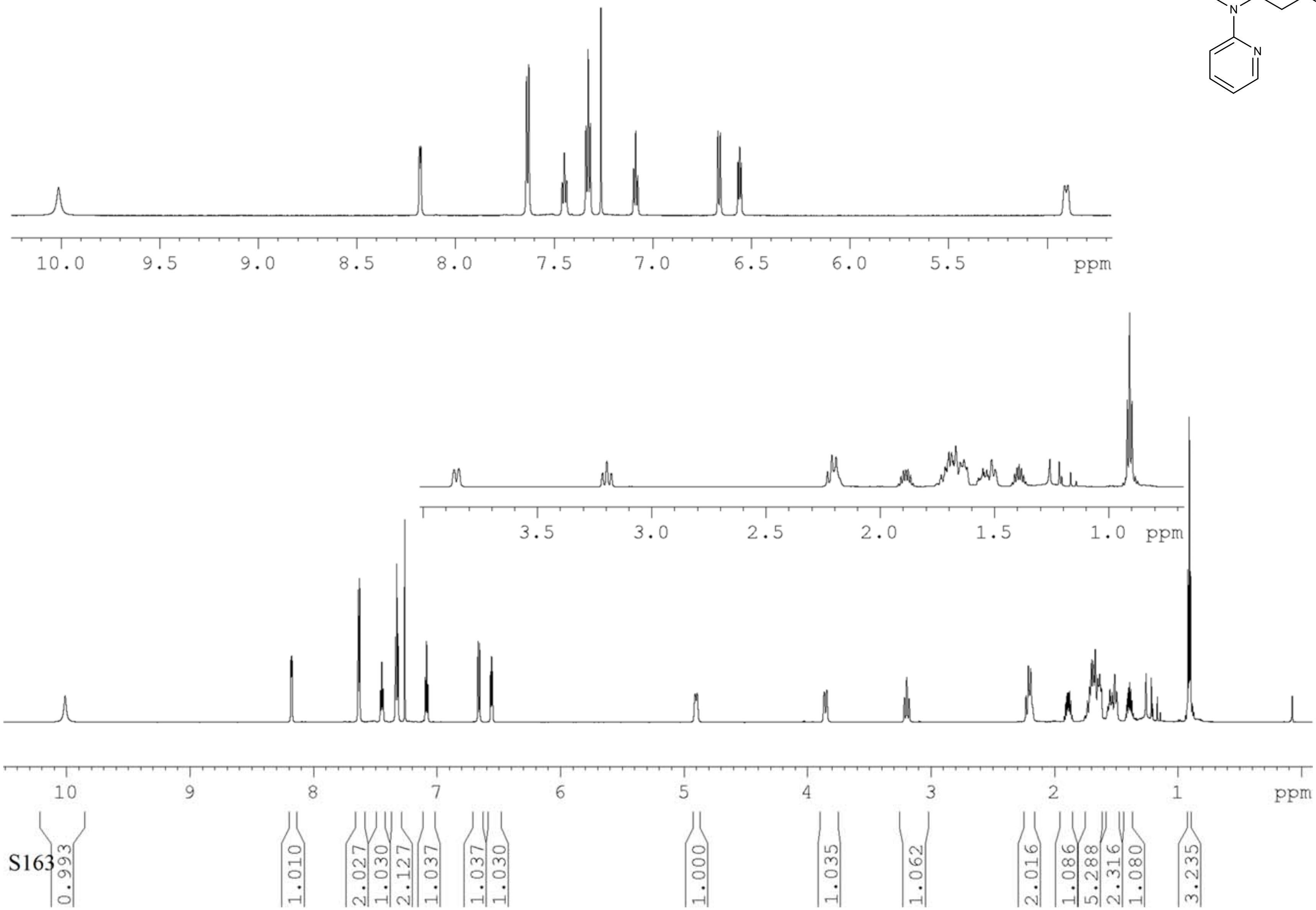

^{13}C NMR (101 MHz, $CDCl_3$) for **(*S*)-*N*-Phenyl-2-(((*S*)-1-(pyridin-2-yl)piperidin-2-yl)methyl)butanamide (5ga)**

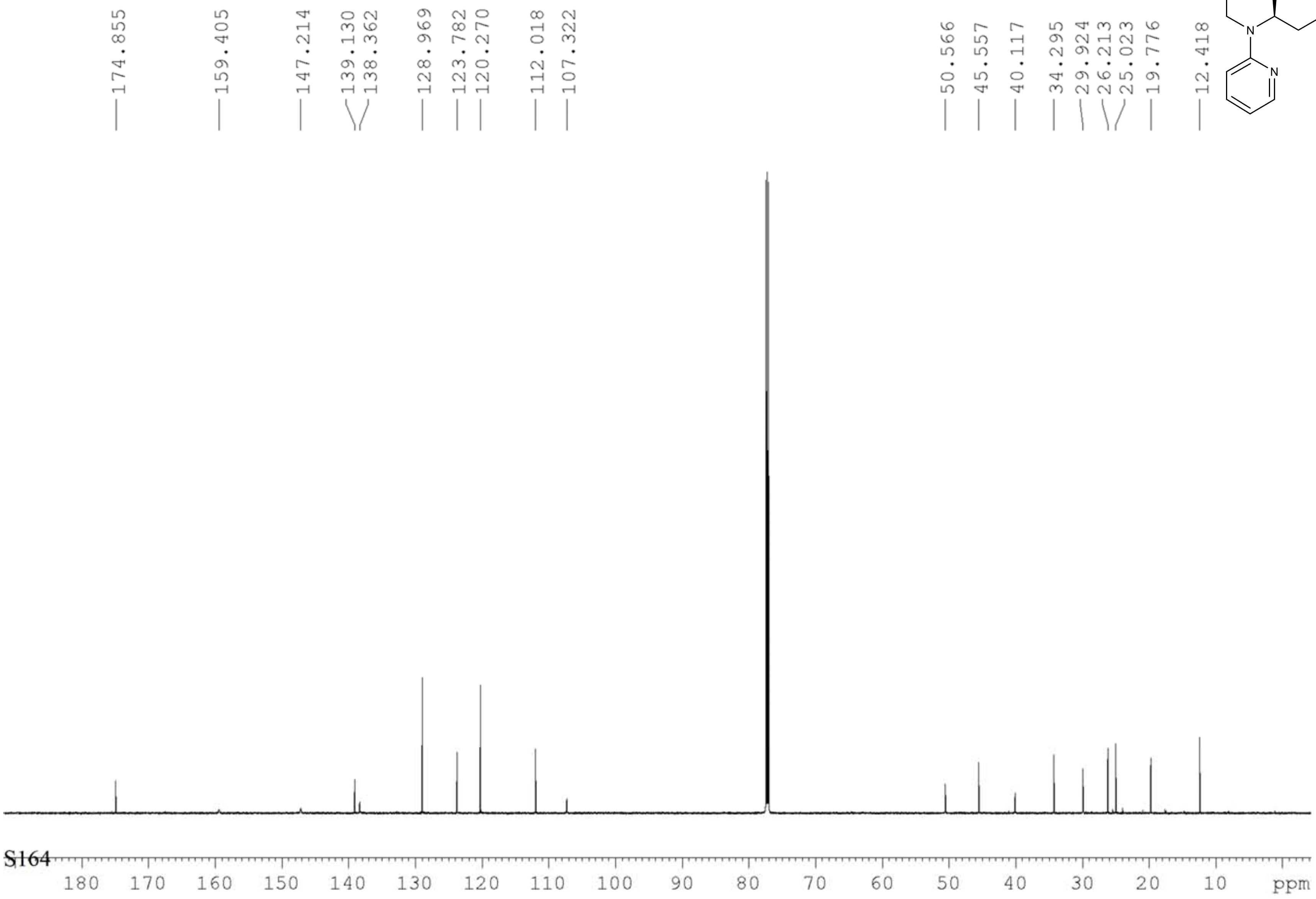

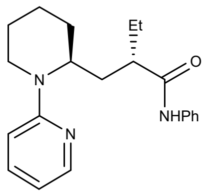

^{1}H NMR (700 MHz, $CDCl_3$) for ***(R)*-*N*-Phenyl-2-(((*S*)-1-(pyridin-2-yl)piperidin-2-yl)methyl)butanamide (5gb)**

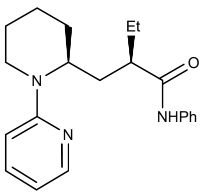

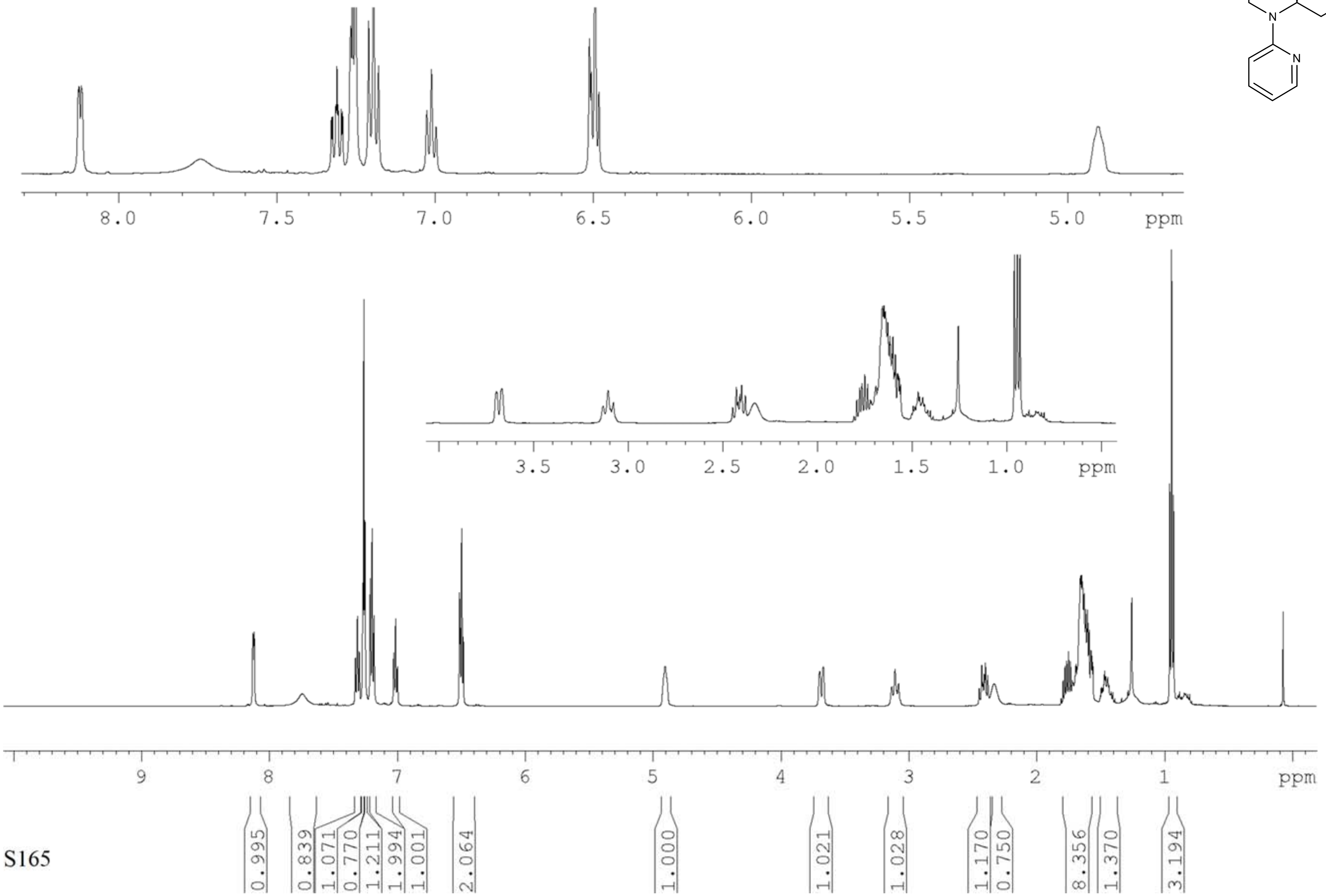

^{13}C NMR (176 MHz, $CDCl_3$) for **(*R*)-*N*-Phenyl-2-(((*S*)-1-(pyridin-2-yl)piperidin-2-yl)methyl)butanamide (5gb)**

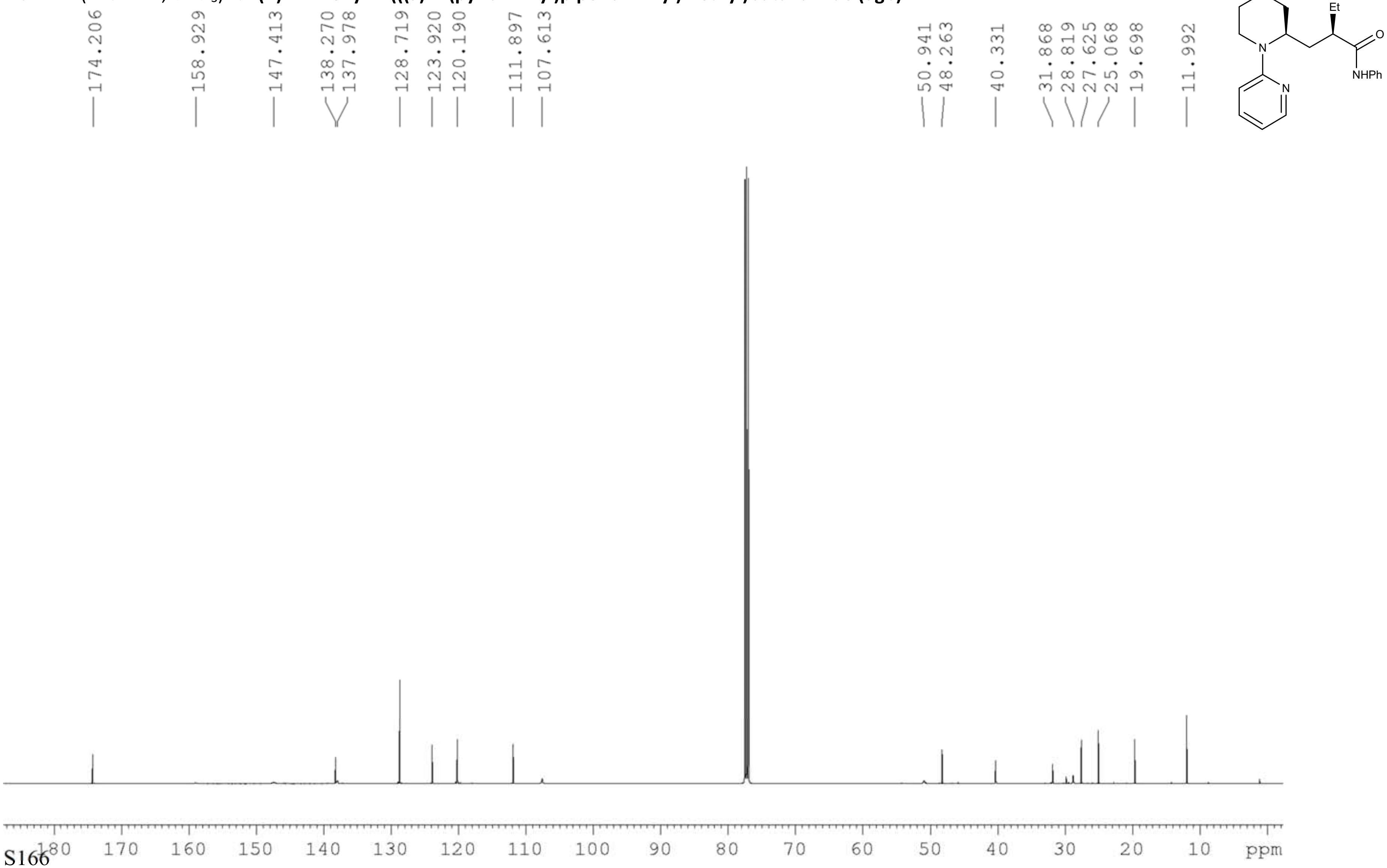

^{1}H NMR (500 MHz, $CDCl_3$) for **(*S*)-2-Benzyl-*N*-phenyl-3-((*S*)-1-(pyridin-2-yl)piperidin-2-yl)propanamide (5ha)**

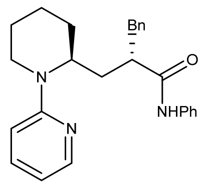

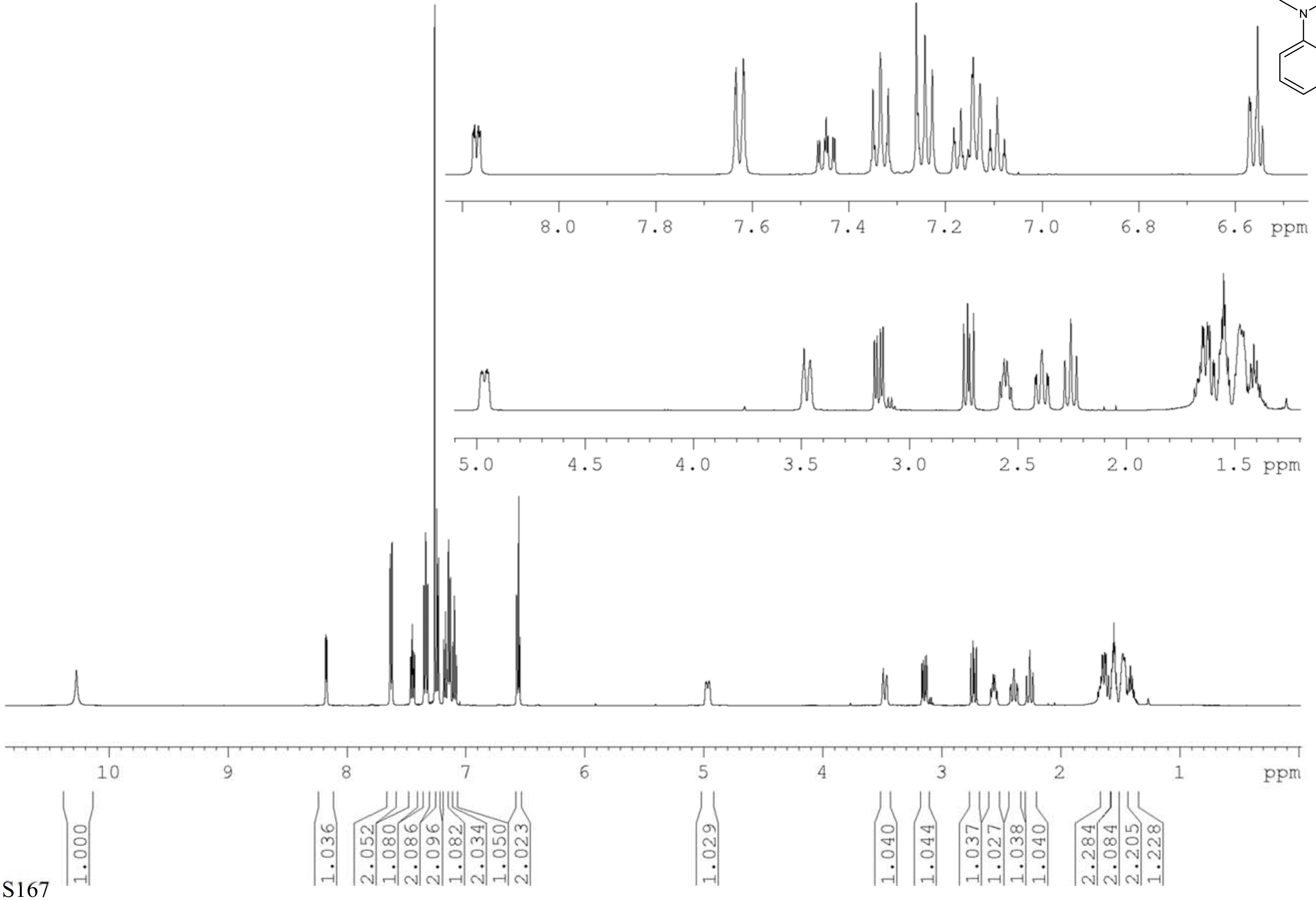

^{13}C NMR (101 MHz, $CDCl_3$) for **(*S*)-2-Benzyl-*N*-phenyl-3-((*S*)-1-(pyridin-2-yl)piperidin-2-yl)propanamide (5ha)**

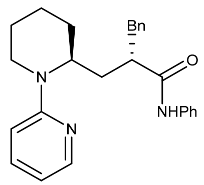

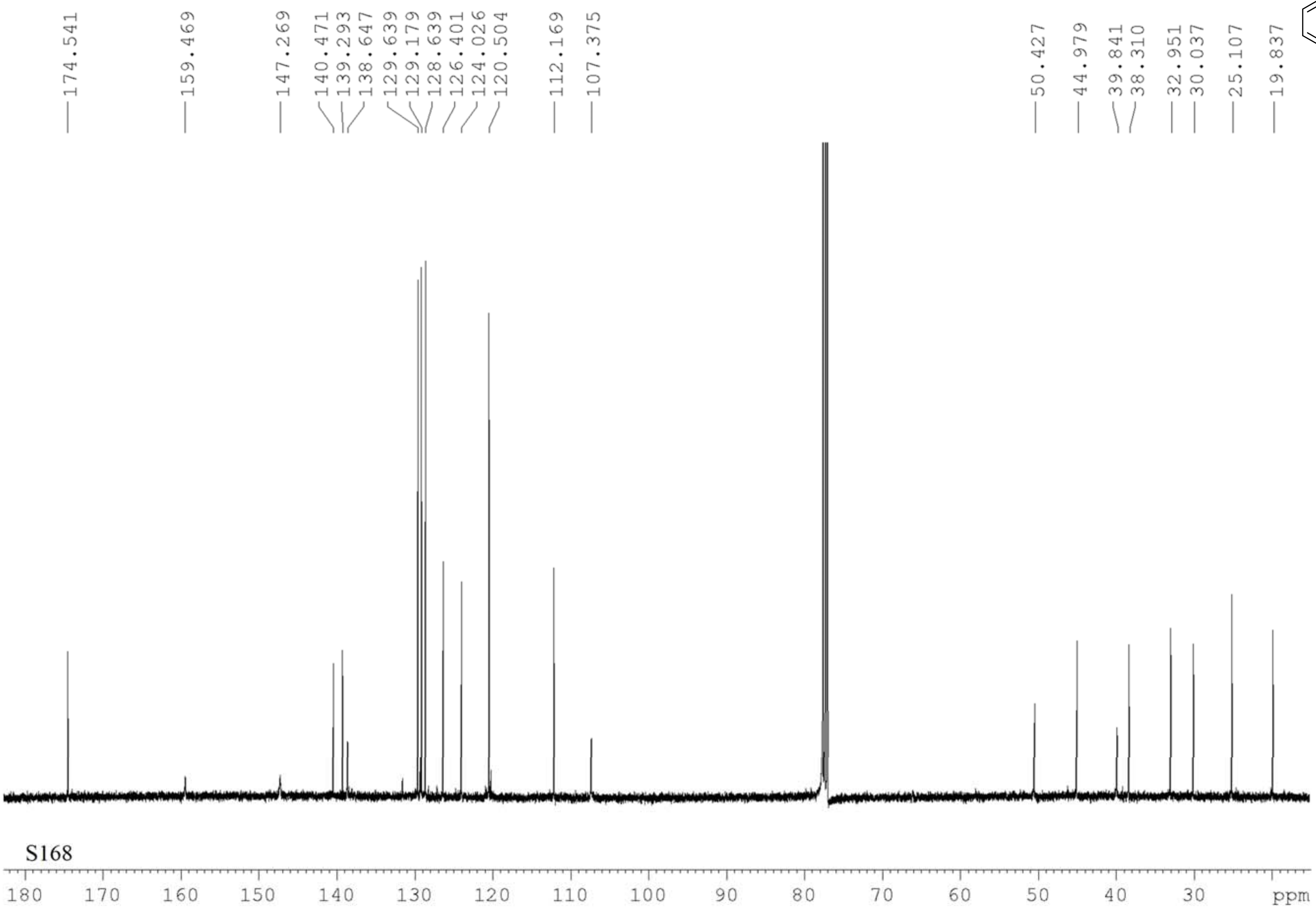

¹H NMR (500 MHz, $CDCl_3$) for **(*R*)-2-Benzyl-*N*-phenyl-3-((*S*)-1-(pyridin-2-yl)piperidin-2-yl)propanamide (5hb)**

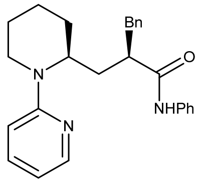

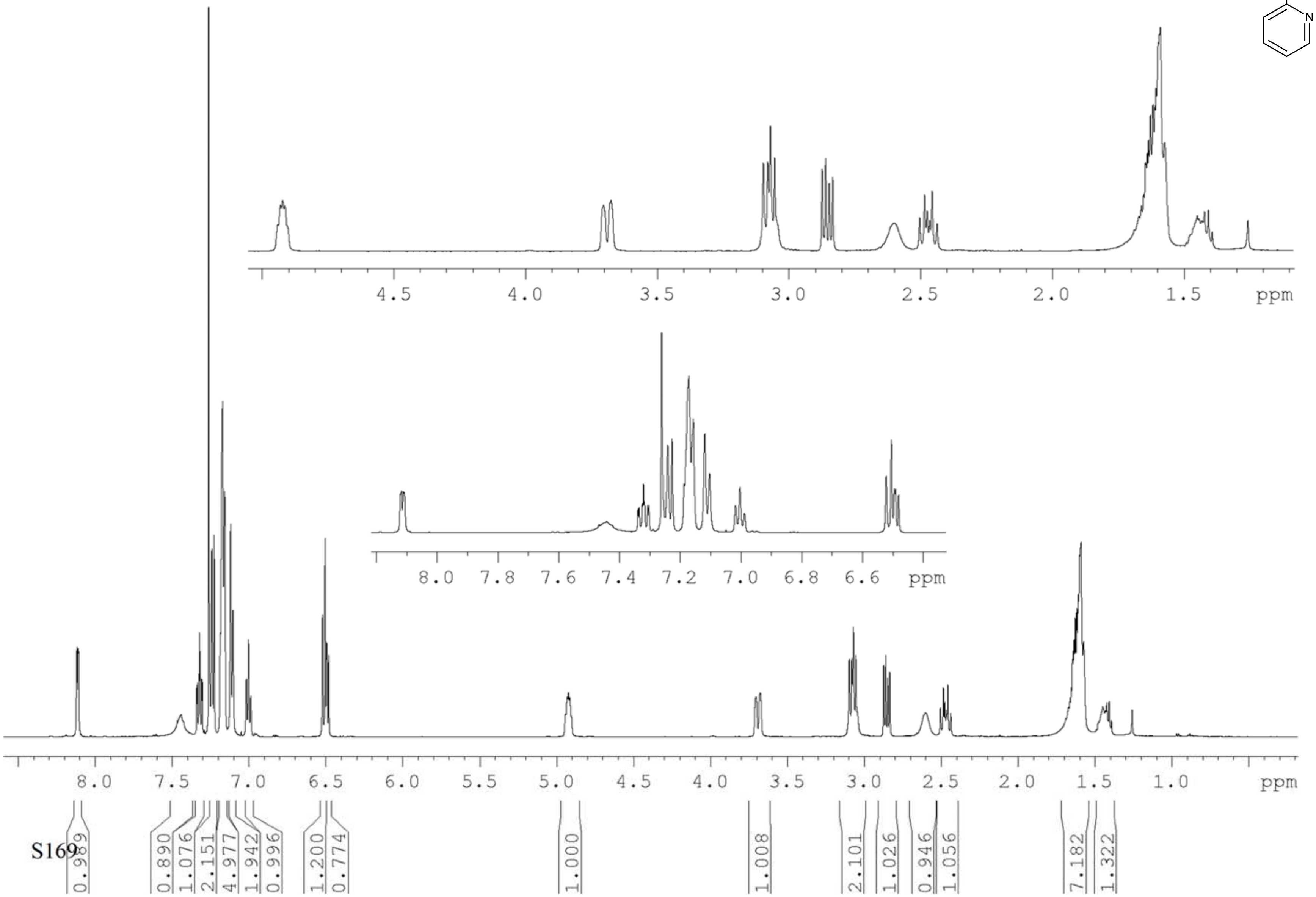

^{13}C NMR (101 MHz, CDCl$_3$) for **(*R*)-2-Benzyl-*N*-phenyl-3-((*S*)-1-(pyridin-2-yl)piperidin-2-yl)propanamide (5hb)**

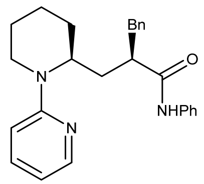

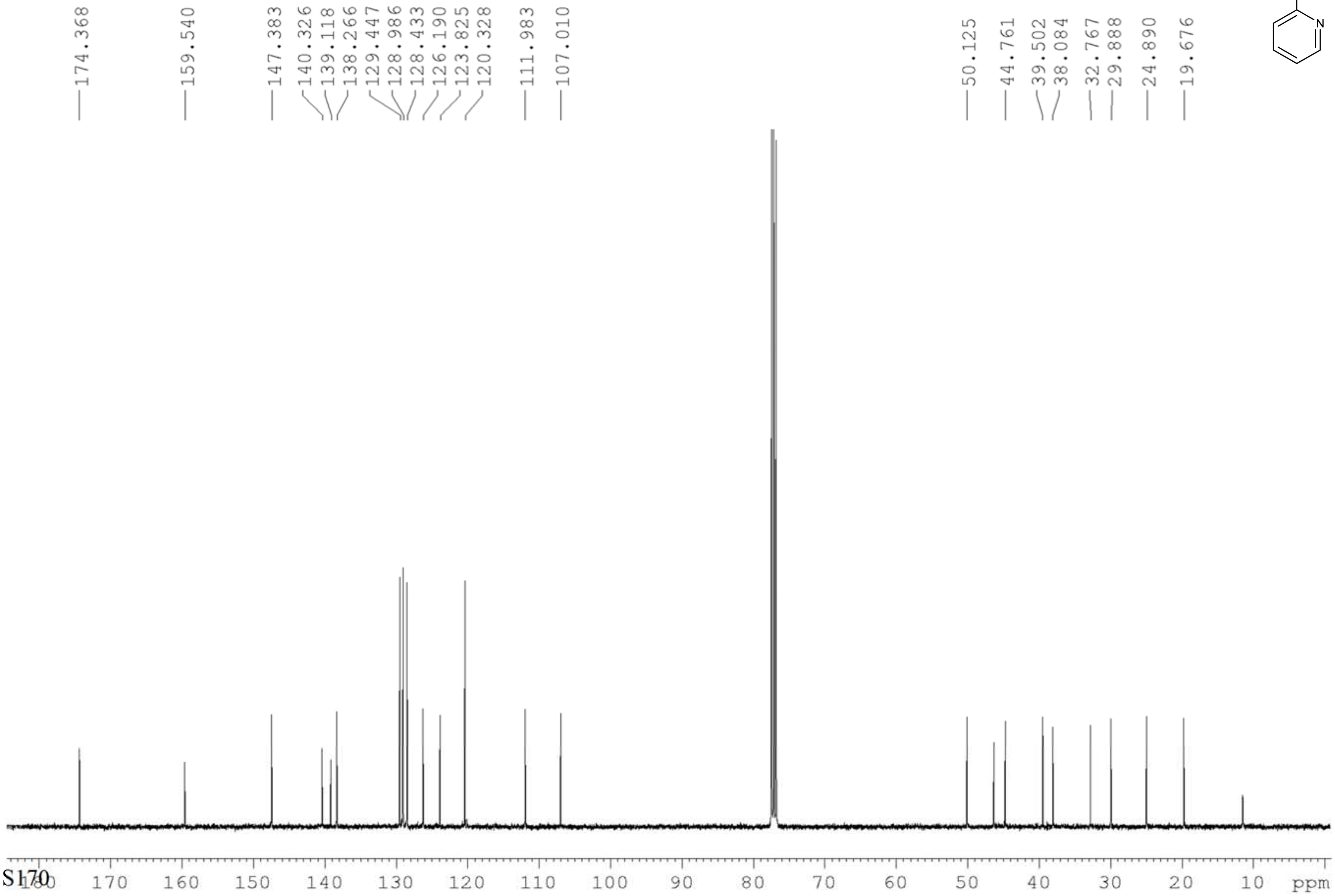

^{1}**H NMR** (400 MHz, $CDCl_3$) for **(*S*)-*N*-Phenyl-2-(((S)-1-(pyridin-2-yl)azepan-2-yl)methyl)butanamide (5ia)**

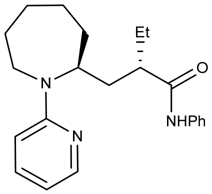

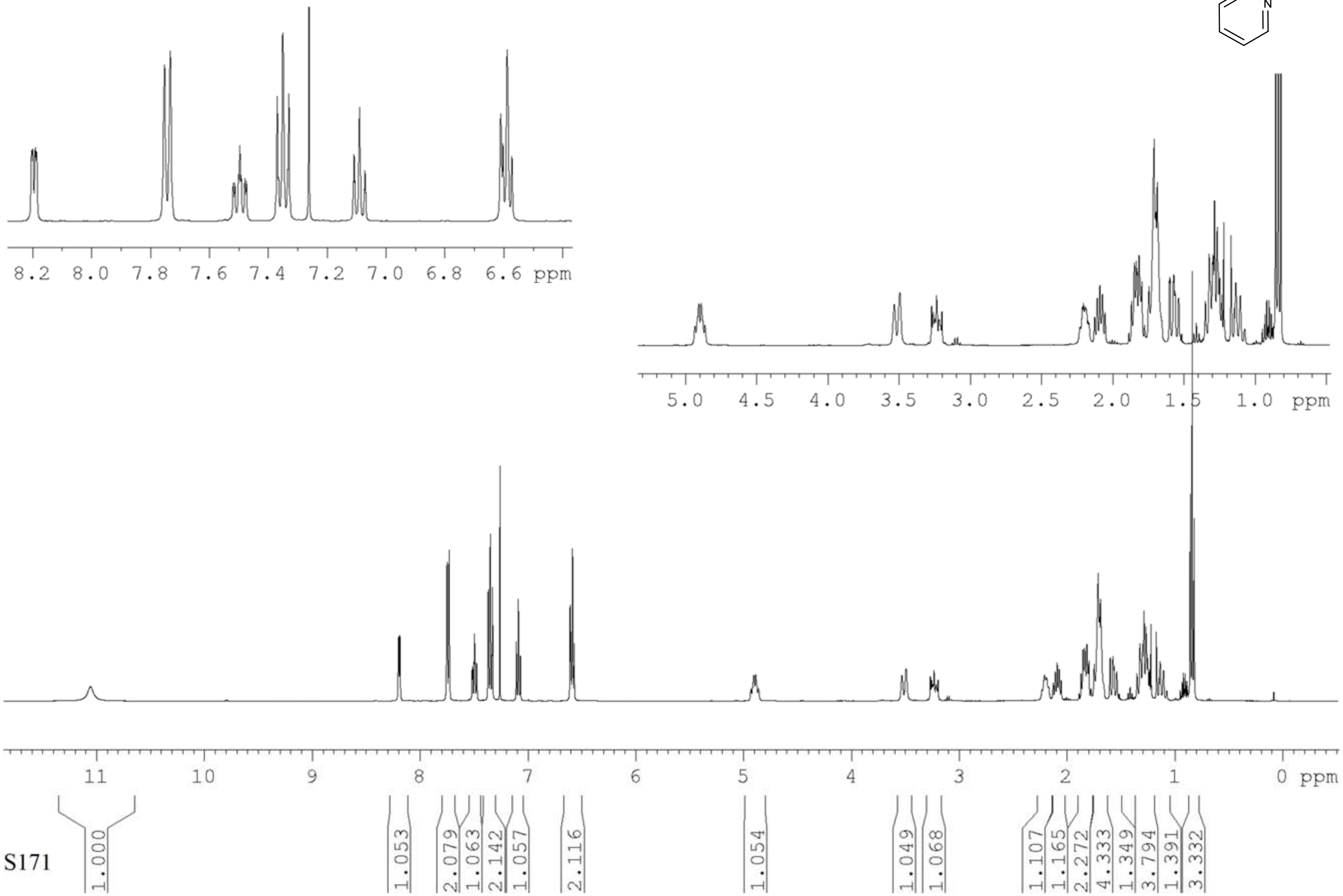

^{13}**C NMR** (101 MHz, $CDCl_3$) for **(*S*)-*N*-Phenyl-2-(((S)-1-(pyridin-2-yl)azepan-2-yl)methyl)butanamide (5ia)**

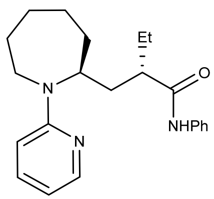

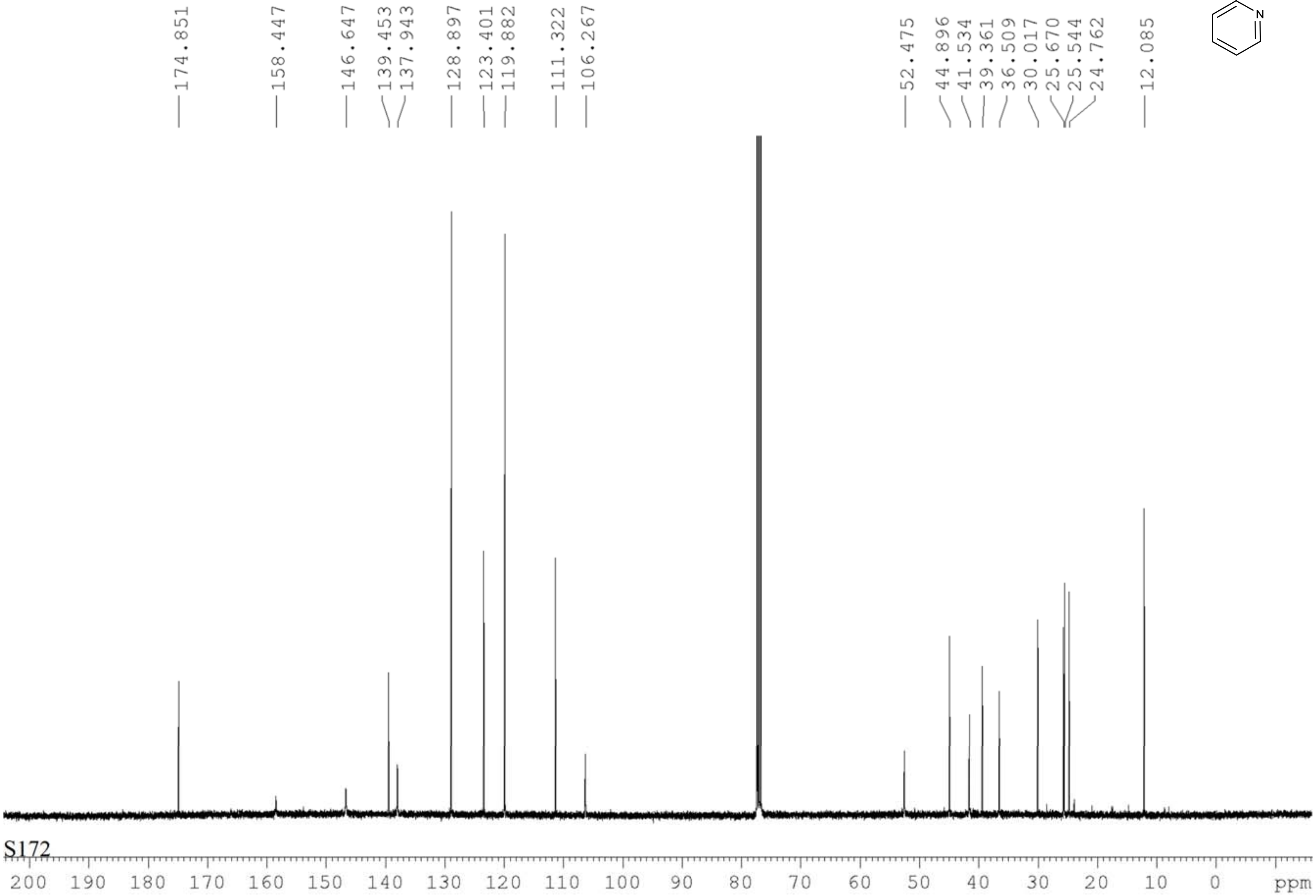

^{1}H NMR (400 MHz, $CDCl_3$) for **(*R*)-*N*-Phenyl-2-(((S)-1-(pyridin-2-yl)azepan-2-yl)methyl)butanamide (5ib)**

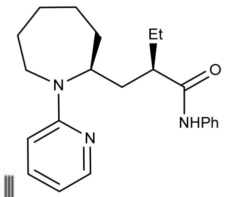

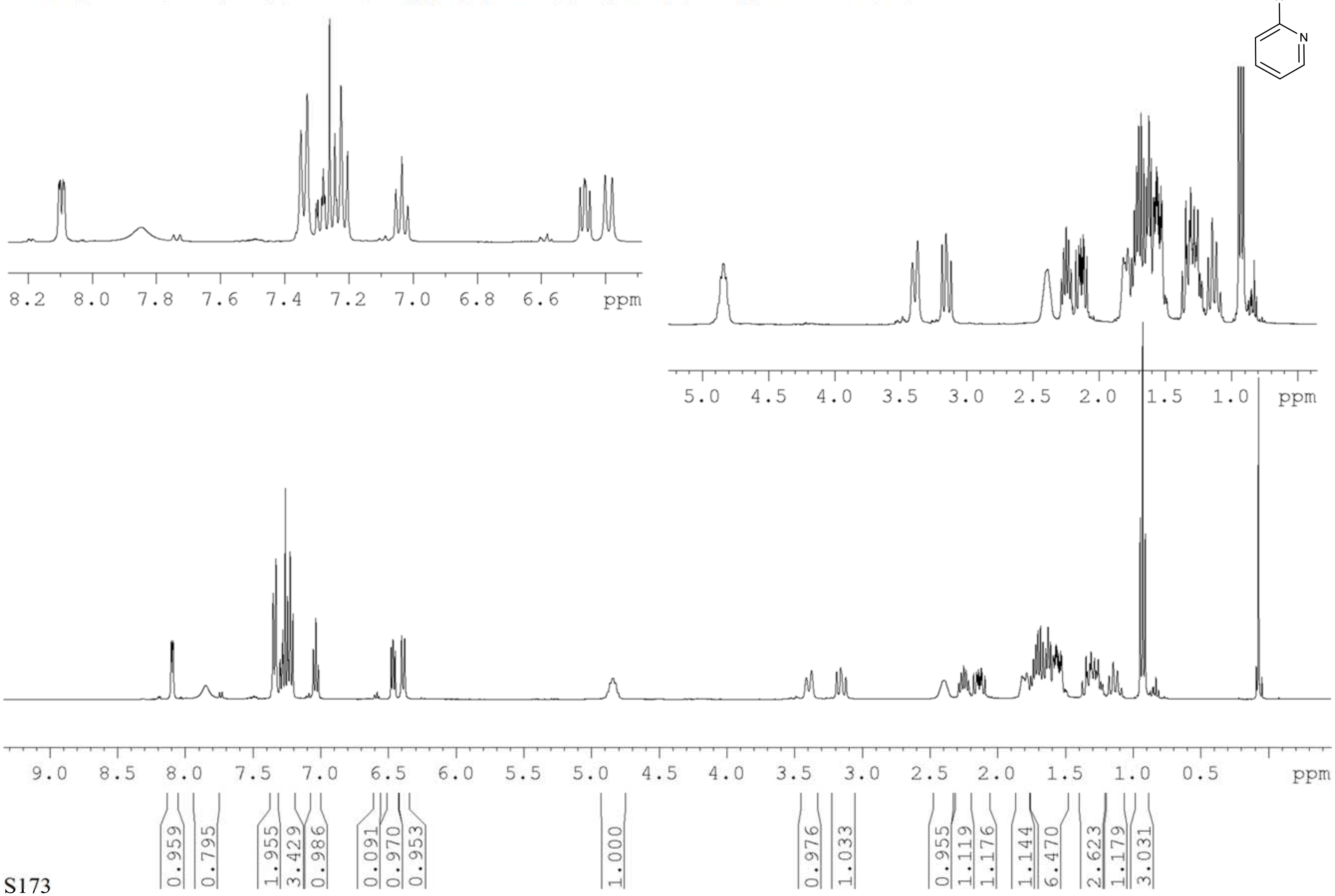

^{13}C NMR (101 MHz, $CDCl_3$) for **(*R*)-*N*-Phenyl-2-(((S)-1-(pyridin-2-yl)azepan-2-yl)methyl)butanamide (5ib)**

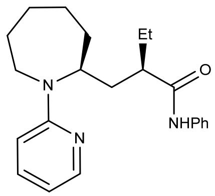

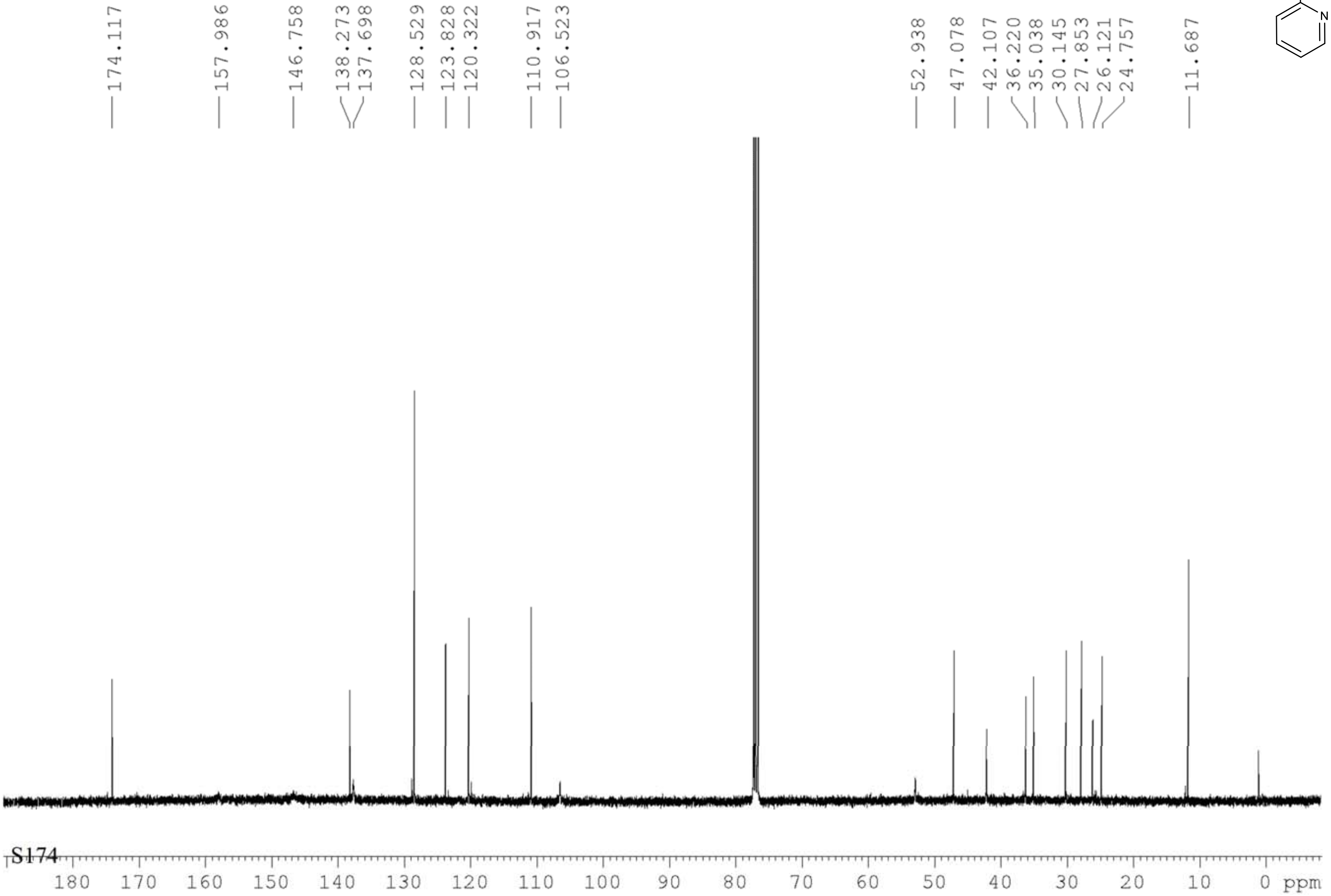

^{1}H NMR (400 MHz, $CDCl_3$) for **(*S*)-2-Benzyl-*N*-phenyl-3-((*S*)-1-(pyridin-2-yl)azepan-2-yl)propanamide (5ja)**

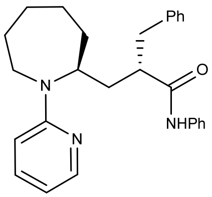

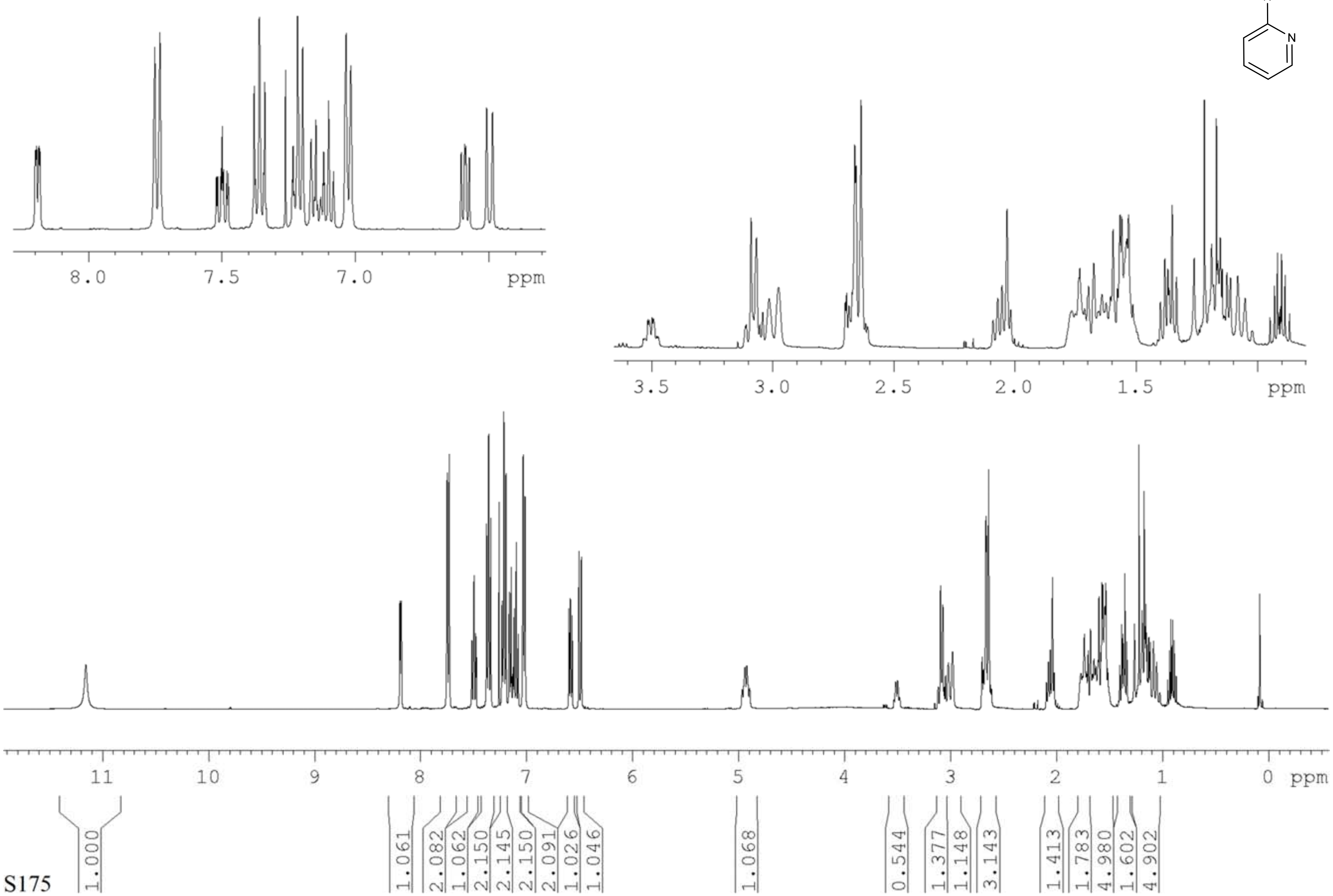

^{13}C NMR (101 MHz, $CDCl_3$) for **(*S*)-2-Benzyl-*N*-phenyl-3-((*S*)-1-(pyridin-2-yl)azepan-2-yl)propanamide (5ja)**

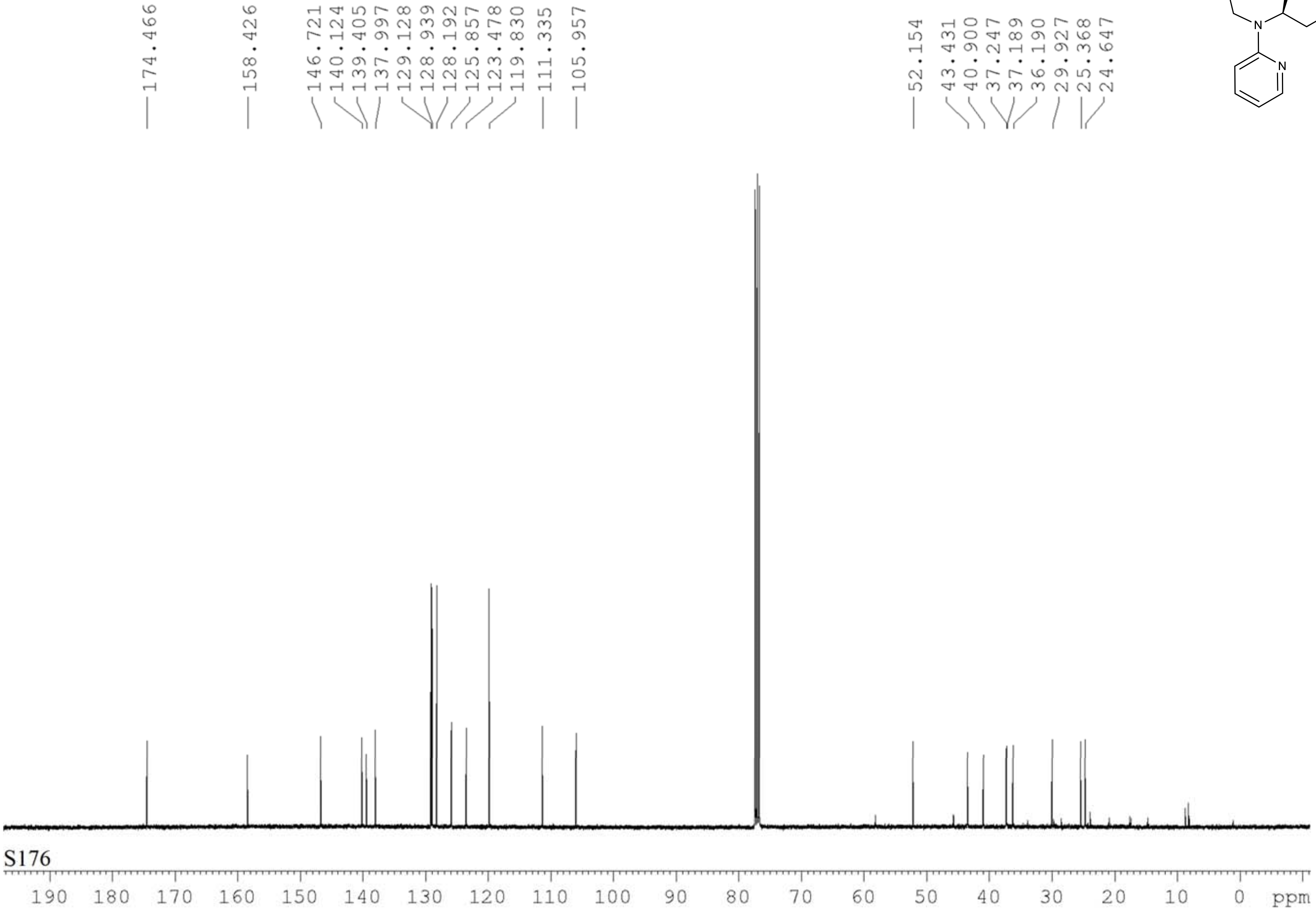

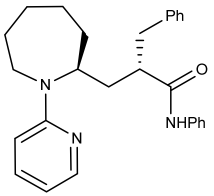

^{1}H NMR (400 MHz, $CDCl_3$) for **(*R*)-2-benzyl-*N*-phenyl-3-((*S*)-1-(pyridin-2-yl)azepan-2-yl)propanamide (5jb)**

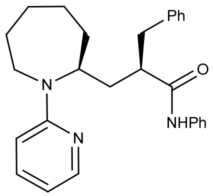

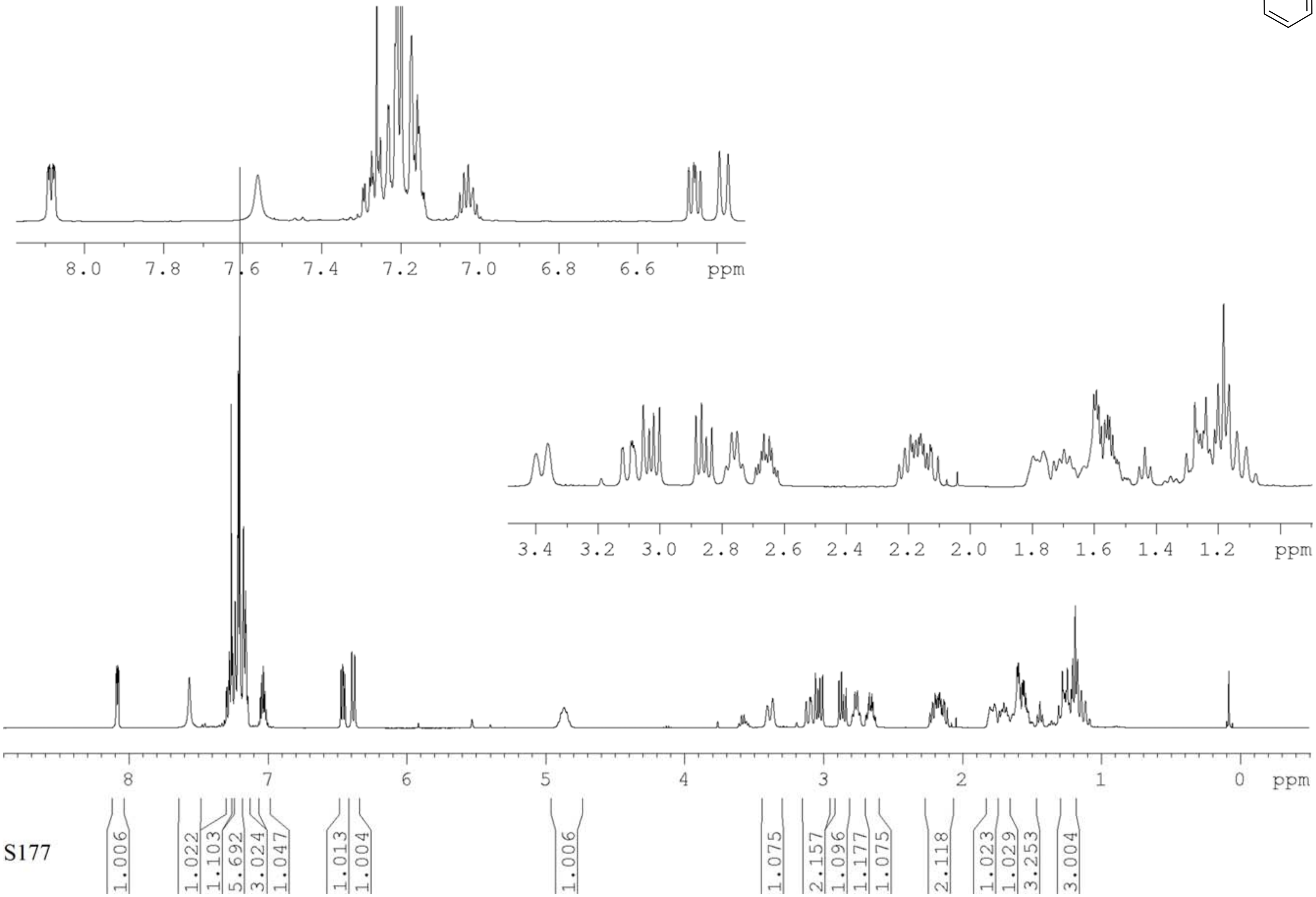

^{13}**C NMR** (101 MHz, $CDCl_3$) for **(*R*)-2-benzyl-*N*-phenyl-3-((*S*)-1-(pyridin-2-yl)azepan-2-yl)propanamide (5jb)**

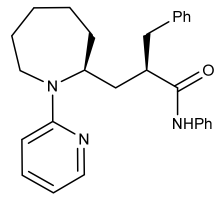

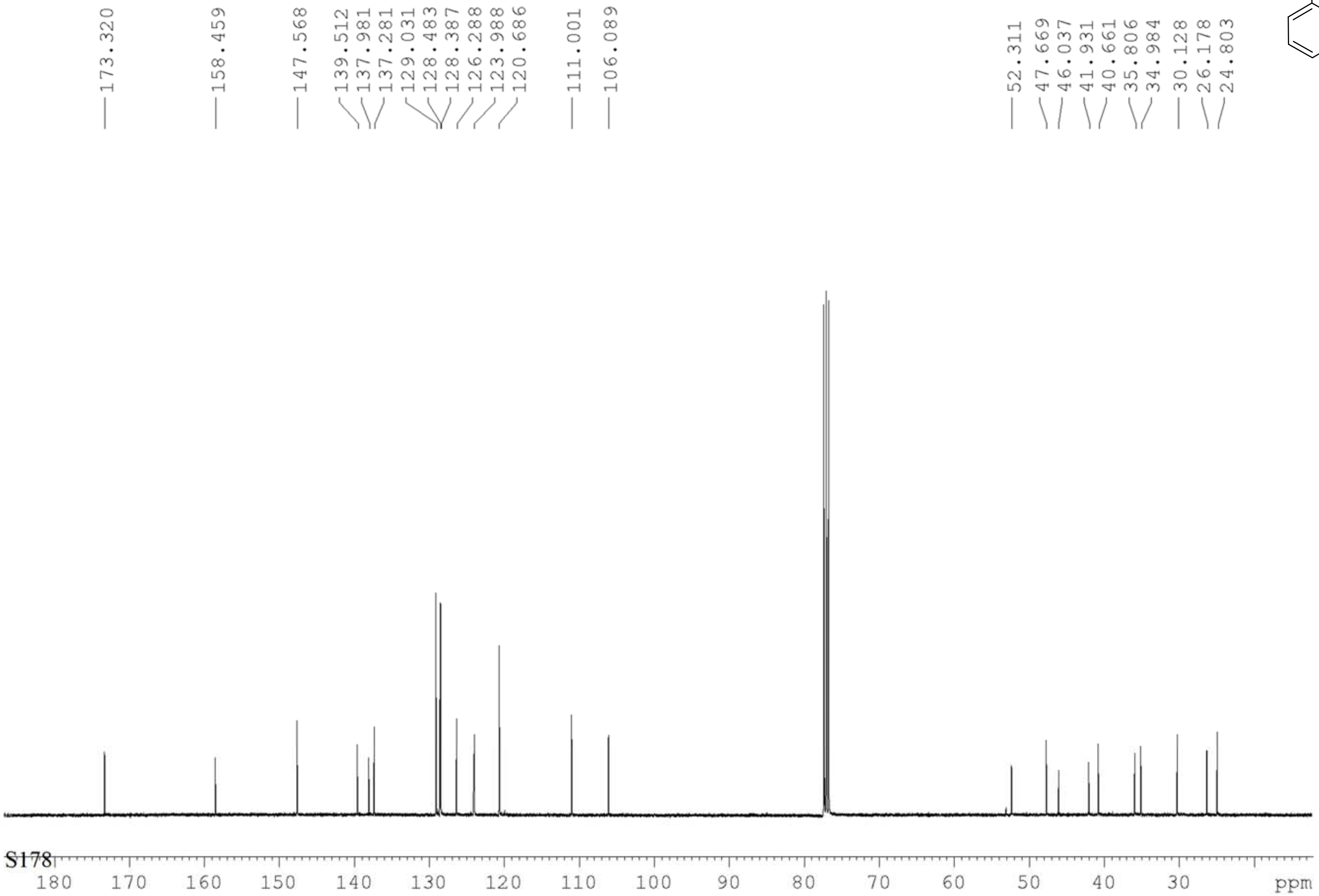

^{1}H NMR (400 MHz, $CDCl_3$) for ***(R)*-3-Methyl-*N*-phenyl-2-(((*S*)-1-(pyridin-2-yl)azepan-2-yl)methyl)butanamide (5ka)**

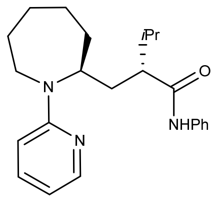

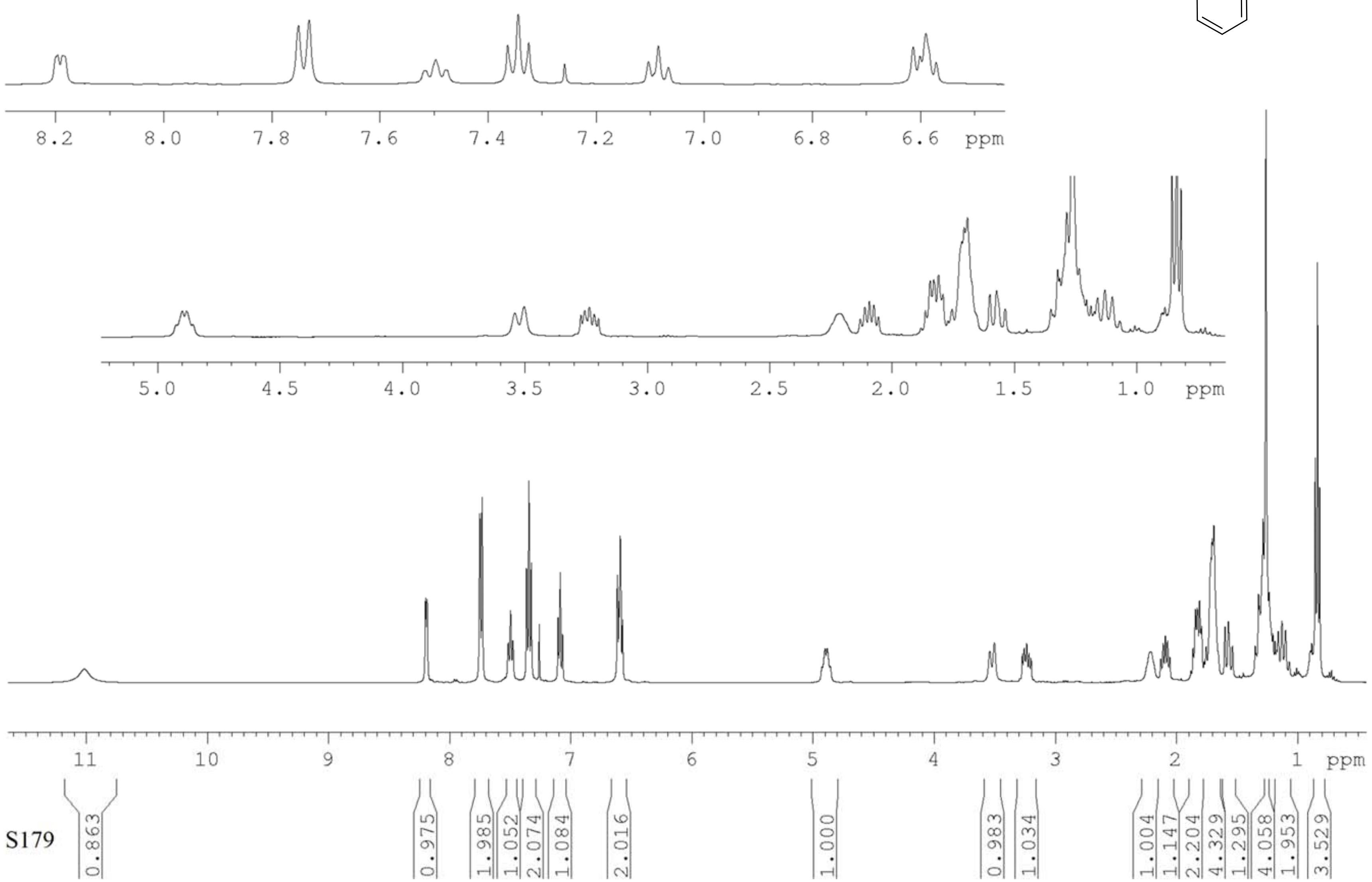

^{13}C NMR (101 MHz, $CDCl_3$) for **(*R*)-3-Methyl-*N*-phenyl-2-(((*S*)-1-(pyridin-2-yl)azepan-2-yl)methyl)butanamide (5ka)**

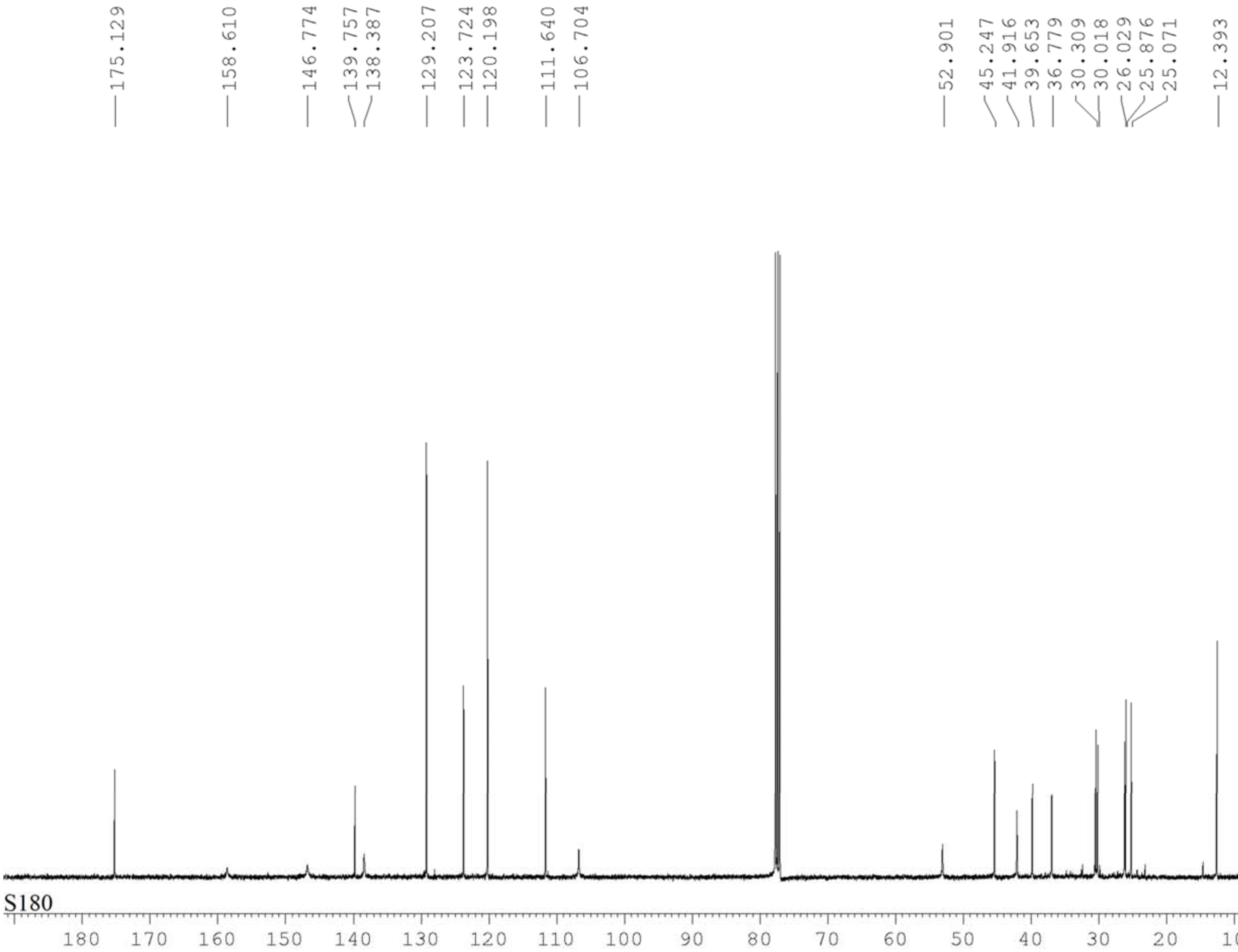

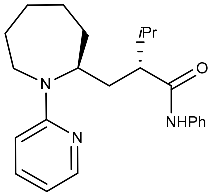

^{1}H NMR (400 MHz, $CDCl_3$) for **(*S*)-3-Methyl-*N*-phenyl-2-(((*S*)-1-(pyridin-2-yl)azepan-2-yl)methyl)butanamide (5kb)**

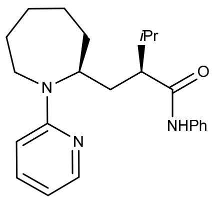

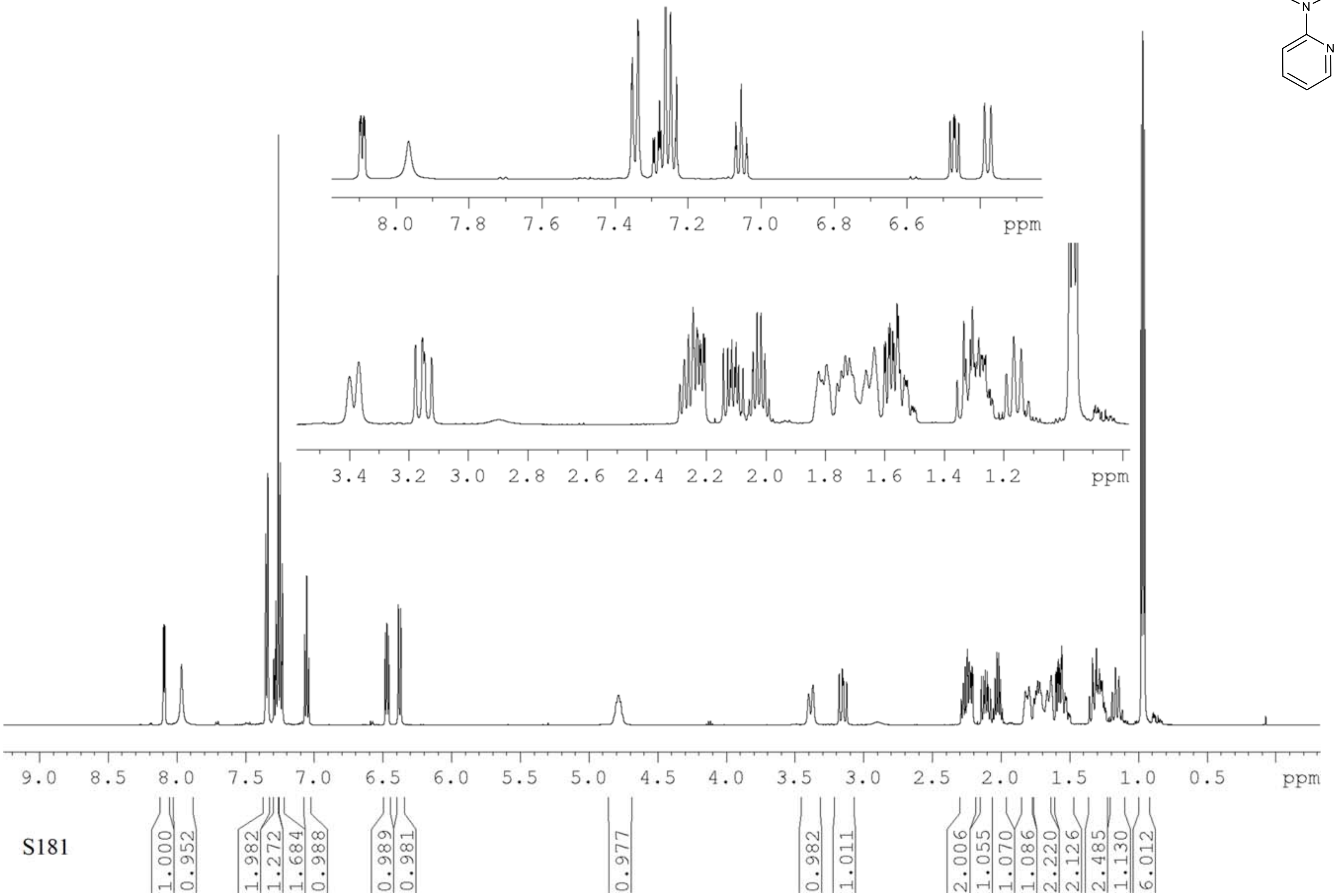

^{13}C NMR (101 MHz, $CDCl_3$) for **(*S*)-3-Methyl-*N*-phenyl-2-(((*S*)-1-(pyridin-2-yl)azepan-2-yl)methyl)butanamide (5kb)**

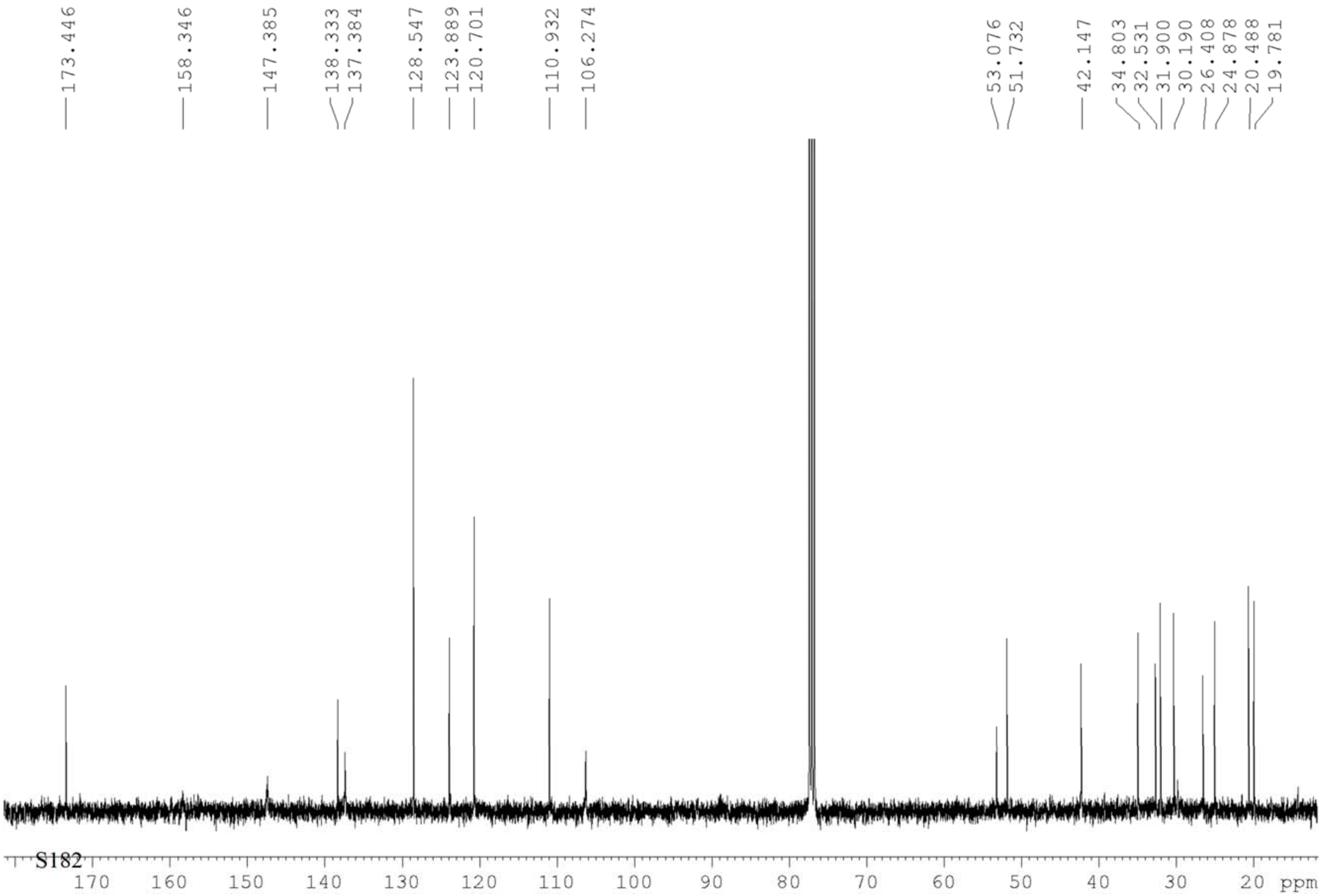

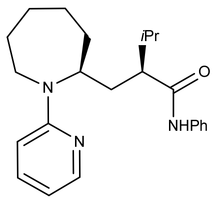

^{1}H NMR (700 MHz, $CDCl_3$) for **(*S*)-*N*-Phenyl-3-(1-(pyridin-2-yl)azepan-2-yl)propenamide (5m)**

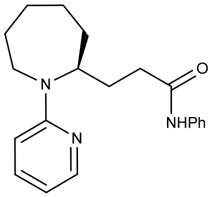

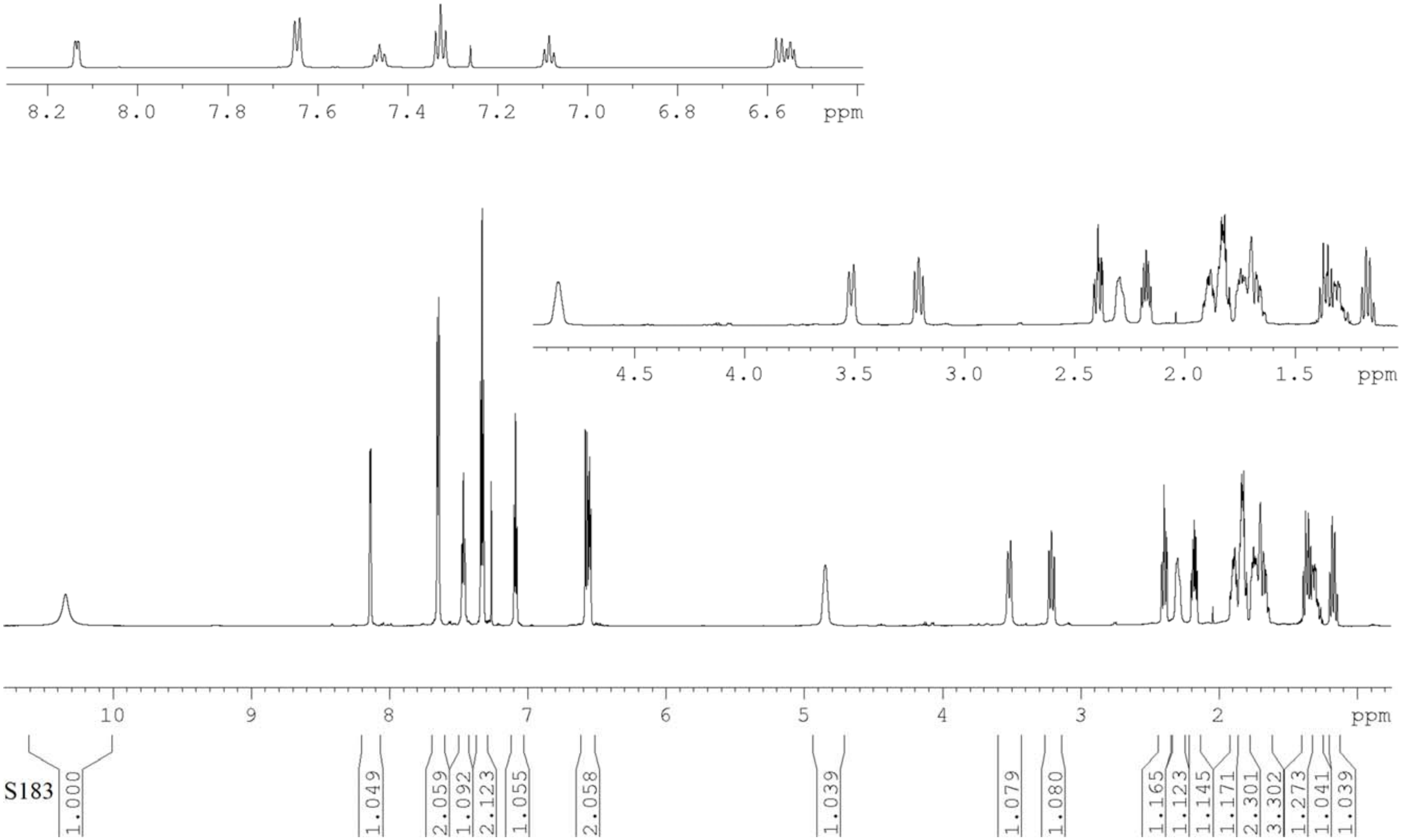

^{13}C NMR (176 MHz, $CDCl_3$) for **(*S*)-*N*-Phenyl-3-(1-(pyridin-2-yl)azepan-2-yl)propenamide (5m)**

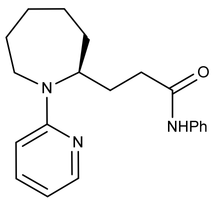

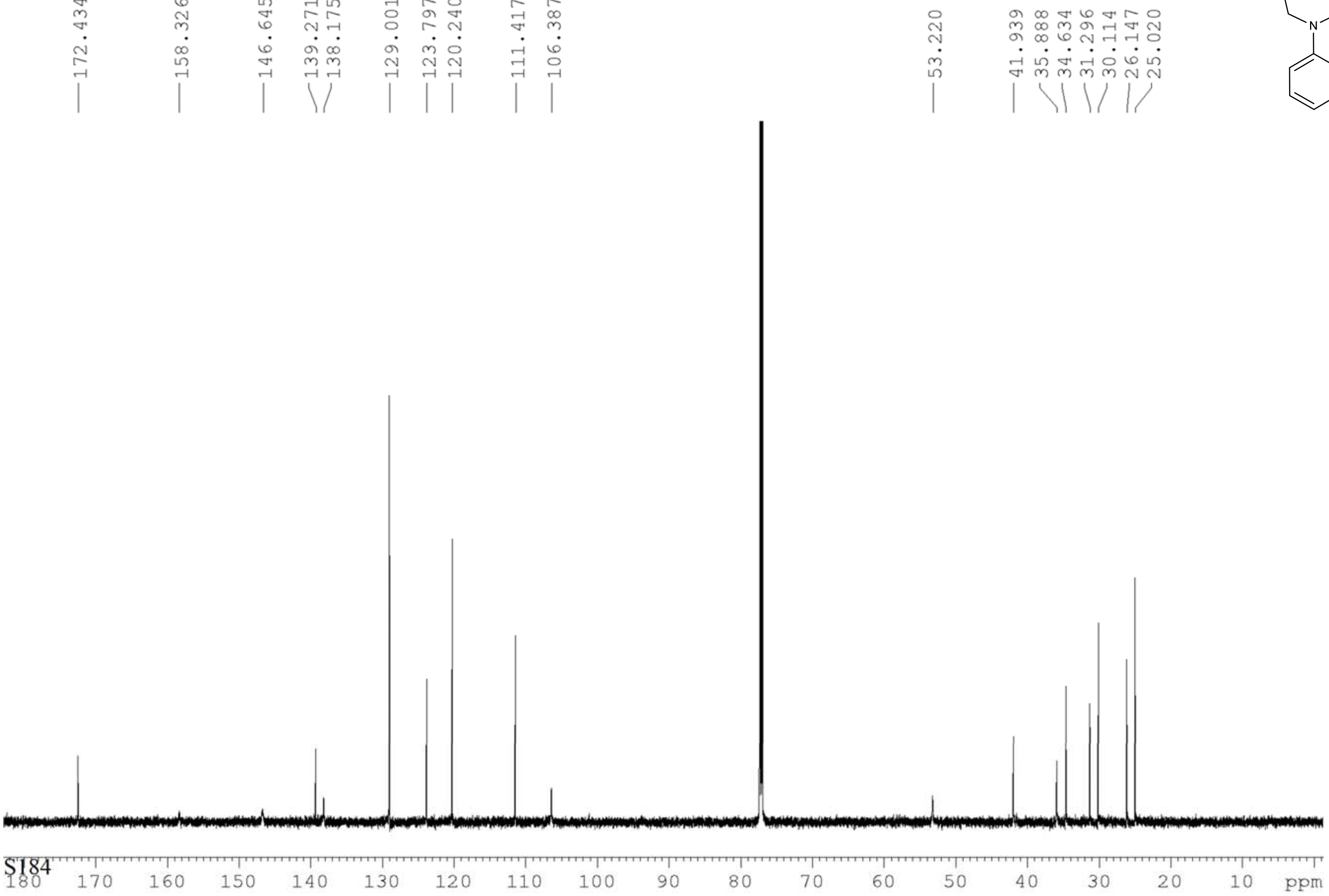

^{1}H NMR (500 MHz, $CDCl_3$) for **methyl (*R*)-4-oxo-4-(phenylamino)-3-(((*S*)-1-(pyridin-2-yl)azepan-2-yl)methyl)butanoate (5lb)**

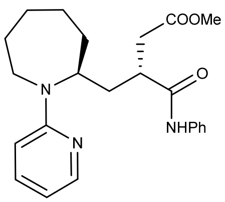

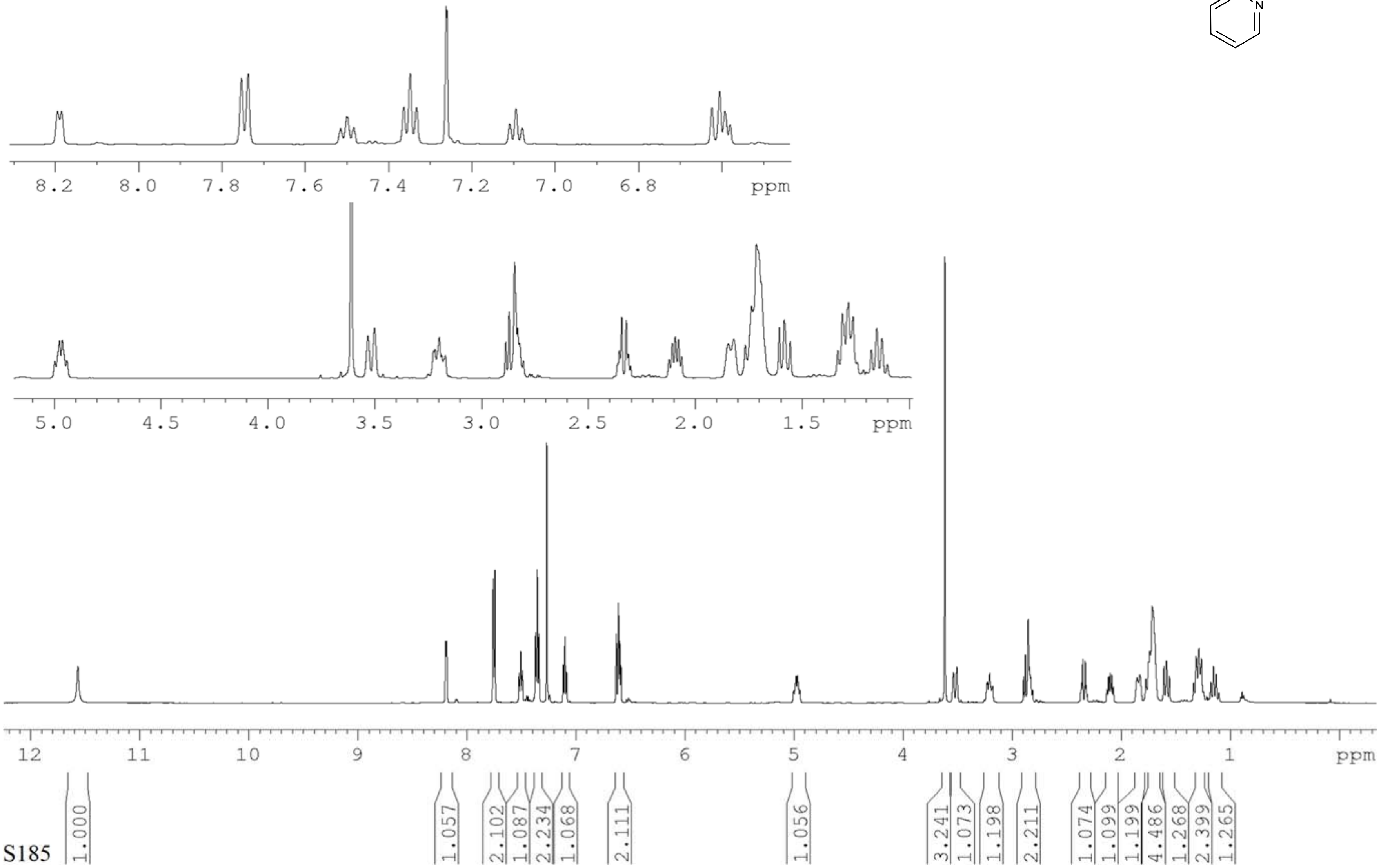

^{13}C NMR (126 MHz, $CDCl_3$) for **Methyl (*R*)-4-oxo-4-(phenylamino)-3-(((*S*)-1-(pyridin-2-yl)azepan-2-yl)methyl)butanoate (5la)**

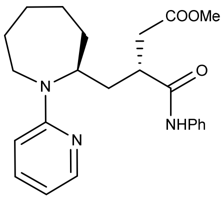

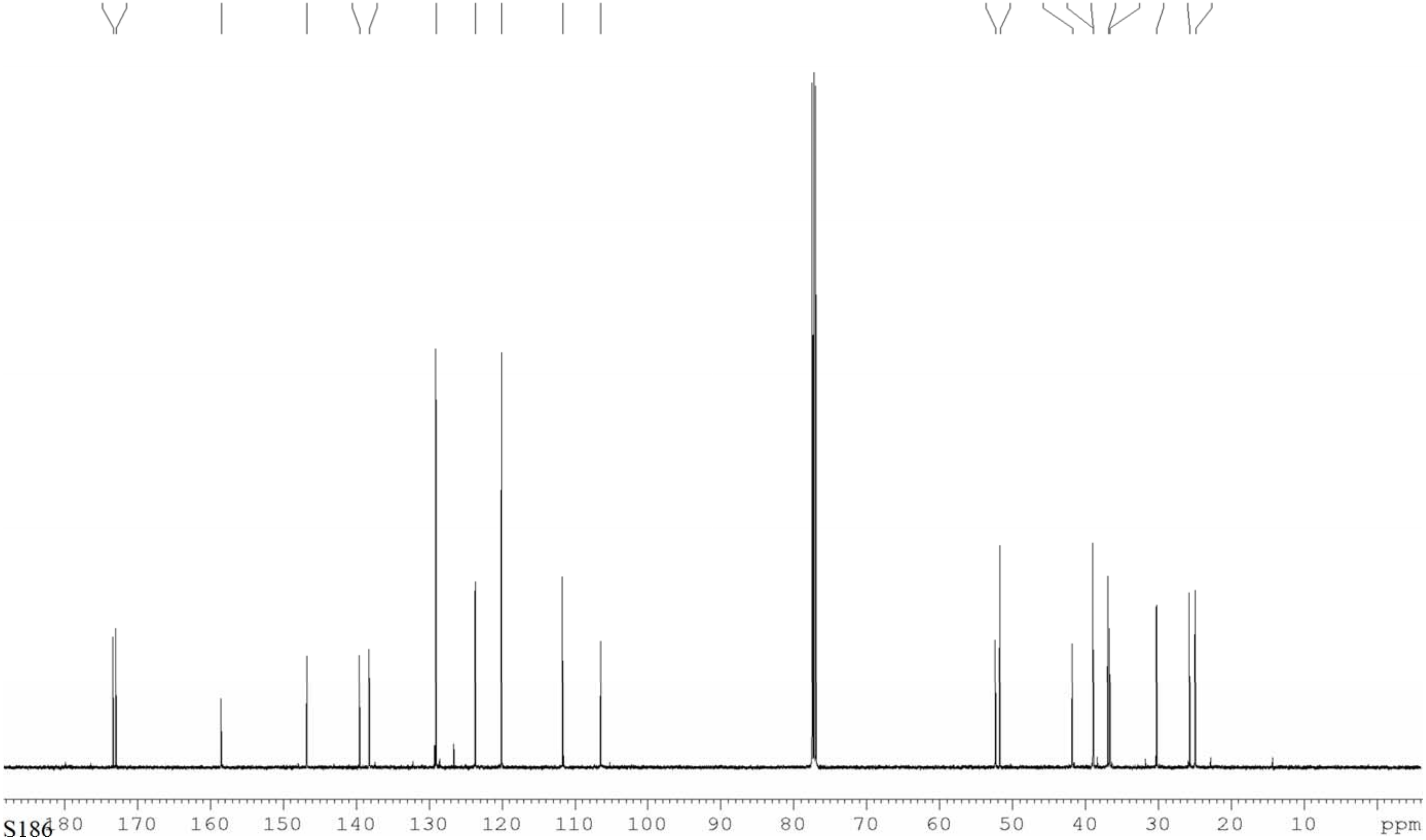

^{1}H NMR (500 MHz, $CDCl_3$) for **methyl (*S*)-4-oxo-4-(phenylamino)-3-(((S)-1-(pyridin-2-yl)azepan-2-yl)methyl)butanoate (5lb)**

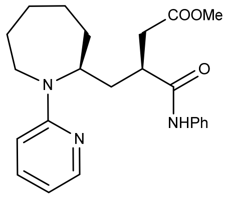

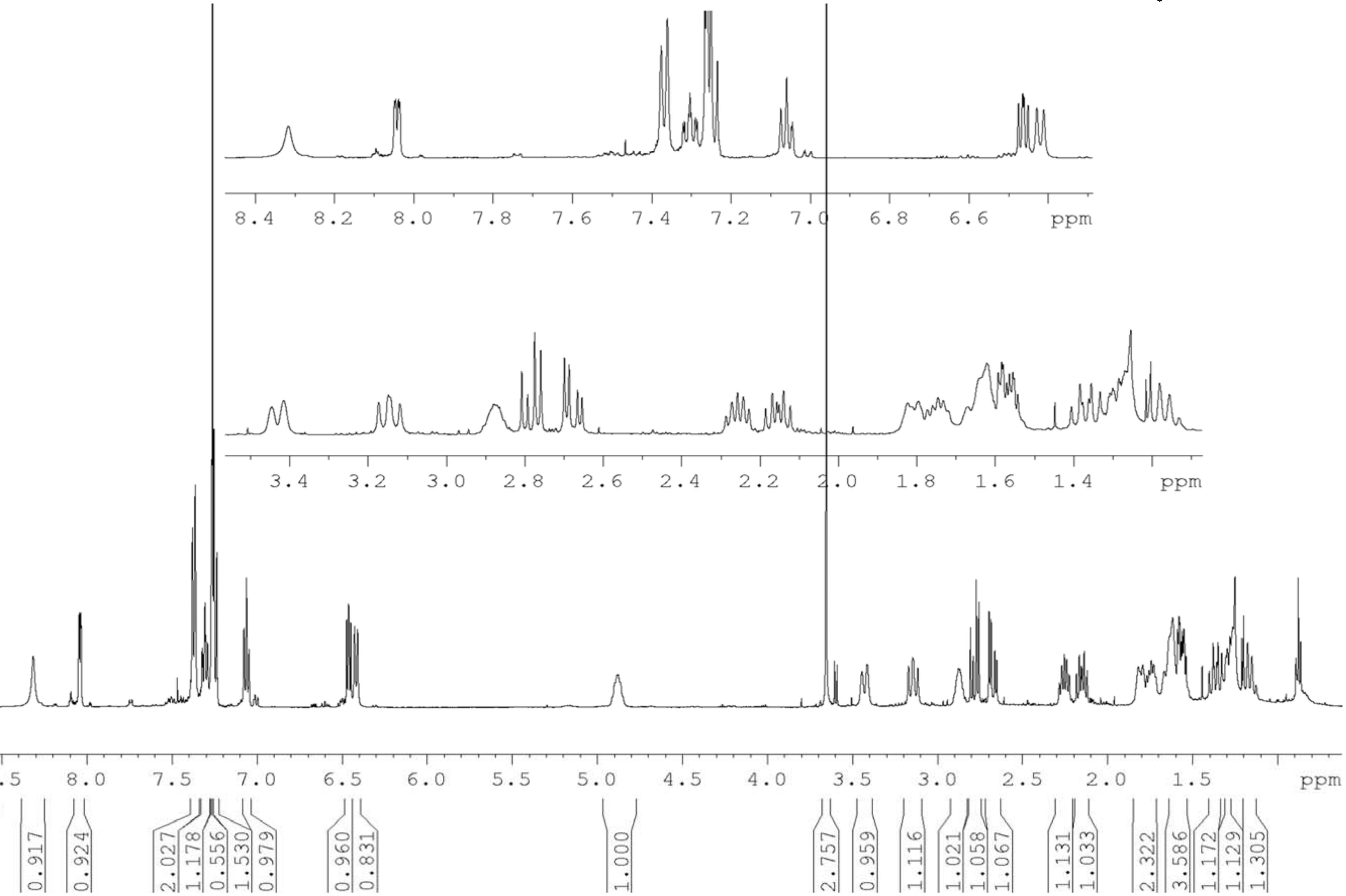

^{13}C NMR (126 MHz, $CDCl_3$) for **methyl (*S*)-4-oxo-4-(phenylamino)-3-(((S)-1-(pyridin-2-yl)azepan-2-yl)methyl)butanoate (5lb)**

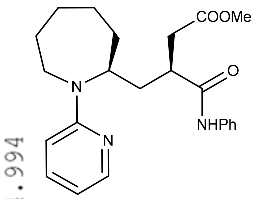

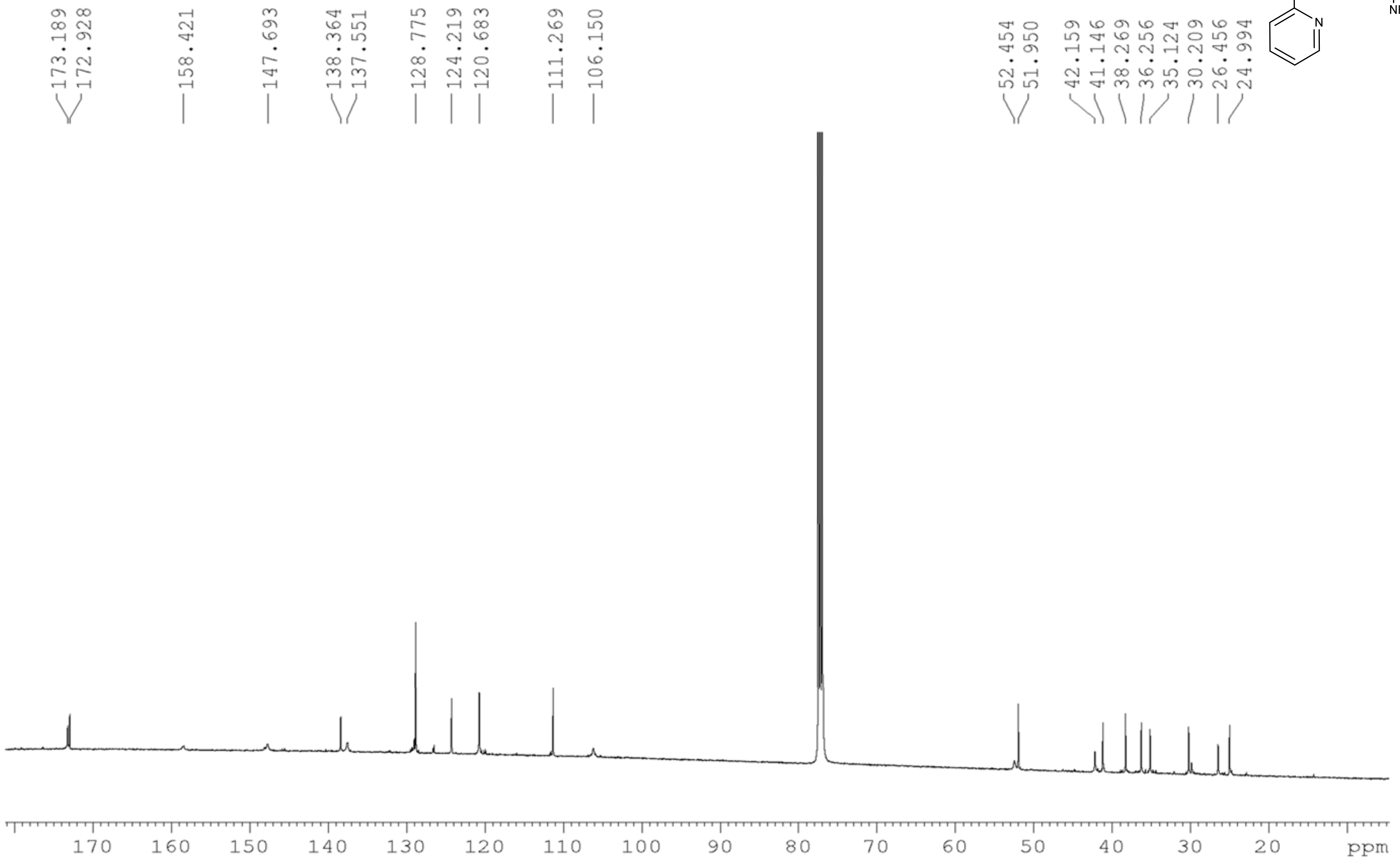

^{1}H NMR (400 MHz, CD_3CN) for **(2*S*,4*S*)-2-Benzyl-4-(ethyl(pyridin-2-yl)amino)-*N*-phenylpentanamide (5pa)**

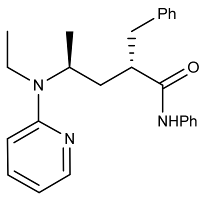

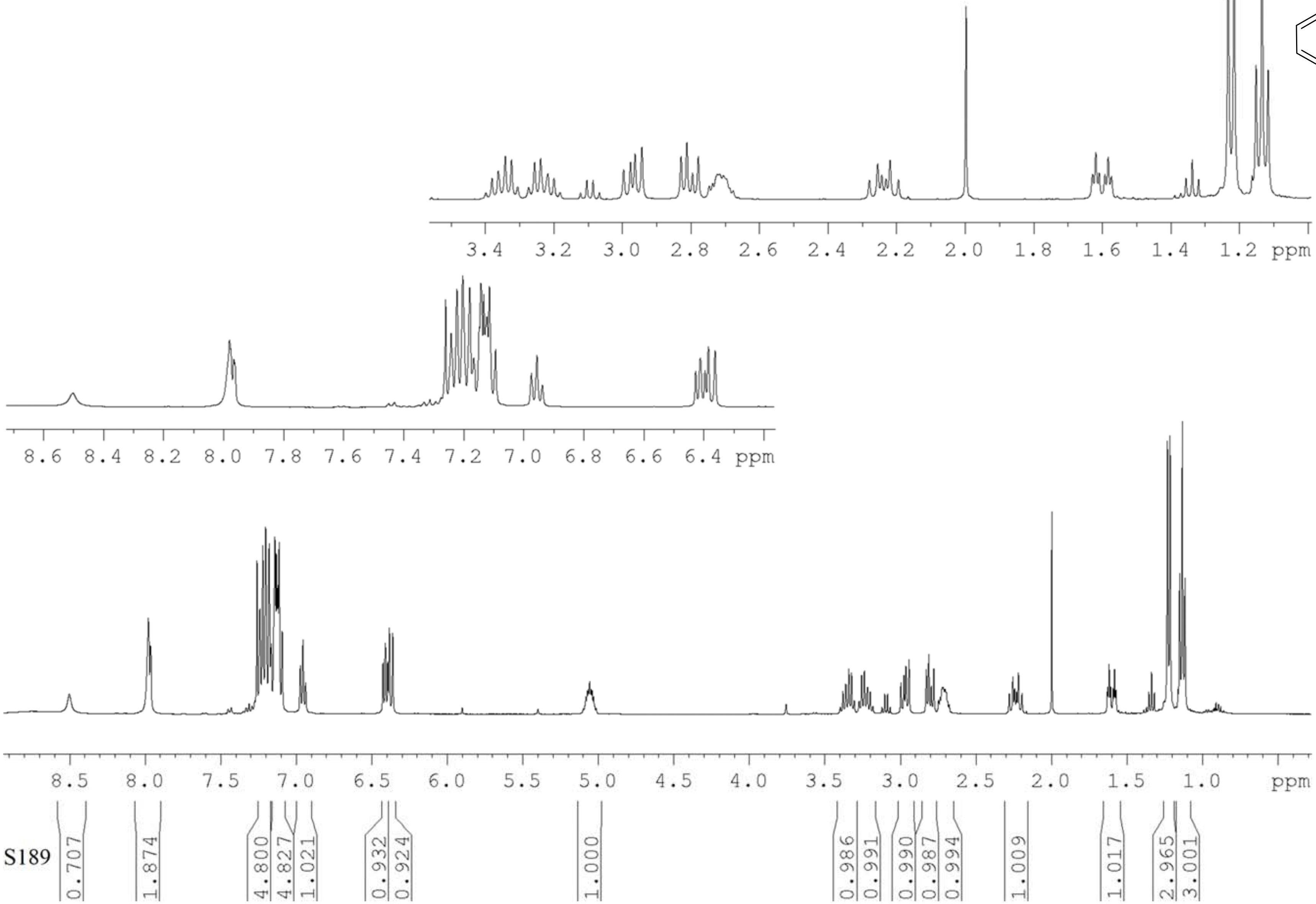

^{13}C NMR (101 MHz, CD_3CN) for **(2*S*,4*S*)-2-Benzyl-4-(ethyl(pyridin-2-yl)amino)-*N*-phenylpentanamide (5pa)**

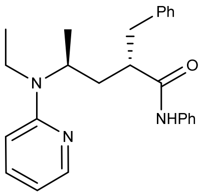

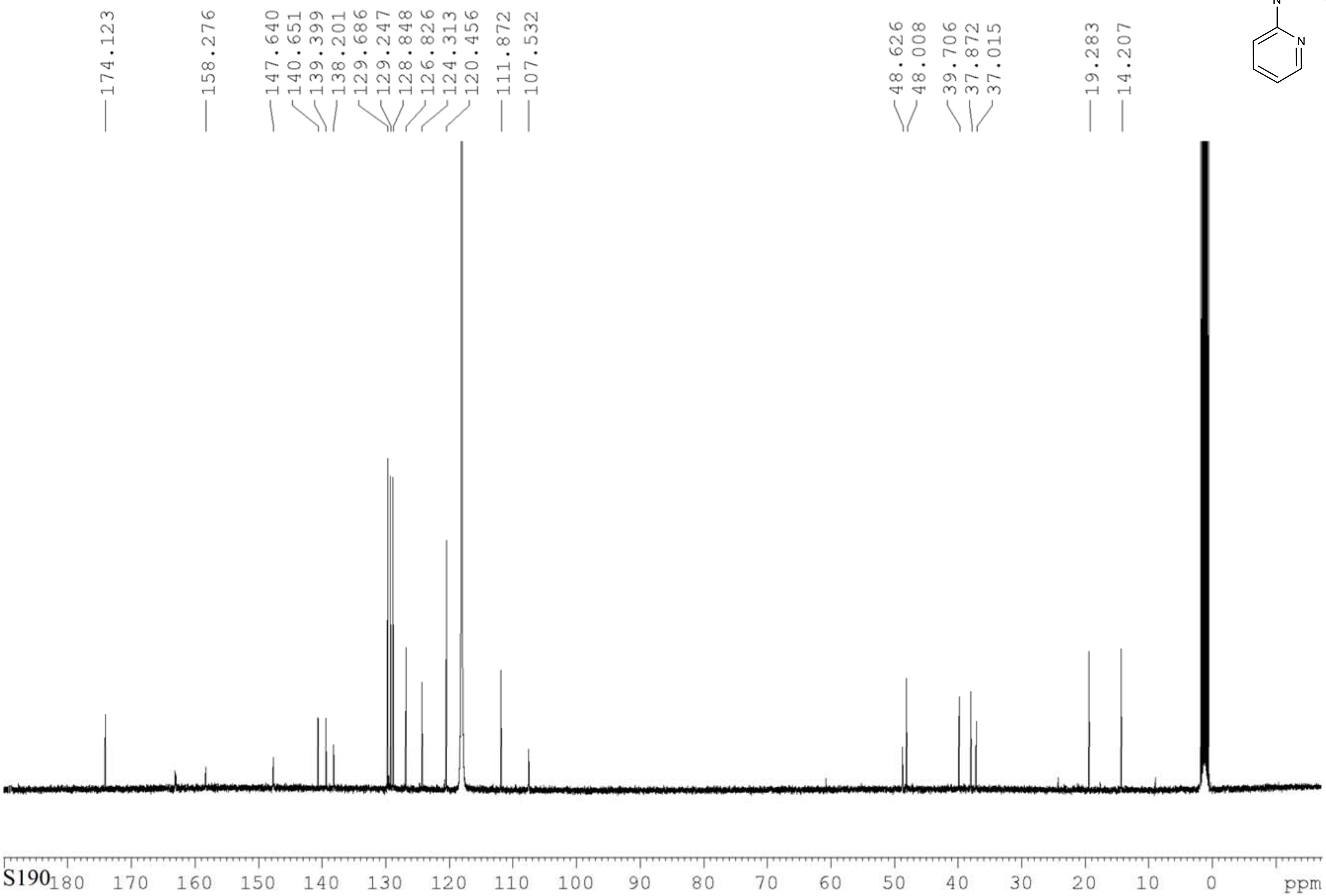

^{1}H NMR (400 MHz, CD_3CN) for **(2*R*,4*S*)-2-Benzyl-4-(ethyl(pyridin-2-yl)amino)-*N*-phenylpentanamide (5pb)**

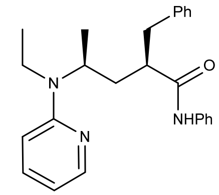

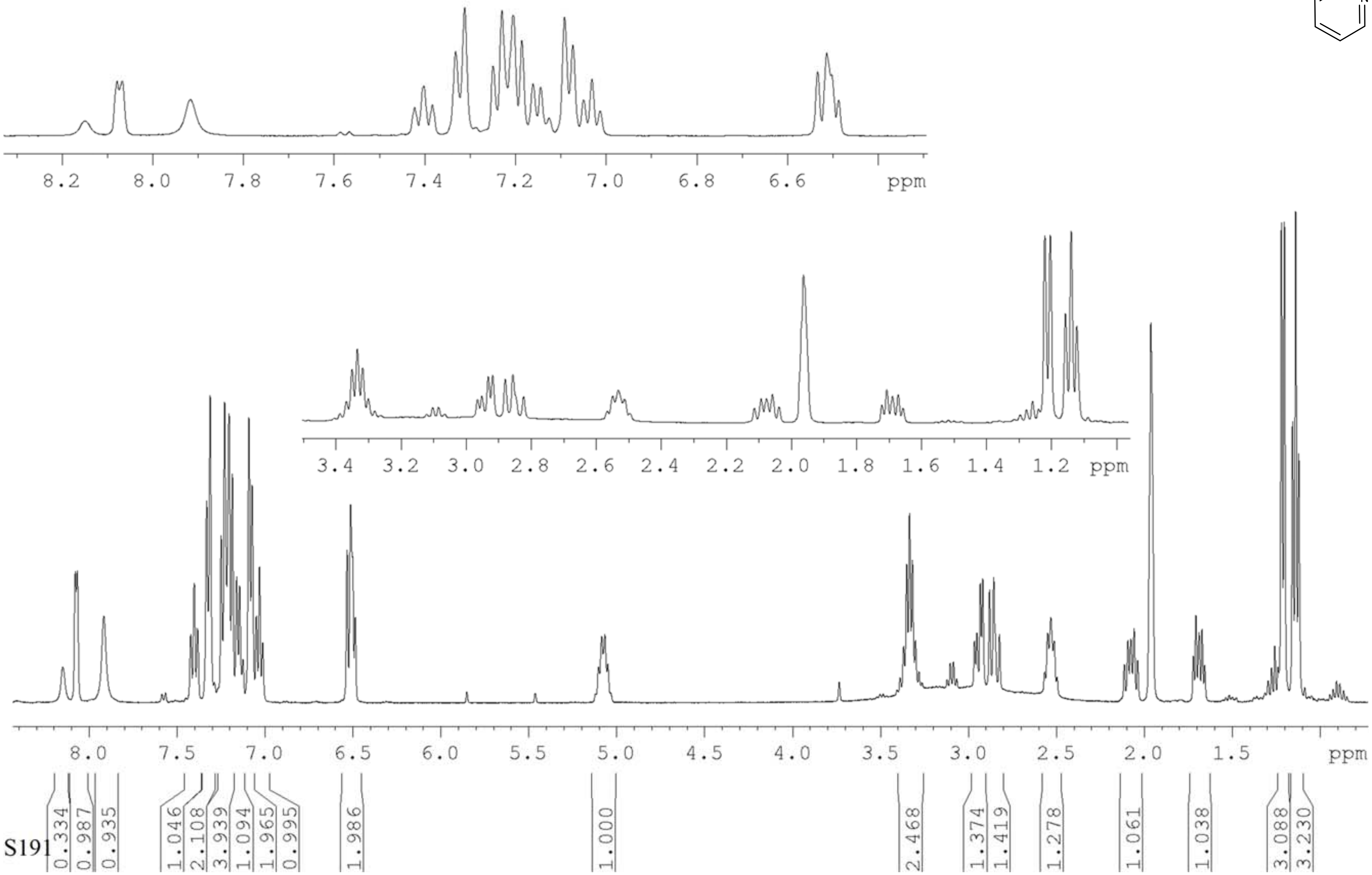

^{13}C NMR (101 MHz, CD_3CN) for **(2*R*,4*S*)-2-Benzyl-4-(ethyl(pyridin-2-yl)amino)-*N*-phenylpentanamide (5pb)**

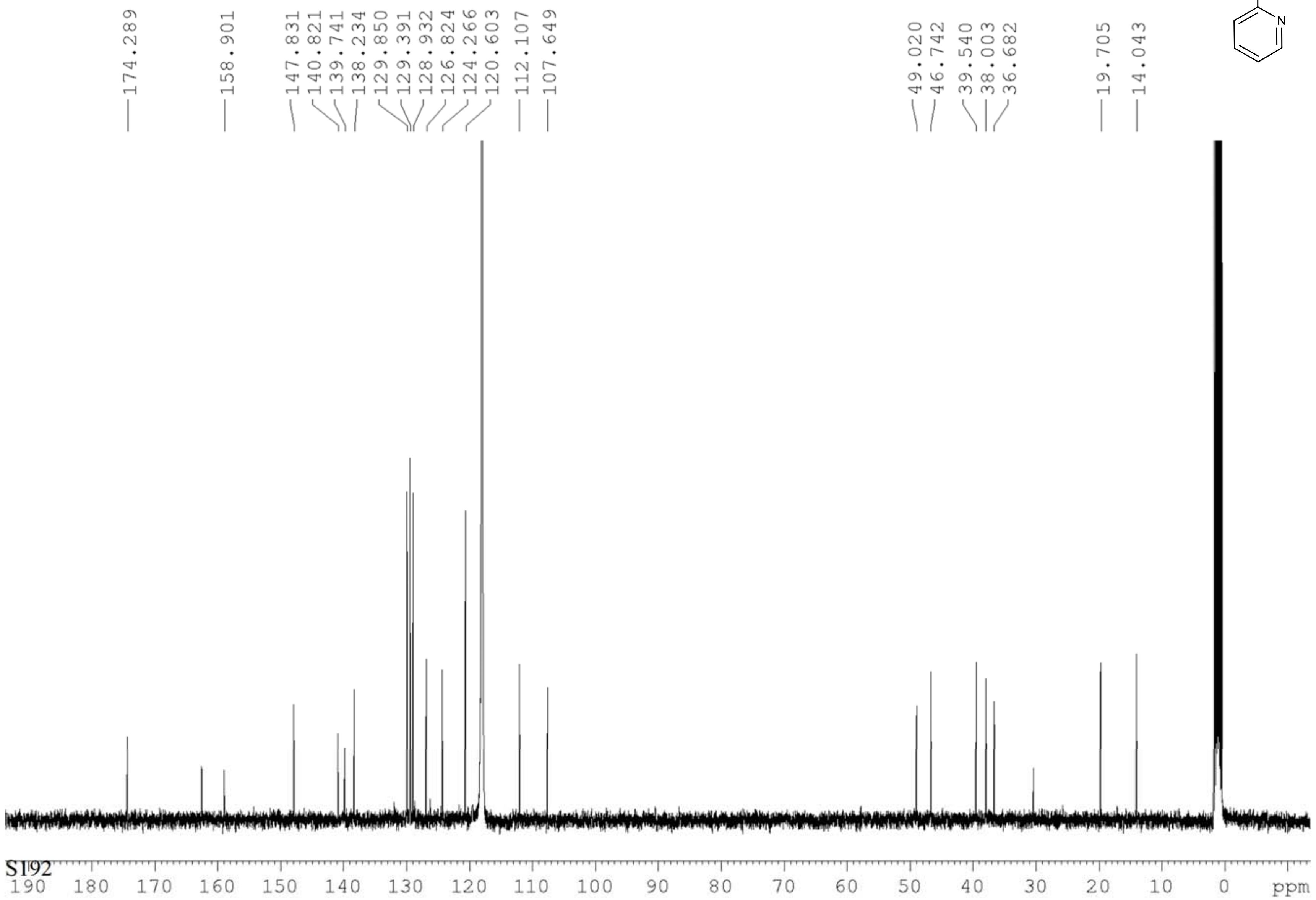

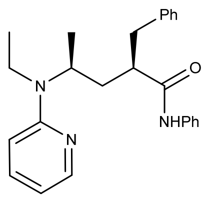

^{1}H NMR (400 MHz, CD_3CN) for **(2*S*,4*S*)-2-Ethyl-4-(ethyl(pyridin-2-yl)amino)-*N*-phenylpentanamide (5qa)**

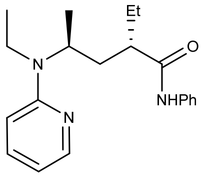

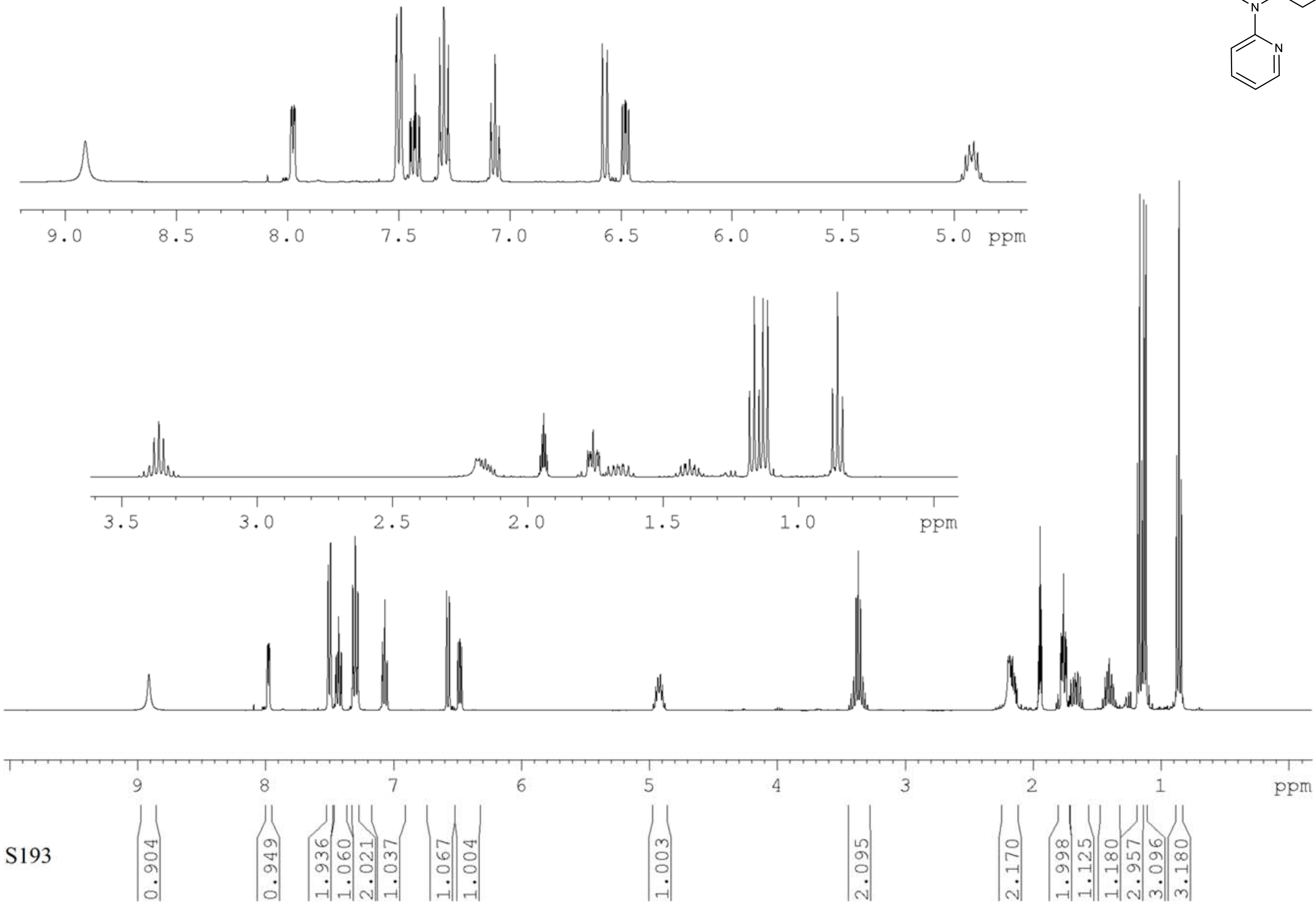

^{13}C NMR (101 MHz, CD_3CN) for **(2*S*,4*S*)-2-Ethyl-4-(ethyl(pyridin-2-yl)amino)-*N*-phenylpentanamide (5qa)**

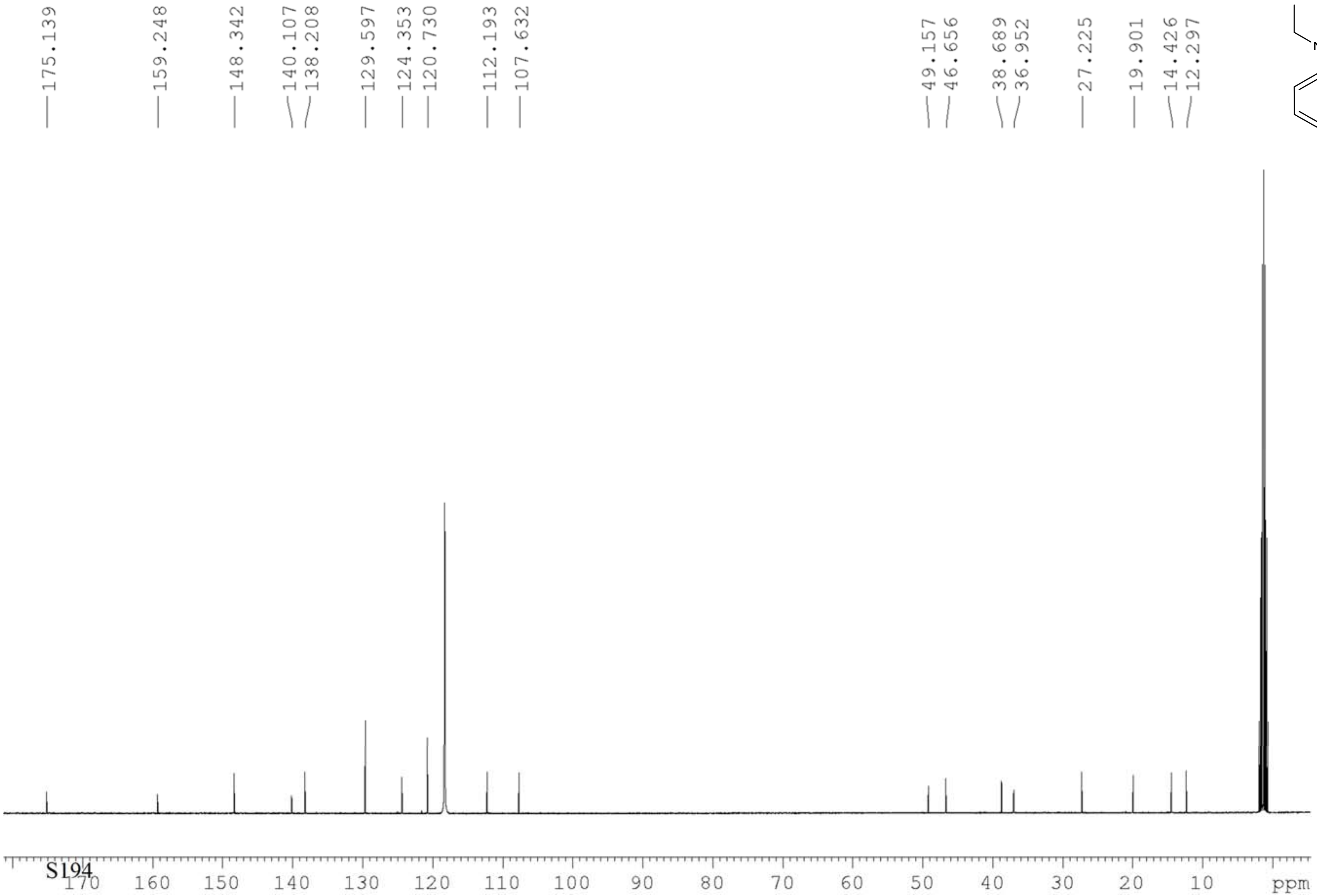

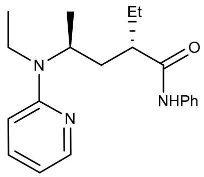

¹H NMR (400 MHz, CD_3CN) for **(2*R*,4*S*)-2-ethyl-4-(ethyl(pyridin-2-yl)amino)-*N*-phenylpentanamide (5qb)**

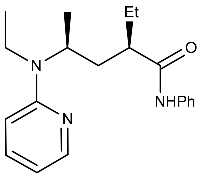

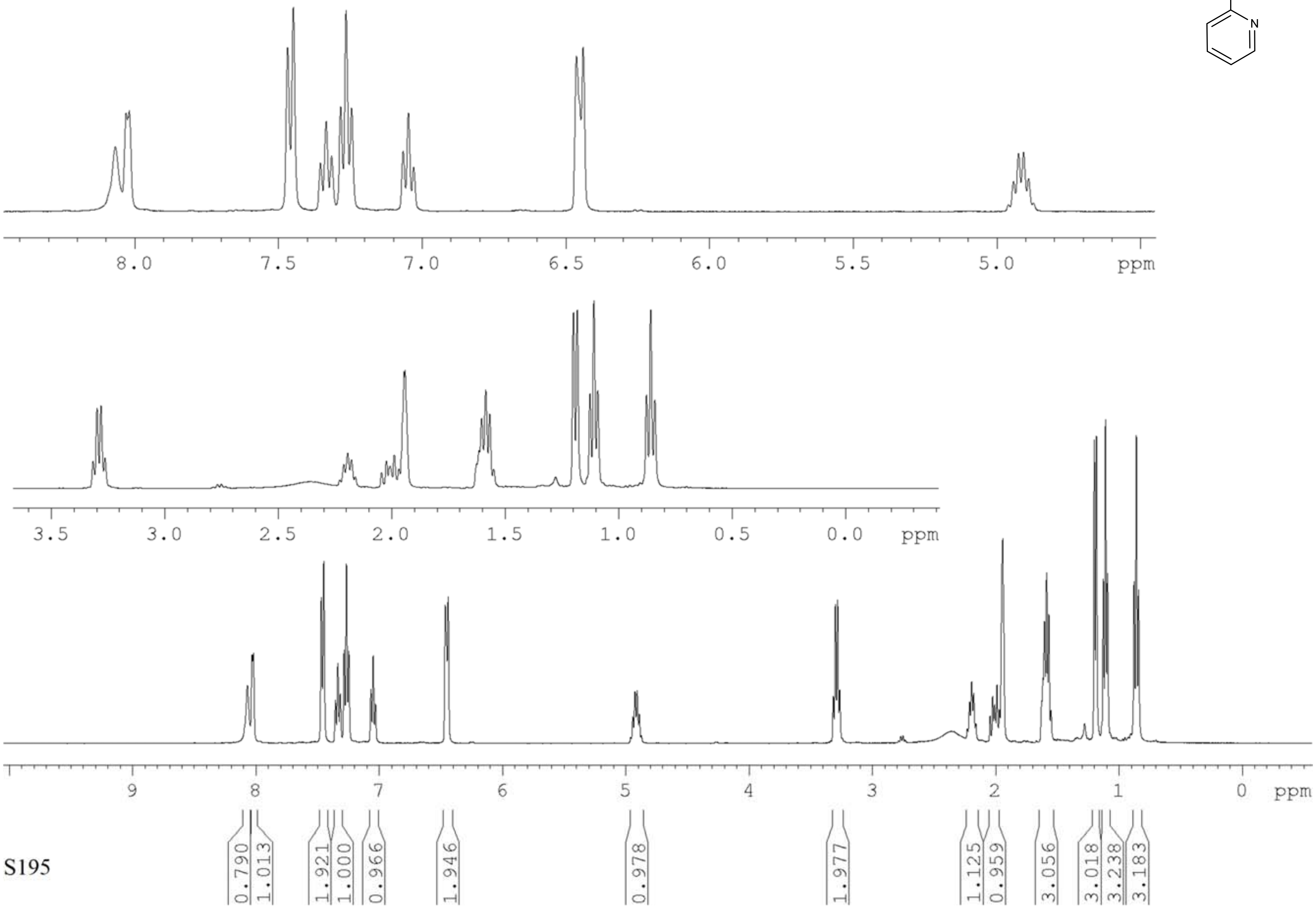

^{13}C NMR (101 MHz, CD_3CN) for **(2*R*,4*S*)-2-ethyl-4-(ethyl(pyridin-2-yl)amino)-*N*-phenylpentanamide (5qb)**

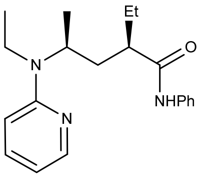

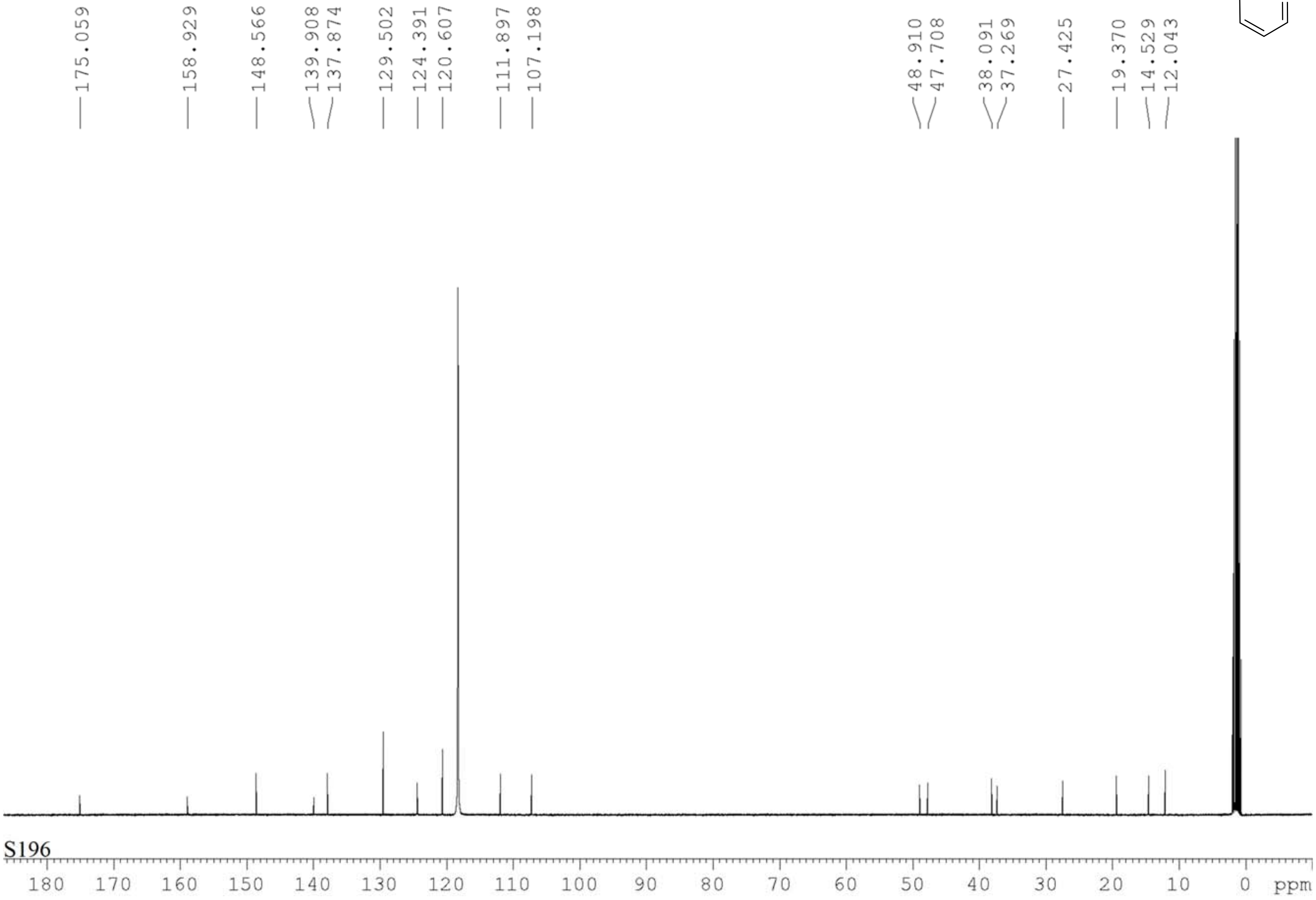

¹H NMR (500 MHz, CD_3CN) for **(2*S*,4*S*)-2-Ethyl-*N*-phenyl-4-(phenyl(pyridin-2-yl)amino)pentanamide (5ra)**

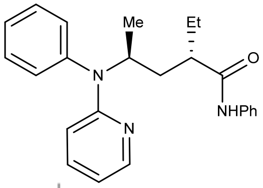

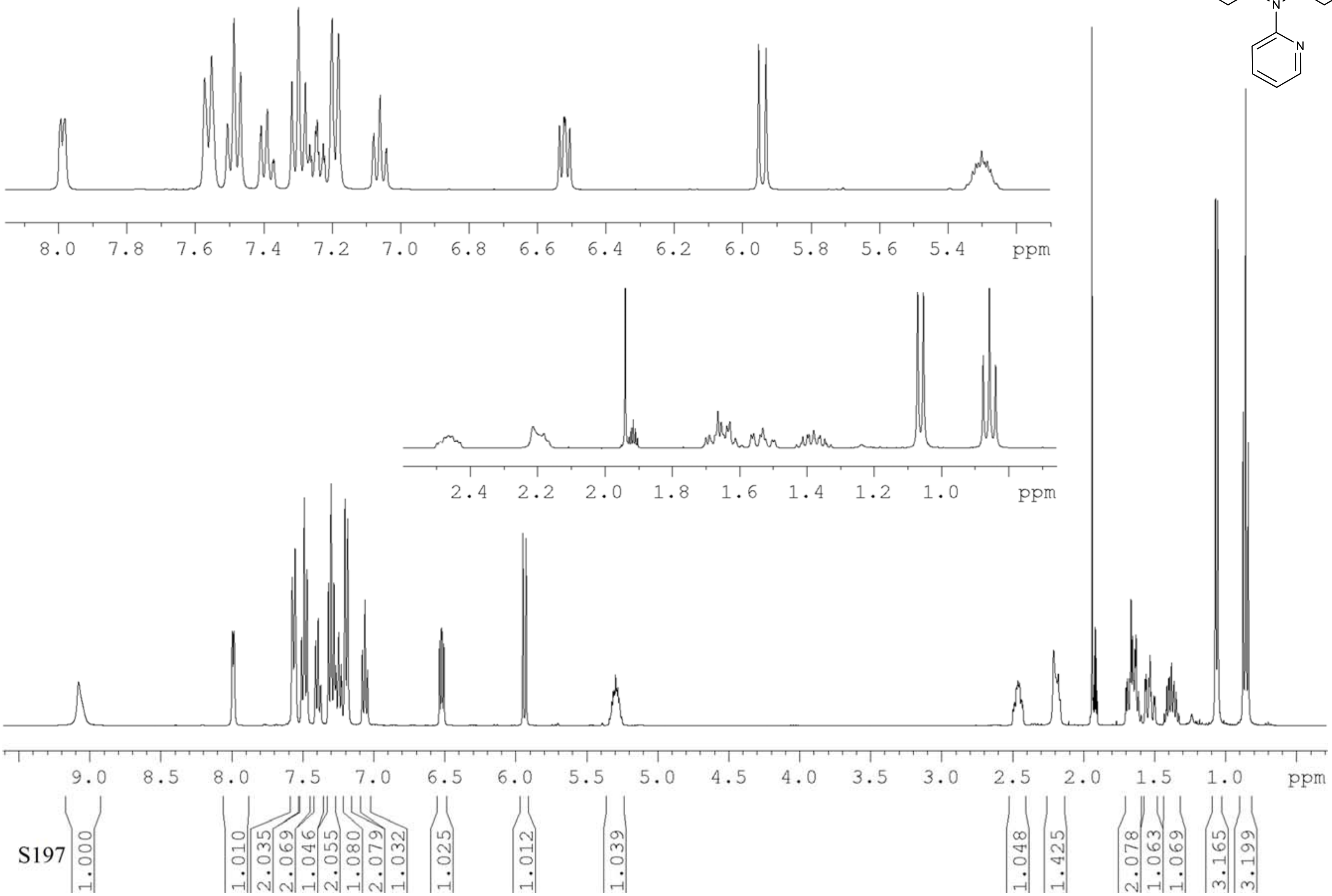

^{13}C NMR (126 MHz, CD_3CN) for **(2*S*,4*S*)-2-Ethyl-*N*-phenyl-4-(phenyl(pyridin-2-yl)amino)pentanamide (5ra)**

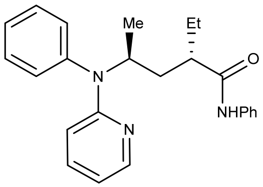

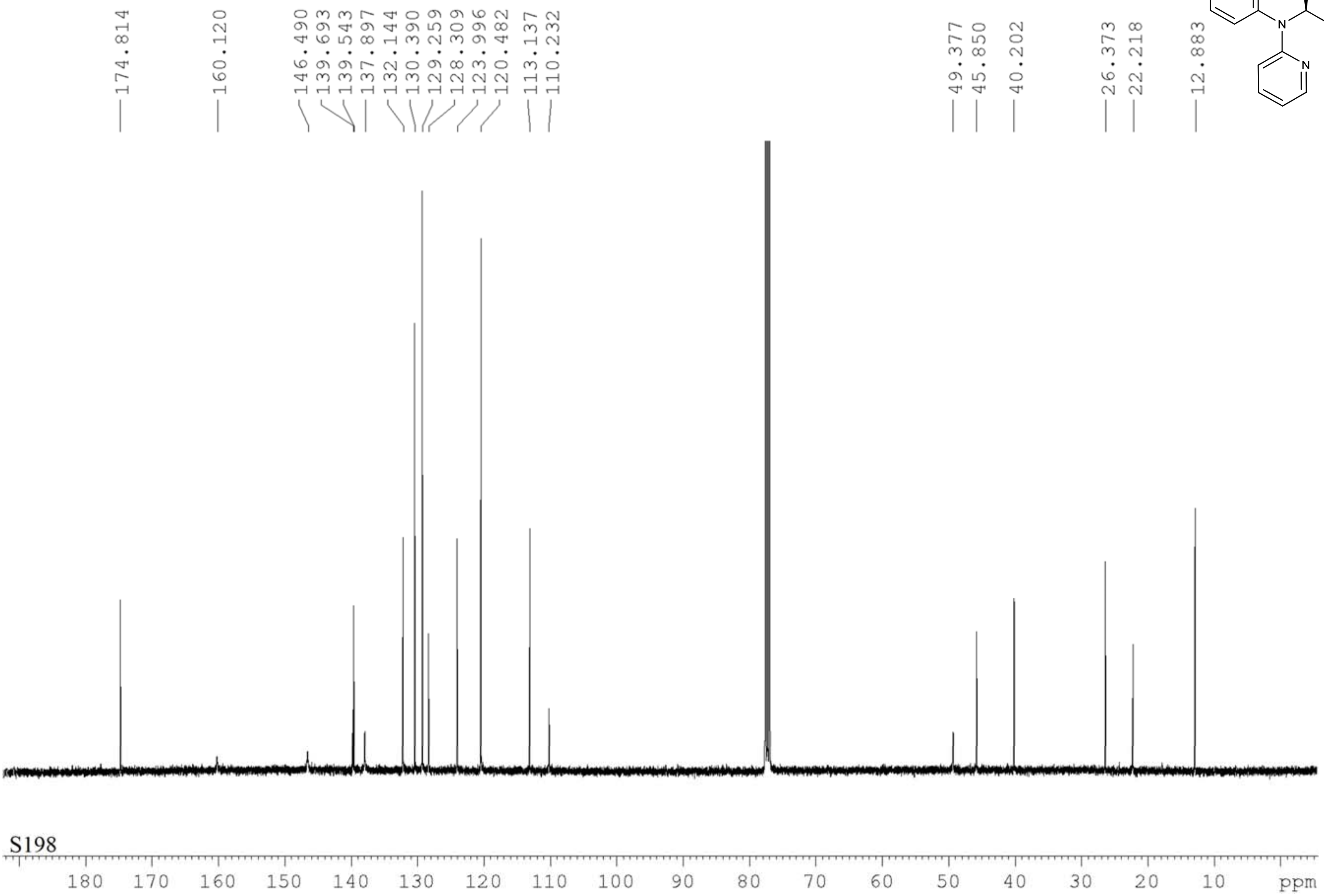

^{1}H NMR (500 MHz, CD_3CN) for **(2*R*,4*S*)-2-Ethyl-*N*-phenyl-4-(phenyl(pyridin-2-yl)amino)pentanamide (5rb)**

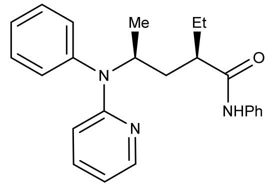

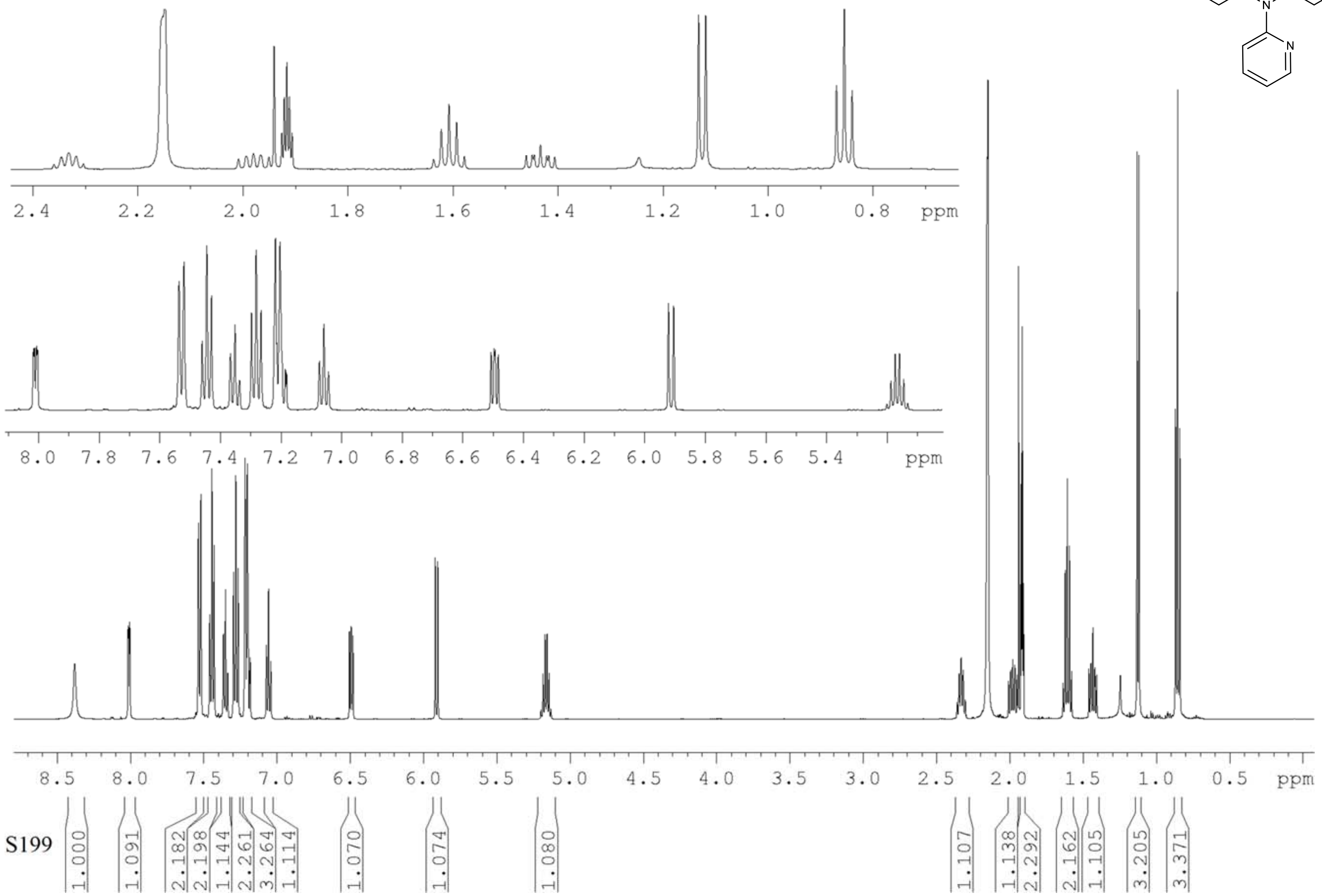

^{13}C NMR (126 MHz, CD_3CN) for **(2*R*,4*S*)-2-Ethyl-*N*-phenyl-4-(phenyl(pyridin-2-yl)amino)pentanamide (5rb)**

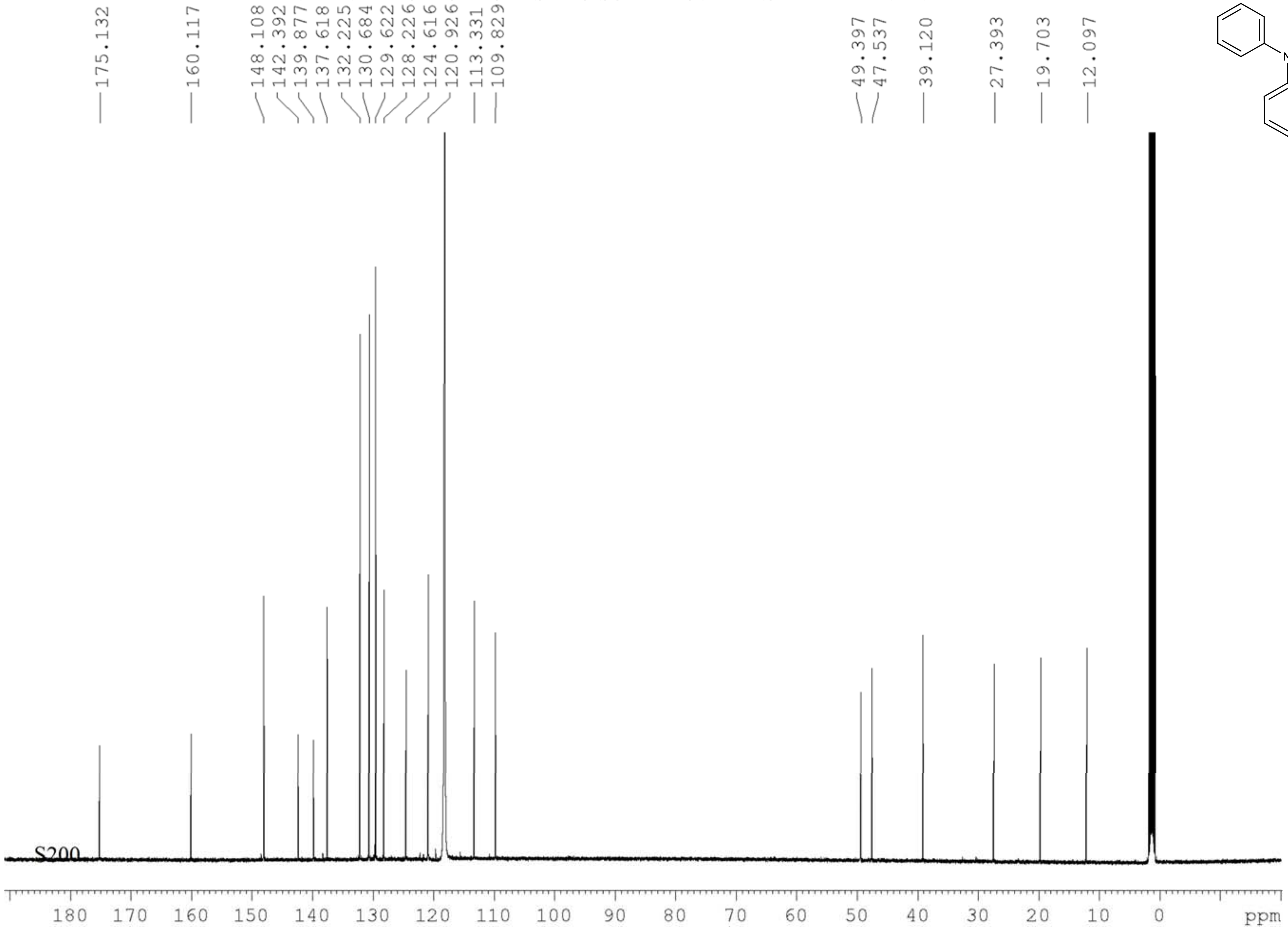

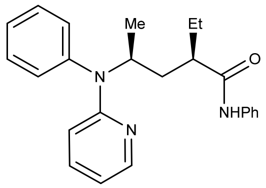

¹H NMR (400 MHz, CD_3CN) for **(2*S*,4*S*)-4-(Benzyl(pyridin-2-yl)amino)-2-ethyl-*N*-phenylpentanamide (5sa)**

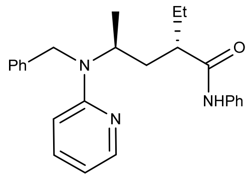

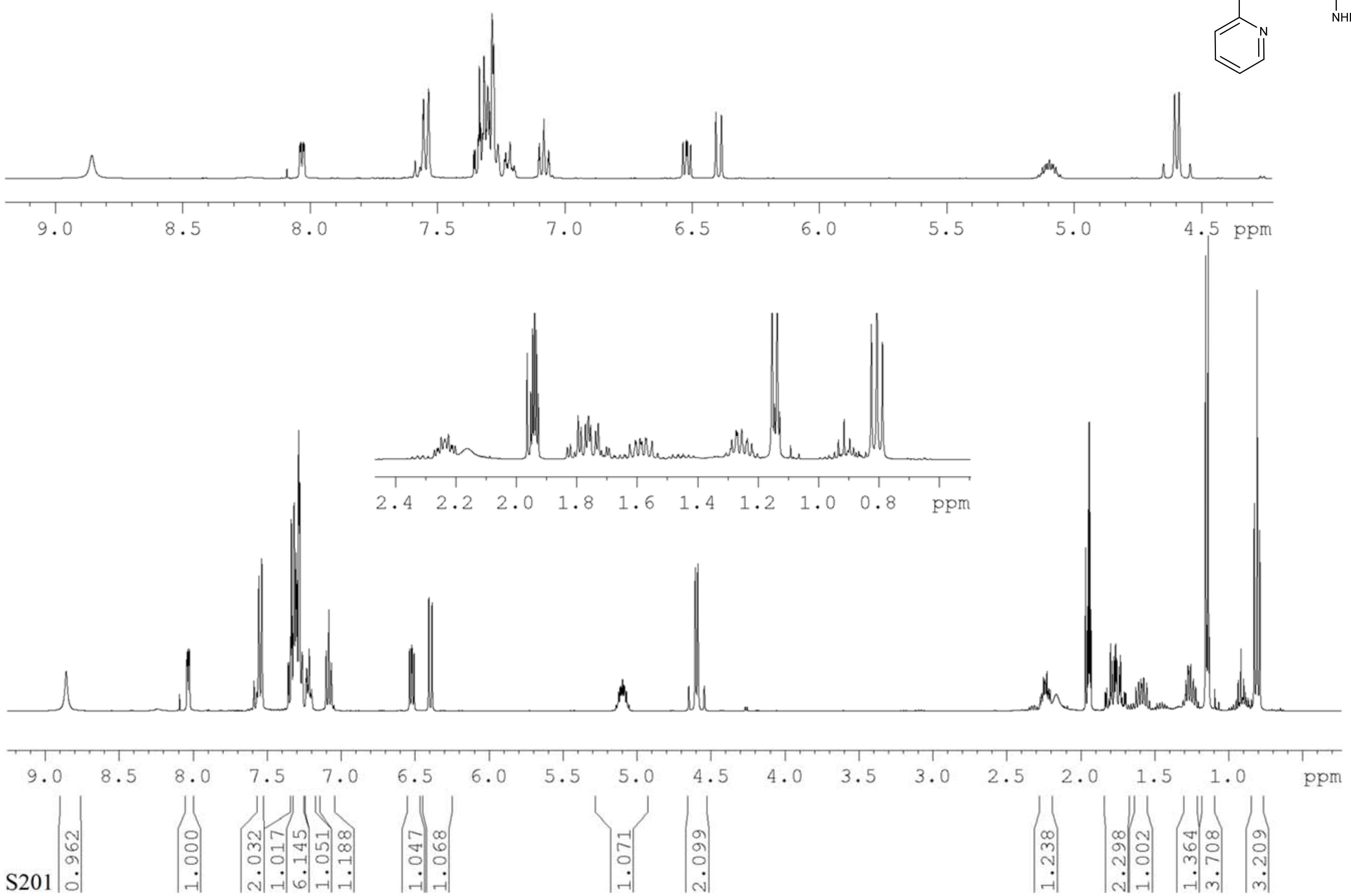

^{13}C NMR (101 MHz, CD_3CN) for **(2*S*,4*S*)-4-(Benzyl(pyridin-2-yl)amino)-2-ethyl-*N*-phenylpentanamide (5sa)**

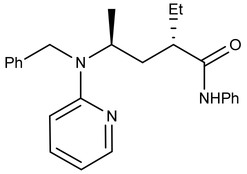

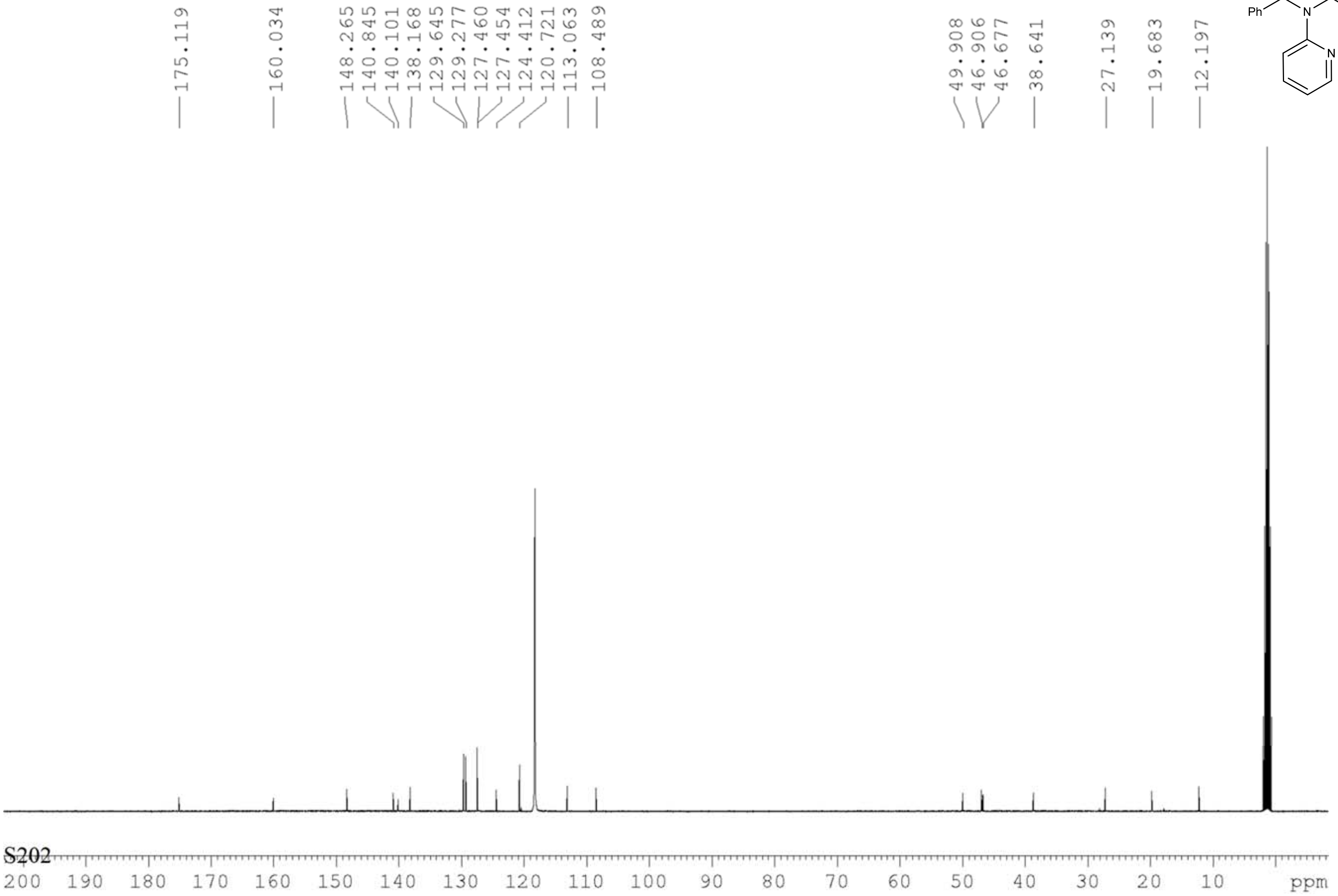

^{1}H NMR (400 MHz, CD_3CN) for **(2*R*,4*S*)-4-(Benzyl(pyridin-2-yl)amino)-2-ethyl-*N*-phenylpentanamide (5sb)**

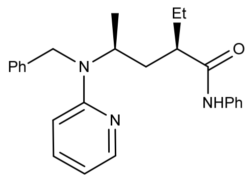

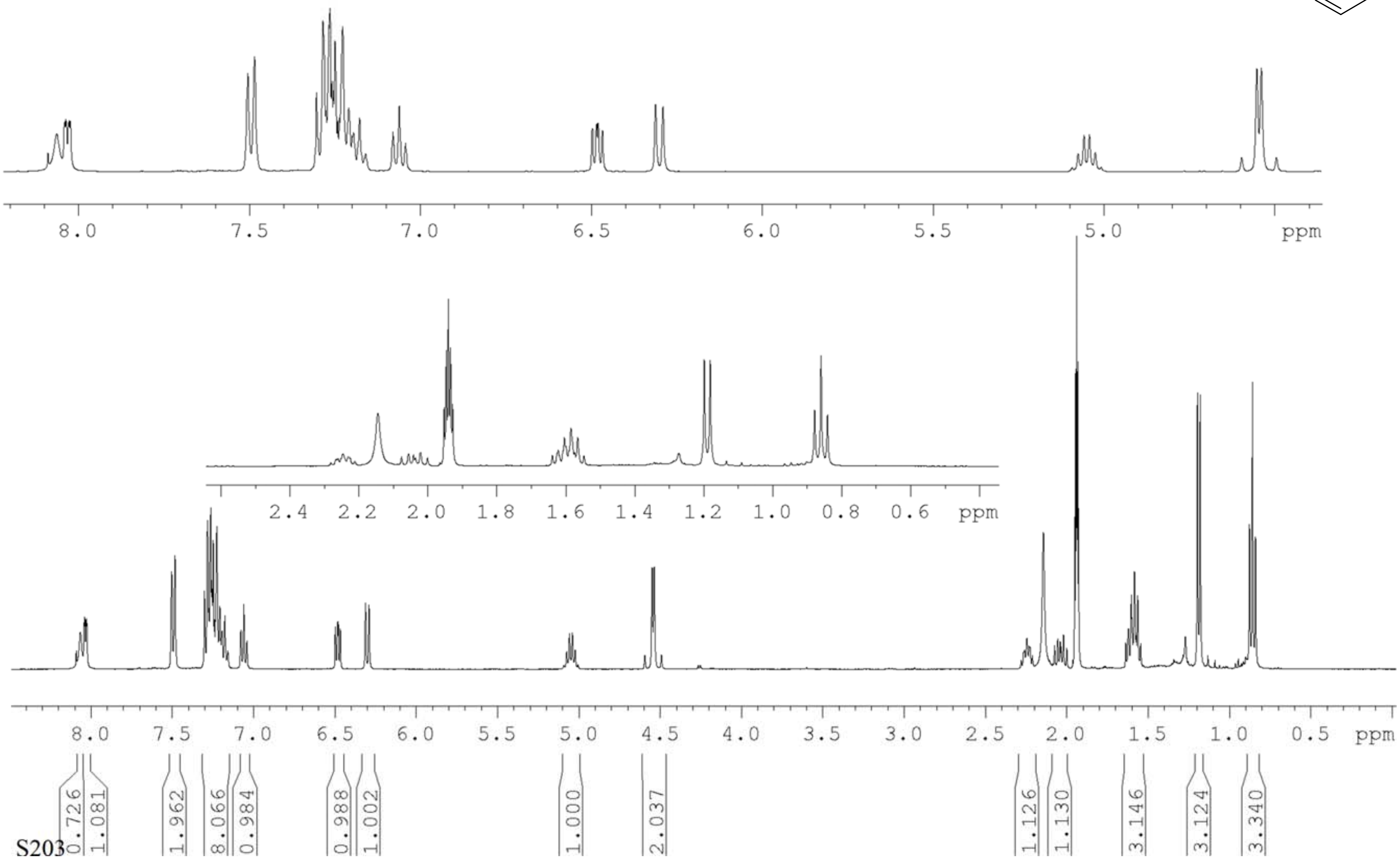

^{13}C NMR (101 MHz, CD_3CN) for **(2*R*,4*S*)-4-(Benzyl(pyridin-2-yl)amino)-2-ethyl-*N*-phenylpentanamide (5sb)**

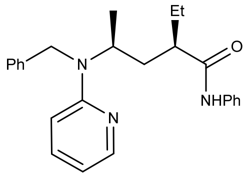

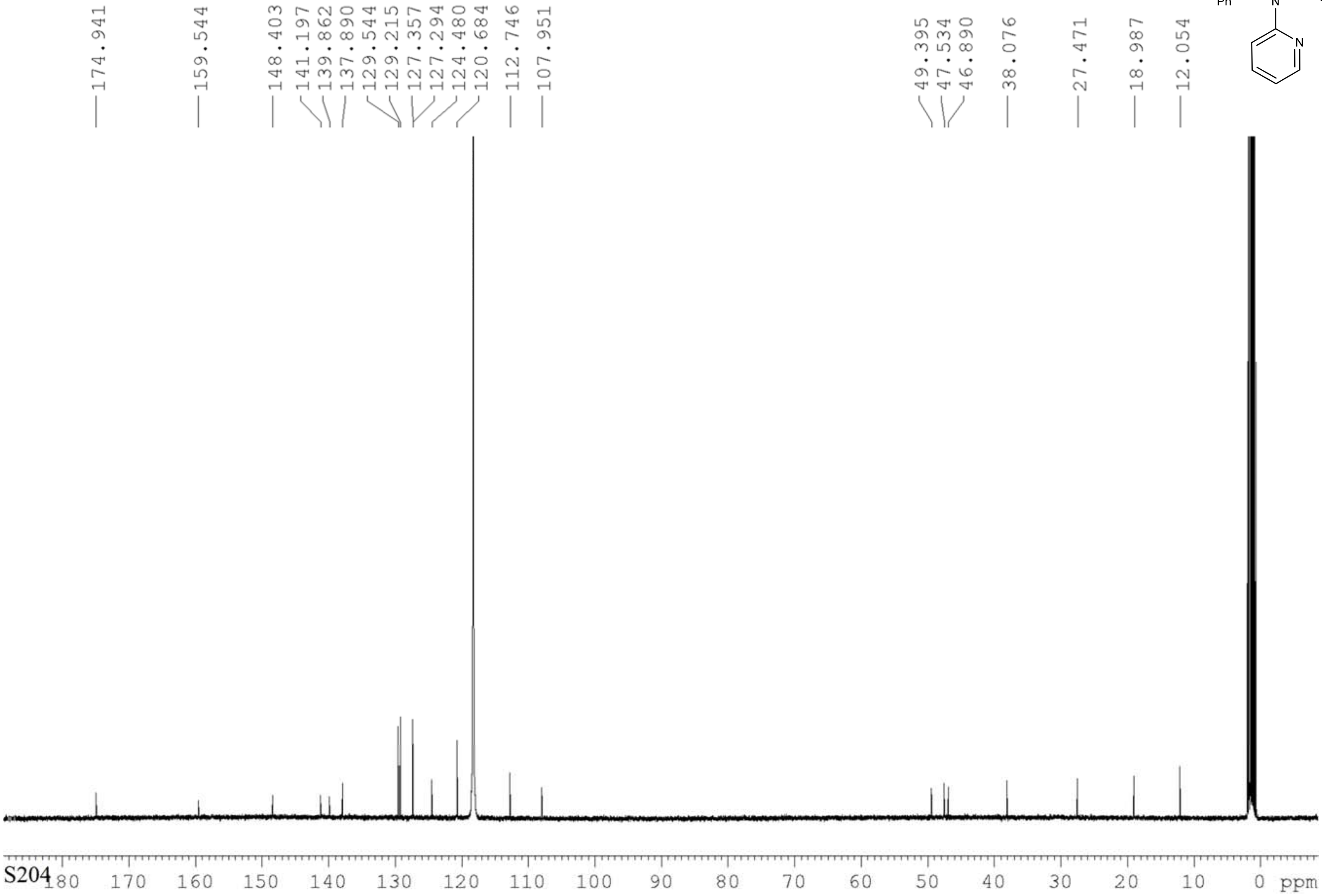

^{1}H NMR (400 MHz, $CHCl_3$) for **2-Benzyl-4-(methyl(pyridin-2-yl)amino)-*N*-phenylbutanamide (5t)**

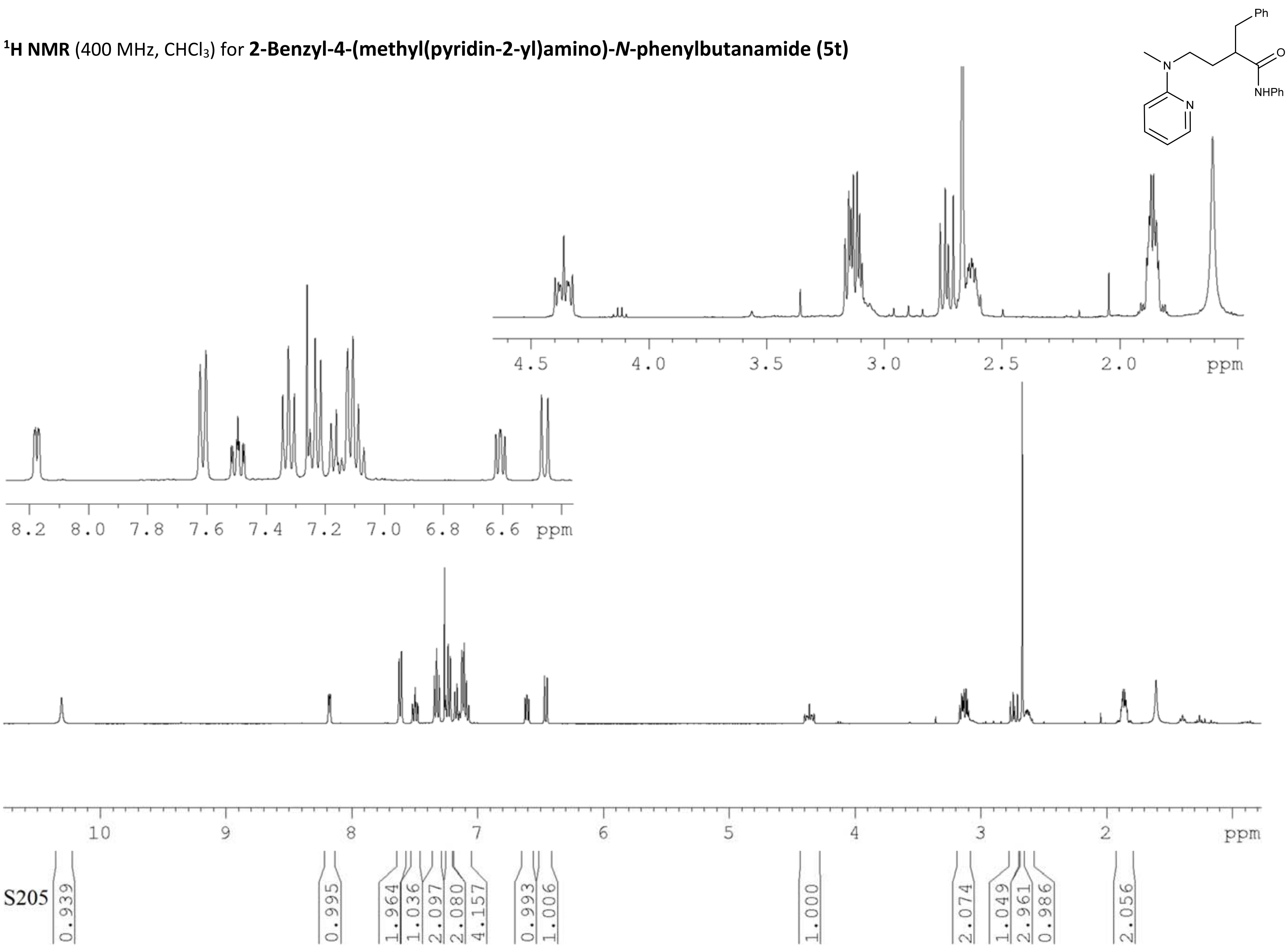

^{13}C NMR (101 MHz, $CDCl_3$) for **2-Benzyl-4-(methyl(pyridin-2-yl)amino)-*N*-phenylbutanamide (5t)**

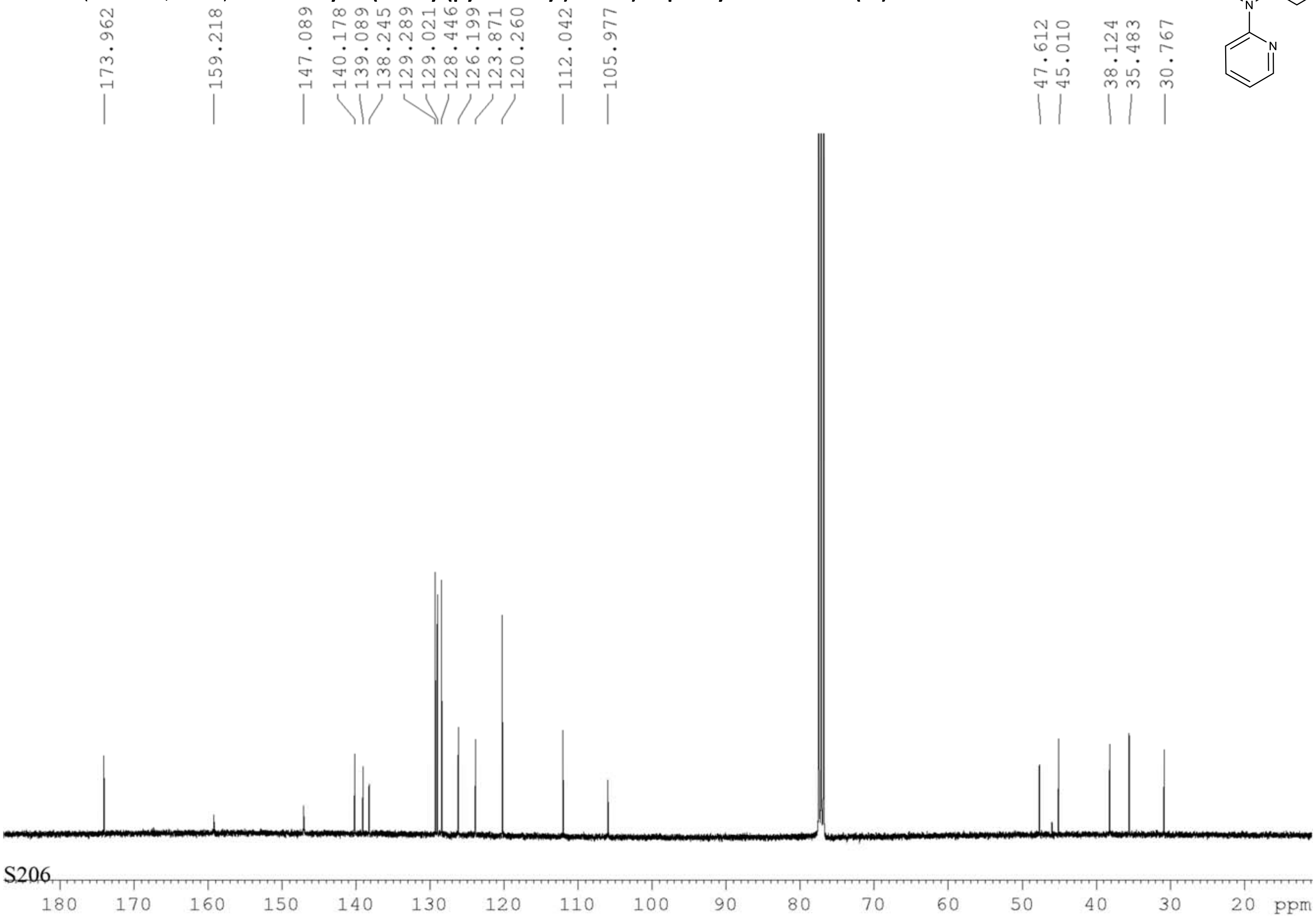

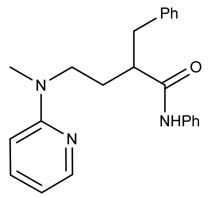

^{1}H NMR (500 MHz, CD_3CN) for **(*R*)-3-Methyl-4-(methyl(pyridin-2-yl)amino)-*N*-phenylbutanamide (6a)**

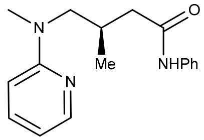

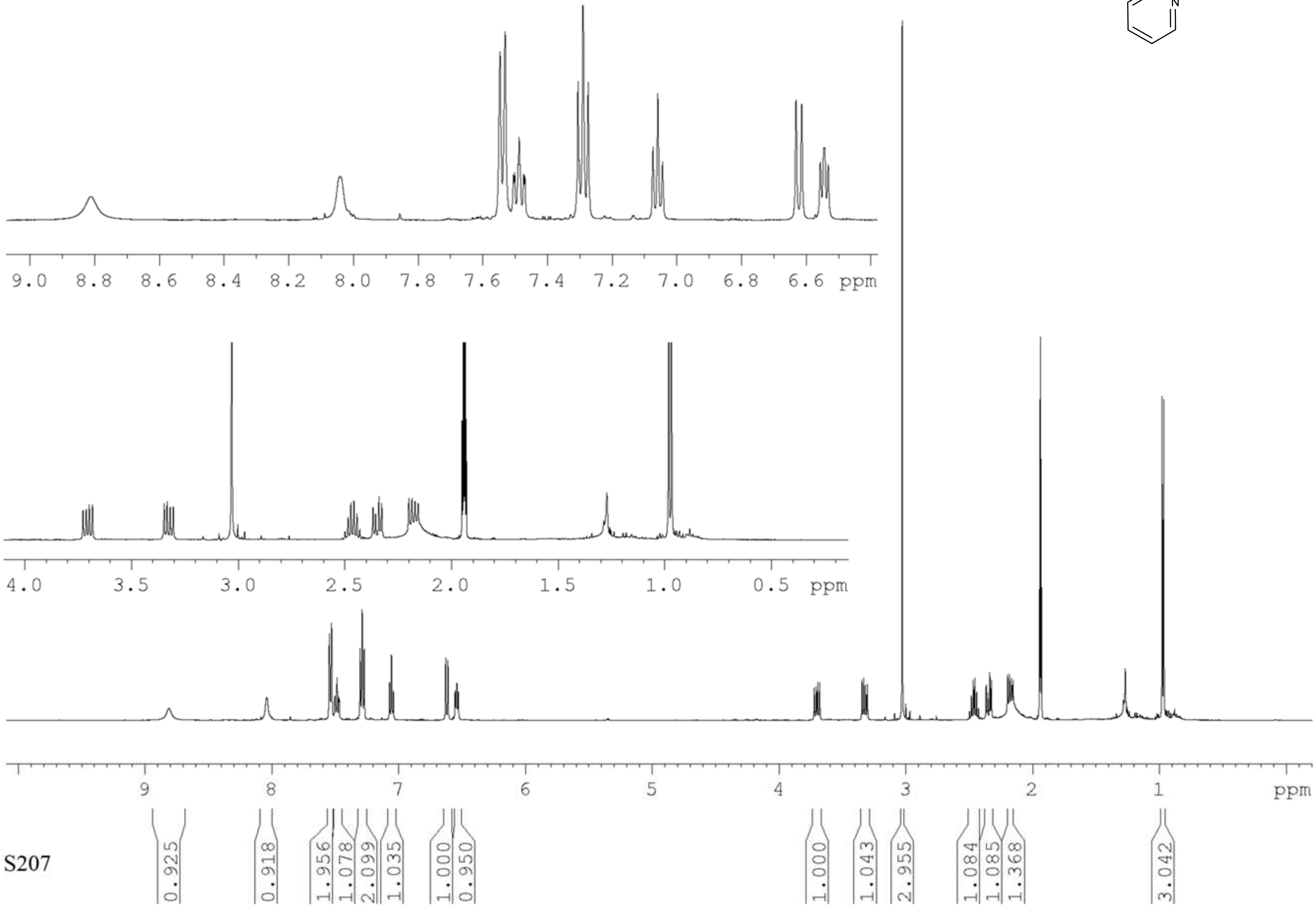

^{13}C NMR (126 MHz, CD_3CN) for **(*R*)-3-Methyl-4-(methyl(pyridin-2-yl)amino)-*N*-phenylbutanamide (6a)**

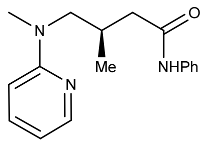

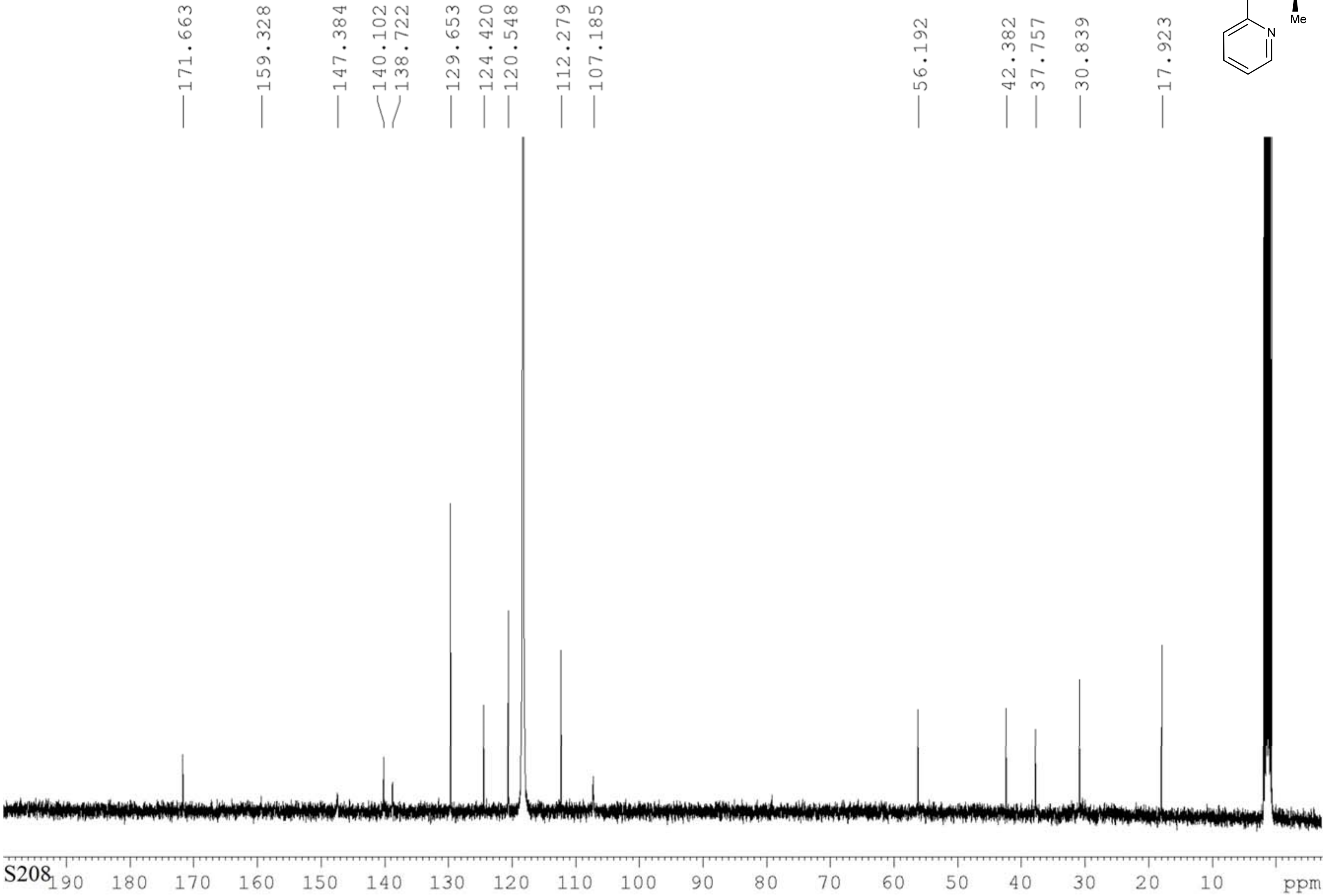

^{1}H NMR (400 MHz, CD_3CN) for **(*R*)-3-Methyl-*N*-phenyl-4-(phenyl(pyridin-2-yl)amino)butanamide (6b)**

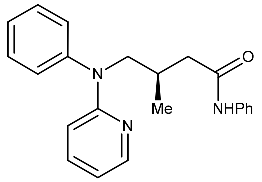

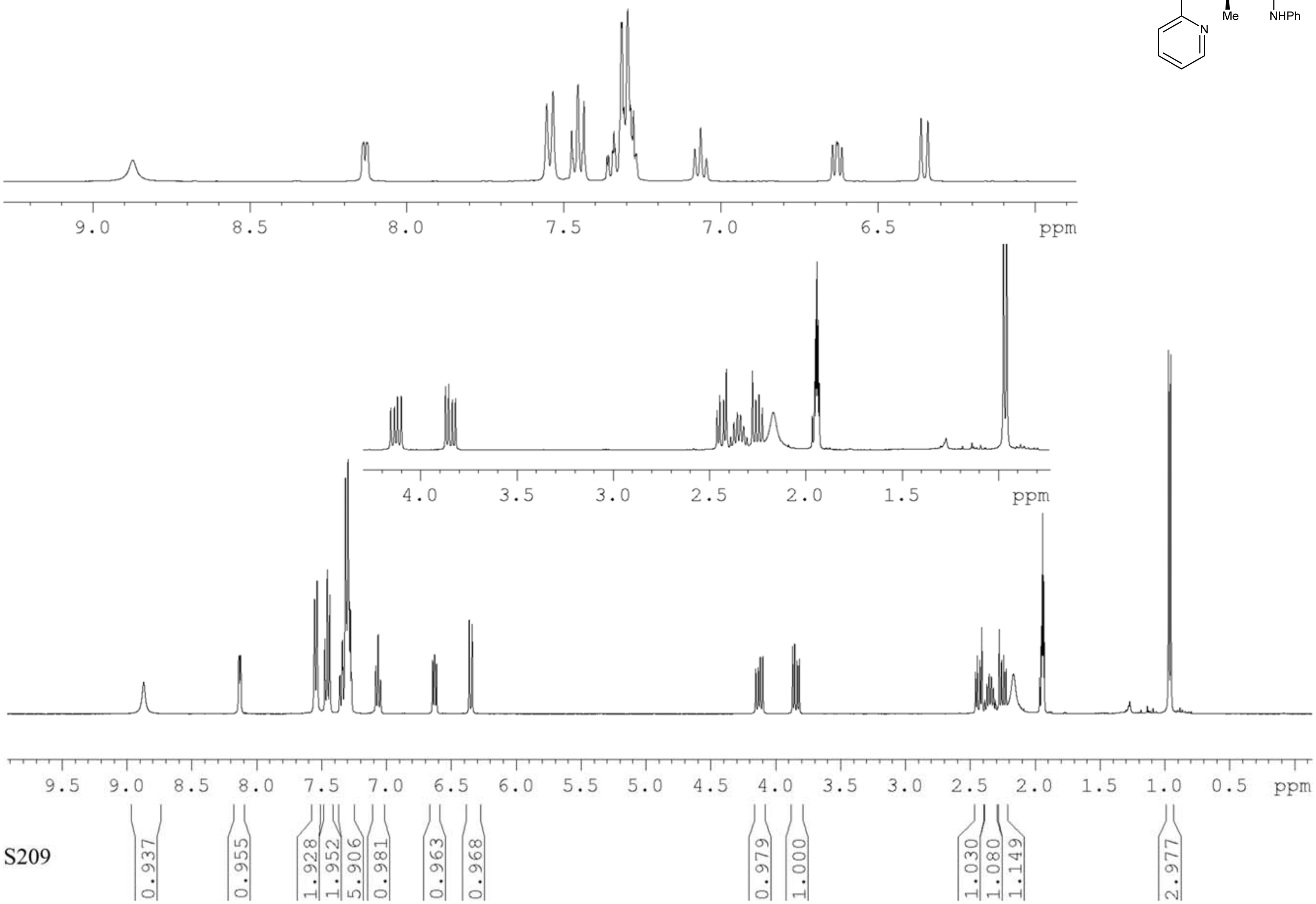

^{13}C NMR (101 MHz, CD_3CN) for **(*R*)-3-Methyl-*N*-phenyl-4-(phenyl(pyridin-2-yl)amino)butanamide (6b)**

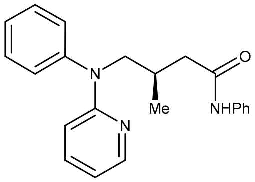

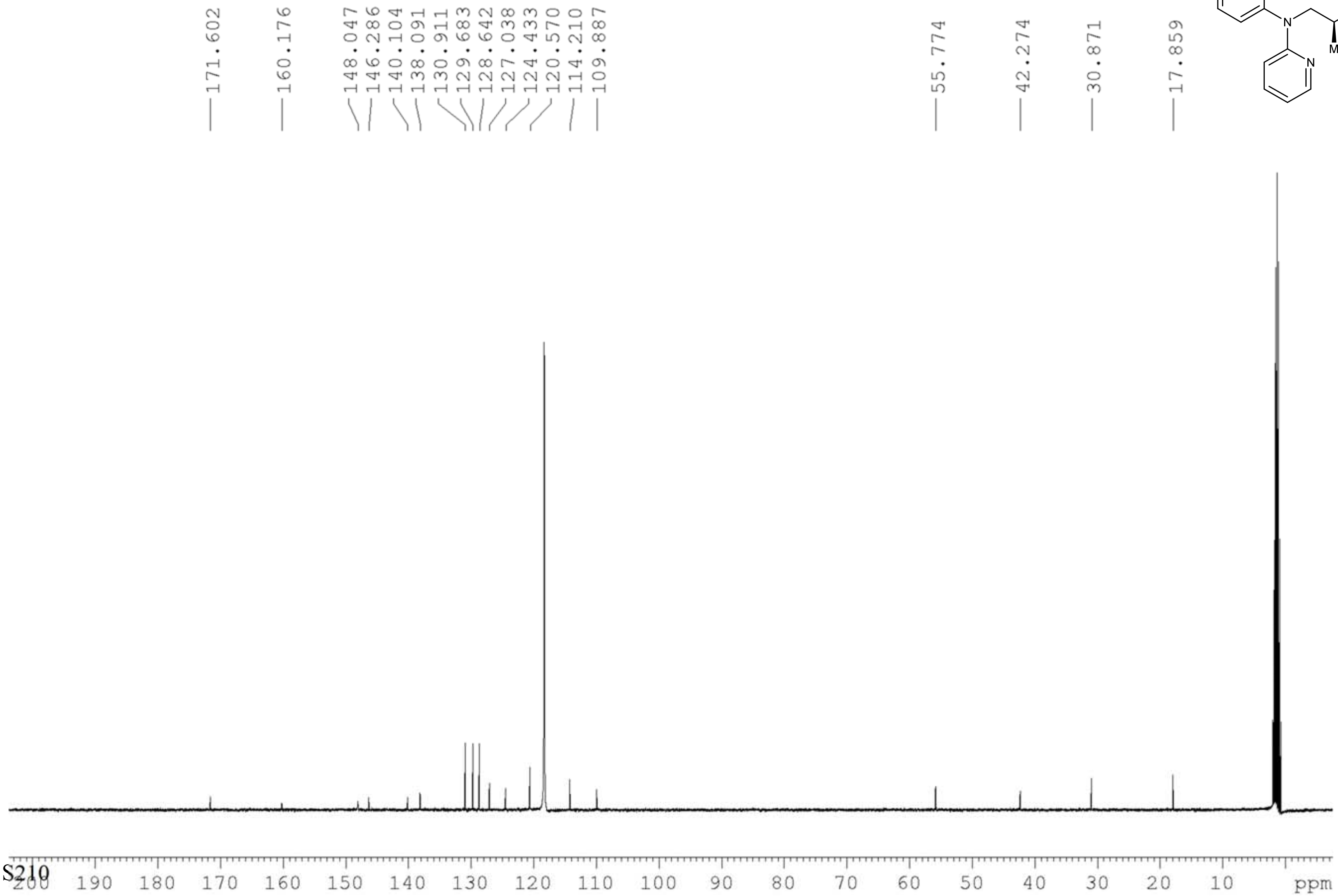

^{1}H NMR (700 MHz, $CDCl_3$) for **(*S*)-4-Methyl-*N*-phenyl-3-((phenyl(pyridin-2-yl)amino)methyl)pentanamide (6c)**

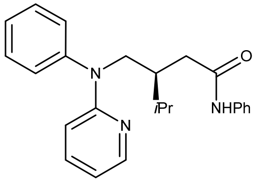

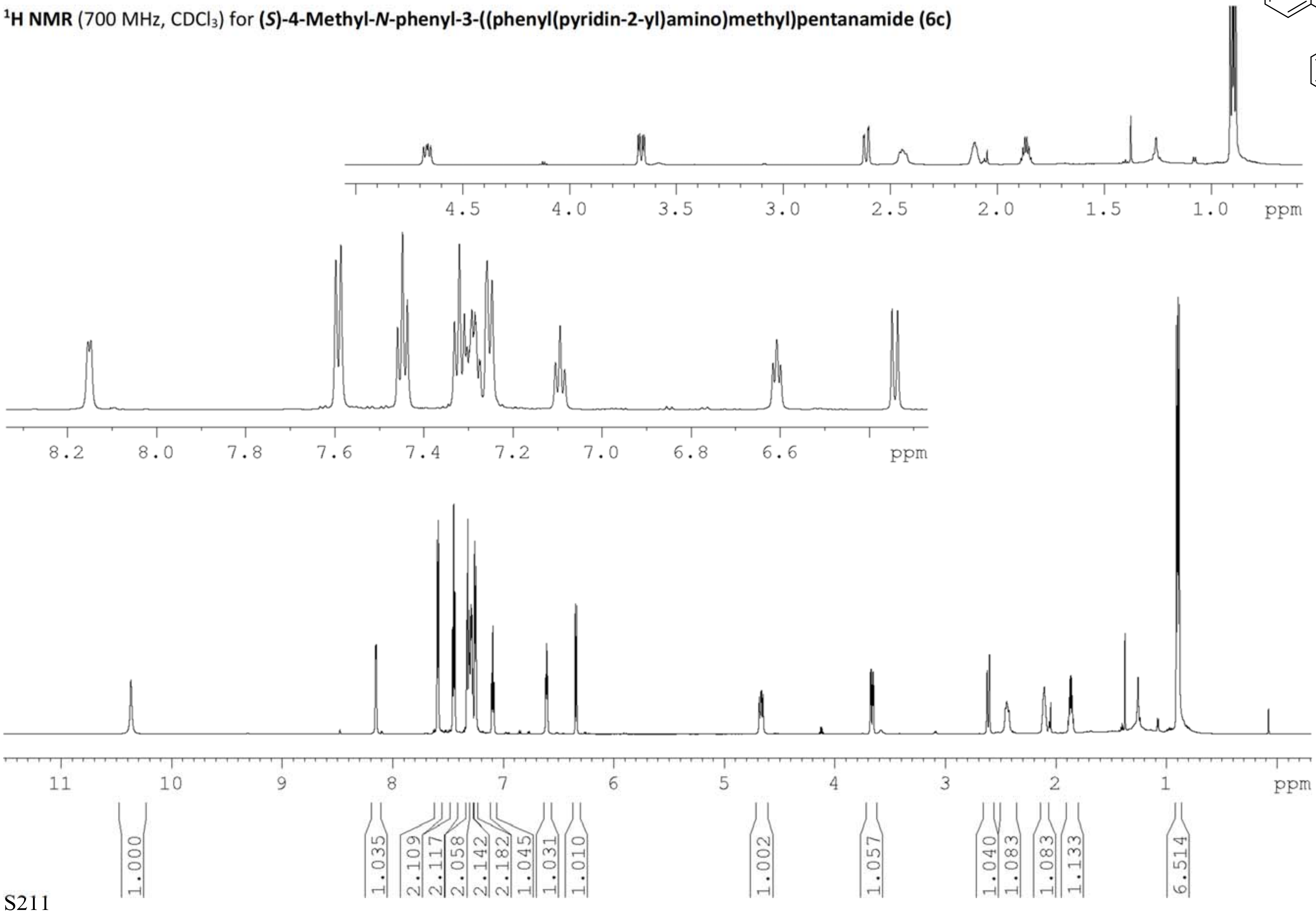

^{13}C NMR (176 MHz, $CDCl_3$) for (*S*)-4-Methyl-*N*-phenyl-3-((phenyl(pyridin-2-yl)amino)methyl)pentanamide (6c)

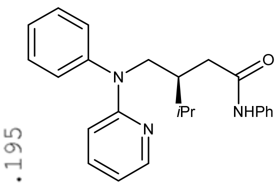

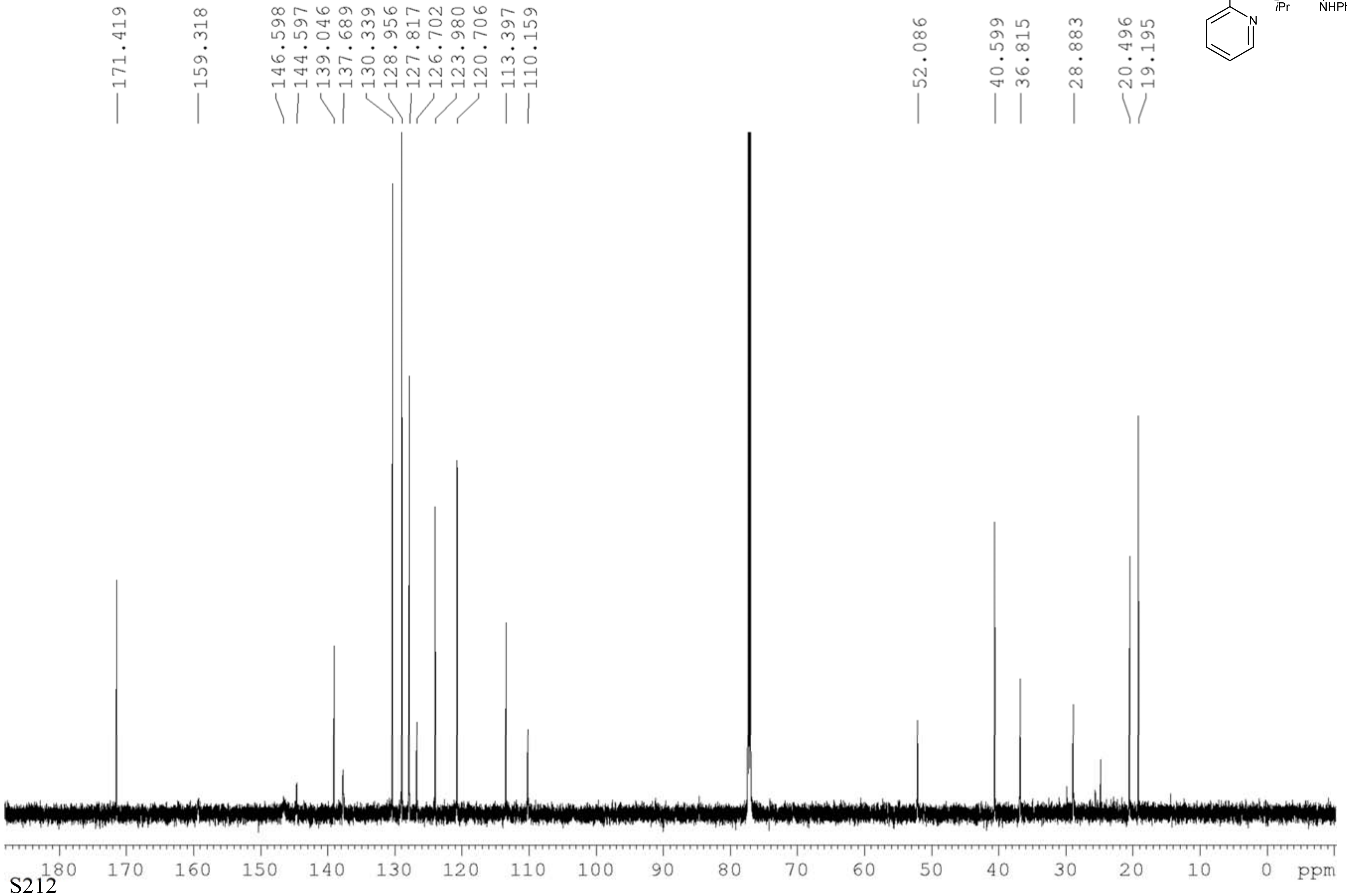

^{1}H NMR (500 MHz, $CDCl_3$) for **(*R*)-3-Methyl-*N*-phenyl-4-(pyridin-2-yl(4-(trifluoromethyl)phenyl)amino)butanamide (6d)**

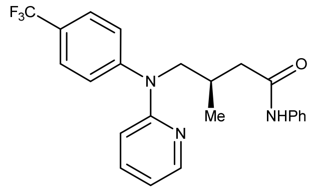

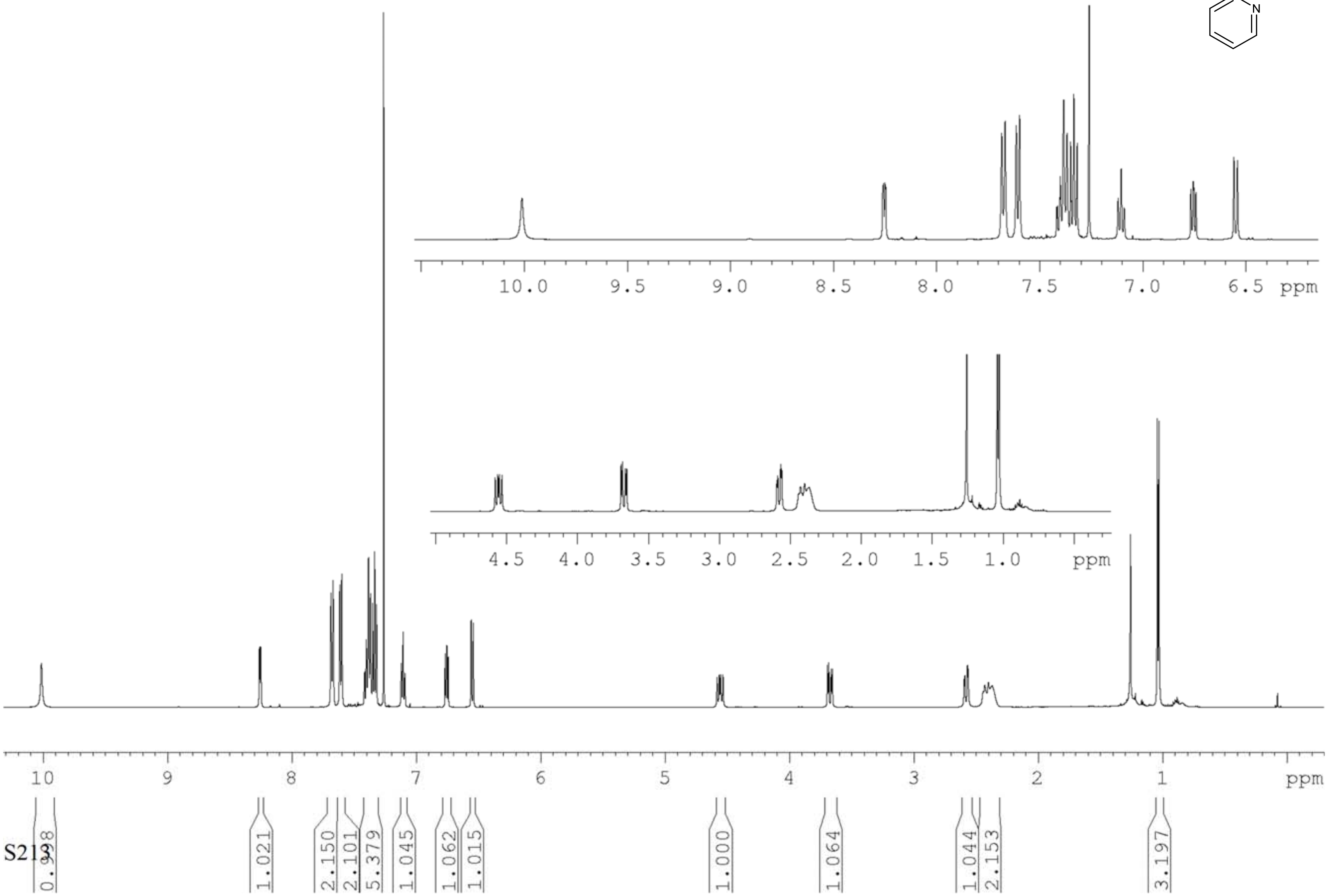

^{13}C NMR (126 MHz, $CDCl_3$) for **(*R*)-3-Methyl-*N*-phenyl-4-(pyridin-2-yl(4-(trifluoromethyl)phenyl)amino)butanamide (6d)**

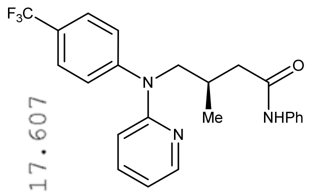

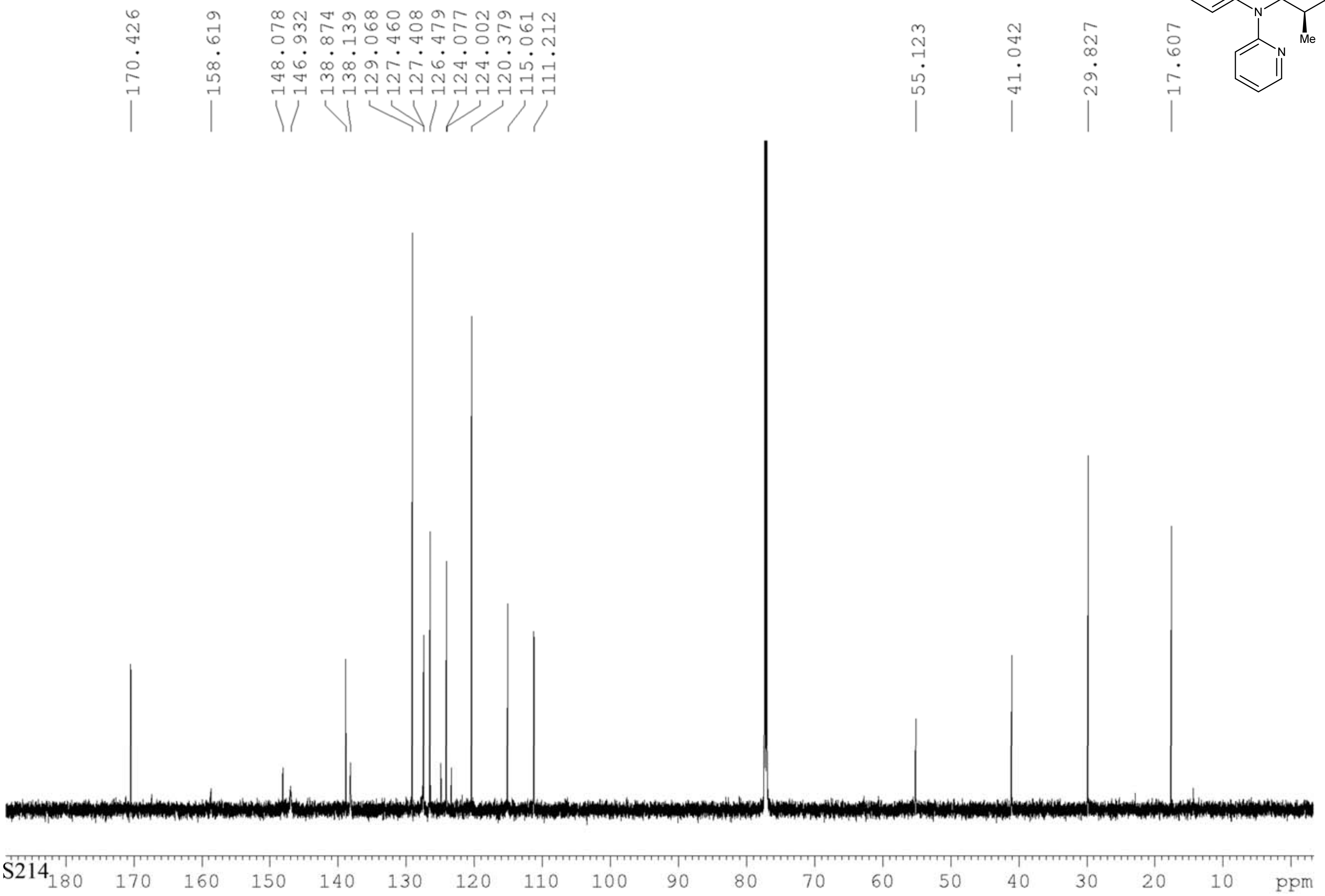

^{19}F NMR (471 MHz, $CDCl_3$) for ***(R)*-3-Methyl-*N*-phenyl-4-(pyridin-2-yl(4-(trifluoromethyl)phenyl)amino)butanamide (6d)**

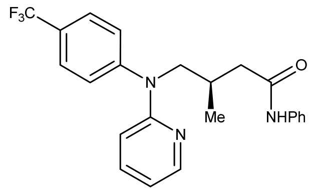

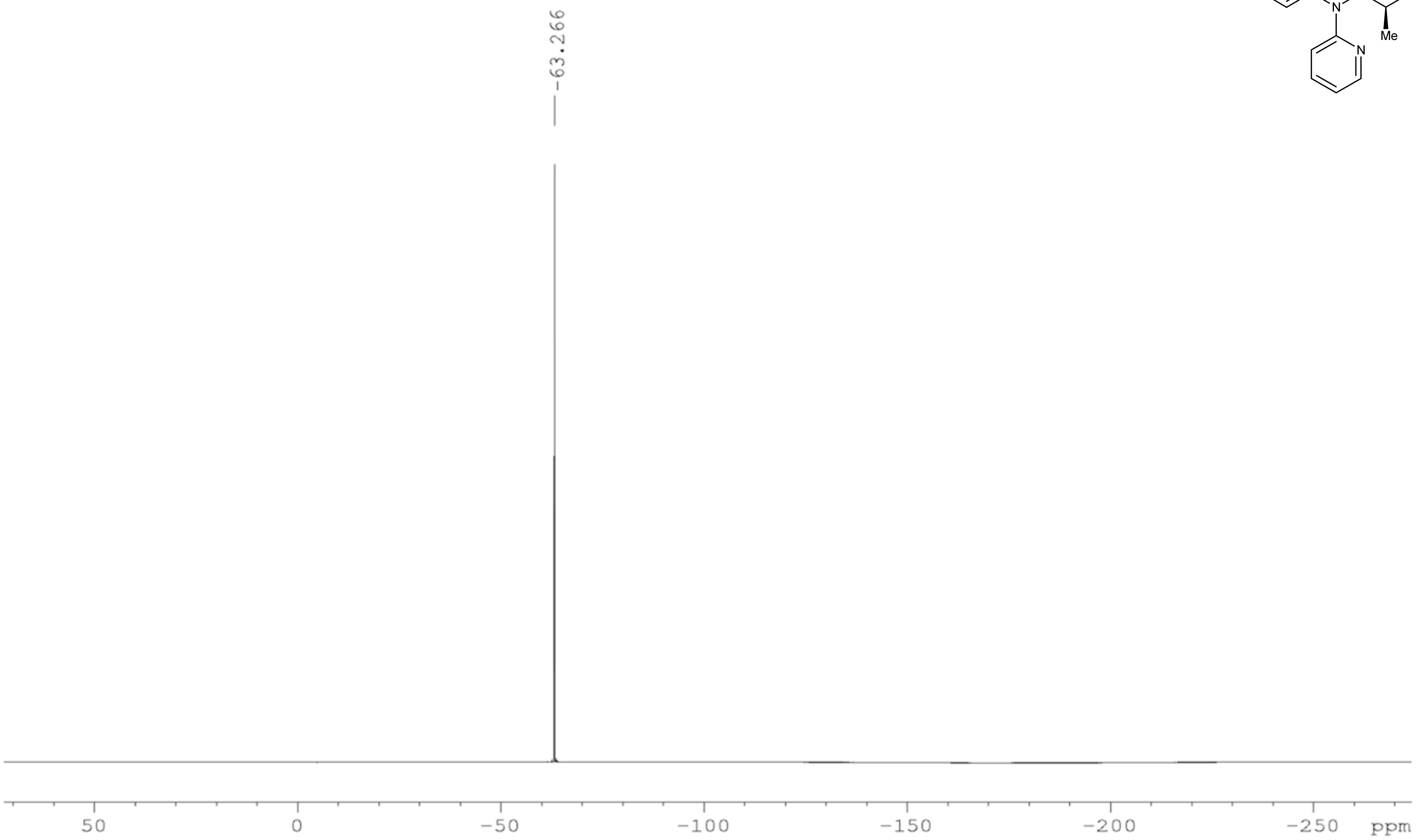

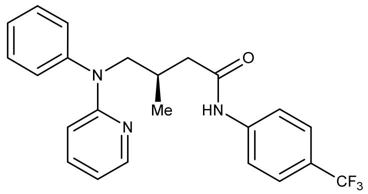

^{1}**H NMR** (400 MHz, $CDCl_3$) for **(*R*)-3-Methyl-4-(phenyl(pyridin-2-yl)amino)-*N*-(4-(trifluoromethyl)phenyl)butanamide (6e)**

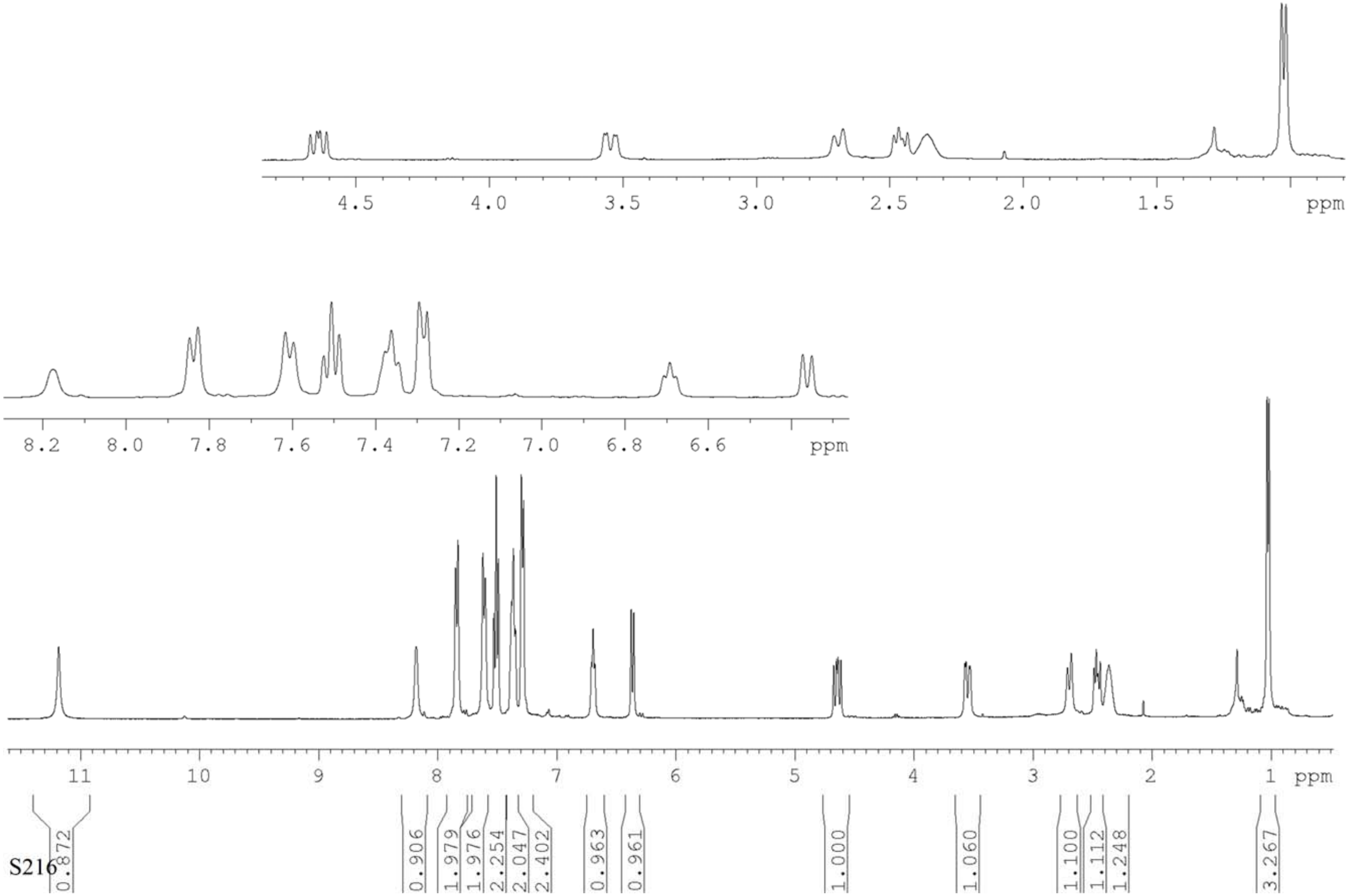

^{13}C NMR (126 MHz, $CDCl_3$) for **(*R*)-3-Methyl-4-(phenyl(pyridin-2-yl)amino)-*N*-(4-(trifluoromethyl)phenyl)butanamide (6e)**

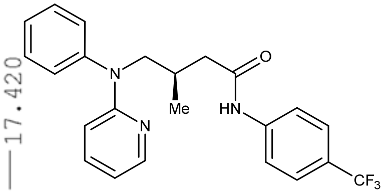

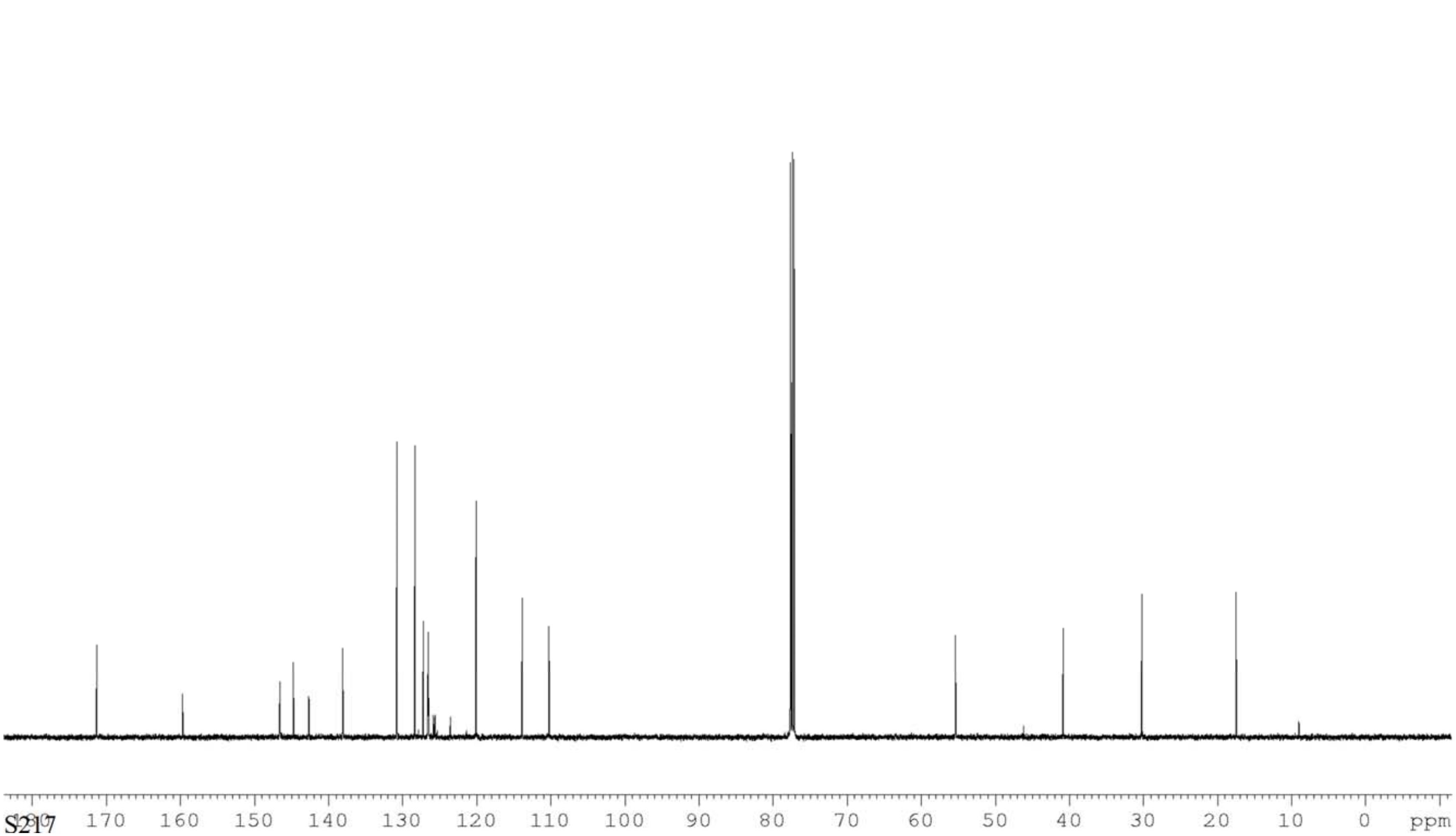

^{19}F NMR (376 MHz, $CHCl_3$) for **(*R*)-3-Methyl-4-(phenyl(pyridin-2-yl)amino)-*N*-(4-(trifluoromethyl)phenyl)butanamide (6e)**

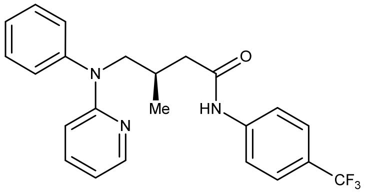

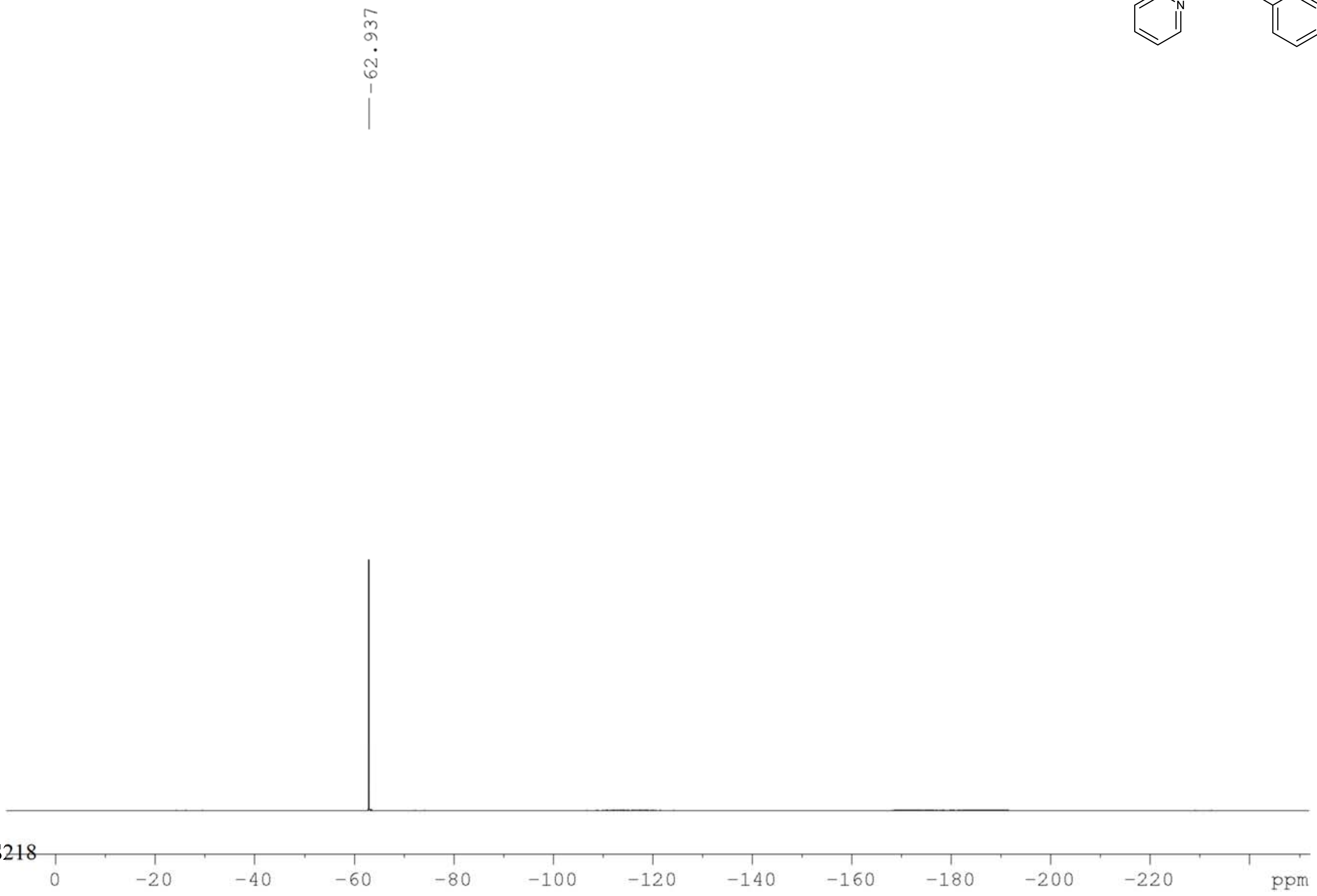

¹H NMR (500 MHz, $CDCl_3$) for **(*R*)-4-(benzyl(pyridin-2-yl)amino)-3-methyl-*N*-phenylbutanamide (6f)**

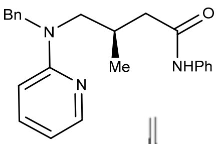

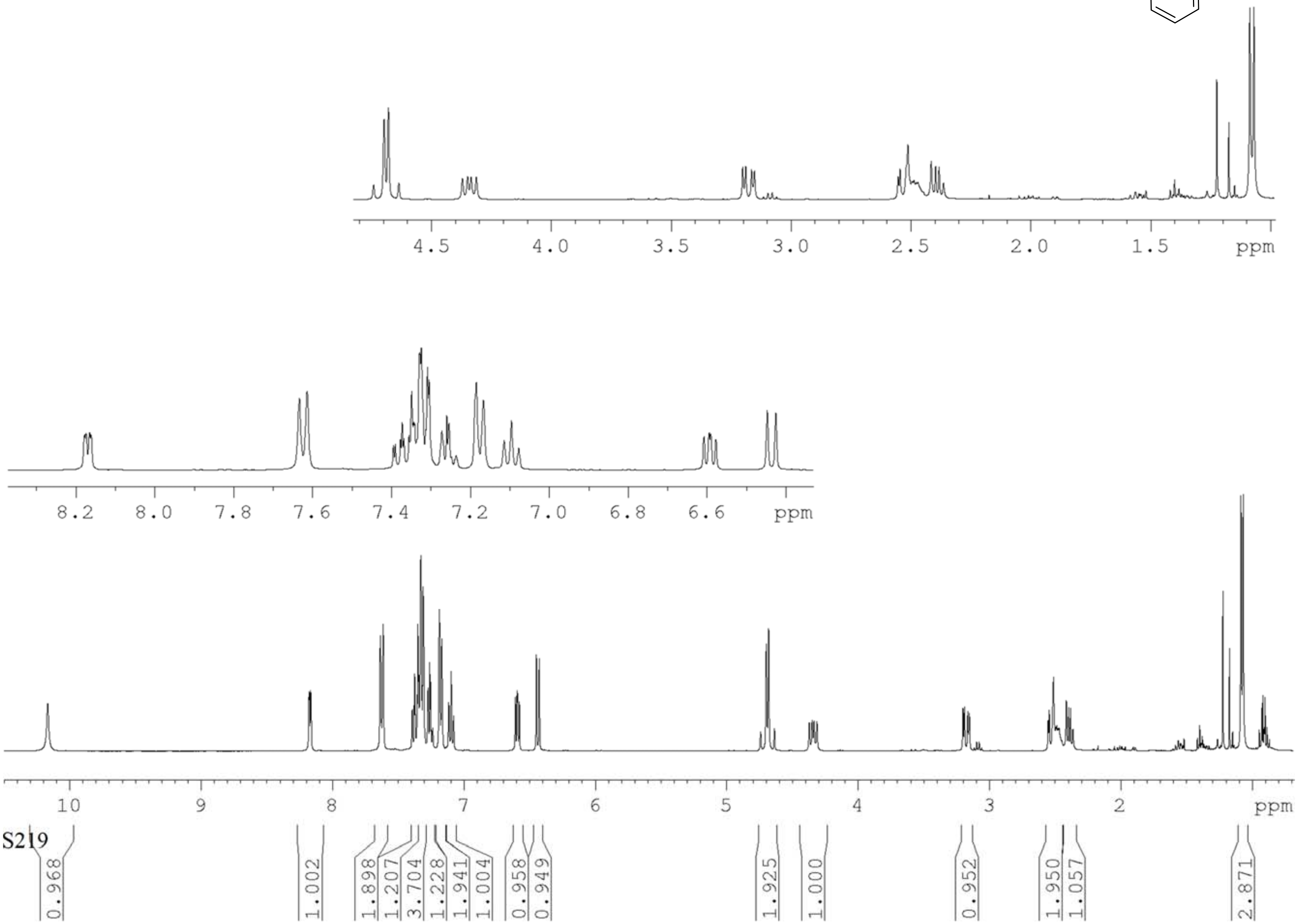

^{13}C NMR (101 MHz, $CDCl_3$) for **(*R*)-4-(benzyl(pyridin-2-yl)amino)-3-methyl-*N*-phenylbutanamide (6f)**

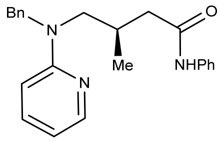

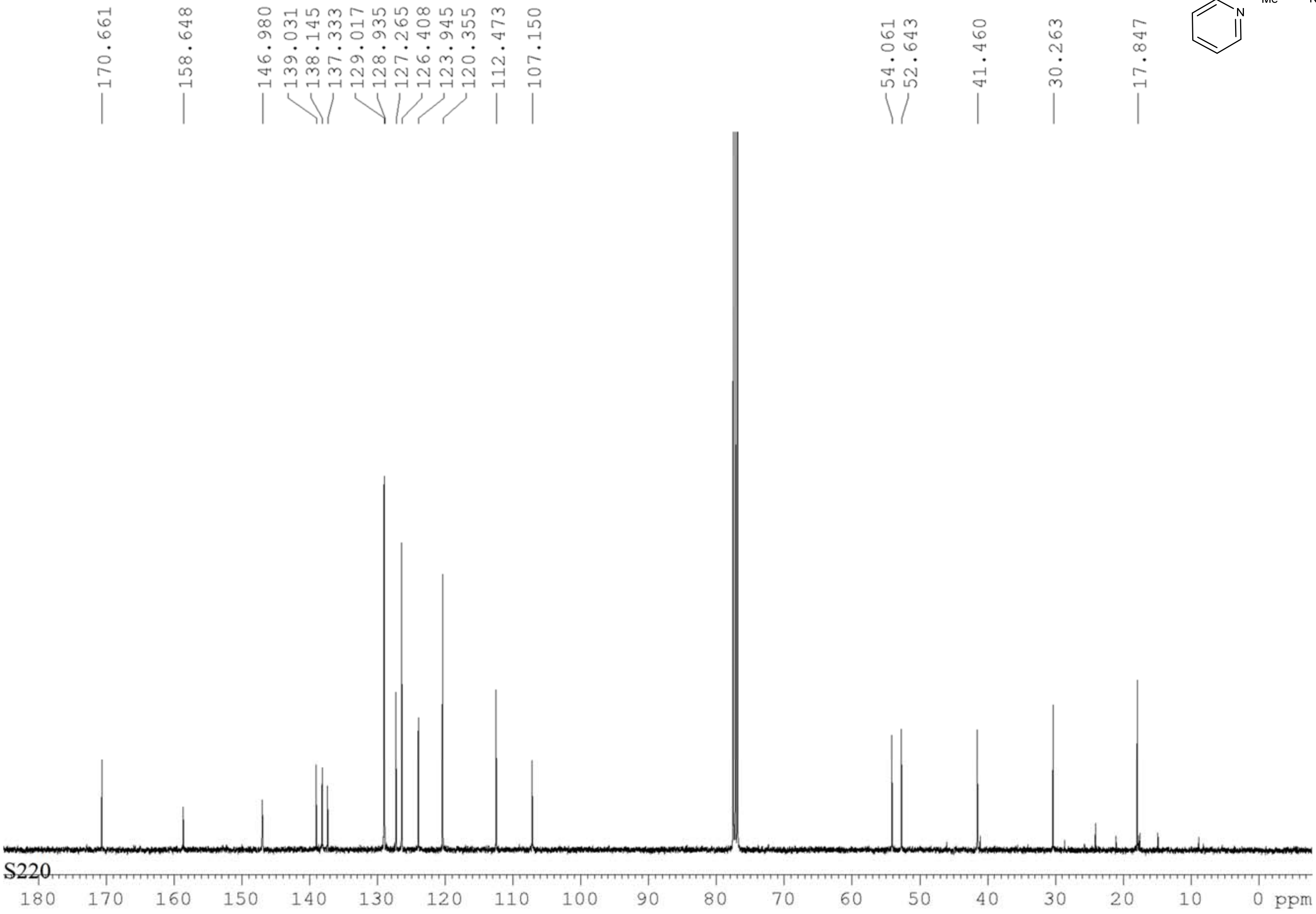

¹H NMR (500 MHz, $CDCl_3$) for ***(R)*-3-((Benzyl(pyridin-2-yl)amino)methyl)-*N*,5-diphenylpentanamide (6g)**

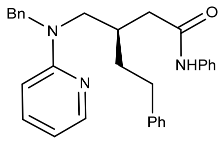

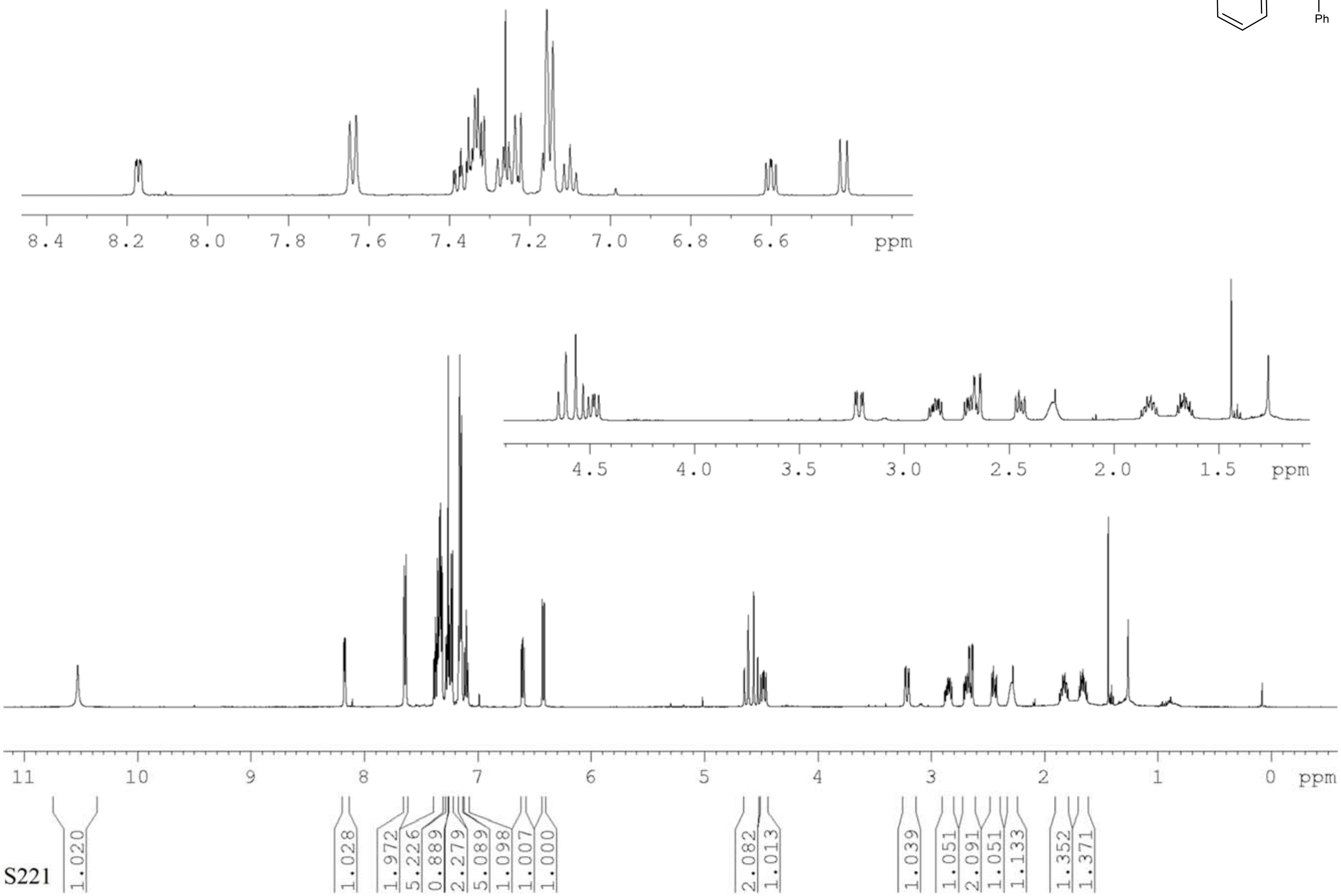

^{13}C NMR (126 MHz, $CDCl_3$) for **(*R*)-3-((Benzyl(pyridin-2-yl)amino)methyl)-*N*,5-diphenylpentanamide (6g)**

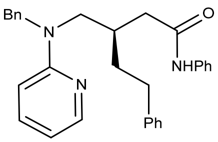

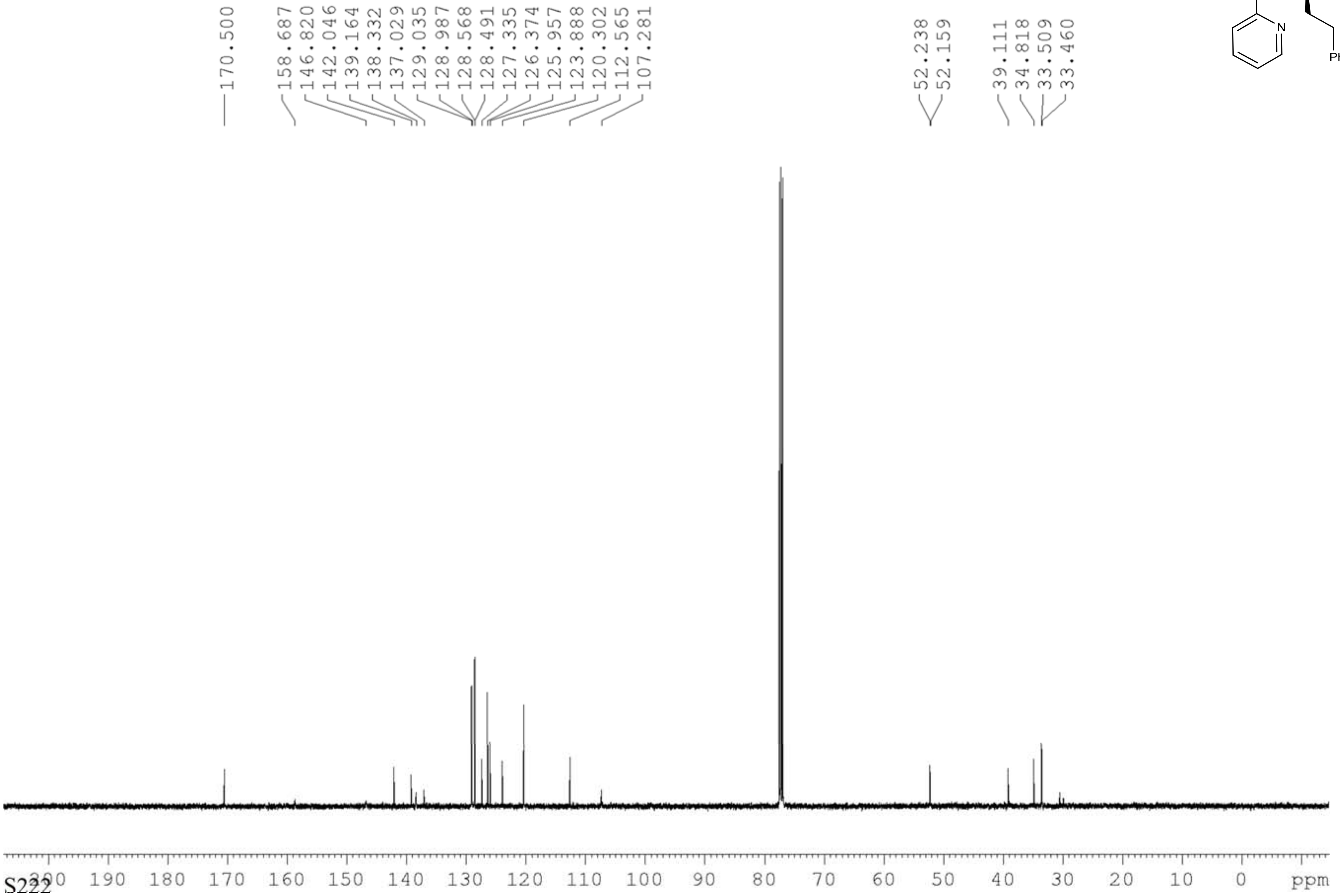

^{1}H NMR (500 MHz, $CDCl_3$) for (***S***)**-3-(((2-((tert-butyldimethylsilyl)oxy)ethyl)(pyridin-2-yl)amino)methyl)-4-methyl-*N*-phenylpentanamide (6h)**

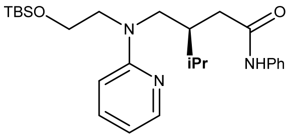

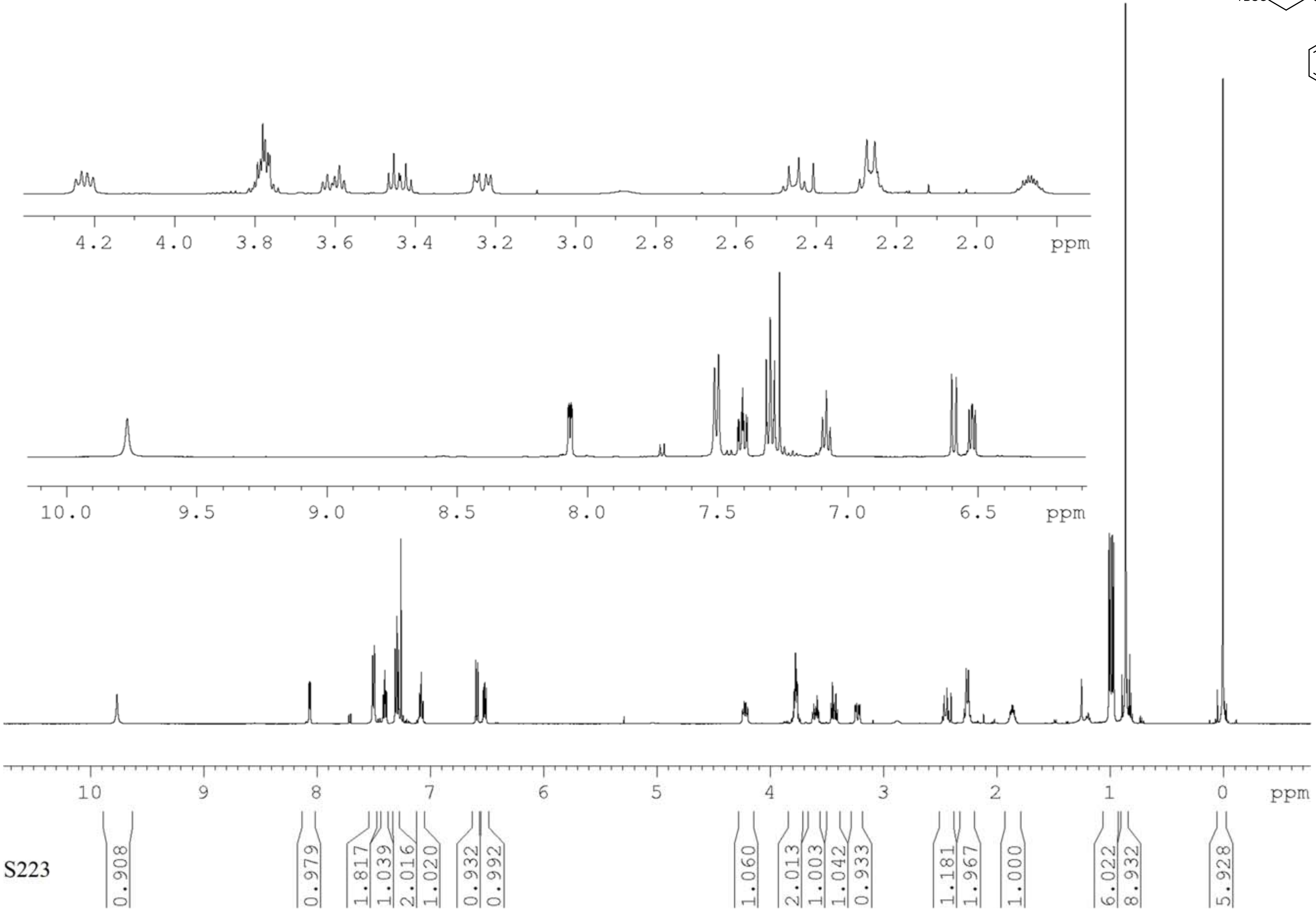

^{13}C NMR (126 MHz, $CDCl_3$) for **(*S*)-3-(((2-((tert-butyldimethylsilyl)oxy)ethyl)(pyridin-2-yl)amino)methyl)-4-methyl-*N*-phenylpentanamide (6h)**

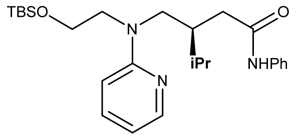

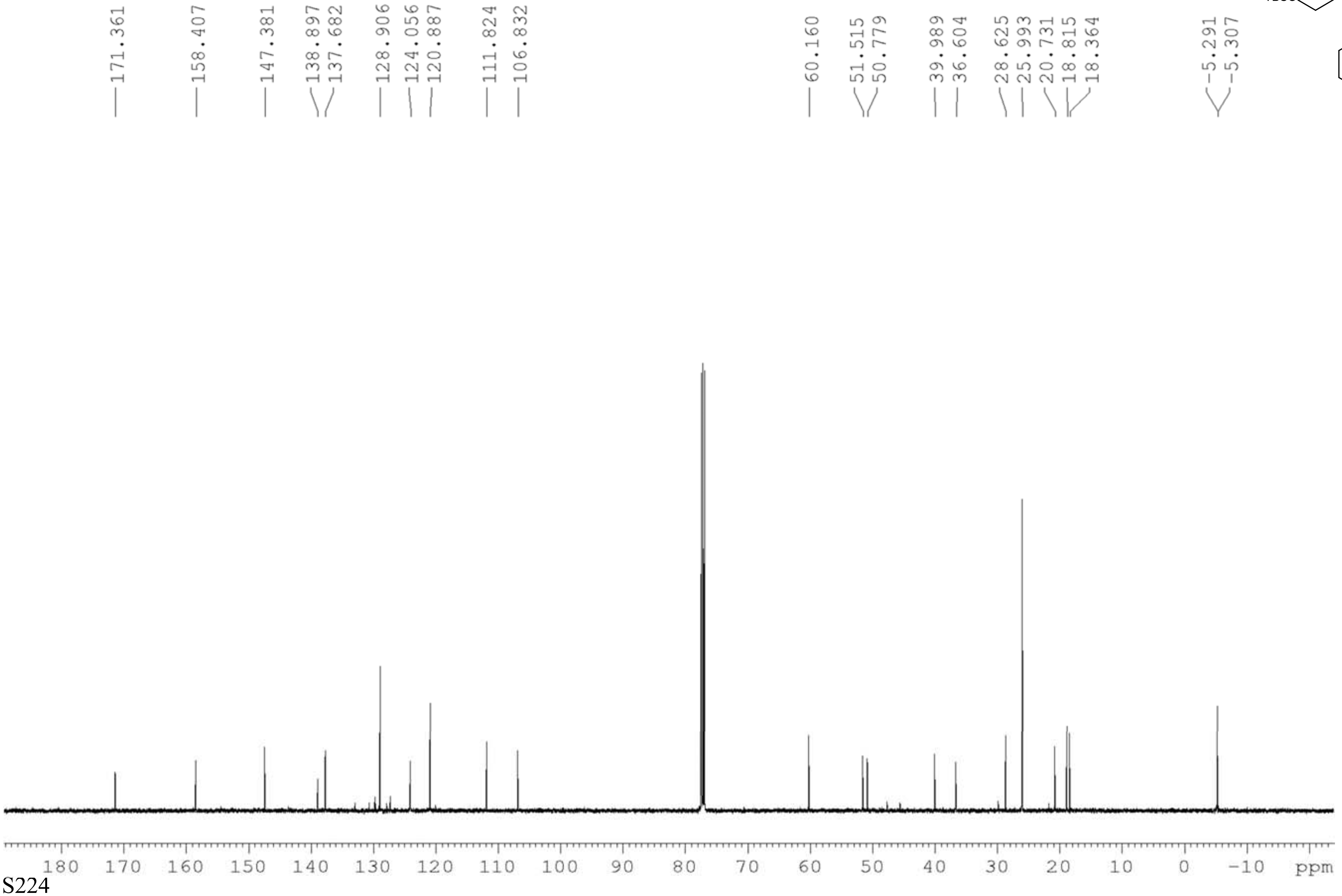

^{1}H NMR (400 MHz, $CDCl_3$) for **2,3-Dimethyl-4-(methyl(pyridin-2-yl)amino)-*N*-phenylbutanamide (6ia)**

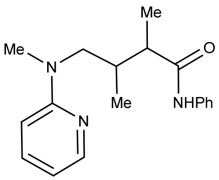

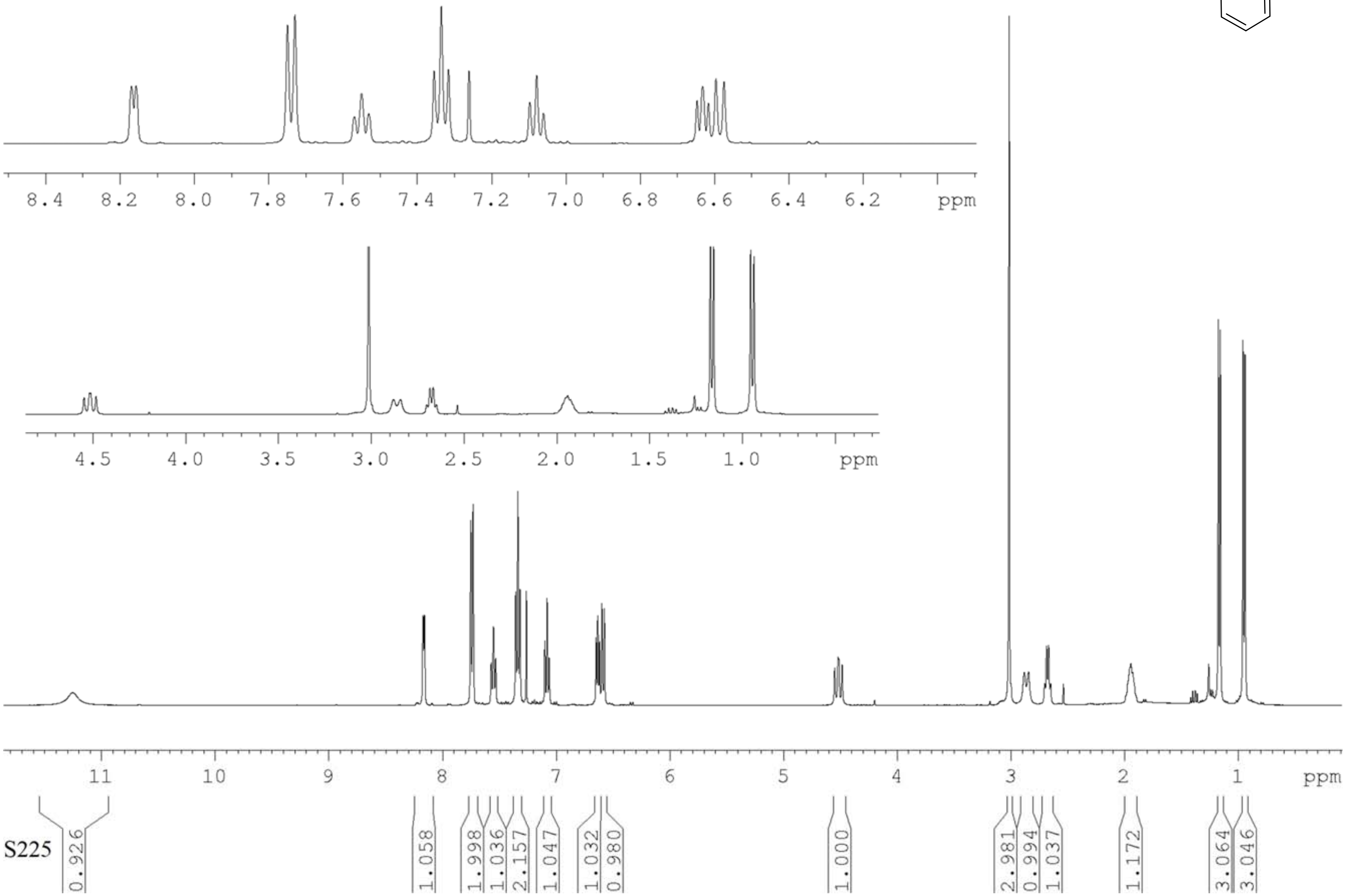

^{13}C NMR (101 MHz, $CDCl_3$) for **2,3-Dimethyl-4-(methyl(pyridin-2-yl)amino)-*N*-phenylbutanamide (6ia)**

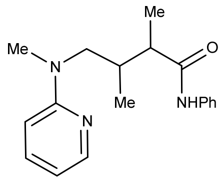

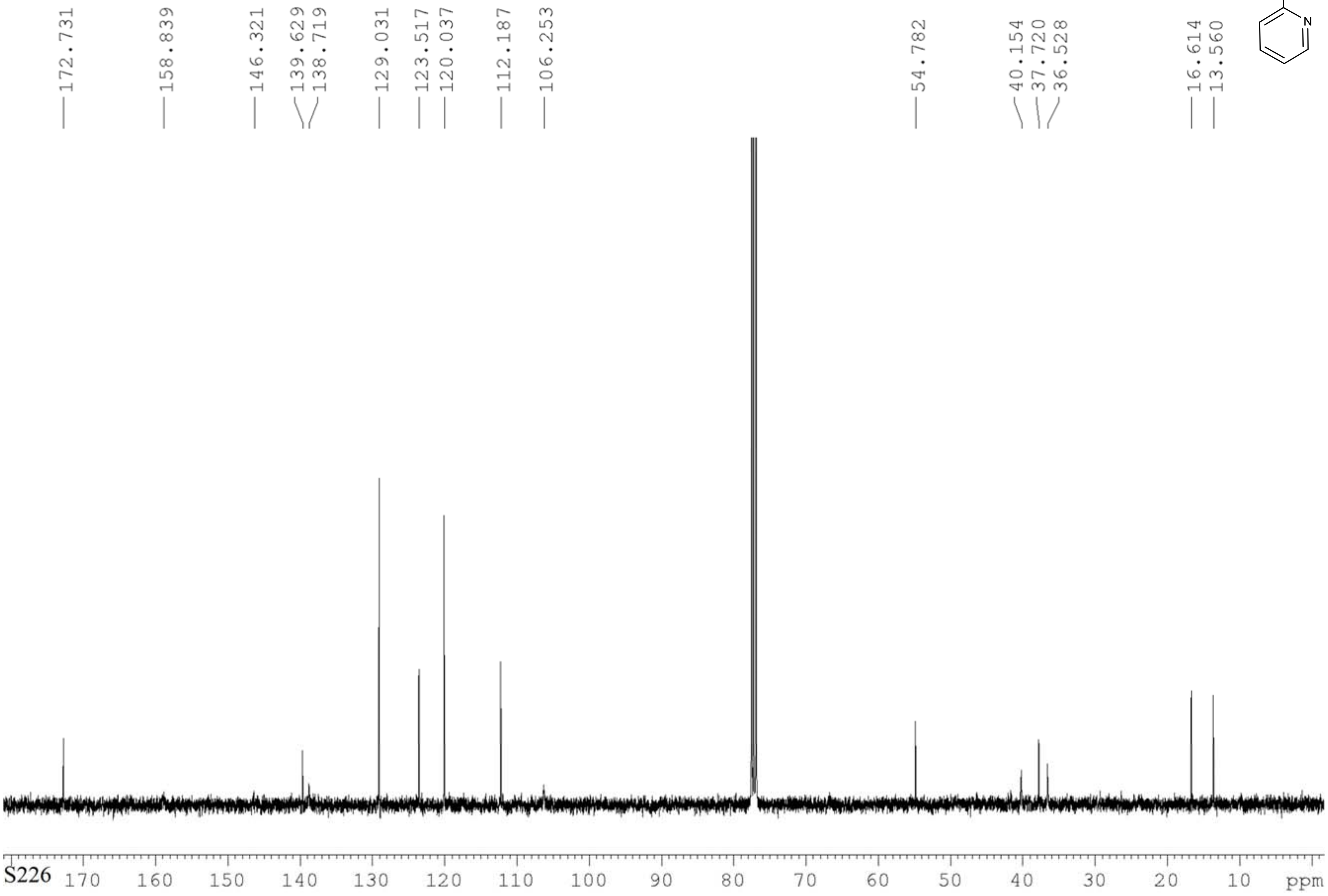

^{1}**H NMR** (400 MHz, $CDCl_3$) for **2,3-Dimethyl-4-(methyl(pyridin-2-yl)amino)-*N*-phenylbutanamide (6ib)**

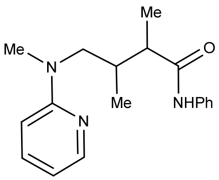

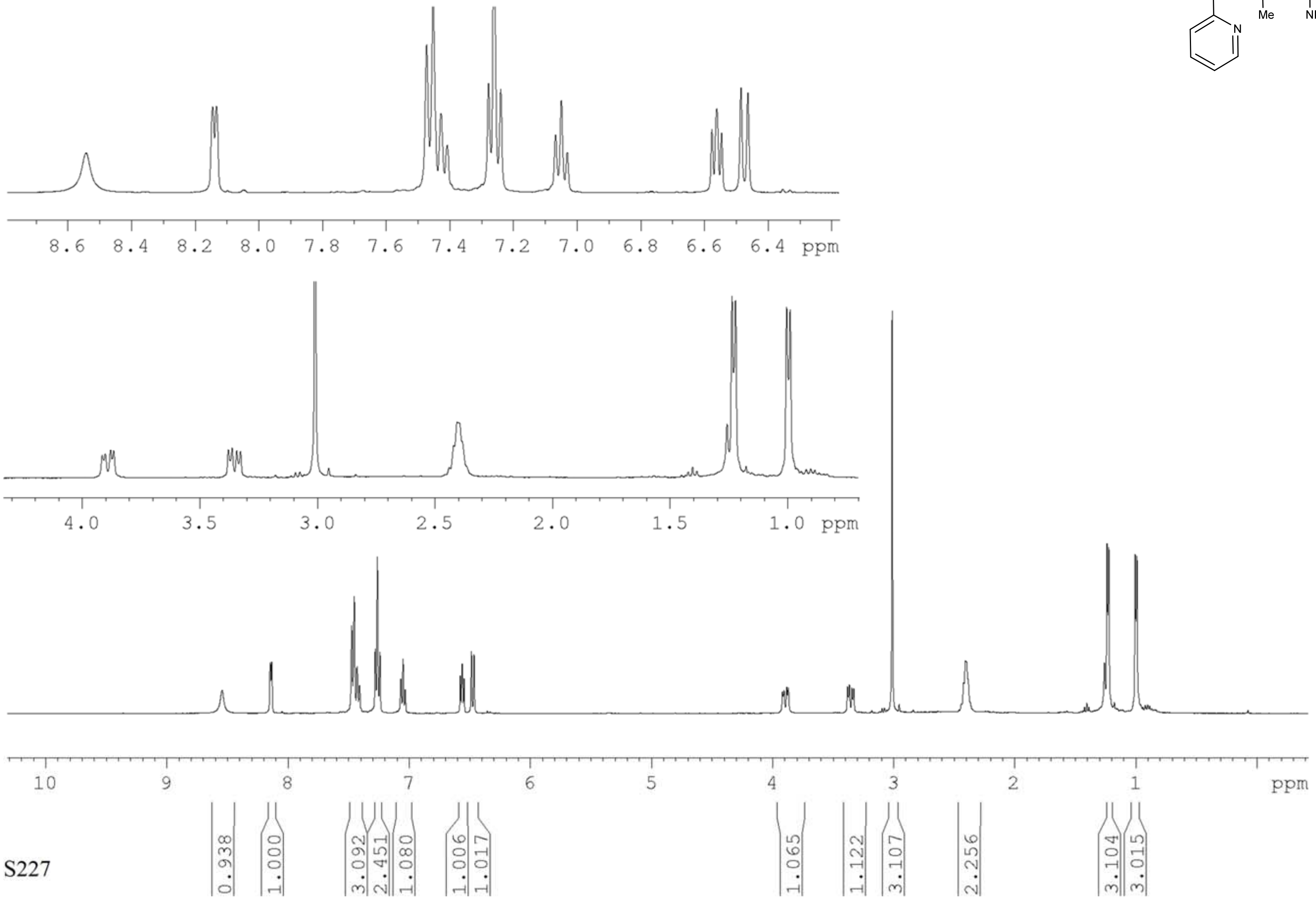

^{13}C NMR (101 MHz, $CDCl_3$) for **2,3-Dimethyl-4-(methyl(pyridin-2-yl)amino)-*N*-phenylbutanamide (6ib)**

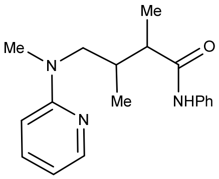

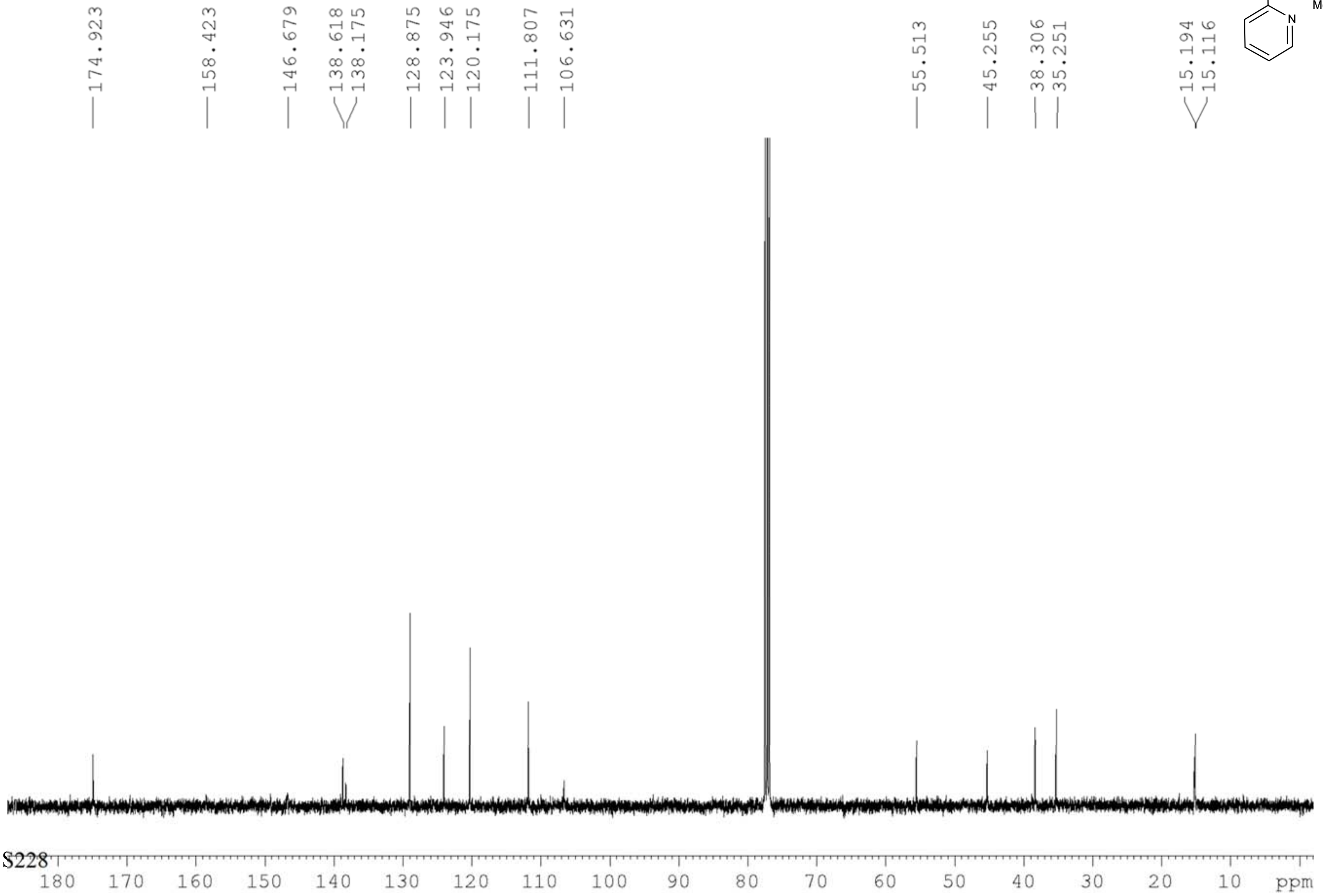

¹H NMR (500 MHz, $CDCl_3$) for **2-((Methyl(pyridin-2-yl)amino)methyl)-*N*-phenylcyclohexane-1-carboxamide (6ja)**

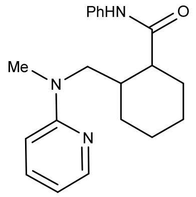

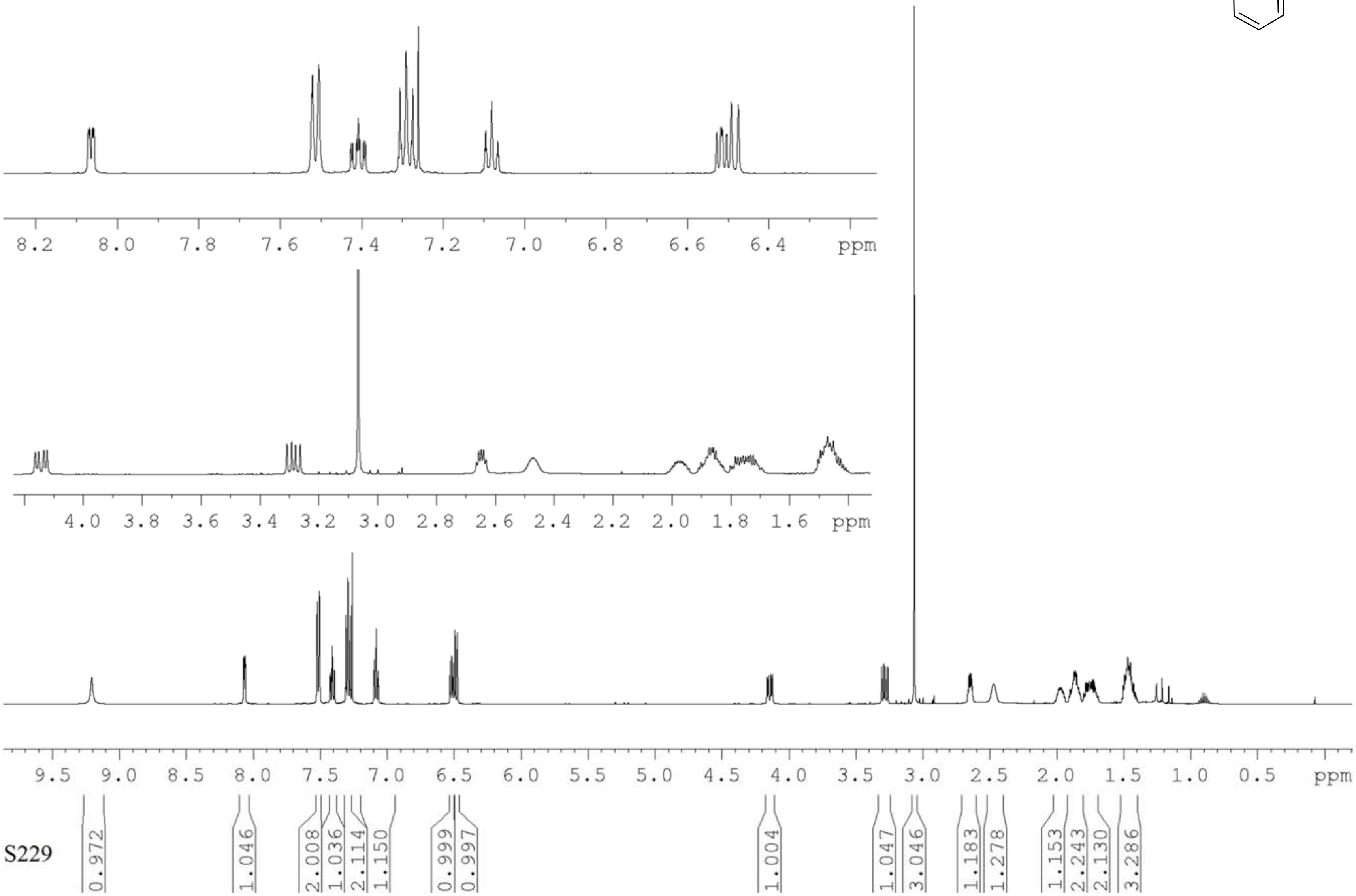

^{13}C NMR (101 MHz, $CDCl_3$) for **2-((Methyl(pyridin-2-yl)amino)methyl)-*N*-phenylcyclohexane-1-carboxamide (6ja)**

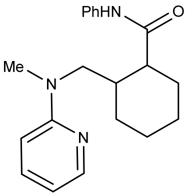

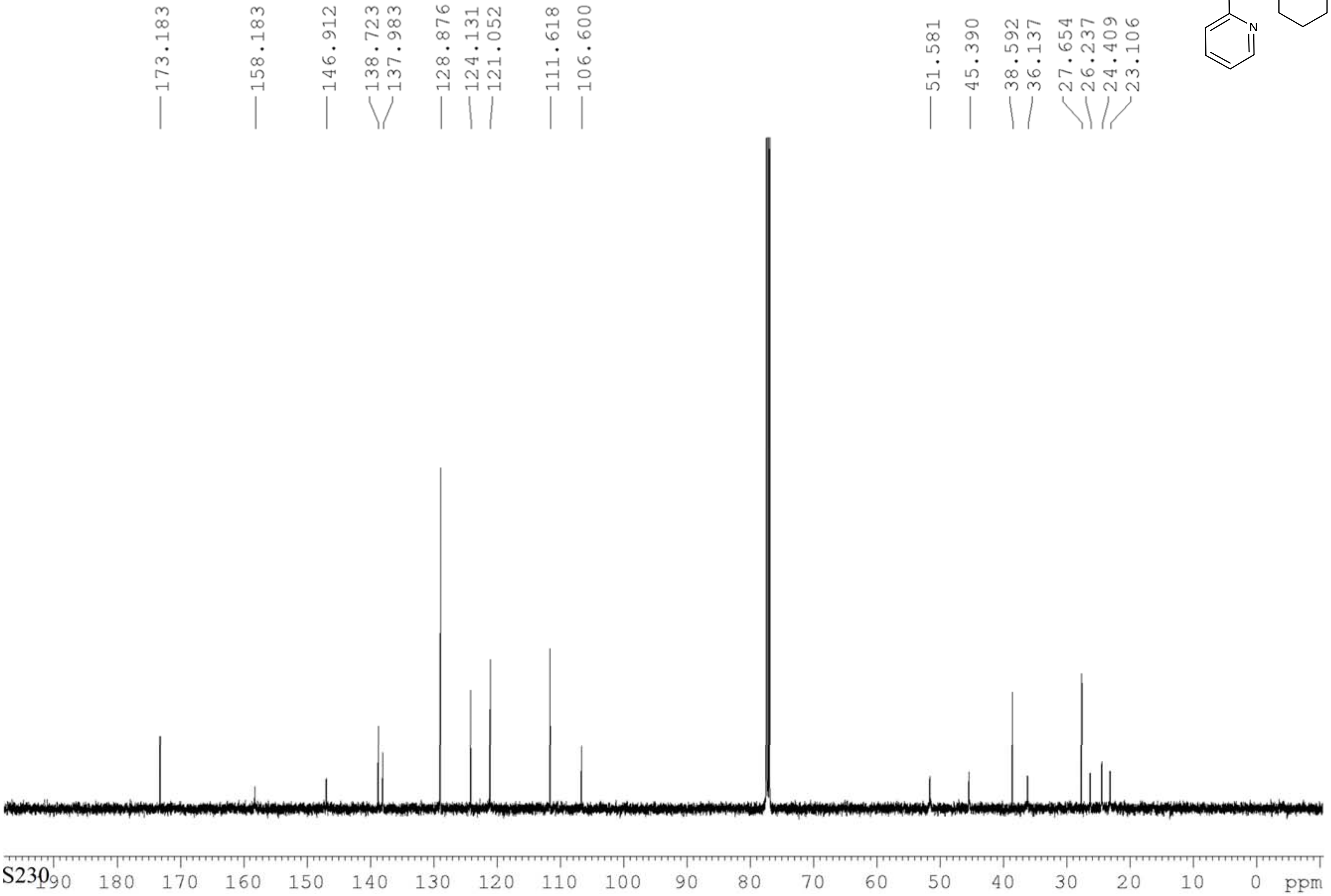

^{1}H NMR (500 MHz, $CDCl_3$) for **2-((Methyl(pyridin-2-yl)amino)methyl)-*N*-phenylcyclohexane-1-carboxamide (6jb)**

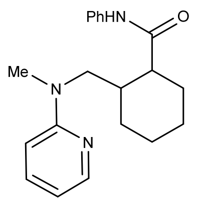

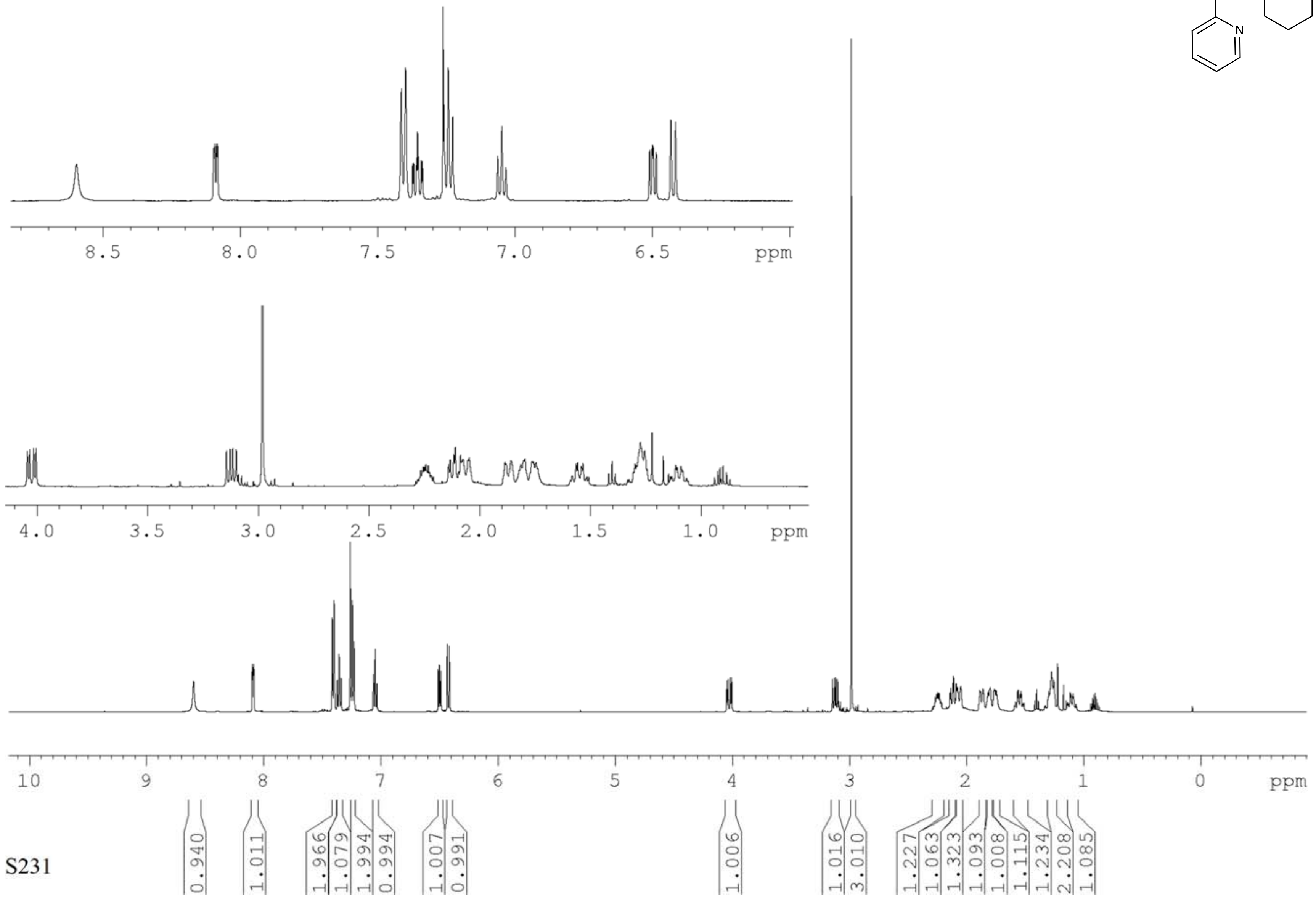

^{13}**C NMR** (101 MHz, $CDCl_3$) for **2-((Methyl(pyridin-2-yl)amino)methyl)-*N*-phenylcyclohexane-1-carboxamide (6jb)**

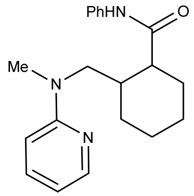

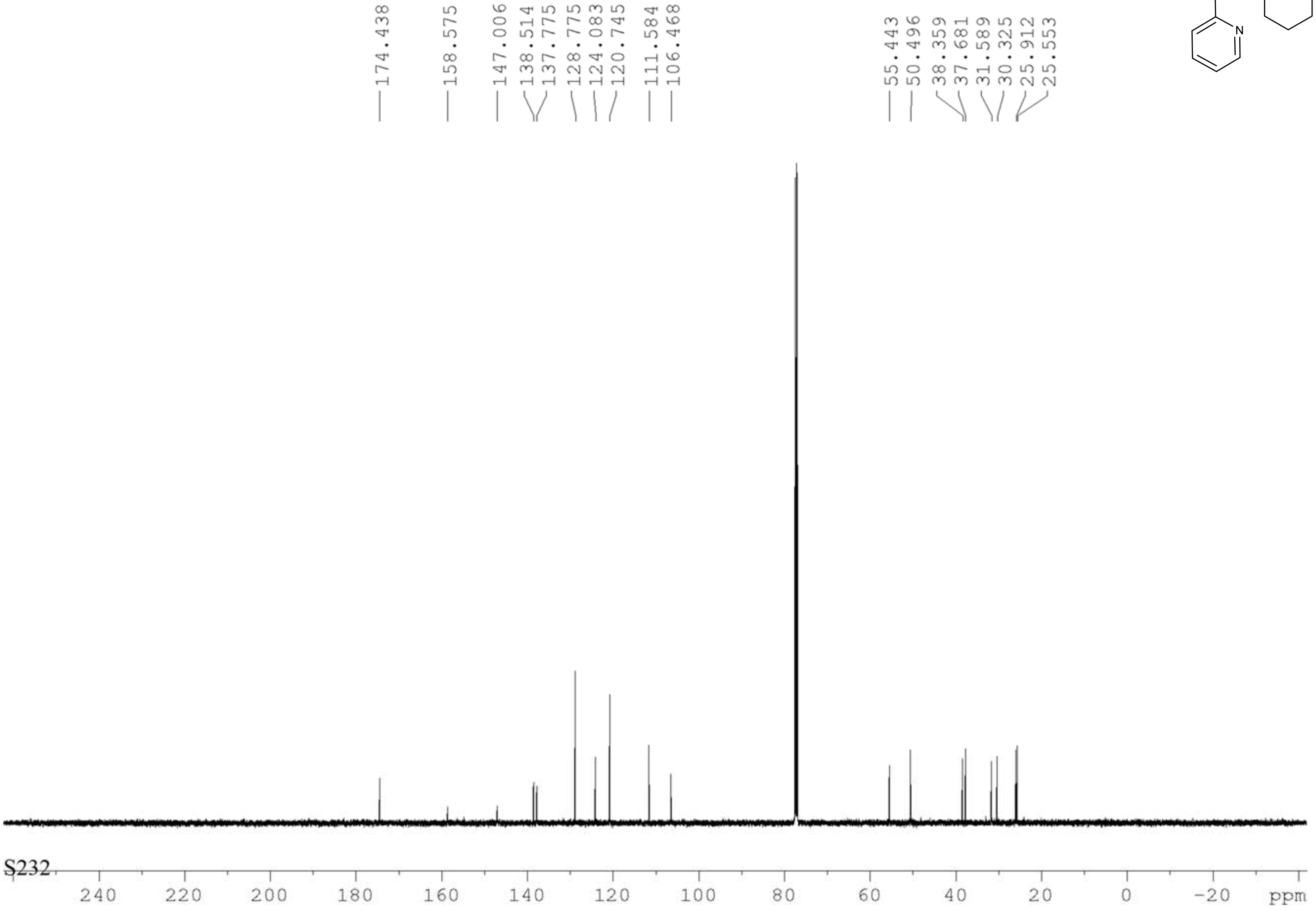

¹H NMR (400 MHz, $CDCl_3$) for **(*R*)-4-Methyl-*N*-phenyl-3-((*S*)-1-(pyridin-2-yl)azetidin-2-yl)pentanamide (6ka)**

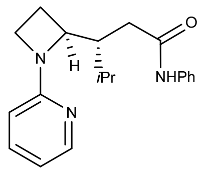

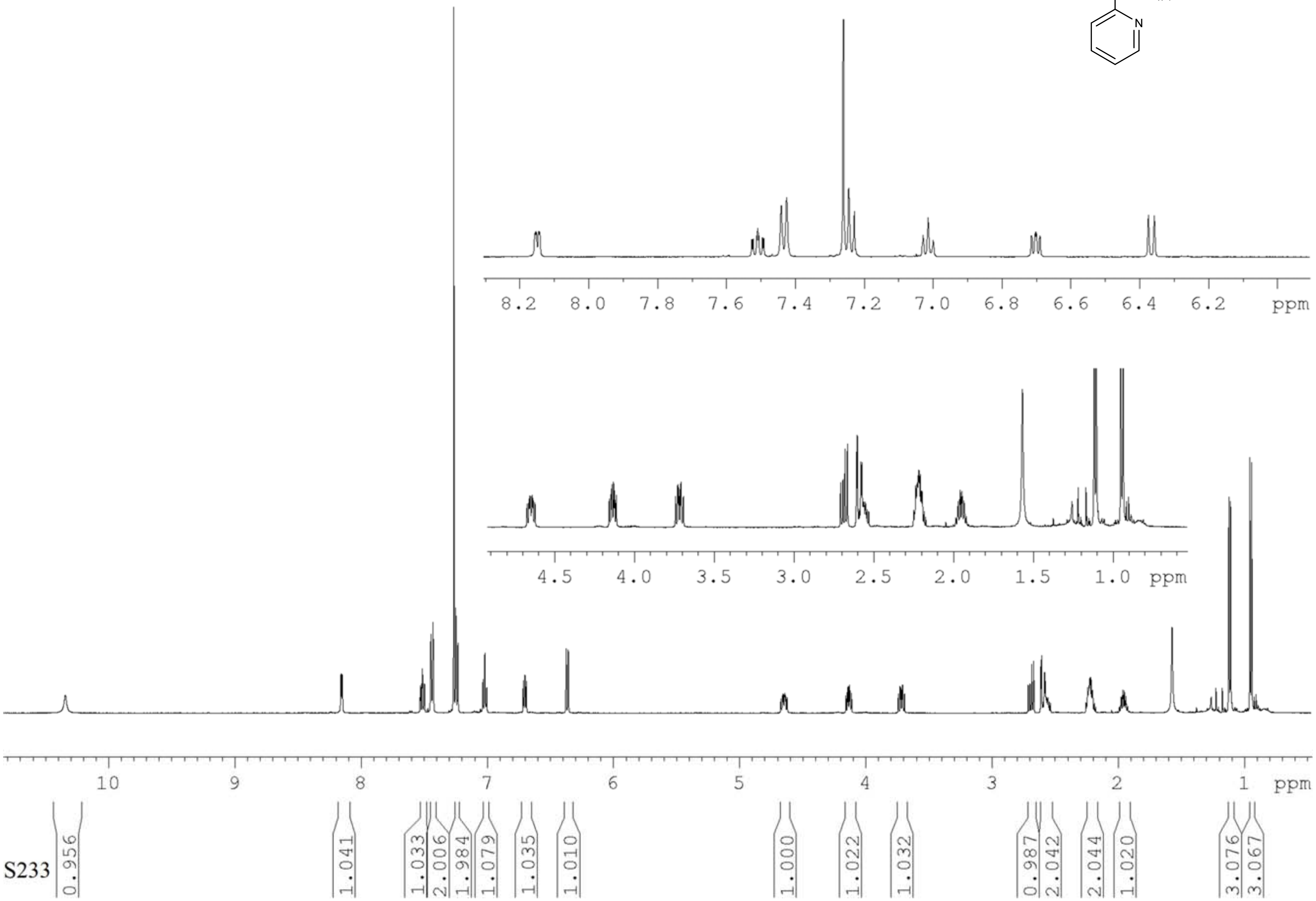

^{13}C NMR (101 MHz, $CDCl_3$) for ***(R)*-4-Methyl-*N*-phenyl-3-((*S*)-1-(pyridin-2-yl)azetidin-2-yl)pentanamide (6ka)**

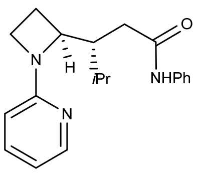

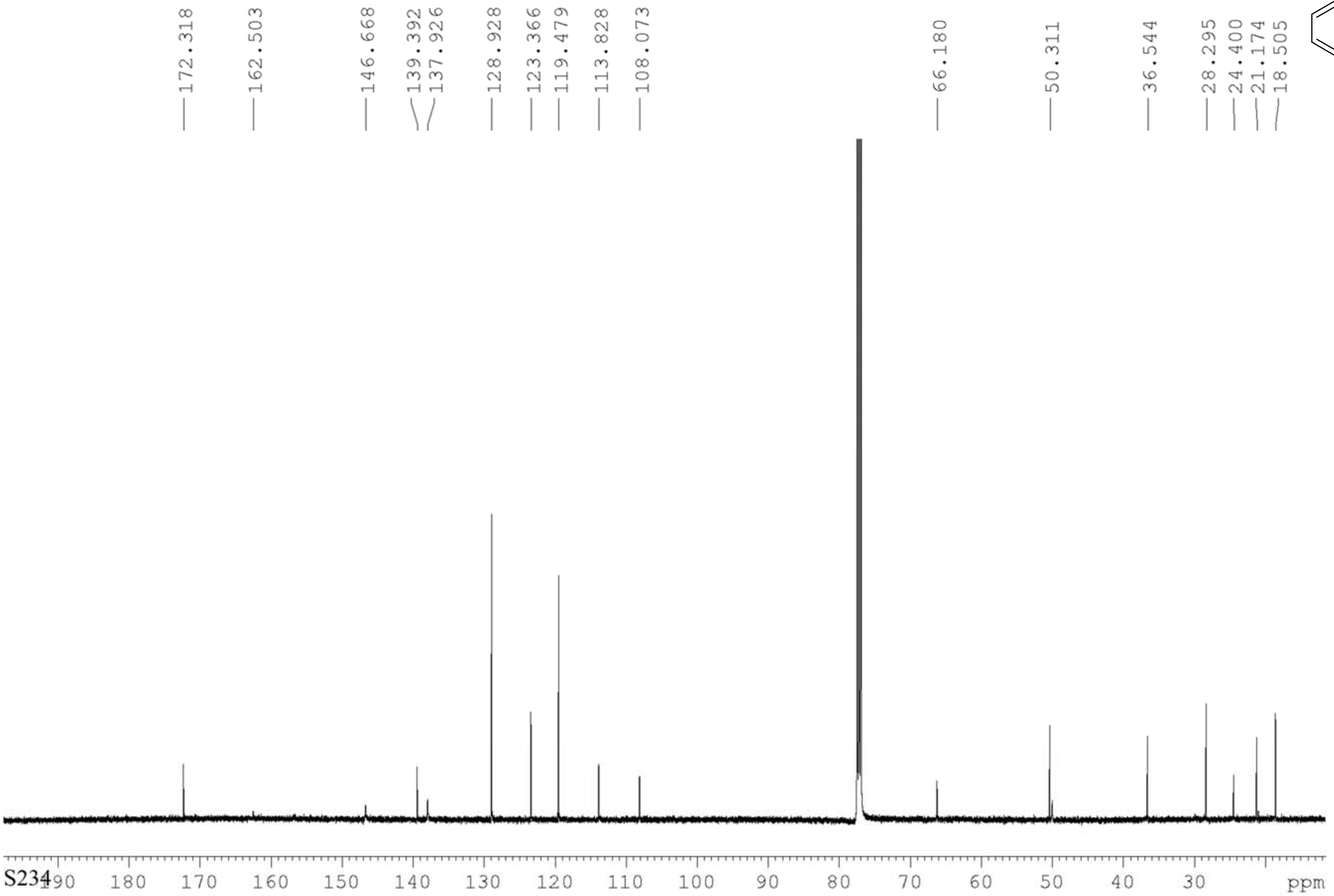

^{1}H NMR (500 MHz, $CDCl_3$) for **(*S*)-4-Methyl-*N*-phenyl-3-((*S*)-1-(pyridin-2-yl)azetidin-2-yl)pentanamide (6kb)**

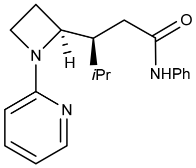

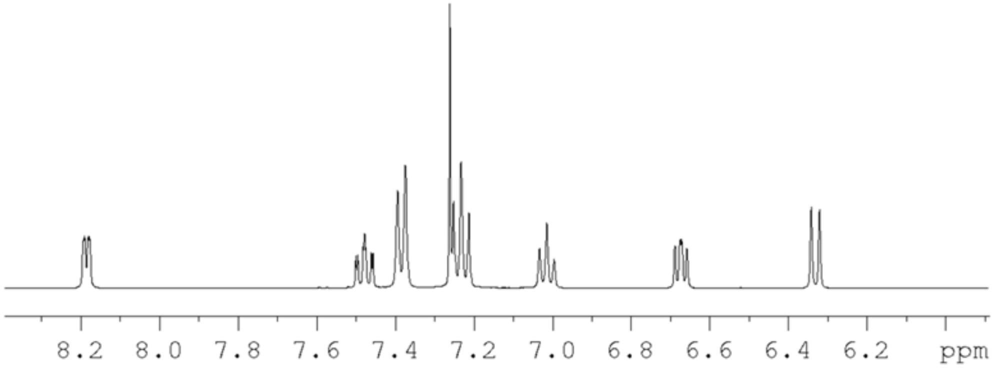

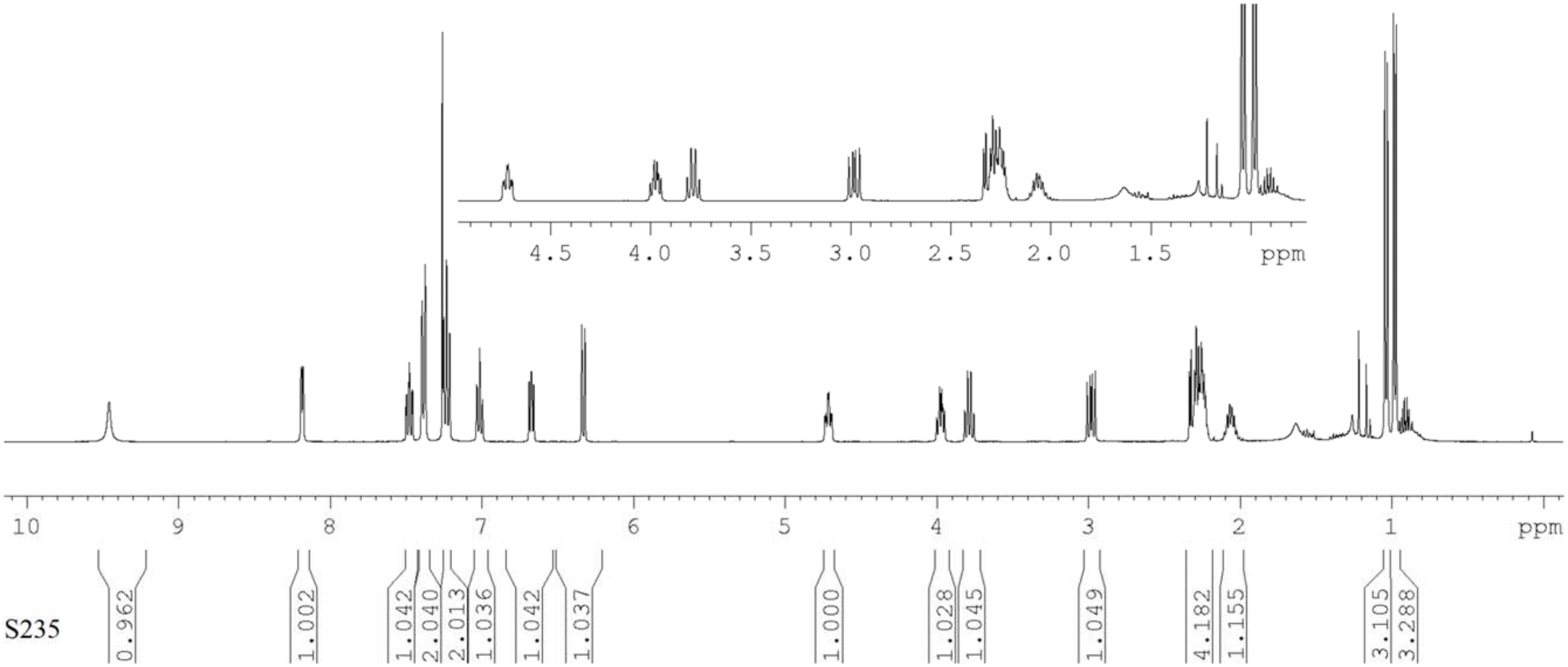

^{13}C NMR (126 MHz, $CDCl_3$) for **(*S*)-4-Methyl-*N*-phenyl-3-((*S*)-1-(pyridin-2-yl)azetidin-2-yl)pentanamide (6kb)**

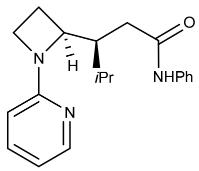

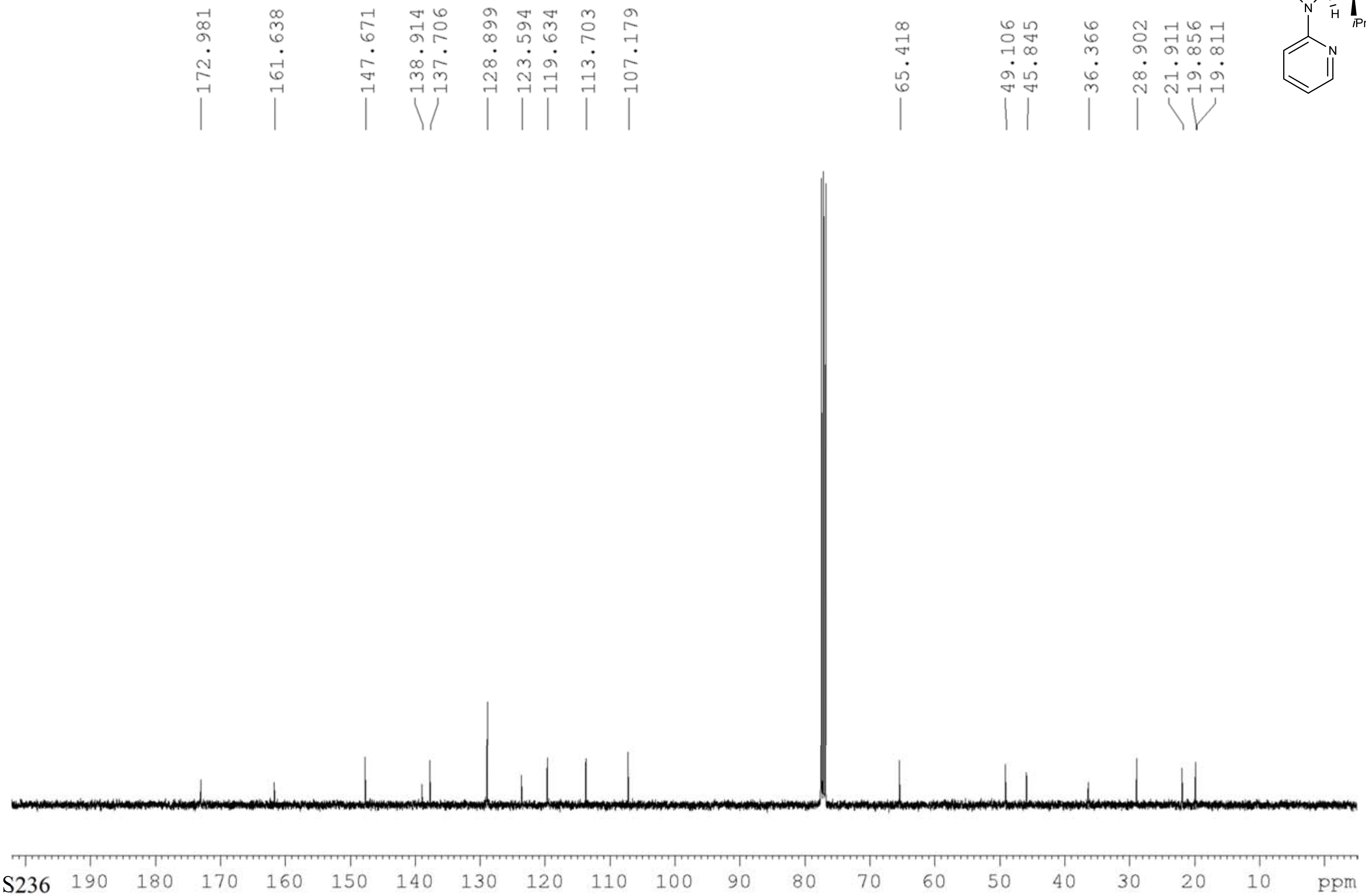

^{1}H NMR (400 MHz, $CDCl_3$) for ***(R)*-*N*-Phenyl-3-((*S*)-1-(pyridin-2-yl)azepan-2-yl)butanamide (6l)**

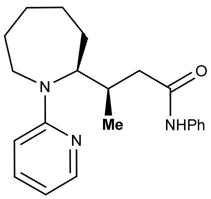

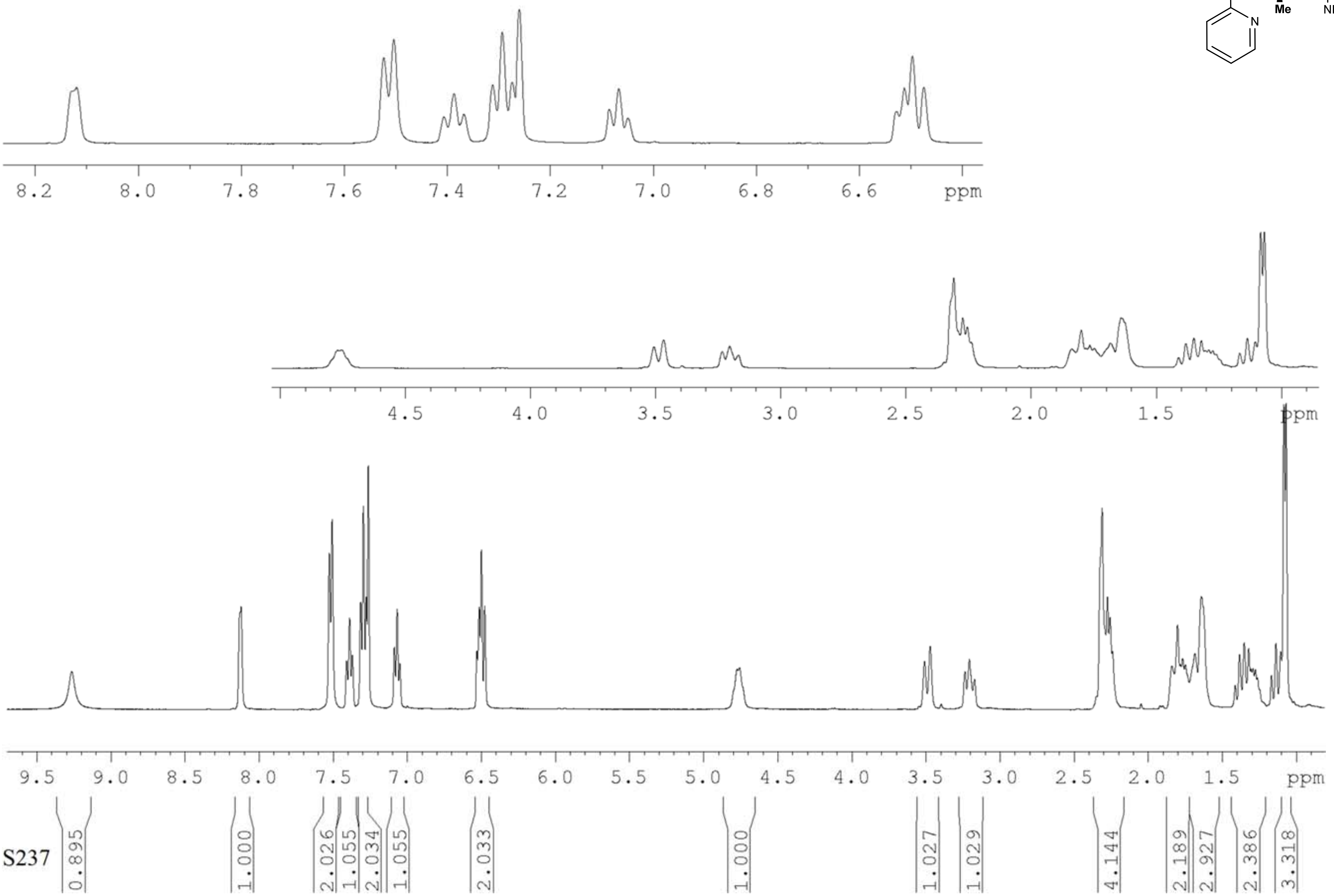

^{13}C NMR (126 MHz, $CDCl_3$) for **(*R*)-*N*-Phenyl-3-((*S*)-1-(pyridin-2-yl)azepan-2-yl)butanamide (6l)**

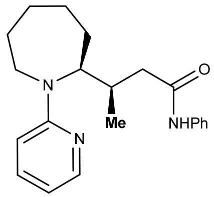

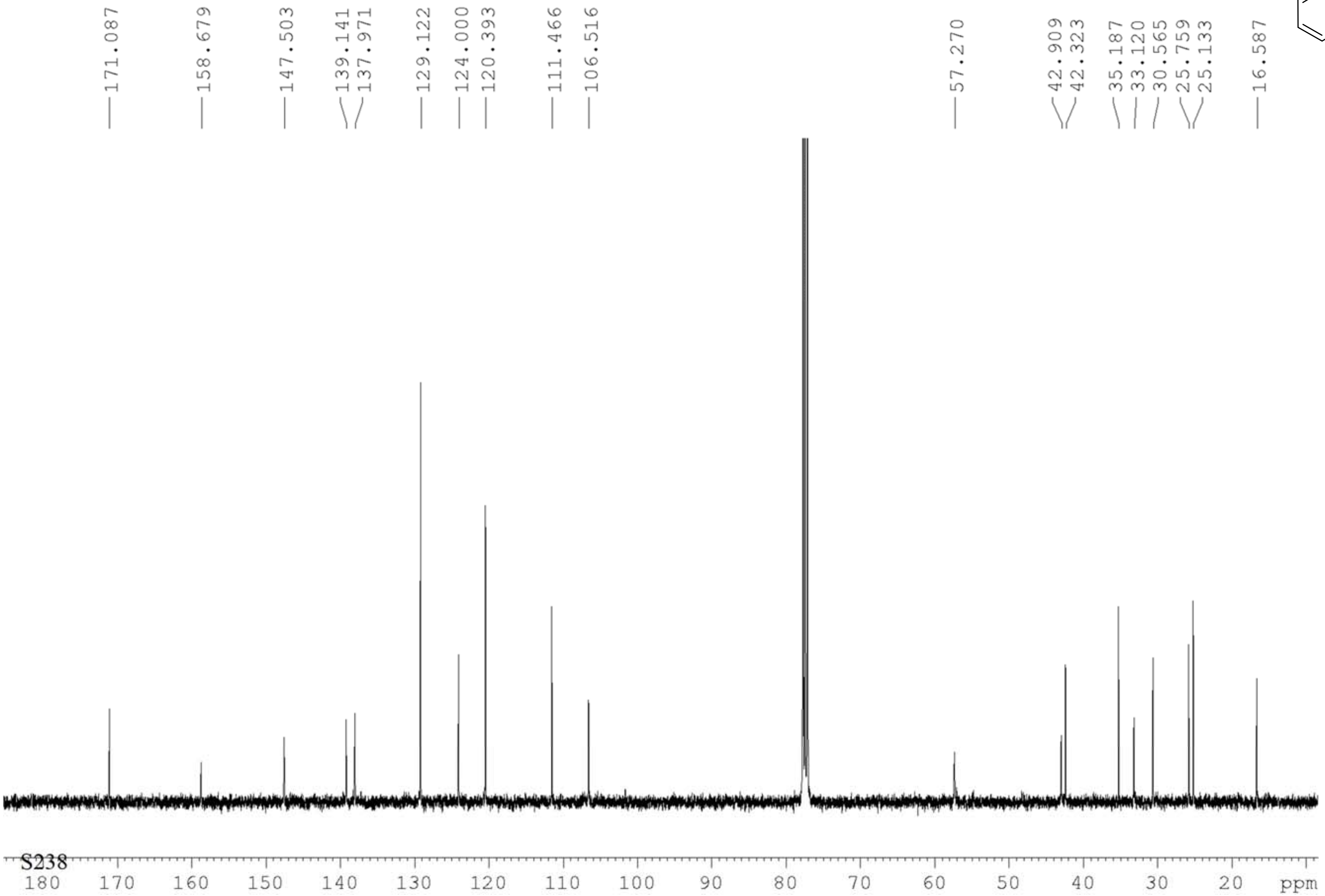

^{1}H NMR (500 MHz, $CDCl_3$) for **(2*S*,3*S*)-2-Methyl-*N*-phenyl-3-((*S*)-1-(pyridin-2-yl)azetidin-2-yl)butanamide (6m)**

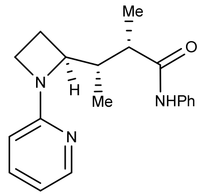

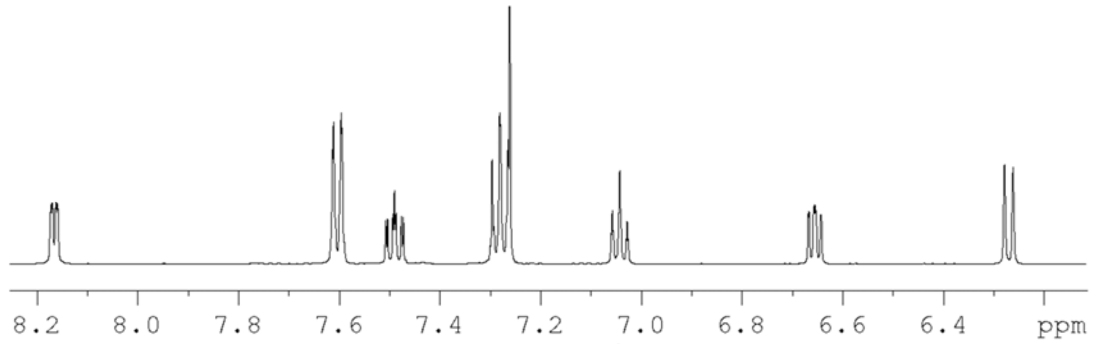

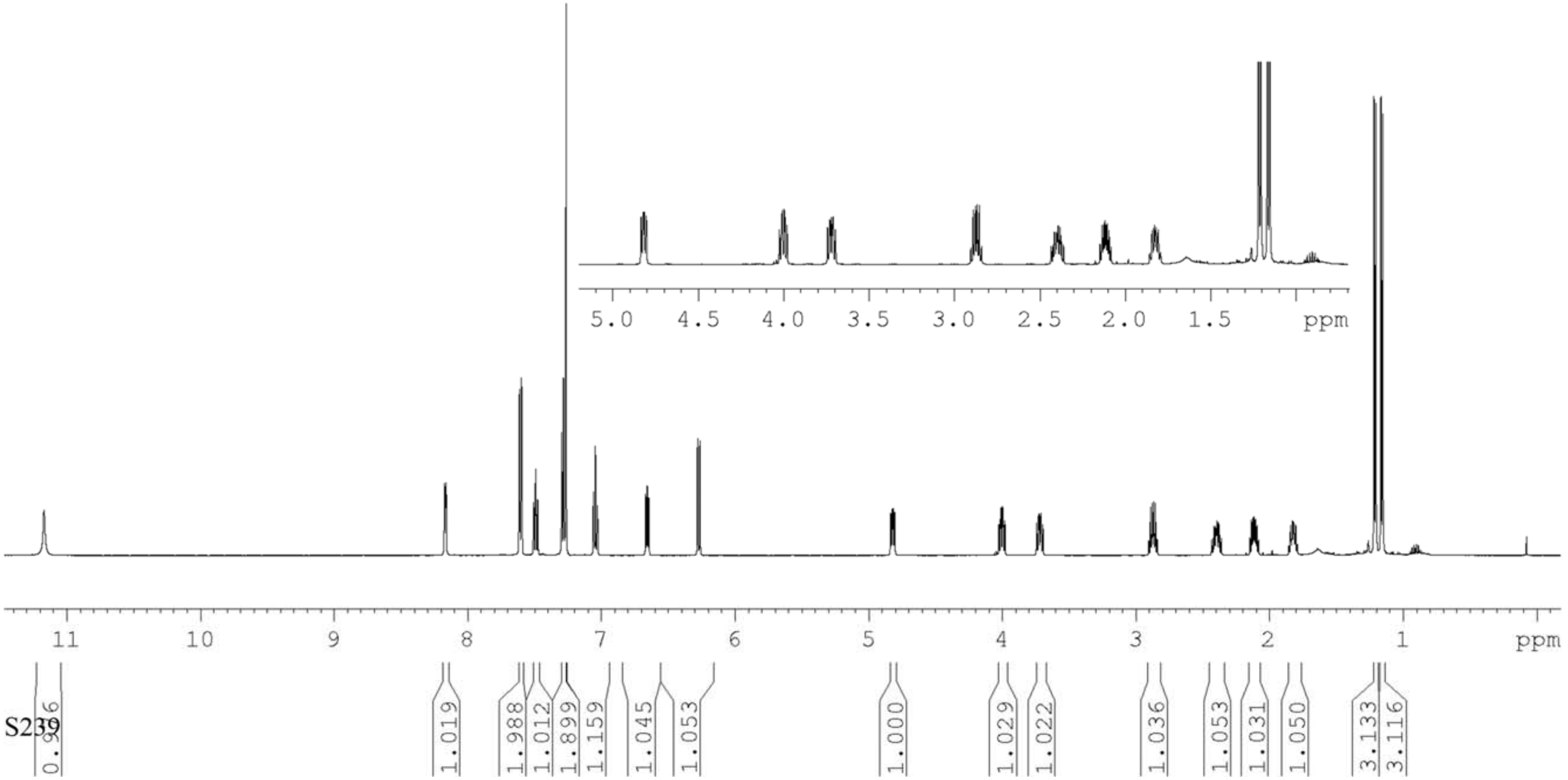

^{13}C NMR (101 MHz, $CDCl_3$) for **(2*S*,3*S*)-2-Methyl-*N*-phenyl-3-((*S*)-1-(pyridin-2-yl)azetidin-2-yl)butanamide (6m)**

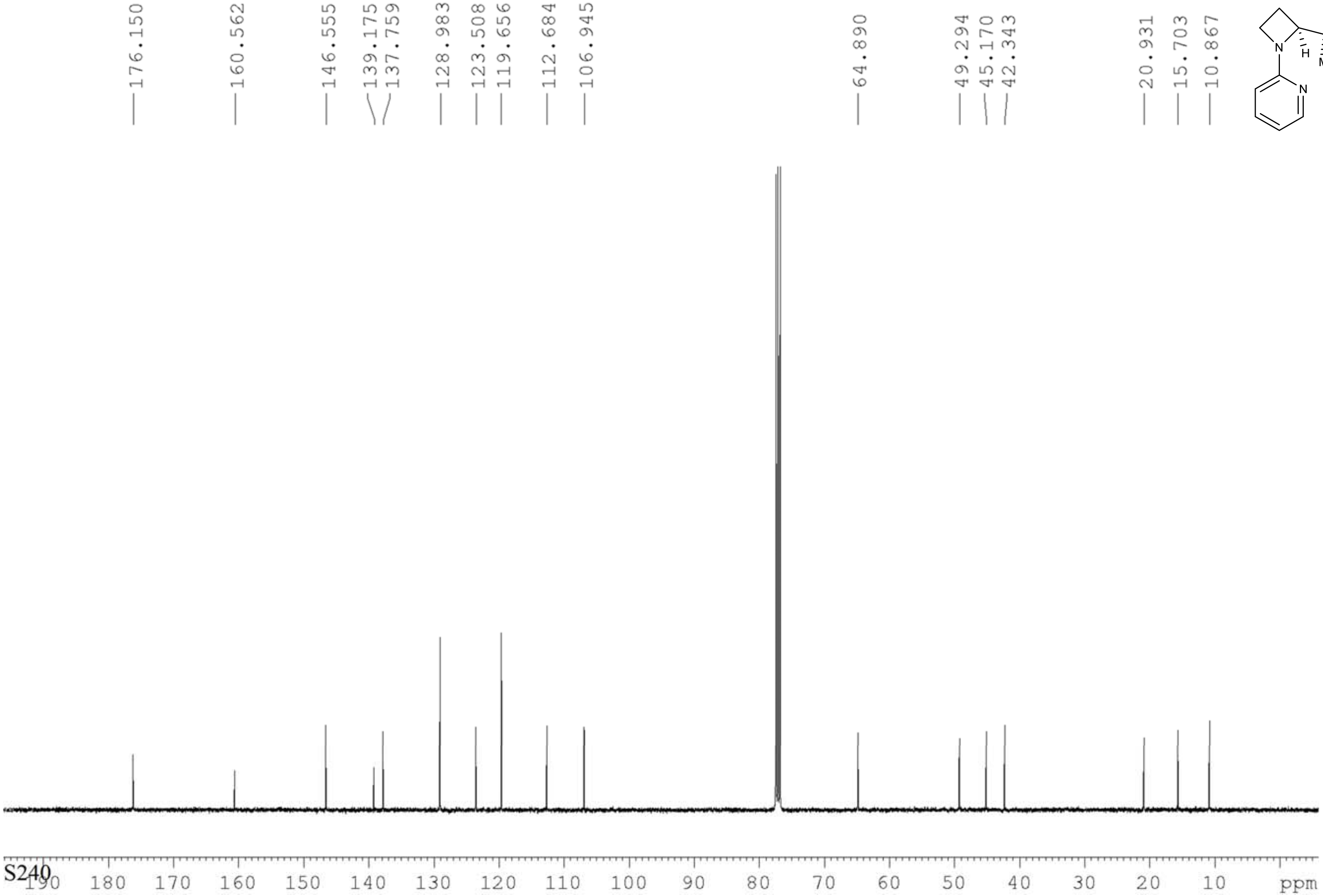

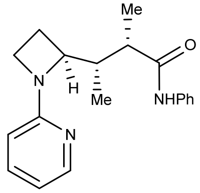

¹H NMR (700 MHz, $CDCl_3$) for **(2S,3S)-2-Methyl-*N*-phenyl-3-((*S*)-1-(pyridin-2-yl)pyrrolidin-2-yl)butanamide (6n)**

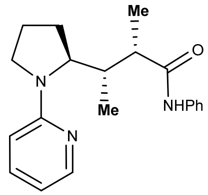

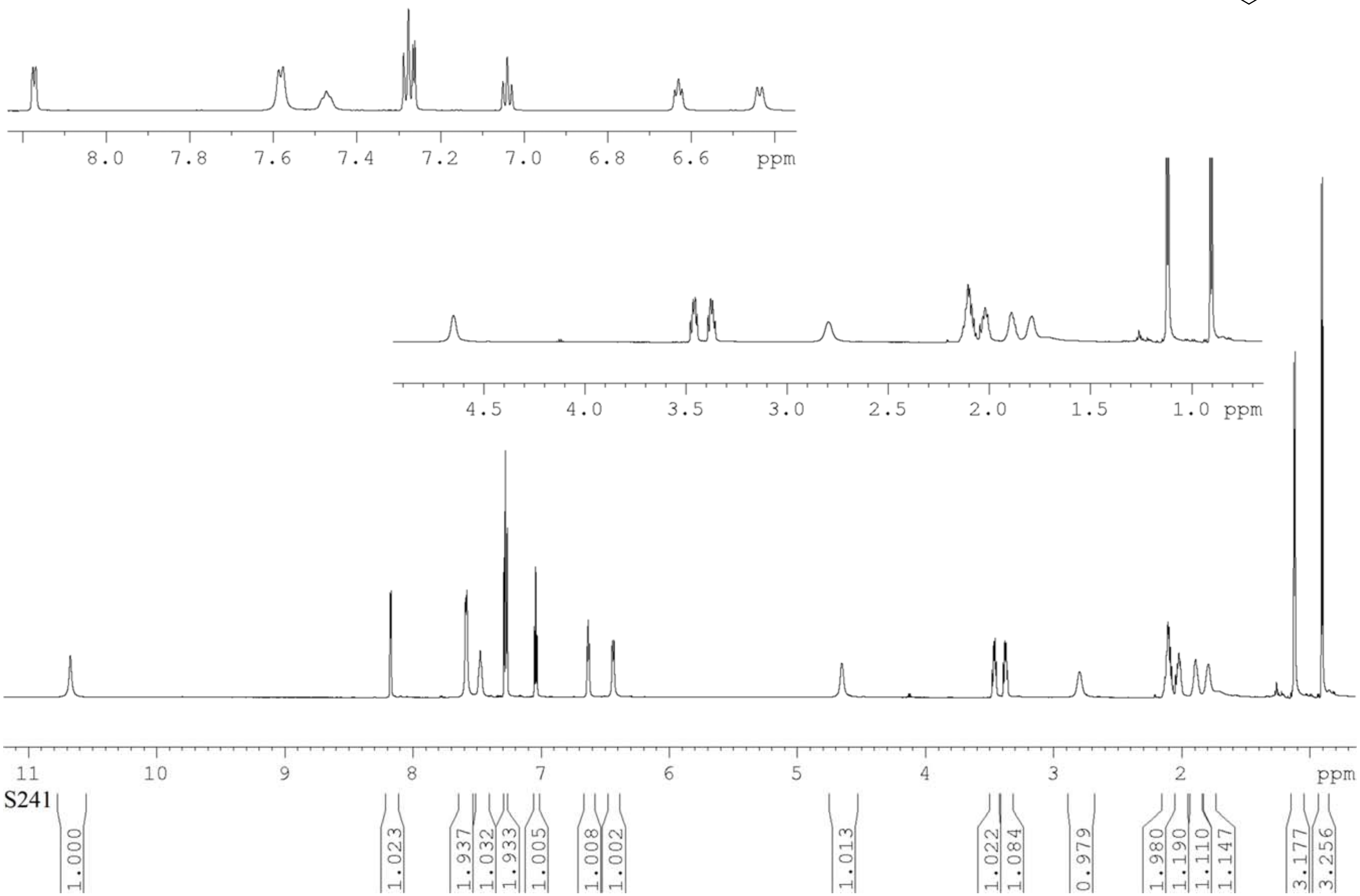

^{13}C NMR (176 MHz, $CDCl_3$) for **(*2S,3S*)-2-Methyl-*N*-phenyl-3-((*S*)-1-(pyridin-2-yl)pyrrolidin-2-yl)butanamide (6n)**

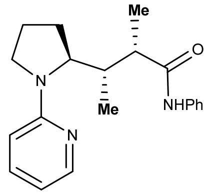

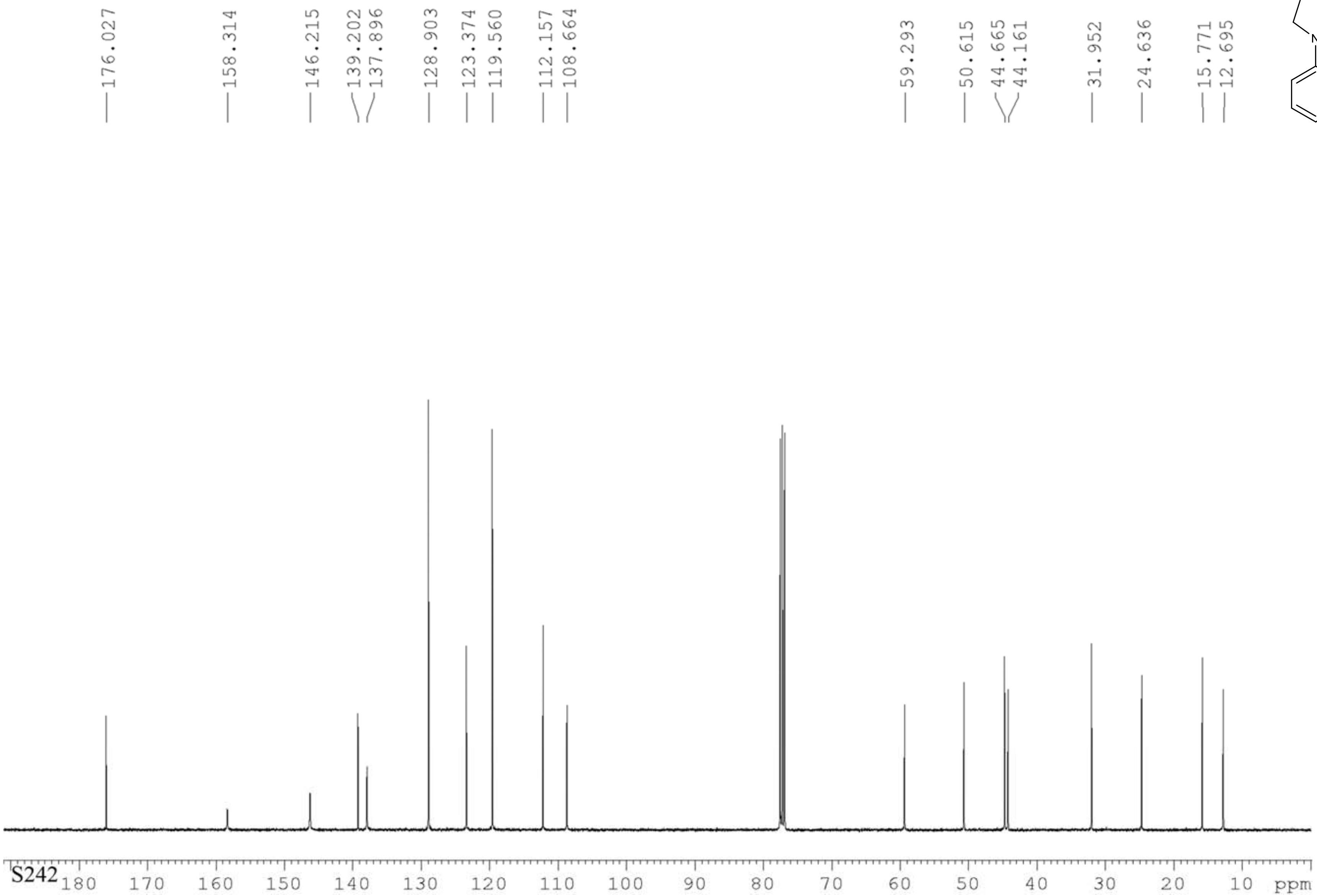

^{1}H NMR (700 MHz, $CDCl_3$) for ***(2S,3S)*-2-Methyl-*N*-phenyl-3-((*S*)-1-(pyridin-2-yl)azepan-2-yl)butanamide (6o)**

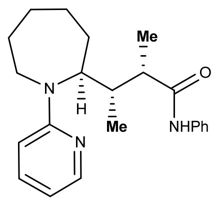

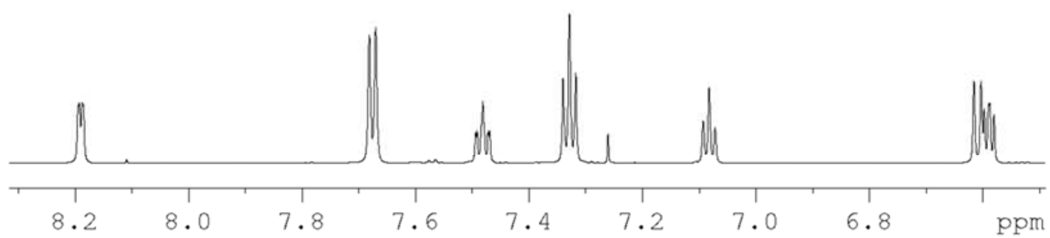

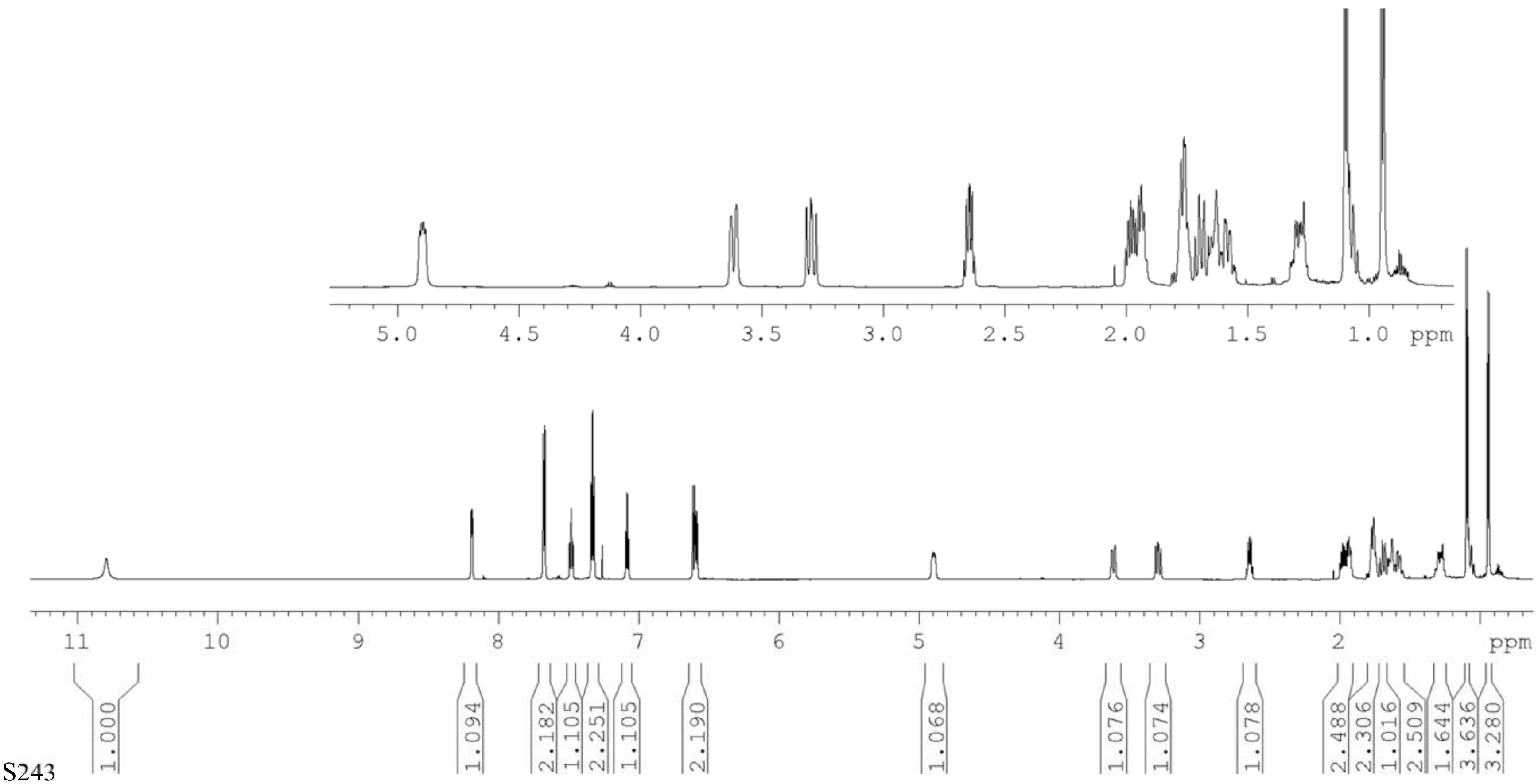

^{13}C NMR (176 MHz, $CDCl_3$) for **(*2S,3S*)-2-Methyl-*N*-phenyl-3-((*S*)-1-(pyridin-2-yl)azepan-2-yl)butanamide (6o)**

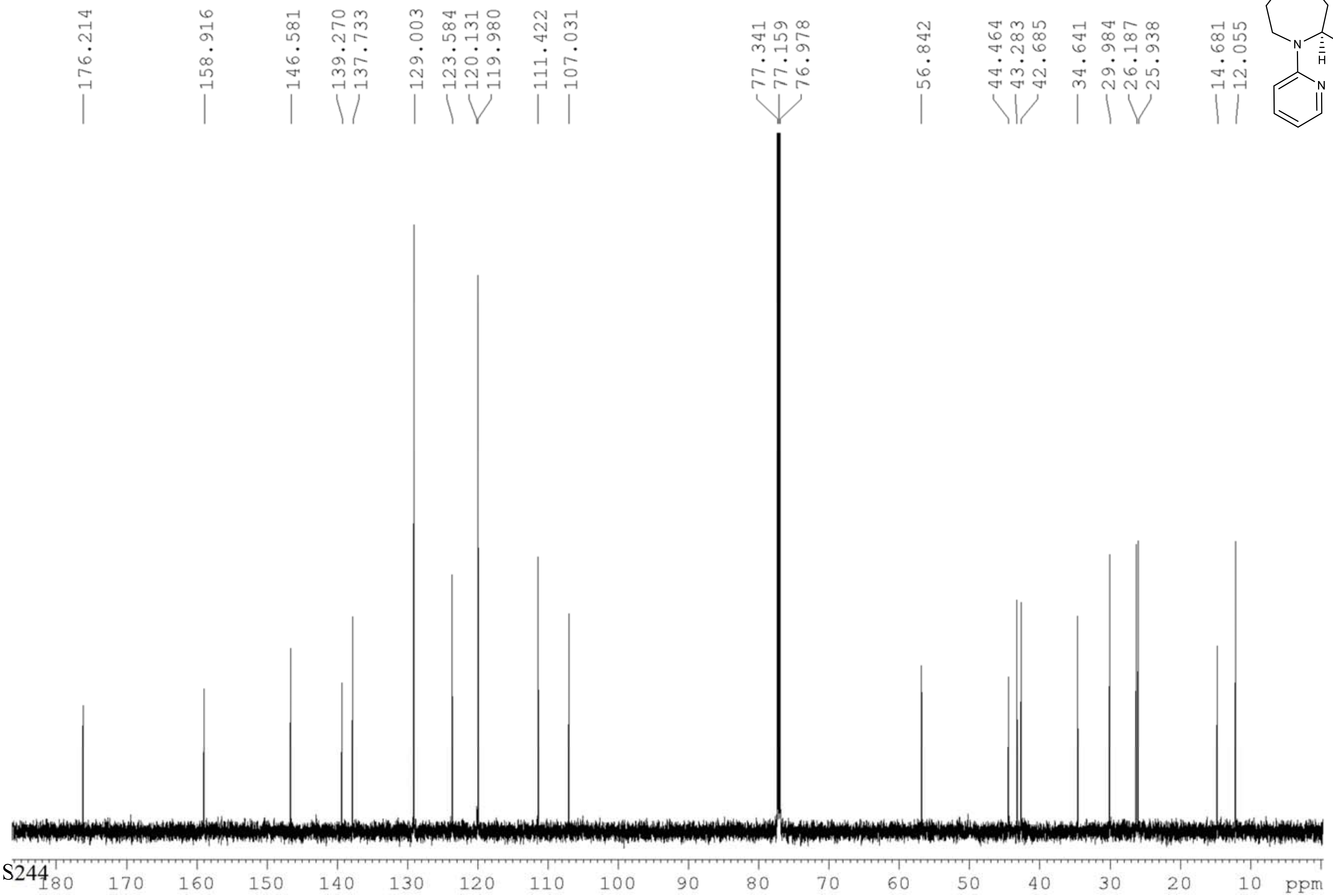

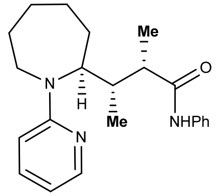

^{1}H NMR (700 MHz, $CDCl_3$) for **(*R*)-3-((Benzyl(pyridin-2-yl)amino)methyl)-5-methyl-*N*-phenylhexanamide (6p)**

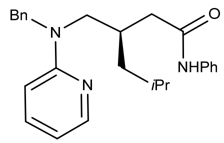

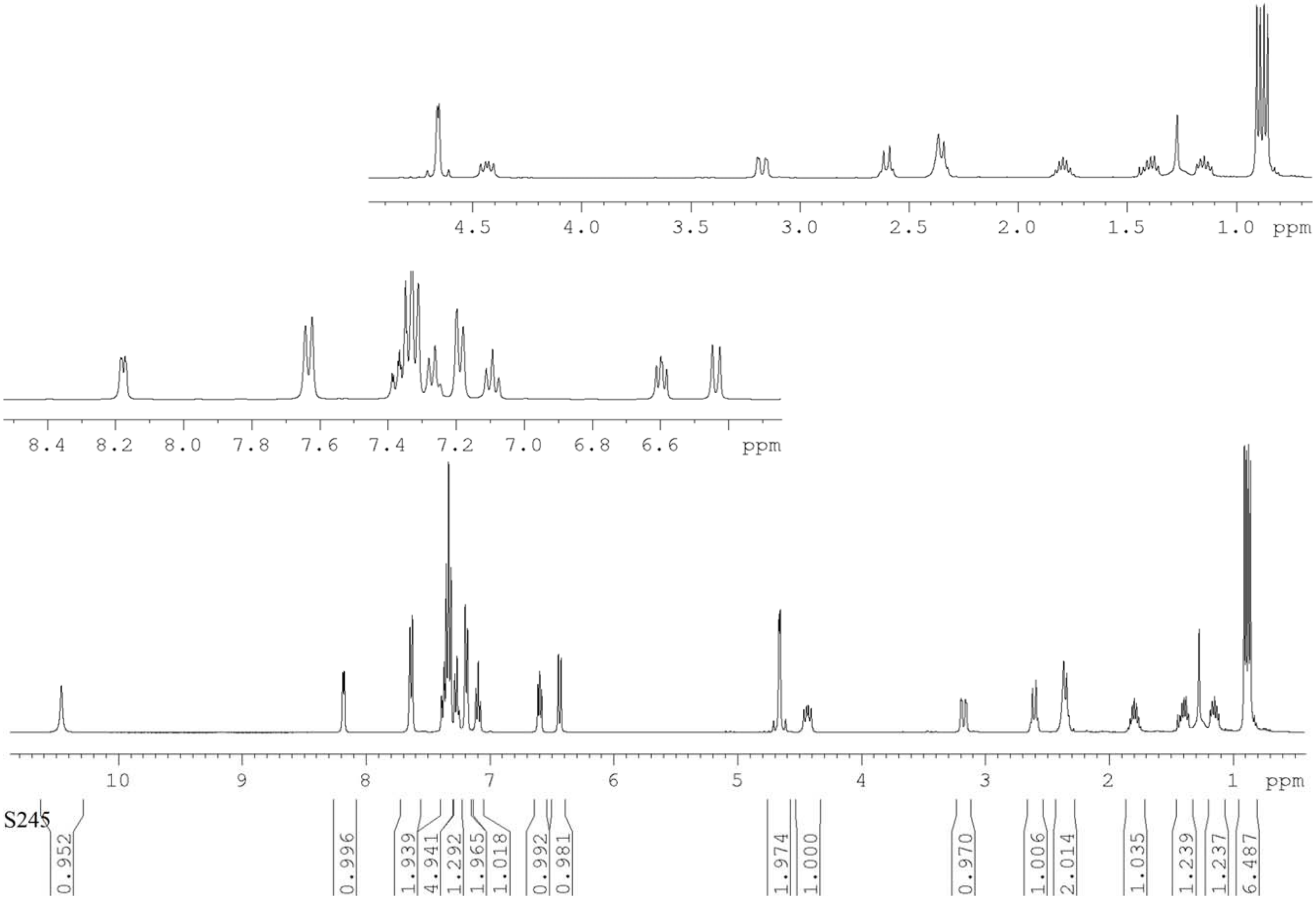

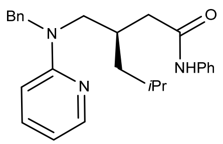

^{13}C NMR (176 MHz, $CDCl_3$) for **(*R*)-3-((Benzyl(pyridin-2-yl)amino)methyl)-5-methyl-*N*-phenylhexanamide (6p)**

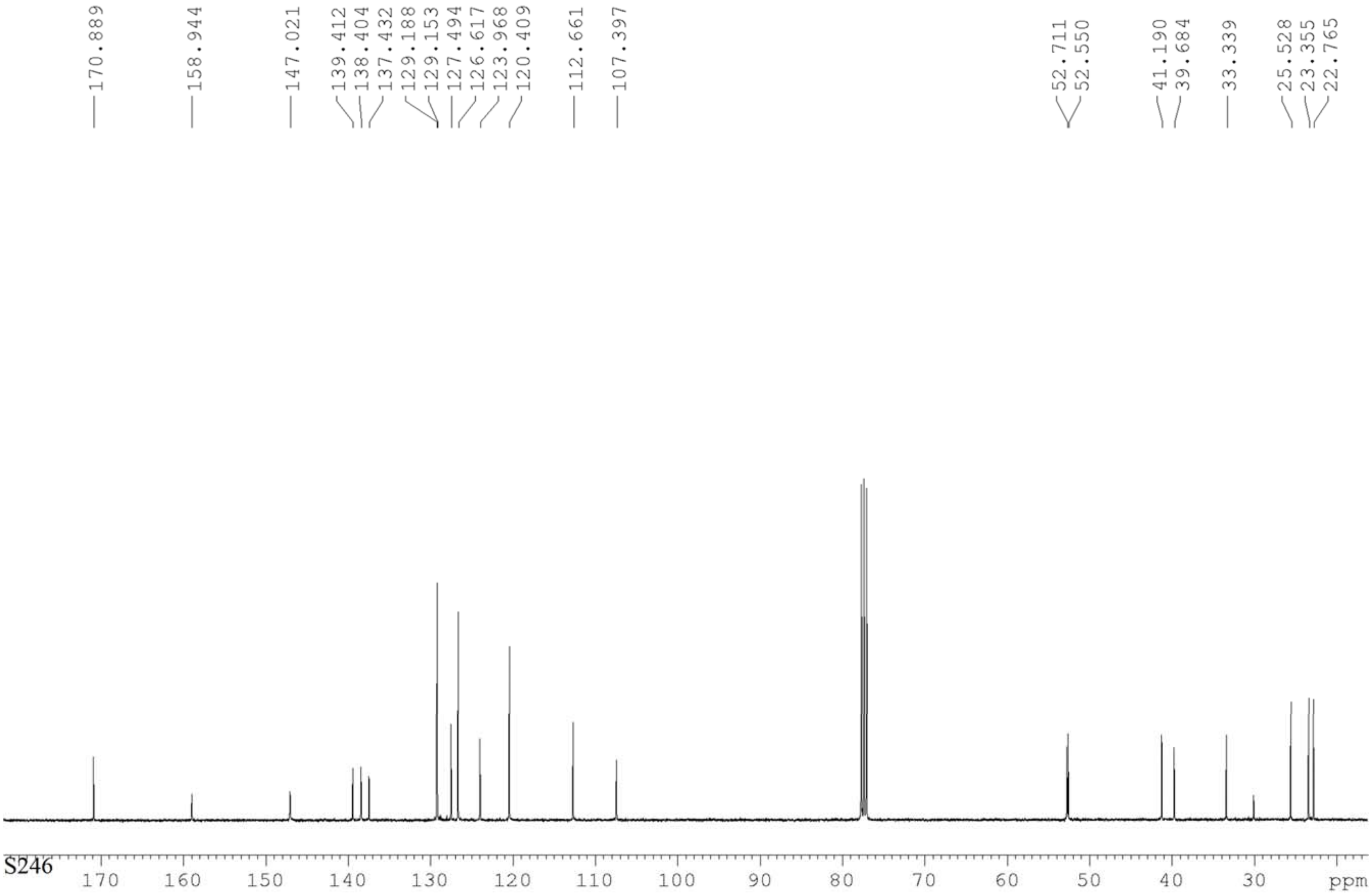

^{1}H NMR (400 MHz, $CDCl_3$) for **(*R*)-*N*-Phenyl-3-((*S*)-1-(pyridin-2-yl)pyrrolidin-2-yl)butanamide (6q)**

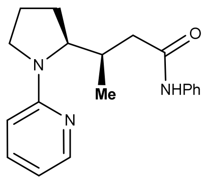

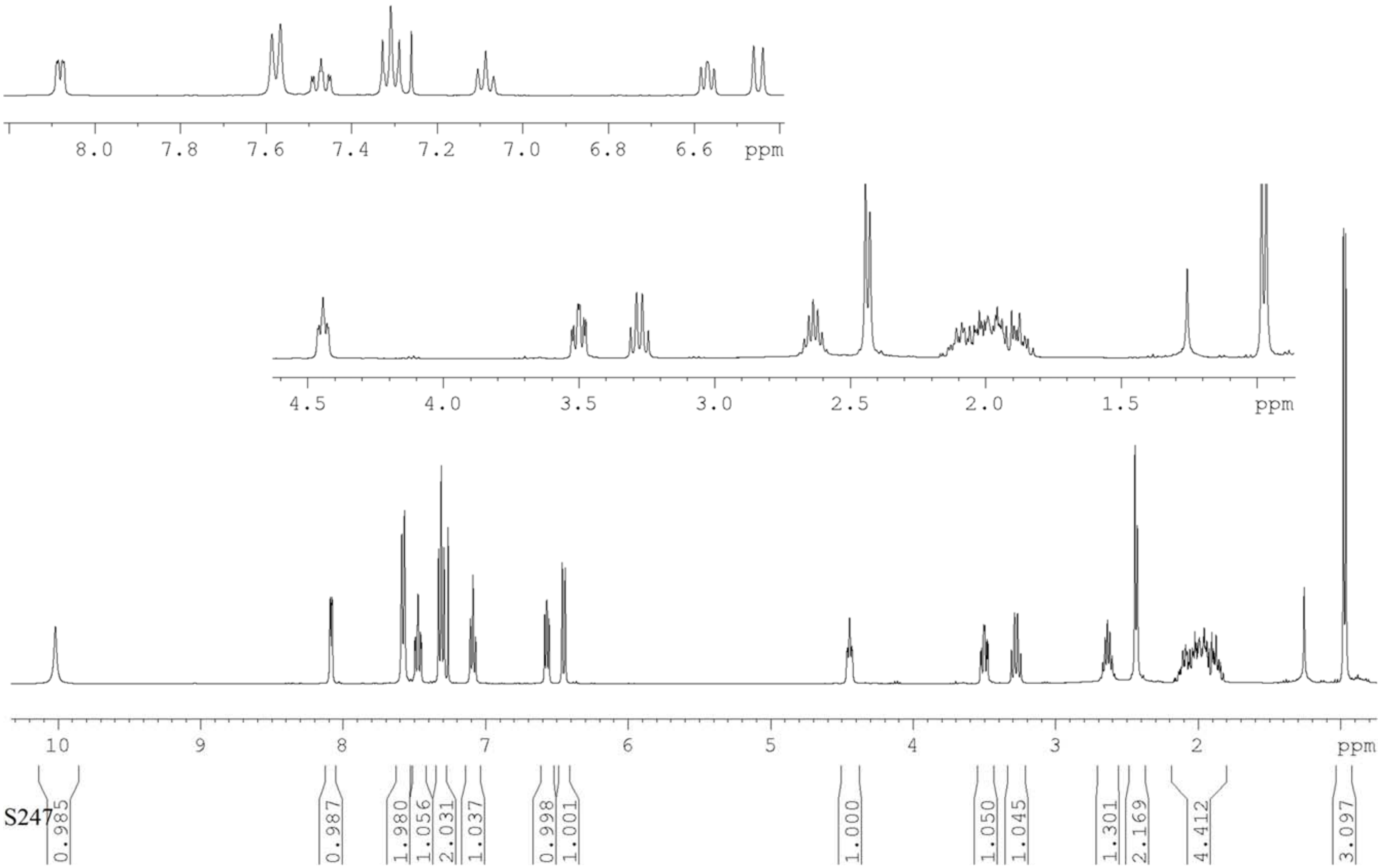

^{13}C NMR (126 MHz, $CDCl_3$) for **(*R*)-*N*-Phenyl-3-((*S*)-1-(pyridin-2-yl)pyrrolidin-2-yl)butanamide (6q)**

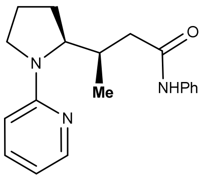

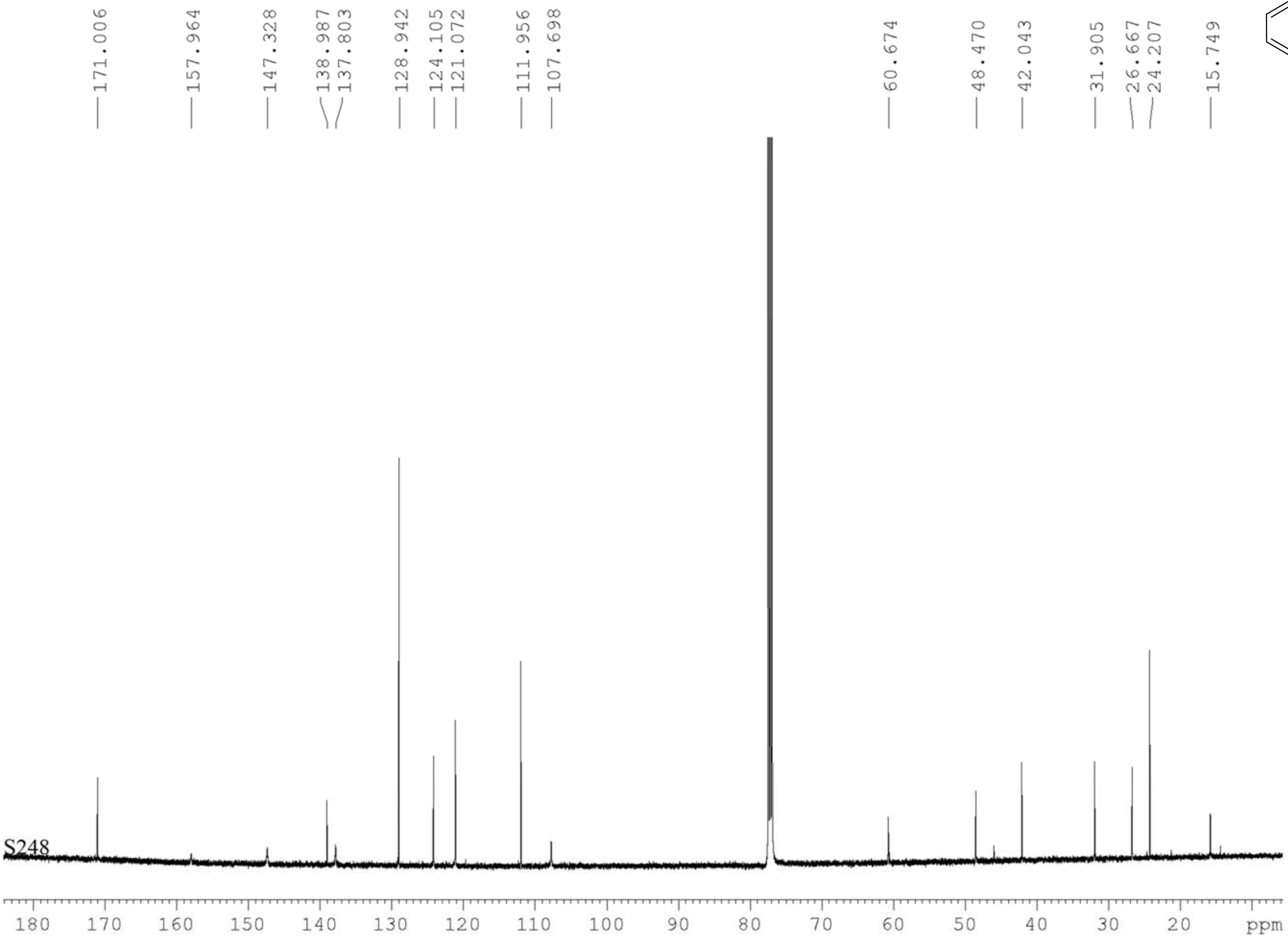

[1]**H NMR** (700 MHz, $CDCl_3$) for **(5*S*,6a*S*)-5-Benzyl-*N*-phenyl-5,6,6a,7,8,9-hexahydropyrrolo[1,2-a][1,5]naphthyridine-5-carboxamide (7ba)**

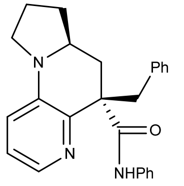

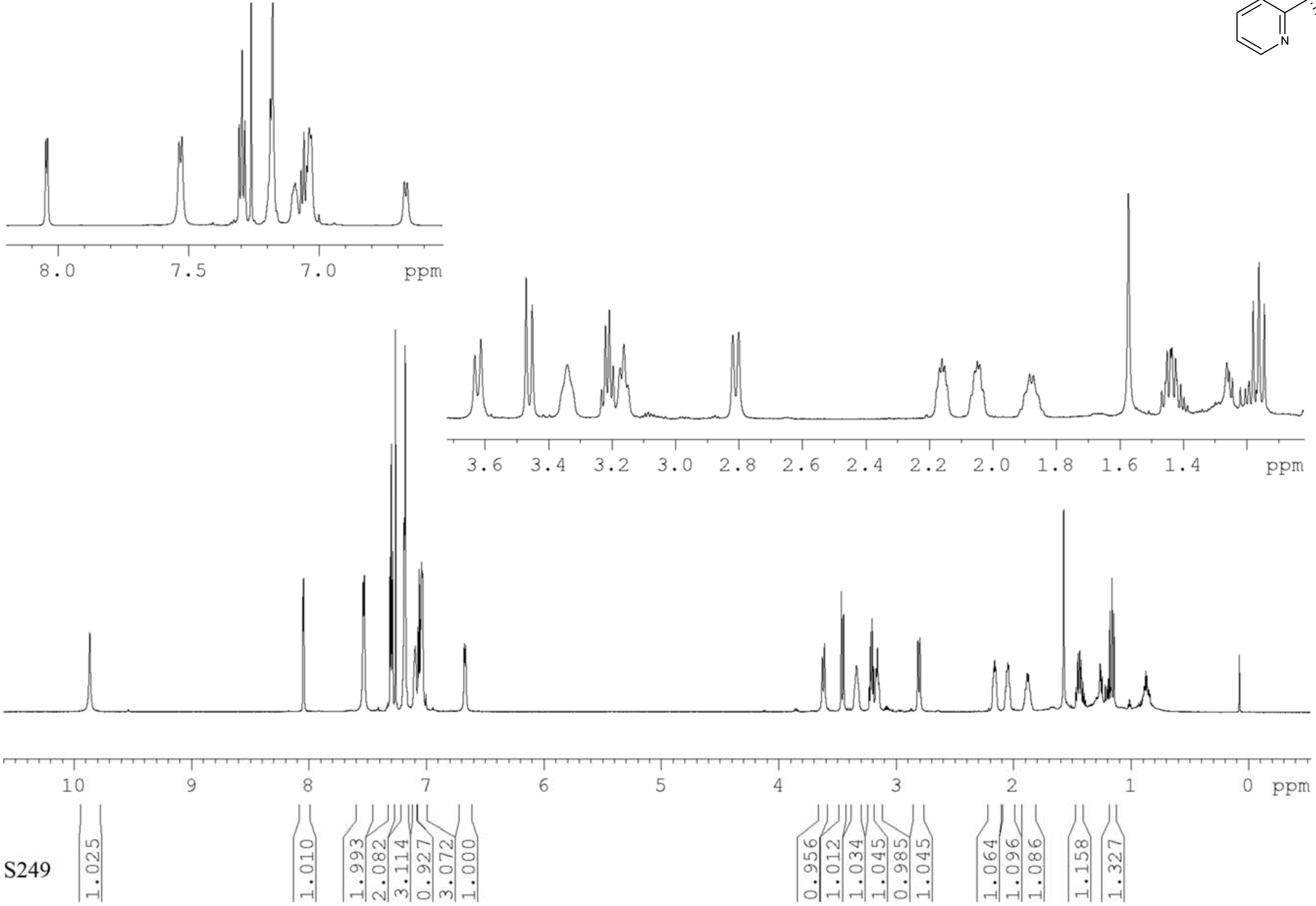

^{13}C NMR (126 MHz, $CDCl_3$) for **(5*S*,6a*S*)-5-Benzyl-*N*-phenyl-5,6,6a,7,8,9-hexahydropyrrolo[1,2-a][1,5]naphthyridine-5-carboxamide (7ba)**

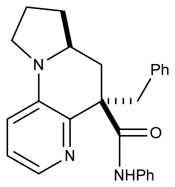

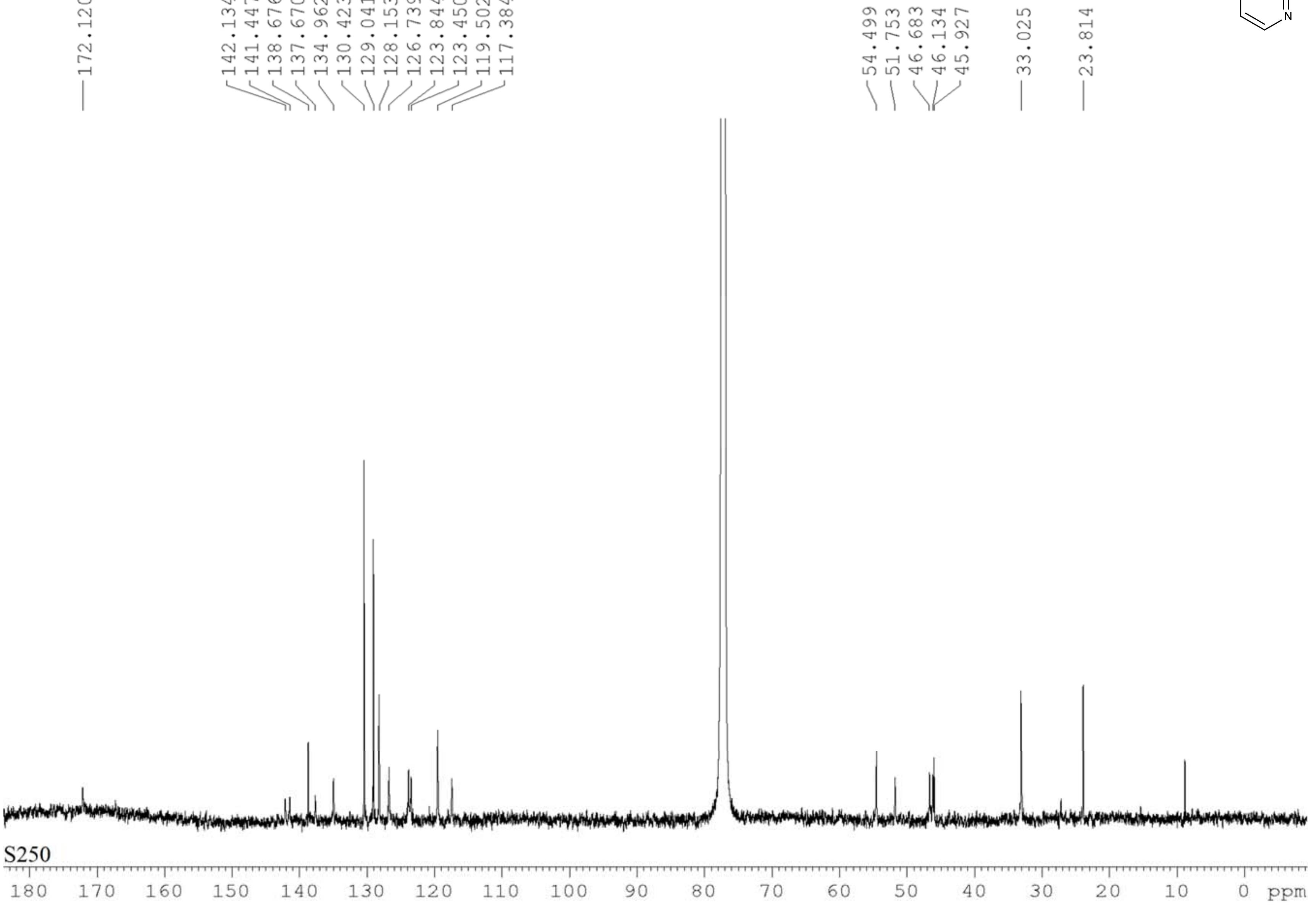

^{1}H NMR (500 MHz, $CDCl_3$) for **(5*R*,6a*S*)-5-Benzyl-*N*-phenyl-5,6,6a,7,8,9-hexahydropyrrolo[1,2-a][1,5]naphthyridine-5-carboxamide (7bb)**

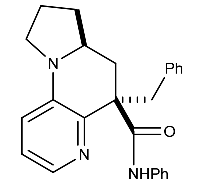

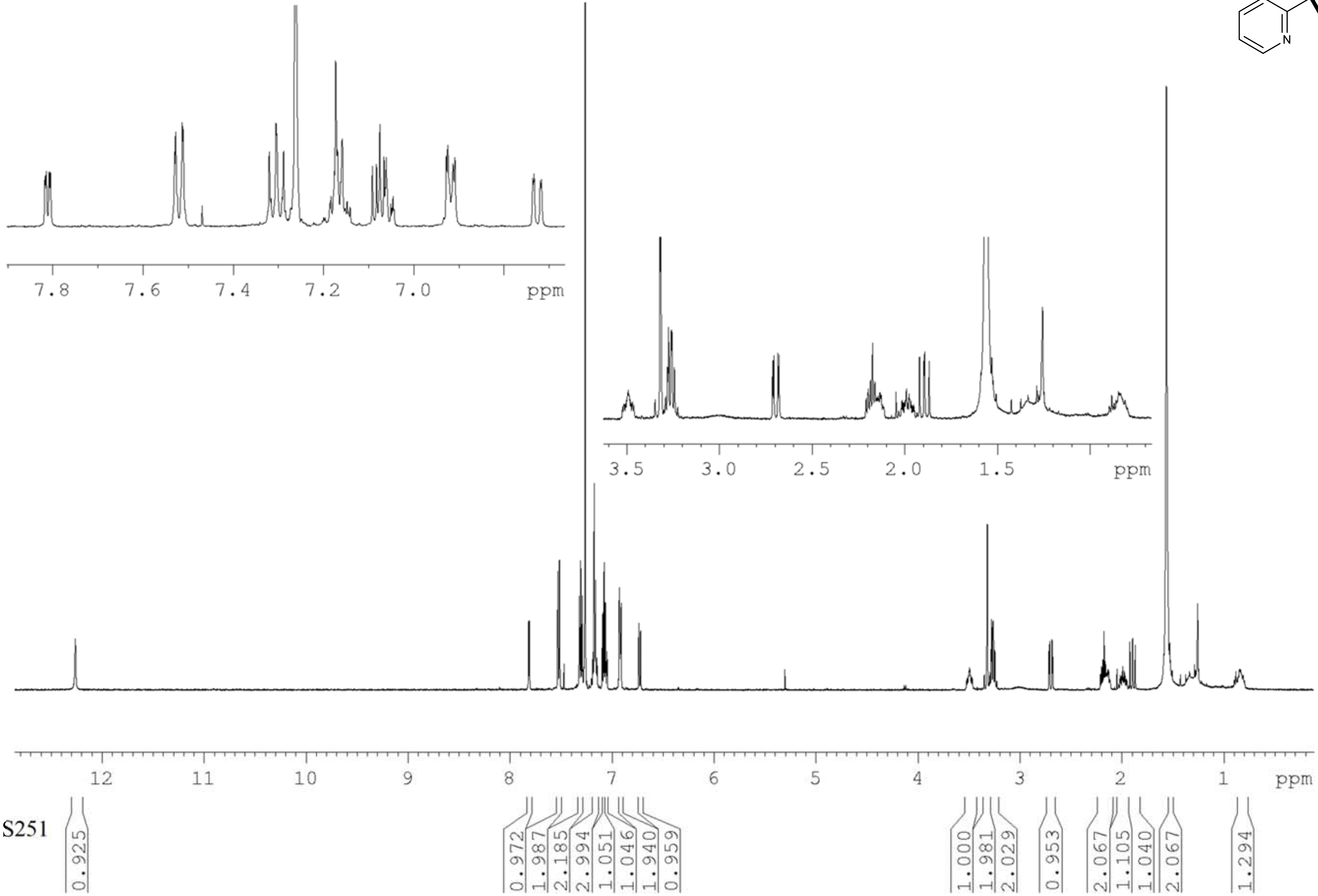

^{13}C NMR (126 MHz, $CDCl_3$) for **(5*R*,6a*S*)-5-Benzyl-*N*-phenyl-5,6,6a,7,8,9-hexahydropyrrolo[1,2-a][1,5]naphthyridine-5-carboxamide (7bb)**

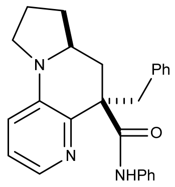

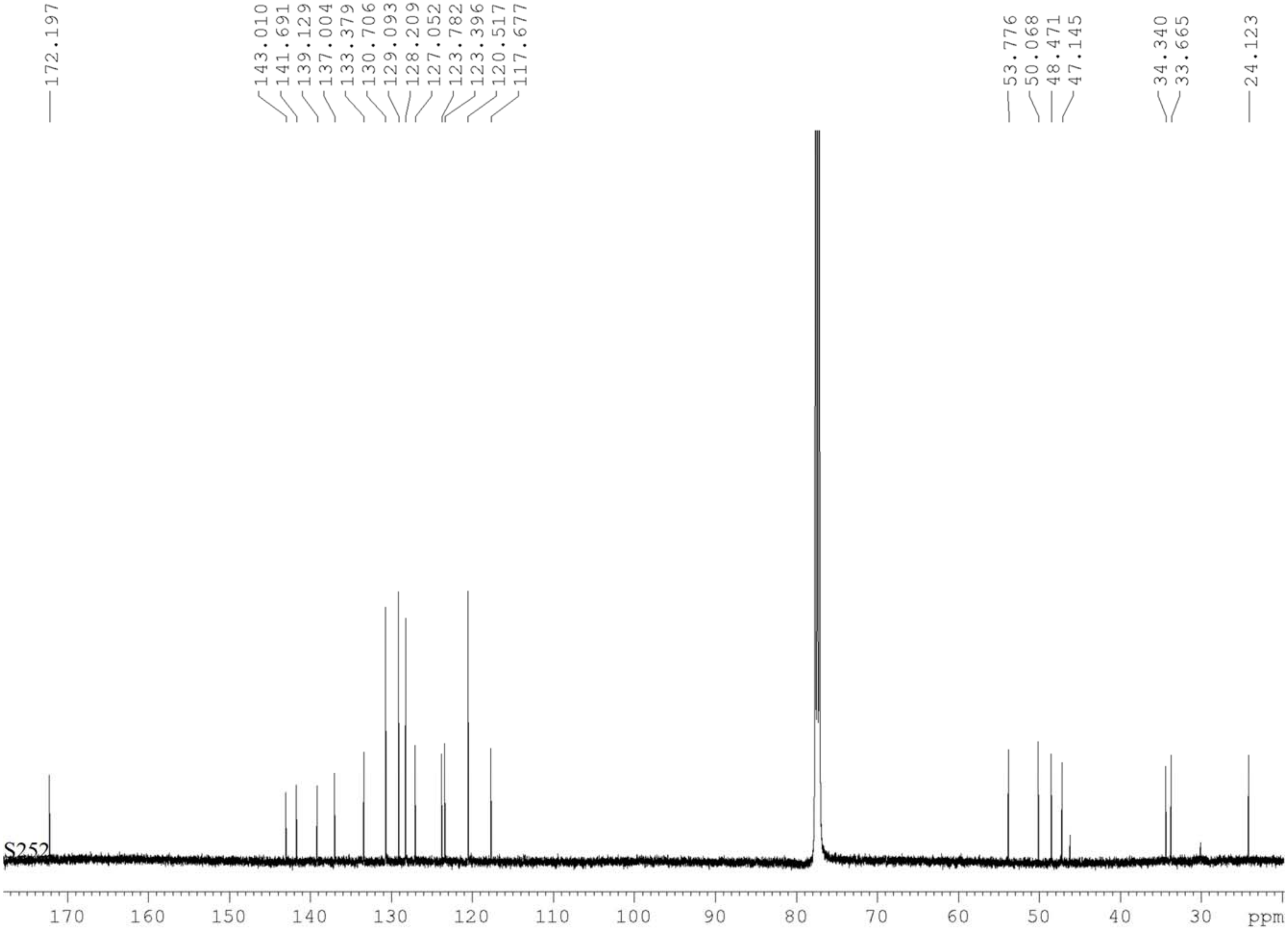

^{1}H NMR (500 MHz, $CDCl_3$) for **(*S*)-2-Benzyl-*N*-methyl-*N*-phenyl-3-((*S*)-1-(pyridin-2-yl)pyrrolidin-2-yl)propenamide (5na) and (*R*)-2-benzyl-*N*-methyl-*N*-phenyl-3-((*S*)-1-(pyridin-2-yl)pyrrolidin-2-yl)propenamide (5nb)**

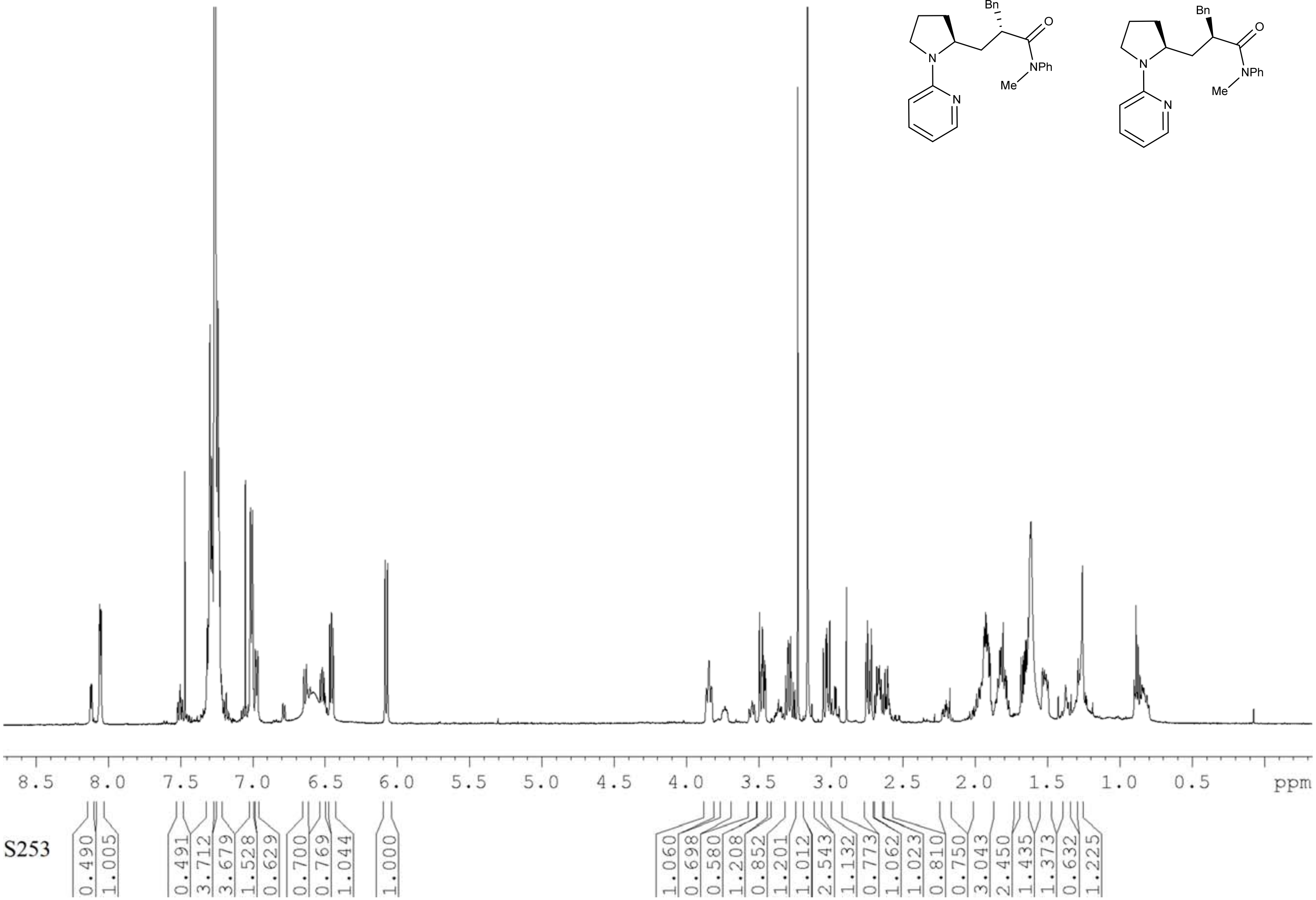

^{13}C NMR (126 MHz, $CDCl_3$) for **(*S*)-2-Benzyl-*N*-methyl-*N*-phenyl-3-((*S*)-1-(pyridin-2-yl)pyrrolidin-2-yl)propenamide (5na) and (*R*)-2-benzyl-*N*-methyl-*N*-phenyl-3-((*S*)-1-(pyridin-2-yl)pyrrolidin-2-yl)propenamide (5nb)**

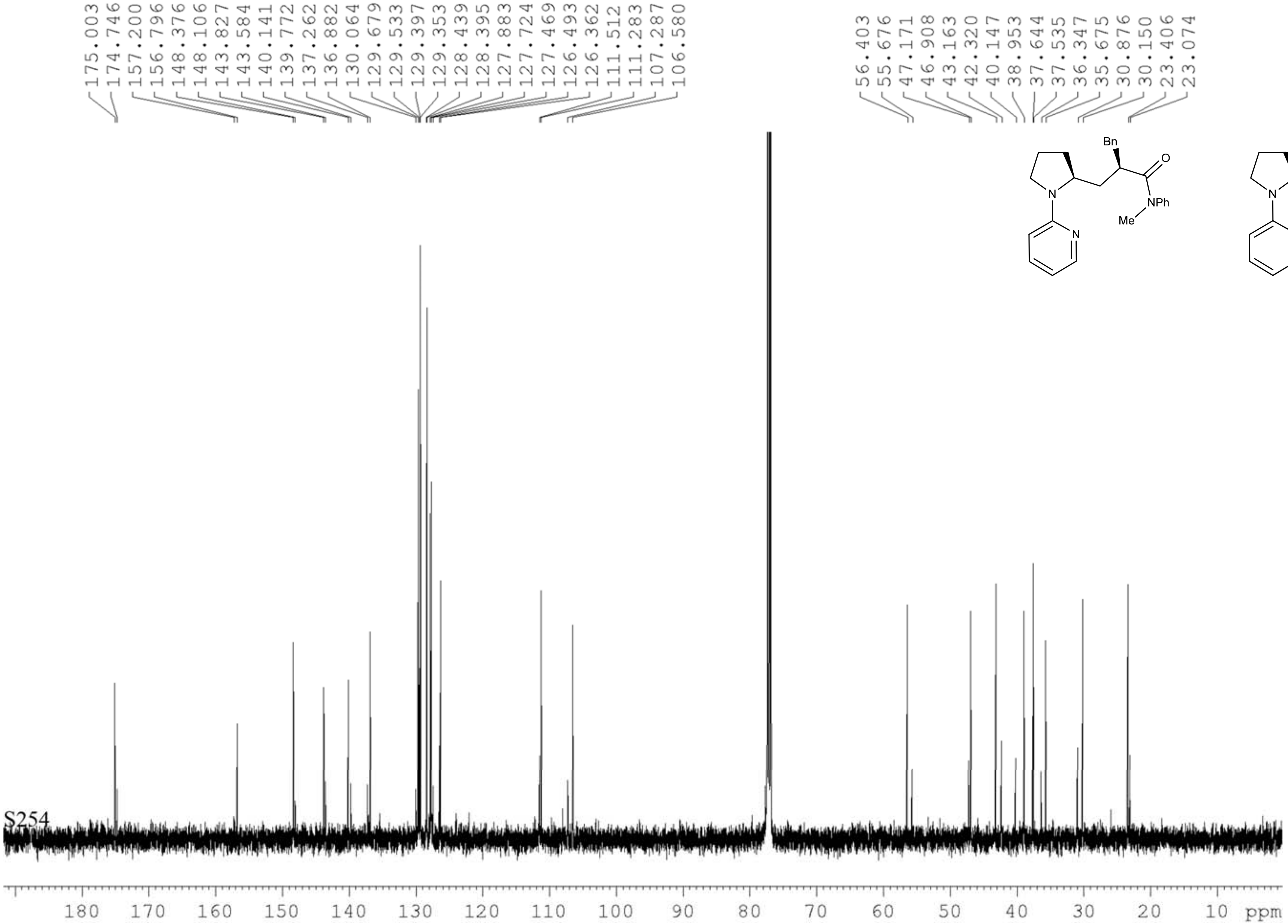

^{1}H NMR (500 MHz, $CDCl_3$) for ***(S)*-2-Benzyl-*N*-mesityl-3-((S)-1-(pyridin-2-yl)pyrrolidin-2-yl)propenamide (5oa)**

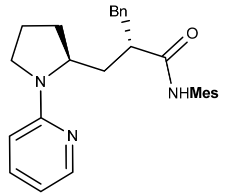

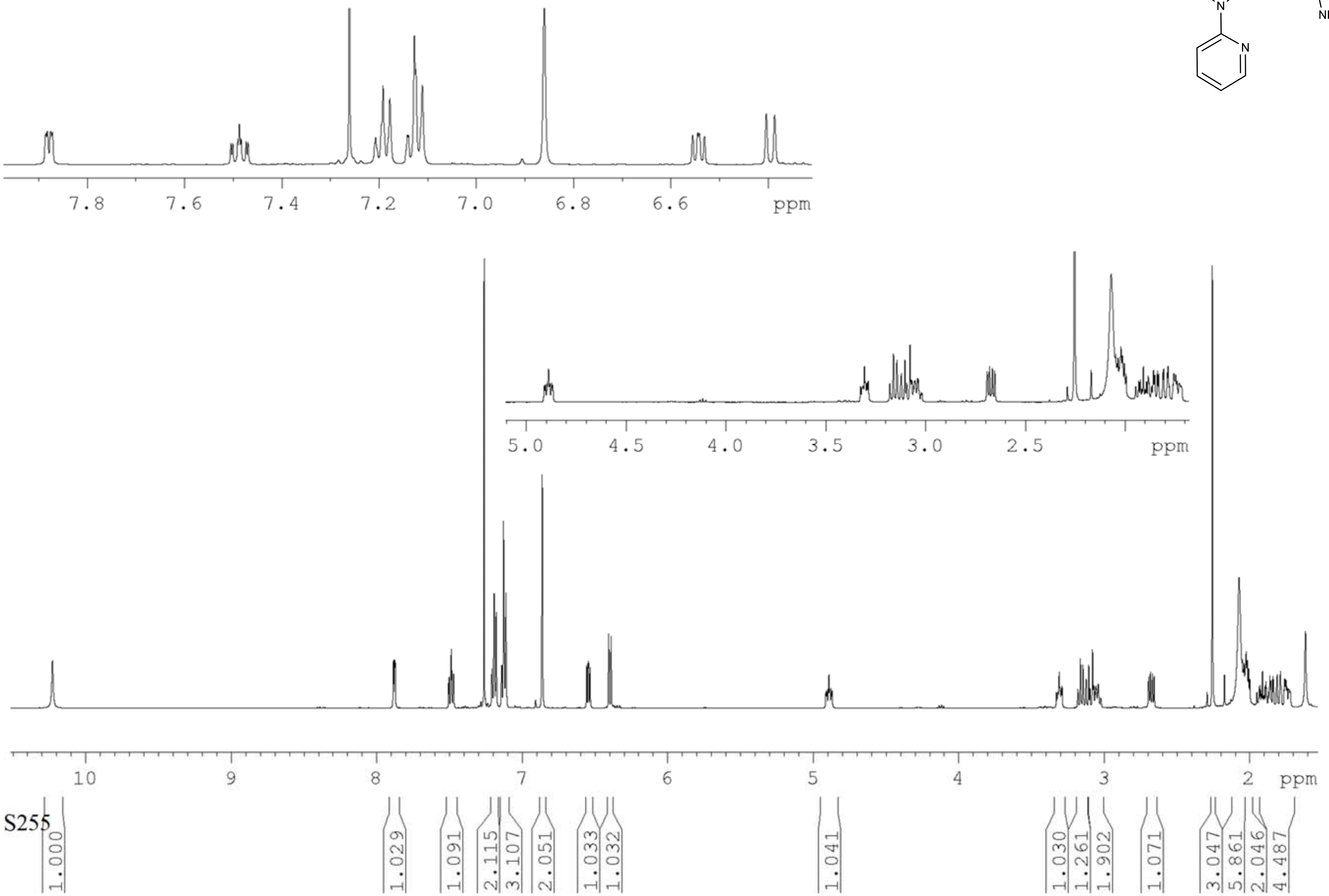

^{13}C NMR (126 MHz, $CDCl_3$) for **(*S*)-2-Benzyl-*N*-mesityl-3-((S)-1-(pyridin-2-yl)pyrrolidin-2-yl)propenamide (5oa)**

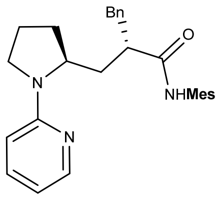

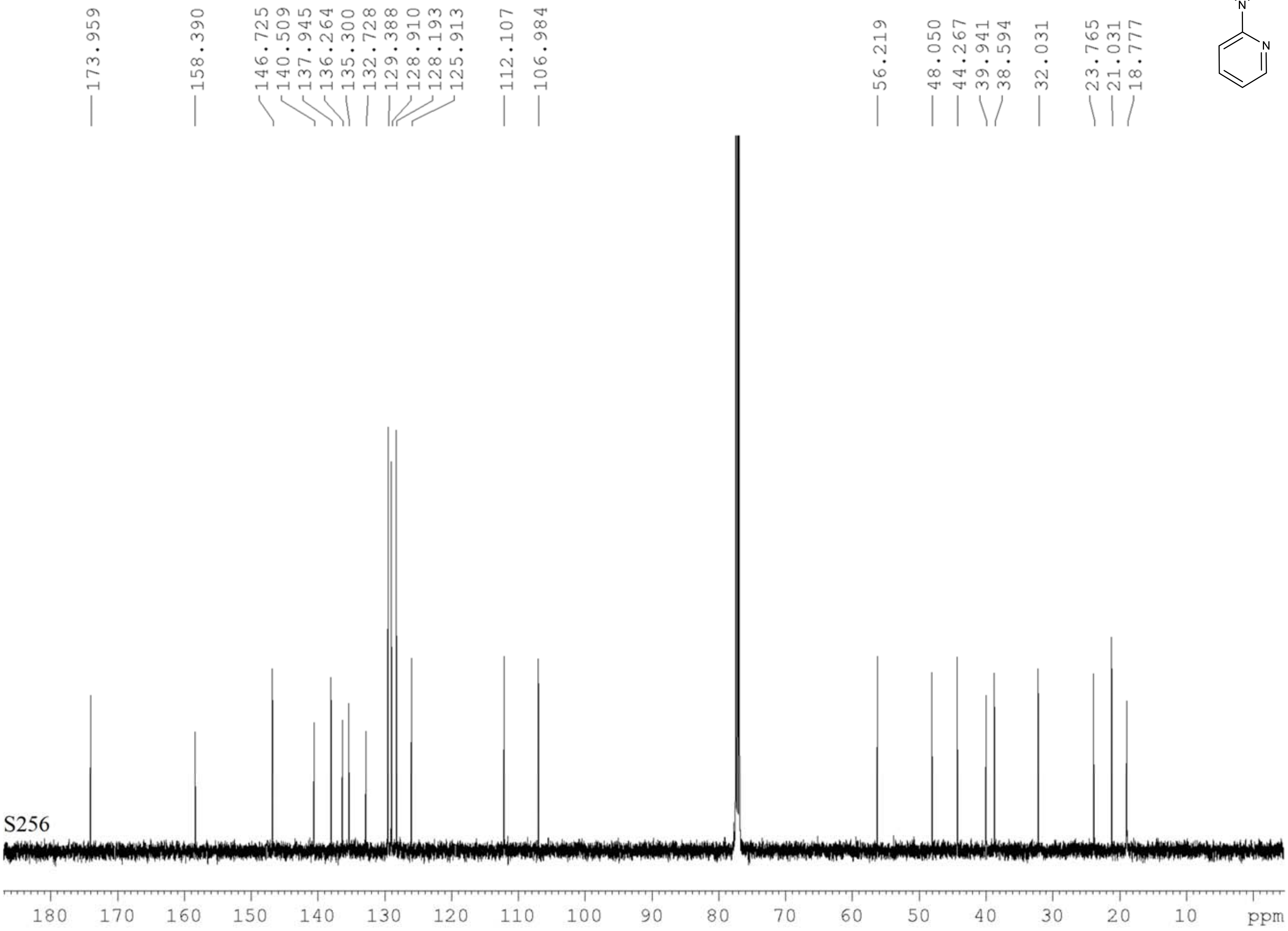

^{1}H NMR (500 MHz, $CDCl_3$) for **(*R*)-2-Benzyl-*N*-mesityl-3-((S)-1-(pyridin-2-yl)pyrrolidin-2-yl)propenamide (5ob)**

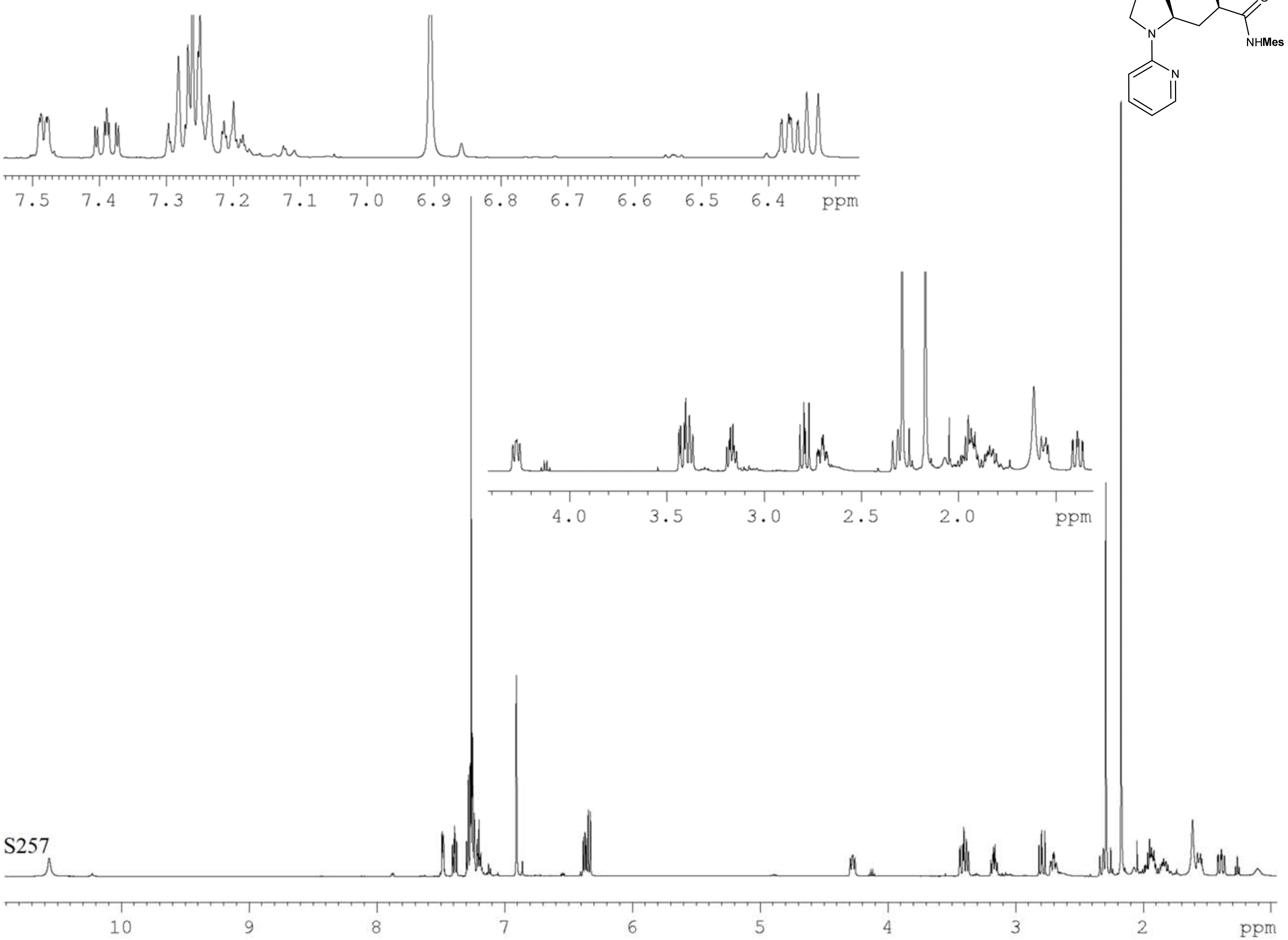

^{13}C NMR (126 MHz, $CDCl_3$) for **(*R*)-2-Benzyl-*N*-mesityl-3-((S)-1-(pyridin-2-yl)pyrrolidin-2-yl)propenamide (5ob)**

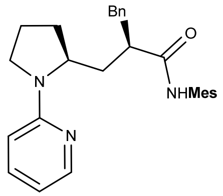

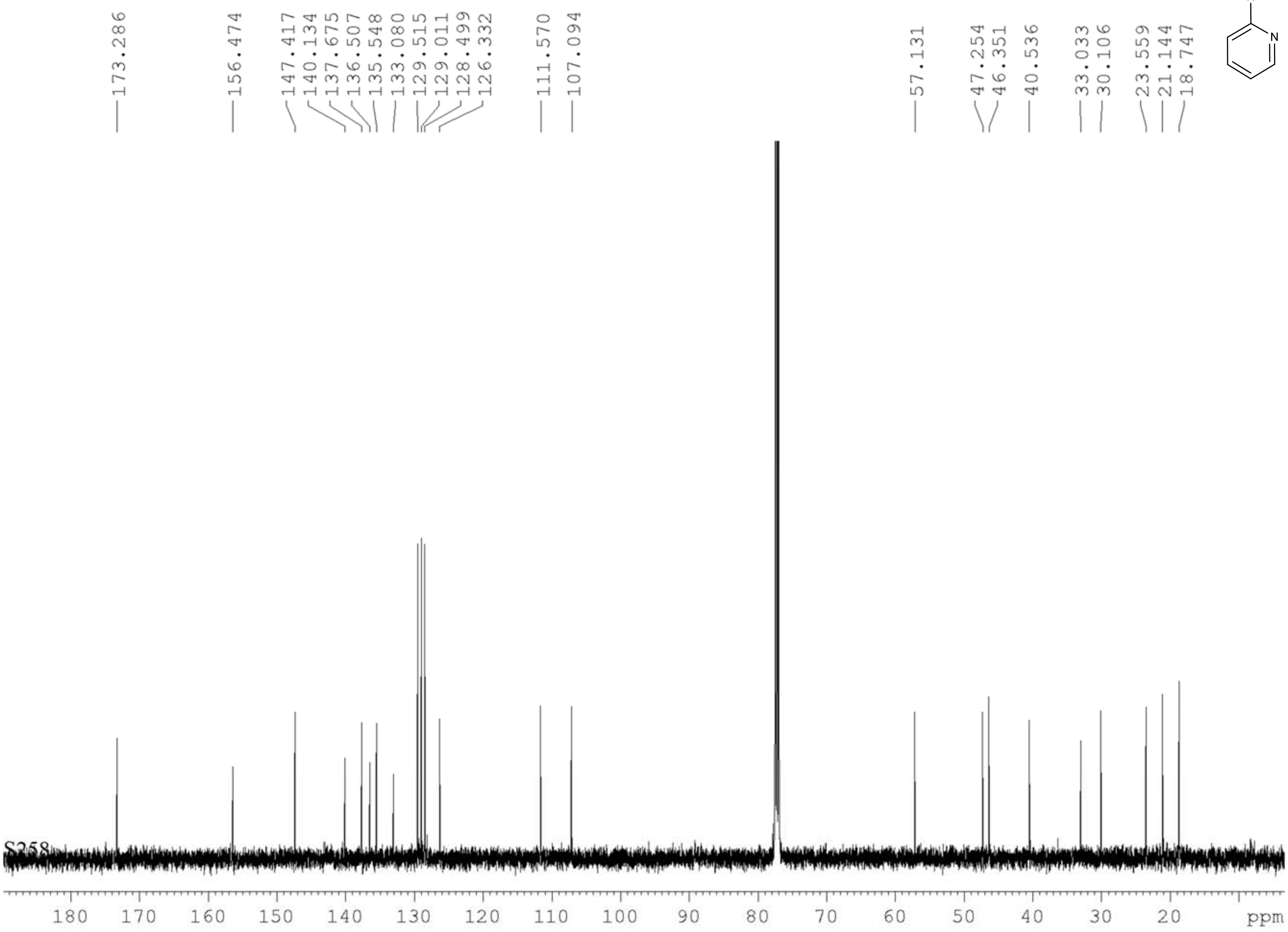

^{1}H NMR (400 MHz, $CDCl_3$) for **(*S*)-2-Benzyl-*N*-phenyl-3-((*S*)-pyrrolidin-2-yl)propenamide (5bg)**

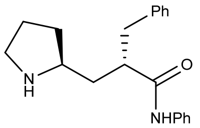

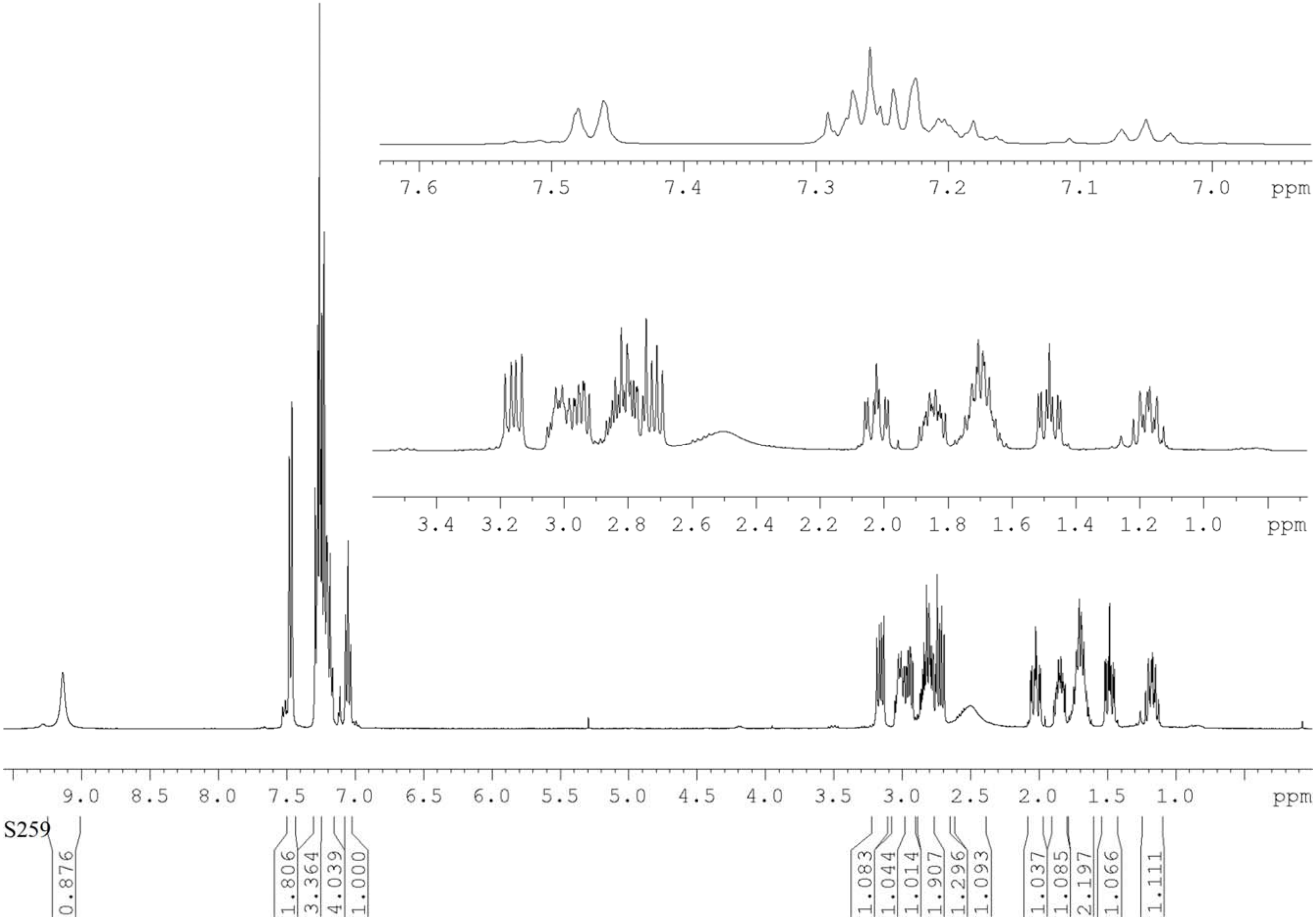

^{13}C NMR (101 MHz, $CDCl_3$) for ***(S)*-2-Benzyl-*N*-phenyl-3-(*(S)*-pyrrolidin-2-yl)propenamide (5bg)**

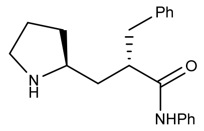

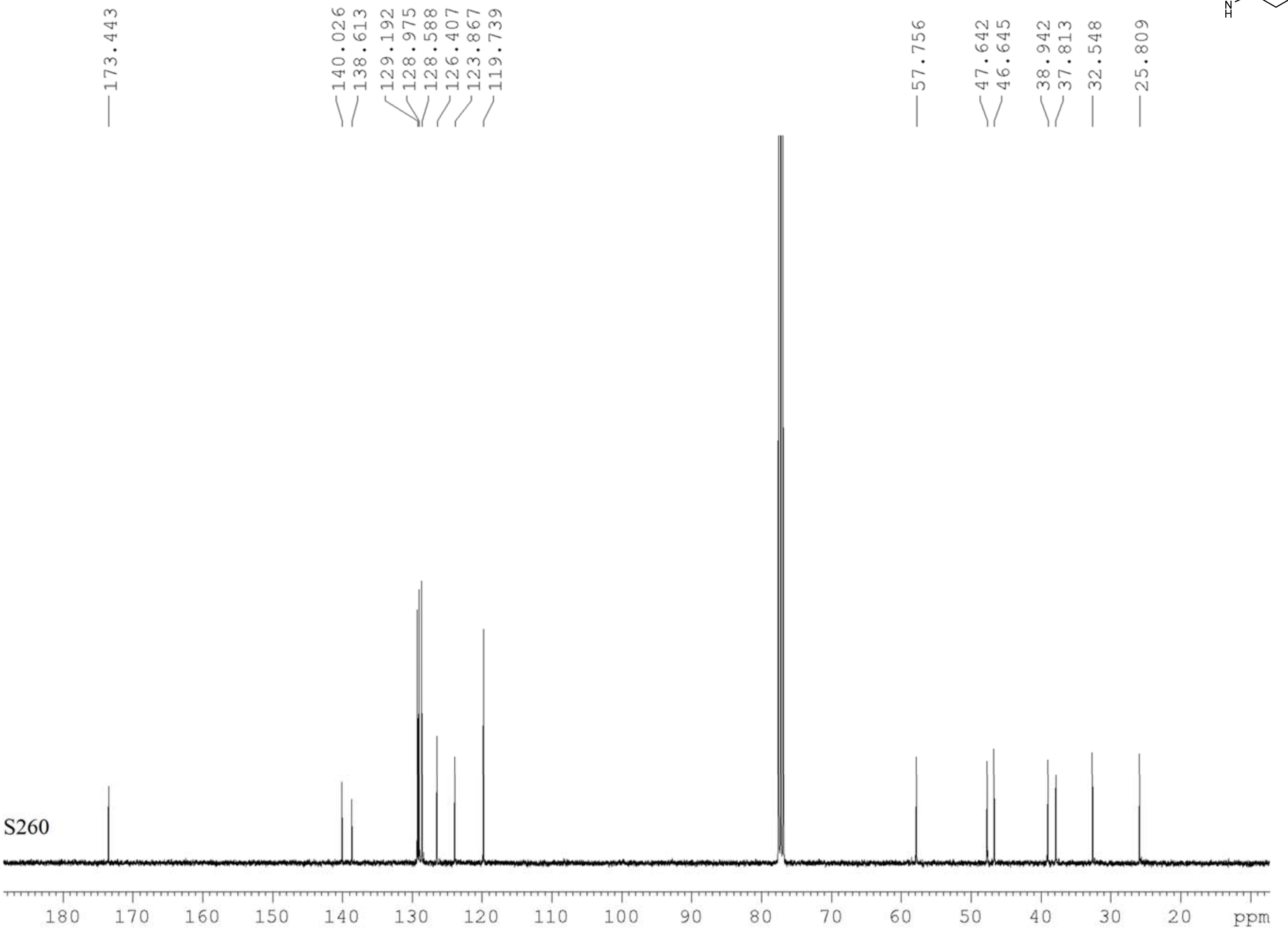

^{1}H NMR (500 MHz, D_2O) for **(*S*)-2-((*S*)-2-Carboxy-3-phenylpropyl)pyrrolidin-1-ium chloride (5bc)**

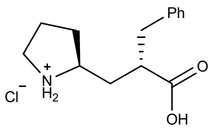

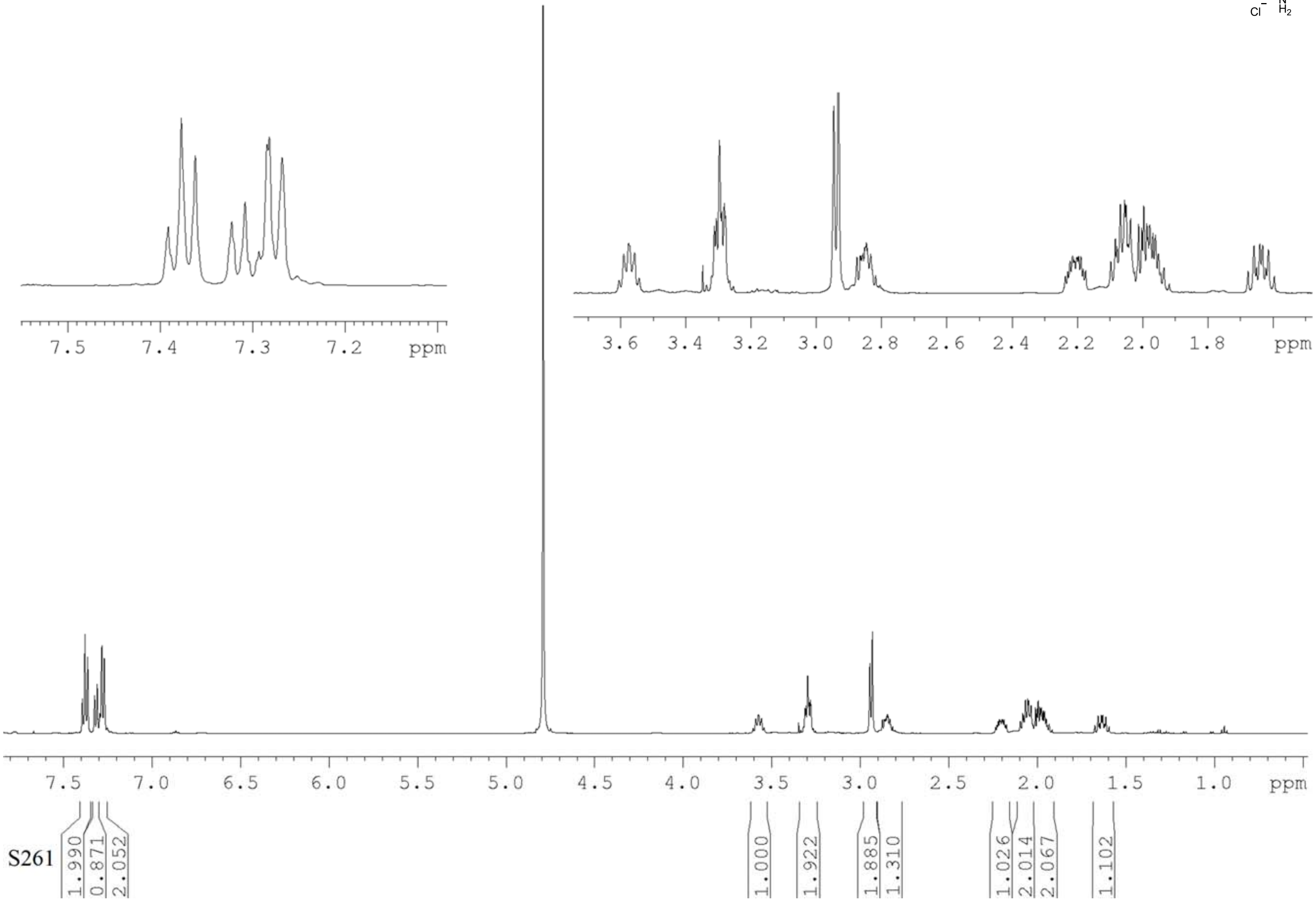

^{13}C NMR (126 MHz, D_2O) for **(*S*)-2-((*S*)-2-Carboxy-3-phenylpropyl)pyrrolidin-1-ium chloride (5bc)**

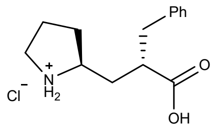

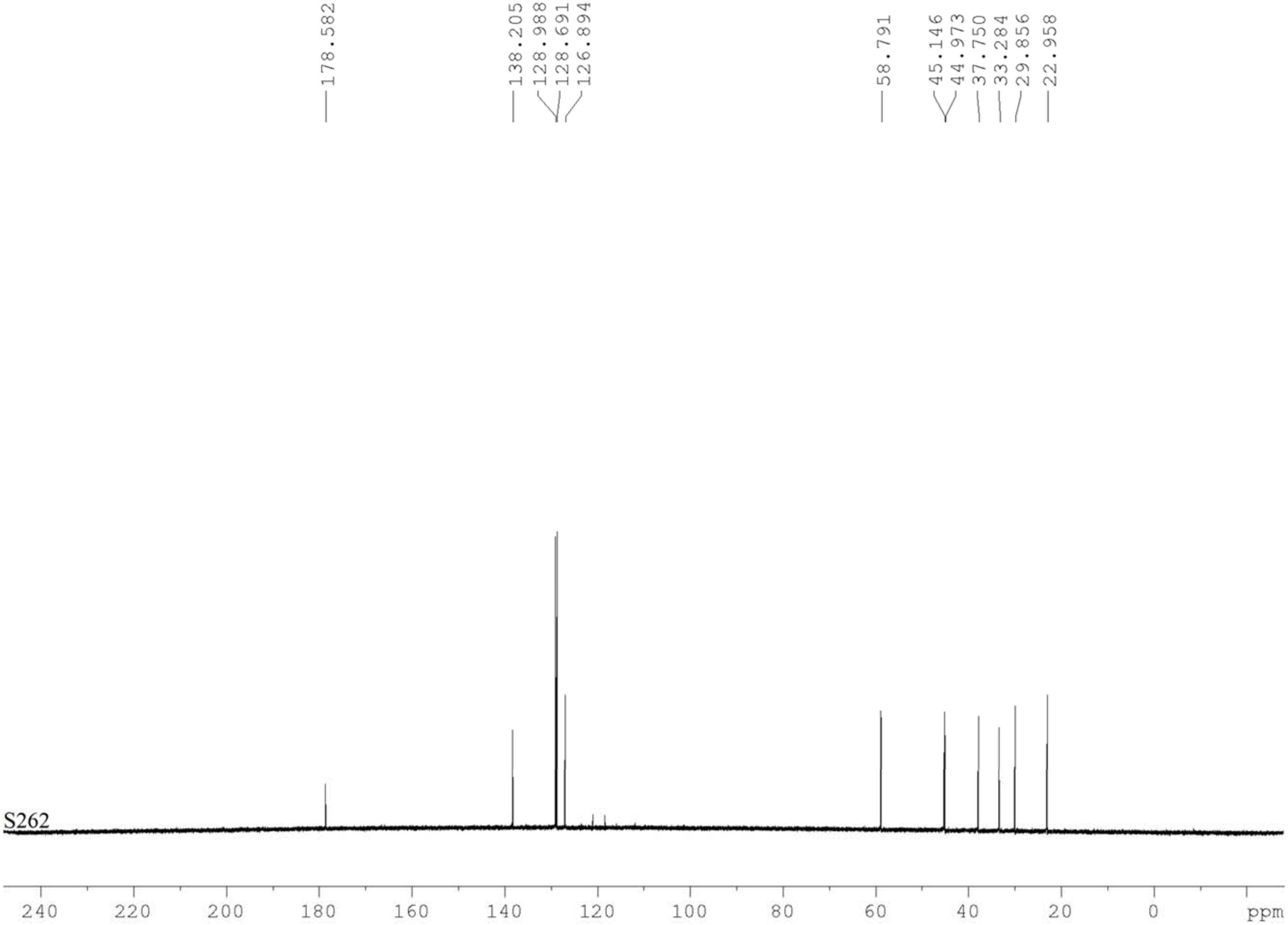

^{1}H NMR (500 MHz, $CDCl_3$) for **(2*S*,7a*S*)-2-Benzylhexahydro-3*H*-pyrrolizin-3-one (5be)**

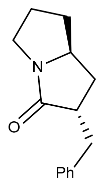

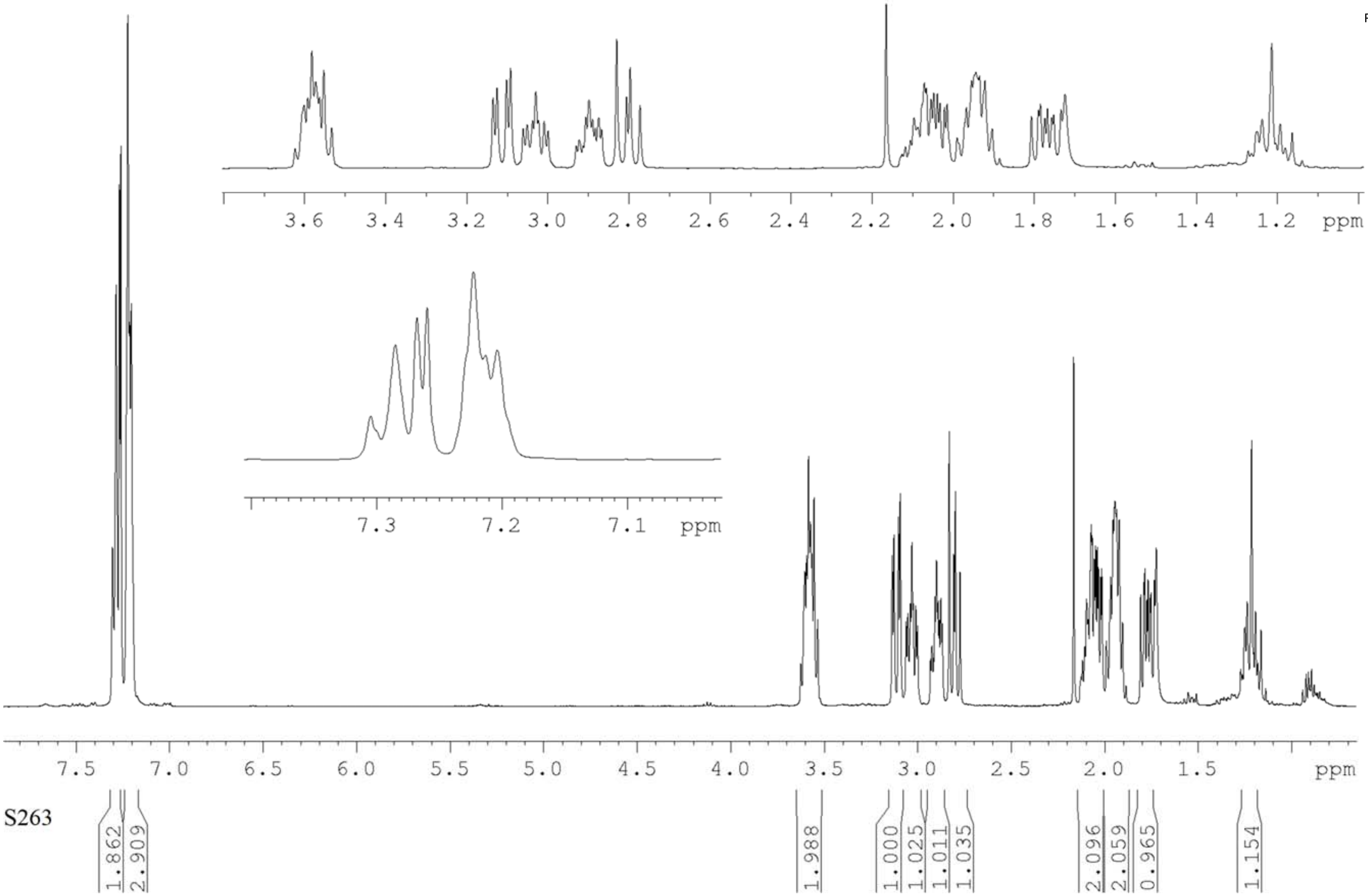

^{13}C NMR (126 MHz, $CDCl_3$) for **(2*S*,7a*S*)-2-Benzylhexahydro-3*H*-pyrrolizin-3-one (5be)**

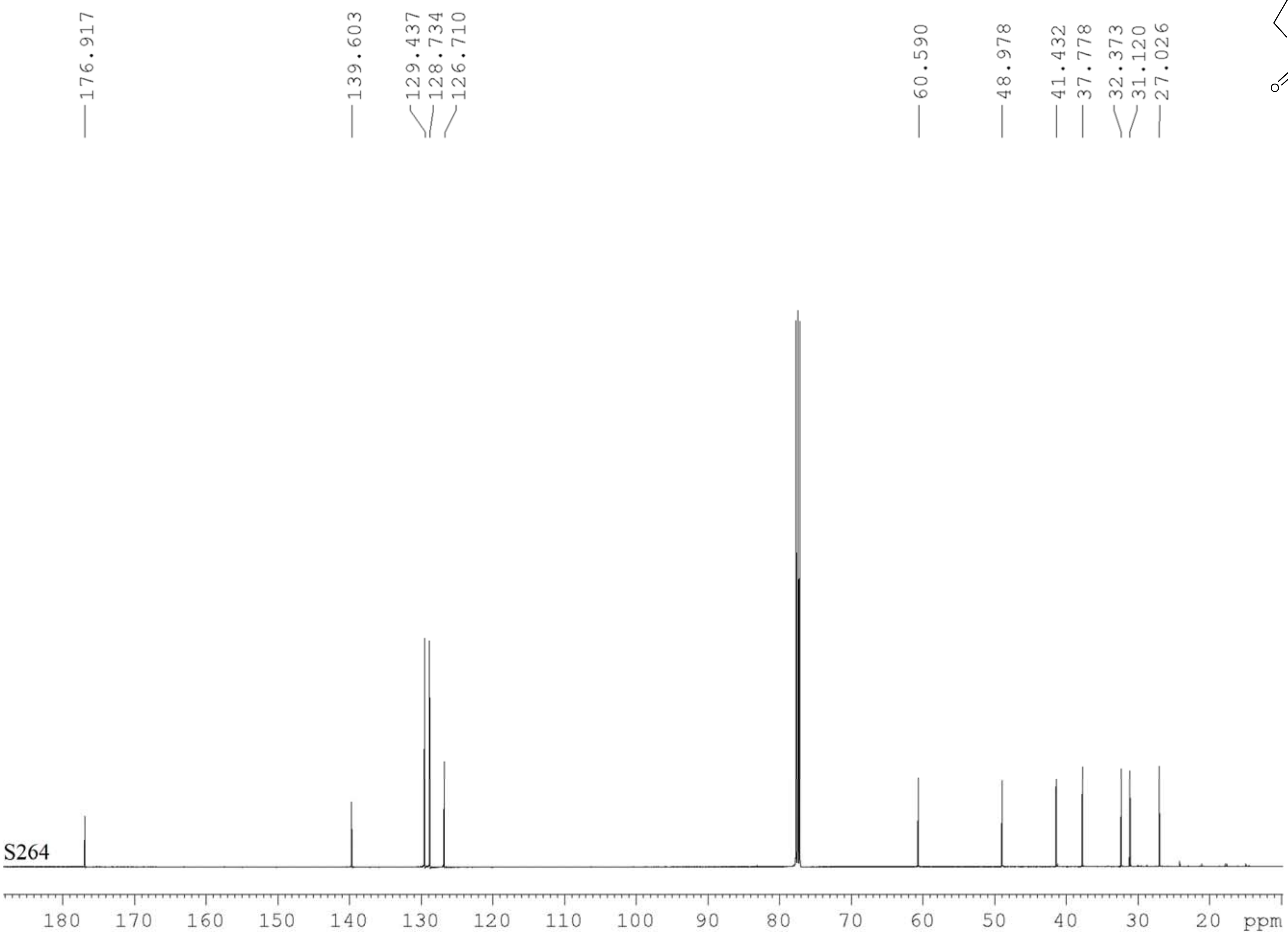

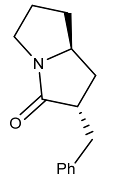

NOESY NMR (500 MHz, $CDCl_3$) for **(2*S*,7a*S*)-2-Benzylhexahydro-3*H*-pyrrolizin-3-one (5be)**

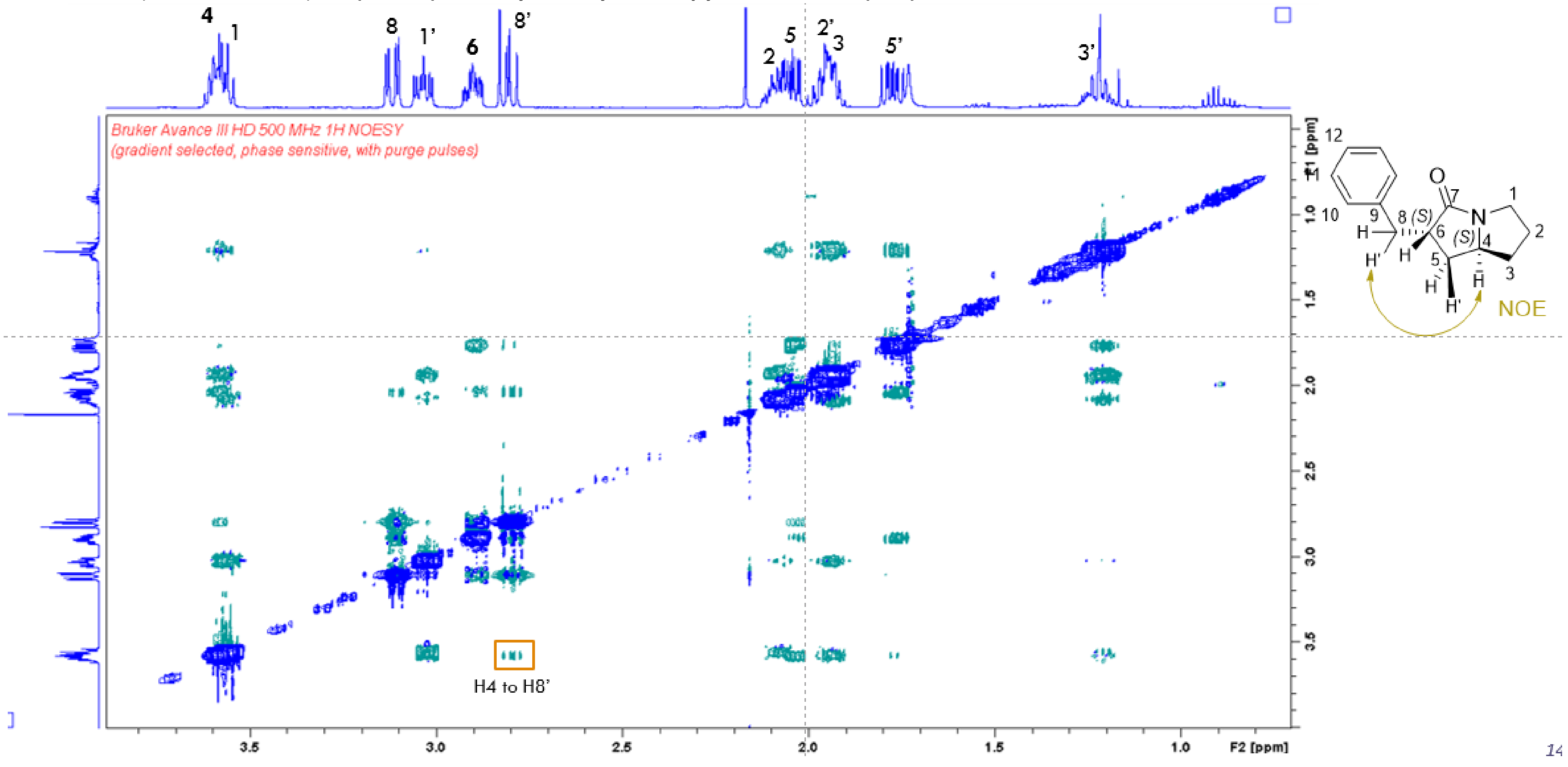

NOESY NMR (500 MHz, $CDCl_3$) for **(2*S*,7a*S*)-2-Benzylhexahydro-3*H*-pyrrolizin-3-one (5be)**

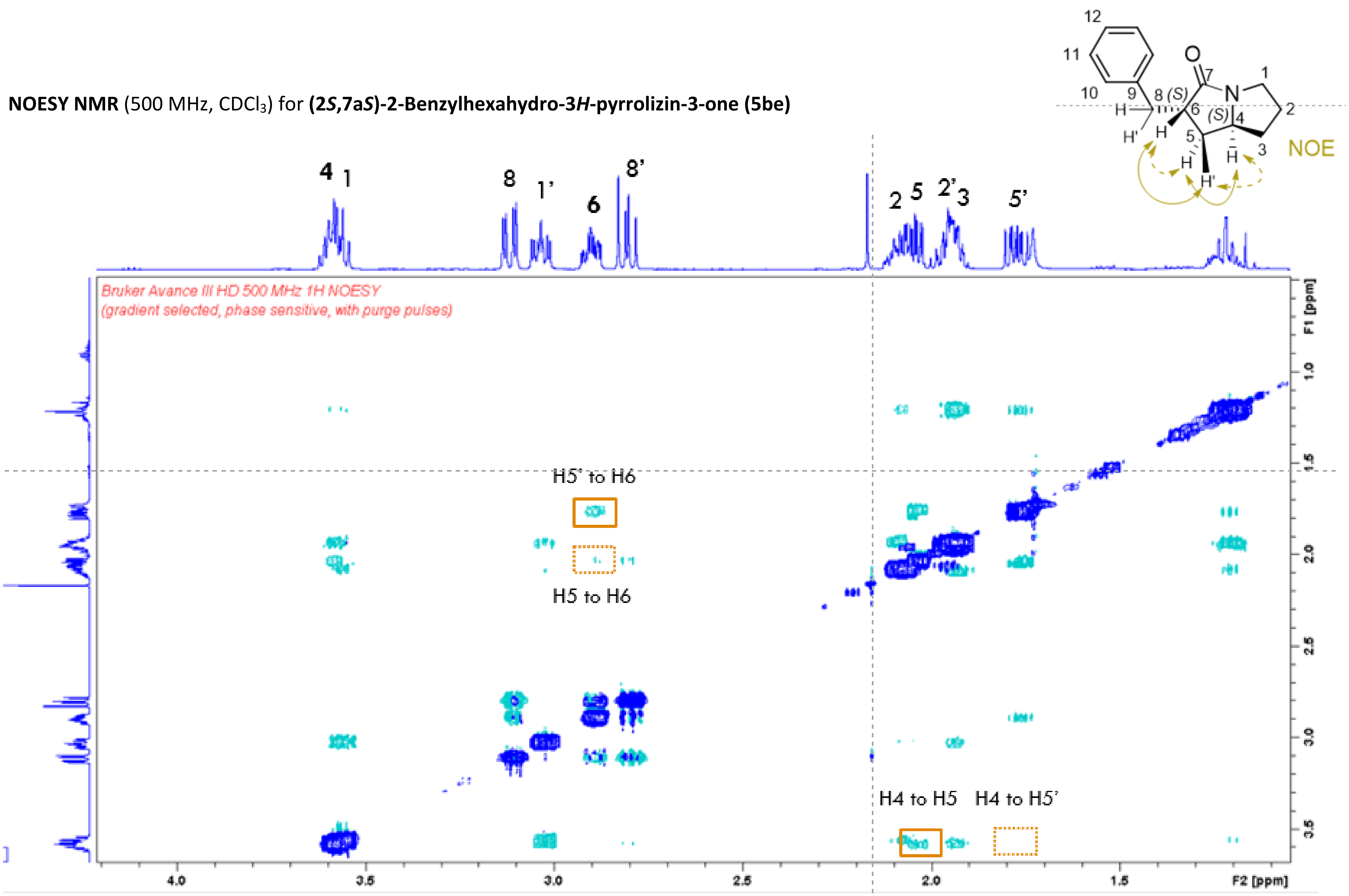

^{1}H NMR (400 MHz, $CDCl_3$) for **(*R*)-2-Benzyl-*N*-phenyl-3-((*S*)-pyrrolidin-2-yl)propenamide (5bh)**

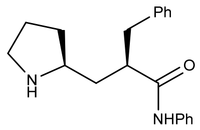

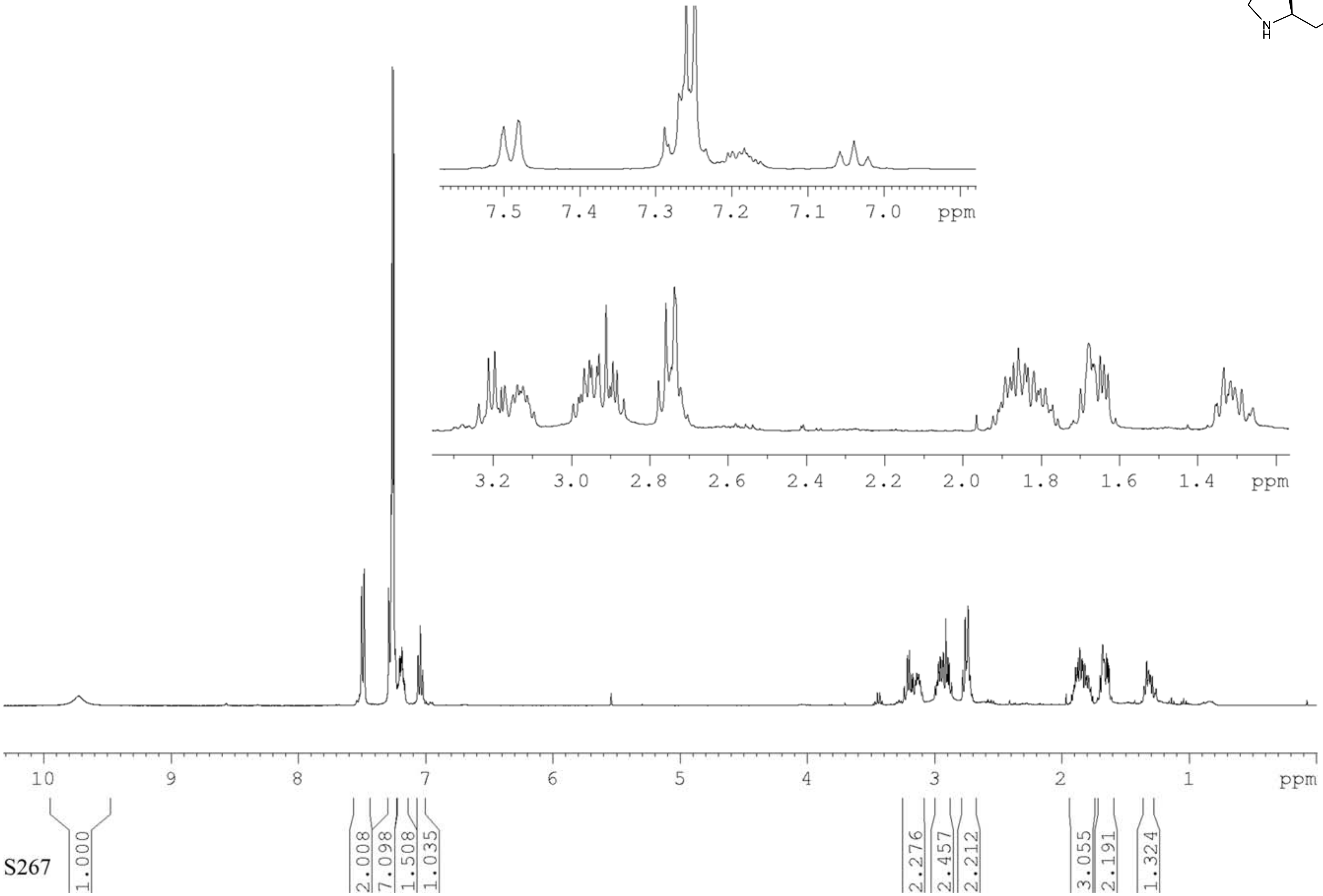

^{13}C NMR (126 MHz, $CDCl_3$) for **(*R*)-2-Benzyl-*N*-phenyl-3-((*S*)-pyrrolidin-2-yl)propenamide (5bh)**

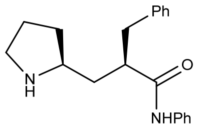

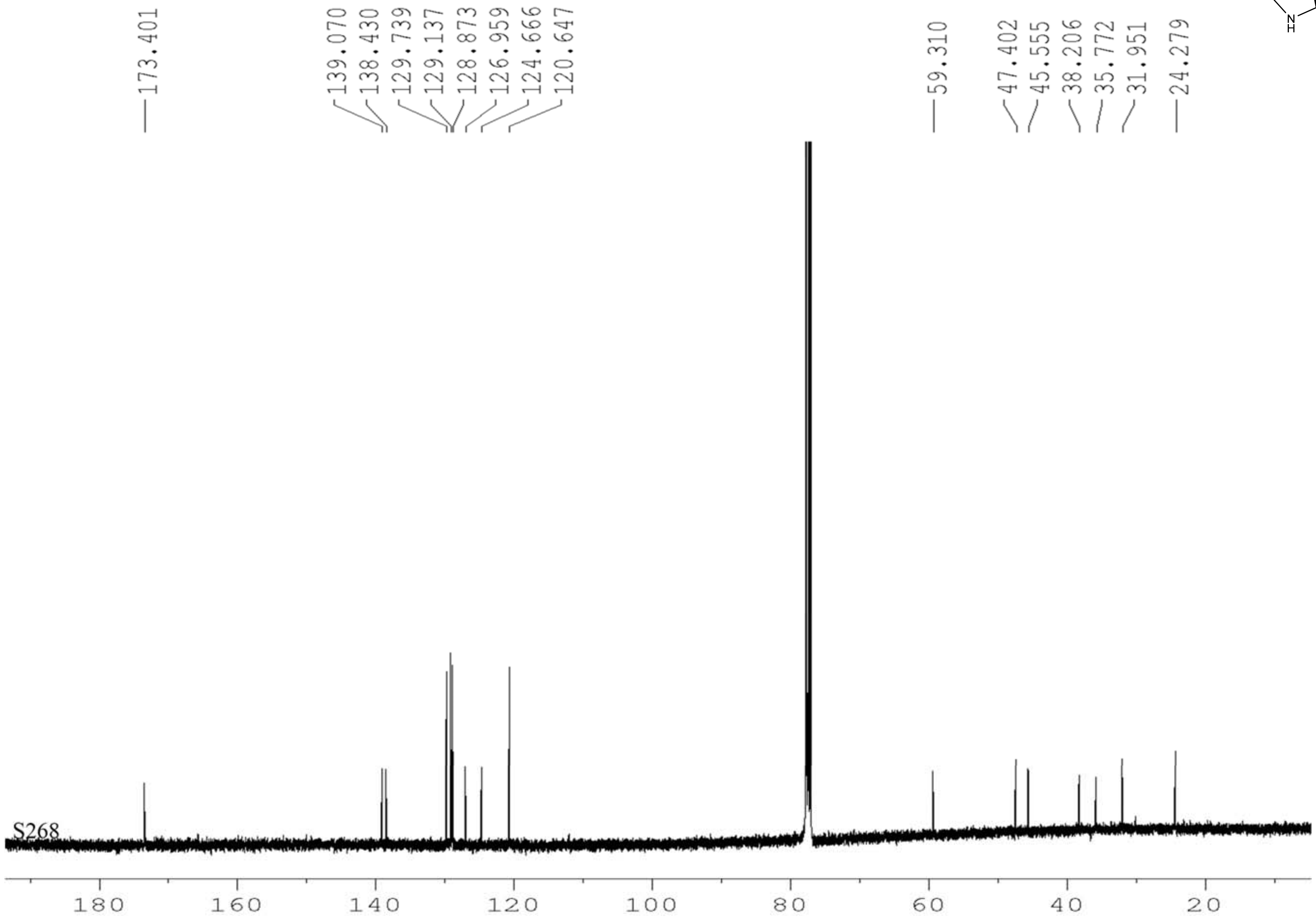

^{1}H NMR (500 MHz, D_2O) for **(*S*)-2-((*R*)-2-Carboxy-3-phenylpropyl)pyrrolidin-1-ium chloride (5bd)**

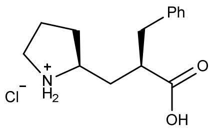

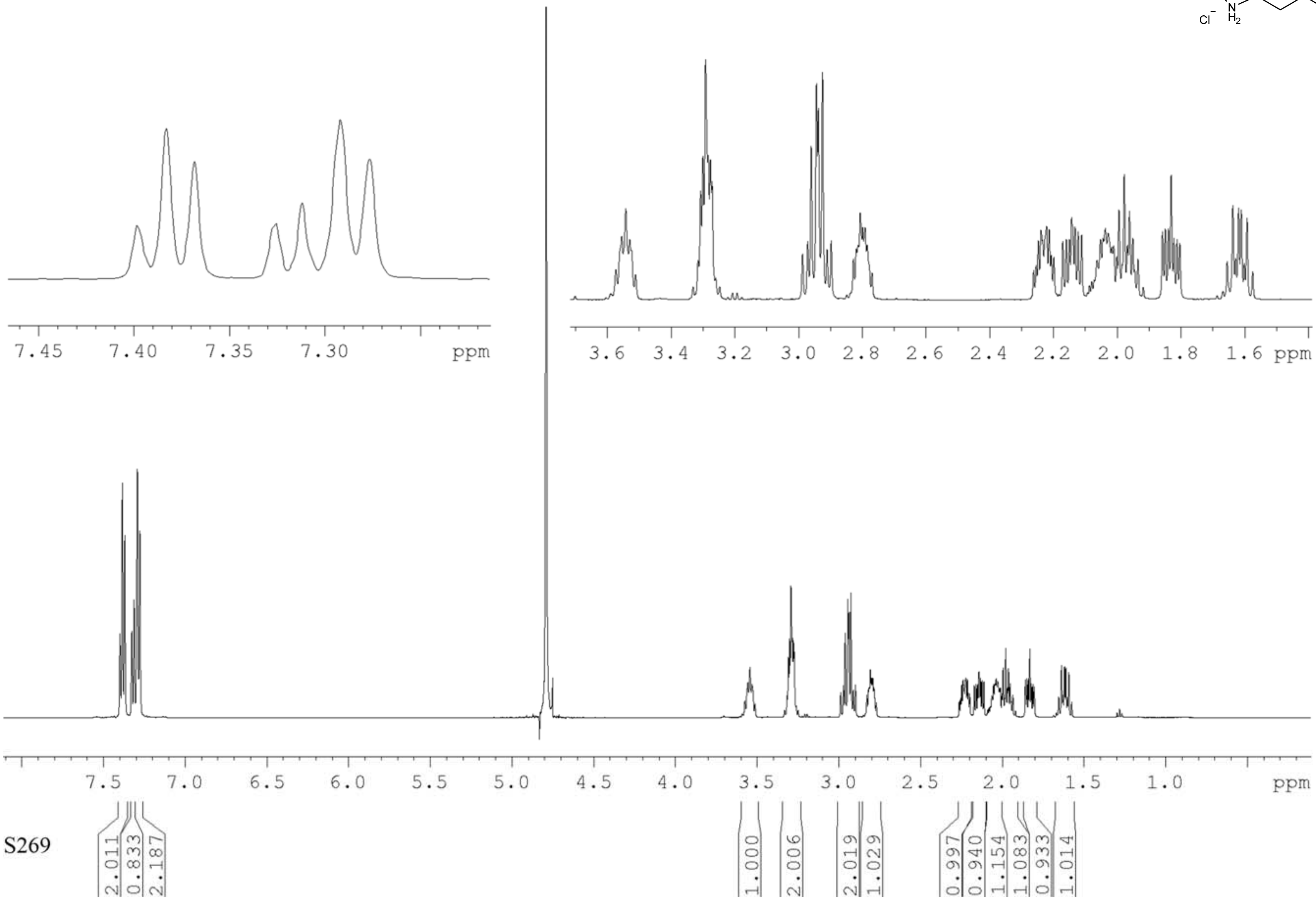

^{13}C NMR (126 MHz, D_2O) for **(*S*)-2-((*R*)-2-Carboxy-3-phenylpropyl)pyrrolidin-1-ium chloride (5bd)**

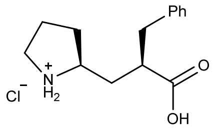

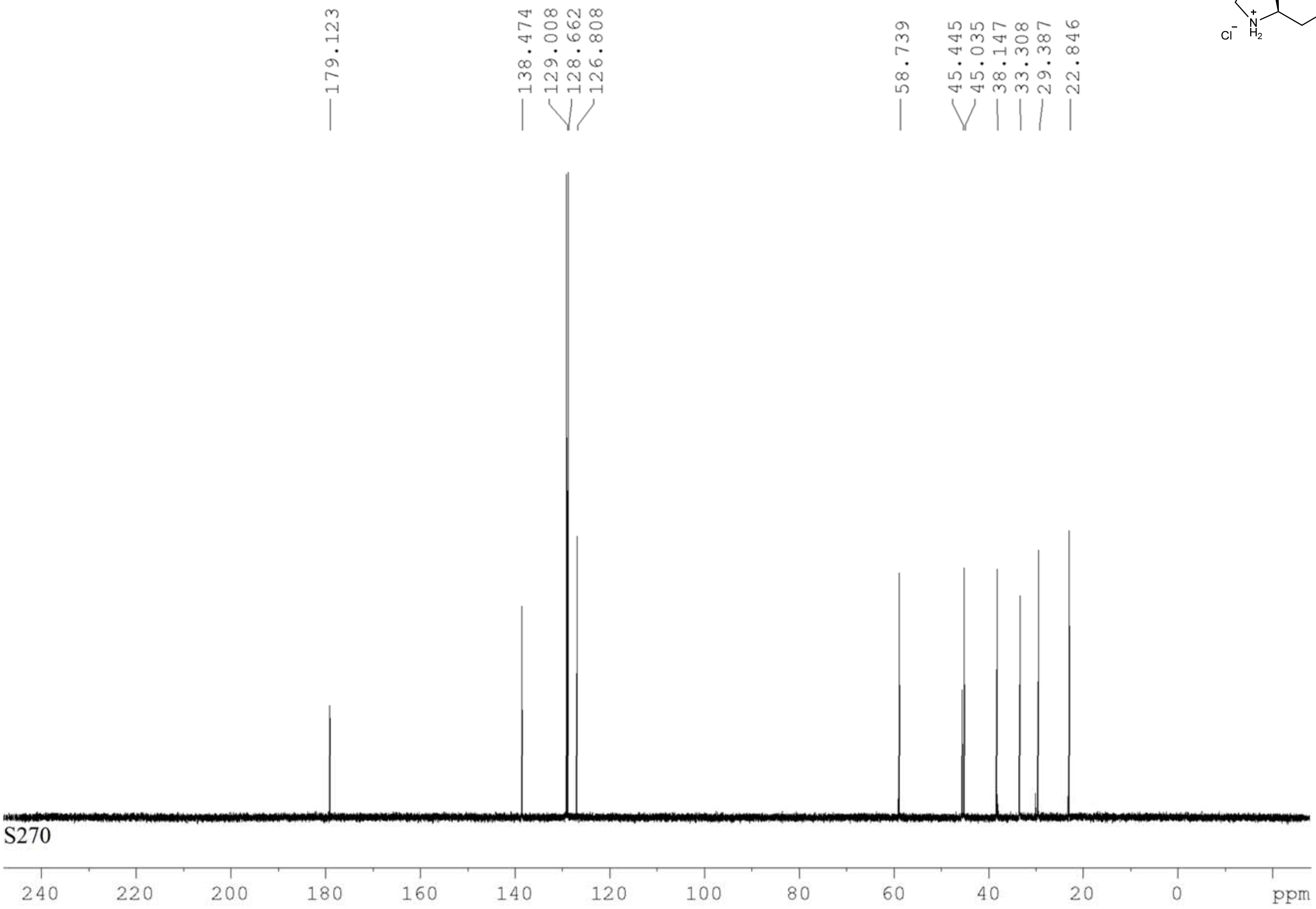

^{1}H NMR (500 MHz, $CDCl_3$) for **(2*R*,7a*S*)-2-Benzylhexahydro-3*H*-pyrrolizin-3-one (5bf)**

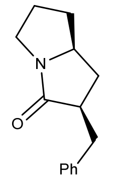

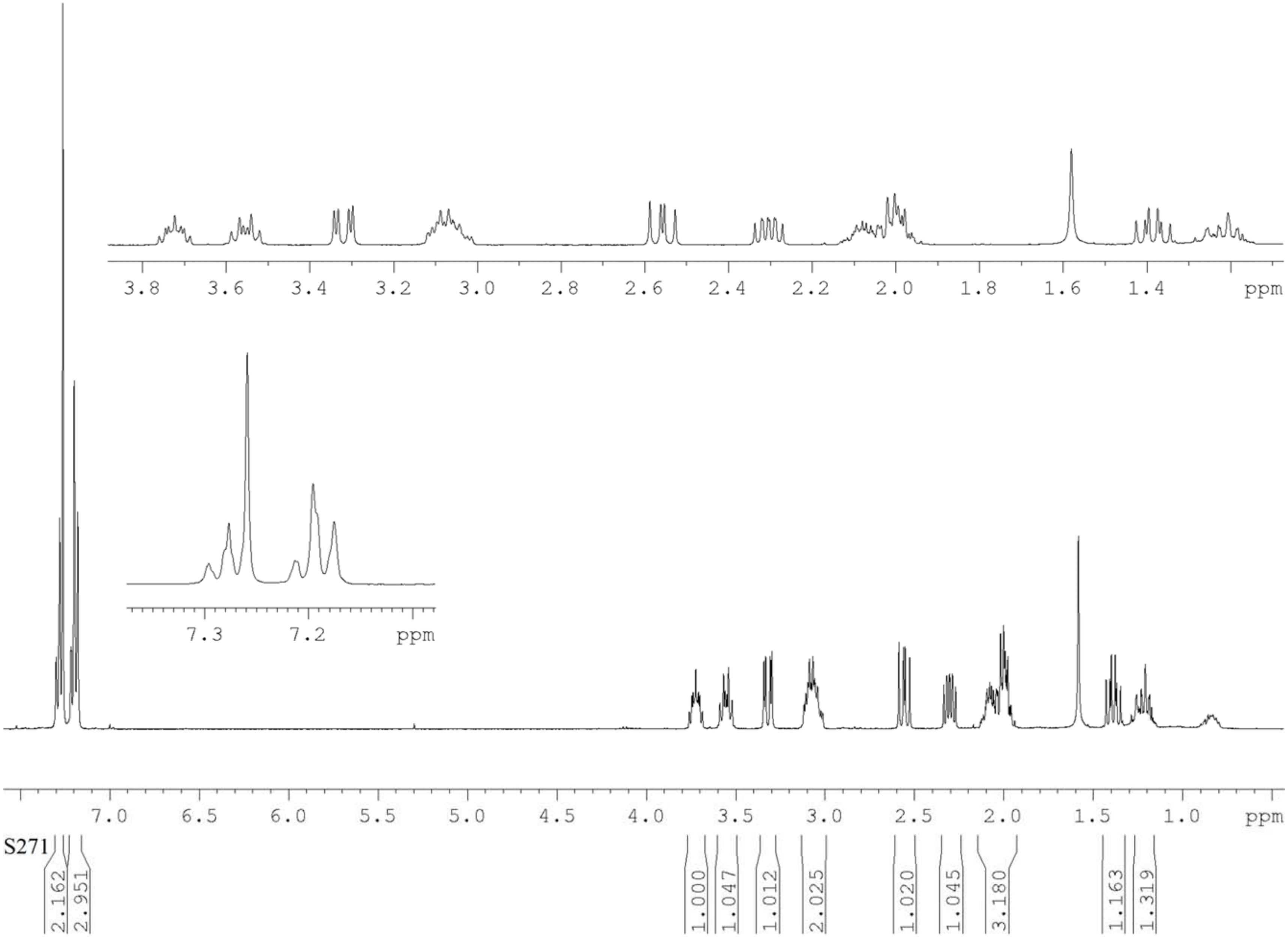

^{13}C NMR (126 MHz, $CDCl_3$) for **(2*R*,7a*S*)-2-Benzylhexahydro-3*H*-pyrrolizin-3-one (5bf)**

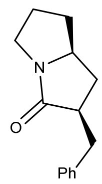

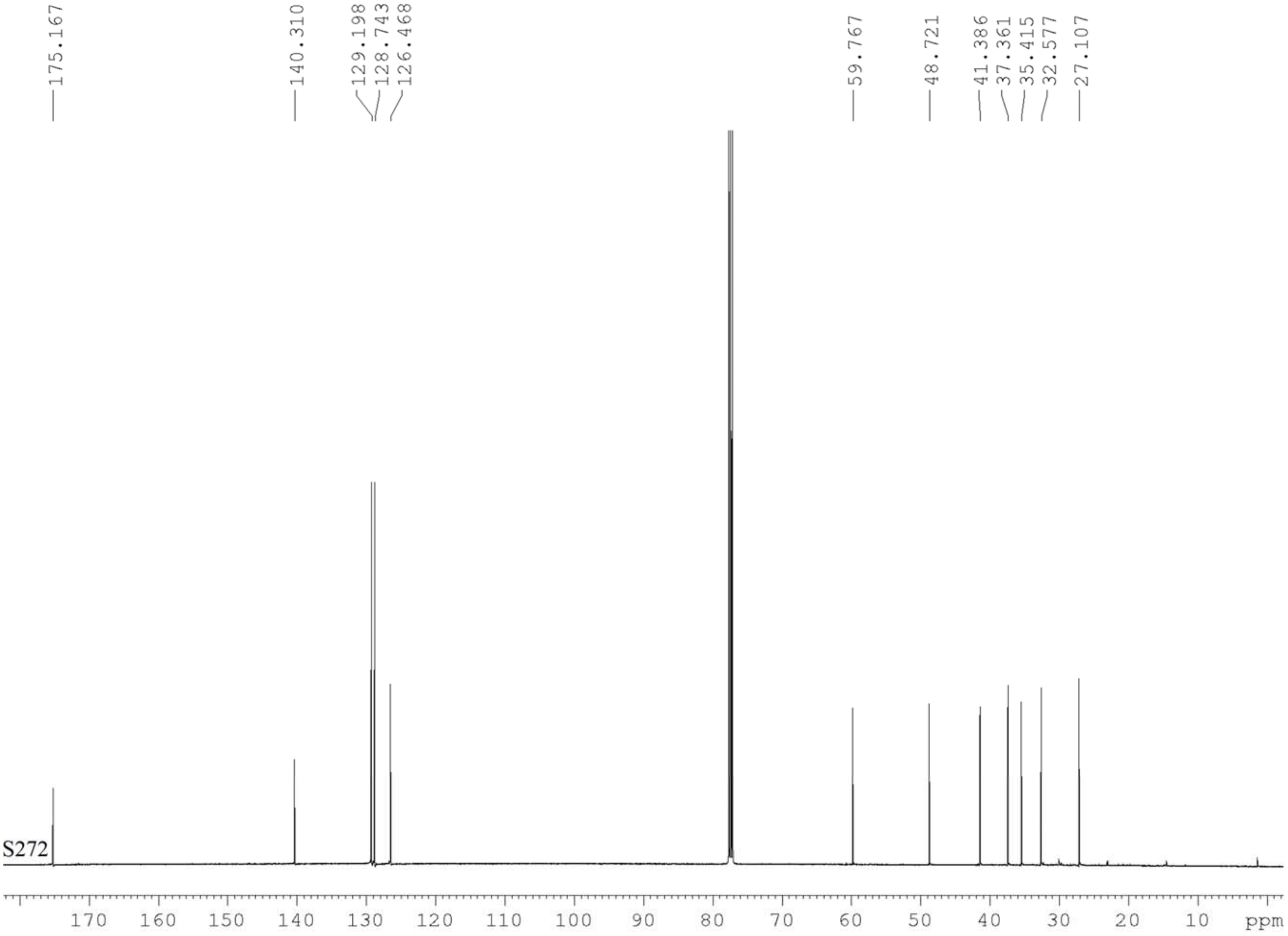

NOESY NMR (500 MHz, $CDCl_3$) for **(2*R*,7a*S*)-2-Benzylhexahydro-3*H*-pyrrolizin-3-one (5bf)**

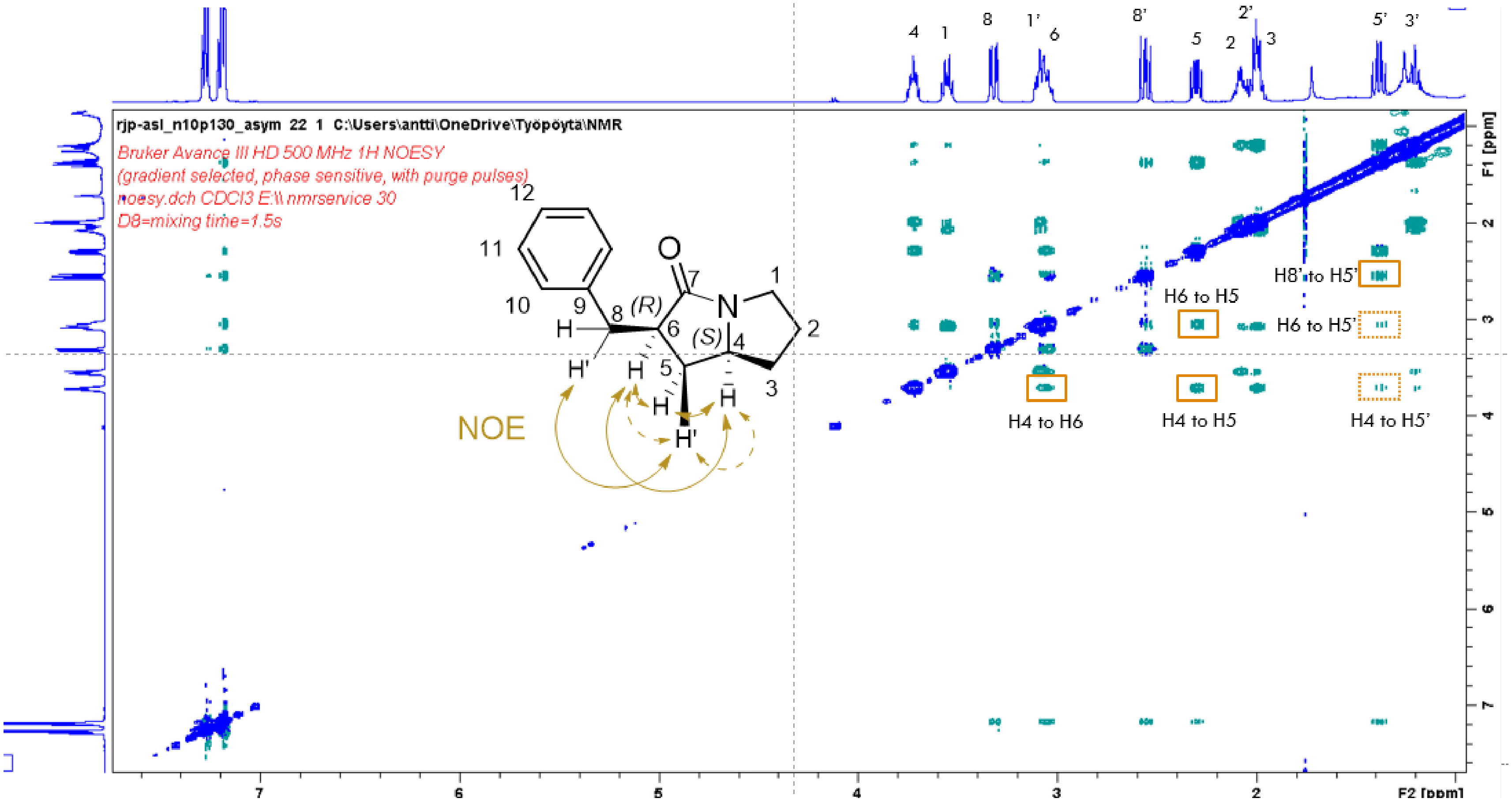

NOESY NMR (500 MHz, $CDCl_3$) for **(2*R*,7a*S*)-2-Benzylhexahydro-3*H*-pyrrolizin-3-one (5bf)**

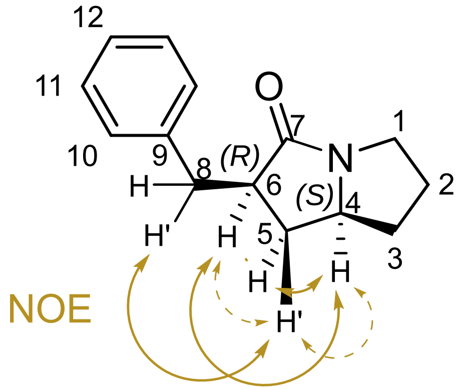

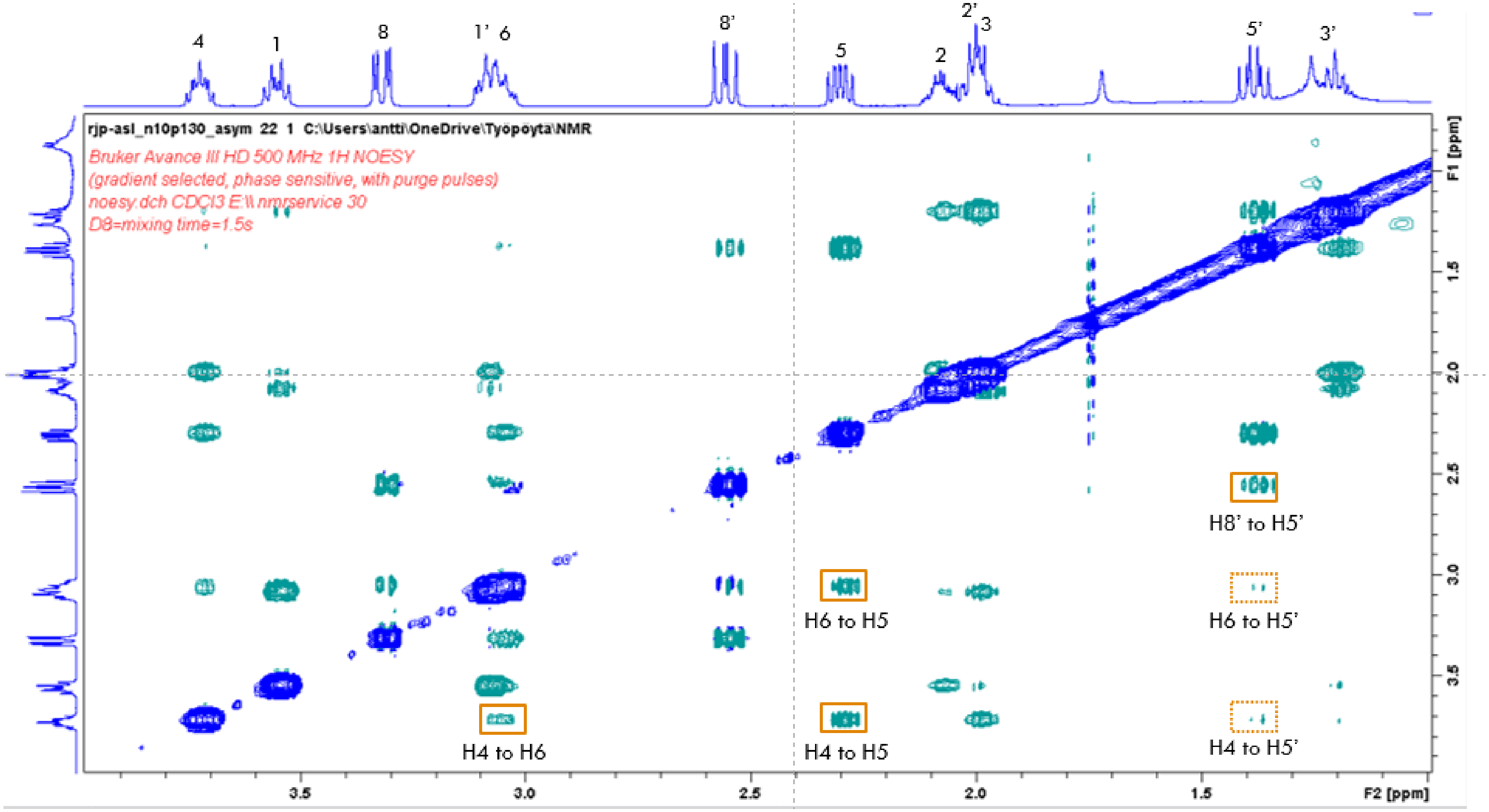

^{1}H NMR (500 MHz, $CDCl_3$) for ***(R)*-*N*-Phenyl-3-(*(S)*-pyrrolidin-2-yl)butanamide (6qa)**

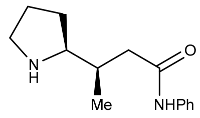

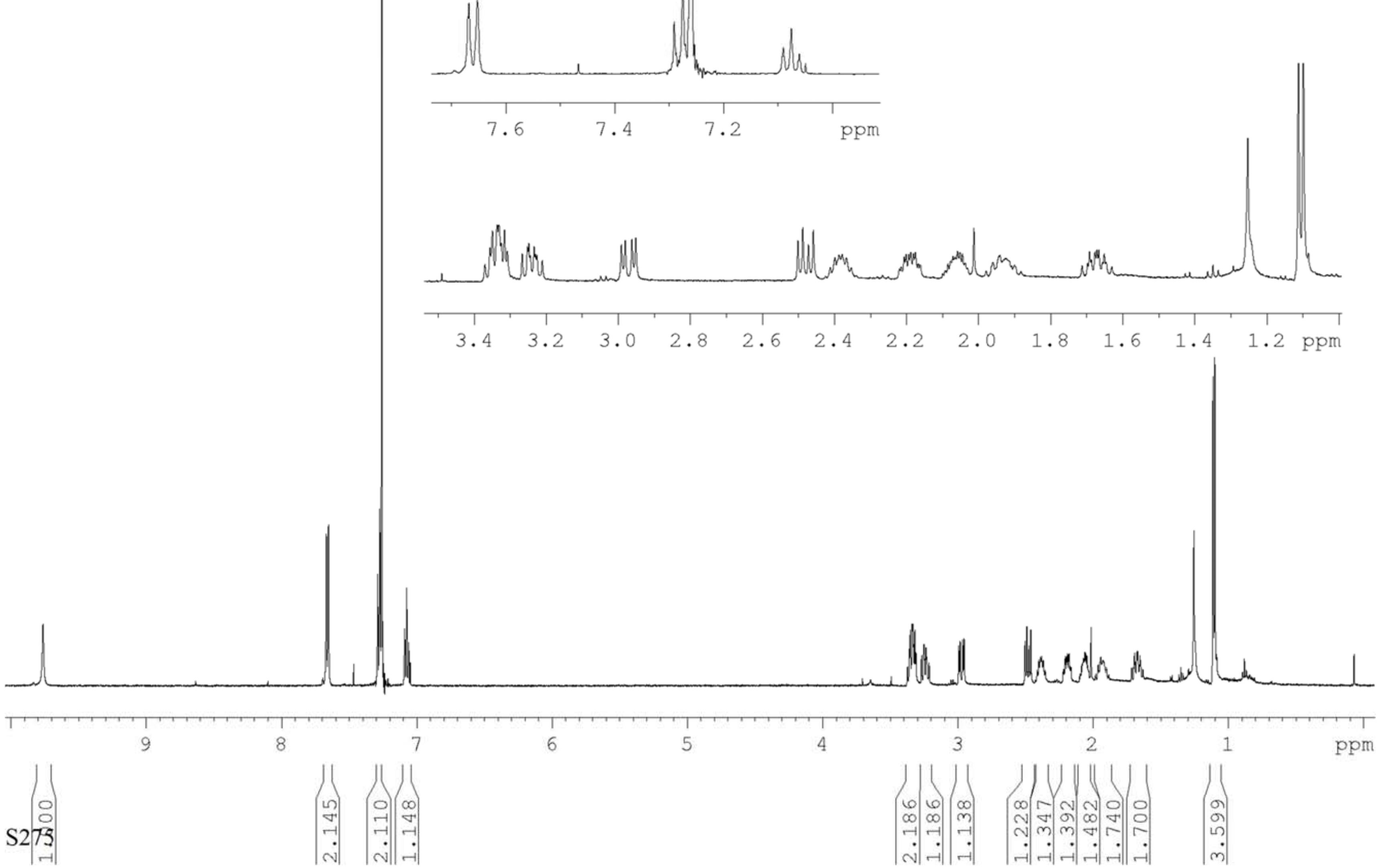

^{1}H NMR (500 MHz, $CDCl_3$) f**or (*R*)-*N*-Phenyl-3-((*S*)-pyrrolidin-2-yl)butanamide (6qa)**

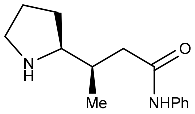

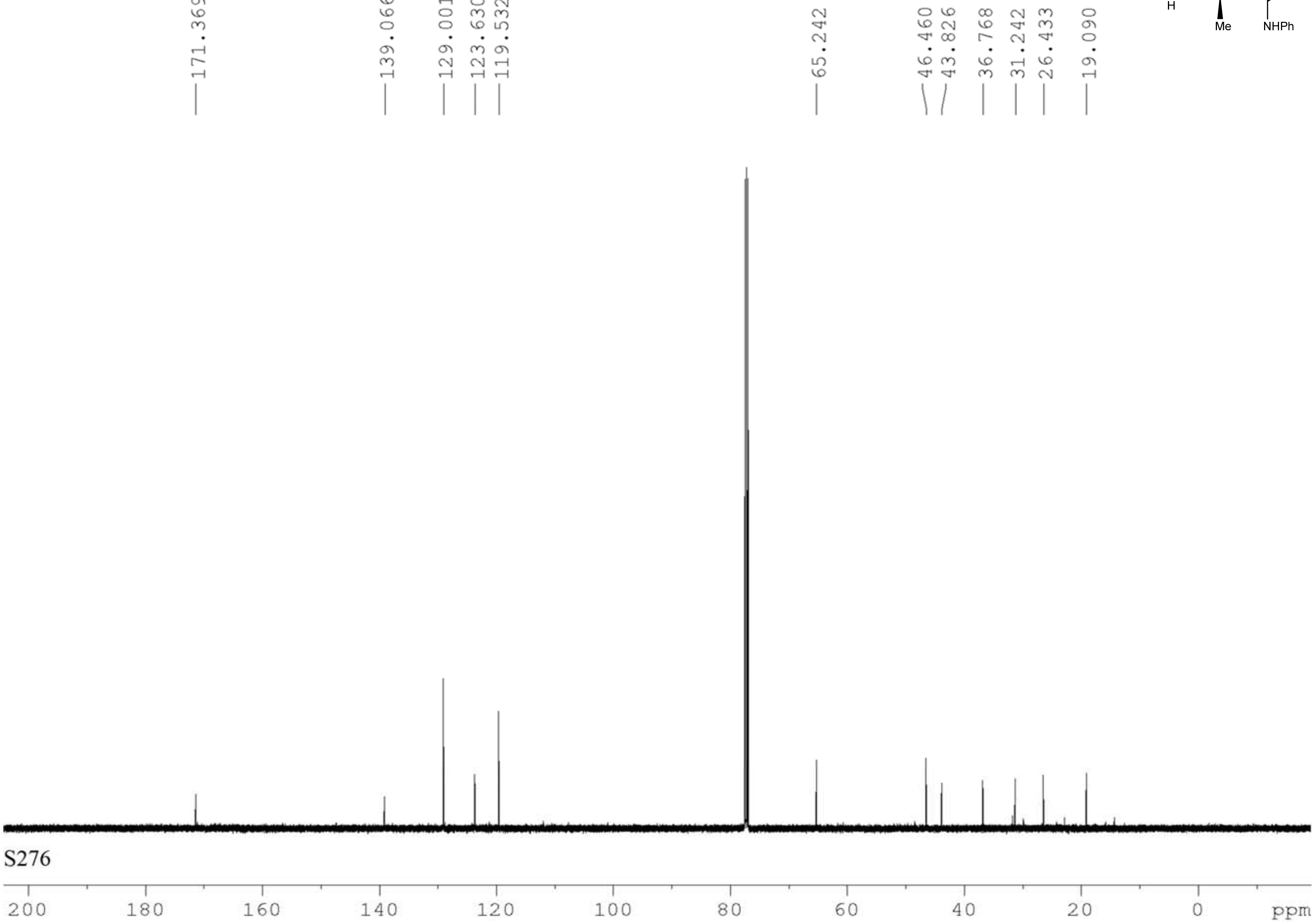

^{1}H NMR (700 MHz, $CDCl_3$) for **(1*R*,7a*S*)-1-Methylhexahydro-3H-pyrrolizin-3-one (6qb)**

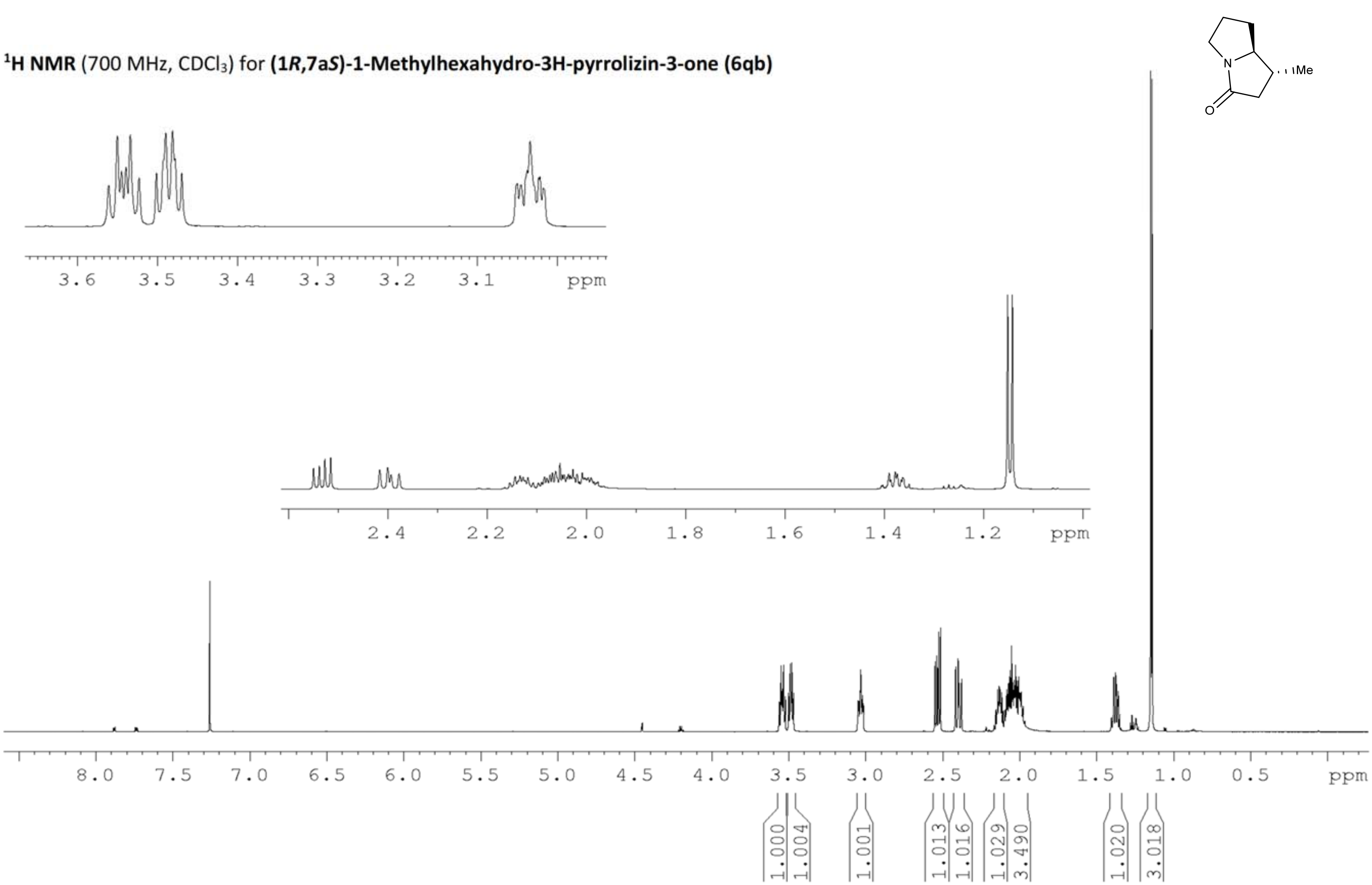

^{13}C NMR (101 MHz, $CDCl_3$) for **(1*R*,7a*S*)-1-Methylhexahydro-3H-pyrrolizin-3-one (6qb)**

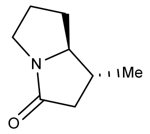

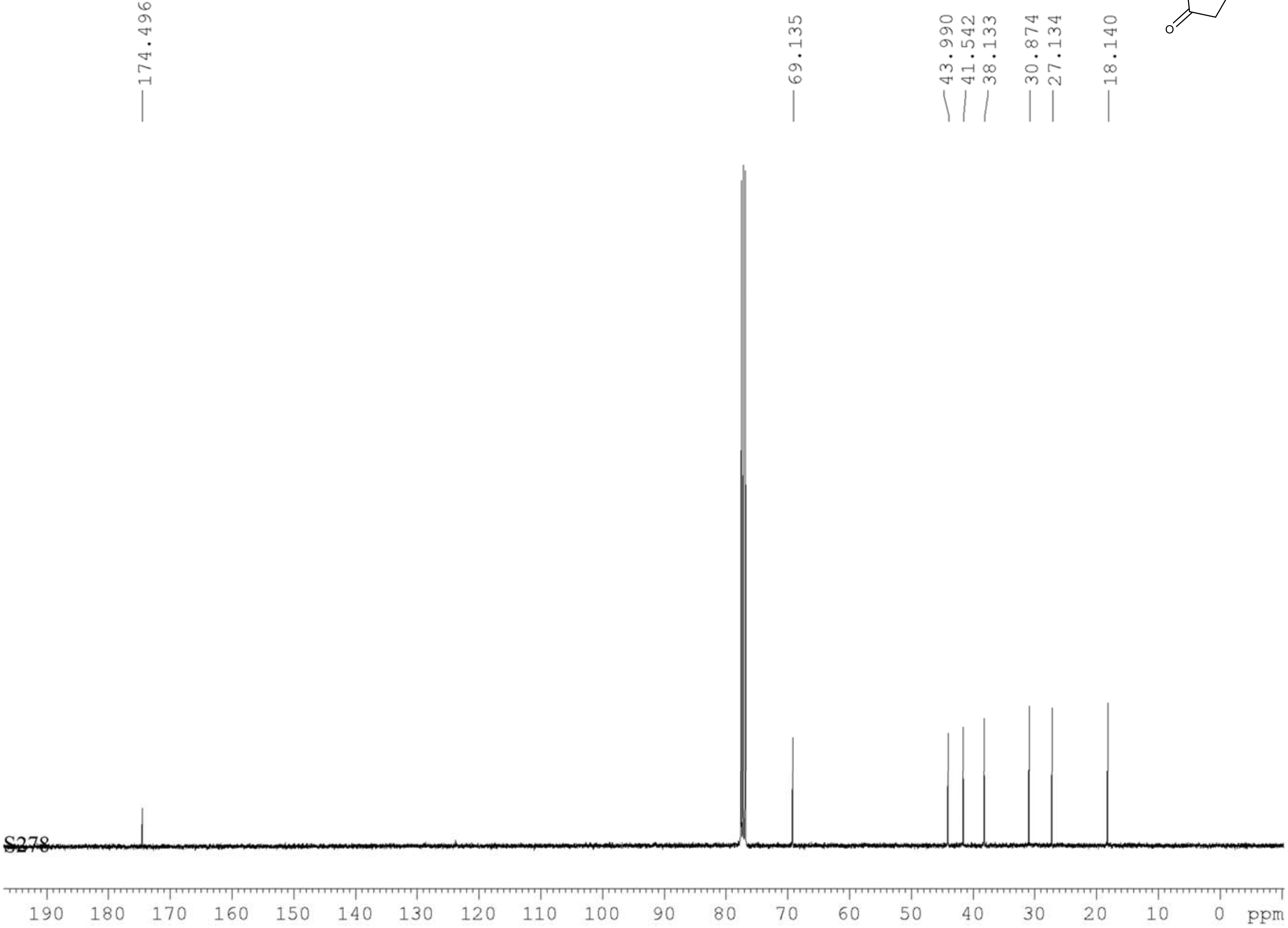

NOESY NMR (500 MHz, $CDCl_3$) for **(1*R*,7a*S*)-1-Methylhexahydro-3H-pyrrolizin-3-one (6qb)**

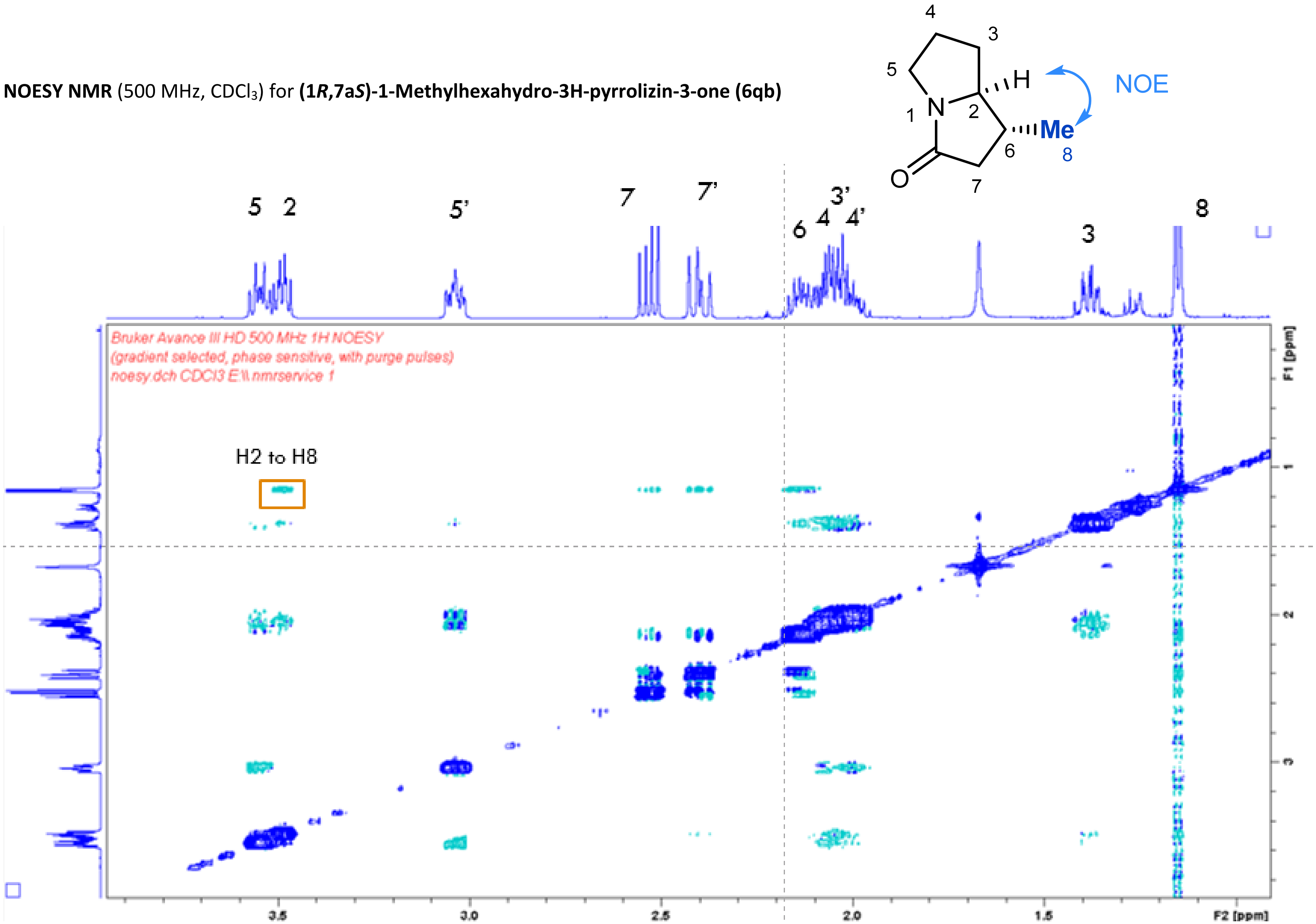

NOESY NMR (500 MHz, $CDCl_3$) for **(1*R*,7a*S*)-1-Methylhexahydro-3H-pyrrolizin-3-one (6qb)**

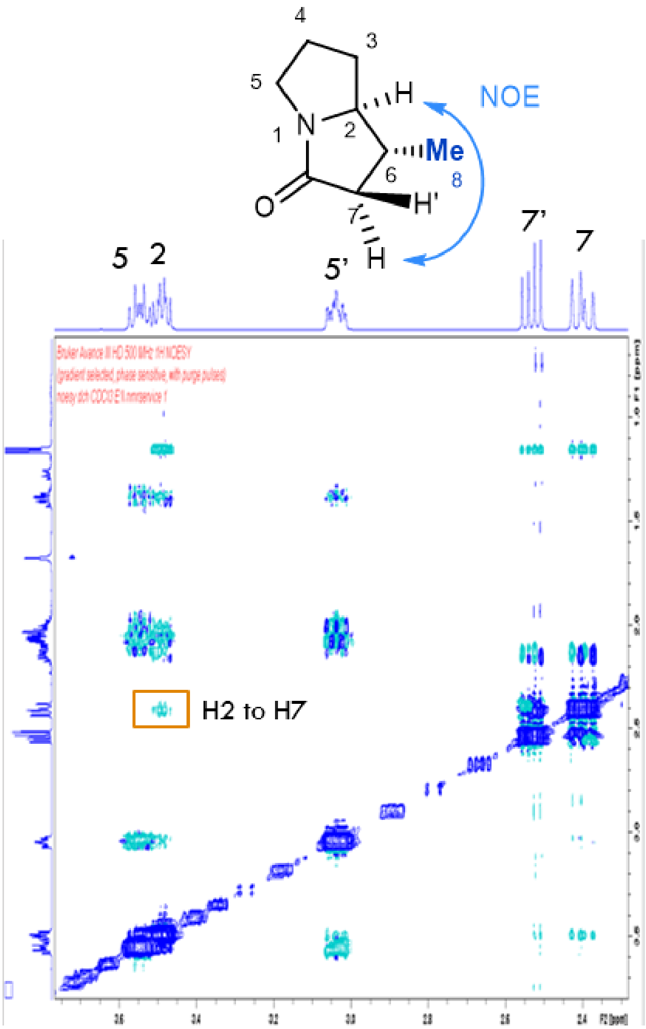

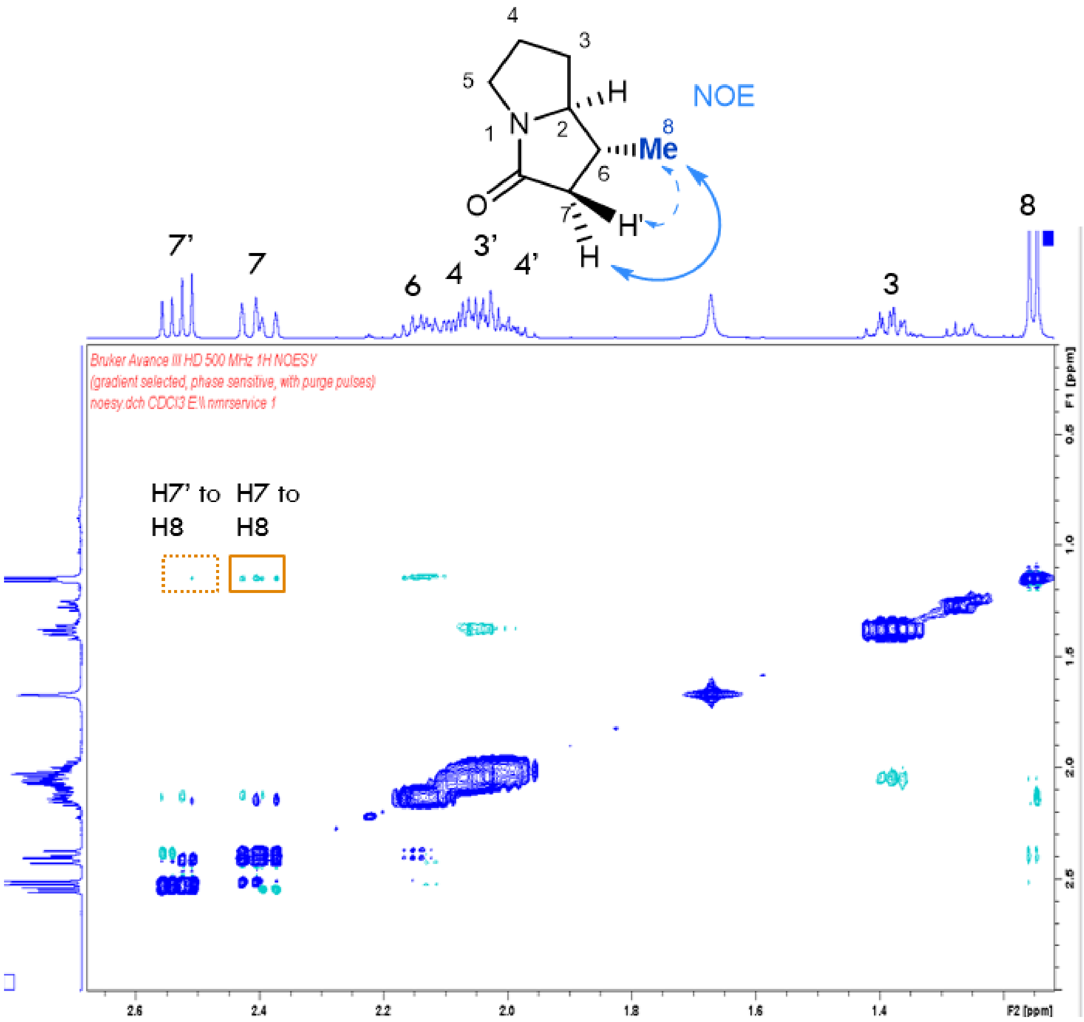

^{1}H NMR (500 MHz, MeOD : D_2O) for **(*R*)-3-(Aminomethyl)-5-methylhexanoic acid hydrochloride ((*R*)-Pregabalin HCl salt) (6pa)**

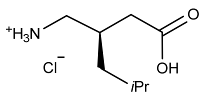

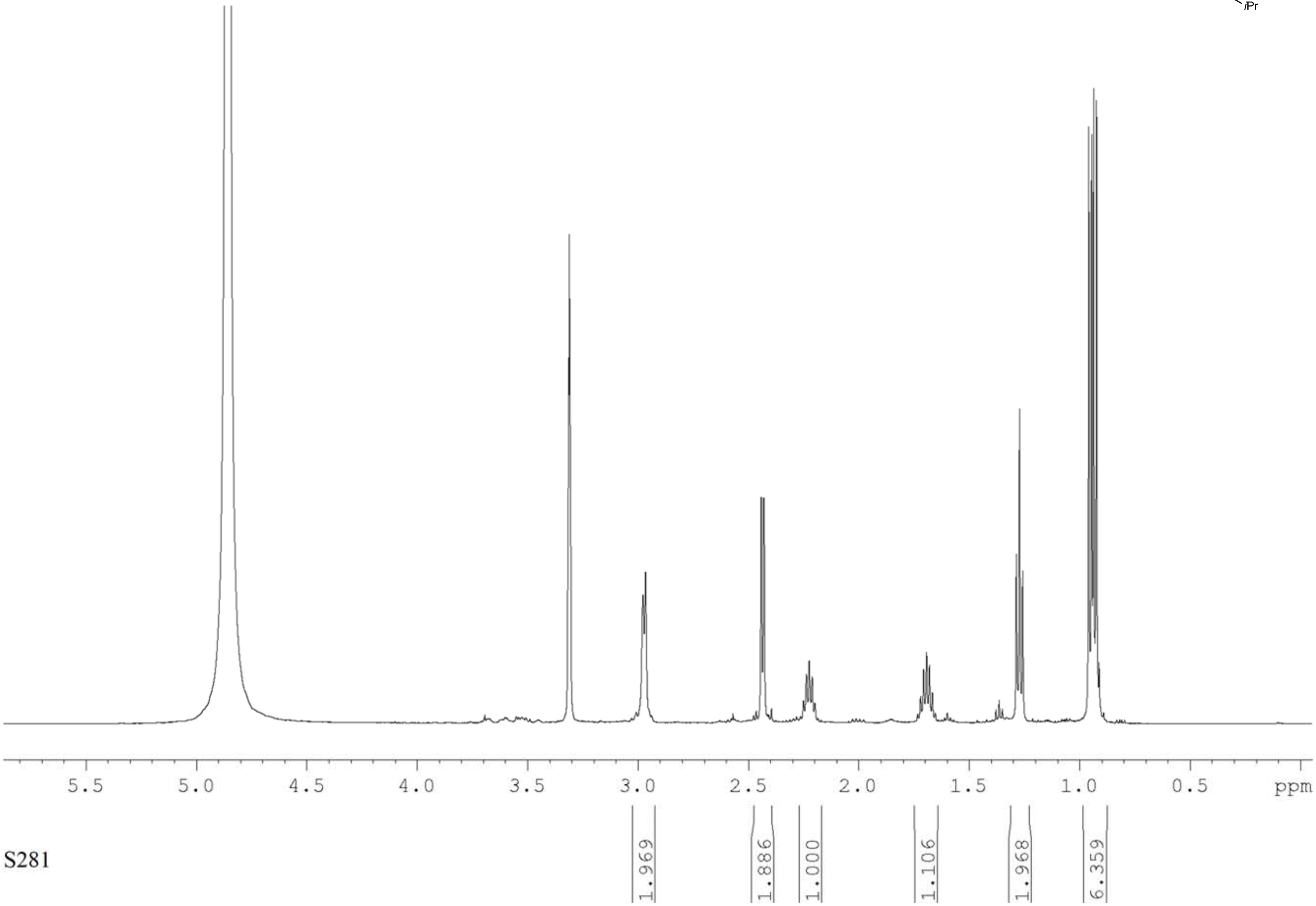

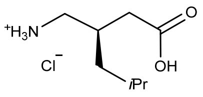

^{13}C NMR (126 MHz, $CDCl_3$) for **(*R*)-3-(Aminomethyl)-5-methylhexanoic acid hydrochloride ((*R*)-Pregabalin HCl salt) (6pa)**

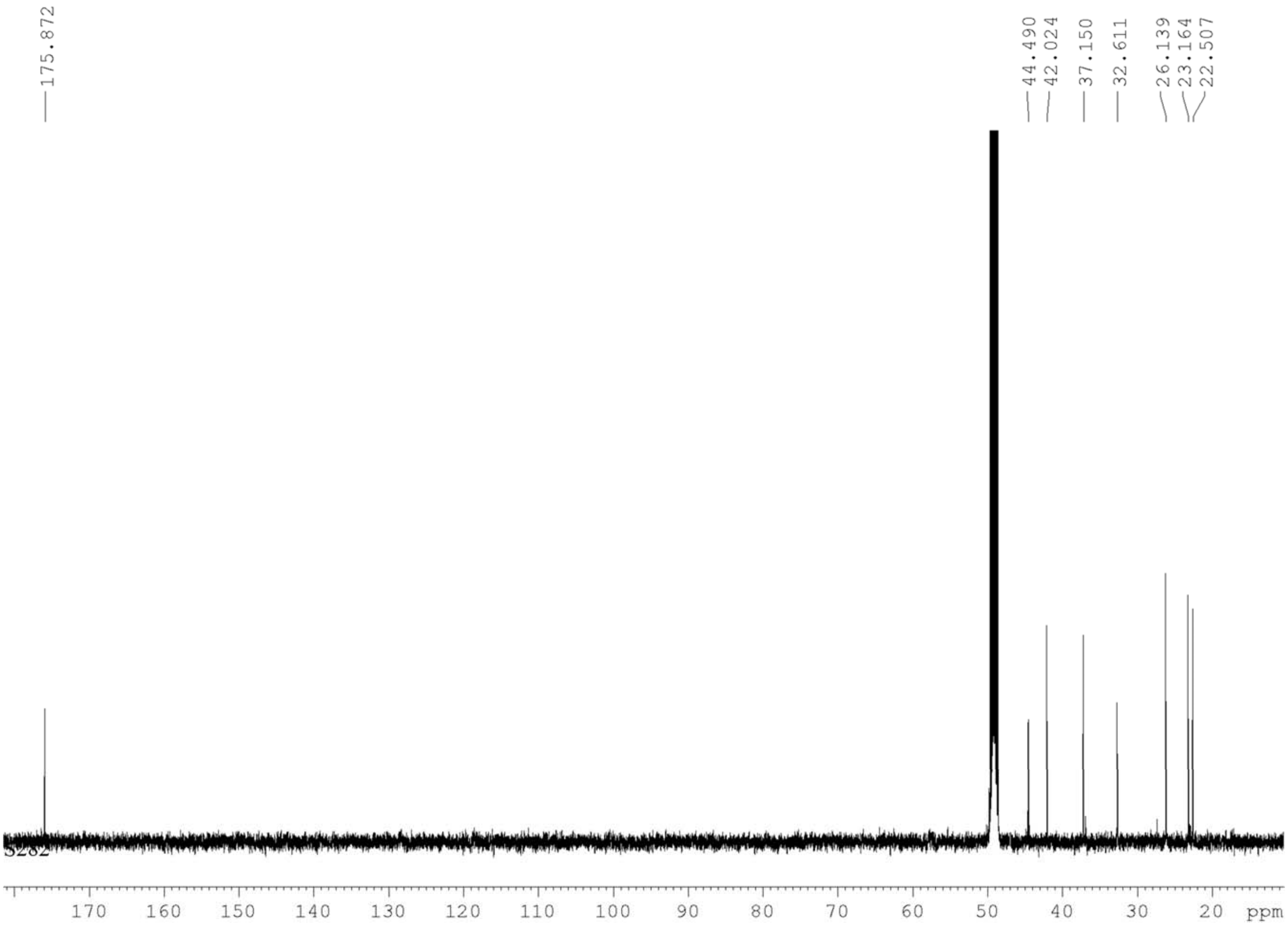

[1]**H NMR** (700 MHz, CDCl$_3$) for **(*R*)-4-Isobutylpyrrolidin-2-one (6pb)**

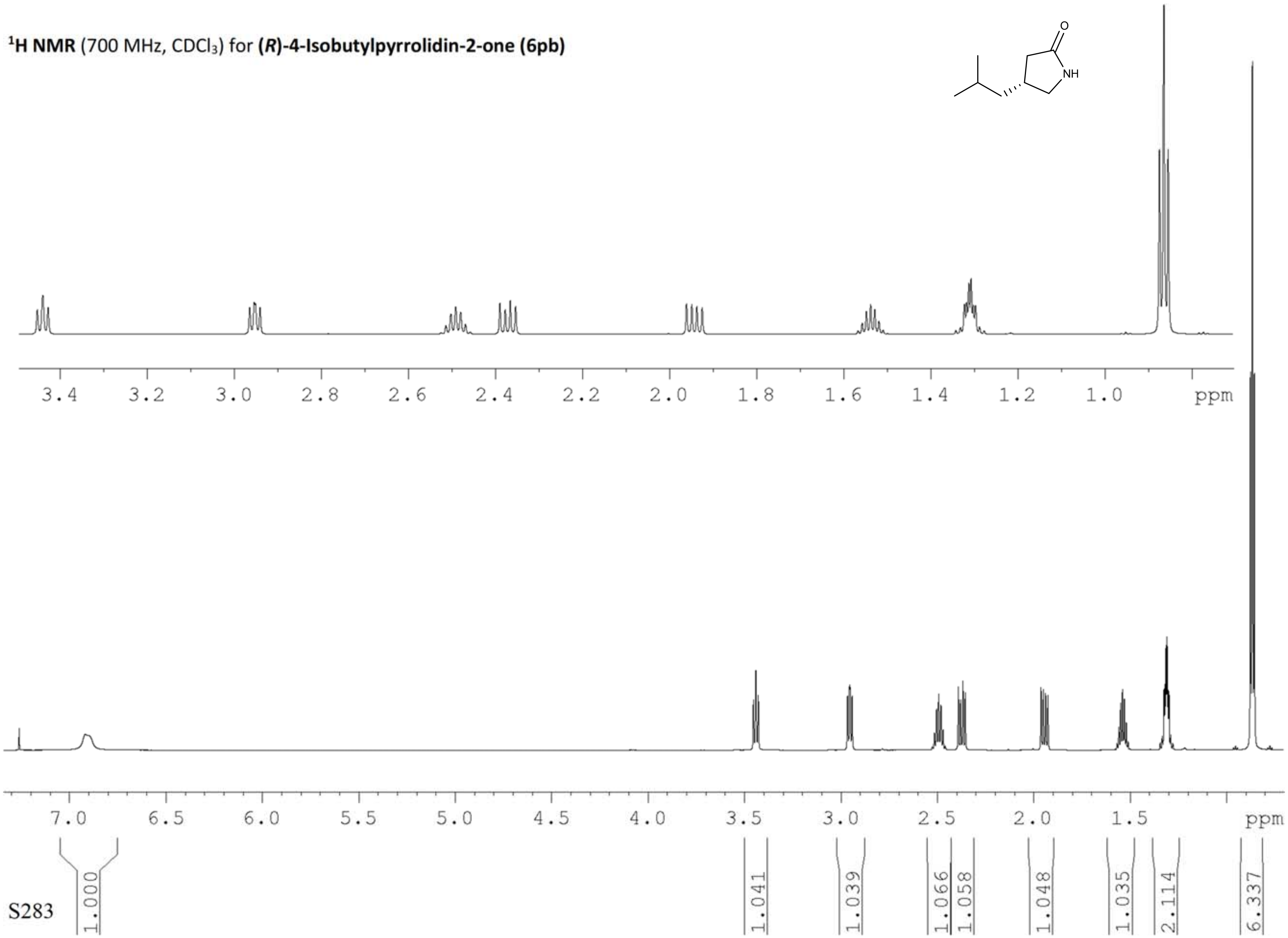

^{13}C NMR (176 MHz, $CDCl_3$) for **(*R*)-4-Isobutylpyrrolidin-2-one (6pb)**

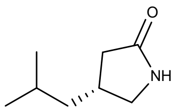

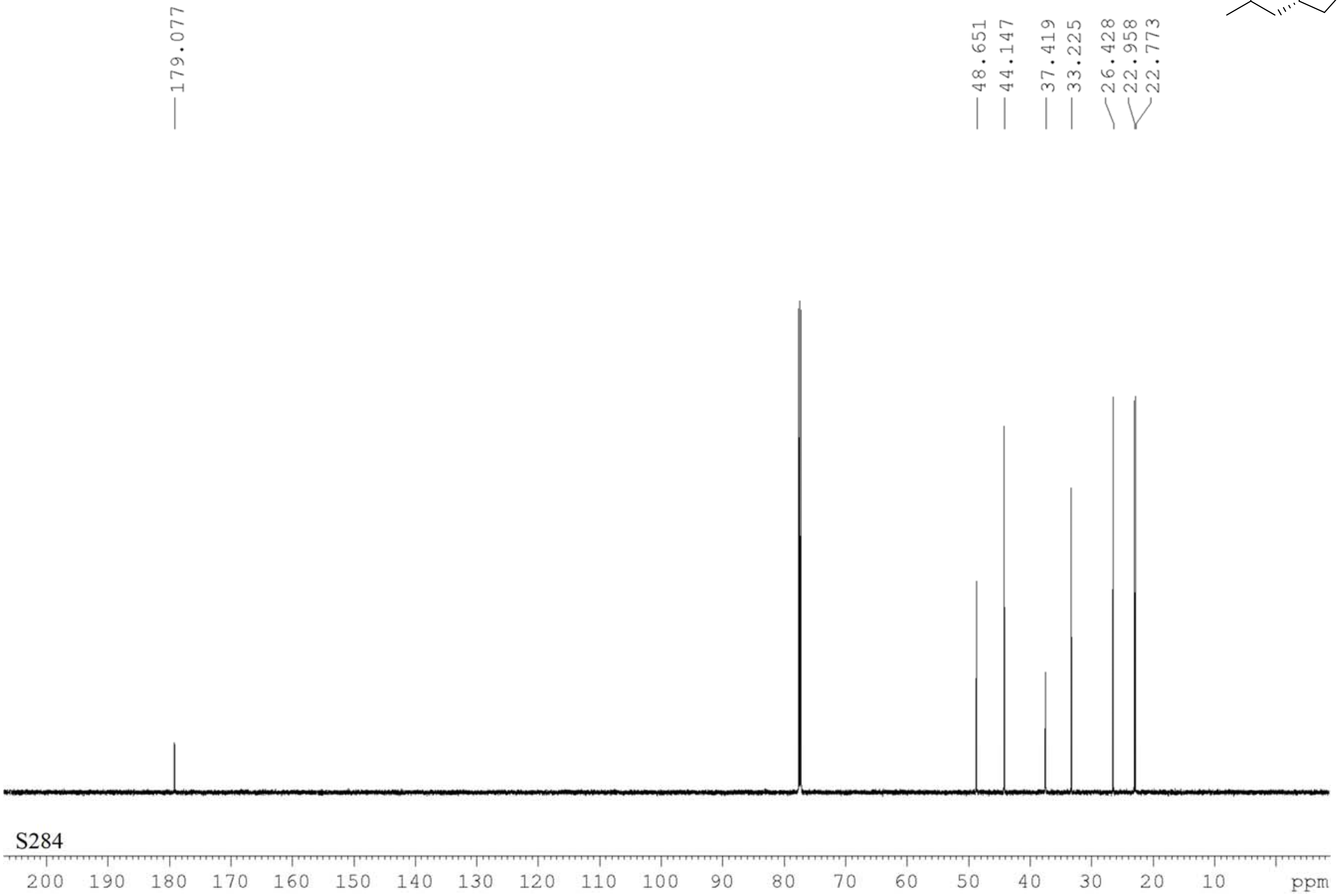

^{1}H NMR (400 MHz, CD_3CN) for ***N*-(3-(Phenyl(pyridin-2-yl)amino)butyl)benzamide (9)**

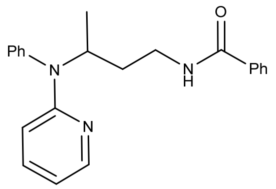

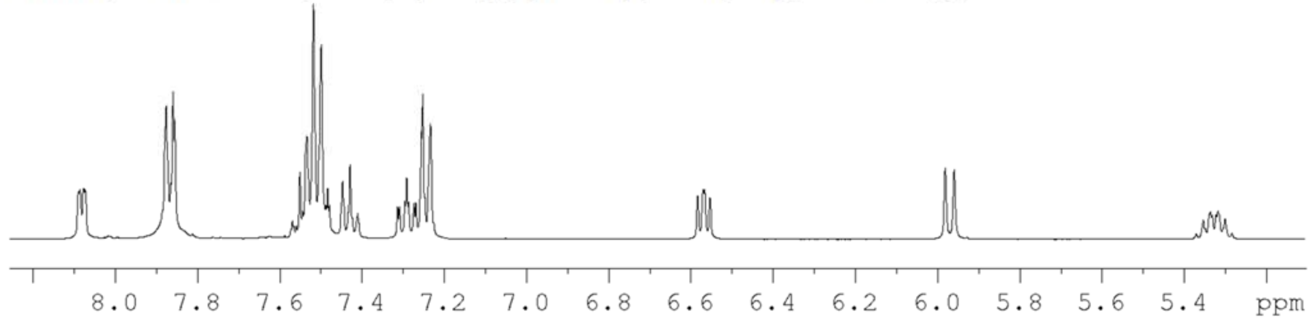

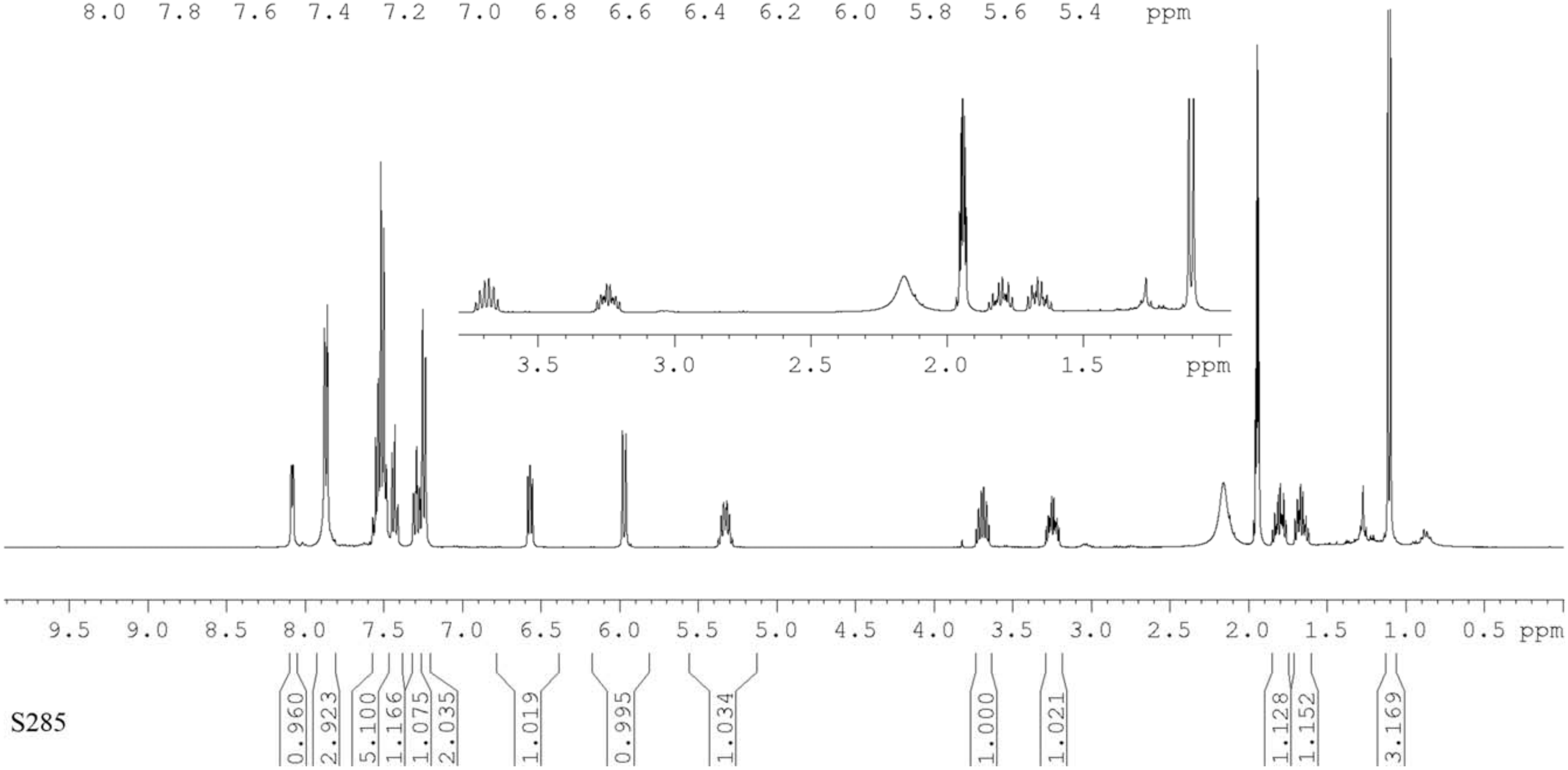

^{13}C NMR (101 MHz, CD_3CN) for ***N*-(3-(Phenyl(pyridin-2-yl)amino)butyl)benzamide (9)**

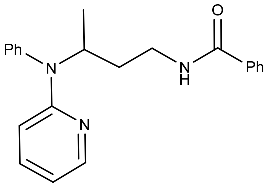

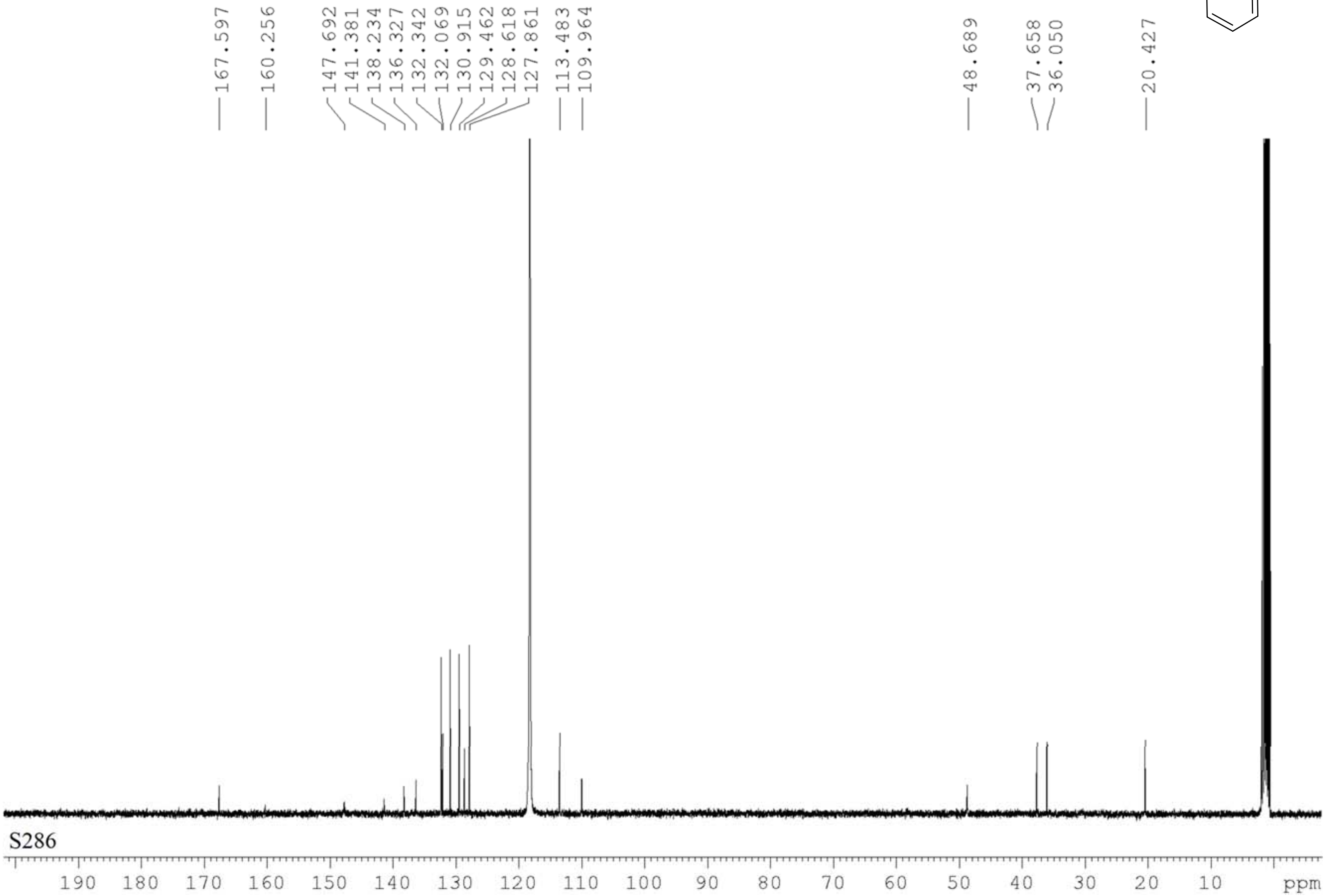

^{1}H NMR (400 MHz, $CDCl_3$) for ***(S)*-4-(Methyl(pyridin-2-yl)amino)-*N*,3-diphenylbutanamide (10)**

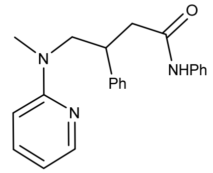

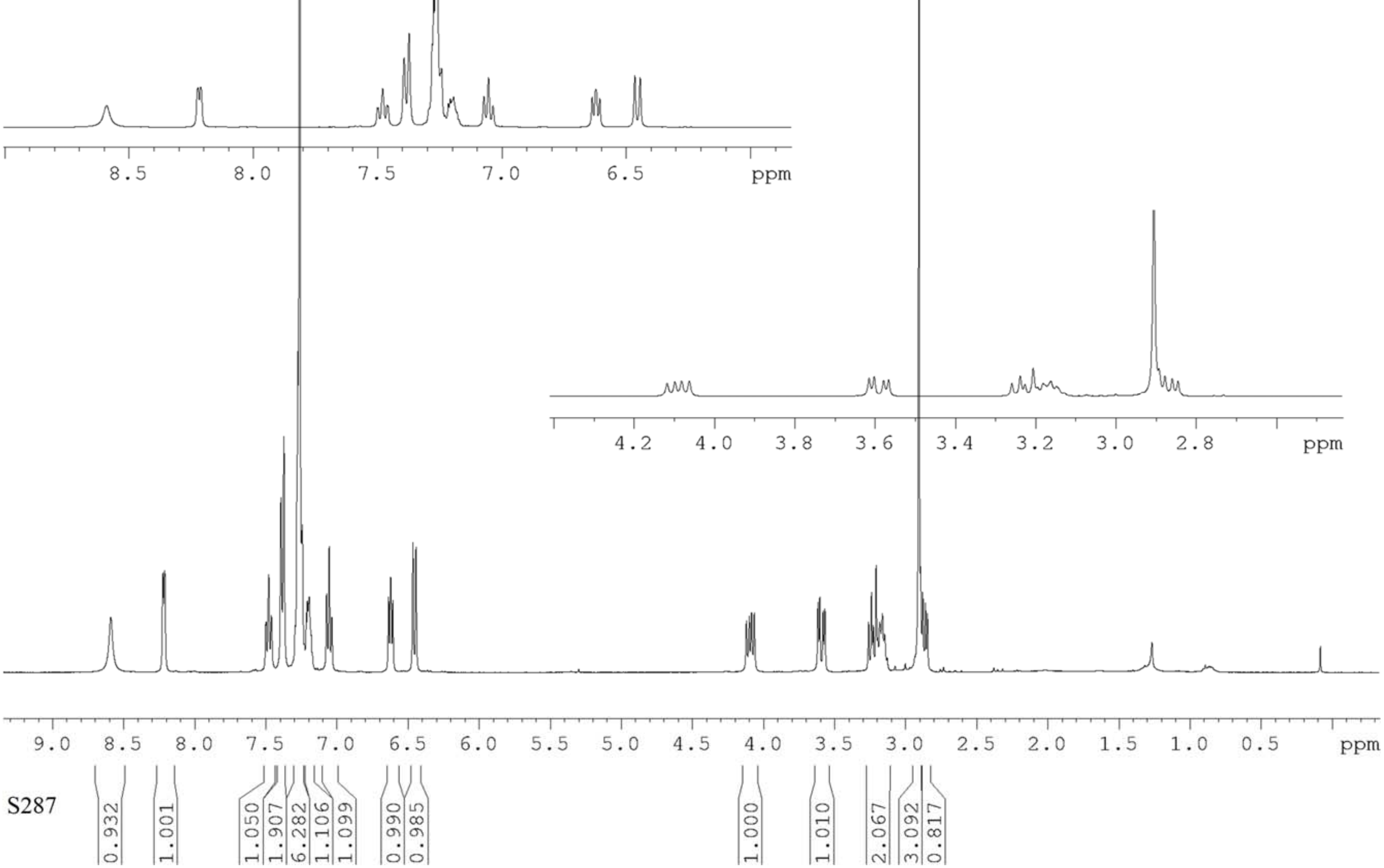

^{13}C NMR (101 MHz, $CDCl_3$) for **(*S*)-4-(Methyl(pyridin-2-yl)amino)-*N*,3-diphenylbutanamide (10)**

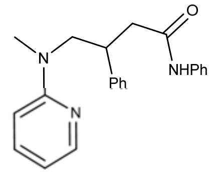

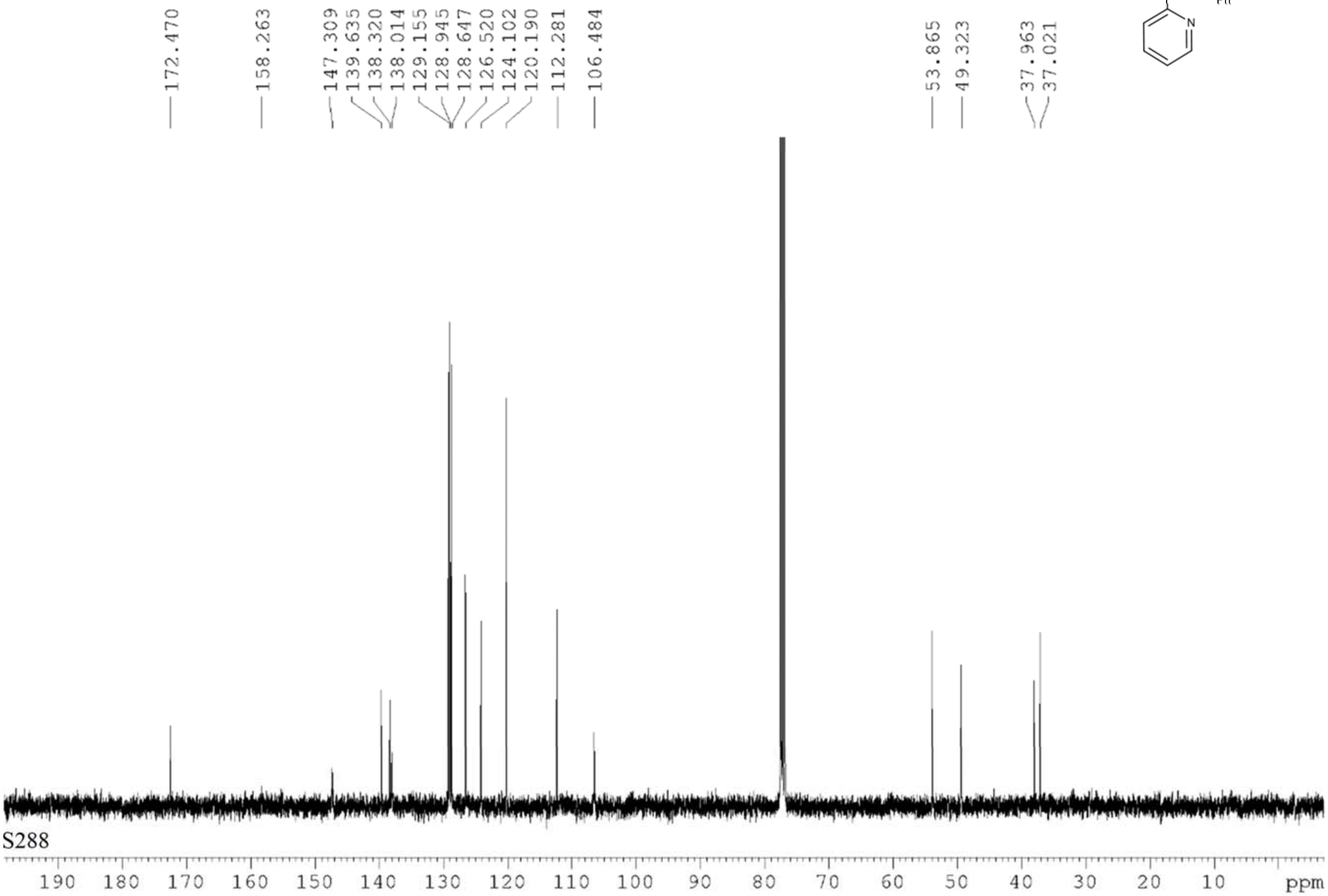

^{1}H NMR (400 MHz, $CDCl_3$) for **2-Benzyl-*N*-phenylacrylamide (4a)**

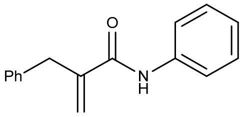

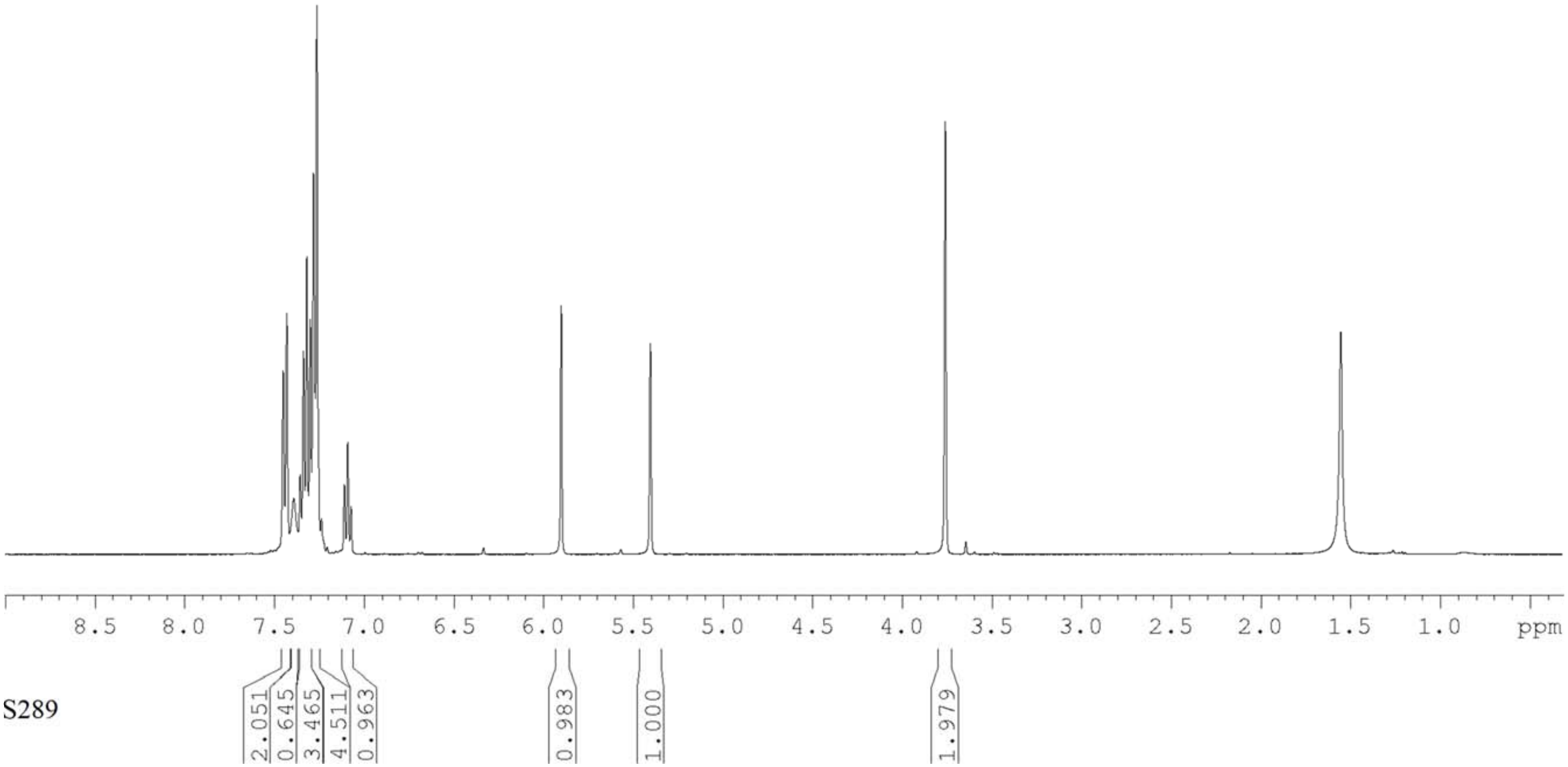

^{13}C NMR (101 MHz, $CDCl_3$) for **2-Benzyl-*N*-phenylacrylamide (4a)**

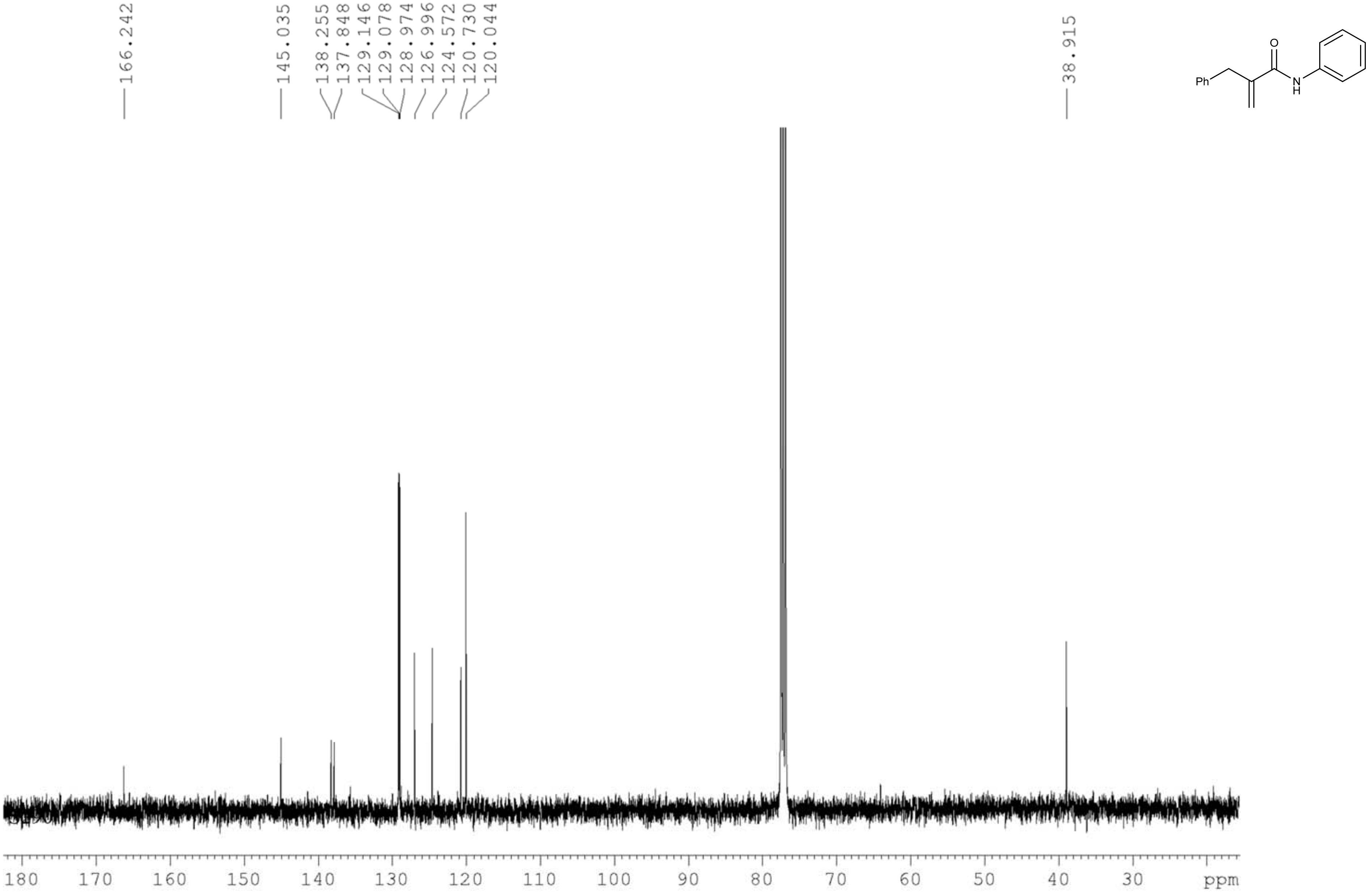

^{1}H NMR (400 MHz, $CDCl_3$) for **2-Benzyl-*N*-mesitylacrylamide (4f)**

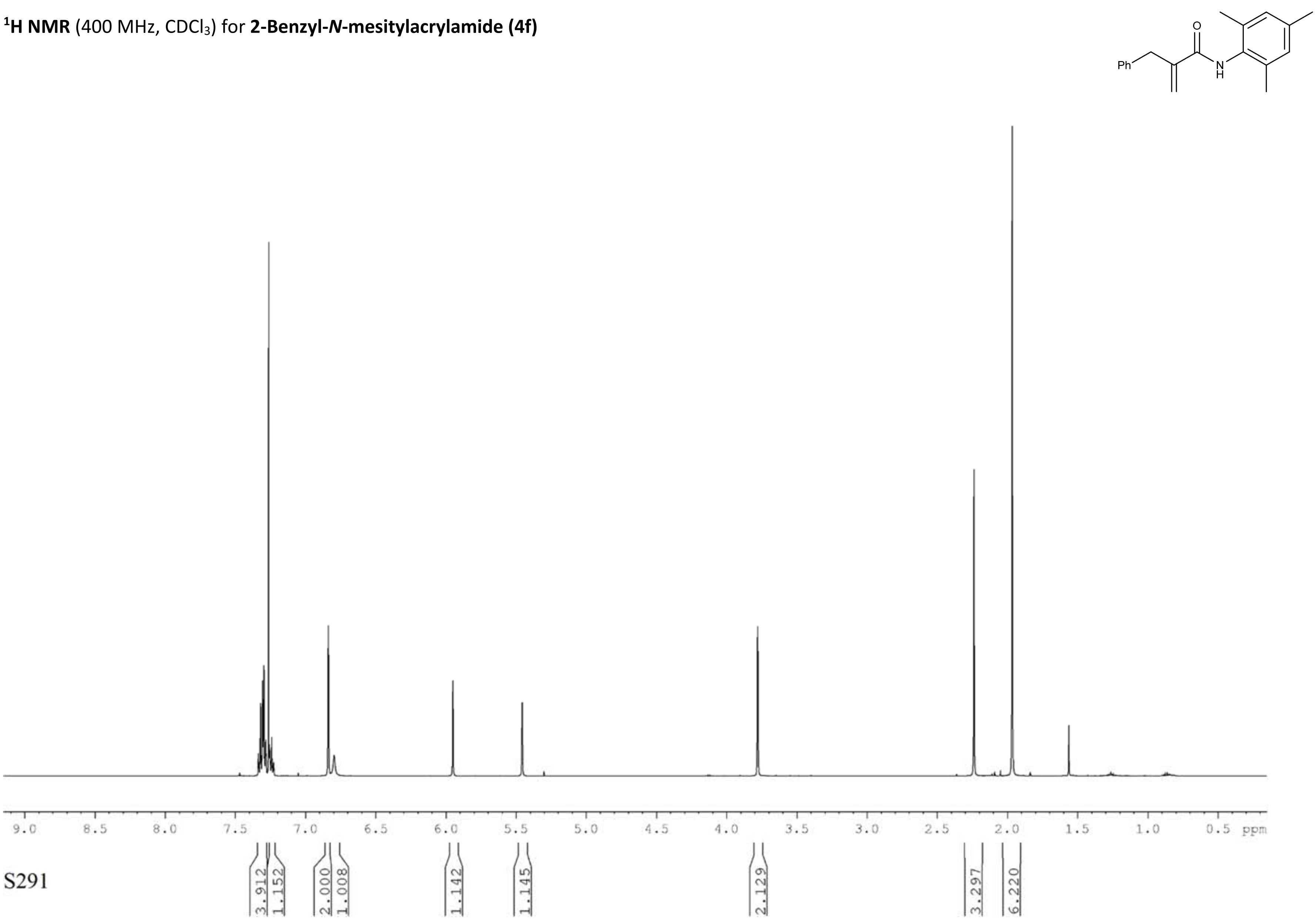

^{13}C NMR (126 MHz, $CDCl_3$) for **2-Benzyl-*N*-mesitylacrylamide (4f)**

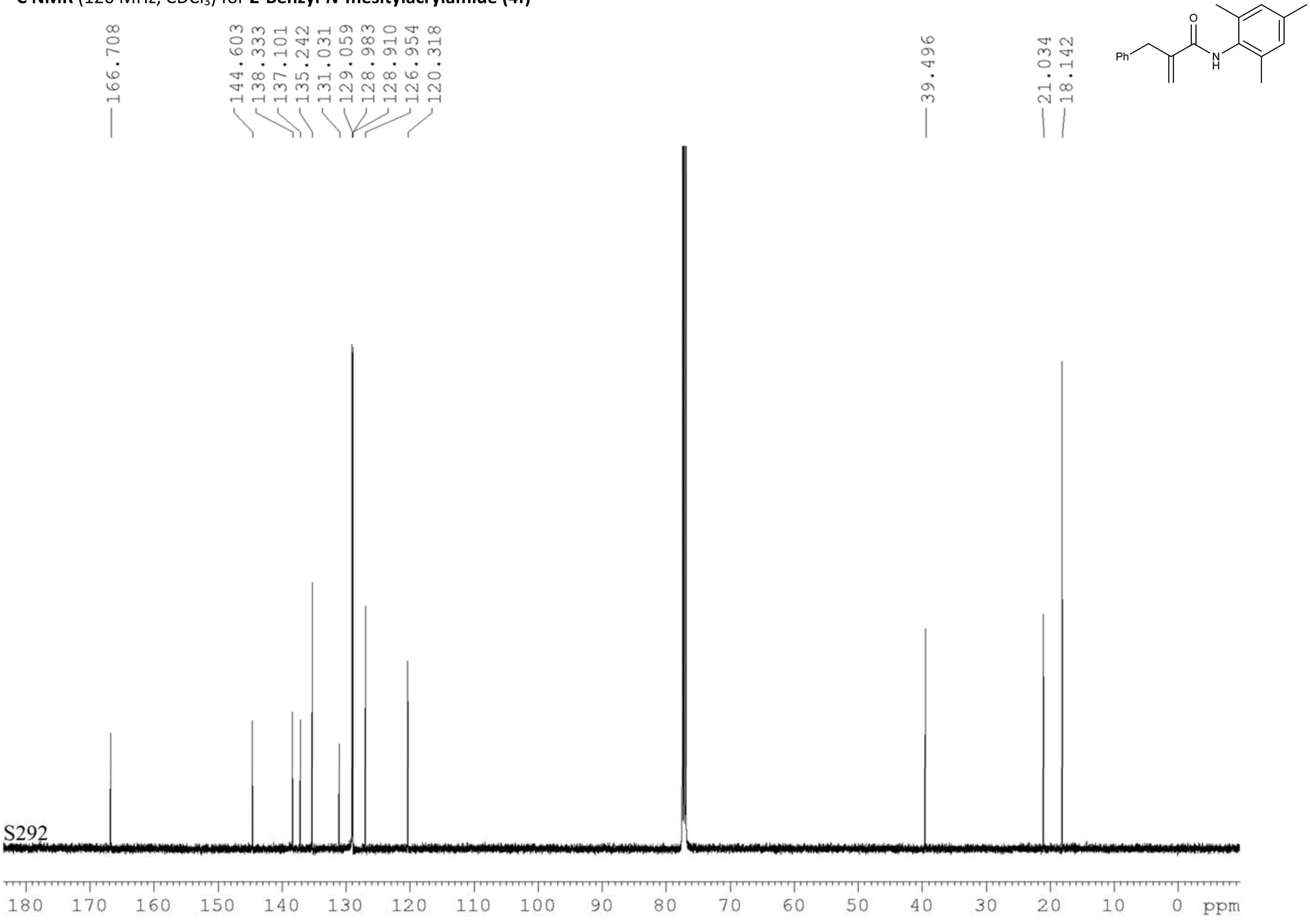

^{1}H NMR (700 MHz, $CDCl_3$) for **2-Benzyl-*N*-methyl-*N*-phenylacrylamide (4e)**

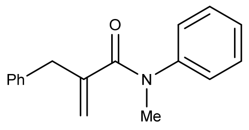

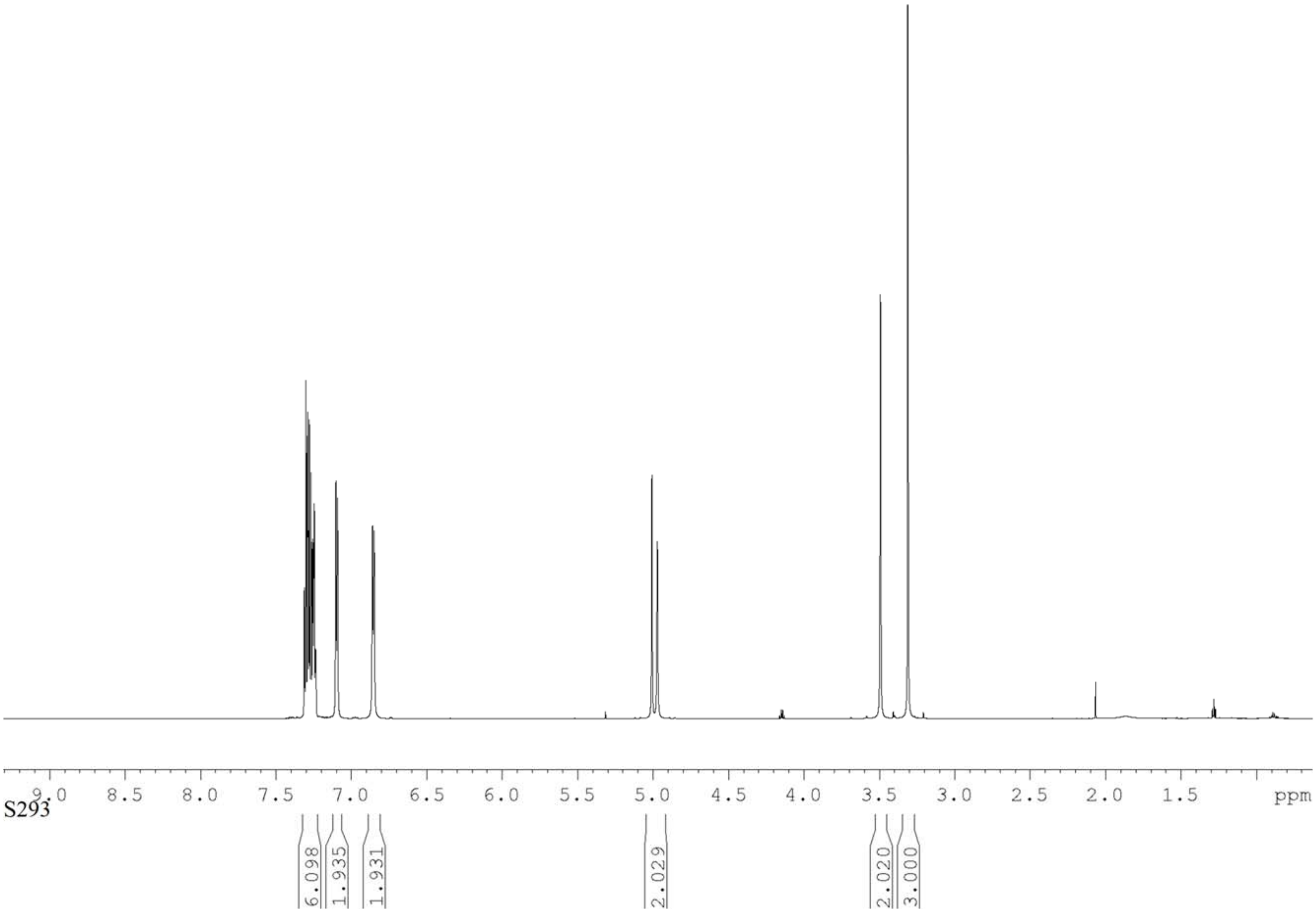

^{13}C NMR (176 MHz, $CDCl_3$) for **2-Benzyl-*N*-methyl-*N*-phenylacrylamide (4e)**

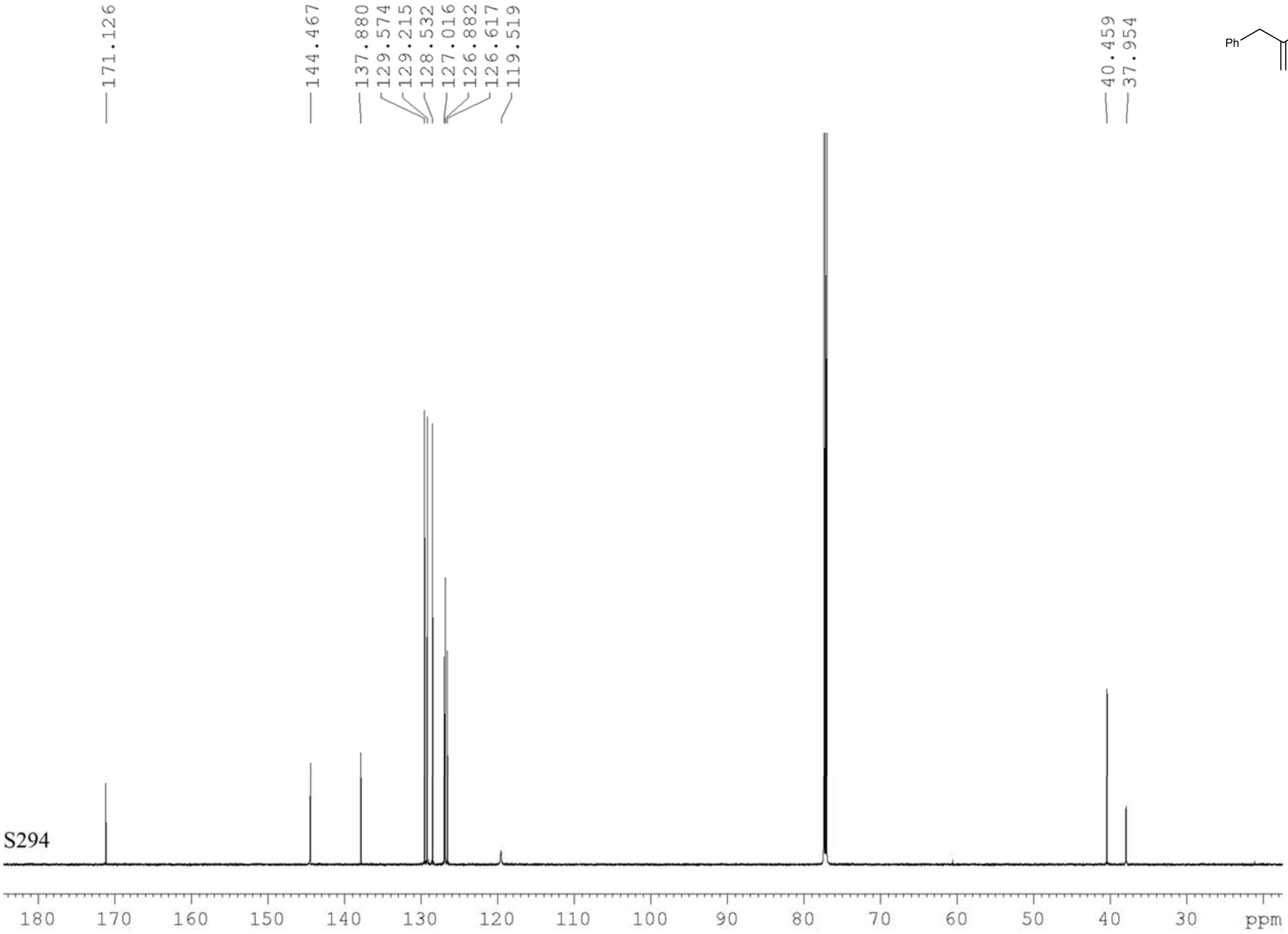

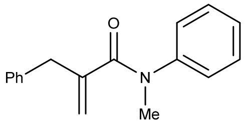

¹H NMR (400 MHz, $CDCl_3$) for **3-Methyl-2-methylene-*N*-phenylbutanamide (4g)**

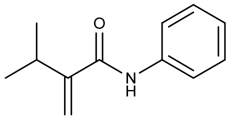

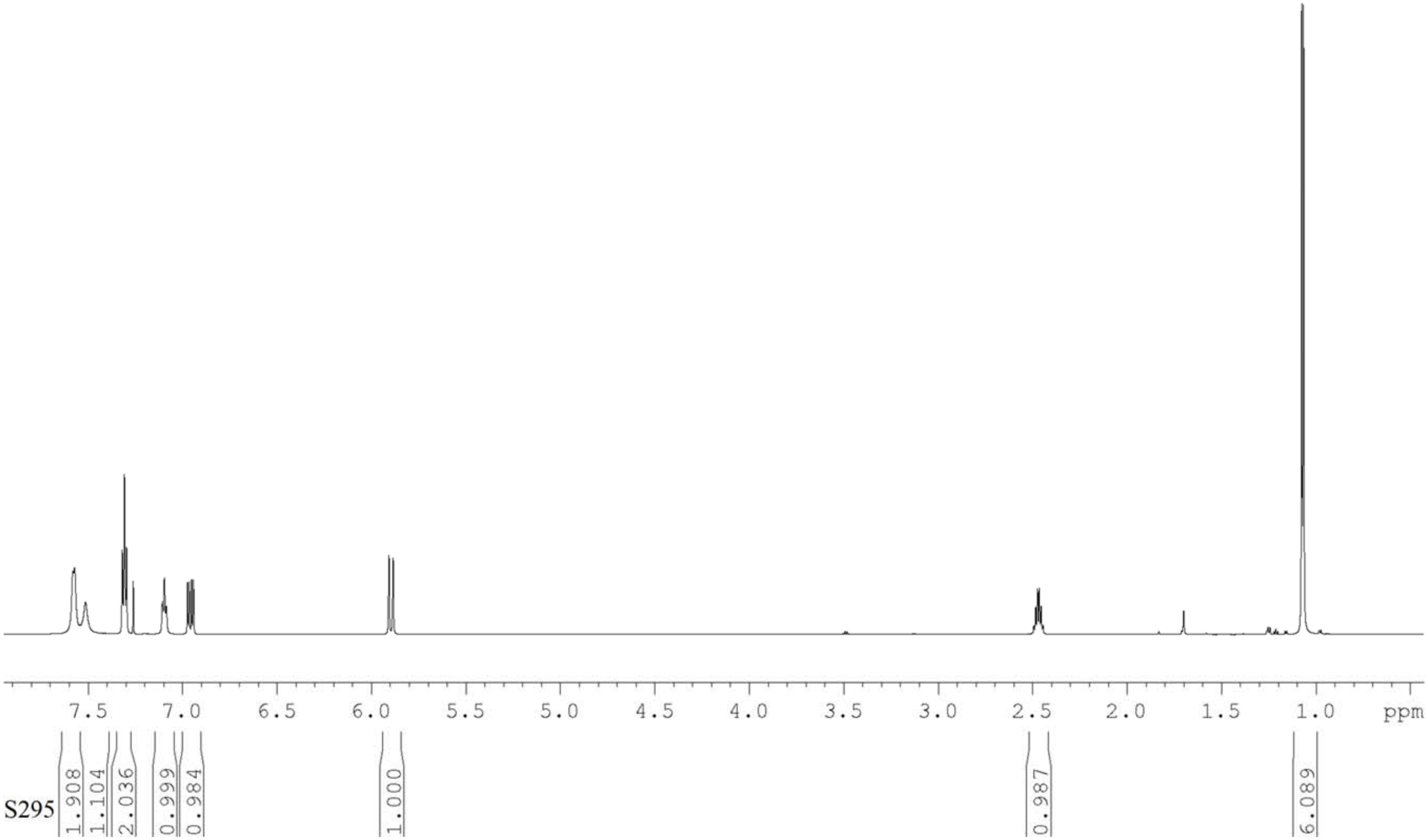

^{13}C NMR (101 MHz, $CDCl_3$) for **3-Methyl-2-methylene-*N*-phenylbutanamide (4g)**

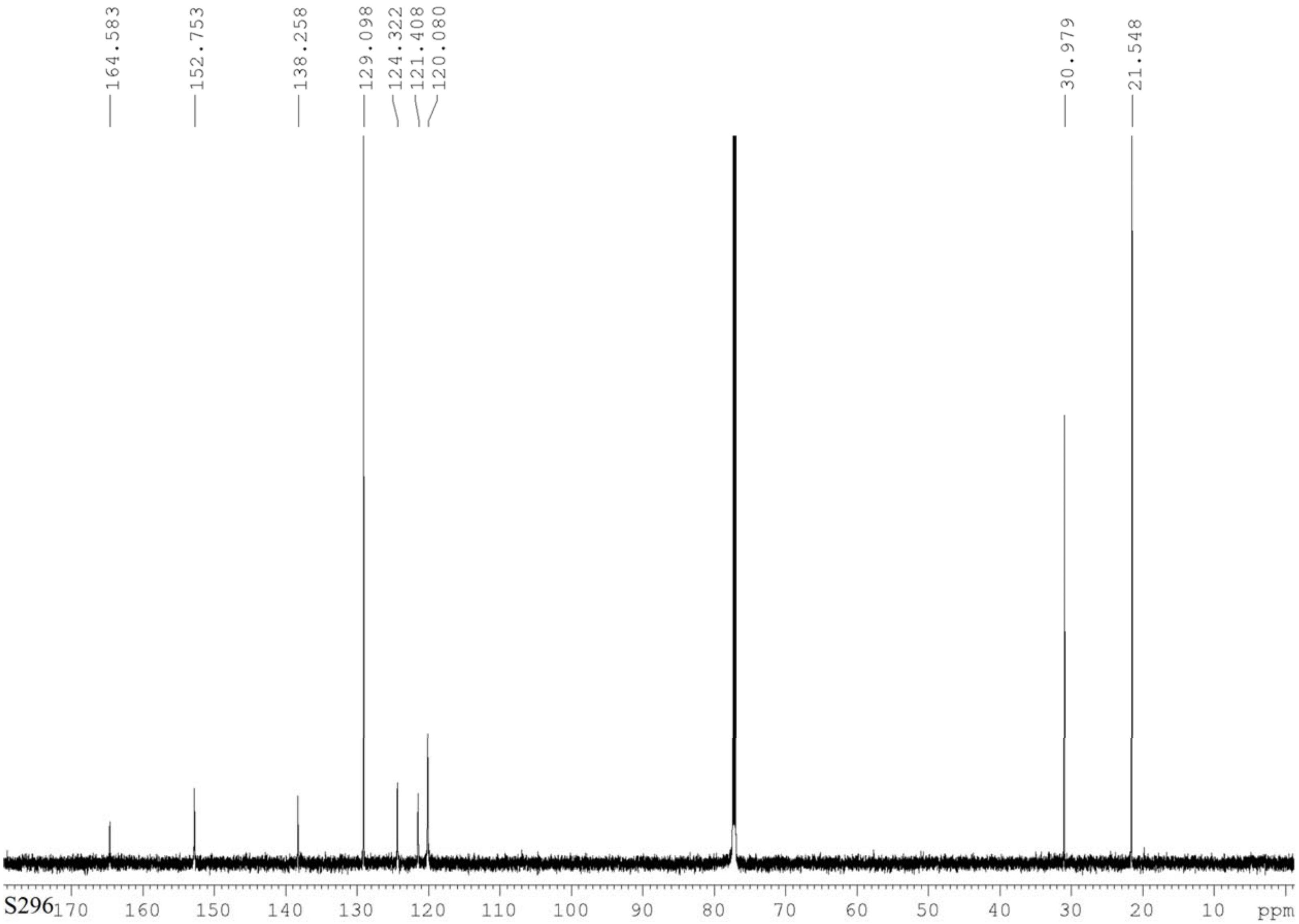

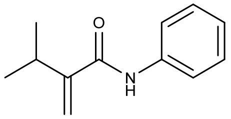

^{1}H NMR (400 MHz, $CDCl_3$) for **2-Methylene-*N*-phenylbutanamide (4i)**

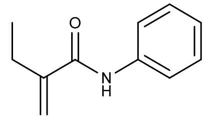

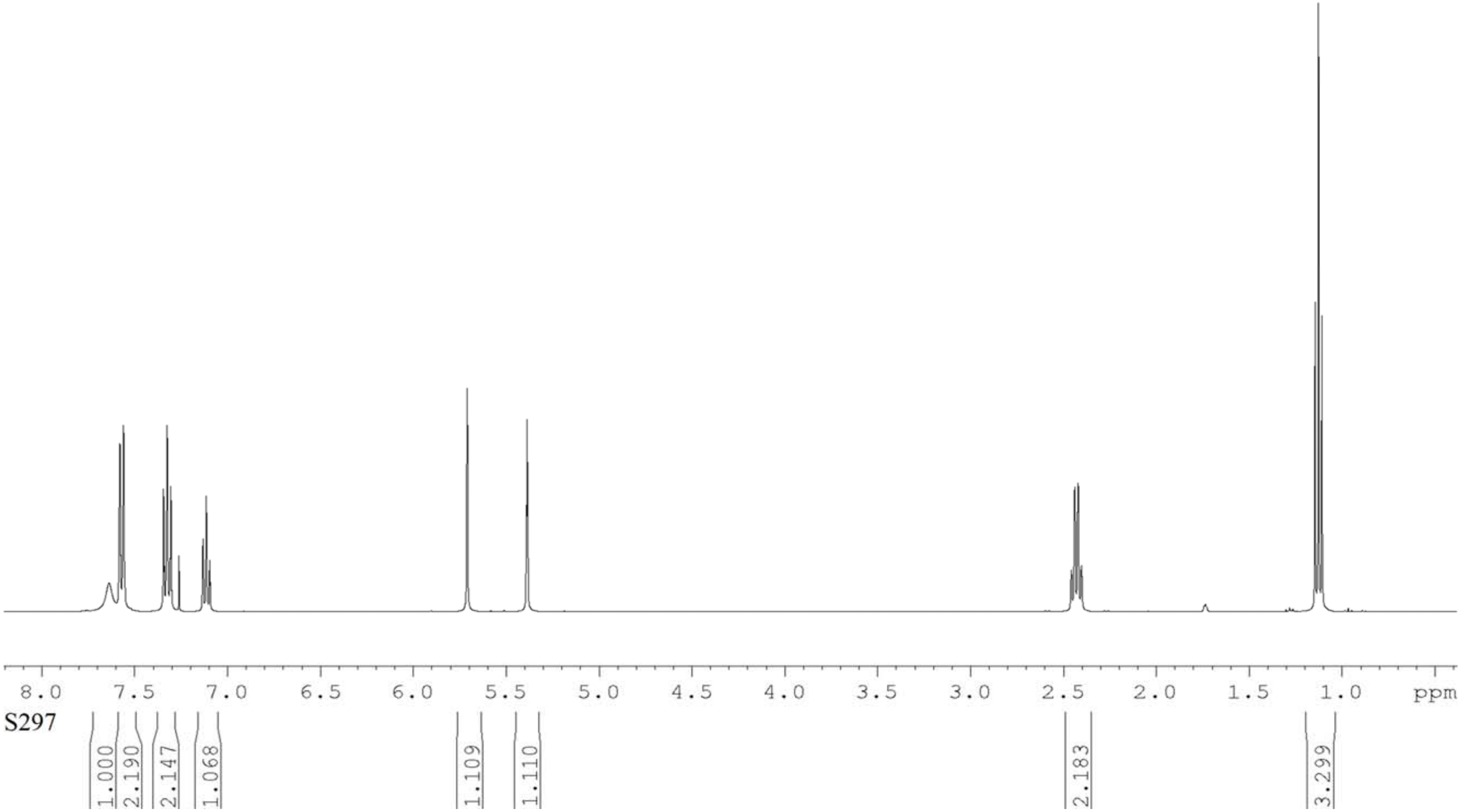

^{13}C NMR (101 MHz, $CDCl_3$) for **2-Methylene-*N*-phenylbutanamide (4i)**

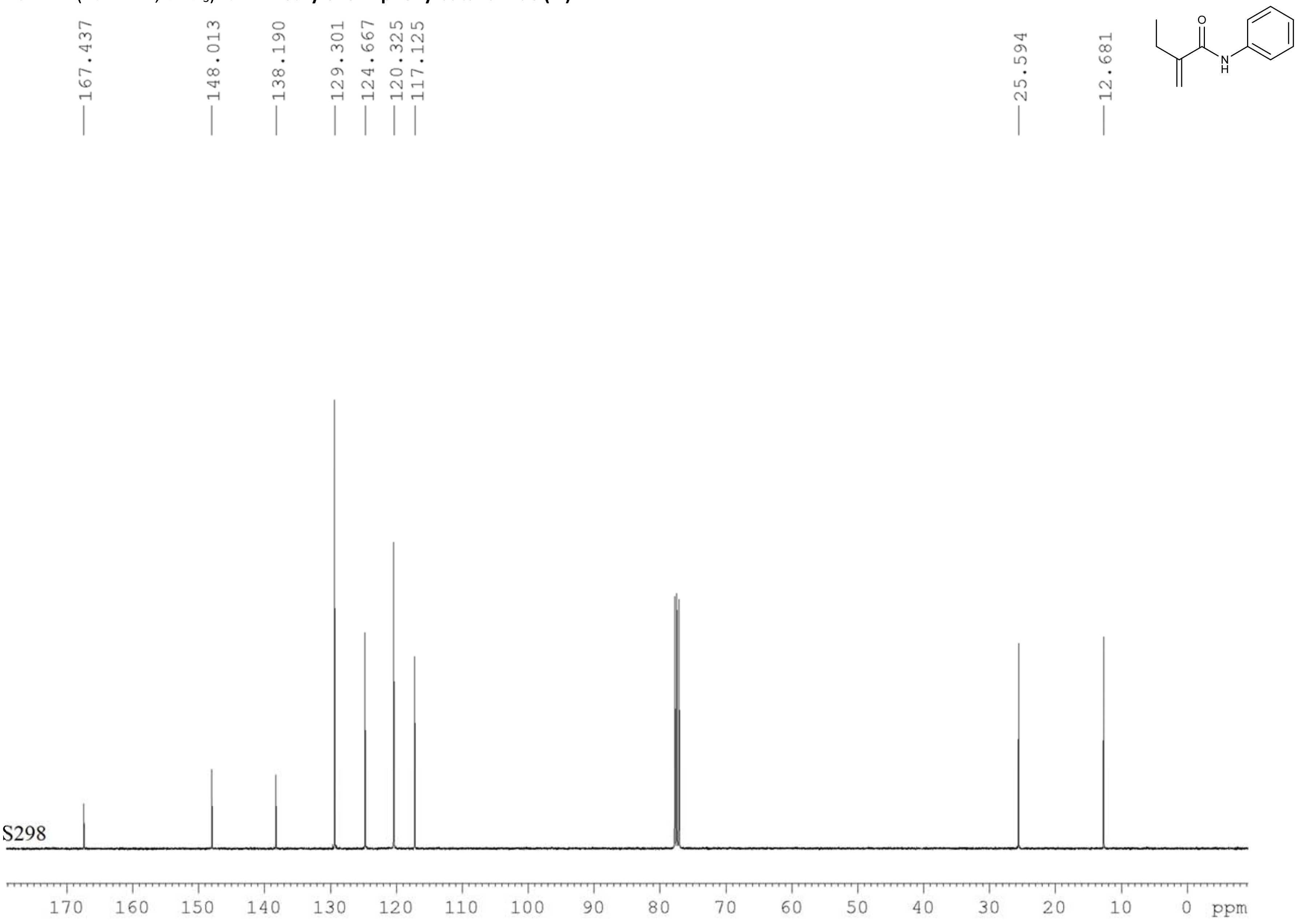

^{1}**H NMR** (400 MHz, $CDCl_3$) for **Methyl 3-(phenylcarbamoyl)but-3-enoate (4h)**

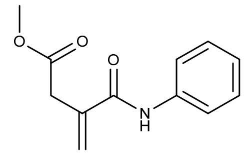

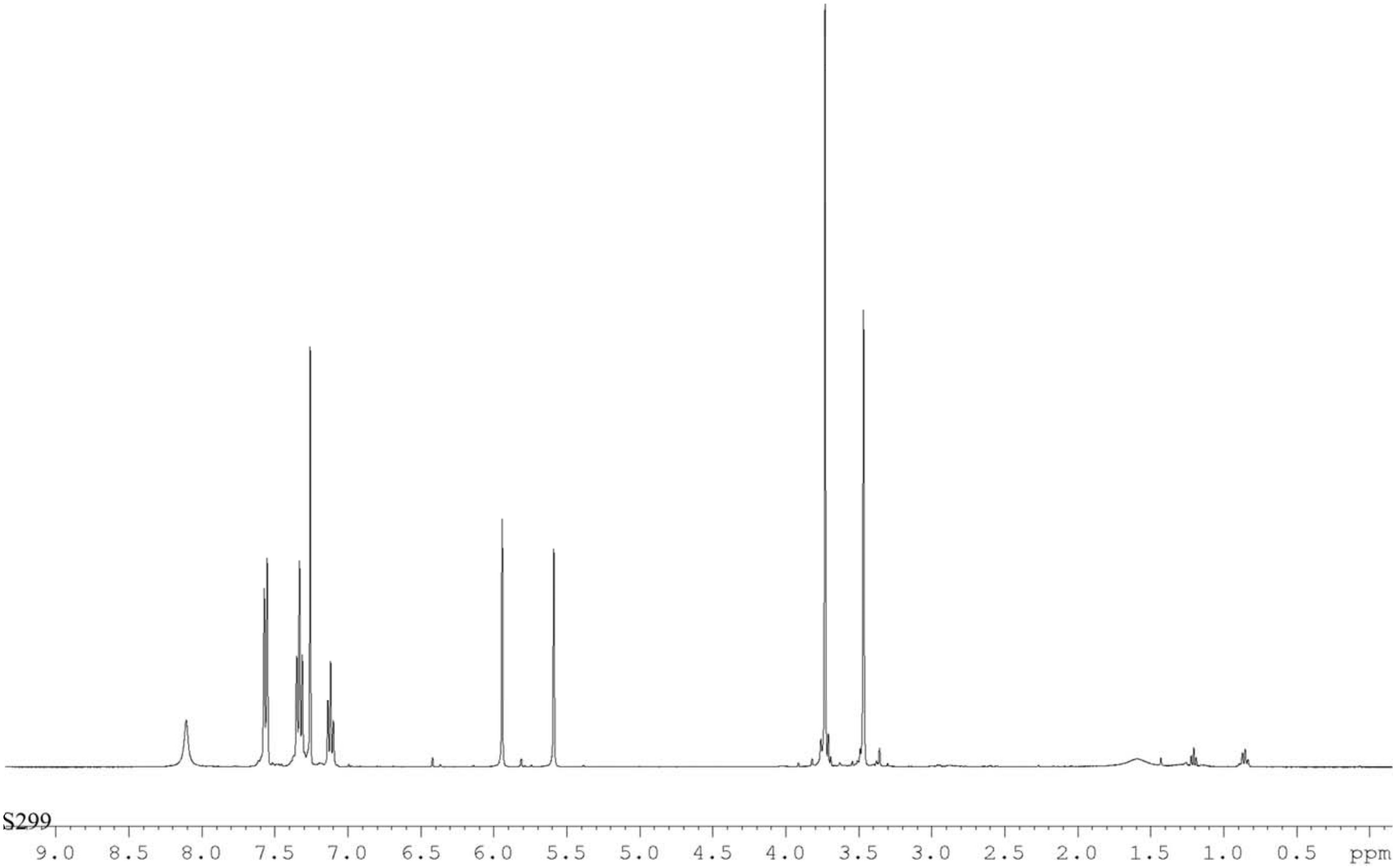

^{13}C NMR (176 MHz, $CDCl_3$) for **Methyl 3-(phenylcarbamoyl)but-3-enoate (4h)**

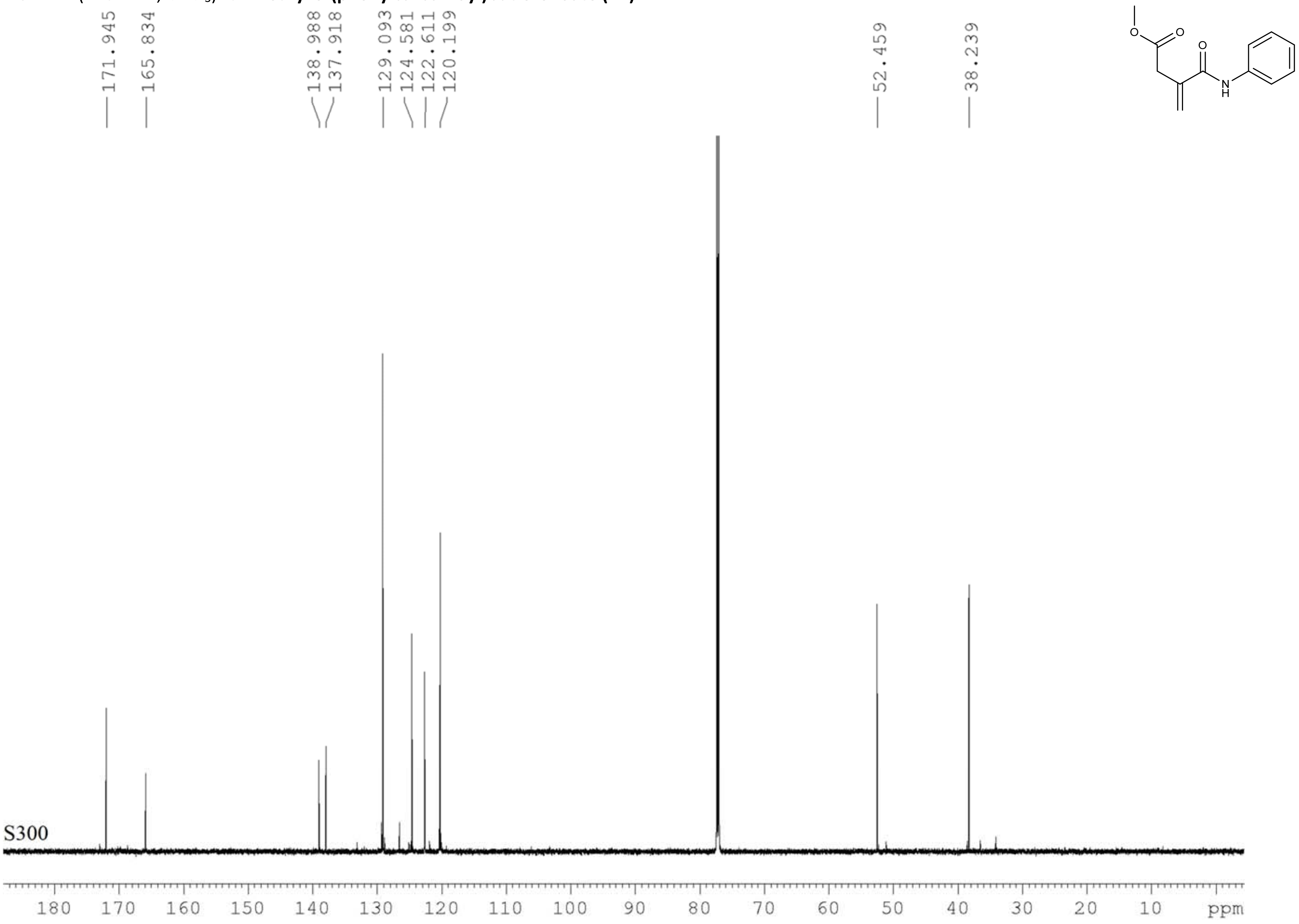

[1]**H NMR** (400 MHz, $CDCl_3$) for **(*E*)-5-Methyl-*N*-phenylhex-2-enamide (4c)**

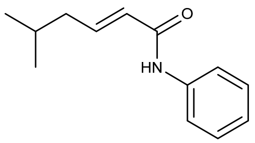

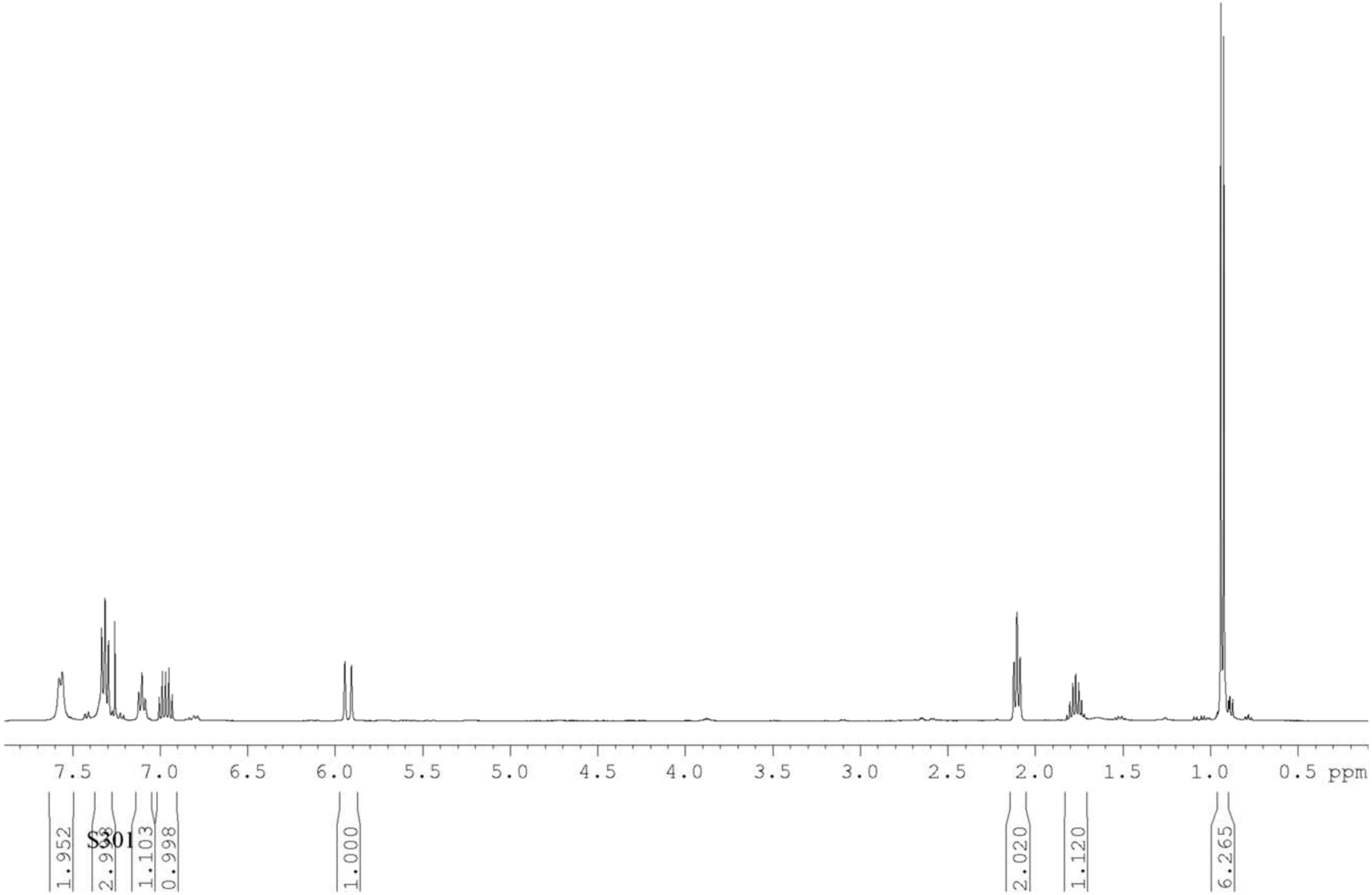

^{13}C NMR (101 MHz, $CDCl_3$) for **(*E*)-5-Methyl-*N*-phenylhex-2-enamide (4c)**

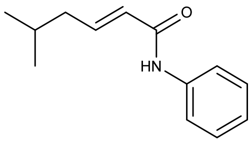

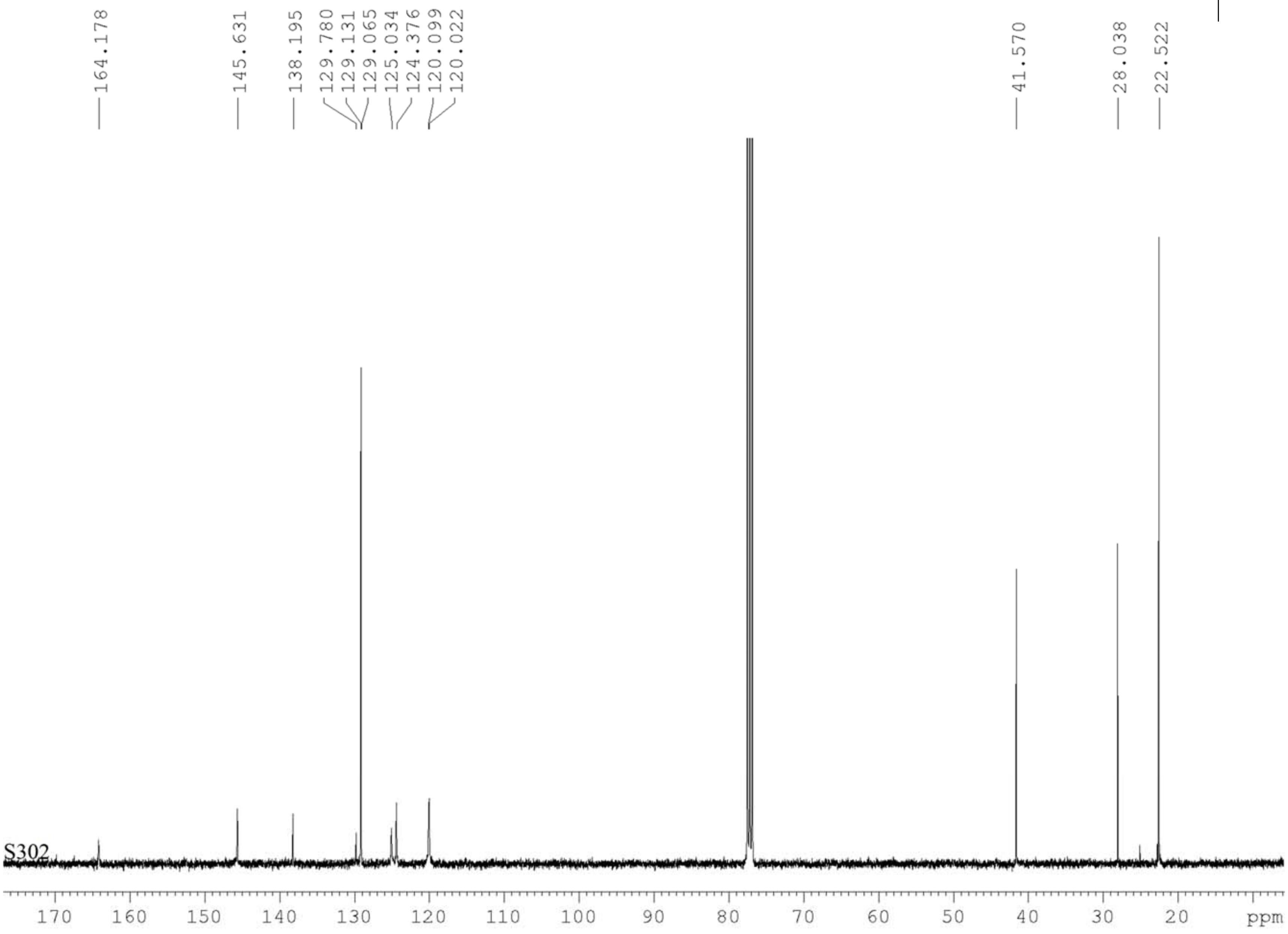

^{1}H NMR (400 MHz, DMSO) for ***(E)*-*N*-(4-(trifluoromethyl)phenyl)but-2-enamide (4k)**

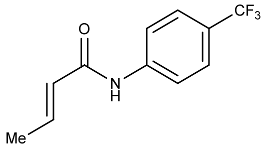

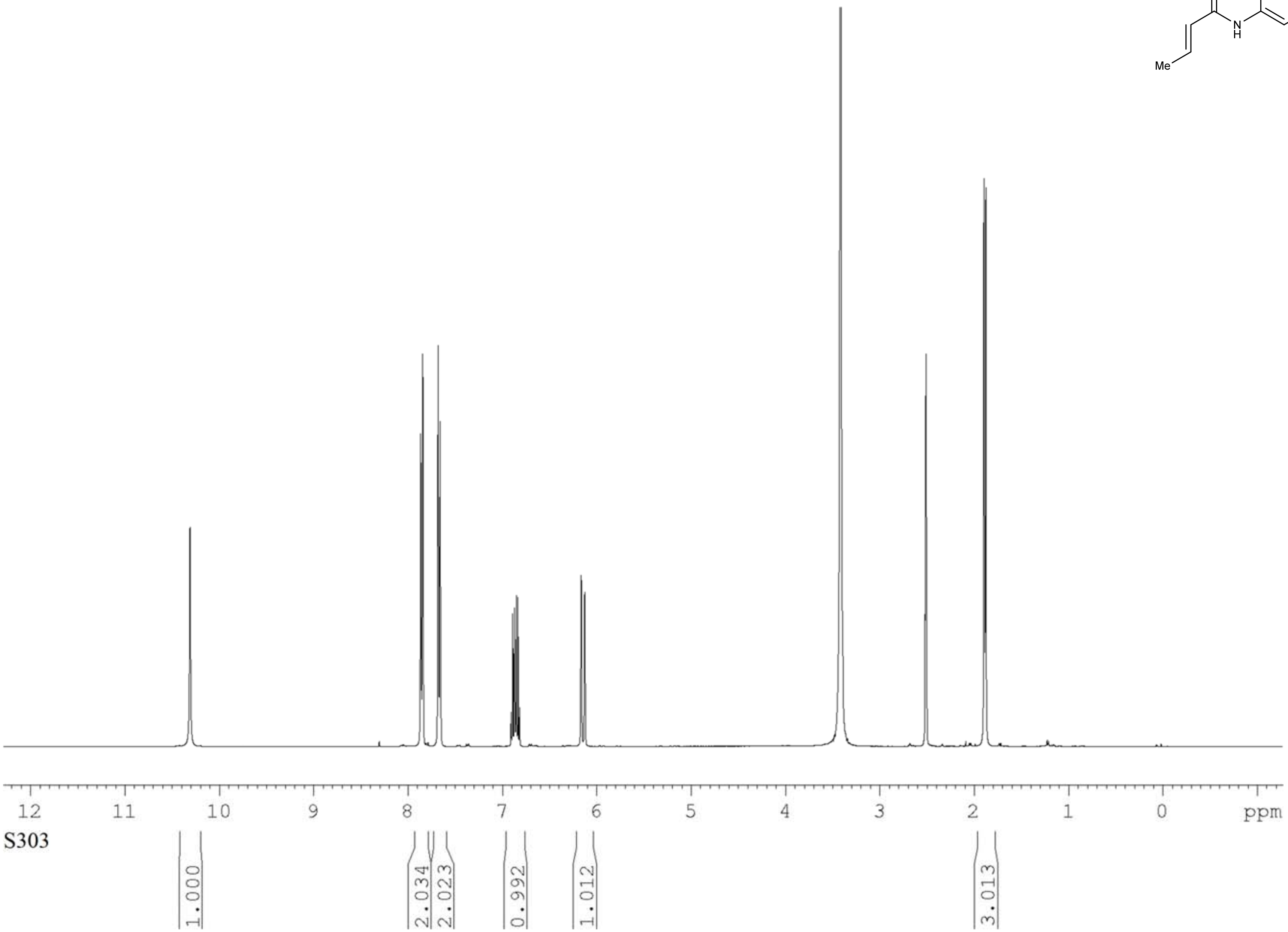

^{13}C NMR (101 MHz, DMSO) for **(*E*)-*N*-(4-(trifluoromethyl)phenyl)but-2-enamide (4k)**

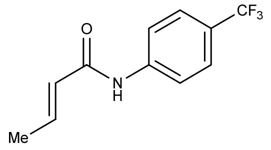

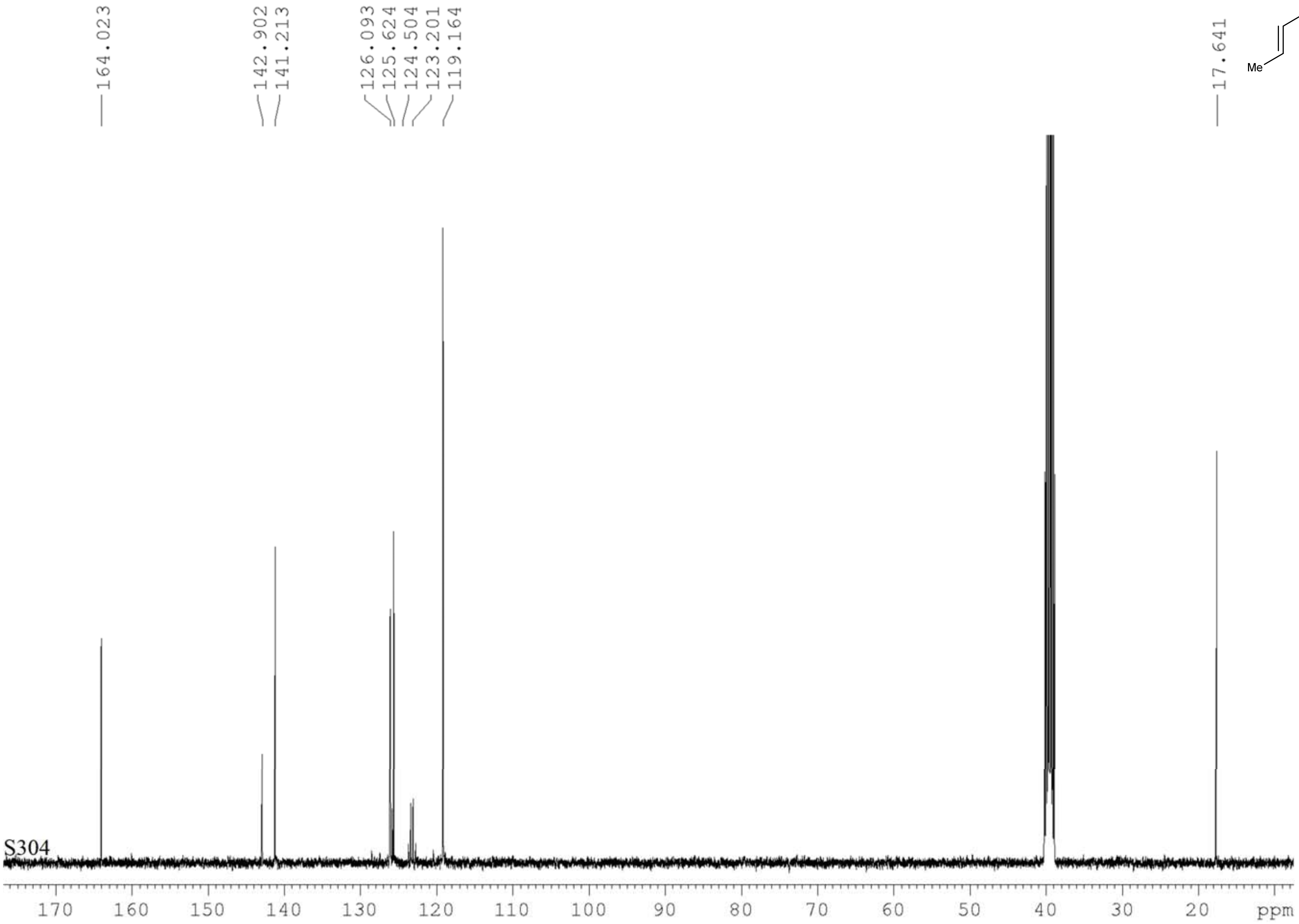

^{19}F NMR (471 MHz, $CHCl_3$) for **(*E*)-*N*-(4-(trifluoromethyl)phenyl)but-2-enamide (4k)**

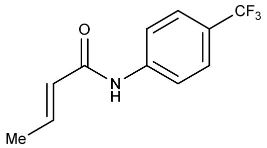

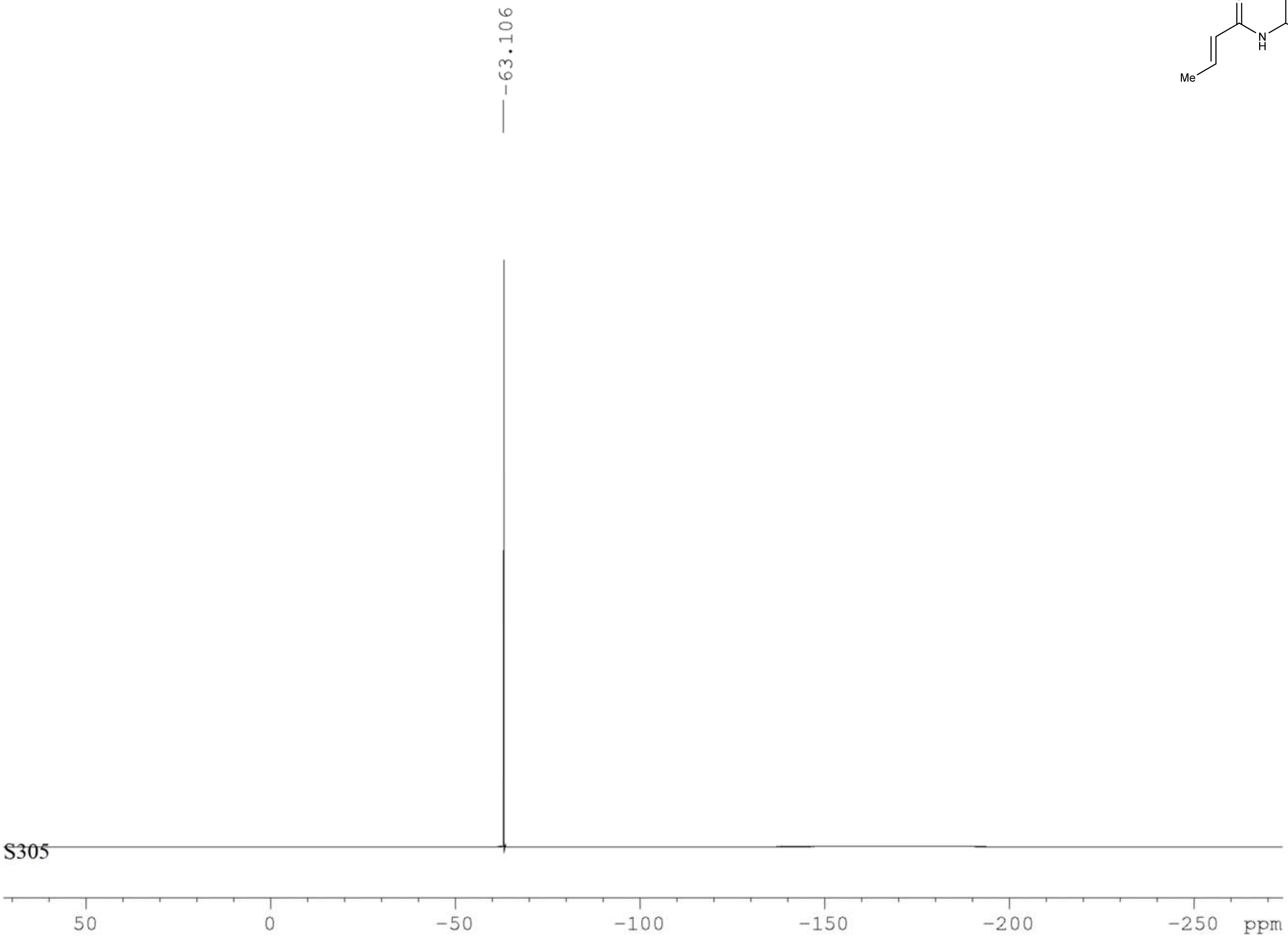

^{1}H NMR (500 MHz, $CDCl_3$) for **(*E*)-*N*-Phenylbut-2-enamide (4d)**

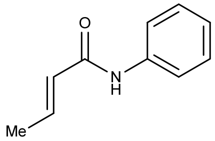

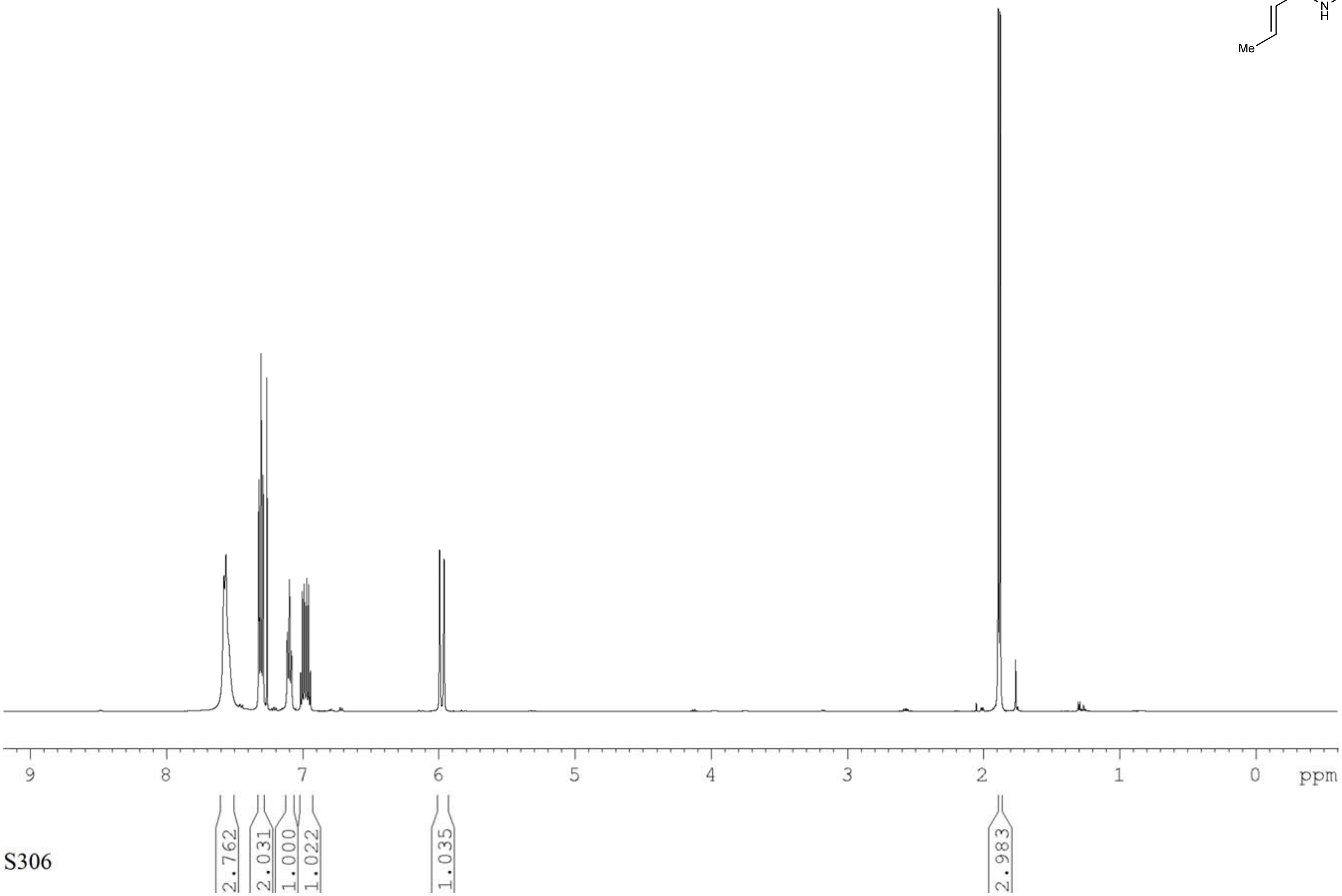

^{13}C NMR (126 MHz, $CDCl_3$) for **(*E*)-*N*-Phenylbut-2-enamide (4d)**

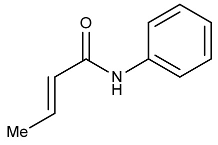

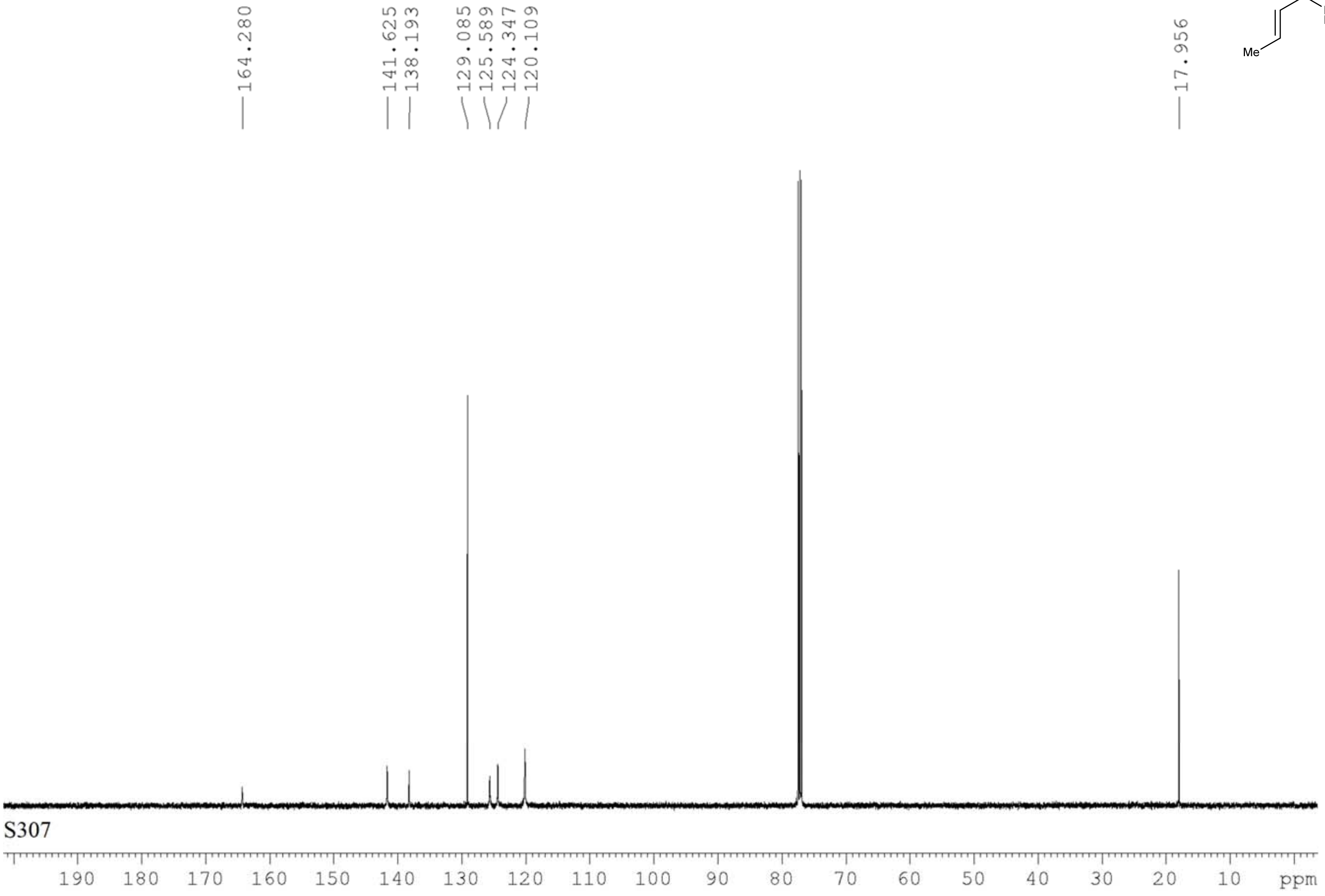

^{1}H NMR (500 MHz, $CDCl_3$) for **(*E*)-4-Methyl-*N*-phenylpent-2-enamide (4j)**

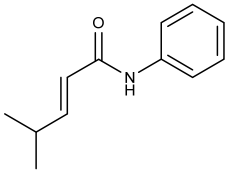

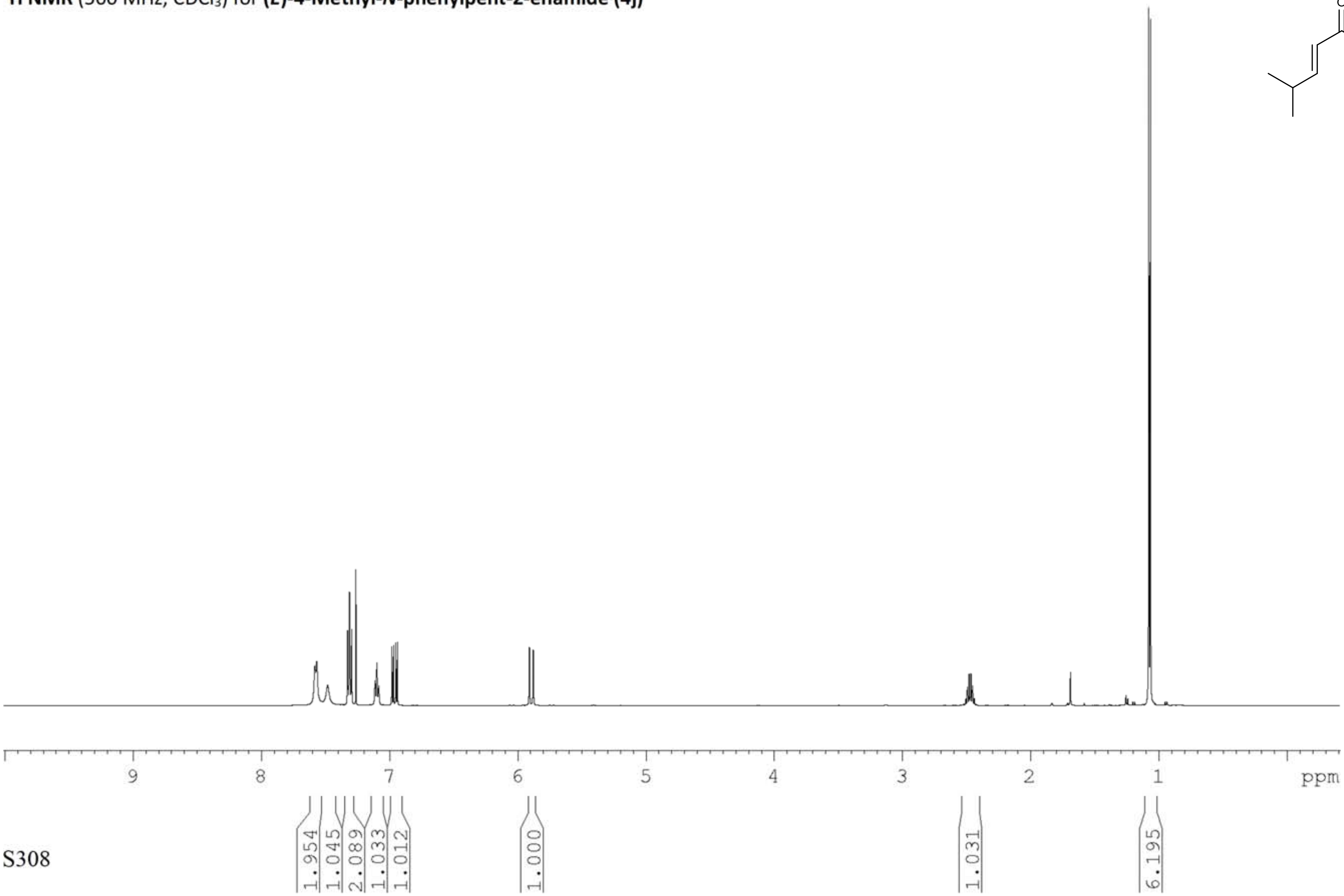

^{13}C NMR (126 MHz, $CDCl_3$) for **(*E*)-4-Methyl-*N*-phenylpent-2-enamide (4j)**

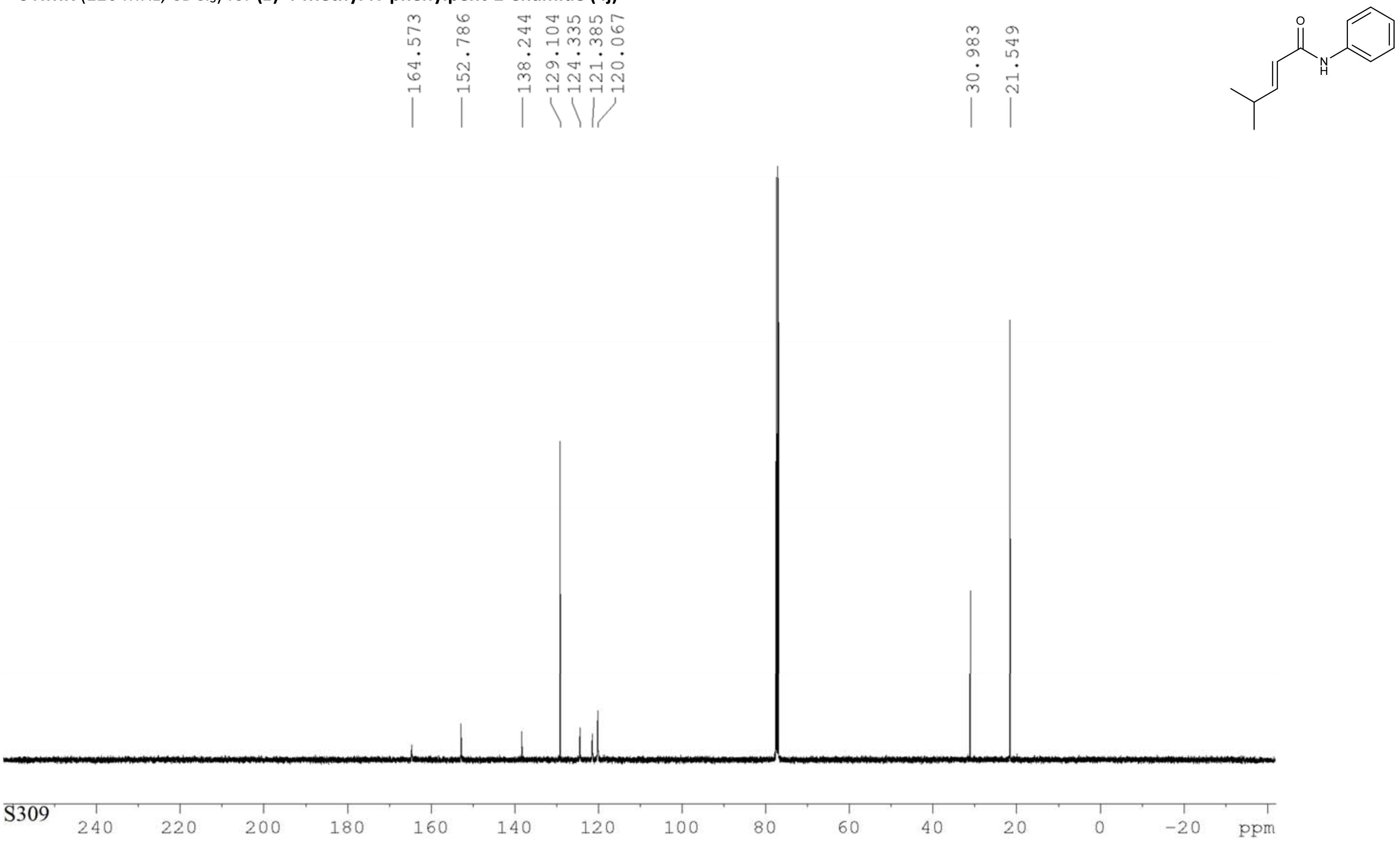

^{1}H NMR (400 MHz, $CDCl_3$) for **(*E*)-2-Methyl-*N*-phenylbut-2-enamide (4m)**

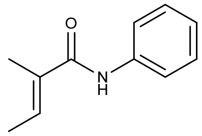

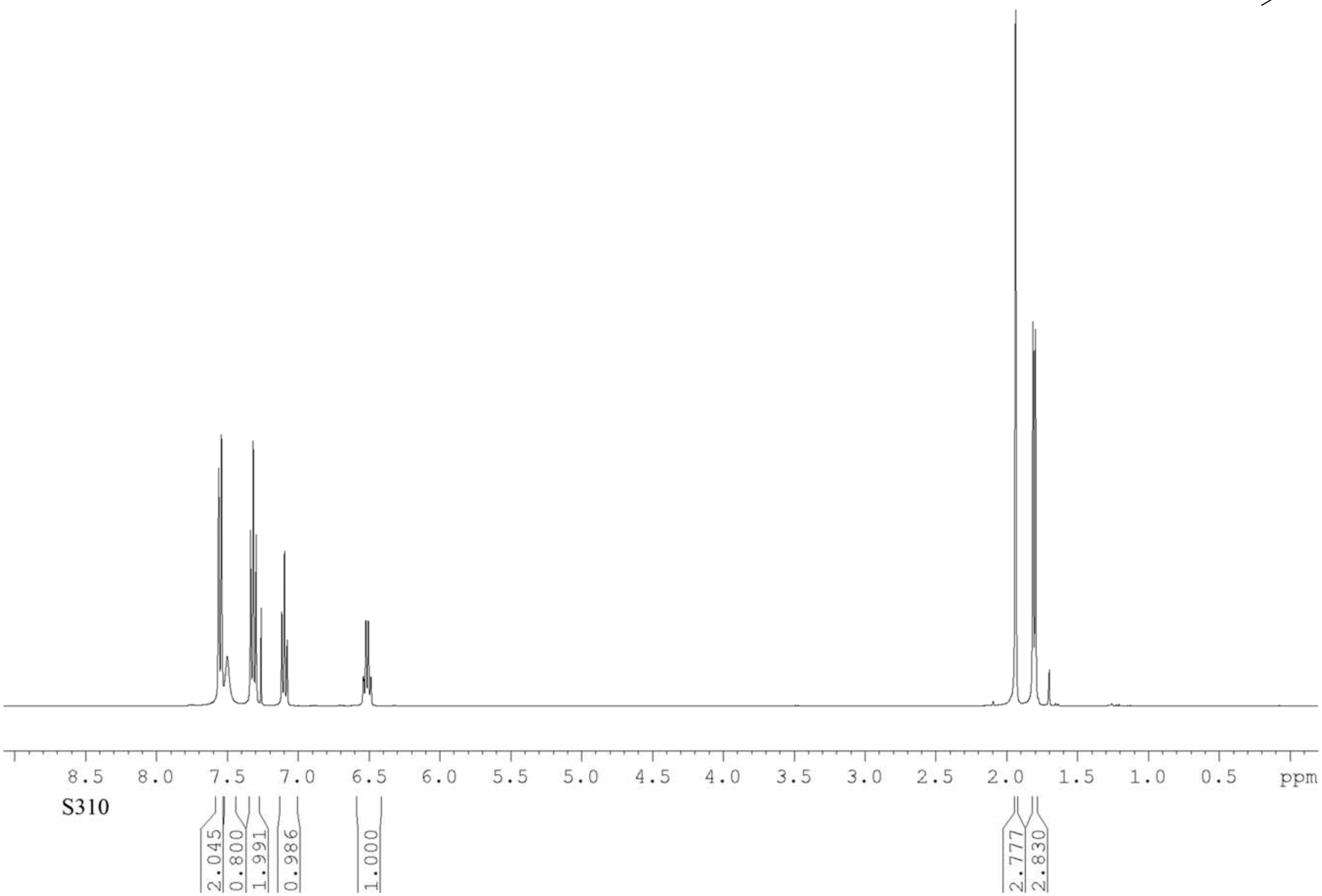

^{13}C NMR (101 MHz, $CDCl_3$) for **(*E*)-2-Methyl-*N*-phenylbut-2-enamide (4m)**

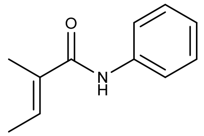

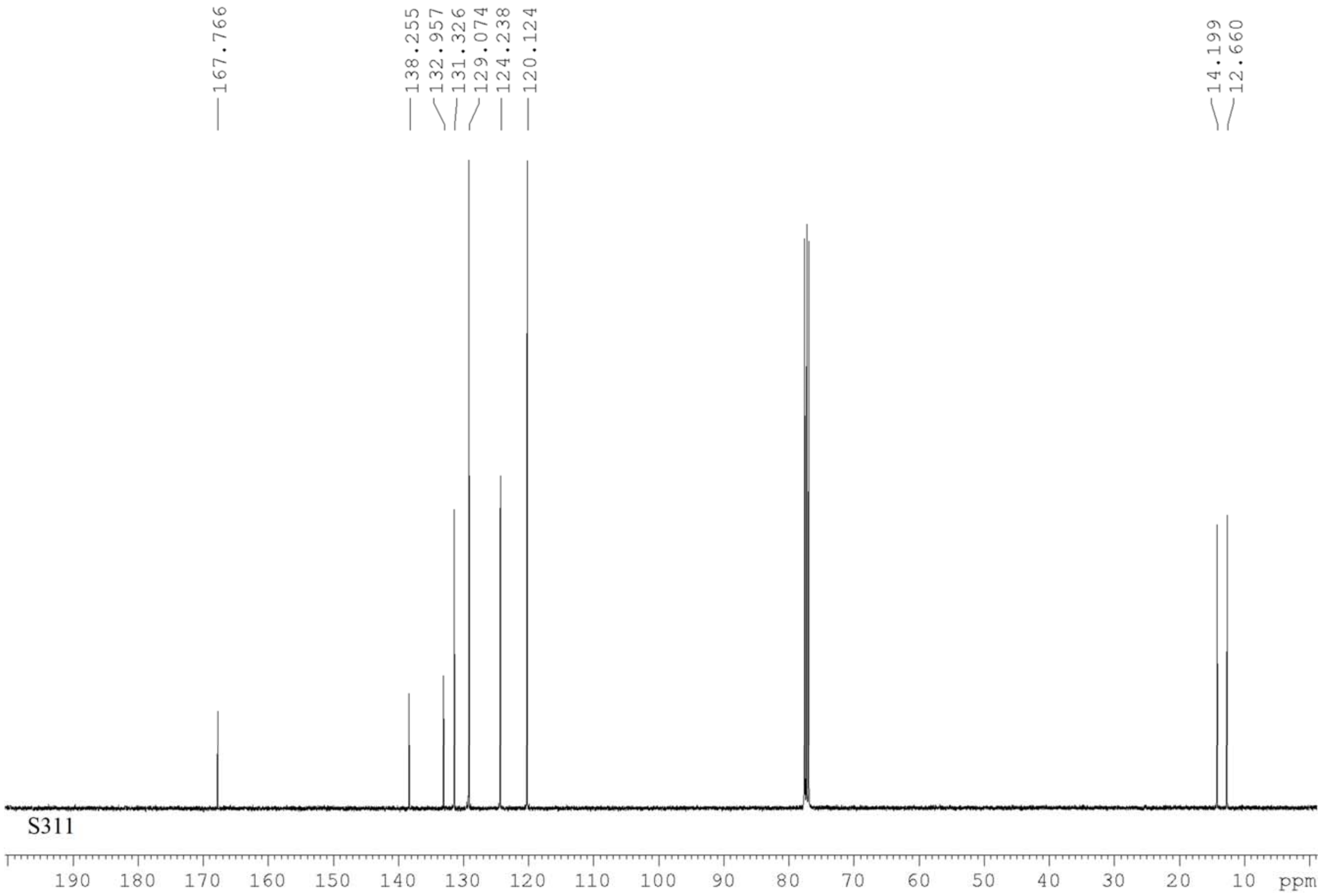

^{1}H NMR (400 MHz, $CDCl_3$) for ***N*-Phenylcyclohex-1-ene-1-carboxamide (4n)**

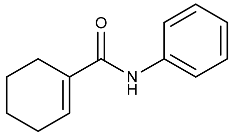

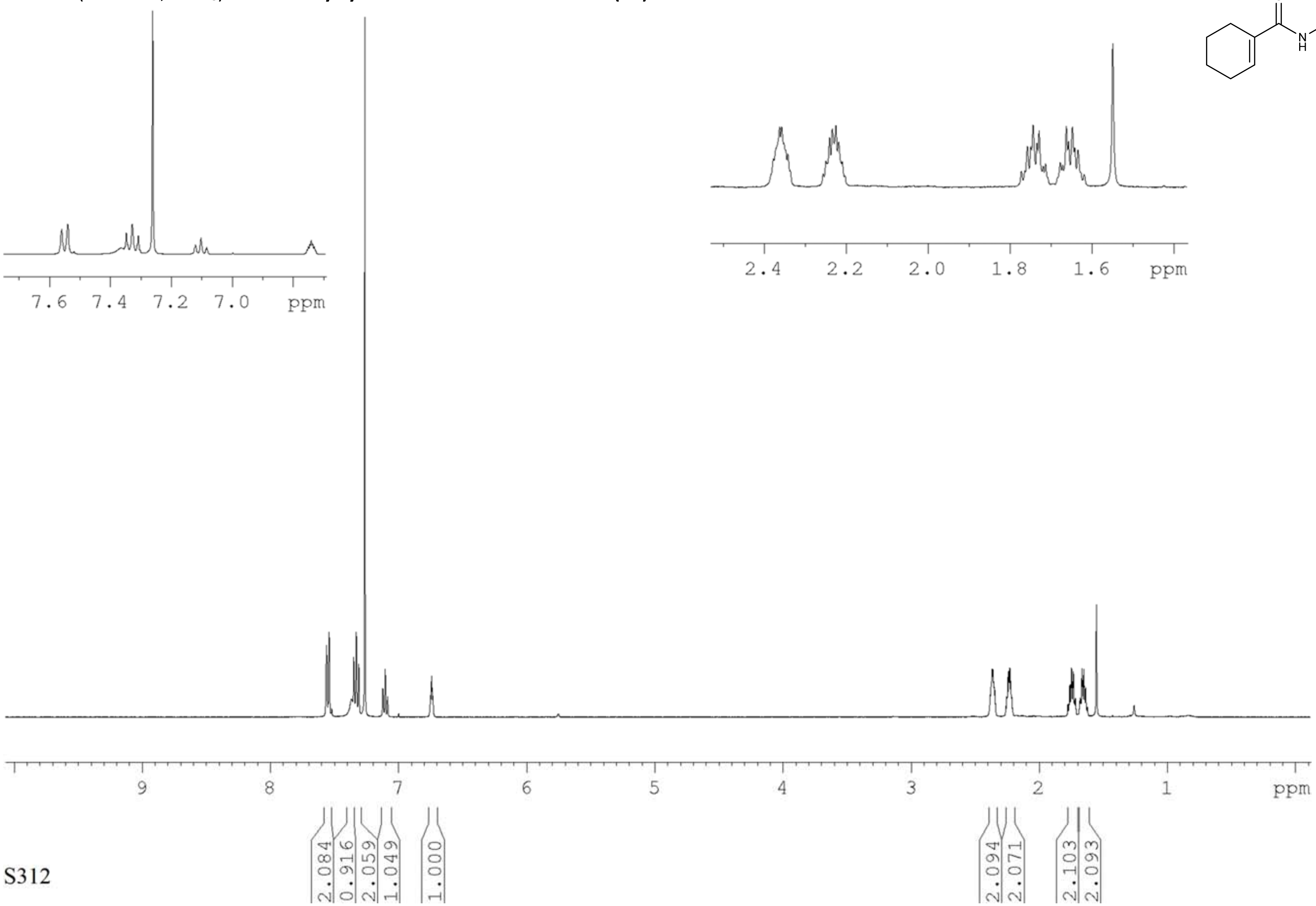

^{13}C NMR (126 MHz, $CDCl_3$) for ***N*-Phenylcyclohex-1-ene-1-carboxamide (4n)**

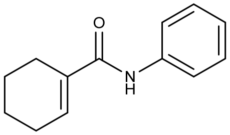

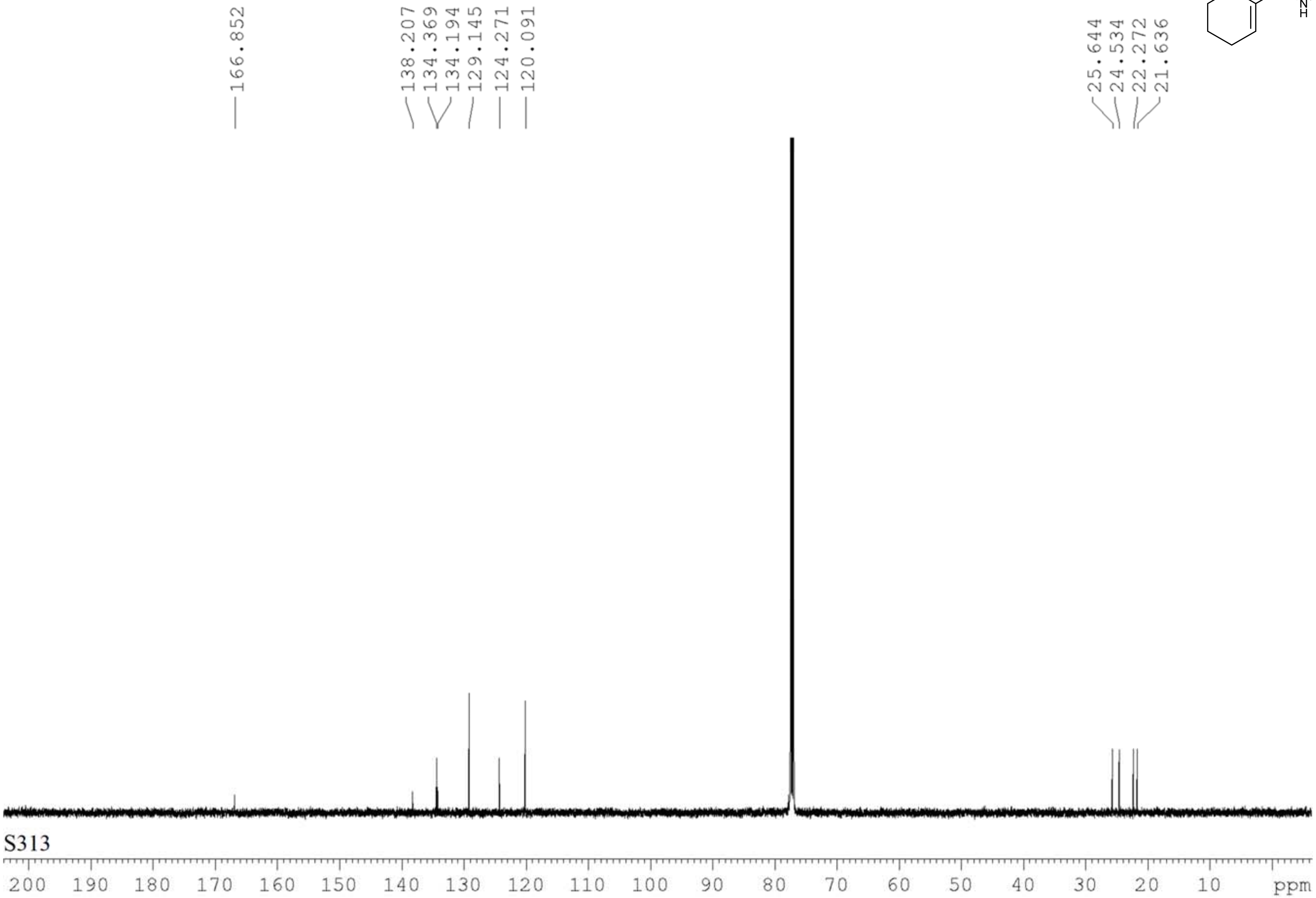

^{1}H NMR (500 MHz, CDCl$_3$) for ***(E)*-*N*,5-Diphenylpent-2-enamide (4l)**

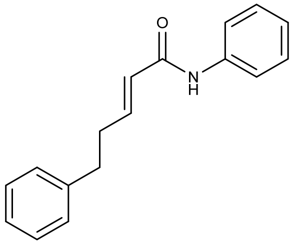

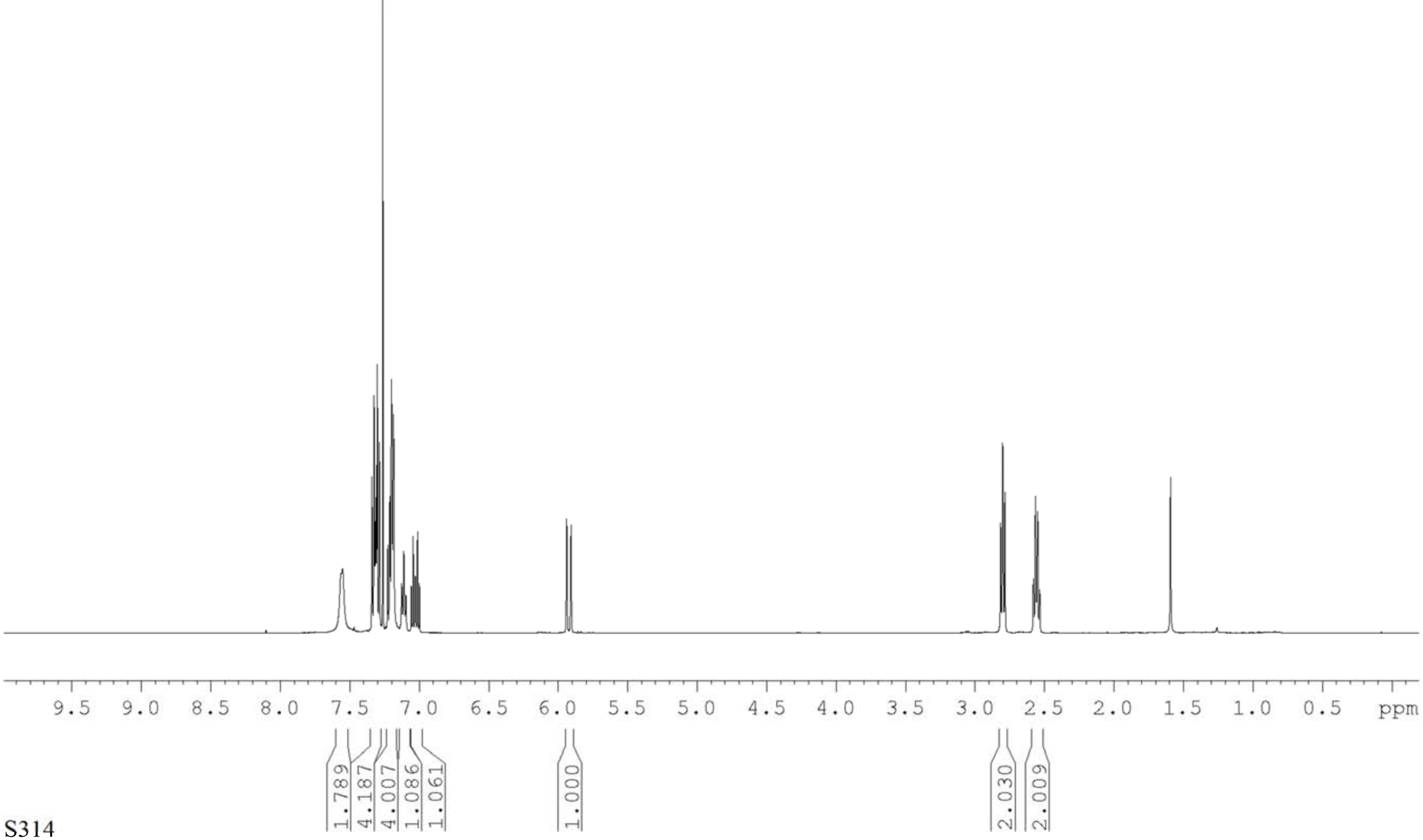

^{13}C NMR (126 MHz, $CDCl_3$) for ***(E)*-*N*,5-Diphenylpent-2-enamide (4l)**

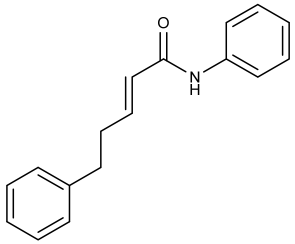

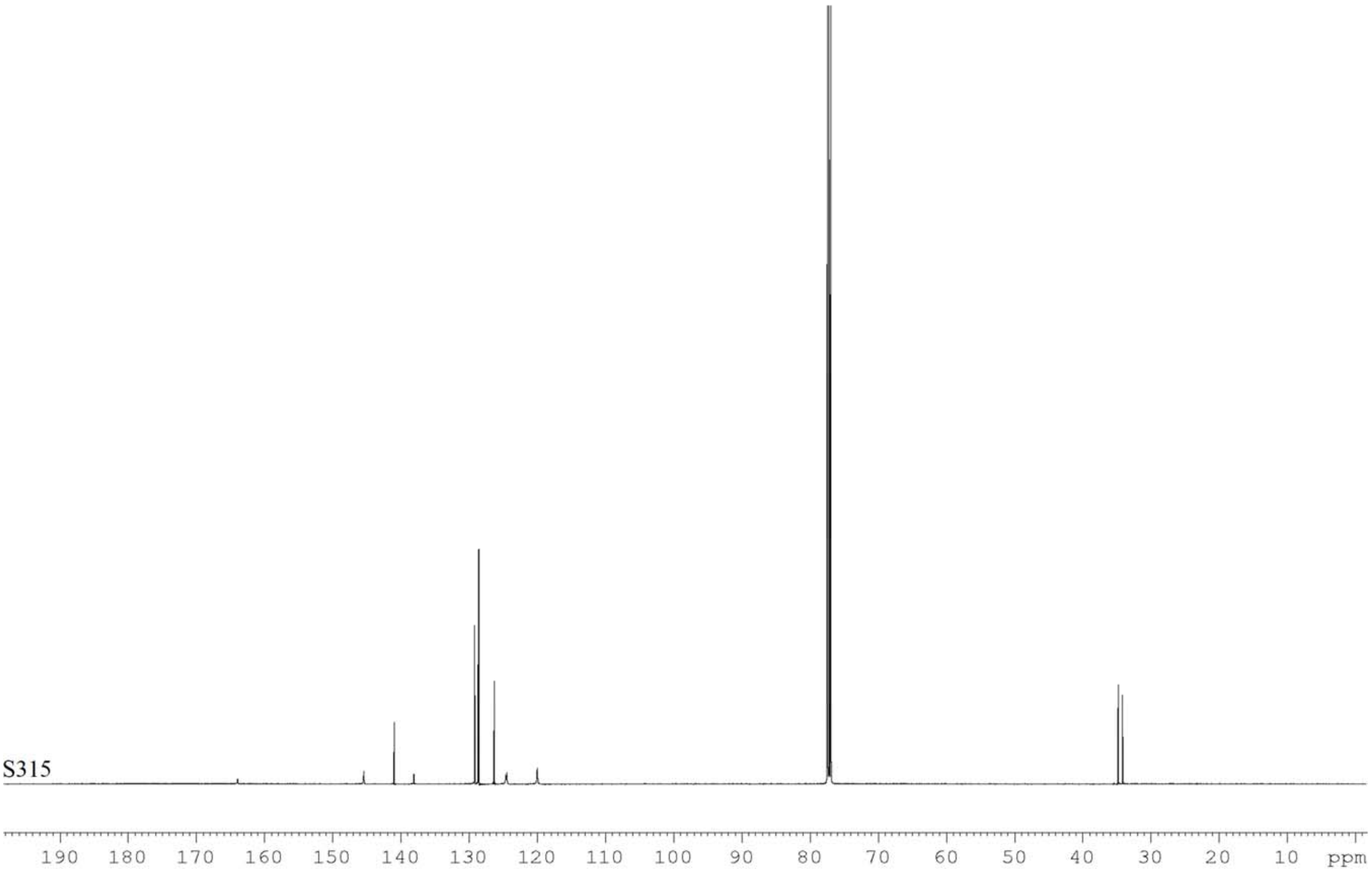

^{1}H NMR (400 MHz, DMSO-d_6) for ***N*-Vinylbenzamide (8)**

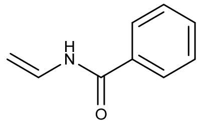

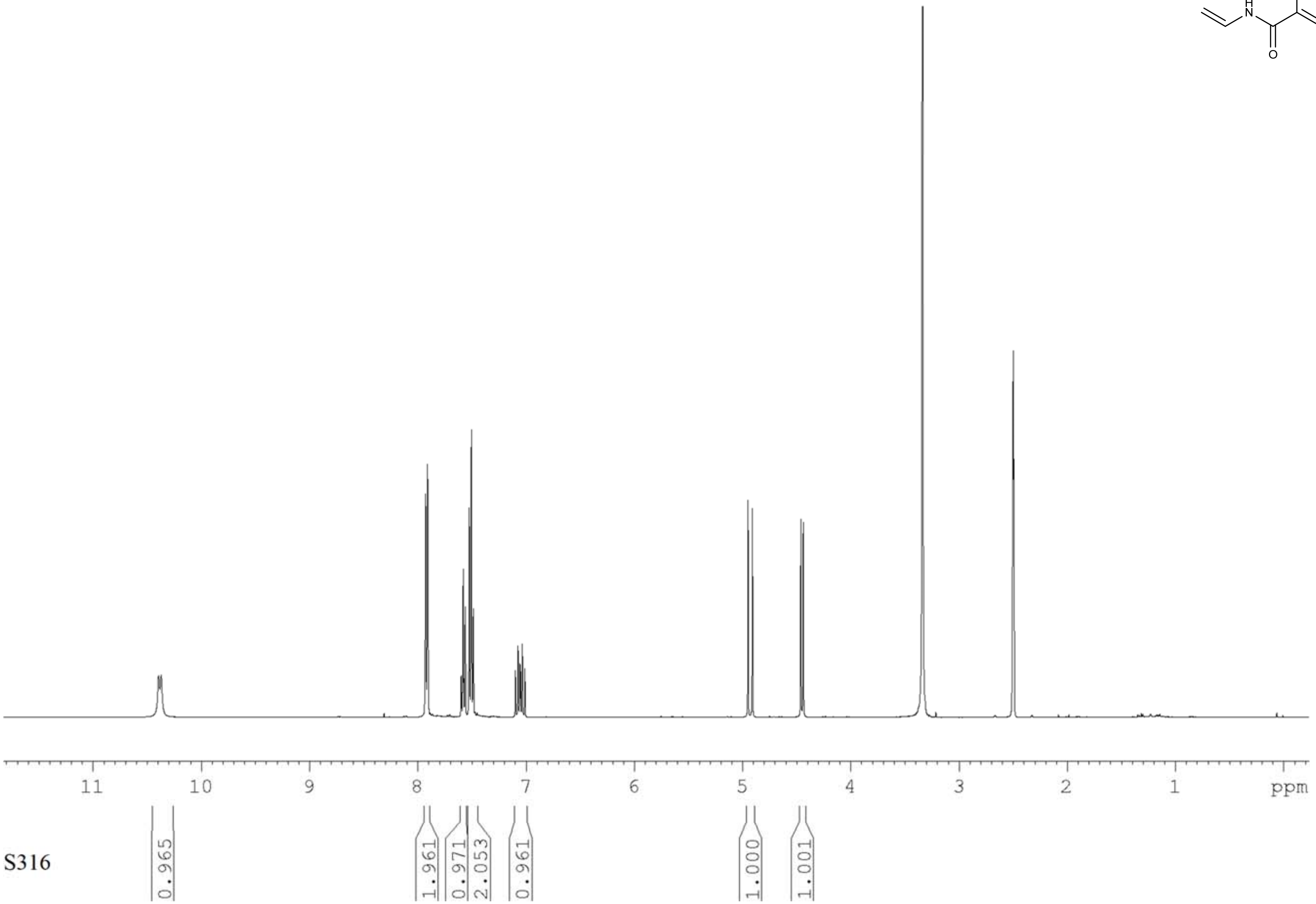

^{13}C NMR (101 MHz, DMSO-d_6) for ***N*-Vinylbenzamide (8)**

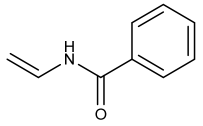

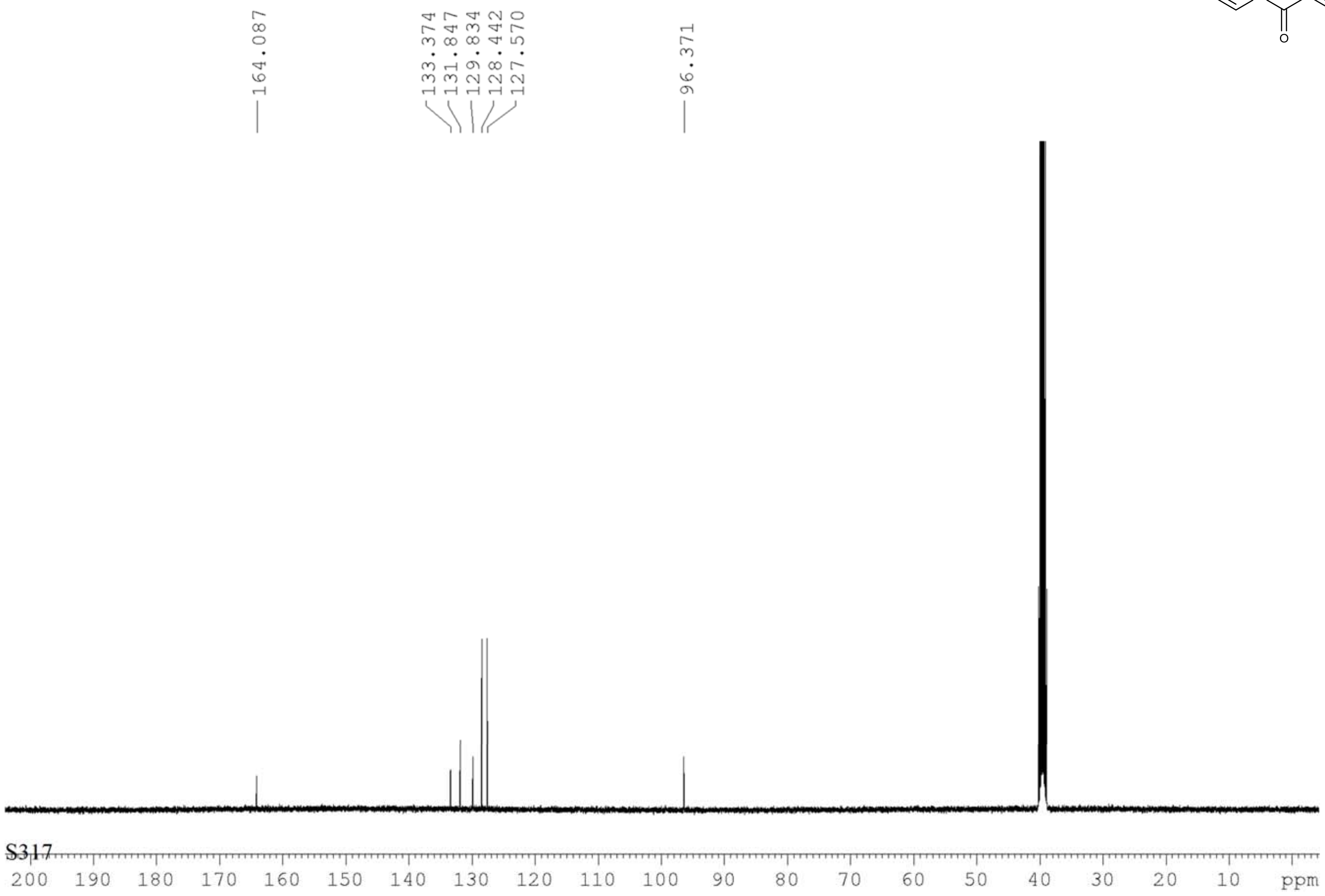

^{1}H NMR (400 MHz, $CDCl_3$) for **2-(Pyrrolidin-1-yl)pyridine (1a)**

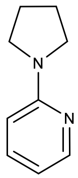

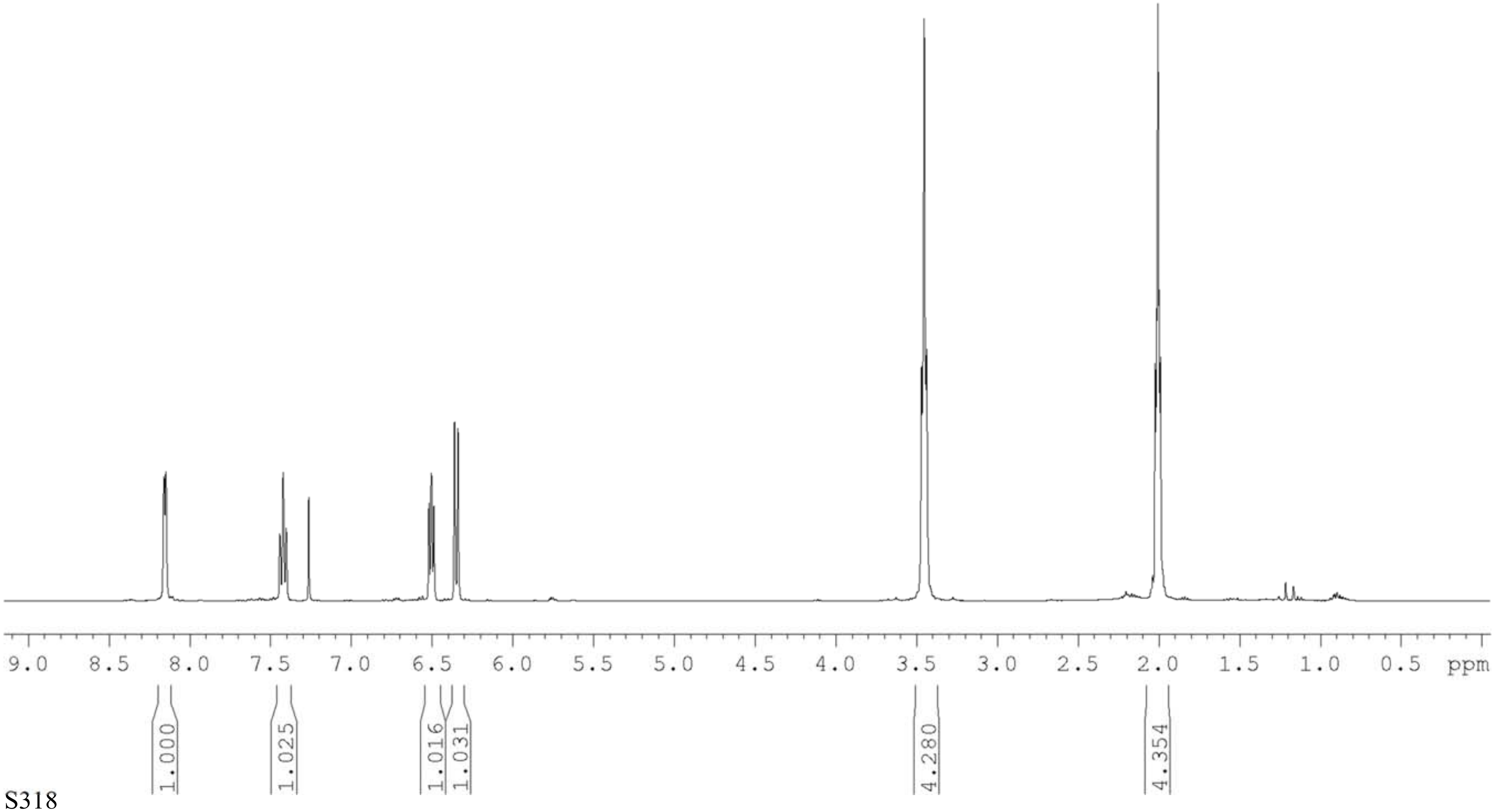

^{13}C NMR (101 MHz, $CDCl_3$) for **2-(Pyrrolidin-1-yl)pyridine (1a)**

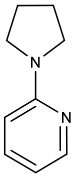

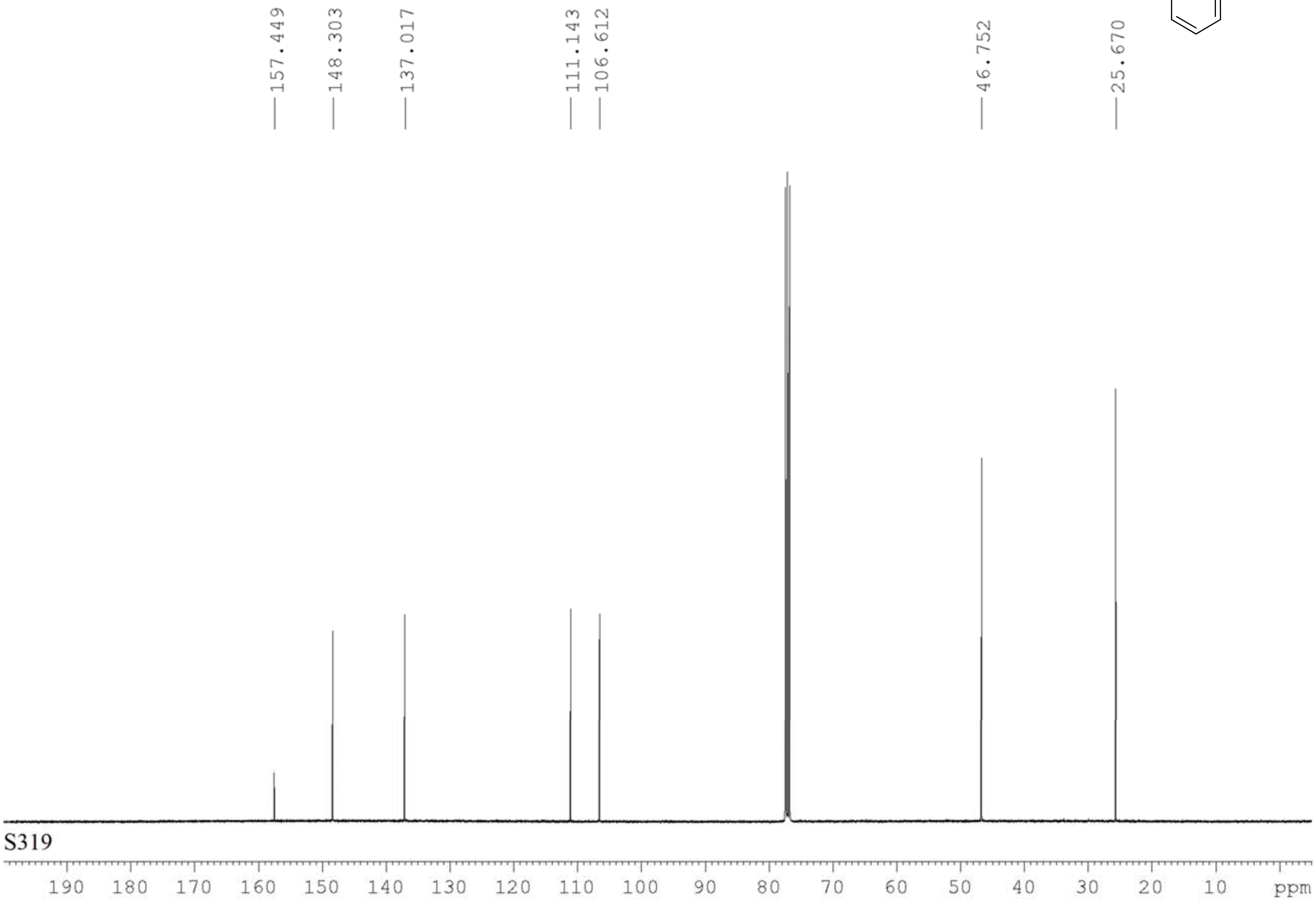

^{1}H NMR (400 MHz, $CDCl_3$) for **2-(Pyrrolidin-1-yl)pyrimidine (1e)**

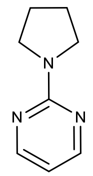

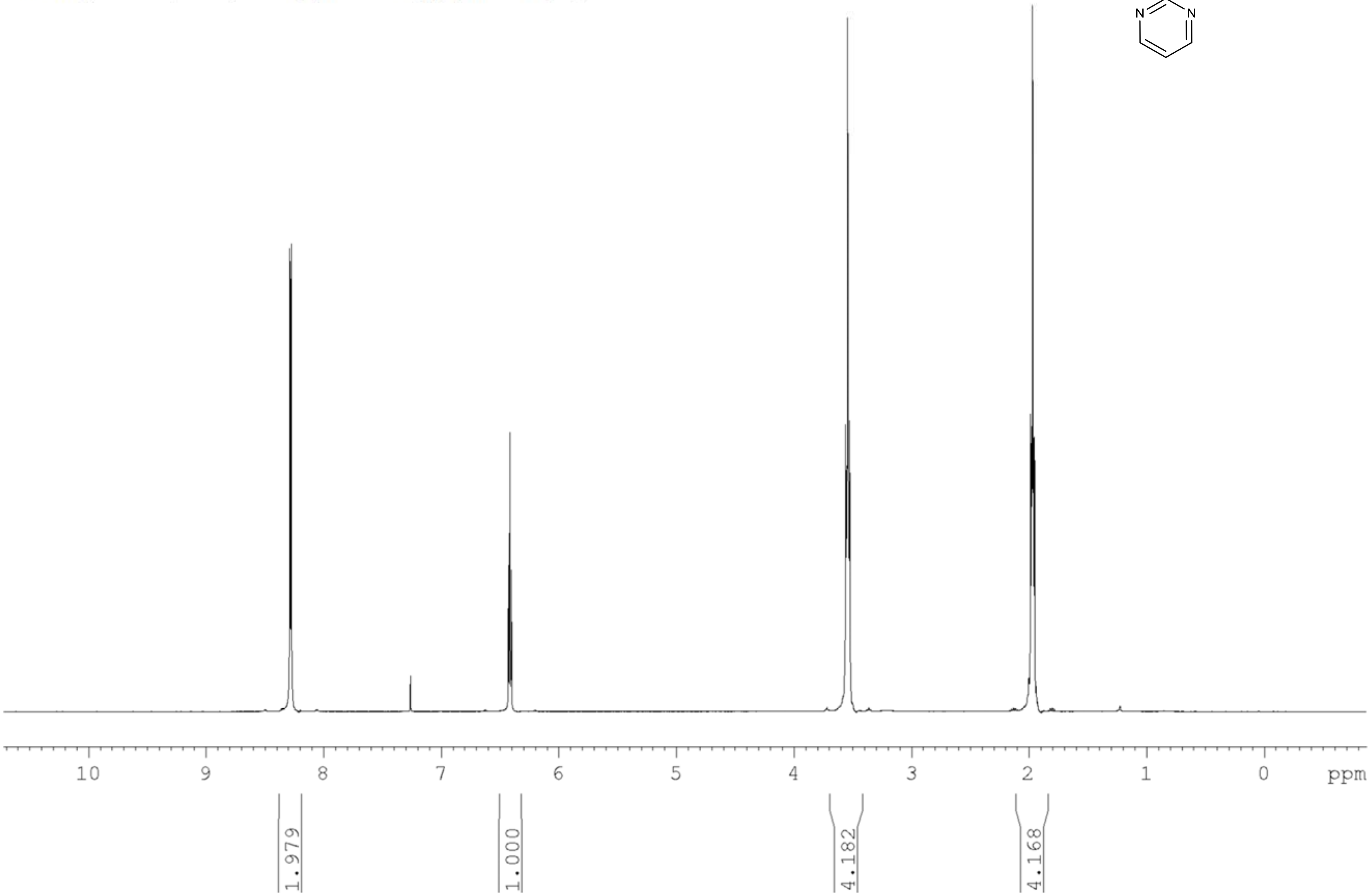

^{13}C NMR (101 MHz, $CDCl_3$) for **2-(Pyrrolidin-1-yl)pyrimidine (1e)**

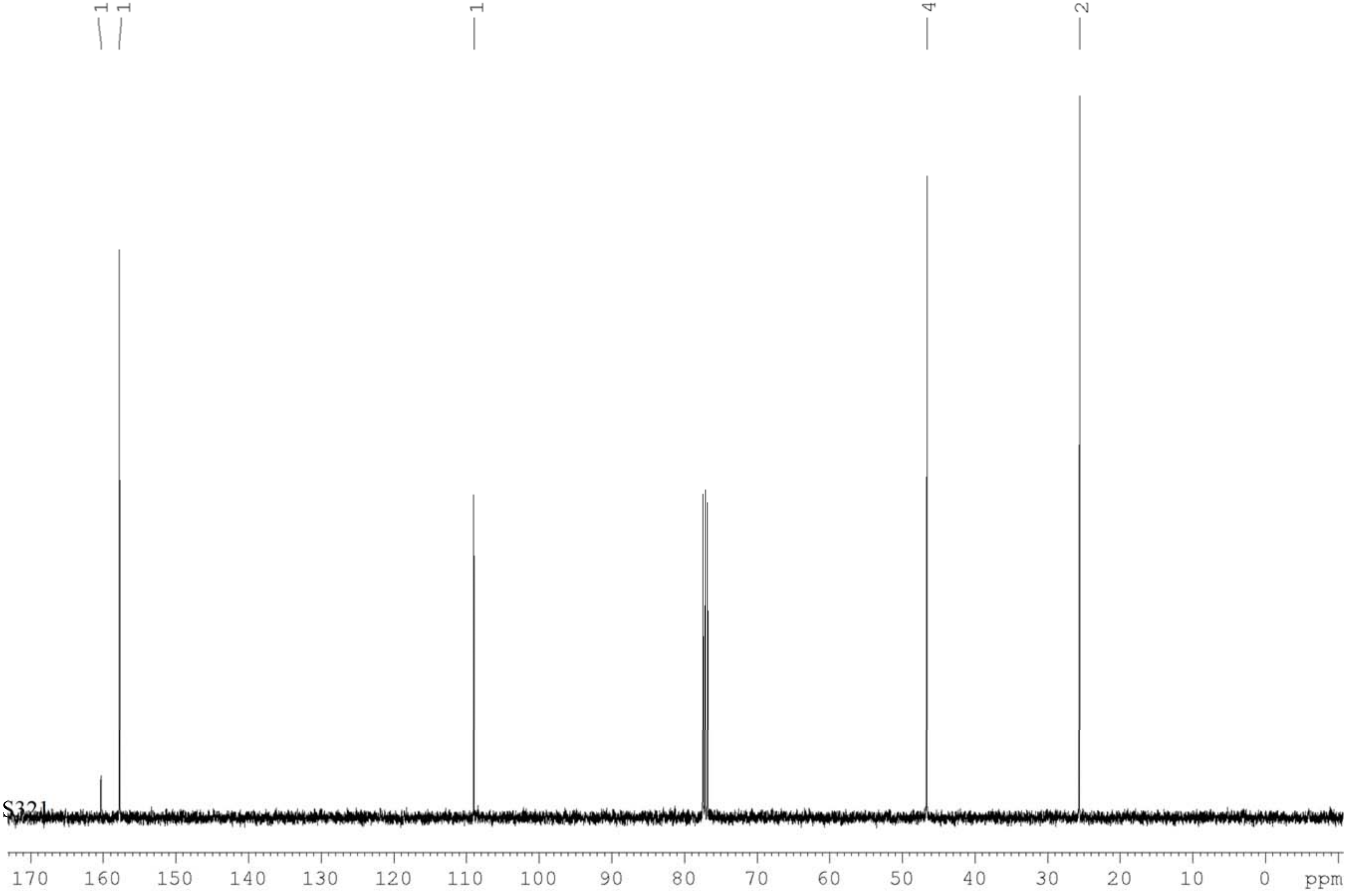

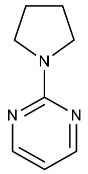

^{1}H NMR (400 MHz, $CDCl_3$) for **3-(Pyrrolidin-1-yl)pyridine (1n)**

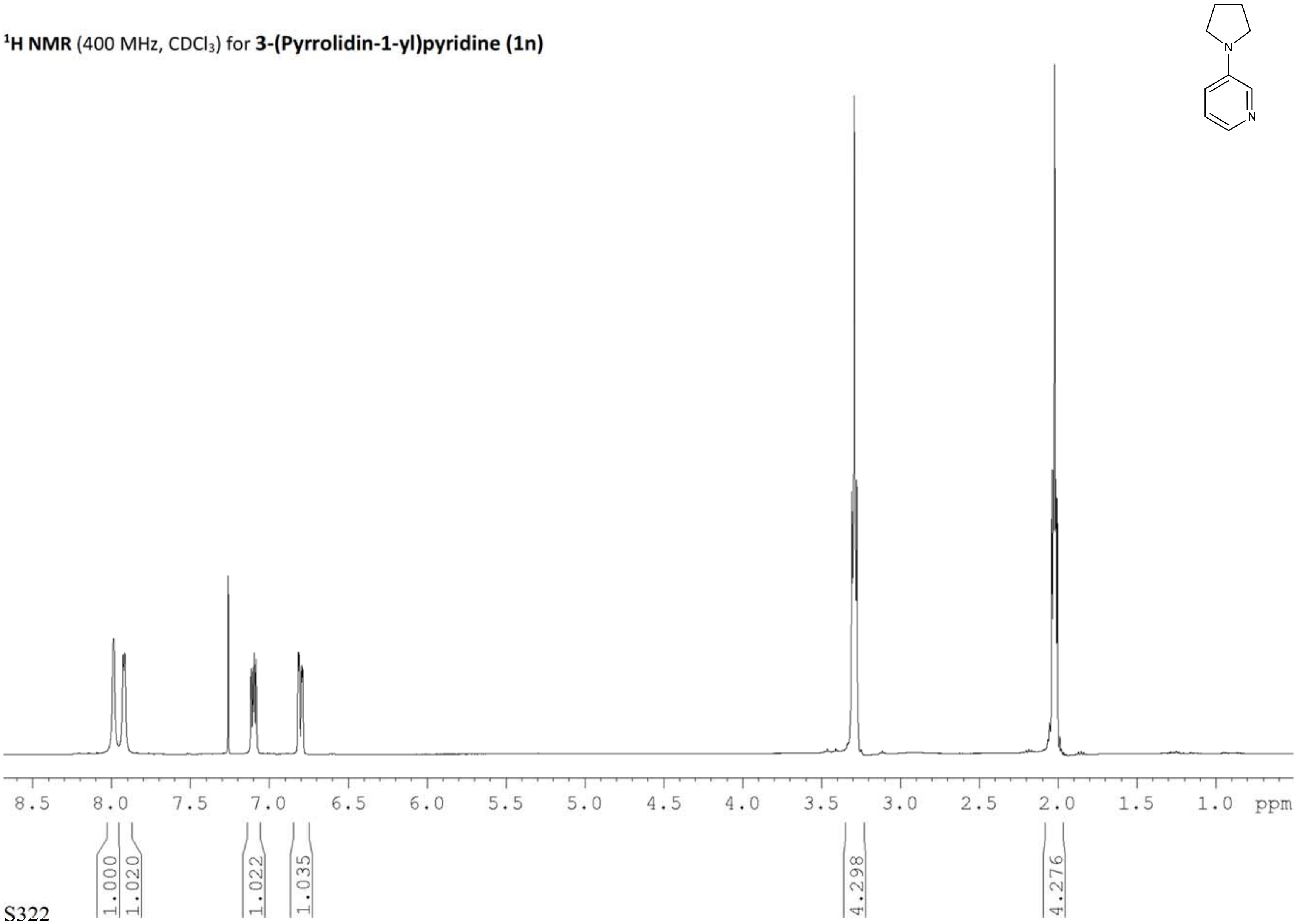

^{13}C NMR (101 MHz, $CDCl_3$) for **3-(Pyrrolidin-1-yl)pyridine (1n)**

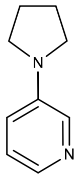

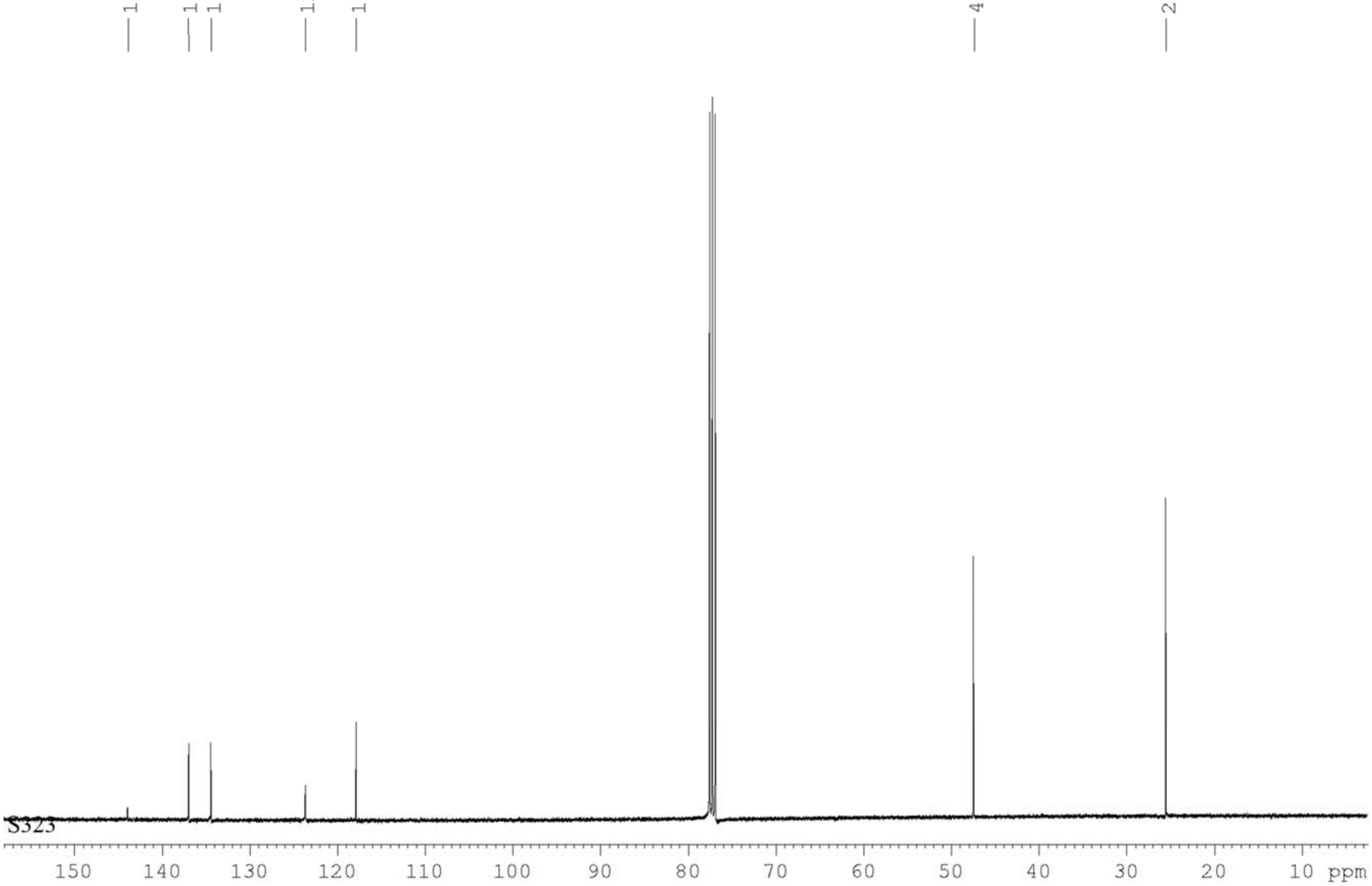

^{1}H NMR (500 MHz, $CDCl_3$) for ***N*-Ethyl-*N*-phenyl-2-aminopyridine (1d)**

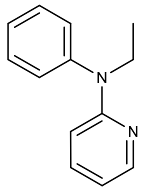

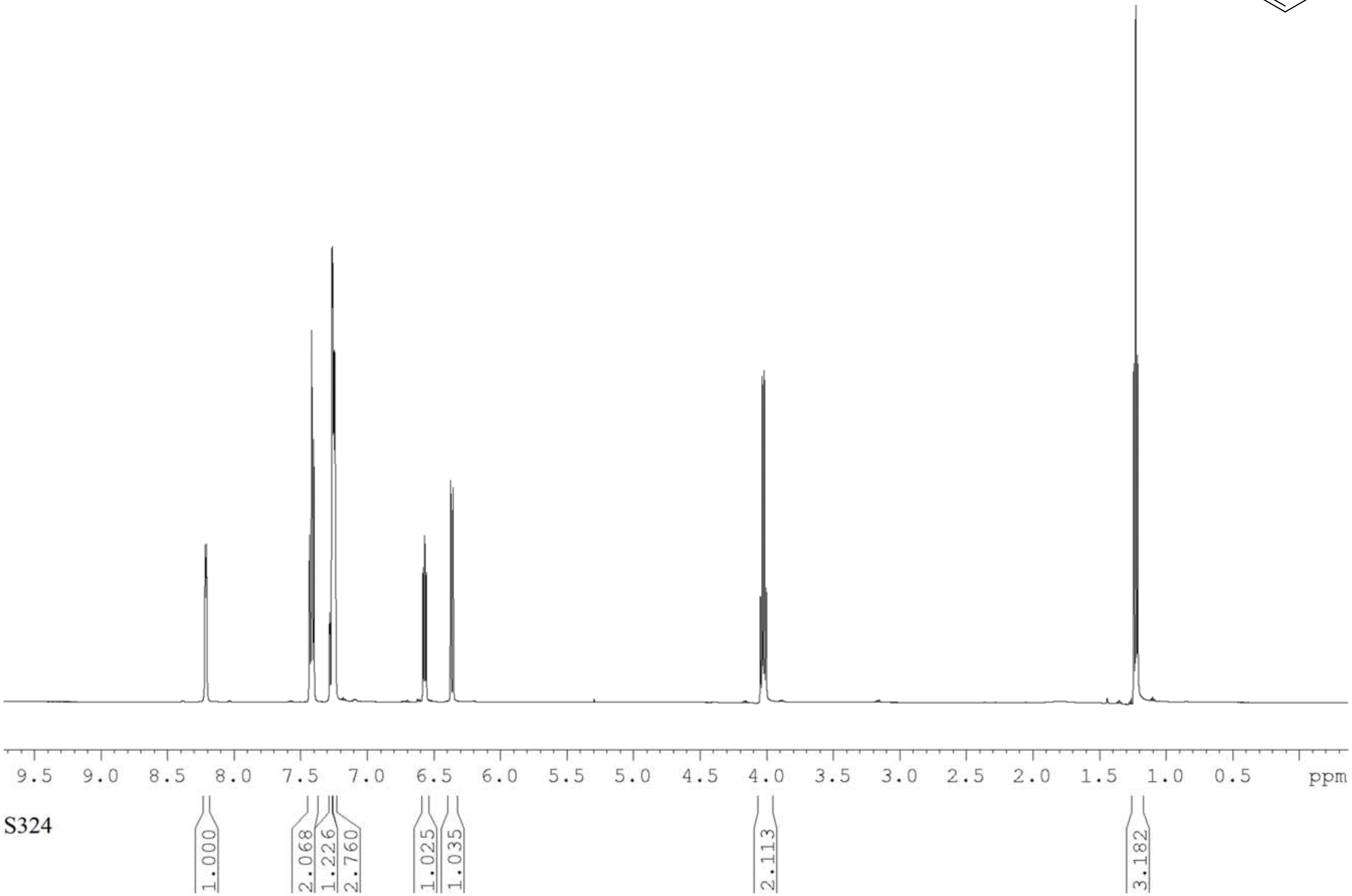

^{13}C NMR (126 MHz, $CDCl_3$) for ***N*-Ethyl-*N*-phenyl-2-aminopyridine (1d)**

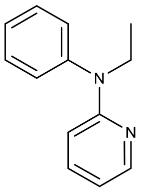

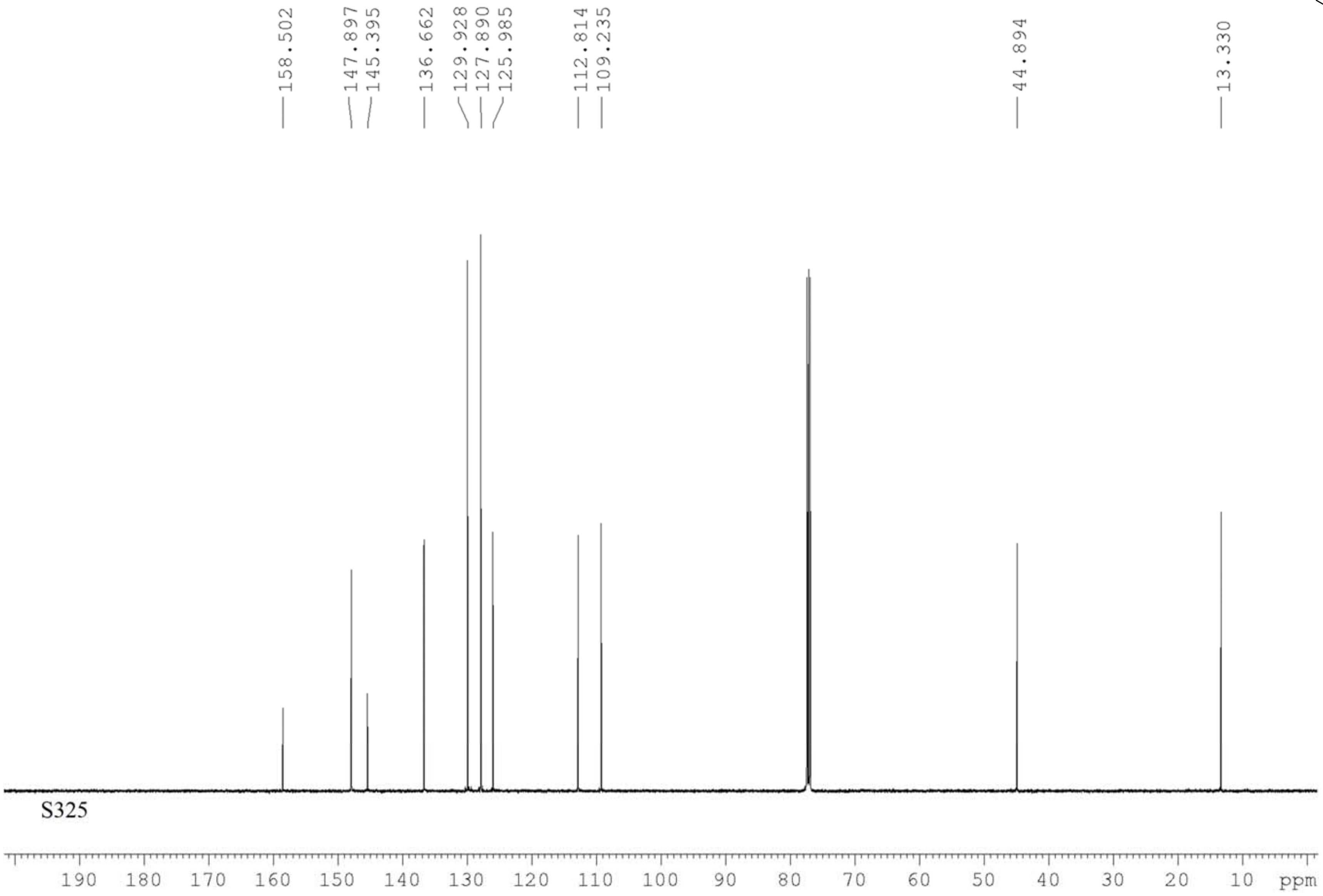

^{1}**H NMR** (500 MHz, $CDCl_3$) for ***N*-Benzyl-*N*-ethyl-2-aminopyridine (1i)**

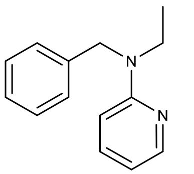

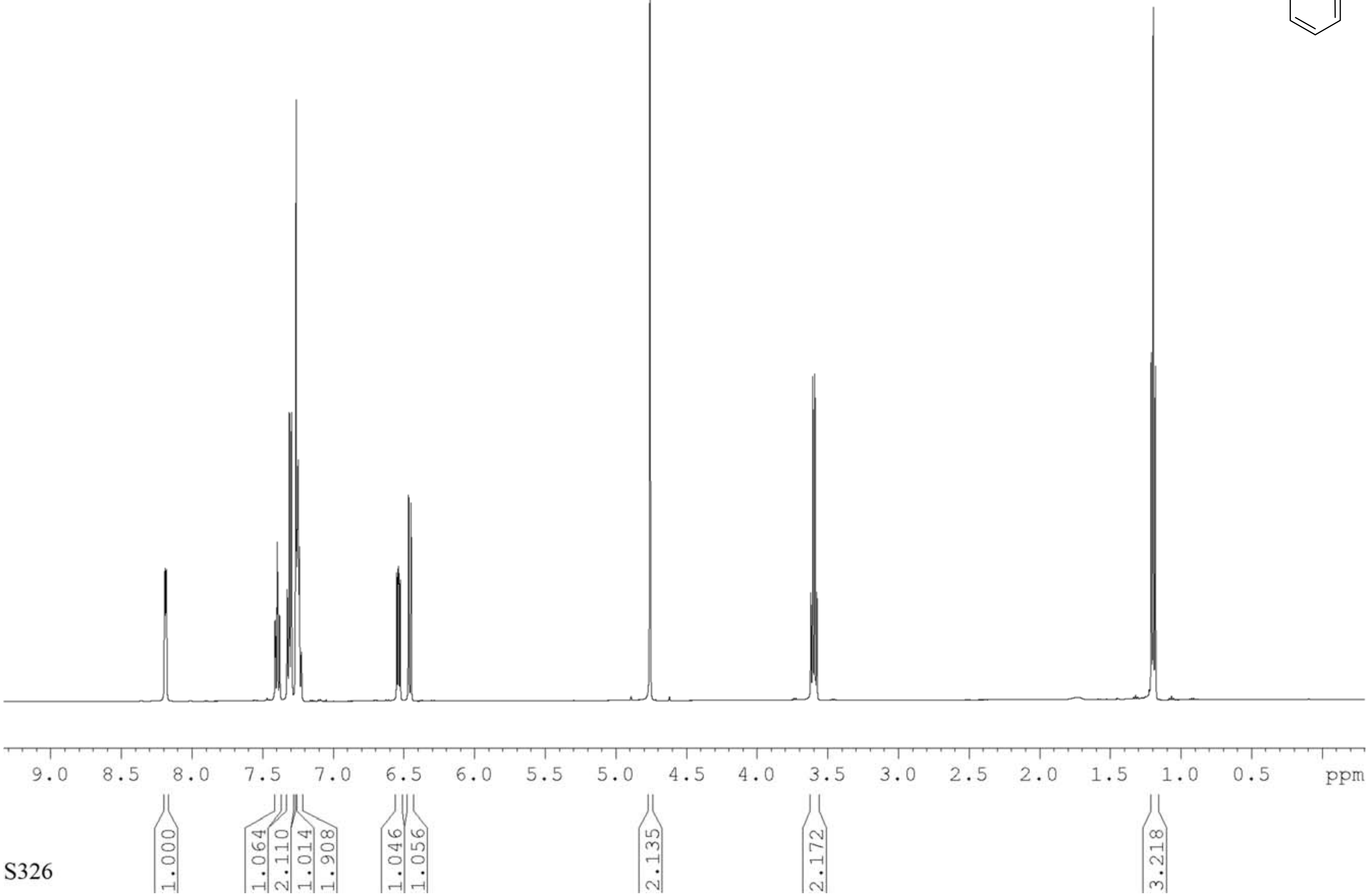

^{13}C NMR (126 MHz, $CDCl_3$) for ***N*-Benzyl-*N*-ethyl-2-aminopyridine (1i)**

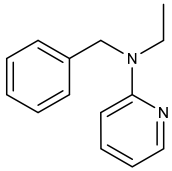

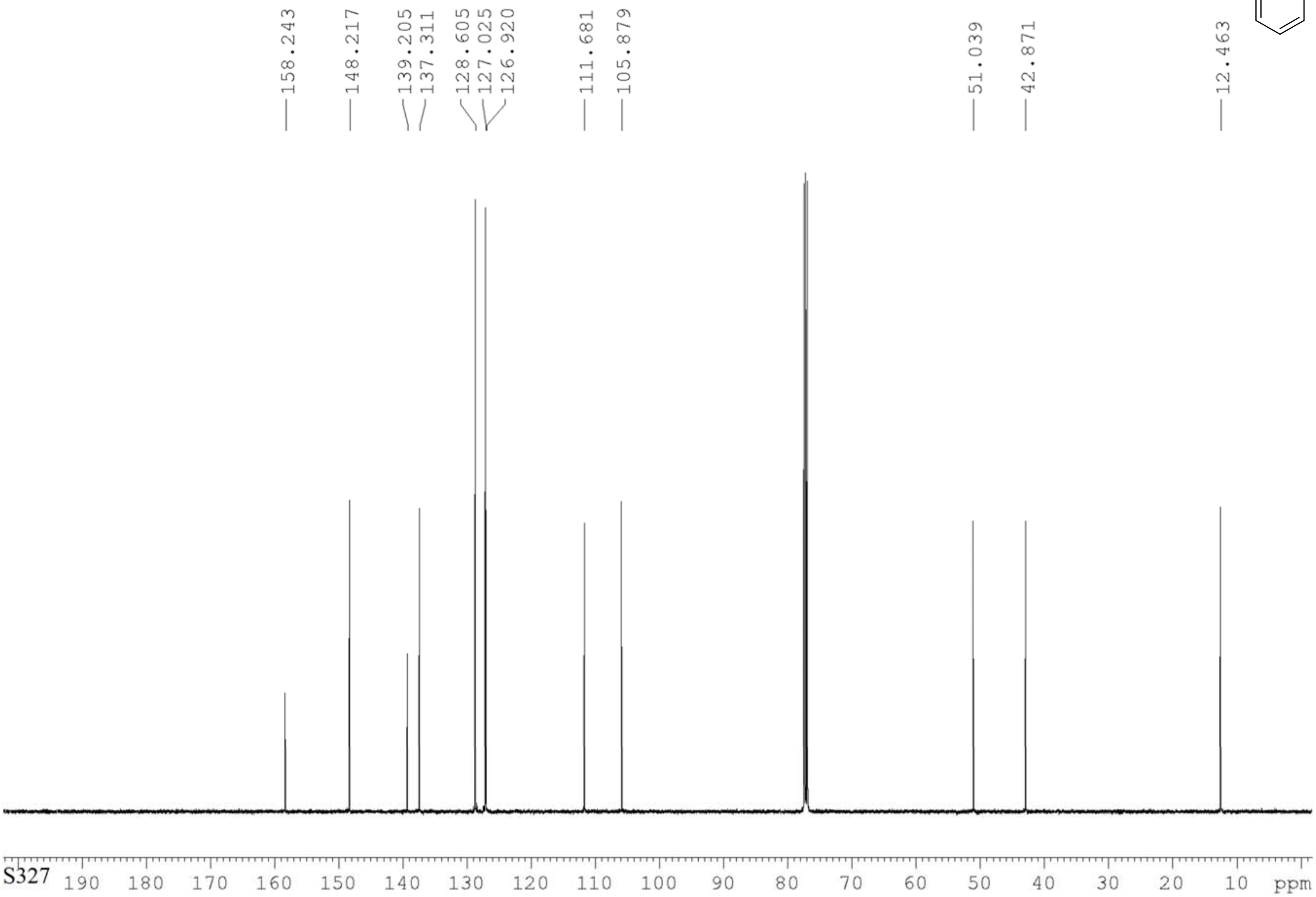

^{1}H NMR (500 MHz, $CDCl_3$) for ***N,N*-Diethyl-2-aminopyridine (1h)**

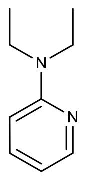

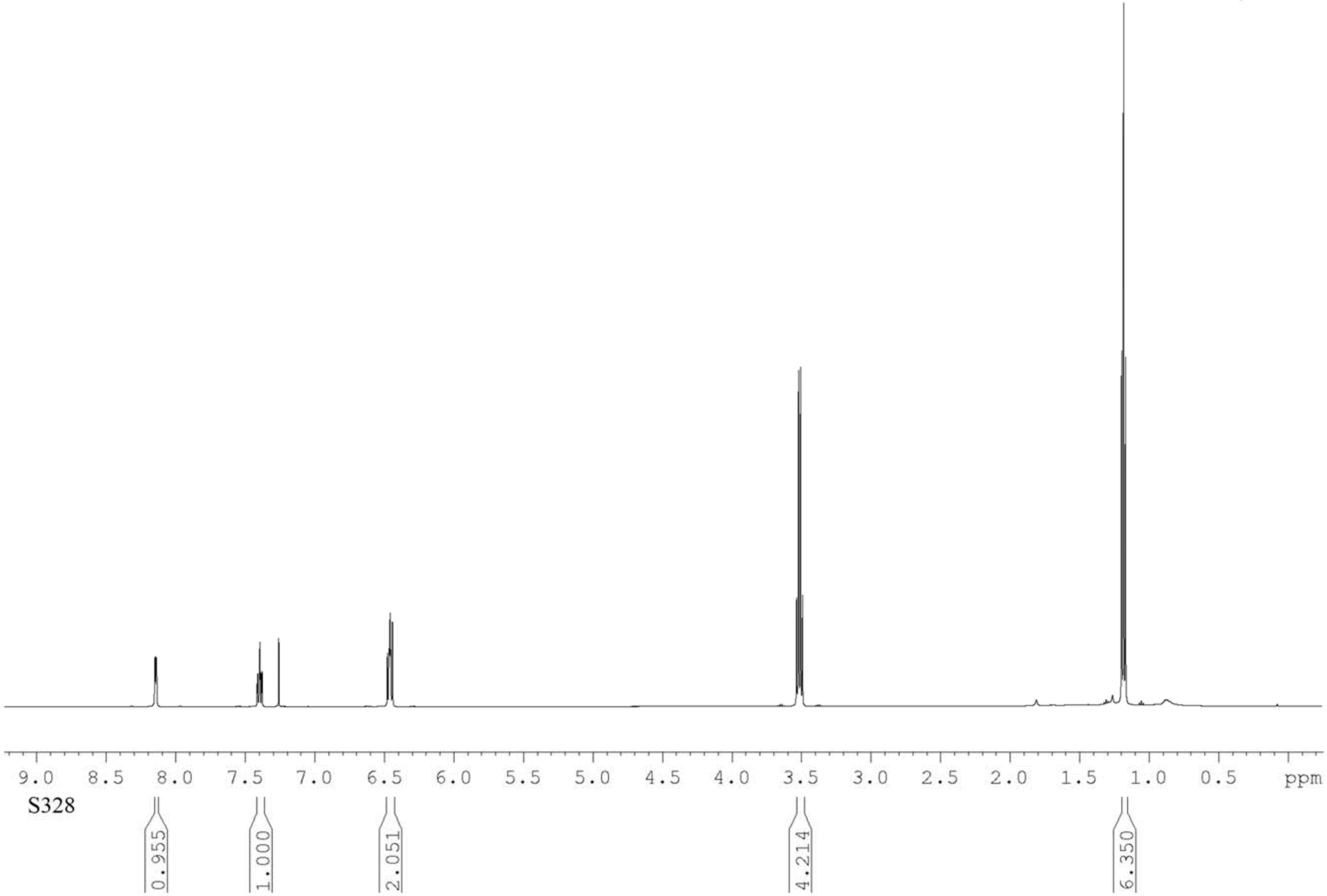

^{13}C NMR (126 MHz, $CDCl_3$) for ***N,N*-Diethyl-2-aminopyridine (1h)**

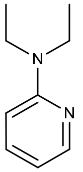

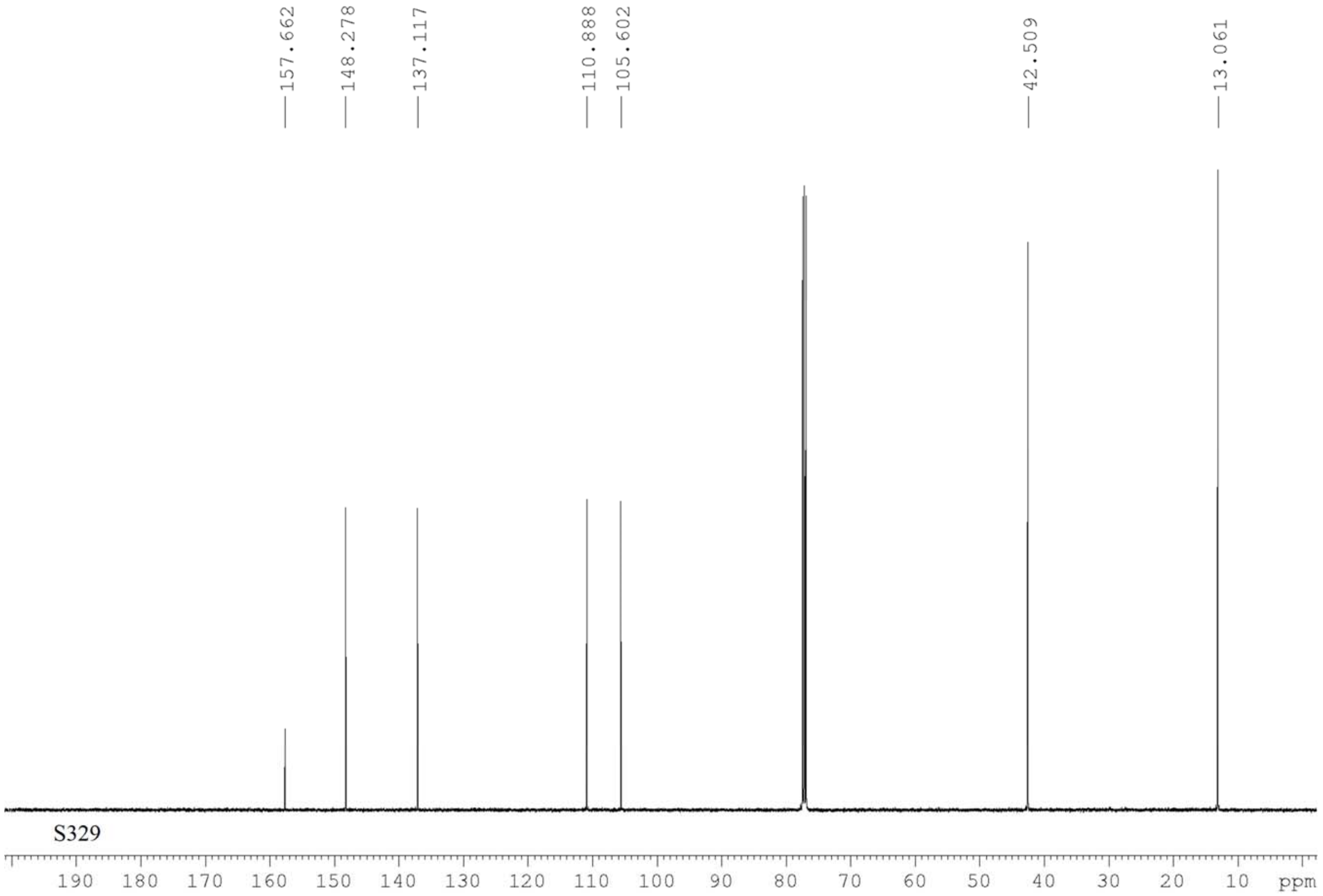

^{1}H NMR (500 MHz, $CDCl_3$) for **2-(Azetidin-1-yl)pyridine (1m)**

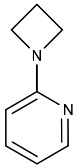

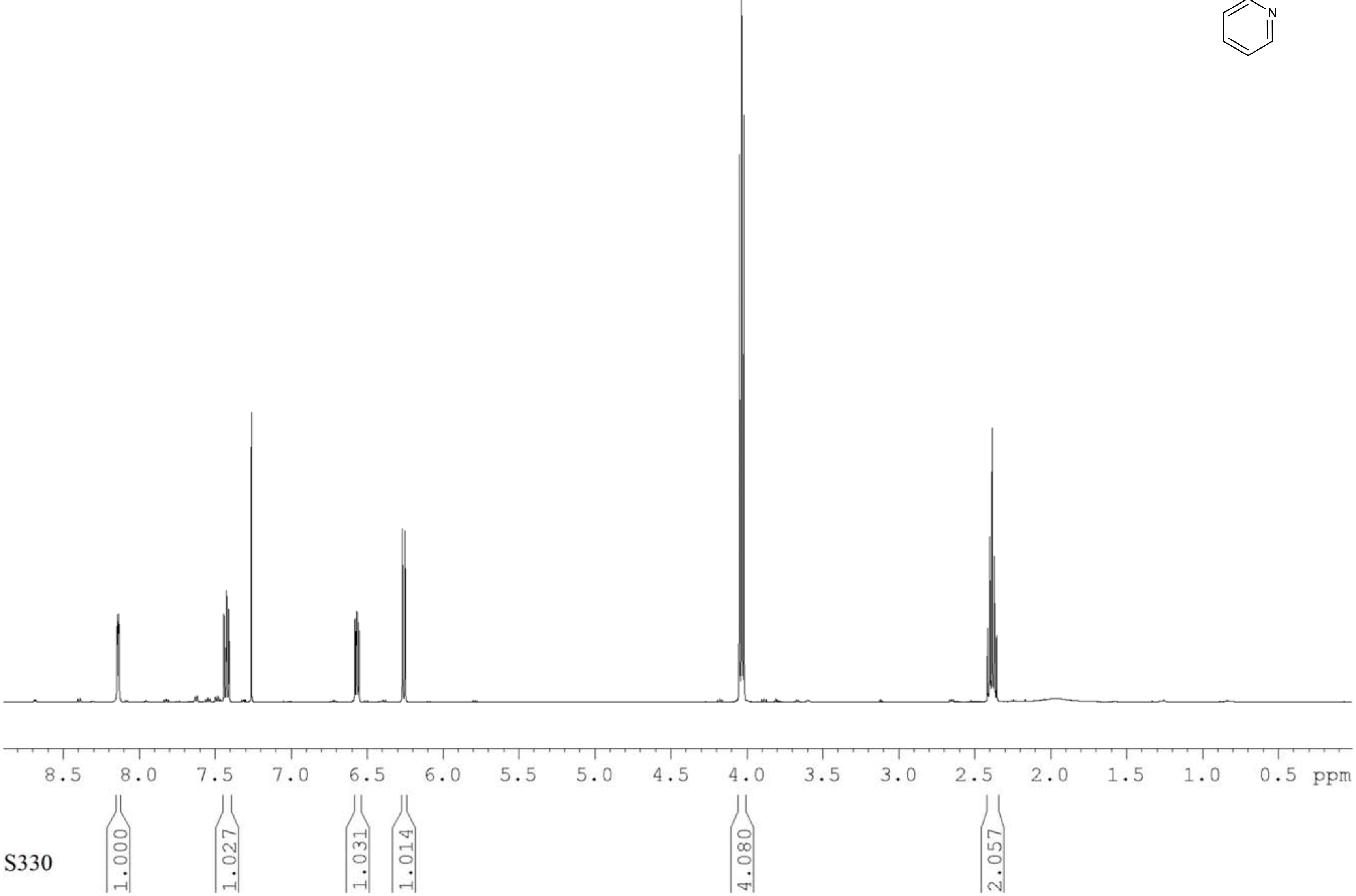

^{13}**C NMR** (126 MHz, $CDCl_3$) for **2-(Azetidin-1-yl)pyridine (1m)**

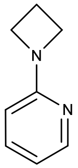

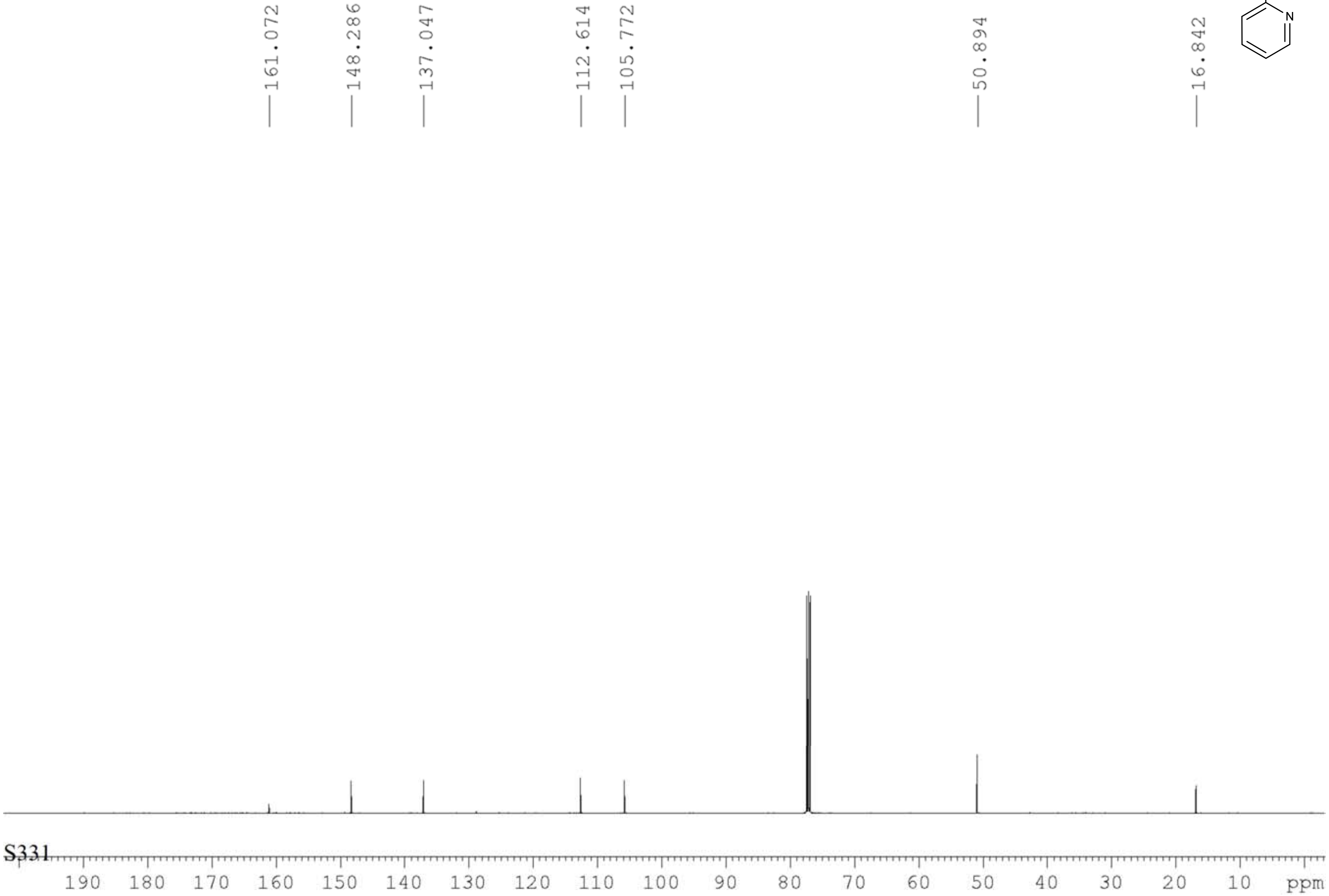

^{1}H NMR (700 MHz, $CDCl_3$) for ***N*-Methyl-*N*-phenyl-2-aminopyridine (1j)**

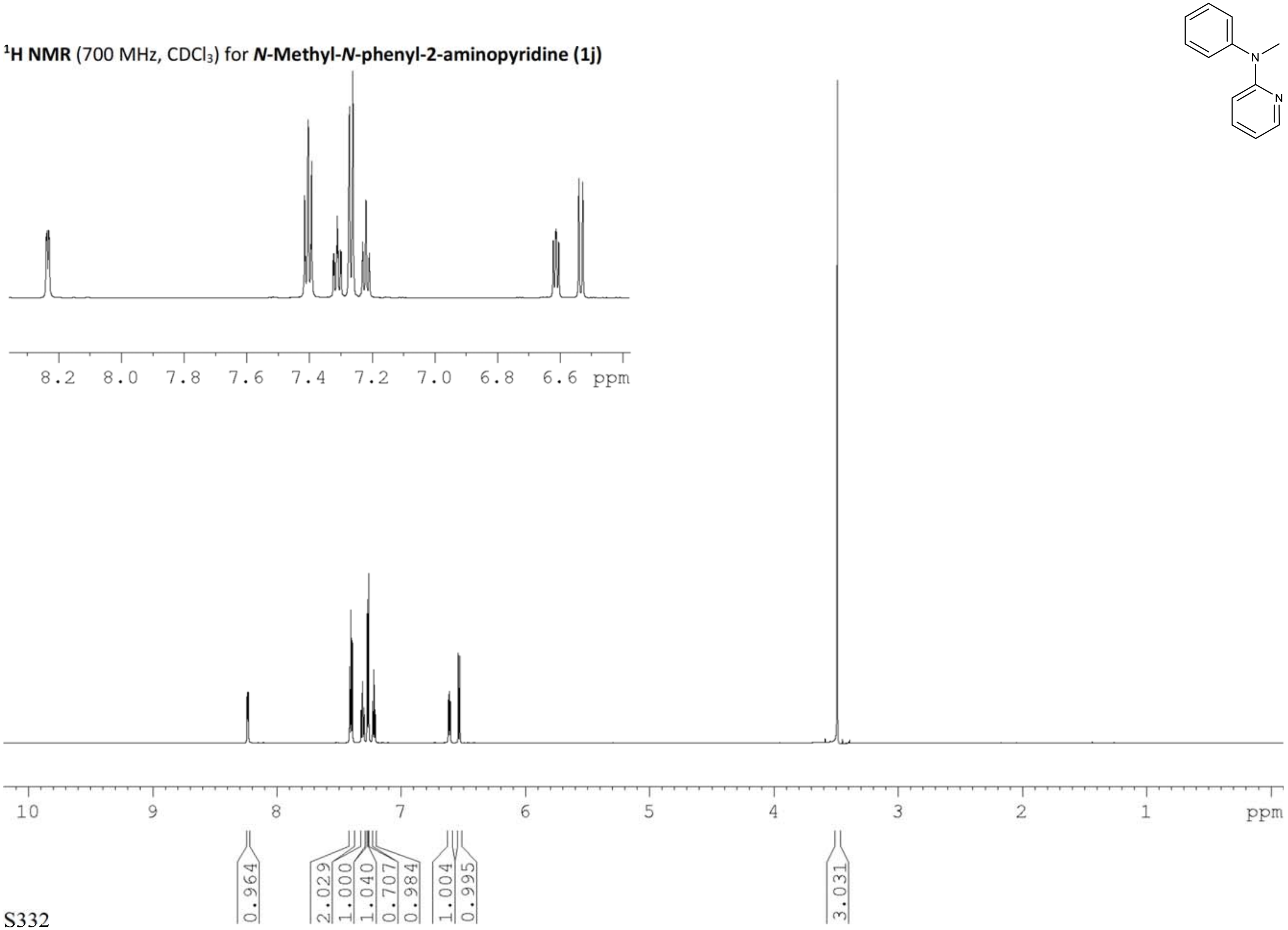

^{13}C NMR (176 MHz, $CDCl_3$) for ***N*-Methyl-*N*-phenyl-2-aminopyridine (1j)**

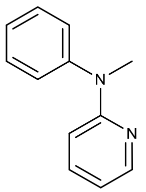

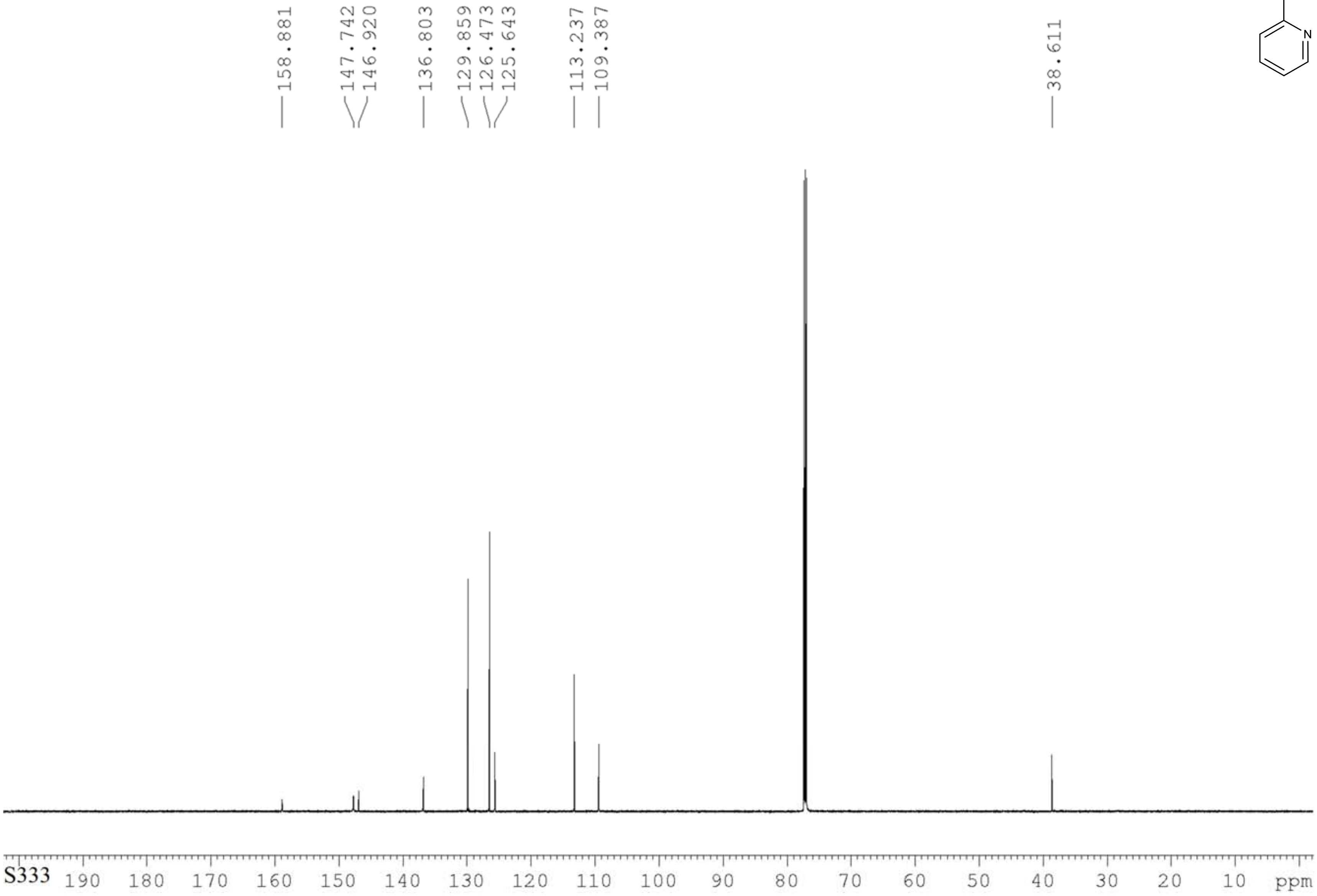

^{1}H NMR (400 MHz, $CDCl_3$) for ***N*-Benzyl-*N*-methyl-2-aminopyridine (1c)**

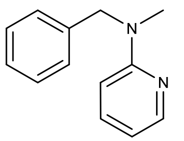

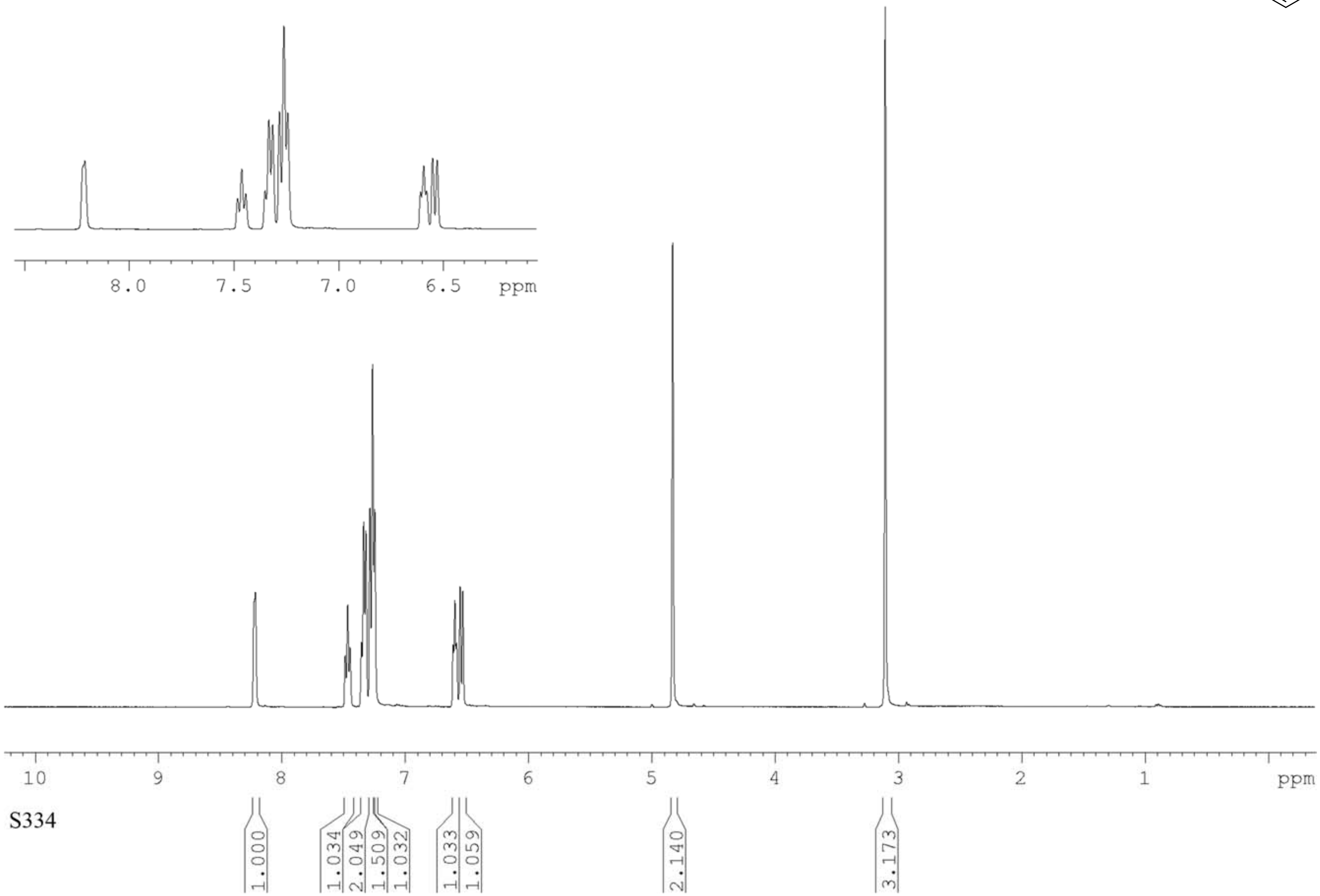

^{13}C NMR (101 MHz, $CDCl_3$) for ***N*-Benzyl-*N*-methyl-2-aminopyridine (1c)**

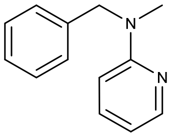

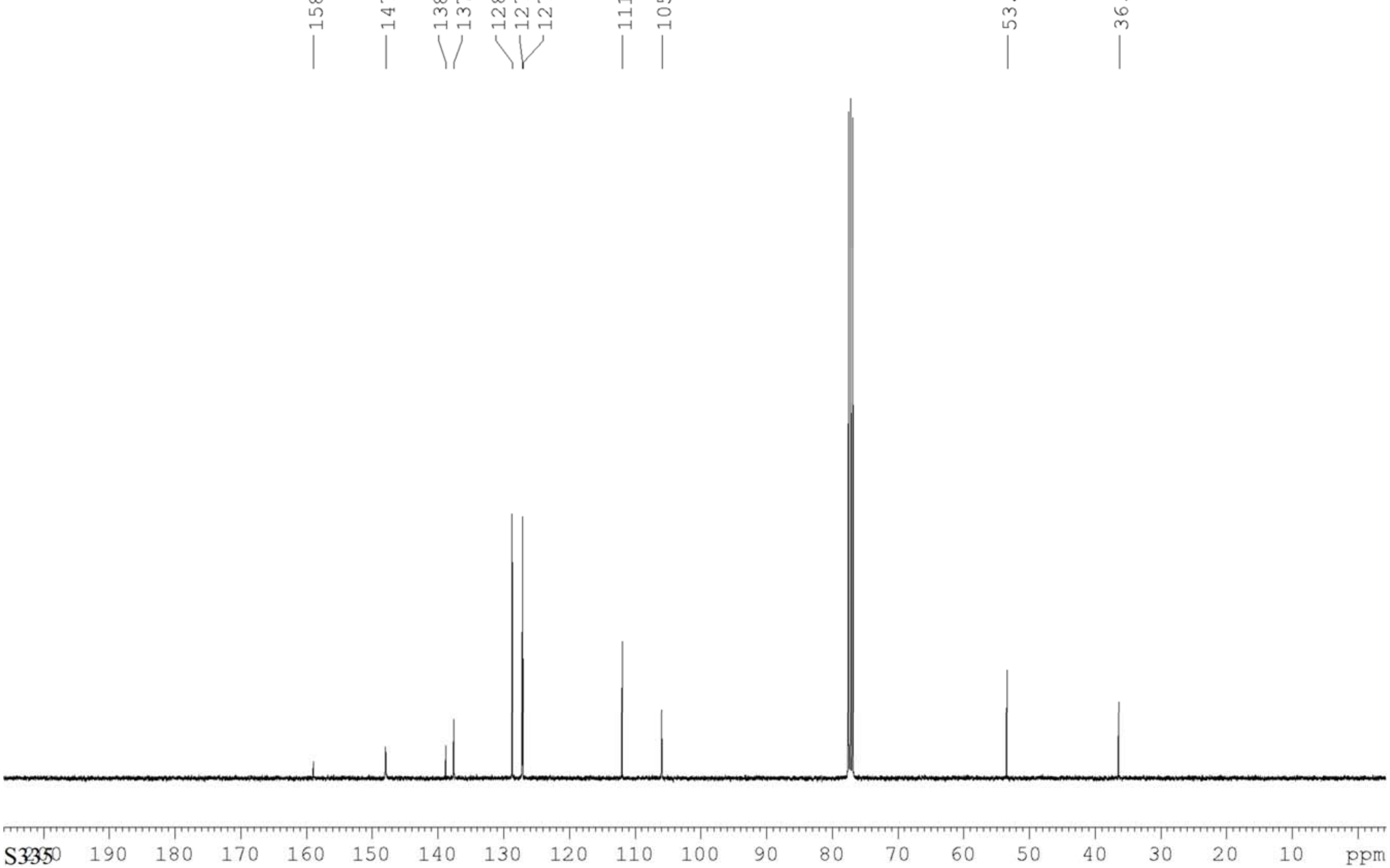

^{1}H NMR (400 MHz, $CDCl_3$) for ***N*-(4-(Trifluoromethyl)phenyl)pyridin-2-amine (1ka)**

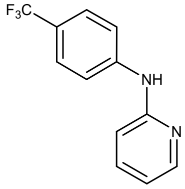

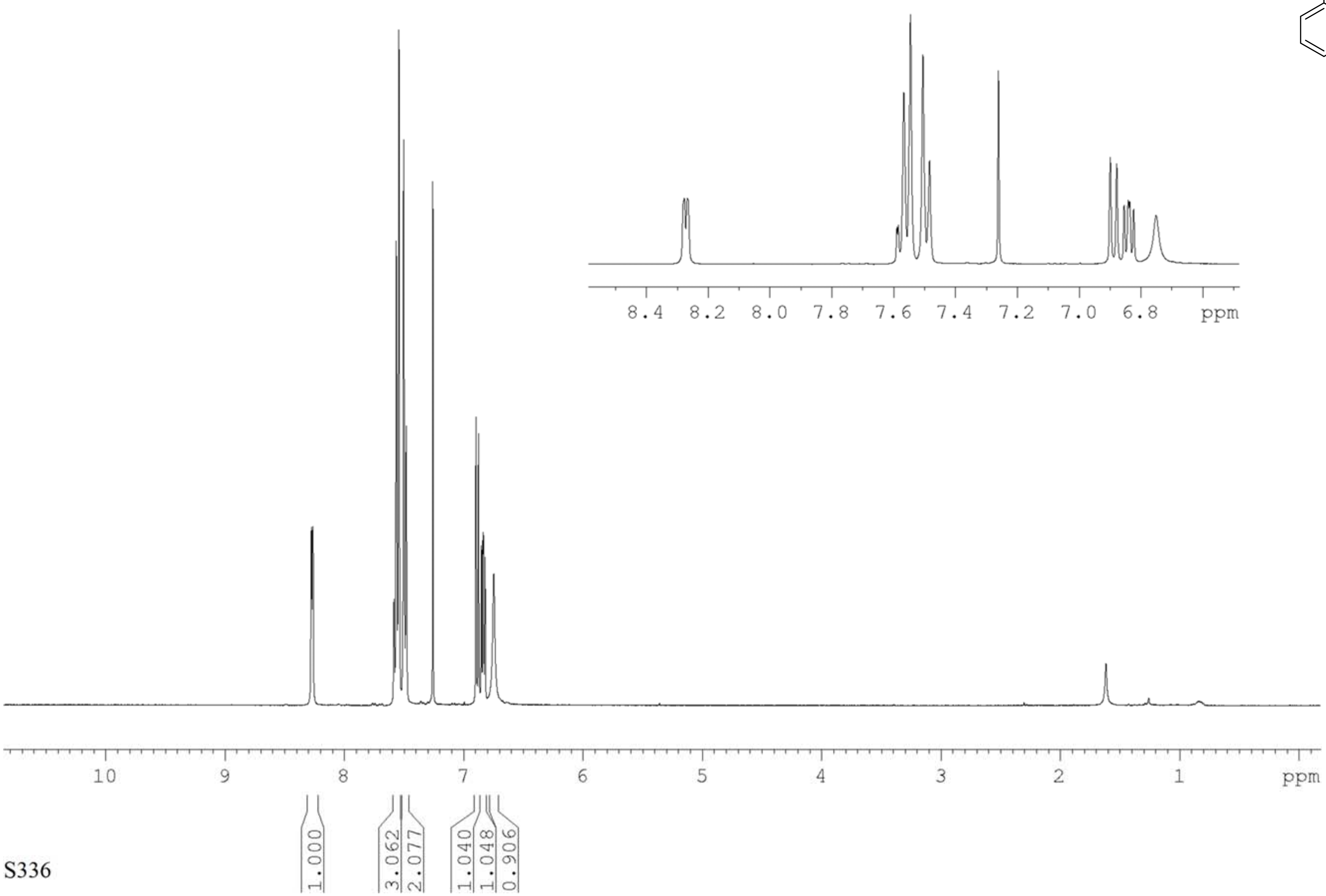

^{13}C NMR (101 MHz, $CDCl_3$) for ***N*-(4-(Trifluoromethyl)phenyl)pyridin-2-amine (1ka)**

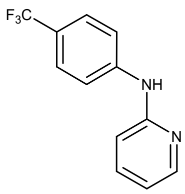

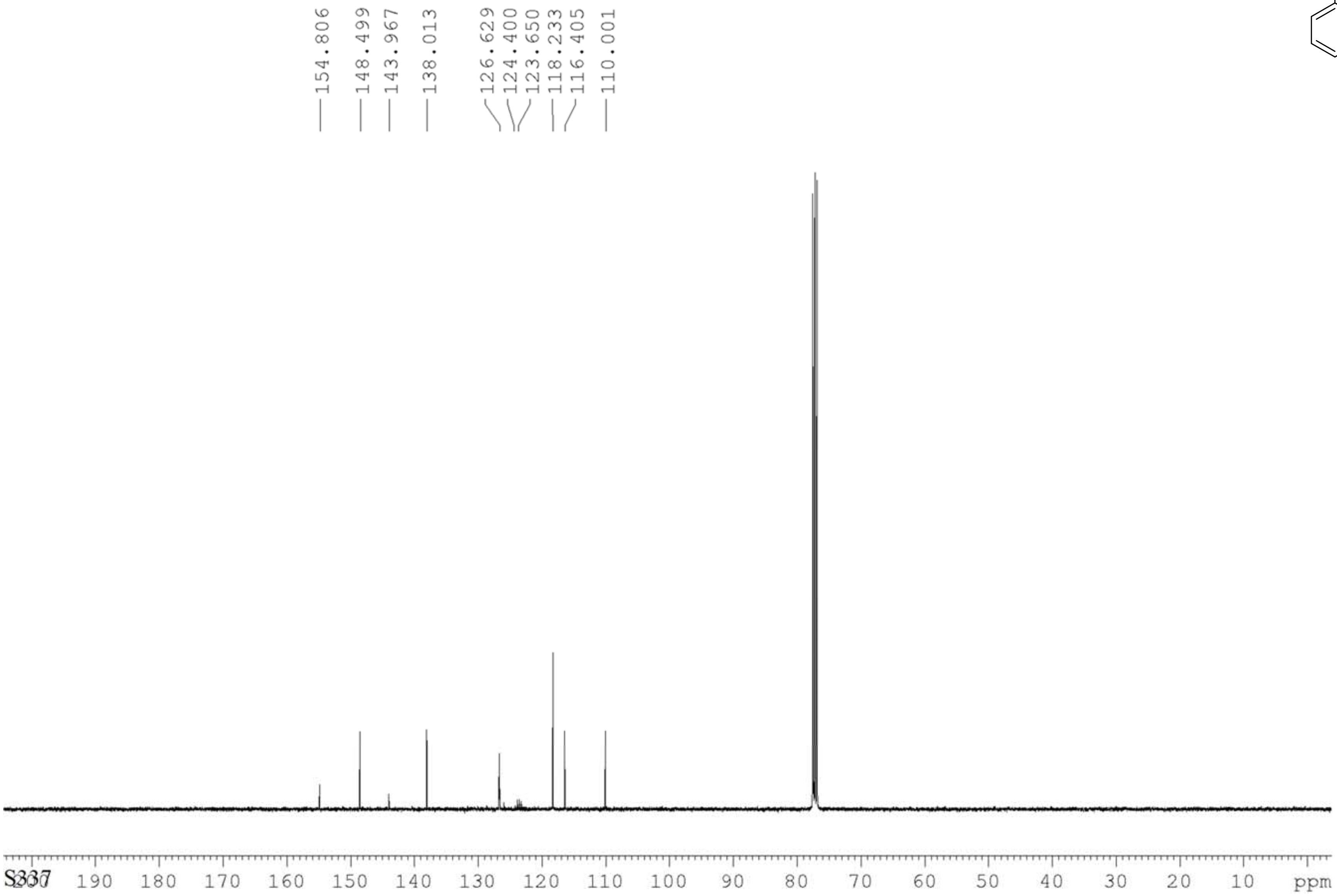

^{19}F NMR (471 MHz, $CDCl_3$) for ***N*-(4-(Trifluoromethyl)phenyl)pyridin-2-amine (1ka)**

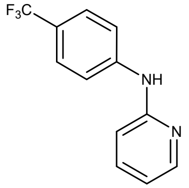

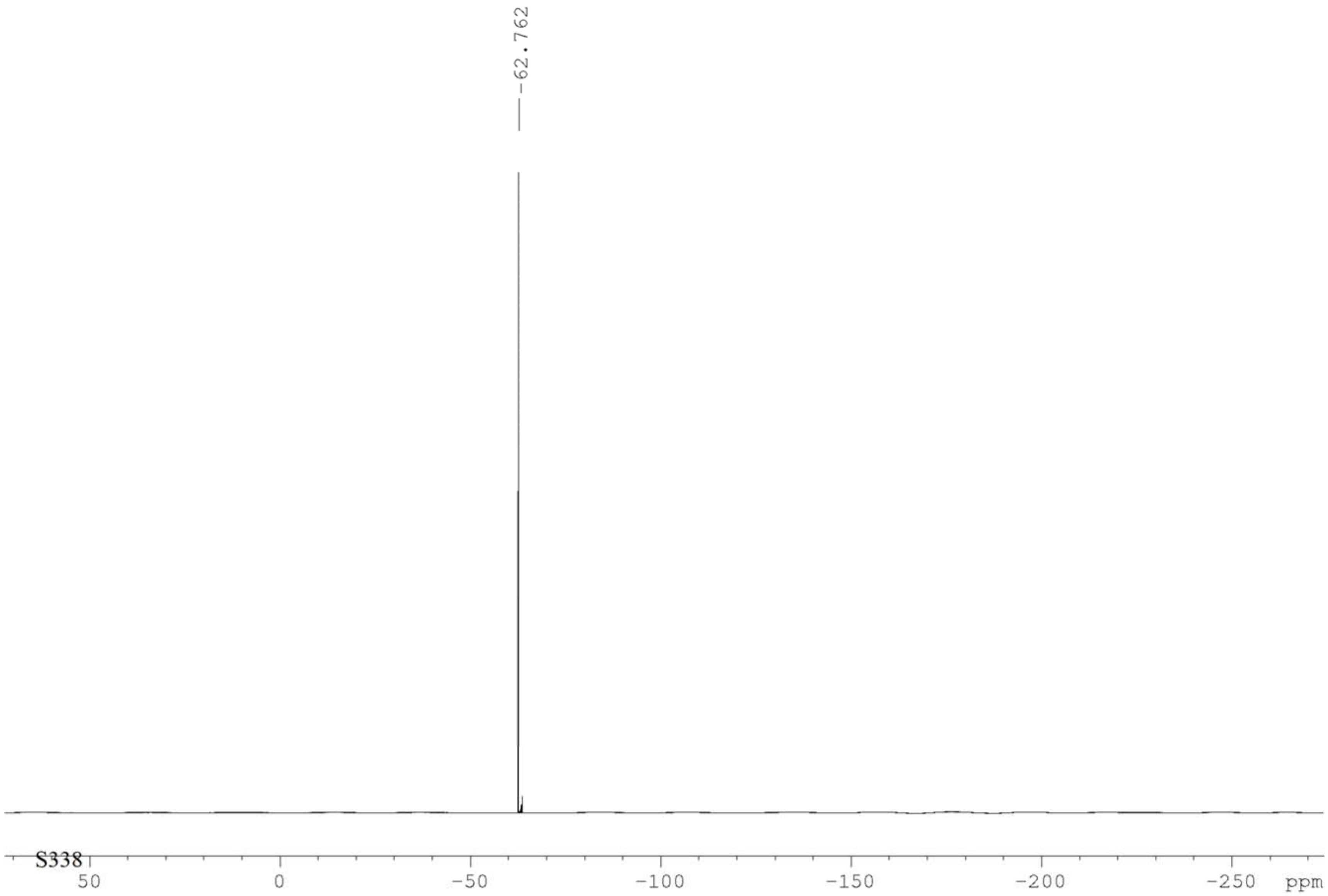

^{1}H NMR (500 MHz, $CDCl_3$) for ***N*-Methyl-*N*-(4-(trifluoromethyl)phenyl)-2-aminopyridine (1k)**

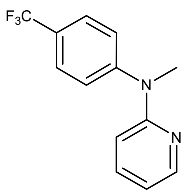

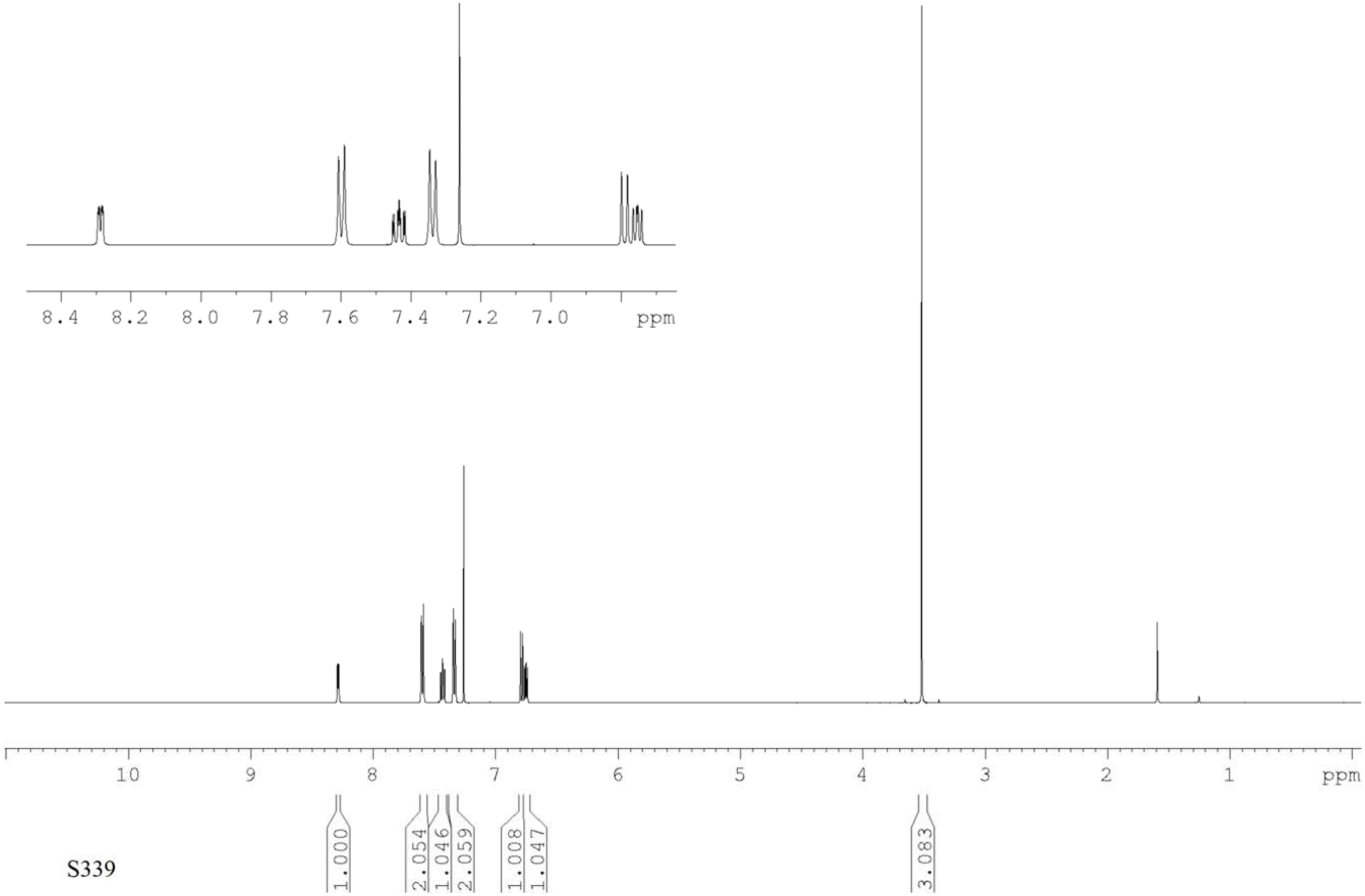

^{13}C NMR (126 MHz, $CDCl_3$) for ***N*-Methyl-*N*-(4-(trifluoromethyl)phenyl)-2-aminopyridine (1k)**

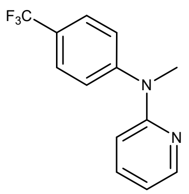

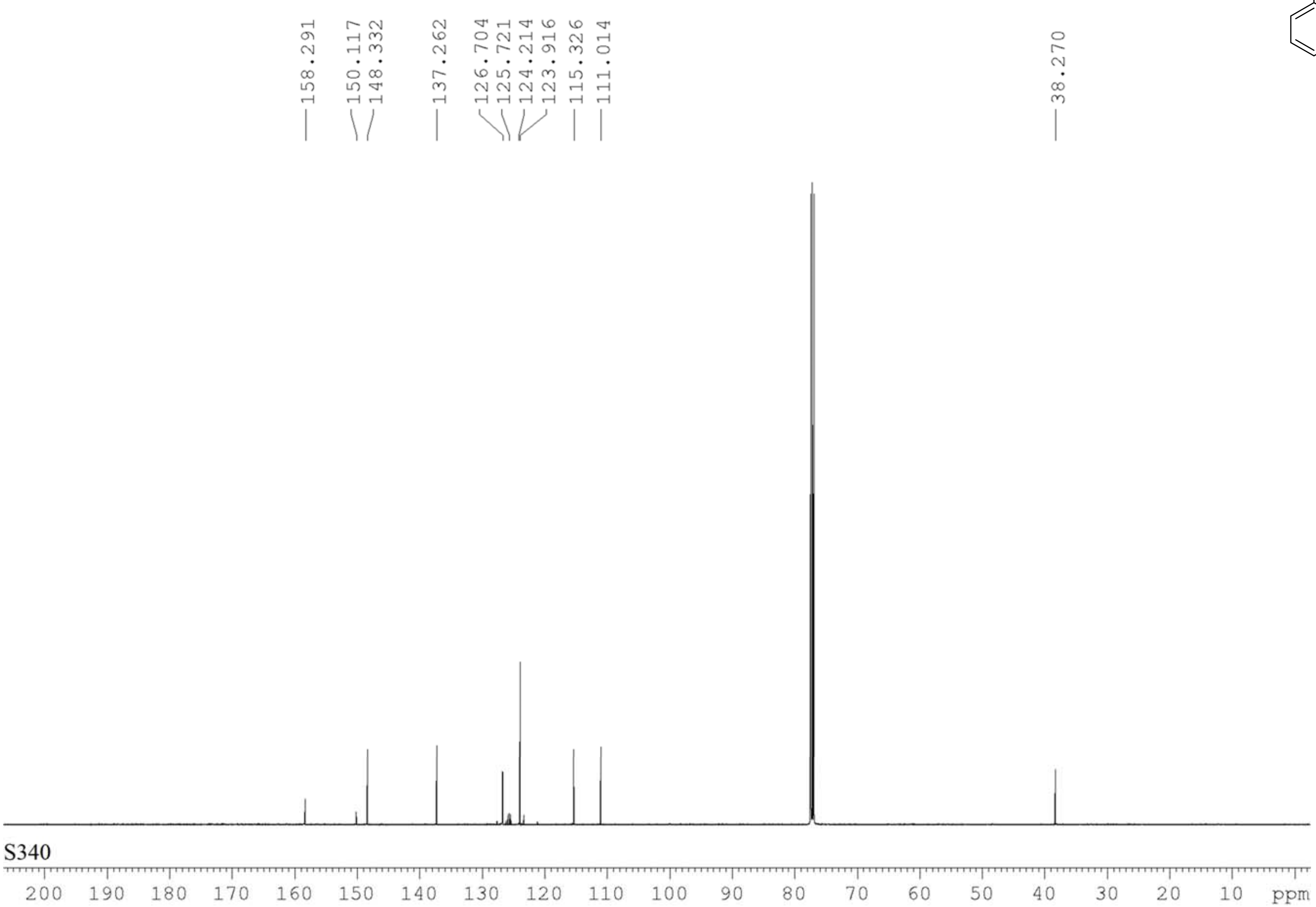

^{19}F NMR (471 MHz, $CDCl_3$) for ***N*-Methyl-*N*-(4-(trifluoromethyl)phenyl)-2-aminopyridine (1k)**

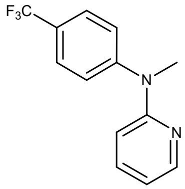

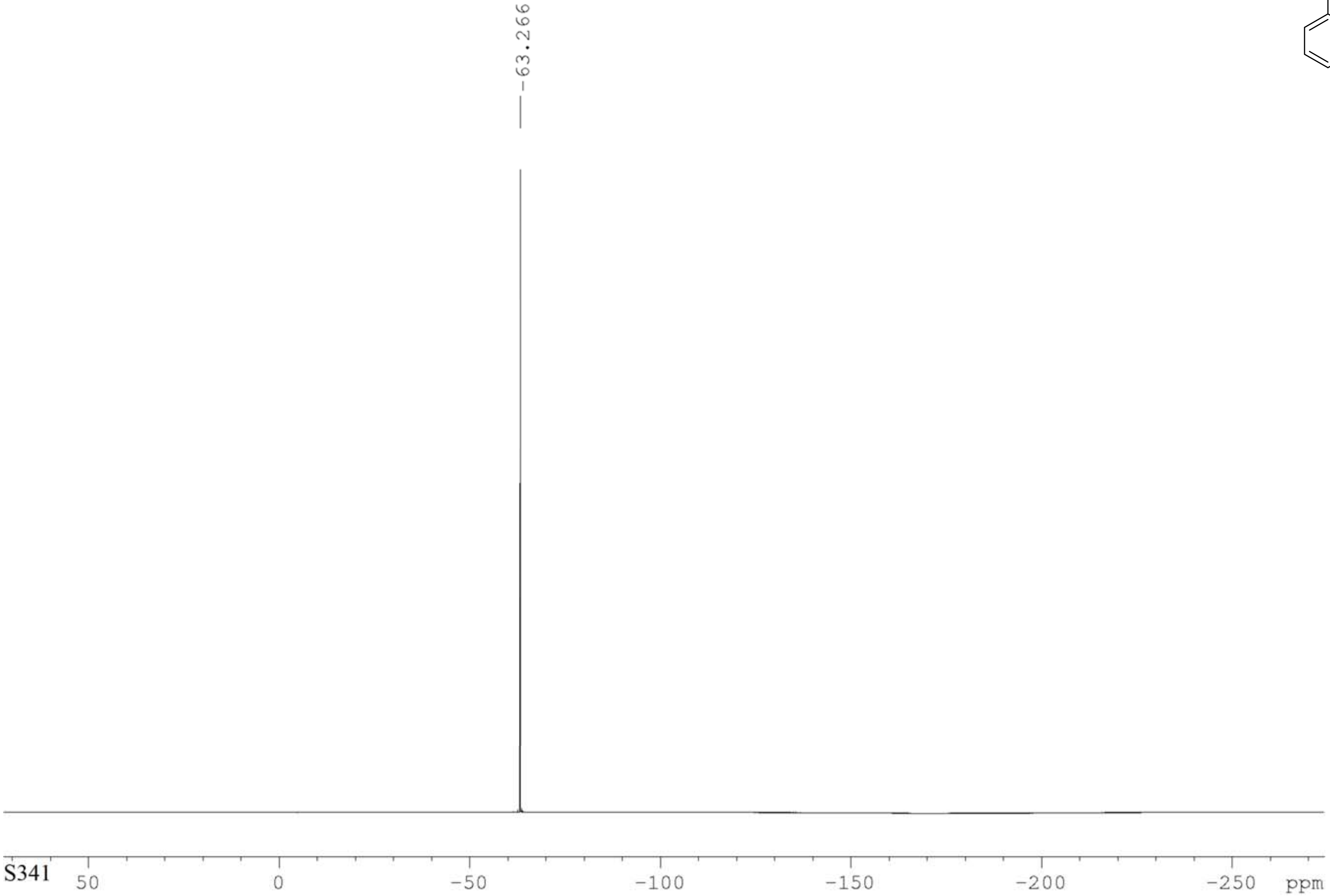

^{1}H NMR (400 MHz, $CDCl_3$) for **(*R*)-2-(3-((*tert*-butyldimethylsilyl)oxy)pyrrolidin-1-yl)pyridine (1b)**

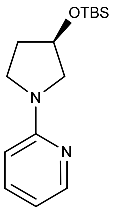

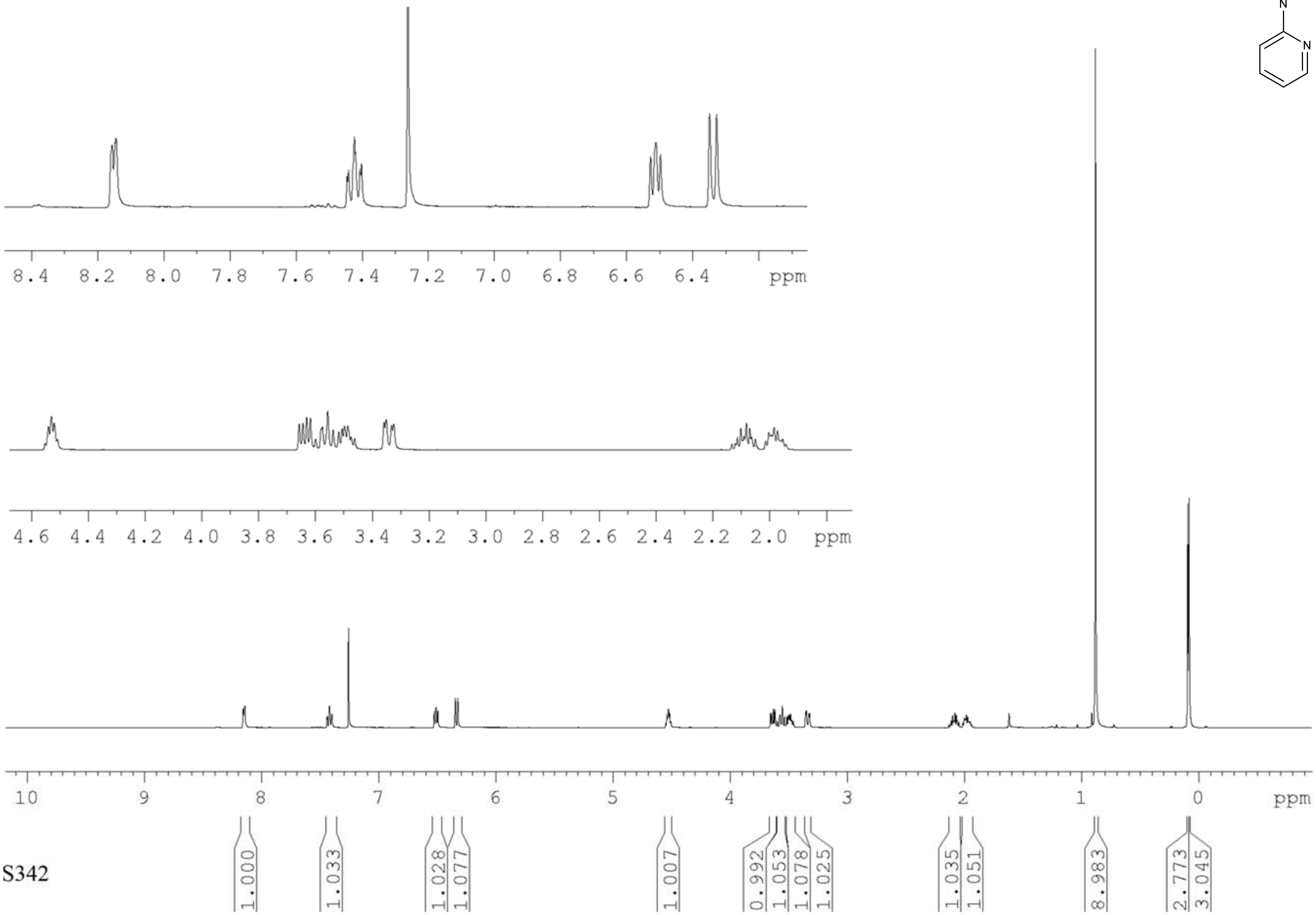

^{13}C NMR (101 MHz, $CDCl_3$) for **(*R*)-2-(3-((*tert*-butyldimethylsilyl)oxy)pyrrolidin-1-yl)pyridine (1b)**

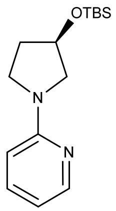

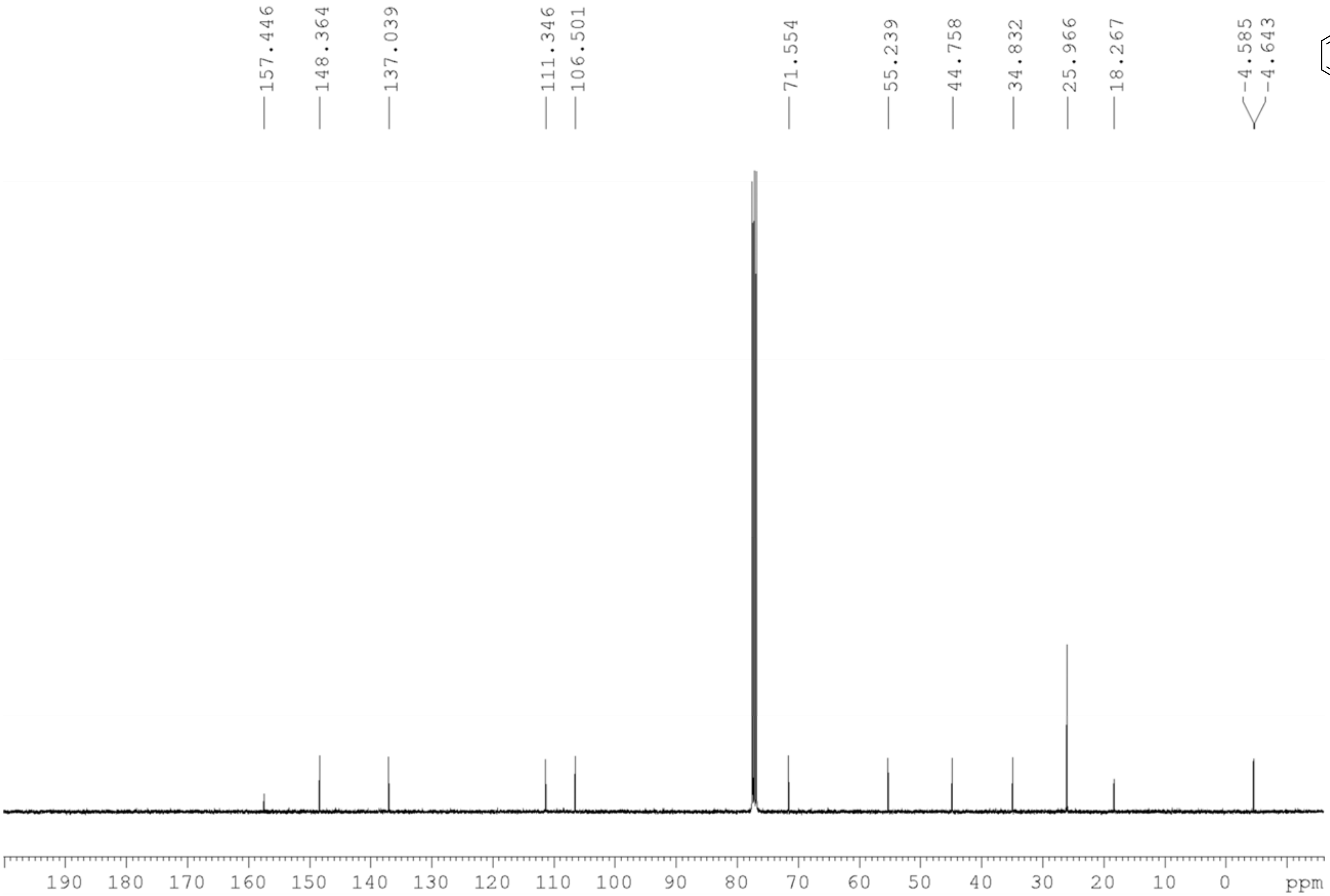

^{1}H NMR (400 MHz, $CDCl_3$) for **2-(Piperidin-1-yl)pyridine (1f)**

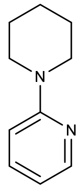

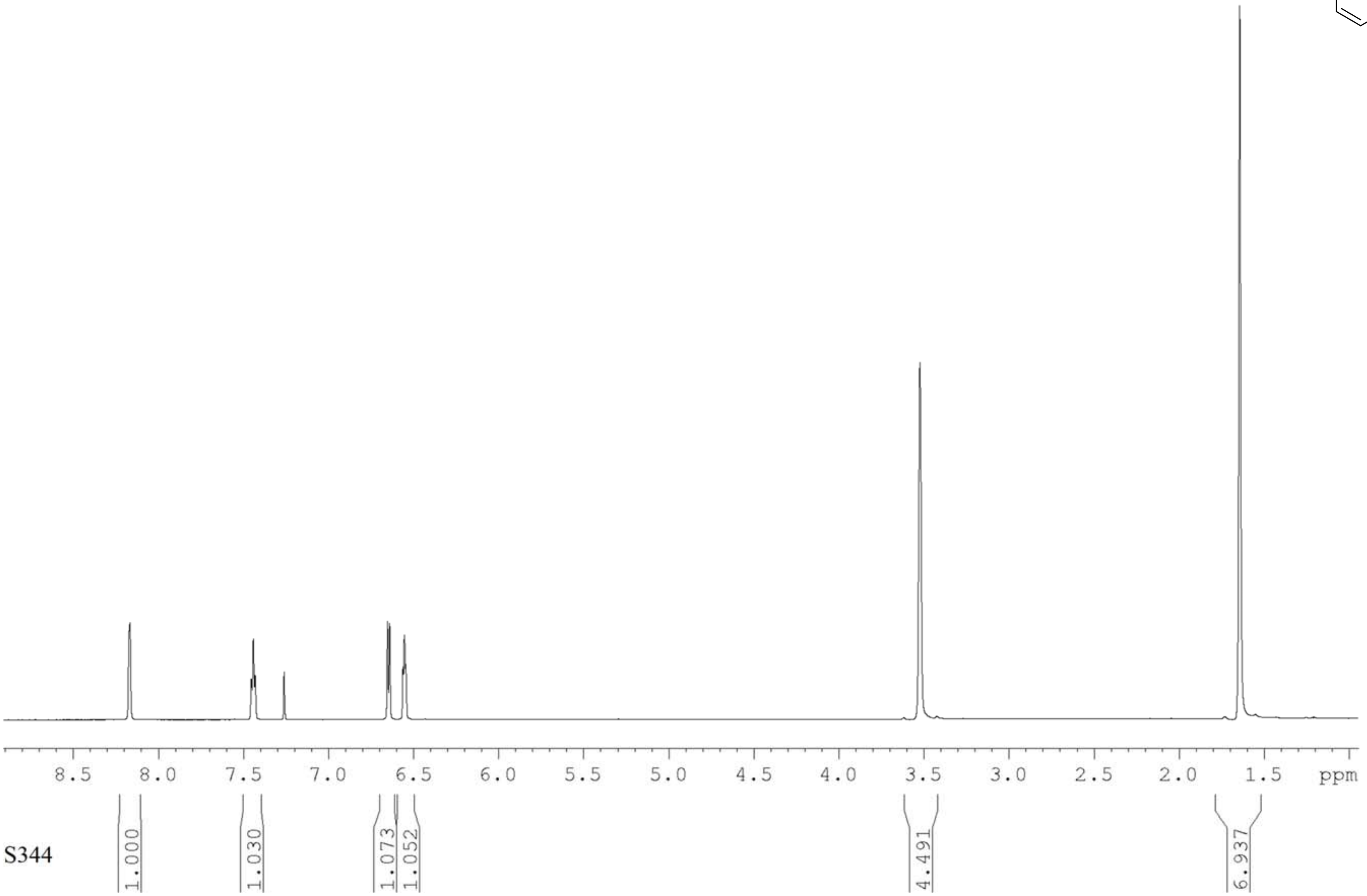

^{13}C NMR (176 MHz, $CDCl_3$) for **2-(Piperidin-1-yl)pyridine (1f)**

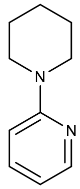

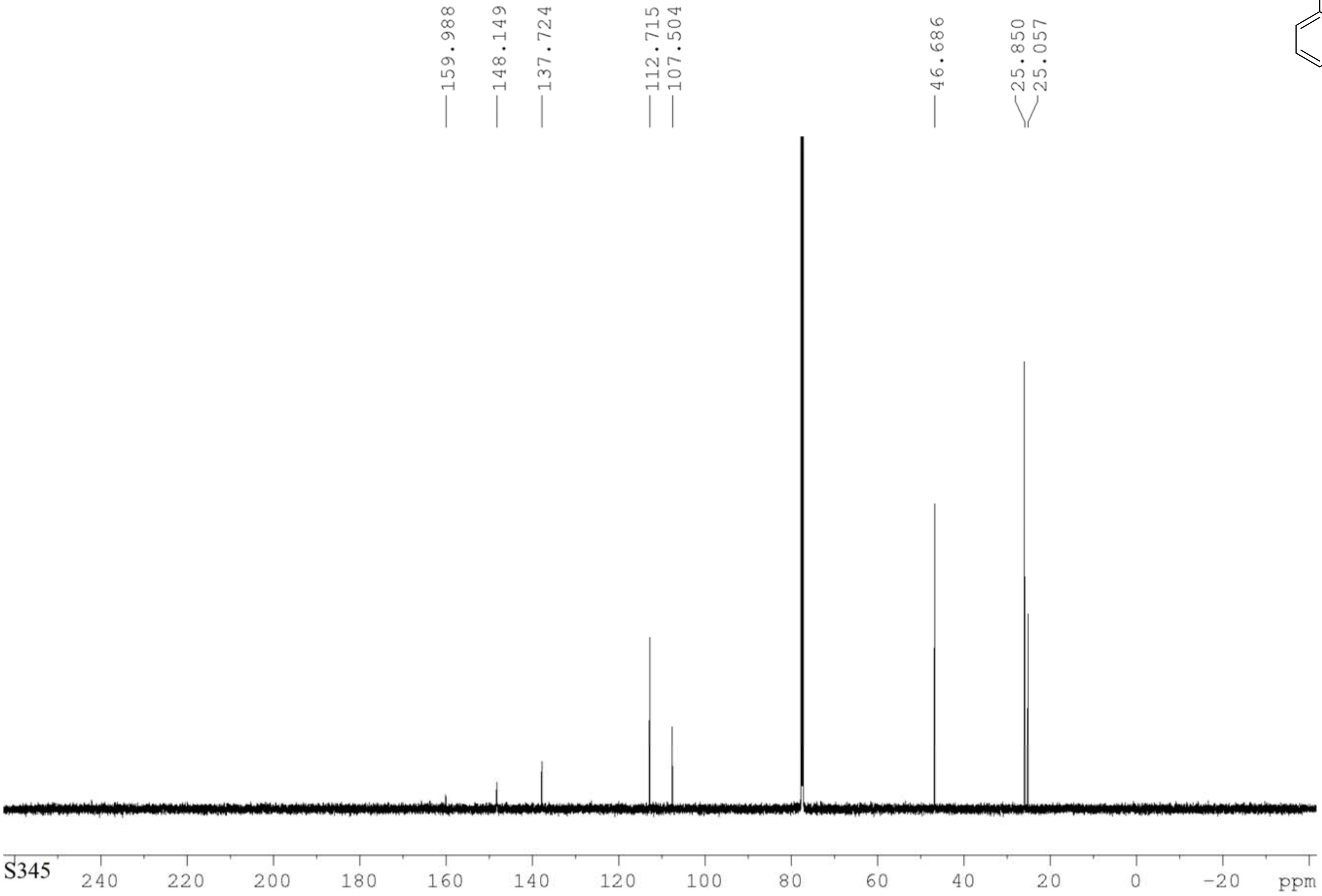

^{1}H NMR (500 MHz, $CDCl_3$) for **1-(Pyridin-2-yl)azepane (1g)**

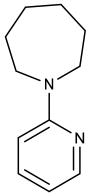

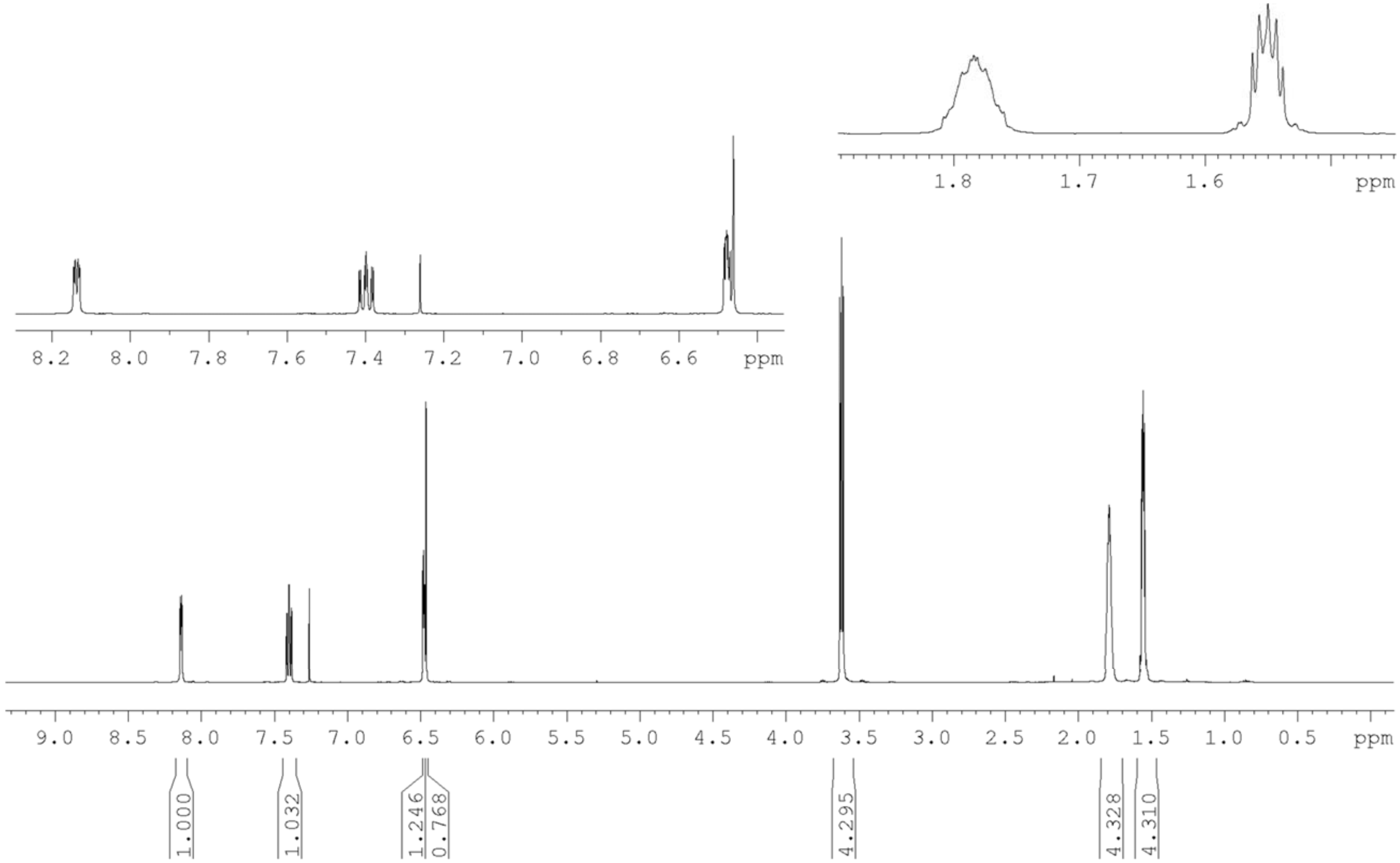

^{13}C NMR (126 MHz, $CDCl_3$) for **1-(Pyridin-2-yl)azepane (1g)**

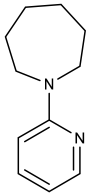

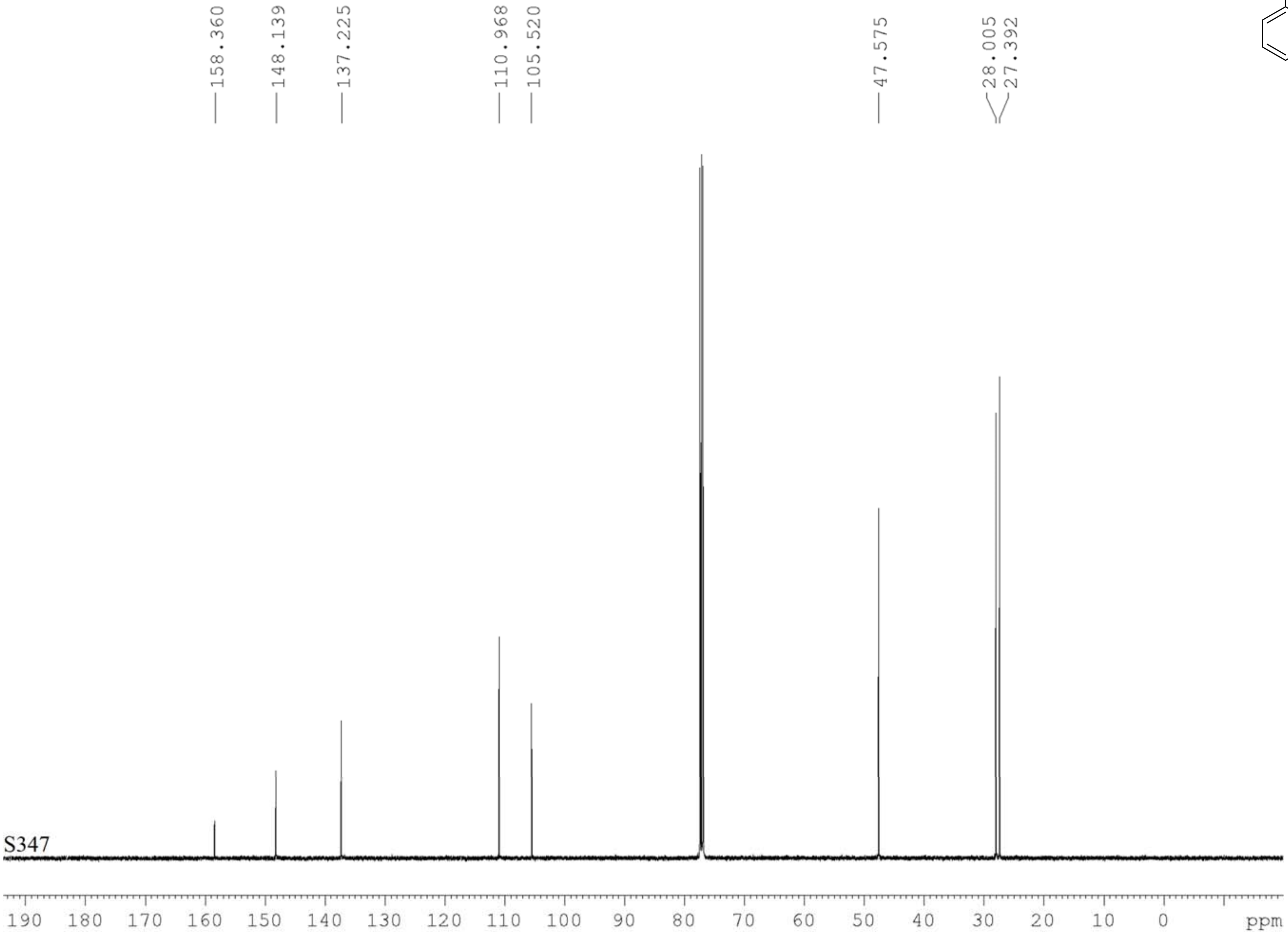

^{1}H NMR (500 MHz, $CDCl_3$) for ***N*-(2-((*tert*-Butyldimethylsilyl)oxy)ethyl)-*N*-methylpyridin-2-amine (1l)**

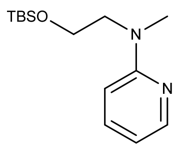

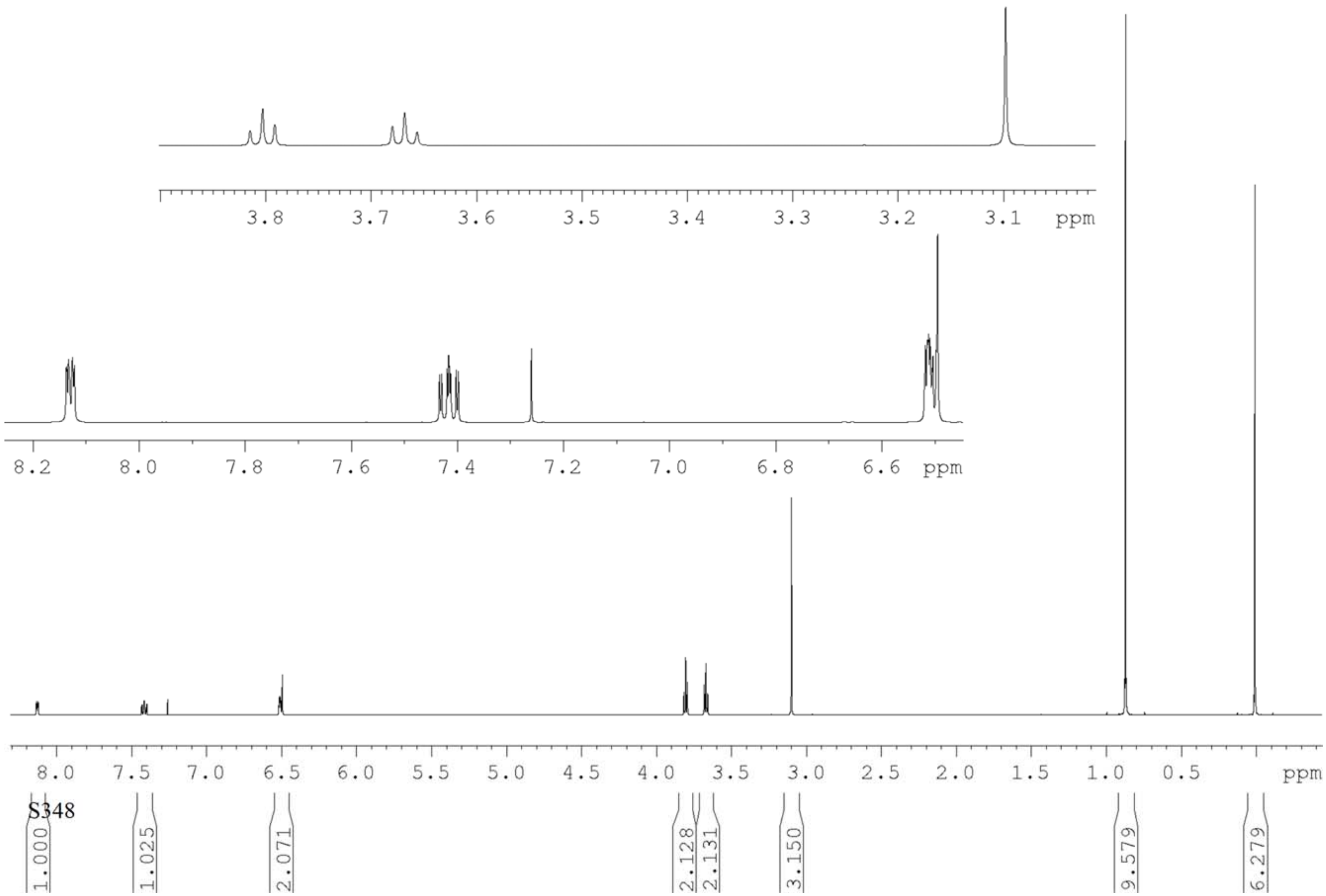

^{13}C NMR (126 MHz, $CDCl_3$) for ***N*-(2-((*tert*-Butyldimethylsilyl)oxy)ethyl)-*N*-methylpyridin-2-amine (1l)**

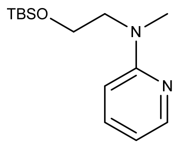

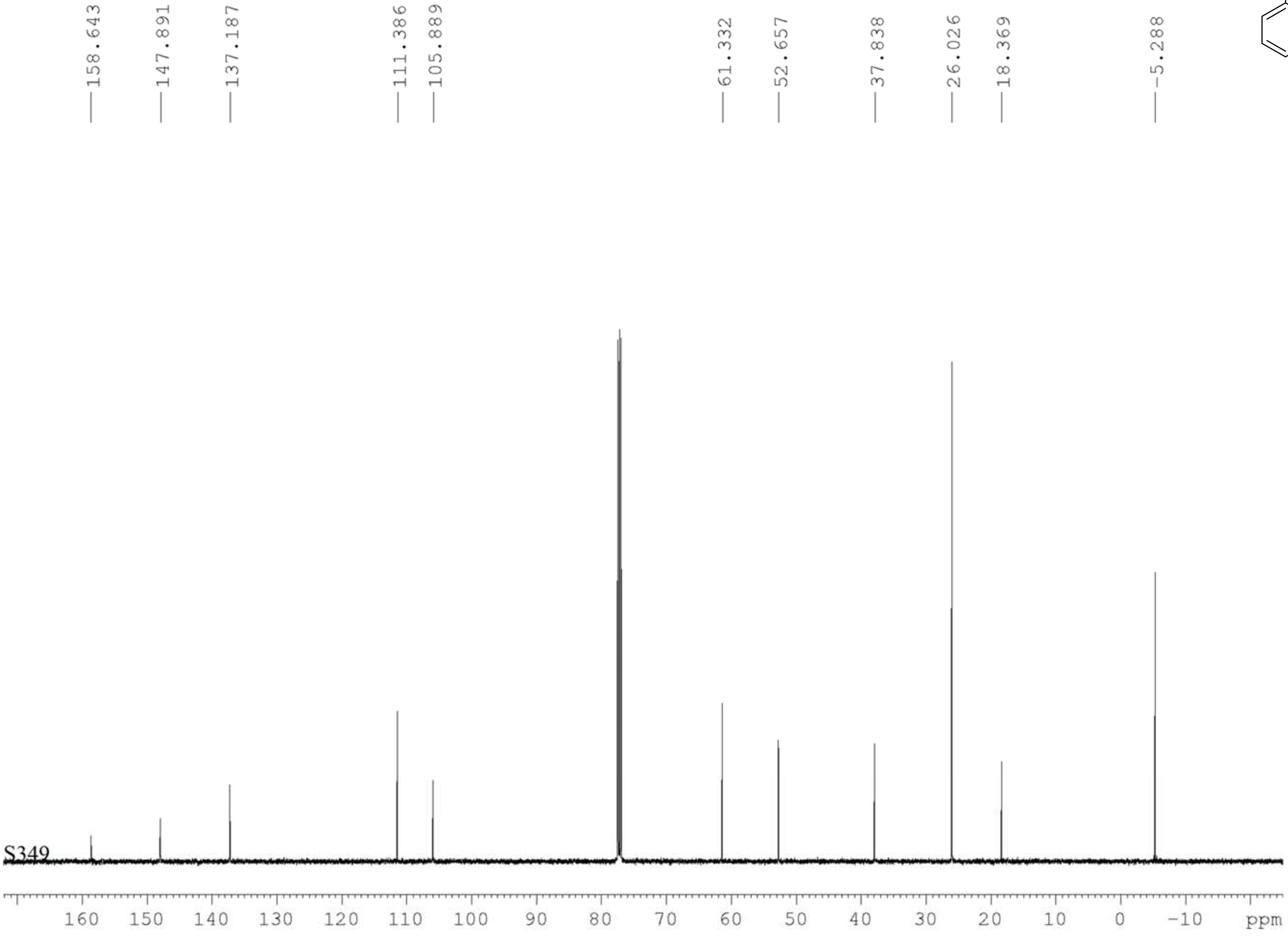

References

(1) Klussmann, M.; Ratjen, L.; Hoffmann, S.; Wakchaure, V.; Goddard, R.; List, B. Synthesis of TRIP and Analysis of Phosphate Salt Impurities. *Synlett* **2010**, *2010*, 2189–2192.

(2) Singh, A.; Teegardin, K.; Kelly, M.; Prasad, K. S.; Krishnan, S.; Weaver, J. D. Facile Synthesis and Complete Characterization of Homoleptic and Heteroleptic Cyclometalated Iridium(III) Complexes for Photocatalysis. *J. Organomet. Chem.* **2015**, *776*, 51–59.

(3) Zhu, Q.; Graff, D. E.; Knowles, R. R. Intermolecular Anti-Markovnikov Hydroamination of Unactivated Alkenes with Sulfonamides Enabled by Proton-Coupled Electron Transfer. *J. Am. Chem. Soc.* **2018**, *140*, 741–747.

(4) Jin, J. Y.; Zheng, M. H.; Wu, X.; Tian, G. R. Stereoselective Synthesis of (2 *S* , 8 *S*)-2-Benzyl Hexahydro-pyrrodizin-3-one Starting from (L)-Proline Based on Stereoselective Benzylation. *Synth. Commun.* **2004**, *34*, 3191–3196.

(5) Hargrave, J. D.; Bish, G.; Frost, C. G. Switching Stereoselectivity in Rhodium-Catalysed 1,4-Additions: The Asymmetric Synthesis of 2-Substituted Pyrrolizidinones. *Chem. Commun.* **2006**, No. 42, 4389–4391.

(6) SHENZHEN DONGYANGGUANG INDUSTRIAL DEVELOPMENT CO LTD. WO2014/5494A1, 2014.

(7) Honda, T.; Yamane, S.-I.; Naito, K.; Suzuki, Y. Chiral Synthesis of a Pyrrolizidine Alkaloid, (-)-Heliotridane. *Heterocycles* **1995**, *40*, 301–310.

(8) Sammis, G. M.; Jacobsen, E. N. Highly Enantioselective, Catalytic Conjugate Addition of Cyanide to α,β-Unsaturated Imides. *J. Am. Chem. Soc.* **2003**, *125*, 4442–4443.

(9) Felluga, F.; Pitacco, G.; Valentin, E.; Venneri, C. D. A Facile Chemoenzymatic Approach to Chiral Non-Racemic β-Alkyl-γ-Amino Acids and 2-Alkylsuccinic Acids. A Concise Synthesis of (S)-(+)-Pregabalin. *Tetrahedron Asymmetry* **2008**, *19*, 945–955.

(10) Hintermann, T.; Seebach, D. A Useful Modification of the Evans Auxiliary: 4-Isopropyl-5,5-Diphenyloxazolidin-2-One. *Helv. Chim. Acta* **1998**, *81*, 2093–2126.

(11) Birkholz, A.; Kopecky, D. J.; Volak, L. P.; Bartberger, M. D.; Chen, Y.; Tegley, C. M.; Arvedson, T.; McCarter, J. D.; Fotsch, C.; Cee, V. J. Systematic Study of the Glutathione Reactivity of N-Phenylacrylamides: 2. Effects of Acrylamide Substitution. *J. Med. Chem.* **2020**, *63*, 11602–11614.

(12) Wang, F.; Yang, H.; Fu, H.; Pei, Z. Efficient Copper-Catalyzed Michael Addition of Acrylic Derivatives with Primary Alcohols in the Presence of Base. *Chem. Commun.* **2013**, *49*, 517–519.

(13) Zhao, J.; Li, P.; Xu, Y.; Shi, Y.; Li, F. Nickel-Catalyzed Transformation of Diazoacetates to Alkyl Radicals Using Alcohol as a Hydrogen Source. *Org. Lett.* **2019**, *21*, 9386–9390.

(14) Wu, H.; Yang, B.; Zhu, L.; Lu, R.; Li, G.; Lu, H. High-Valent Palladium-Promoted Formal Wagner-Meerwein Rearrangement. *Org. Lett.* **2016**, *18*, 5804–5807.

(15) Schiffner, J. A.; Oestreich, M. All-Carbon-Substituted Quaternary Carbon Atoms in Oxindoles by an Aerobic Palladium(II)-Catalyzed Ring Closure onto Tri- and Tetrasubstituted Double Bonds. *European J. Org. Chem.* **2011**, *2011*, 1148–1154.

(16) Zhang, S.; Neumann, H.; Beller, M. Pd-Catalyzed Carbonylation of Vinyl Triflates to Afford α,β-Unsaturated Aldehydes, Esters, and Amides under Mild Conditions. *Org. Lett.* **2019**, *21*, 3528–3532.

(17) Sun, R.; Yang, X.; Chen, X.; Zhang, C.; Zhao, X.; Wang, X.; Zheng, X.; Yuan, M.; Fu, H.; Li, R.; Chen, H. Rh(III)-Catalyzed [4 + 2] Self-Annulation of N-Vinylarylamides. *Org. Lett.* **2018**, *20*, 6755–6759.

(18) Brittain, W. D. G.; Cobb, S. L. Carboxylic Acid Deoxyfluorination and One-Pot Amide Bond Formation Using Pentafluoropyridine (PFP). *Org. Lett.* **2021**, *23*, 5793–5798.

(19) Pezzetta, C.; Folli, A.; Matuszewska, O.; Murphy, D.; Davidson, R. W. M.; Bonifazi, D. *Peri* -Xanthenoxanthene (PXX): A Versatile Organic Photocatalyst in Organic Synthesis. *Adv. Synth.*

Catal. **2021**, *363*, 4740–4753.
(20) Fan, G. G.; Jiang, B. W.; Sang, W.; Cheng, H.; Zhang, R.; Yu, B. Y.; Yuan, Y.; Chen, C.; Verpoort, F. Metal-Free Synthesis of Heteroaryl Amines or Their Hydrochlorides via an External-Base-Free and Solvent-Free C-N Coupling Protocol. *J. Org. Chem.* **2021**, *86*, 14627–14639.
(21) Kim, S. H.; Park, S. H.; Chang, S. Palladium-Catalyzed Oxidative Alkynylation of Arene C-H Bond Using the Chelation-Assisted Strategy. *Tetrahedron* **2012**, *68*, 5162–5166.
(22) Bhujabal, Y. B.; Vadagaonkar, K. S.; Gholap, A.; Sanghvi, Y. S.; Dandela, R.; Kapdi, A. R. HFIP Promoted Low-Temperature SNAr of Chloroheteroarenes Using Thiols and Amines. *J. Org. Chem.* **2019**, *84*, 15343–15354.
(23) Thomas, S.; Roberts, S.; Pasumansky, L.; Gamsey, S.; Singaram, B. Aminoborohydrides 15. The First Mild and Efficient Method for Generating 2-(Dialkylamino)-Pyridines from 2-Fluoropyridine. *Org. Lett.* **2003**, *5*, 3867–3870.
(24) Pasumansky, L.; Hernández, A. R.; Gamsey, S.; Goralski, C. T.; Singaram, B. Synthesis of Aminopyridines from 2-Fluoropyridine and Lithium Amides. *Tetrahedron Lett.* **2004**, *45*, 6417–6420.
(25) Balkenhohl, M.; François, C.; Sustac Roman, D.; Quinio, P.; Knochel, P. Transition-Metal-Free Amination of Pyridine-2-Sulfonyl Chloride and Related N-Heterocycles Using Magnesium Amides. *Org. Lett.* **2017**, *19*, 536–539.
(26) Schönbauer, D.; Lukas, F.; Schnürch, M. Toluene and Its Derivatives as Atom-Efficient Benzylating Agents for Secondary Amines. *Synlett* **2019**, *30*, 94–98.
(27) Huang, F. D.; Xu, C.; Lu, D. D.; Shen, D. S.; Li, T.; Liu, F. S. Pd-PEPPSI-IPentAn Promoted Deactivated Amination of Aryl Chlorides with Amines under Aerobic Conditions. *J. Org. Chem.* **2018**, *83*, 9144–9155.
(28) Liu, J.; Jia, X.; Chen, X.; Sun, H.; Li, Y.; Kramer, S.; Lian, Z. Nickel-Catalyzed Intramolecular Desulfitative C - N Coupling: A Synthesis of Aromatic Amines. *J. Org. Chem.* **2020**, *85*, 5702–5711.
(29) Chen, J.; Natte, K.; Man, N. Y. T.; Stewart, S. G.; Wu, X. F. Convenient Copper-Mediated Chan-Lam Coupling of 2-Aminopyridine: Facile Synthesis of N-Arylpyridin-2-Amines. *Tetrahedron Lett.* **2015**, *56*, 4843–4847.
(30) D'andrea, S. V.; Freeman, J. P.; Szmuszkovicz, J. 2-(1-Azetidinyl)- and 2-(1-Pyrrolidinyl)Pyridine via Snar Reactions of 2-Fluoropyridine and 2-Pyridinyl Triflate. *Org. Prep. Proced. Int.* **1999**, *31*, 698–701.
